# Determination of lethal electric field threshold for pulsed field ablation in *ex vivo* perfused porcine and human hearts

**DOI:** 10.3389/fcvm.2023.1160231

**Published:** 2023-06-23

**Authors:** Bor Kos, Lars Mattison, David Ramirez, Helena Cindrič, Daniel C. Sigg, Paul A. Iaizzo, Mark T. Stewart, Damijan Miklavčič

**Affiliations:** ^1^ Faculty of Electrical Engineering, University of Ljubljana, Ljubljana, Slovenia; ^2^ Cardiac Ablation Solutions, Medtronic, Inc., Minneapolis, MN, United States; ^3^ Department of Surgery, Visible Heart® Laboratories, University of Minnesota, Minneapolis, MN, United States

**Keywords:** pulsed field ablation, lethal electric field thresholds, cardiac ablation, irreversible electroporation, muscle anisotropy

## Abstract

**Introduction:**

Pulsed field ablation is an emerging modality for catheter-based cardiac ablation. The main mechanism of action is irreversible electroporation (IRE), a threshold-based phenomenon in which cells die after exposure to intense pulsed electric fields. Lethal electric field threshold for IRE is a tissue property that determines treatment feasibility and enables the development of new devices and therapeutic applications, but it is greatly dependent on the number of pulses and their duration.

**Methods:**

In the study, lesions were generated by applying IRE in porcine and human left ventricles using a pair of parallel needle electrodes at different voltages (500–1500 V) and two different pulse waveforms: a proprietary biphasic waveform (Medtronic) and monophasic 48 × 100 μs pulses. The lethal electric field threshold, anisotropy ratio, and conductivity increase by electroporation were determined by numerical modeling, comparing the model outputs with segmented lesion images.

**Results:**

The median threshold was 535 V/cm in porcine ((*N* = 51 lesions in *n* = 6 hearts) and 416 V/cm in the human donor hearts ((*N* = 21 lesions in *n* = 3 hearts) for the biphasic waveform. The median threshold value was 368 V/cm in porcine hearts ((*N* = 35 lesions in *n* = 9 hearts) cm for 48 × 100 μs pulses.

**Discussion:**

The values obtained are compared with an extensive literature review of published lethal electric field thresholds in other tissues and were found to be lower than most other tissues, except for skeletal muscle. These findings, albeit preliminary, from a limited number of hearts suggest that treatments in humans with parameters optimized in pigs should result in equal or greater lesions.

## 1. Introduction

Pulsed Field Ablation (PFA) is an emerging non-thermal energy modality for intracardiac catheter-based ablation used for treatments of cardiac arrhythmias (1–3). The main mechanism of cell death associated with PFA is irreversible electroporation (IRE); in which cell membranes are disrupted by exposure of cells to intense short electric field pulses, causing cells to lose their abilities to maintain or recover homeostasis, leading to cell death (4–6). DC ablation was used for cardiac ablation, before radiofrequency ablation (RFA) became the dominant ablation modality (7, 8). Electroporation was shown to be involved with DC ablations as well as in defibrillation, as a response to the applied electric shocks (9, 10). Irreversible electroporation was (re)introduced as a promising method for cardiac ablation in 2007 (11) and has since been shown to be a successful procedure for ablation of both atrial (2, 12–17), and ventricular tissues (18–21). There are several potential advantages of PFA compared with existing thermal ablation modalities. First, it offers the potential for greater safety and less collateral damage compared with thermal ablation technologies (22–24). Second, PFA is a field-based technology and is not critically dependent on direct electrode-tissue contact and contact force (25, 26). In addition, PFA has the potential to increase procedural efficiency and reduce procedure times (22, 23). There are several recent reports showing minimal to minor effects on nerves and esophagus compared to RFA energy (27–29) as well as a reduction in pulmonary vein stenosis when energy is applied directly in the veins (30). The primarily non-thermal mechanisms of action have become widely accepted as an important safety aspect. Although it should be noted, that the non-thermality of the treatment also depends on the selected pulse parameters, electrode designs, and ultimate fine-tuning between those two factors (31, 32). There are currently numerous ongoing clinical trials in multiple centers to gain experiences with PFA, with the first reported results in treating both paroxysmal and persistent atrial fibrillation are promising (33–36).

Electroporation as a method of inducing cell death is considered a threshold-based mechanism, as sufficiently strong local electric fields in the target tissue are required to cause increases in transmembrane voltages, and if sufficiently high, it results in membrane bilayer breakdown (6, 37). The strengths of the electric fields in the tissue leading to cell death, hereafter referred to as the lethal electric field threshold (LET), are expressed in units of V/cm and typically lie in the range of 500–1,000 V/cm (38–42). LET depends on the type of tissue and even more so on the selected pulse parameters (43, 44). It has been claimed that myocardium is more sensitive to electric fields (i.e., has a lower lethal threshold) than other tissues, but this was largely based on chronic preclinical data and informed by experimental *in vitro* data (45, 46). The lower thresholds of cardiac myocytes was in part confirmed by more recent *in vitro* studies (47, 48), however, the differences were much smaller than what were reported by Kaminska et al. (45). Interestingly, Avazzadeh et al. (49) showed that some types of neurons are even more sensitive than cardiomyocytes, while Sowa et al. (50) found only 20% of dead cells after exposure to fields of 2 kV/cm, so the data available thus far seems inconclusive. Furthermore, due to the specifics of *in vitro* cell experimental setups, thresholds determined in this manner cannot be easily extrapolated to *in vivo* tissue environments (51–53). Interestingly, in recent skeletal muscle study authors found an even lower LET value of 193 V/cm (54). In cardiac tissue, LET has so far been experimentally determined in a single study in rats (55), where the authors report 640 V/cm for 10 × 100 μs monophasic pulses.

LET values determined in *in vivo* studies are typically dependent on the specific study design, i.e., the selection of experimental animals, the method and chosen time point of the outcome assessment, and the numerical model used to calculate the electric field (56). Electroporation thresholds also depend on the pulse protocol, i.e., the number and duration of applied pulses (57). In other words, electroporation may occur at a lower threshold if more pulses or longer pulses are applied or vice versa. However, the thresholds for electroporation should not depend on the electrode arrangement chosen and the voltage applied, but only on the local electric field, since the cells are locally exposed to this electric field. While the distribution of the electric field clearly depends on the electrode arrangement and the applied voltage, the determined LET should only depend on the chosen pulse protocol and not on the electrode geometry or the applied voltage. When multiple electrodes are used, certain regions of the tissue are cumulatively exposed to more pulses than others due to the overlapping fields of the different electrode pairs, effectively extending the exposure time. An overlap can reduce the LET, particularly for treatments with low pulse numbers (58).

Knowledge of underlying LET (for different tissues) plays very important roles in the design and development of catheters and pulse generators; as the electric field and its distribution is a crucial factor determining the efficacy of electroporation-based treatments (59). Therefore, calculations of LET based on tissue experiments are crucial for the development of the field of PFA for cardiac applications. Within the present study, we aimed to: (1) determine the LET for proprietary biphasic Medtronic waveform (MDT) in healthy isolated porcine hearts and in isolated human hearts; (2) determine the LET in porcine hearts for the most most reported monophasic 100 μs IRE pulses; (3) compare the LET values obtained with different waveforms and tissues; and (4) compare the obtained LET with existing literature data obtained from *in vivo* experiments.

## 2. Materials and methods

### 2.1. Experimental design: overview

This research protocol was approved by the Institutional Animal Care and Use Committee of the University of Minnesota. Human tissues were provided via a donor network (The International Institute for the Advancement of Medicine, Edison, NJ, USA) after Institutional Review Board approval and informed consent.

### 2.2. Isolated porcine heart preparation

Isolated porcine hearts were prepared (*n* = 6) as previously described (60). In brief, the isolated heart preparation is perfused using a modified right-sided working mode setup, perfused with Krebs-Henseleit buffer. Sinus rhythm and physiological temperatures (37°C) were maintained throughout the experiments.

### 2.3. Isolated human heart preparation

Isolated human hearts (*n* = 3) were prepared from donors (61, 62). In brief, the isolated human heart preparations were perfused using either a Langendorff (*n* = 2) or modified Langendorff (*n* = 1, same as the porcine preparation) set up with Krebs-Henseleit buffer (see Table 1). Physiological temperatures (37°C) were maintained throughout the experiment. Langendorff perfused human hearts showed electrical and mechanical activity but were not defibrillated into normal sinus rhythm to provide a simplified experimental set up, whereas in the modified Langendorff preparation the heart was defibrillated into normal sinus rhythm. In all cases the coronary arteries were perfused with an oxygenated buffer to ensure tissue viability.

**Table 1:** Human heart donor characteristics.

Heart	Sex	Donor age	Cause of death	Preparation	Relevant medical history
1	F	44	Anoxia—drug OD	Modified Langendorff	Excessive alcohol consumption
2	M	62	Stroke	Langendorff	Hypertension
3	F	58	Head trauma	Langendorff	Hypertension, diabetes

### 2.4. Electrode geometry and pulse protocols

**Figure 1:**
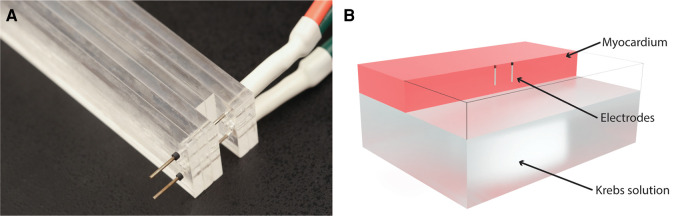
Electrodes and computational model. (**A**) Photograph of the electrodes used in the experiments. (**B**) Computational model: electrodes were modeled as cylinders with a diameter of 0.7 mm and a center-to-center distance of 8 mm. The total active length of 7 mm was inserted 8 mm into the upside of the ventricle, with the topmost 1 mm of the electrode insulated. The model included a layer of Krebs solution on the inside of the ventricle. For clarity, the myocardium is transparent at the location of the electrodes, so only the wireframe of the model is shown in that part of the model.

The pulses were delivered to the left ventricular tissues via a pair of parallel needle electrodes. The electrodes had an active length of 7 mm, diameter of 0.7 mm, and a center-to-center distance of 8 mm (Figure 1) (42, 63). A voltage probe was connected directly at the electrode terminals to measure the delivered voltages with a MSOS104A (Keysight, Santa Rosa, CA) oscilloscope, while the currents were measured as voltages on an internal shunt resistor of the pulse generator. A Medtronic research pulse generator was used to deliver the pulses (Medtronic, Minneapolis, MN) (2).

Two different pulse protocols were used: a biphasic Medtronic pulse protocol (23) and a monophasic IRE protocol. The Medtronic pulse protocol (MDT) consisted of 4 individual trains of biphasic pulses with an approximately 2 s pause between each train. The pulses were delivered at the following voltage levels: 500, 700, 1000, 1100, 1200, 1300, and 1500 V. These voltage levels were consistent with previous preclinical studies in pigs (25, 26, 30, 64).

The monophasic IRE protocol consisted of 6 trains of 8 monophasic pulses with a 100 μs duration, consistent with a recent study on skeletal muscle (54). The pulse delivery frequency was 1 kHz with an approximately 3 s delay between each train. These pulses were delivered at the following voltage levels: 600, 800, 1,000, and 1,200 V. The rationale for choosing the number of pulses was the following: the intention was to keep the protocol as close to the most researched and reported at 90 × 100 μs pulses. However, with that number of pulses, the lesions were too large, which would lead to requiring too many animals. Therefore, the number of pulses was reduced, to keep total lesion areas comparable to the ones with MDT protocol. The voltage was also reduced for the same reason, but multiple voltages were always tested to ensure that the obtained LET was independent of the applied voltage.

### 2.5. Image processing

All lesions were allowed to mature for 60–90 min following the series of energy applications; i.e., before perfusion was ended. Afterwards, the given heart was removed from the perfusion apparatus. Ventricular tissue sections containing the lesions were sliced 4 mm below the epicardial surface. Fresh lesioned tissues were submerged in the cell viability stain [Triphenyl Tetrazolium Chloride (TTC)] for 3–5 min until the borders were clearly demarcated (65). Lesions were then photographed with a scale bar in plane. Lesion images were processed using ImageJ (NIH Bethesda, MD) (66). Images were imported and key landmarks were located (pin location and muscle fiber orientation). A manual thresholding of the lesion was performed to create a binary alive(black)/dead(white) segmentation of the lesion.

The locations (centers) of the pin electrodes were marked on the lesion images with two gray pixels, while the direction of the fibers was indicated by two red pixels. The segmented image tiff files were then imported into Matlab (R2022a, The MathWorks Inc., Natick, MA), rotated, and scaled so that the location of the centers of the electrodes was placed at coordinates (−4,0) and (4,0). This compensated for the varying rotations and magnifications of the photographs. The images were then resampled to a standardized grid with a 0.02 mm pixel size. The angles of the fibers relative to the line connecting the two electrodes were determined from the marked pixels. The lesion images were then processed using a morphological open operation with a disk imaging element with a radius of 3 pixels to remove small islands away from the main lesion. The grayscale images obtained this way were then used to compare experimental images with the results of the numerical model.

### 2.6. Numerical modeling

A simplified numerical model of the *ex vivo* heart ventricle was constructed in COMSOL Multiphysics (version 6.0, COMSOL AB, Stockholm, Sweden), and the simulations were setup and ran automatically using the Live link to Matlab. The model consisted of two cuboid volumes. One cuboid represented the myocardial wall of the ventricle with dimensions of 80 mm × 60 mm × 10 mm, while the other, with dimensions of 80 mm × 60 mm × 20 mm, represented the Krebs buffer, which filled the lumen of the ventricle. The geometry is shown in Figure 1B. Since two of the Human hearts were not restored to sinus rhythm (Hearts No. 2 and 3), their chambers were not filled with the Krebs solution. In these cases, the lower block was removed, and an insulating boundary condition was used on the lower side of the ventricle to represent an air pocket inside the chamber. The numerical model was used to reconstruct and match the lesion shapes of the model to the experimental lesions obtained with TTC staining.

The numerical model included an anisotropic representation of the tissue conductivity (67), and an electric field dependent increase in conductivity, using the built in COMSOL function for a smoothed Heaviside function with two continuous derivatives (68). Similarly, the anisotropy ratio in cardiac muscle has been previously described to be between 1.5 and 4 (69–72). Because the fibers in the ventricle exhibit twisted anisotropy—i.e., their orientation changes from the epicardial to the endocardial side of the ventricle wall (73), the exact fiber orientation was not always possible to predict at the 4 mm depth of slicing. The total increase in conductivity due to electroporation has been shown to range 2 and 4 for other tissues (68, 74–76), but it had not yet been determined for cardiac tissues.

The conductivities in parallel to the fibers and perpendicular to the fibers were related by the parameter AR:

$$AR=\frac{\sigma_{∥}}{\sigma_{⊥}}$$

Where $\sigma_{∥}$ represents the conductivity in parallel with the fibers (0.5 S/m), and $\sigma_{⊥}$ represents conductivity perpendicular to the fibers (value adjusted by optimization algorithm). The choice of value $\sigma_{∥}$ is on the higher end of the reported values from the literature (71), which also aligns with higher interstitial fluid volume in perfused hearts (77). The conductivity increases due to electroporation were implemented using the COMSOL smoothed Heaviside function with two continuous derivatives. The function *f_E_* presented a smooth increase from 1 to the value of the electroporation conductivity increase factor (EF) parameter. The center of the transition region was fixed at 550 V/cm and the width of the transition region was 500 V/cm (78).

The final equation for anisotropic conductivity was:

$$\sigma (E,\phi )=\left\{\begin{array}{ccc} \left(\sigma_{∥}\cdot \;cos^{2}(\nu (z))+\sigma_{⊥}\cdot \;sin^{2}(\nu (z)))\cdot f_{E}(\|E\;\|\right) & \cos(\nu (z))\cdot \sin(\nu (z))\cdot (\sigma_{∥}-\sigma_{⊥})\cdot f_{E}(\|E\;\|) & 0\\ \cos(\nu (z))\cdot \sin(\nu (z))\cdot (\sigma_{∥}-\sigma_{⊥})\cdot f_{E}(\|E\;\|) & \left(\sigma_{∥}\cdot \;sin^{2}(\nu (z))+\sigma_{⊥}\cdot \;cos^{2}(\nu (z){)}^{2})\cdot f_{E}(\|E\;\|\right) & 0\\ 0 & 0 & \sigma_{⊥}\cdot f_{E}(\|E\;\|) \end{array}\right\},$$

where ν(z) is the function for the angle of the fibers. The function ν(z) was defined as ν(z)=(z+4)/10π+θ, where *z* is the height coordinate in mm, and *θ* is the angle measured from the images. This allowed the model to take into account the twisted anisotropy of changing fiber orientation by 180° from the epicardial to the endocardial surface (79), while matching the observed angle of the fibers at the location of the slice for each specific data point.

**Figure 2:**
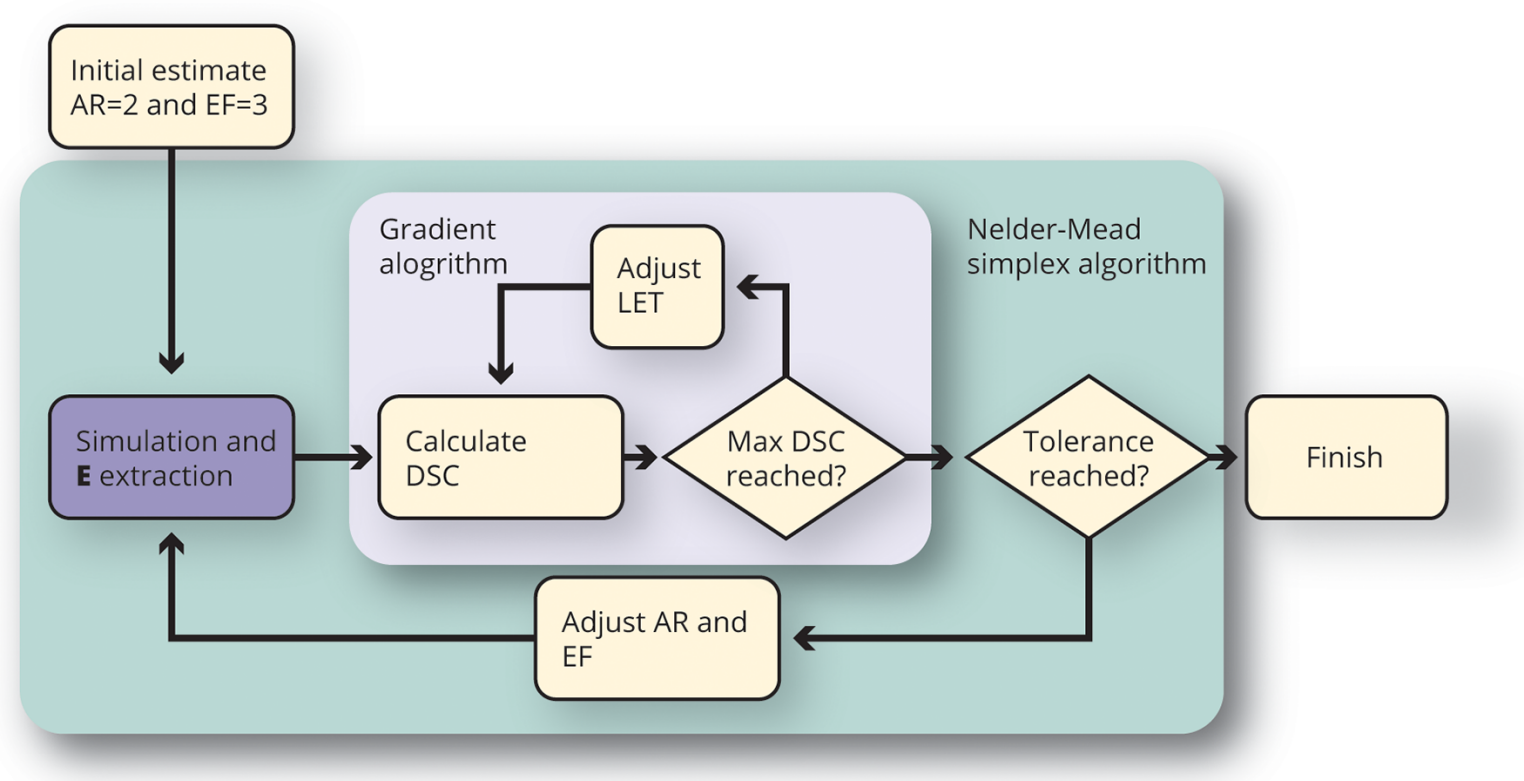
Illustration of the optimization algorithm to find the best matching set of anisotropy ratio (AR), electroporation conductivity increase factor (EF), and lethal electroporation threshold (LET) parameters for each lesion. The simulation and E extraction is the only computationally intensive part of the process, and from the simulated E field, the best fitting threshold can be found without rerunning the simulation.

The similarities between the numerical model and the experimental lesions were obtained using the Dice-Sørensen coefficient. Each experimental lesion was numerically reconstructed in an individual optimization loop, as shown in Figure 2. The measured voltage, and fiber orientation was used as an input to the model. The Nelder-Mead simplex gradient-free optimization algorithm (*fminsearch* in Matlab) was used to find the values of the AR and EF parameter, that best described the lesion shape. Because the output of each simulation was the electric field strength, the best matching LET for each set of AR and EF parameters could be quickly found using a gradient-based optimization algorithm (*fmincon* in Matlab). The final output of each simulation therefore consisted of three quantities (AR, EF, and LET).

### 2.7. Statistical analyses

Statistical analyses of the aspect ratio (AR), electroporation conductivity increase factor (EF), and lethal electroporation threshold (LET) were performed using Matlab Statistics Toolbox. The Kolmogorov-Smirnoff test was used to test for normality. If the data were normally distributed, the two-sample Student’s *t*-test was used to compare the means; otherwise, the Wilcoxson rank sum test was used. The Kruskal-Wallis test was used to check the effects of different experimental conditions, such as the voltage level and experimental sample, on the LET values studied.

## 3. Results

### 3.1. Porcine lesions

**Figure 3:**
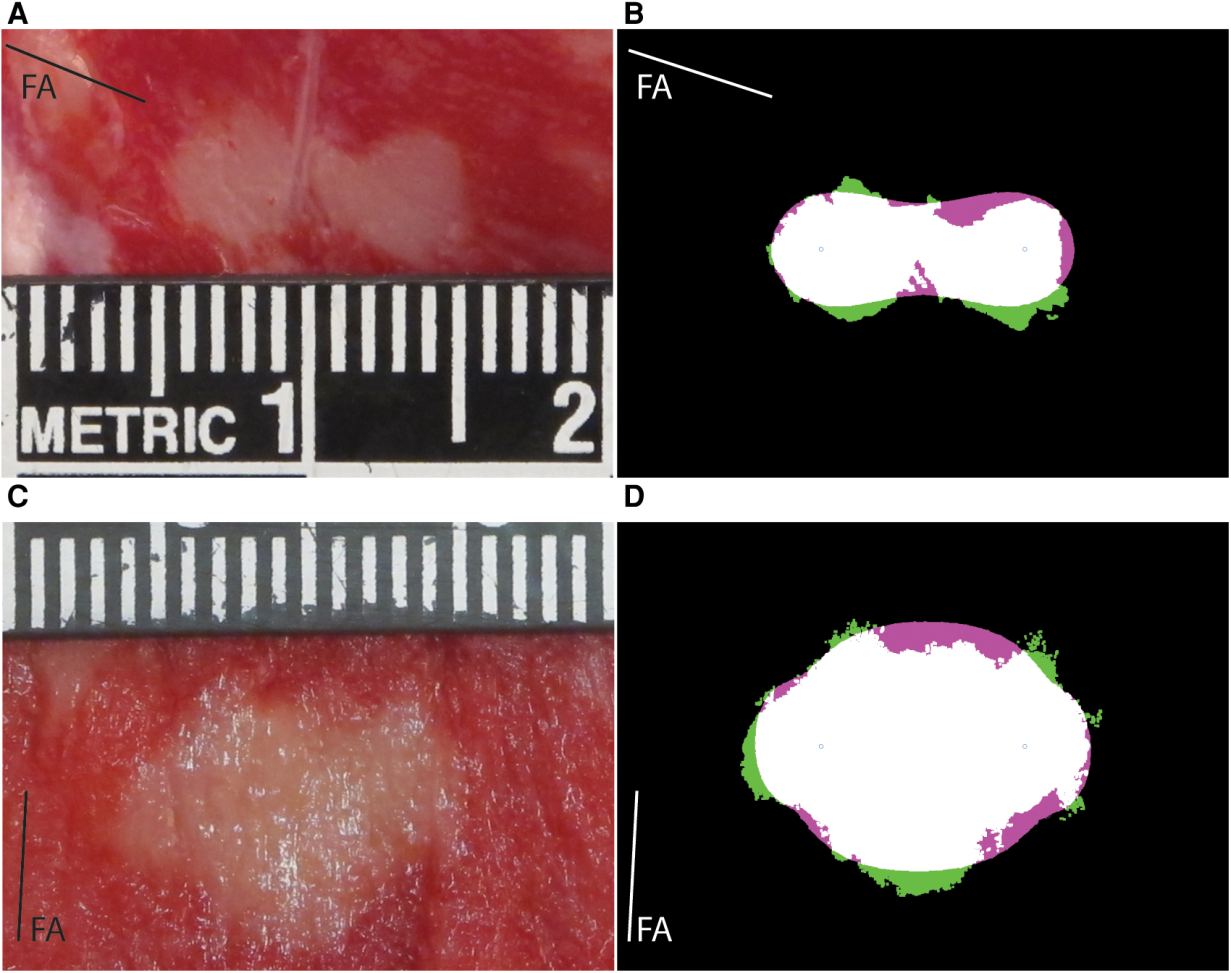
Example results for lesions obtained with MDT protocol with different fiber orientation from porcine subjects. FA labeled line shows the angle of the fibers in each panel. (**A**) Lesion photograph, 700 V, parallel orientation of fibers (−18 degrees). (**B**) Comparison of the lesion and model. The experimental lesion is shown in green, the model output is shown in magenta, while the overlap is white. Best fitting parameters: LET = 511 V/cm, AR = 1.0, EF = 1.88. (**C**) Lesion photograph, 1,200 V, perpendicular fiber orientation (87 degrees). (**D**) Comparison of lesion and model. Best fitting parameters: LET = 543 V/cm, AR = 2.15, EF = 3.18.

**Figure 4:**
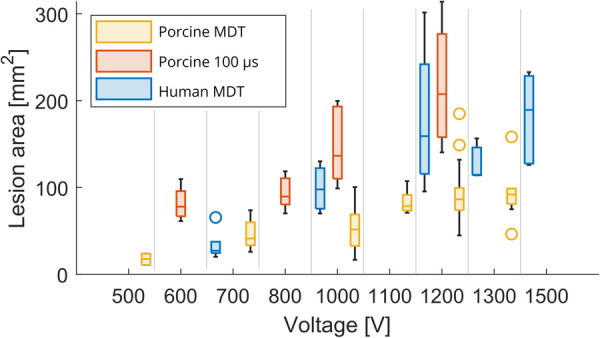
Lesion area as a function of voltage. Box plot shows median and inter-quartile range (IQR). The whiskers indicate the range of the data. Outliers are denoted with a circle symbol and are defined as data, which are more than 1.5⋅IQR away from the box.

A total of 51 lesions were generated with MDT pulses from 6 swine hearts (median 8 per heart, range: 6–13). The experimental lesions were well demarcated by TTC staining (Figures 3A,C). There was an expected trend of increasing lesion size with increasing voltage as seen in Figure 4. At 500 V amplitude application all lesions (total of 5) were disconnected, meaning that there was a distinct lesion around each of the two electrode locations without connection between the two dead areas (see Supplementary Material, e.g., pages 12, 14, 20, 22, 25). At 700 V, 3/6 created lesions were disconnected, while at 1,000 V, only 1/9 lesions was disconnected. At 1,100 to 1,300 V, all resultant lesions were connected, i.e., contiguous. The lesions were differently shaped depending on the orientation of the fibers relative to the electrode positions. Of interest, the lesions where the fibers were near to parallel with the line connecting the needles were figure 8 shaped with a narrower region in between the electrodes. In contrast, the resultant lesions where the fibers were near to perpendicular to the connecting line between the electrodes tended to be wider in the middle. Importantly, this observation indicates that anisotropy in the tissue has an influence on the formed lesion shapes and sizes.

**Figure 5:**
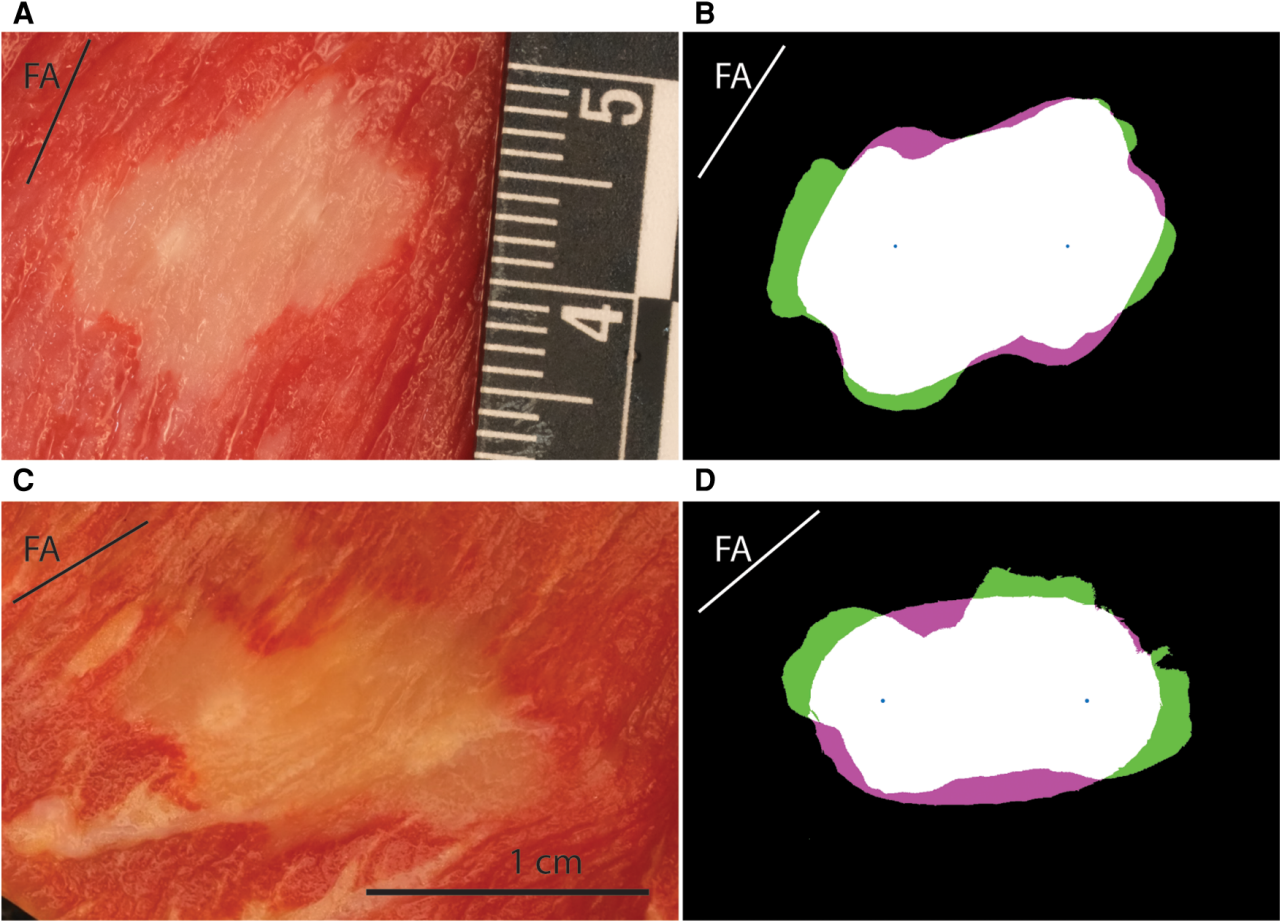
Example lesions for the porcine 100 μs and human MDT experiment. FA labeled line shows the angle of the fibers in each panel. (**A**) Lesion photograph of a porcine 100 μs lesion, 1,200 V, perpendicular orientation of fibers (57 degrees). (**B**) Comparison of the lesion and model. The experimental lesion is shown in green, the model output is shown in magenta, while the overlap is white. Best fitting parameters: LET = 321 V/cm, AR = 4.85, EF = 1.09. (**C**) Lesion photograph of a human lesion, 1,200 V, parallel orientation of fibers (40 degrees). (**D**) Comparison of the lesion and model. Best fitting parameters: LET = 502 V/cm, AR = 1.57, EF = 1.76.

A total of 31 lesions were generated with monophasic 100 μs pulses [median 3 per heart, range (2–6)]. The resultant lesions were visibly larger than those obtained by MDT pulses. All lesions were connected, even at the lowest voltage levels. Similar to the MDT pulses, anisotropies were present in the lesion shapes relative to the direction of the fibers (Figure 5A).

### 3.2. Human lesions

A total of 21 lesions were generated with the MDT pulses in 3 human hearts [median 8 per heart, range (4–9)]. All 3 resultant lesions at 700 V applications were disconnected. The remaining lesions at 1,000, 1,200, 1,300, and 1,500 V applications were all connected. Similar to the swine hearts, there was evidence of anisotropic tissue behavior, also in these human hearts the perpendicular lesions were wider in the area between the electrodes. A representative image from a human heart experiment is shown in Figure 5C.

### 3.3. Numerical results—optimization of threshold values, anisotropy ratios, and electroporation tissue conductivity increase factor

#### 3.3.1. Agreement between numerical results and experimental data

A total of 103 lesions were reconstructed numerically. Good agreement was obtained between the numerical results and the experimental data. Mean values of the Dice-Sørensen coefficient were 0.86, 0.85, and 0.89 for the porcine MDT, human MDT, and porcine 100 μs pulses, respectively. Figure 3 shows two examples of experimental lesions using the MDT pulse parameters applied in porcine hearts: one perpendicular and the other parallel to the fibers. These lesions were selected as representative examples because their LETs were close to the mean values, and the actual fiber orientations were very close to 90 and 0 degrees. Figure 5 shows examples of 100 μs porcine lesion and a human lesion. The two examples are chosen because their voltage levels are the same and fiber orientations are very similar.

#### 3.3.2. Lethal electric field threshold (LET) results

**Figure 6:**
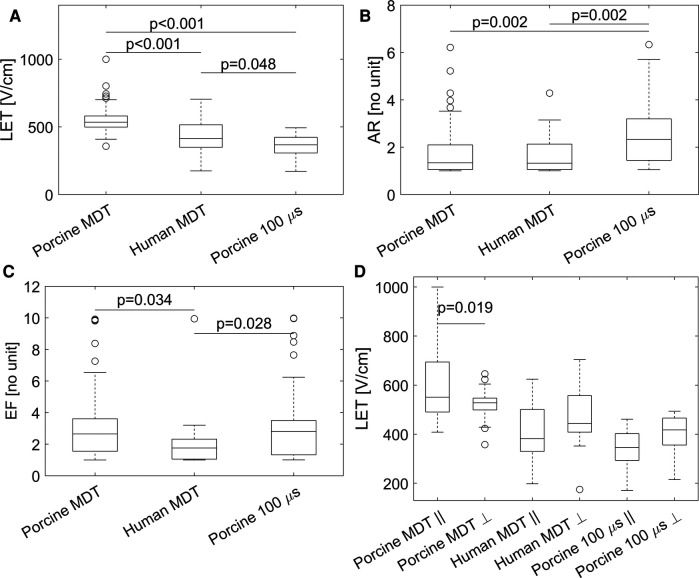
Graphical representation of the results. (**A**) Lethal electric field threshold (LET) in the three different experiments. (**B**) Aspect ratio (AR) in the three different experiments. (**C**) Electroporation conductivity increase factor (EF). (**D**) LET for fiber angle >45° (indicated by ⊥), and angle ≤45° (indicated by ∥). In a comparison with a 2-sample *t*-test the values for porcine MDT experiment are significantly different (*p* = 0.033), but there is no difference in the other experiments.

The descriptive statistics of the three main parameters are given in Table 2 and the main outcomes are graphically represented in Figure 6. Since the distributions of LET, AR, and EF parameters in porcine MDT experiments was not normal (K-S test *p*-value: 0.0397) and the other experiments have a smaller number of samples, we used the Wilcoxson rank sum test in all cases to compare the distributions. These tests showed statistically significant differences in LET between the porcine MDT and 100 μs experiment (*p* < 0.001), between the porcine and human MDT experiment (*p* < 0.001, Figure 6A), and between the human MDT and porcine 100 μs experiment (*p* = 0.048). Figure 6D shows LET when separated into parallel and perpendicular lesions. In a comparison with a 2-sample *t*-test the values for porcine MDT experiment are significantly different (*p* = 0.033), but there is no difference in the other experiments.

**Table 2:** Descriptive statistics of the main parameters obtained in the numerical simulations.

	Porcine MDT	Human MDT	Porcine 100 us
Sample size	51	21	31
Lethal electric field threshold (LET)			
Mean [V/cm]	551	430	363
Median [V/cm]	535	416	368
Standard deviation [V/cm]	110	135	84
*p* value MDT vs. 100 us	**<0** **.** **001**		
*p* value MDT porcine vs. human	**<0** **.** **001**		
*p* value 100 us porcine vs. MDT human	**0** **.** **048**		
Aspect ratio (AR)			
Mean [no unit]	1.86	1.73	2.70
Median [no unit]	1.34	1.33	2.33
Standard deviation [no unit]	1.19	1.02	1.47
*p* value MDT vs. 100 us	**0** **.** **002**		
*p* value MDT porcine vs. human	0.673		
*p* value 100 us porcine vs. MDT human	**0** **.** **002**		
Electroporation conductivity increase factor (EF)			
Mean [no unit]	3.18	2.19	3.48
Median [no unit]	2.65	1.76	2.80
Standard deviation [no unit]	2.31	2.73	1.94
*p* value MDT vs. 100 us	0.796		
*p* value MDT porcine vs. human	**0** **.** **028**		
*p* value 100 us porcine vs. MDT human	**0** **.** **034**		

Bold values are considered statistically significant at *p* < 0.05.

#### 3.3.3. Results of anisotropy ratio (AR) and electroporation conductivity increase factor (EF) optimization

**Figure 7:**
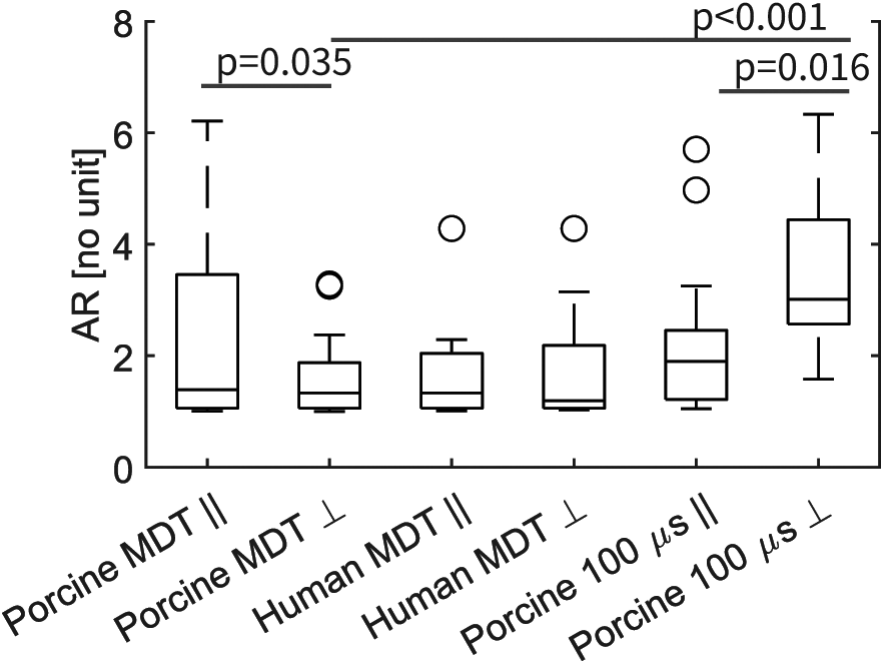
Graphical representation of the AR for fiber angle >45° (indicated by ⊥), and angle ≤45° (indicated by ∥). In a comparison with a 2-sample *t*-test the values for porcine MDT experiment are significantly different (*p* = 0.035), as are the mean values for the 100 μs experiment. In comparing the MDT and 100 μs pulses in the procine experiment, there is a significant difference in the value of AR in lesions perpendicular to the fibers.

The median values of AR were not significantly different between the two experiments with MDT pulses; i.e., between porcine and human heart (Figure 6B). But the value was significantly larger in the 100 μs porcine experiment, than in the other two experiments, (*p* = 0.002 in both cases). When the AR parameter was separated into parallel and perpendicular lesions (Figure 7), there were significant differences between the parallel and perpendicular values in the porcine MDT, and in the porcine 100 μs experiments. The values of AR in perpendicular lesions in the porcine MDT experiment were lower (perpendicular mean = 1.54) than in the parallel lesions (parallel mean = 2.24). The opposite was true for the porcine 100 μs lesions (perpendicular mean = 3.42; parallel mean = 2.17).

Median values for the parameter EF (Figure 6C) were significantly lower in the human experiments (*p* = 0.028 vs. porcine MDT, and *p* = 0.034 vs. 100 μs porcine). When separated in perpendicular and parallel orientations, there were no differences between the groups from the same experiment, i.e., the values of EF were not significantly different between parallel and perpendicular orientations.

#### 3.3.4. Reproducibility and experimental integrity checks

To check experimental integrities, we also tested the influences of experimental patterns and voltage levels on resultant LET. The null hypothesis was that the voltage levels had no effect on the determined LET and that the determined thresholds were ideally independent of the subject. The Kruskal-Wallis test was used to test the influence of voltage on the LET. None of the tests showed a significant influence of voltage (*p*-values: 0.59 porcine MDT; 0.12 porcine 100 μs; 0.20 human; Supplementary Figures S1–S3). Within-sample variability was also tested using the Kruskal-Wallis test. LET in MDT porcine results showed a significant difference (*p* = 0.029), while the porcine 100 μs and human experiment showed no differences (*p*-values: 0.42 porcine 100 μs; 0.89 human). *Post-hoc* analysis showed that the median value of LET in experimental animal 6 was significantly higher than in animal 3, but there were no other differences (Supplementary Figure S4). Despite that, we used all experimental points in evaluating the descriptive statistics and comparisons of experiments to better reflect the experimental variability.

### 3.4. Literature review on *in vivo* threshold determination across different tissues

Lethal electric field thresholds (LET) are usually determined by comparing the size/volume of the resultant lesions in the follow up imaging or histology, with the distributions of the electric field calculated with numerical models. Table 3 provides an overview of *in vivo* studies in which LET was determined for various tissues/organs. We focused exclusively on studies where *in vivo* experiments were performed using protocols for classic (using monophasic pulses with 50–100 µs duration) irreversible electroporation. In the reviewed studies, the employed protocols used a range of 8–100 pulses, with 50–100 µs duration, 1–4 Hz repetition rate and needle electrodes. Considering the differences in investigated pulse parameters it is not surprising that the LET values reported for the same tissue differ from study to study. The format of the reported data also varied from study to study, e.g., some studies reported on the minimum and maximum value, while some reported the mean and standard deviation. Therefore, we compiled (when available) the mean, minimum, and maximum values of LET from the studies and calculated the mean, standard deviation, and range across all studies for a given tissue. In most studies, the mean LET was found to be 500–700 V/cm, except for bone tissue, where the mean LET is much higher (1,050 V/cm); possibly due to the low conductivity of the mineralized parts of the bone tissue. Another exception was a recent study on skeletal muscle tissue, in which the exact same pulsing protocol was employed as in our present study (54). The LET of skeletal muscle as determined in that aforementioned study was much lower than in any other tissue reported previously including the heart tissue in our own study. Because the other four studies on muscle tissue all used 8 pulses, we are reporting them separately in Table 3. We found only one study in which LET was determined *in vivo* for heart tissue (55). The threshold value reported there was 640 V/cm, which is significantly higher than the 363 V/cm reported in our study. However, the number of pulses applied in our study (48 pulses) was much higher than used by García-Sanchez et al. (55) (10 pulses), which explains at least part of the difference in the LET values obtained.

**Table 3:** Overview of lethal electric field threshold values (LET), reported in *in vivo* studies for various tissues.

Tissue type	Mean (V/cm)	Std (V/cm)	Min (V/cm)	Max (V/cm)	Pulse number	Pulse length (µs)	No. of studies	Outcome evaluation (no. of studies)	Time of evaluation (no. of studies)	Animal model	References
Bone	1,050	212	900	1,310	90, 120	70, 100	2	Histology (2)	7 days	Pig, rabbit	(38, 81)
Brain	585	145	495	875	50–90	50–90	3	Gd MRI (3)	1 h (2), 3 weeks (1)	Dog, rat	(81–83)
Kidney	538	53	500	642	70–100	70–100	2	Histology (1), gross pathology & CT (1)	6 h, 24 h	Pig, dog	(39, 84)
Liver	539	158	309	680	8–90	100	5	Gross pathology (1), histology (4)	24 h (1), 72 h (4)	Pig, dog, rabbit	(42, 76, 85, 86)
Prostate	700	262	440	1,191	70–100	70–100	4	Gd MRI (3), gross pathology/histology (1)	6 h (1), 1 week (1), 3–4 weeks (3)	Human, dog	(40, 87–89)
Muscle	570	171	430	800	8	100	4	Cr-EDTA uptake (3), Gd-DOTA uptake (1)	1 h (1), 1 day (2), 3 days (1, Gd)	Mouse, rat	(41, 75, 90, 91)
Muscle	193	84	130	291	48	100	1	TTC stain (gross pathology)	1–1.5 h	Pig	(54)
Heart	640	/	/	/	10	100	1	Histology	21 days	Rat	(55)
*This study*	363	84	171	493	48	100	1	TTC stain (gross pathology)	1–1.5 h	Pig	

## 4. Discussion

In the present study on isolated human and swine hearts, we determined the values of LET using a method which enables the simultaneous determination of lethal electric field threshold (LET), anisotropy ratio (AR) and electroporation conductivity increase factor (EF). We used *in vitro* experimental setups and pulsing protocols consistent with previously published literature (42, 54, 85). The median value of 380 V/cm obtained in the 100 μs porcine experiment was lower than the results reported for all tissues included in our current literature review (Table 3), even when compared to protocols with a larger number of pulses. We consider this a more solid evidence for lower thresholds of cardiac tissues compared to other tissues studied so far with *in vivo* experiments, with the notable exception of the skeletal muscle reported recently (54). However, the evidence for lower LET of cardiac tissue due to short biphasic pulses is still limited, and is potentially more complex due to the large diversity of pulse parameters reported in such studies (14, 76, 92, 93).

Noteworthy was the fact that the LET using the MDT protocol was higher than LET using the 100 μs-pulse protocol in healthy swine hearts. We consider this to be the result of the fact that the MDT protocol uses biphasic pulses of significantly shorter durations as well as shorter total ON time of pulses. The use of short bipolar pulses has previously been shown to increase LET *in vitro* (43, 94) and *in vivo* (76, 92, 95). A similar result with biphasic pulses was found in rat hearts (55), where the LET increased dramatically with the increase in pulse frequency. The finding that human hearts exhibited lower LET than porcine hearts may be attributed, at least in part, to the fact that these donated hearts were not eligible for transplantation for various reasons: i.e., they had a medical history that may have led to cardiac dysfunction (i.e., alcohol use, hypertension, and diabetes), and were therefore in poorer conditions than the young healthy porcine hearts. Furthermore, the human hearts were also not optimally procured for the uses in these experiments (i.e., longer times between explantations and starts of experiments). It is suspected that longer ischemic times could render cardiac cells more susceptible to injury; including injury following PFA applications, via a variety of mechanisms including less efficient ion channel exchanges or elevated pre-ablation calcium levels (96, 98). In addition, statistically significant differences between the EF for the porcine and human MDT experiments may be due to the fact that only 1/3 of the human hearts used the Modified Langendorff setup with filled chambers and restarted mechanical activity; although we did not detect a significant effect of the donor heart on the LET in these human hearts (Supplementary Figure S5).

The anisotropy present in the structure of the large mammalian heart plays an important role in its function and also affects the propagation of its electrical signals. The present results show that anisotropy also influences the shape of the elicited lesions. The parameter AR determines how much the lesion shapes are “stretched” in the directions of the fibers. In the present results, in the porcine experiments with 100 μs pulse width, we observed a significantly larger AR than the experiments with MDT pulses. Interestingly, when the data were separated into parallel and perpendicular groups (Figure 7), the differences were even more striking. The mean values of the AR in perpendicular lesions were lower than in parallel lesions for MDT pulses; on the other hand, the AR was higher in perpendicular lesions with the 100 μs pulses. This could be due to the differing effects that the pulses of different durations have on elongated cells (78, 99). The results suggest that MDT pulse protocol, with its shorter pulse duration and biphasic pulses, resulted in lesions that were less dependent on fiber orientations than the treatment with longer pulse duration.

It is important to also note, that the choice of the numerical model of electroporation, namely the increase of conductivity due to electroporation can affect the determined values of the LET. For example, Miklavčič et al. (85) used a model with constant conductivity, while the reanalysis of the same experimental dataset with a field dependent conductivity (42) yielded a higher threshold, i.e., 637 vs. 700 V/cm, respectively. In the reviewed literature (Table 3) approximately half the studies used constant electric conductivity, and for the same tissue, in the studies with constant conductivities they reported lower LET, consistent with the observation above. The exact choice of the function for the increase in conductivity seems however to be less important, although some sort of sigmoid function is most often employed; Corovic et al. (68) explored several different functions and found the best match between the numerical model and experiment using a smoothed Heaviside function, also used in our current study. Although, the study by García-Sanchez et al. (55) used a different formulation for the sigmoid function, the shape and location of the transition function match ours very closely. Again, in our optimization the function with the transition region was always the same, but the EF parameter (factor of maximum increase of conductivity relative to the initial unperturbed value) was varied to find the value which resulted in a lesion shape that best matched the obtained experimental lesion. The resulting median values of the EF parameter of approximately 3, are very similar to values reported in the literature (68, 76, 97). The main effect is the increase of conductivity near the electrodes where the electric fields are the strongest. This results in stronger electric fields further away from the electrodes in comparison to a model with constant electric conductivity, and in our study affects the widths of the numerically obtained lesions. Because the spread of the EF values in our study was relatively high (Figure 6C), we also checked if the values of the EF parameters influenced the LET values of the corresponding lesions. While there was a statistically significant negative correlation of LET with EF (*k* = −13, *p* = 0.037, Supplementary Figure S7), the *R*^2^ value was small (0.08), and the correlation also accounted for a change in LET smaller than one standard deviation.

There are several potential limitations of this study, first we need to consider the experimental variability, the employment of an idealized model, and also a small number of human hearts. The experimental variability in our current study can be explained in part by both the variability in the slicing of the heart tissues and locating the exact centers of the electrodes during the experiments. The employed numerical model was always the same and idealized, i.e., the local thicknesses of the myocardium and the roles of internal structures, e.g., papillary muscles, were not considered. Further it should be noted that the electrodes used in these *in vitro* studies are different from those used in the clinic, but there are several advantages to the used electrodes. The electrodes’ fixed geometry resulted in repeatable and constant distance between the electrodes, their penetrating nature ensured independence of contact pressure, and the distance of the electrodes also resulted in electric field gradient (i.e., the change in electric field over distance), which allowed the model to be compared to the experiments with reasonable sensitivity at clinically relevant voltage levels. This means, that with a different setup, a pixel of difference at the lesion edges might represent a higher change in the electric field strength, which would result in larger variability of the estimated LET. While the experimental setup ensured good robustness of the estimated LET (and other parameters), this means that lesion sizes cannot be used directly to predict lesion sizes which would result with the uses of clinical devices. Overall, the numerical model developed is the most important result of this study. Using the determined parameters reported here with device-specific geometries should be able to successfully predict lesion sizes with different devices, as shown in a previous study (25). Note, the small number of human hearts included in the study was due to the fact that they are not readily available.

## 5. Conclusion

We determined lethal electric field thresholds (LET) in *ex vivo* porcine heart and human heart studies for different applied PFA: i.e., proprietary biphasic waveform (Medtronic) and 100 μs pulses. Interestingly, the LET values we determined for this limited number (*n* = 3) of human hearts were not higher than those of healthy porcine hearts. These findings, albeit preliminary, from a limited number of hearts suggest that treatments in humans with parameters optimized in pigs should result in equal or greater lesions. We believe that the obtained LET values can be used in defining safe and effective PFA protocols for future cardiac applications. Given the highly variable anisotropy in the heart and the varying thickness of the myocardium, ablations performed with Medtronic’s proprietary waveforms were found to be less affected by anisotropy than 100 µs pulses, resulting in more predictable lesions. Overall, the results obtained showed that *ex vivo* swine and human hearts had lower LETs than other tissues reported in the literature, with the exception of skeletal muscle. Our ability to accurately model the experimental *in vitro* results should help in the clinical application of these findings.

## Data Availability

Datasets presented in this study are available in Supplementary Material.

## References

[1] Reddy VY Neuzil P Koruth JS Petru J Funosako M Cochet H Pulsed field ablation for pulmonary vein isolation in atrial fibrillation. J Am Coll Cardiol. (2019) 74:315–26. 10.1016/j.jacc.2019.04.021 31085321

[2] Stewart MT Haines DE Verma A Kirchhof N Barka N Grassl E Intracardiac pulsed field ablation: proof of feasibility in a chronic porcine model. Heart Rhythm. (2019) 16:754–64. 10.1016/j.hrthm.2018.10.030 30385383

[3] Reddy VY Anter E Rackauskas G Peichl P Koruth JS Petru J Lattice-tip focal ablation catheter that toggles between radiofrequency and pulsed field energy to treat atrial fibrillation: a first-in-human trial. Circ Arrhythm Electrophysiol. (2020) 13:e008718. 10.1161/CIRCEP.120.008718 32383391

[4] Davalos R Mir L Rubinsky B. Tissue ablation with irreversible electroporation. Ann Biomed Eng. (2005) 33:223–31. 10.1007/s10439-005-8981-8 15771276

[5] Yarmush ML Golberg A Serša G Kotnik T Miklavčič D. Electroporation-based technologies for medicine: principles, applications, and challenges. Annu Rev Biomed Eng. (2014) 16:295–320. 10.1146/annurev-bioeng-071813-104622 24905876

[6] Kotnik T Rems L Tarek M Miklavčič D. Membrane electroporation and electropermeabilization: mechanisms and models. Annu Rev Biophys (2019) 48: 63–91. 10.1146/annurev-biophys-052118-115451 30786231

[7] Gallagher JJ Svenson RH Svenson RH Kasell JH German LD Bardy GH Catheter technique for closed-chest ablation of the atrioventricular conduction system. Am J Cardiol. (1982) 306:194–200. 10.1016/0002-9149(82)92457-2 7054682

[8] Gallagher JJ Svenson RH Kasell JH German LD Bardy GH Broughton A Critelli G Catheter technique for closed chest ablation of the atrioventricular conduction system a therapeutic alternative for the treatment of refractory supraventricular tachycardia. N Engl J Med (1982) 10.1056/nejm198201283060402 7054682

[9] Neunlist M Tung L. Dose-dependent reduction of cardiac transmembrane potential by high-intensity electrical shocks. Am J Physiol-Heart Circ Physiol. (1997) 273:H2817–25. 10.1152/ajpheart.1997.273.6.H2817 9435619

[10] Nikolski VP Efimov IR. Electroporation of the heart. EP Eur. (2005) 7:S146–54. 10.1016/j.eupc.2005.04.011 16102512

[11] Lavee J Onik G Mikus P Rubinsky B. A novel nonthermal energy source for surgical epicardial atrial ablation: irreversible electroporation. Heart Surg Forum. (2007) 10:E162–167. 10.1532/HSF98.20061202 17597044

[12] Wittkampf FH Driel VJV Wessel HV Vink A Hof IE Gründeman PF Feasibility of electroporation for the creation of pulmonary vein ostial lesions. J Cardiovasc Electrophysiol. (2011) 22:302–9. 10.1111/j.1540-8167.2010.01863.x 20653809

[13] Neven K van Driel V van Wessel H van Es R Doevendans PA Wittkampf F. Epicardial linear electroporation ablation and lesion size. Heart Rhythm. (2014) 11:1465–70. 10.1016/j.hrthm.2014.04.031 24768609

[14] van Es R Konings MK Du Pré BC Neven K van Wessel H van Driel VJHM High-frequency irreversible electroporation for cardiac ablation using an asymmetrical waveform. Biomed Eng OnLine. (2019) 18:75. 10.1186/s12938-019-0693-7 31221146 PMC6585075

[15] Witt CM Sugrue A Padmanabhan D Vaidya V Gruba S Rohl J Intrapulmonary vein ablation without stenosis: a novel balloon-based direct current electroporation approach. J Am Heart Assoc. (2018) 7:1–8. 10.1161/JAHA.118.009575 PMC6064854 29987121

[16] Yavin H Brem E Zilberman I Shapira-Daniels A Datta K Govari A Circular multielectrode pulsed field ablation catheter lasso pulsed field ablation: lesion characteristics, durability, and effect on neighboring structures. Circ Arrhythm Electrophysiol. (2021) 14:157–65. 10.1161/CIRCEP.120.009229 PMC7909749 33417475

[17] Zhu T Wang Z Wang S Shi T Zhu X Ma K Pulsed field ablation of superior vena cava: feasibility and safety of pulsed field ablation. Front Cardiovasc Med. (2021) 8:698716. 10.3389/fcvm.2021.698716 34434976 PMC8382124

[18] Livia C Sugrue A Witt T Polkinghorne MD Maor E Kapa S Elimination of purkinje fibers by electroporation reduces ventricular fibrillation vulnerability. J Am Heart Assoc. (2018) 7:e009070. 10.1161/JAHA.118.009070 30371233 PMC6201470

[19] Yavin HD Higuchi K Sroubek J Younis A Zilberman I Anter E. Pulsed-field ablation in ventricular myocardium using a focal catheter: the impact of application repetition on lesion dimensions. Circ Arrhythm Electrophysiol. (2021) 14:819–28. 10.1161/CIRCEP.121.010375 34459210

[20] Im SI Higuchi S Lee A Stillson C Buck E Morrow B Pulsed field ablation of left ventricular myocardium in a swine infarct model. JACC Clin Electrophysiol. (2022) 8:722–31. 10.1016/j.jacep.2022.03.007 35738848

[21] Grimaldi M Di Monaco A Gomez T Berman D Datta K Sharma T Time course of irreversible electroporation lesion development through short- and long-term follow-up in pulsed-field ablation–treated hearts. Circ Arrhythm Electrophysiol. (2022) 15:435–42. 10.1161/CIRCEP.121.010661 35763432

[22] Ekanem E Reddy VY Schmidt B Reichlin T Neven K Metzner A Multi-national survey on the methods, efficacy, and safety on the post-approval clinical use of pulsed field ablation (MANIFEST-PF). EP Eur. (2022) 24:1256–66. 10.1093/europace/euac050 PMC9435639 35647644

[23] Verma A Haines DE Boersma LV Sood N Natale A Marchlinski FE Pulsed field ablation for the treatment of atrial fibrillation: PULSED AF pivotal trial. Circulation. (2023) 147(19):1422–32. 10.1161/CIRCULATIONAHA.123.063988 36877118 PMC10158608

[24] Reddy VY Peichl P Anter E Rackauskas G Petru J Funasako M A focal ablation catheter toggling between radiofrequency and pulsed field energy to treat atrial fibrillation. JACC Clin Electrophysiol. (2023) (in press). 10.1016/j.jacep.2023.04.002 37227340

[25] Howard B Verma A Tzou WS Mattison L Kos B Miklavčič D Effects of electrode-tissue proximity on cardiac lesion formation using pulsed field ablation. Circ Arrhythm Electrophysiol. (2022) 15:706–13. 10.1161/CIRCEP.122.011110 PMC9584049 36166690

[26] Mattison L Verma A Tarakji KG Reichlin T Hindricks G Sack KL Effect of contact force on pulsed field ablation lesions in porcine cardiac tissue. J Cardiovasc Electrophysiol. (2023) 34(3):693–9. 10.1111/jce.15813 36640426

[27] Neven K van Es R van Driel V van Wessel H Fidder H Vink A Acute and long-term effects of full-power electroporation ablation directly on the porcine esophagus. Circ Arrhythm Electrophysiol. (2017) 10:e004672. 10.1161/CIRCEP.116.004672 28487347

[28] van Driel VJHM Neven K van Wessel H Vink A Doevendans PAFM Wittkampf FHM. Low vulnerability of the right phrenic nerve to electroporation ablation. Heart Rhythm. (2015) 12:1838–44. 10.1016/j.hrthm.2015.05.012 25998897

[29] Howard B Haines DE Verma A Kirchhof N Barka N Onal B Characterization of phrenic nerve response to pulsed field ablation. Circ Arrhythm Electrophysiol. (2022) 15:393–401. 10.1161/CIRCEP.121.010127 PMC9213085 35649121

[30] Howard B Haines DE Verma A Packer D Kirchhof N Barka N Reduction in pulmonary vein stenosis and collateral damage with pulsed field ablation compared with radiofrequency ablation in a canine model. Circ Arrhythm Electrophysiol. (2020) 13:e008337. 10.1161/CIRCEP.120.008337 32877256 PMC7495982

[31] Belalcazar A. Safety and efficacy aspects of pulsed field ablation catheters as a function of electrode proximity to blood and energy delivery method. Heart Rhythm O2. (2021) 2:560–9. 10.1016/j.hroo.2021.10.004 34988500 PMC8703144

[32] Mahnič-Kalamiza S Miklavčič D. Scratching the electrode surface: insights into a high-voltage pulsed-field application from in vitro & in silico studies in indifferent fluid. Electrochim Acta. (2020) 363:137187. 10.1016/j.electacta.2020.137187

[33] Reddy VY Dukkipati SR Neuzil P Anic A Petru J Funasako M Pulsed field ablation of paroxysmal atrial fibrillation. JACC Clin Electrophysiol. (2021) 7:614–27. 10.1016/j.jacep.2021.02.014 33933412

[34] Verma A Boersma L Haines DE Natale A Marchlinski FE Sanders P First-in-human experience and acute procedural outcomes using a novel pulsed field ablation system: the PULSED AF pilot trial. Circ Arrhythm Electrophysiol. (2022) 15:e010168. 10.1161/CIRCEP.121.010168 34964367 PMC8772438

[35] Reddy VY Anic A Koruth J Petru J Funasako M Minami K Pulsed field ablation in patients with persistent atrial fibrillation. J Am Coll Cardiol. (2020) 76:1068–80. 10.1016/j.jacc.2020.07.007 32854842

[36] Loh P van Es R Groen MHA Neven K Kassenberg W Wittkampf FHM Pulmonary vein isolation with single pulse irreversible electroporation: a first in human study in 10 patients with atrial fibrillation. Circ Arrhythm Electrophysiol. (2020) 13:e008192. 10.1161/CIRCEP.119.008192 32898450

[37] Kotnik T Kramar P Pucihar G Miklavcic D Tarek M. Cell membrane electroporation-part 1: the phenomenon. IEEE Electr Insul Mag. (2012) 28:14–23. 10.1109/MEI.2012.6268438

[38] Tam AL Abdelsalam ME Gagea M Ensor JE Moussa M Ahmed M Irreversible electroporation of the lumbar vertebrae in a porcine model: is there clinical-pathologic evidence of neural toxicity? Radiology. (2014) 272:709–19. 10.1148/radiol.14132560 24766034

[39] Neal RE Garcia PA Kavnoudias H Rosenfeldt F Mclean CA Earl V In vivo irreversible electroporation kidney ablation: experimentally correlated numerical models. IEEE Trans Biomed Eng. (2015) 62:561–9. 10.1109/TBME.2014.2360374 25265626

[40] Scheltema MJ O’Brien TJ van den Bos W de Bruin DM Davalos RV van den Geld CWM Numerical simulation modeling of the irreversible electroporation treatment zone for focal therapy of prostate cancer, correlation with whole-mount pathology and T2-weighted MRI sequences. Ther Adv Urol. (2019) 11:1756287219852305. 10.1177/1756287219852305 31217820 PMC6557022

[41] Čorović S Županič A Kranjc S Al Sakere B Leroy-Willig A Mir LM The influence of skeletal muscle anisotropy on electroporation: in vivo study and numerical modeling. Med Biol Eng Comput. (2010) 48:637–48. 10.1007/s11517-010-0614-1 20424926 PMC2886894

[42] Sel D Cukjati D Batiuskaite D Slivnik T Mir LM Miklavcic D. Sequential finite element model of tissue electropermeabilization. IEEE Trans Biomed Eng. (2005) 52:816–27. 10.1109/TBME.2005.845212 15887531

[43] Mercadal B Beitel-White N Aycock KN Castellví Q Davalos RV Ivorra A. Dynamics of cell death after conventional IRE and H-FIRE treatments. Ann Biomed Eng. (2020) 48:1451–62. 10.1007/s10439-020-02462-8 32026232 PMC7154019

[44] Napotnik TB Polajžer T Miklavčič D. Cell death due to electroporation—a review. Bioelectrochemistry. (2021) 141:107871. 10.1016/j.bioelechem.2021.107871 34147013

[45] Kaminska I Kotulska M Stecka A Saczko J Drag-Zalesinska M Wysocka T Electroporation-induced changes in normal immature rat myoblasts (H9C2). Gen Physiol Biophys. (2012) 31:19–25. 10.4149/gpb_2012_003 22447827

[46] Reddy VY Koruth J Jais P Petru J Timko F Skalsky I Ablation of atrial fibrillation with pulsed electric fields: an ultra-rapid, tissue-selective modality for cardiac ablation. JACC Clin Electrophysiol. (2018) 4:987–95. 10.1016/j.jacep.2018.04.005 30139499

[47] Hunter DW Kostecki G Fish JM Jensen JA Tandri H. In vitro cell selectivity of reversible and irreversible: electroporation in cardiac tissue. Circ Arrhythm Electrophysiol. (2021) 14:440–8. 10.1161/CIRCEP.120.008817 33729827

[48] Casciola M Keck D Feaster TK Blinova K. Human cardiomyocytes are more susceptible to irreversible electroporation by pulsed electric field than human esophageal cells. Physiol Rep. (2022) 10:e15493. 10.14814/phy2.15493 36301726 PMC9612150

[49] Avazzadeh S Dehkordi MH Owens P Jalali A O’Brien B Coffey K Establishing electroporation thresholds for targeted cell specific cardiac ablation in a 2D culture model. J Cardiovasc Electrophysiol. (2022) 33(9):2050–61. 10.1111/jce.15641 35924470 PMC9543844

[50] Sowa PW Kiełbik AS Pakhomov AG Gudvangen E Mangalanathan U Adams V How to alleviate cardiac injury from electric shocks at the cellular level. Front Cardiovasc Med. (2022) 9:1004024. 10.3389/fcvm.2022.1004024 36620647 PMC9812960

[51] Kotnik T Pucihar G Miklavčič D. “The cell in the electric field”. In: Kee ST Gehl J Lee EW, editors. Clinical aspects of electroporation. New York, NY: Springer New York (2011). p. 19–29.

[52] Dermol J Miklavčič D. Mathematical models describing Chinese hamster ovary cell death due to electroporation in vitro. J Membr Biol. (2015) 248:865–81. 10.1007/s00232-015-9825-6 26223863

[53] Rems L Miklavčič D. Tutorial: electroporation of cells in complex materials and tissue. J Appl Phys. (2016) 119:201101. 10.1063/1.4949264

[54] Smerc R Ramirez DA Mahnic-Kalamiza S Dermol-Cerne J Sigg DC Mattison LM A multiscale computational model of skeletal muscle electroporation validated using *in situ* porcine experiments. IEEE Trans Biomed Eng. (2023) 70(6):1826–37. 10.1109/TBME.2022.3229560 37022450

[55] García-Sánchez T Amorós-Figueras G Jorge E Campos MC Maor E Guerra JM Parametric study of pulsed field ablation with biphasic waveforms in an in vivo heart model: the role of frequency. Circ Arrhythm Electrophysiol. (2022) 15:e010992. 10.1161/CIRCEP.122.010992 36178752

[56] Jiang C Davalos RV Bischof JC. A review of basic to clinical studies of irreversible electroporation therapy. IEEE Trans Biomed Eng. (2015) 62:4–20. 10.1109/TBME.2014.2367543 25389236

[57] Pucihar G Krmelj J Reberšek M Napotnik TB Miklavčič D. Equivalent pulse parameters for electroporation. IEEE Trans Biomed Eng. (2011) 58:3279–88. 10.1109/TBME.2011.2167232 21900067

[58] Perera-Bel E Mercadal B Garcia-Sanchez T Gonzalez Ballester MA Ivorra A. Modeling methods for treatment planning in overlapping electroporation treatments. IEEE Trans Biomed Eng. (2021) 69(4):1318–27. 10.1109/TBME.2021.3115029 34559631

[59] Miklavcic D Beravs K Semrov D Cemazar M Demsar F Sersa G. The importance of electric field distribution for effective in vivo electroporation of tissues. Biophys J. (1998) 74:2152–8. 10.1016/S0006-3495(98)77924-X 9591642 PMC1299558

[60] Chinchoy E Soule CL Houlton AJ Gallagher WJ Hjelle MA Laske TG Isolated four-chamber working swine heart model. Ann Thorac Surg. (2000) 70:1607–14. 10.1016/S0003-4975(00)01977-9 11093495

[61] Hill AJ Coles JA Sigg DC Laske TG Iaizzo PA. Images of the human coronary sinus ostium obtained from isolated working hearts. Ann Thorac Surg (2003) 76:2108. 10.1016/S0003-4975(03)00268-6 14667663

[62] Howard SA Quill JL Eggen MD Swingen CM Iaizzo PA. Novel imaging of atrial septal defects in isolated human hearts. J Cardiovasc Transl Res. (2013) 6:218–20. 10.1007/s12265-013-9451-6 23413124

[63] Miklavcic D Semrov D Mekid H Mir LM. A validated model of in vivo electric field distribution in tissues for electrochemotherapy and for DNA electrotransfer for gene therapy. Biochim Biophys Acta. (2000) 1523:73–83. 10.1016/S0304-4165(00)00101-X 11099860

[64] Stewart MT Haines DE Miklavčič D Kos B Kirchhof N Barka N Safety and chronic lesion characterization of pulsed field ablation in a porcine model. J Cardiovasc Electrophysiol. (2021) 32:958–69. 10.1111/jce.14980 33650743 PMC8048690

[65] Fishbein MC Meerbaum S Rit J Lando U Kanmatsuse K Mercier JC Early phase acute myocardial infarct size quantification: validation of the triphenyl tetrazolium chloride tissue enzyme staining technique. Am Heart J. (1981) 101:593–600. 10.1016/0002-8703(81)90226-X 6164281

[66] Schneider CA Rasband WS Eliceiri KW. NIH image to ImageJ: 25 years of image analysis. Nat Methods. (2012) 9:671–5. 10.1038/nmeth.2089 22930834 PMC5554542

[67] Labarbera N Drapaca C. Anistropically varying conductivity in irreversible electroporation simulations. Theor Biol Med Model. (2017) 14:20. 10.1186/s12976-017-0065-6 29089031 PMC5664922

[68] Corovic S Lackovic I Sustaric P Sustar T Rodic T Miklavcic D. Modeling of electric field distribution in tissues during electroporation. Biomed Eng OnLine. (2013) 12:16. 10.1186/1475-925X-12-16 23433433 PMC3614452

[69] Rush S Abildskov JA Mcfee R. Resistivity of body tissues at low frequencies. Circ Res. (1963) 12:40–50. 10.1161/01.RES.12.1.40 13975592

[70] Steendijk P Mur G Van Der Velde ET Baan J. The four-electrode resistivity technique in anisotropic media: theoretical analysis and application on myocardial tissue in vivo. IEEE Trans Biomed Eng. (1993) 40:1138–48. 10.1109/10.245632 8307598

[71] Steendijk P Velde ET Baan J. Dependence of anisotropic myocardial electrical resistivity on cardiac phase and excitation frequency. Basic Res Cardiol. (1994) 89:411–26. 10.1007/BF00788279 7702534

[72] Young RJ Panfilov AV. Anisotropy of wave propagation in the heart can be modeled by a riemannian electrophysiological metric. Proc Natl Acad Sci U S A. (2010) 107:15063–8. 10.1073/pnas.1008837107 20696934 PMC2930580

[73] Nielsen PM Le Grice IJ Smaill BH Hunter PJ. Mathematical model of geometry and fibrous structure of the heart. Am J Physiol Heart Circ Physiol. (1991) 260:H1365–78. 10.1152/ajpheart.1991.260.4.H1365 2012234

[74] Cukjati D Batiuskaite D Andre F Miklavcic D Mir L. Real time electroporation control for accurate and safe in vivo non-viral gene therapy. Bioelectrochemistry. (2007) 70:501–7. 10.1016/j.bioelechem.2006.11.001 17258942

[75] Ivorra A Al-Sakere B Rubinsky B Mir L. In vivo electrical conductivity measurements during and after tumor electroporation: conductivity changes reflect the treatment outcome. Phys Med Biol. (2009) 54:5949–63. 10.1088/0031-9155/54/19/019 19759406

[76] Zhao Y Bhonsle S Dong S Lv Y Liu H Safaai-Jazi A Characterization of conductivity changes during high-frequency irreversible electroporation for treatment planning. IEEE Trans Biomed Eng. (2018) 65:1810–9. 10.1109/TBME.2017.2778101 29989932

[77] Aliev MK Dos Santos P Hoerter JA Soboll S Tikhonov AN Saks VA. Water content and its intracellular distribution in intact and saline perfused rat hearts revisited. Cardiovasc Res. (2002) 53:48–58. 10.1016/S0008-6363(01)00474-6 11744012

[78] Scuderi M Dermol-Černe J Batista Napotnik T Chaigne S Bernus O Benoist D Characterization of experimentally observed complex interplay between pulse duration, electrical field strength, and cell orientation on electroporation outcome using a time-dependent nonlinear numerical model. Biomolecules. (2023) 13:727. 10.3390/biom13050727 37238597 PMC10216437

[79] Xie F Zemlin CW. Effect of twisted fiber anisotropy in cardiac tissue on ablation with pulsed electric fields. PLoS One. (2016) 11:e0152262. 10.1371/journal.pone.0152262 27101250 PMC4839574

[80] Song Y Zheng J Yan M Ding W Xu K Fan Q The effect of irreversible electroporation on the femur: experimental study in a rabbit model. Sci Rep. (2015) 5:18187. 10.1038/srep18187 26655843 PMC4674754

[81] Garcia PA Rossmeisl JH Neal RE Ellis TL Olson JD Henao-Guerrero N Intracranial nonthermal irreversible electroporation: in vivo analysis. J Membr Biol. (2010) 236:127–36. 10.1007/s00232-010-9284-z 20668843

[82] Garcia PA Neal RE Rossmeisl JH Davalos RV. Non-thermal irreversible electroporation for deep intracranial disorders. Annu Int Conf IEEE Eng Med Biol. (2010):2743–6. 10.1109/IEMBS.2010.5626371 21095962

[83] Hjouj M Last D Guez D Daniels D Sharabi S Lavee J MRI study on reversible and irreversible electroporation induced blood brain barrier disruption. PLoS One. (2012) 7:e42817. 10.1371/journal.pone.0042817 22900052 PMC3416789

[84] Wimmer T Srimathveeravalli G Gutta N Ezell PC Monette S Maybody M Planning irreversible electroporation in the porcine kidney: are numerical simulations reliable for predicting empiric ablation outcomes? Cardiovasc Intervent Radiol. (2015) 38:182–90. 10.1007/s00270-014-0905-2 24831827 PMC5497497

[85] Miklavčič D Šemrov D Mekid H Mir LM. A validated model of in vivo electric field distribution in tissues for electrochemotherapy and for DNA electrotransfer for gene therapy. Biochim Biophys Acta (2000) 1523:73–83. 10.1016/S0304-4165(00)00101-X 11099860

[86] Yao C Dong S Zhao Y Lv Y Liu H Gong L Bipolar microsecond pulses and insulated needle electrodes for reducing muscle contractions during irreversible electroporation. IEEE Trans Biomed Eng. (2017) 64:2924–37. 10.1109/TBME.2017.2690624 28391185

[87] Neal RE Millar JL Kavnoudias H Royce P Rosenfeldt F Pham A In vivo characterization and numerical simulation of prostate properties for non-thermal irreversible electroporation ablation. Prostate. (2014) 74:458–68. 10.1002/pros.22760 24442790

[88] Campelo S Valerio M Ahmed HU Hu Y Arena SL Neal RE An evaluation of irreversible electroporation thresholds in human prostate cancer and potential correlations to physiological measurements. APL Bioeng. (2017) 1:016101. 10.1063/1.5005828 31069281 PMC6481690

[89] Srimathveeravalli G Cornelis F Mashni J Takaki H Durack JC Solomon SB Comparison of ablation defect on MR imaging with computer simulation estimated treatment zone following irreversible electroporation of patient prostate. SpringerPlus. (2016) 5:219. 10.1186/s40064-016-1879-0 27026913 PMC4771651

[90] Gehl J Sorensen TH Nielsen K Raskmark P Nielsen SL Skovsgaard T In vivo electroporation of skeletal muscle: threshold, efficacy and relation to electric field distribution. Biochim Biophys Acta Gen Subj. (1999) 1428:233–40. 10.1016/S0304-4165(99)00094-X 10434041

[91] Pavselj N Bregar Z Cukjati D Batiuskaite D Mir LM Miklavcic D. The course of tissue permeabilization studied on a mathematical model of a subcutaneous tumor in small animals. IEEE Trans Biomed Eng. (2005) 52:1373–81. 10.1109/TBME.2005.851524 16119232

[92] Yao C Dong S Zhao Y Lv Y Liu H Gong L Bipolar microsecond pulses and insulated needle electrodes for reducing muscle contractions during irreversible electroporation. IEEE Trans Biomed Eng. (2017) 64:2924–37. 10.1109/TBME.2017.2690624 28391185

[93] Partridge BR O’Brien TJ Lorenzo MF Coutermarsh-Ott SL Barry SL Stadler K High-frequency irreversible electroporation for treatment of primary liver cancer: a proof-of-principle study in canine hepatocellular carcinoma. J Vasc Interv Radiol. (2020) 31:482–491.e4. 10.1016/j.jvir.2019.10.015 31956003 PMC7418213

[94] Sweeney DC Reberšek M Dermol J Rems L Miklavčič D Davalos RV. Quantification of cell membrane permeability induced by monopolar and high-frequency bipolar bursts of electrical pulses. Biochim Biophys Acta. (2016) 1858:2689–98. 10.1016/j.bbamem.2016.06.024 27372268

[95] O’Brien TJ Passeri M Lorenzo MF Sulzer JK Lyman WB Swet JH Experimental high-frequency irreversible electroporation using a single-needle delivery approach for nonthermal pancreatic ablation in vivo. J Vasc Interv Radiol. (2019) 30:854–862.e7. 10.1016/j.jvir.2019.01.032 31126597

[96] Eisner DA Caldwell JL Trafford AW Hutchings DC. The control of diastolic calcium in the heart: basic mechanisms and functional implications. Circ Res. (2020) 126:395–412. 10.1161/CIRCRESAHA.119.315891 31999537 PMC7004450

[97] Cukjati D Batiuskaite D André F Miklavcic D Mir LM. Real time electroporation control for accurate and safe in vivo non-viral gene therapy. Bioelectrochemistry Amst Neth. (2007) 70:501–7. 10.1016/j.bioelechem.2006.11.001 17258942

[98] Chaigne S Sigg DC Stewart MT Hocini M Batista Napotnik T Miklavčič D Reversible and irreversible effects of electroporation on contractility and calcium homeostasis in isolated cardiac ventricular myocytes. Circ Arrhythm Electrophysiol. (2022) 15:762–72. 10.1161/CIRCEP.122.011131 PMC9665944 36306333

[99] Dermol-Černe J Batista Napotnik T Reberšek M Miklavčič D. Short microsecond pulses achieve homogeneous electroporation of elongated biological cells irrespective of their orientation in electric field. Sci Rep. (2020) 10:9149. 10.1038/s41598-020-65830-3 32499601 PMC7272635

