# Supplementary material for: Determination of lethal electric field threshold for pulsed field ablation in ex vivo perfused porcine and human hearts
Source: Front Cardiovasc Med. 2023 Jun 23;10:1160231. doi: 10.3389/fcvm.2023.1160231 (PMC10326317; doi:10.3389/fcvm.2023.1160231)

Supplementary Material

Determination of lethal electric field threshold for pulsed field ablation in ex vivo perfused porcine and human hearts

Bor Kos, Lars Mattison, David Ramirez, Helena Cindrič, Daniel C. Sigg, Paul Iaizzo, Mark Stewart, Damijan Miklavčič

*** Correspondence:** Corresponding Author: email@uni.edu

1 Supplementary Tables

Table S1: All MDT pig data (Lesion N, Experiment number, LET, AR, EF, exact angle)

Heart number	Voltage [V]	exactAngle [°]	LET [V/cm]	ARSP	EFSP
**1**	500	59	623	3.25	1.01
**1**	1000	29	675	4.28	1.01
**1**	500	56	508	1.32	1.68
**1**	700	-80	530	1.63	1.48
**1**	1000	81	555	1.23	2.63
**1**	1100	-86	585	1.92	4.32
**1**	1200	-68	544	1.02	5.44
**1**	1200	-55	428	1.07	9.91
**2**	500	-64	483	1.05	2.34
**2**	700	-25	437	5.22	2.54
**2**	1000	29	1000	3.25	1.01
**2**	1100	84	550	1.94	3.71
**2**	1200	50	534	1.34	1.97
**2**	500	23	549	1.75	2.84
**2**	700	-41	486	3.52	9.81
**2**	1000	87	527	1.33	2.22
**2**	1100	72	501	1.83	7.26
**2**	1200	-74	434	1.00	8.38
**2**	1200	-35	453	3.23	4.32
**2**	1000	5	727	1.24	3.14
**2**	1200	13	464	3.67	1.00
**3**	500	-2	408	1.03	1.00
**3**	700	-18	511	1.00	1.88
**3**	1000	56	437	3.29	1.29
**3**	1100	-73	498	1.06	2.20
**3**	1200	-56	358	2.17	2.20

**3**	1200	50	507	1.75	4.06
**3**	1200	26	559	1.59	3.03
**3**	1200	-78	500	1.06	4.00
**3**	1300	51	646	2.38	2.65
**4**	1200	-89	588	1.33	2.70
**4**	1300	-84	538	1.43	3.27
**4**	1200	87	543	2.15	3.18
**4**	1300	43	546	1.06	2.28
**4**	1200	-5	558	1.39	3.07
**4**	1300	7	503	1.06	3.11
**5**	1200	-79	604	1.06	2.20
**5**	1200	-84	535	1.04	2.05
**5**	1300	-78	507	1.01	4.04
**5**	1300	-84	424	1.43	9.85
**5**	700	-45	513	3.97	1.07
**5**	1200	-81	542	1.09	6.54
**5**	1000	-85	501	1.02	1.50
**6**	1200	2	712	1.06	3.19
**6**	1200	9	802	1.38	1.38
**6**	1300	-1	744	1.06	3.19
**6**	1300	6	551	1.52	2.81
**6**	700	24	635	6.21	1.00
**6**	1000	6	701	1.06	3.11
**6**	1100	-2	474	1.04	1.01
**6**	1200	-31	570	1.03	1.21

Table S2: All MDT human data (Lesion N, Experiment number, LET, AR, EF, exact angle)

Heart number	Voltage [V]	exactAngle [°]	LET [V/cm]	ARSP	EFSP
**1**	700	25	567	1.04	1.08
**1**	700	47	572	1.43	1.00
**1**	1000	63	408	1.06	2.20
**1**	1000	58	495	1.03	1.40
**1**	1300	62	423	1.33	1.18
**1**	1300	79	465	1.06	2.20
**1**	1500	38	198	1.33	1.03
**1**	1500	51	352	1.06	2.20
**1**	1500	69	416	2.18	9.94
**2**	1200	-27	371	1.01	1.59
**2**	1300	-23	382	1.06	3.19
**2**	1500	-2	397	2.11	1.00

**2**	1000	-18	340	1.06	2.20
**2**	1200	-26	327	1.06	3.19
**2**	700	9	624	4.28	1.01
**2**	1000	77	557	3.15	1.90
**2**	1200	40	502	1.57	1.76
**3**	700	11	262	2.29	1.06
**3**	700	64	704	4.28	1.01
**3**	1200	-67	175	1.06	3.19
**3**	1500	-15	497	1.84	2.67

Table S3: All 100 μs pig data (Lesion N, Experiment number, LET, AR, EF, exact angle)

**Heart number**	**Voltage [V]**	**exactAngle [°]**	**LET [V/cm]**	**ARSP**	**EFSP**
**1**	600	10	415	1.08	3.24
**1**	800	8	293	2.33	1.06
**1**	1000	-2	351	1.06	3.19
**2**	600	68	418	3.09	8.88
**2**	800	31	313	1.40	2.18
**2**	1200	20	171	1.05	3.03
**3**	1000	-80	376	3.20	9.98
**3**	1200	63	419	3.00	2.05
**4**	1000	88	477	6.33	1.95
**4**	1200	57	321	4.85	1.09
**4**	1200	77	215	5.14	1.05
**4**	600	67	224	2.90	1.02
**4**	800	80	389	2.66	2.81
**4**	1000	86	368	4.30	3.58
**5**	600	2	261	1.86	1.02
**5**	600	16	402	1.94	2.46
**5**	800	10	365	1.06	3.03
**5**	800	4	400	2.46	7.65
**6**	600	10	307	1.24	3.14
**6**	600	12	340	1.95	6.23
**6**	800	6	264	2.03	1.12
**6**	800	-3	461	1.24	3.14
**7**	600	13	290	3.25	1.01
**7**	800	26	307	1.22	1.07
**7**	1000	-87	478	3.01	2.80
**8**	1000	41	444	3.19	4.25
**8**	800	-50	432	2.28	2.08
**9**	600	-18	359	4.97	8.47
**9**	600	-27	424	5.70	9.95

**9**	800	74	462	1.58	2.64
**9**	800	60	493	2.21	2.68

2 Supplementary Figures

2.1 Experimental validation

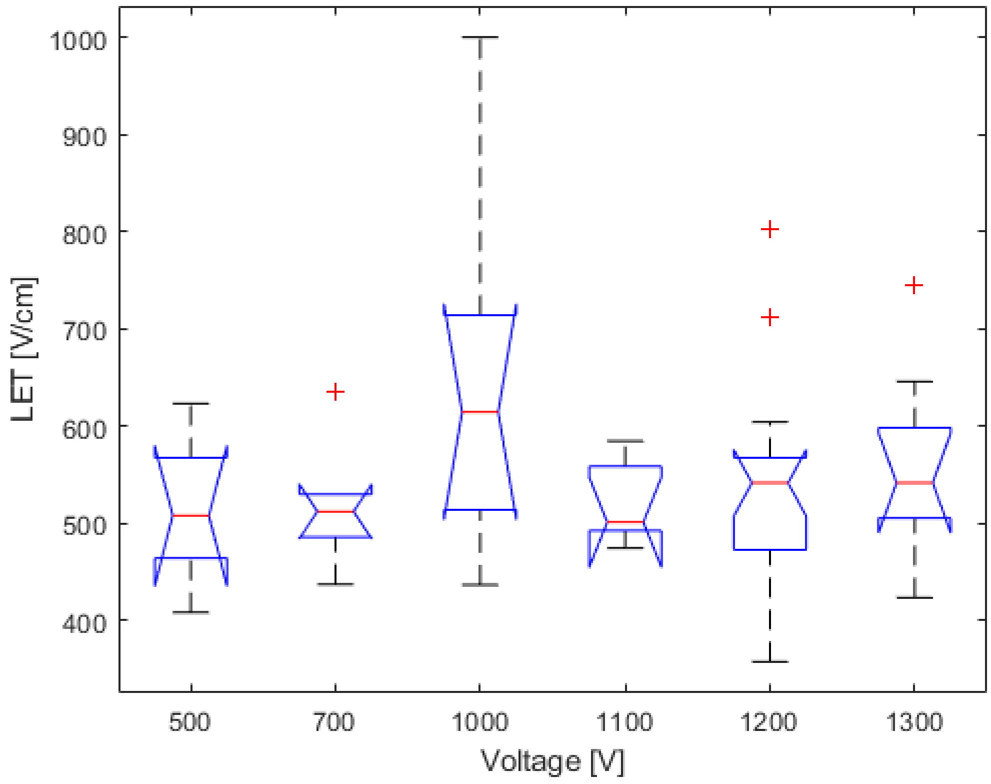

Supplementary Figure S1. ANOVA (Kruskal-Wallis) plot for LET vs Voltage in MDT pig

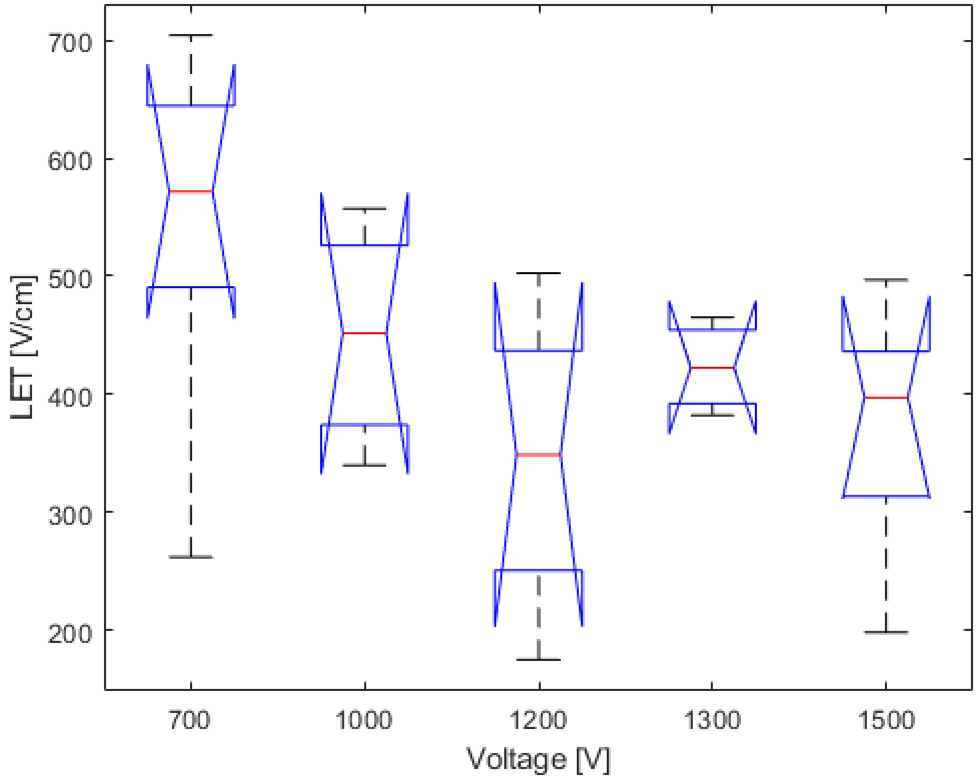

Supplementary Figure S2. ANOVA (Kruskal-Wallis) plot for LET vs Voltage in MDT human

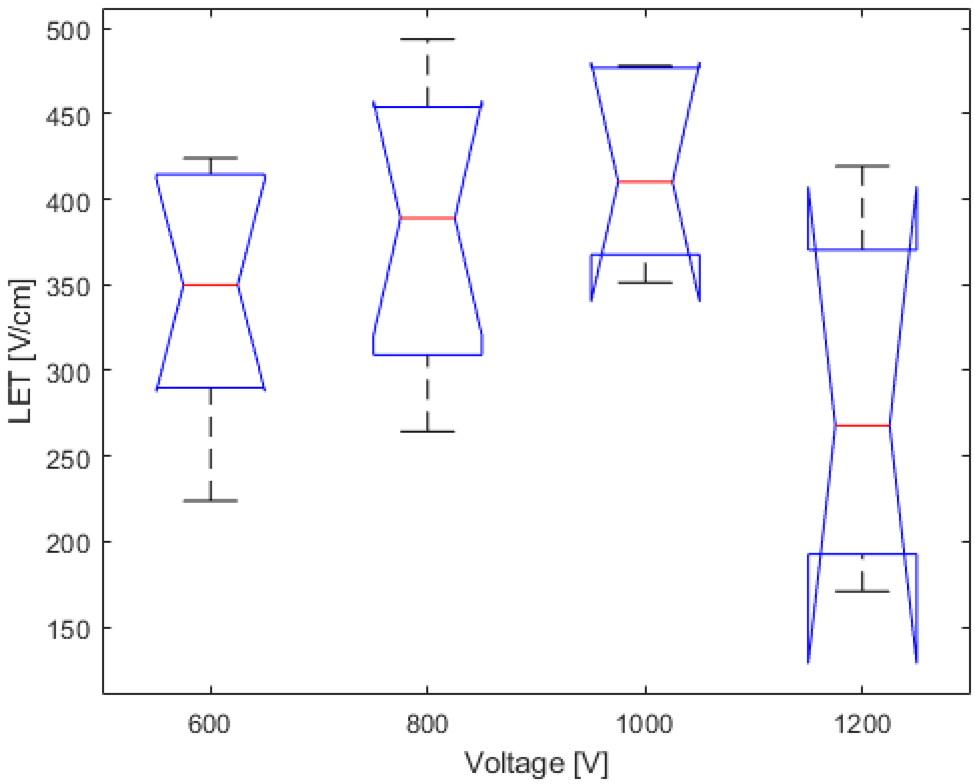

Supplementary Figure S3. ANOVA (Kruskal-Wallis) plot for LET vs Voltage in 100 µs pig

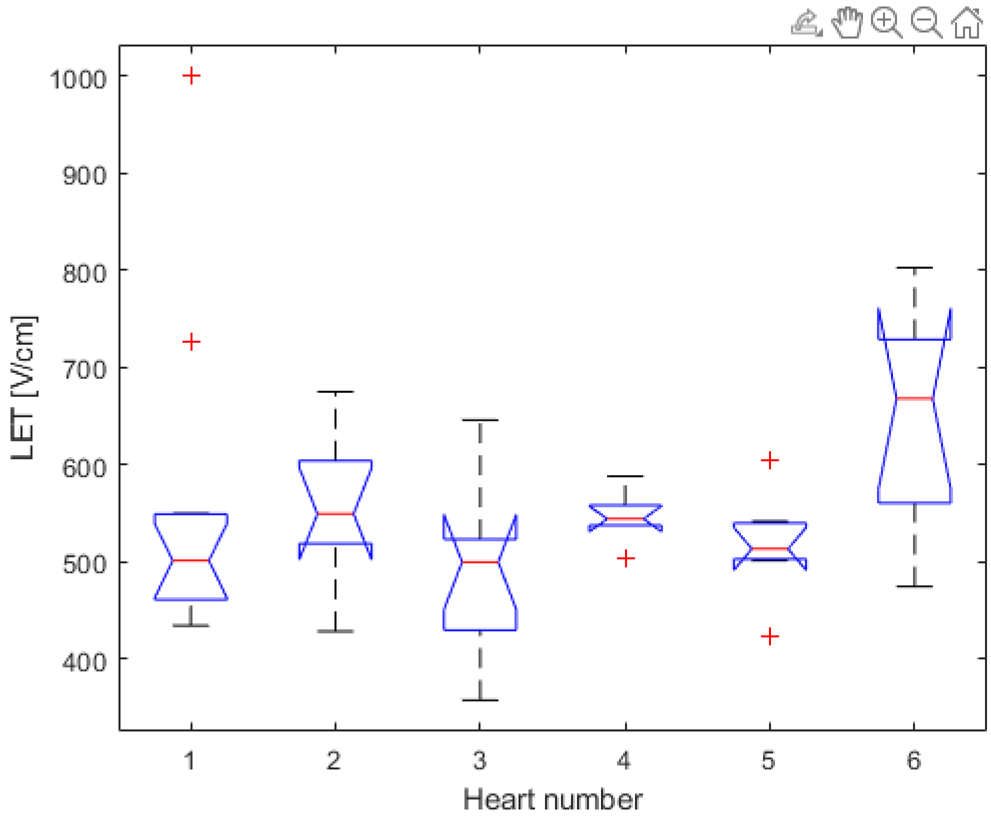

Supplementary Figure S4. ANOVA (Kruskal-Wallis) plot for LET vs heart sample in MDT pig

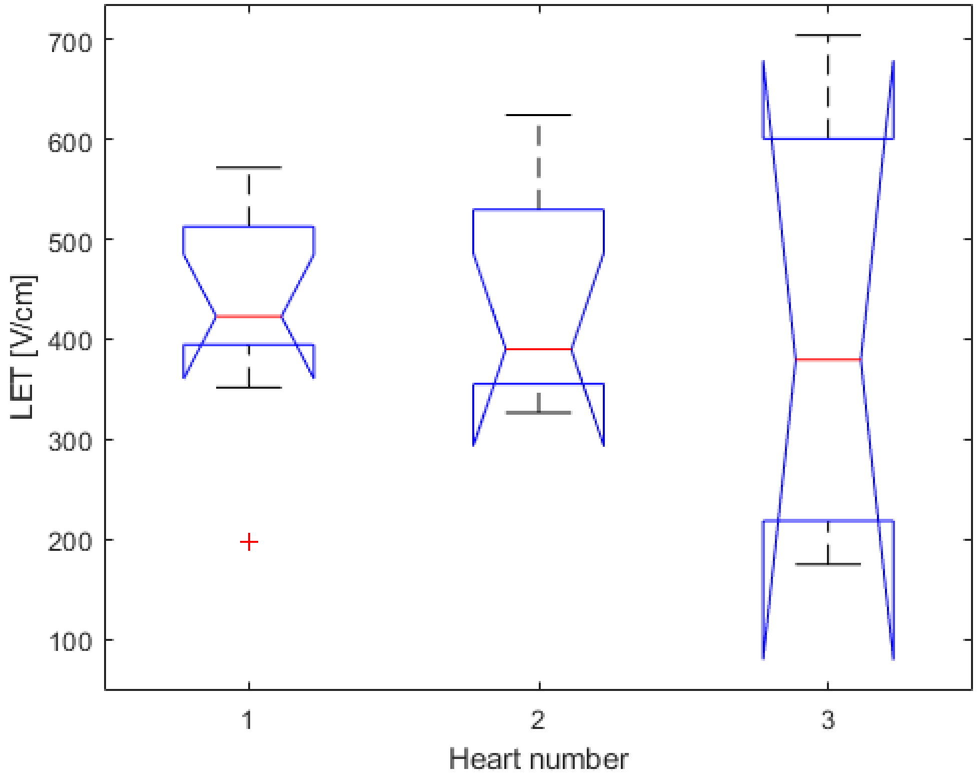

Supplementary Figure S5. ANOVA (Kruskal-Wallis) plot for LET vs heart sample in MDT human

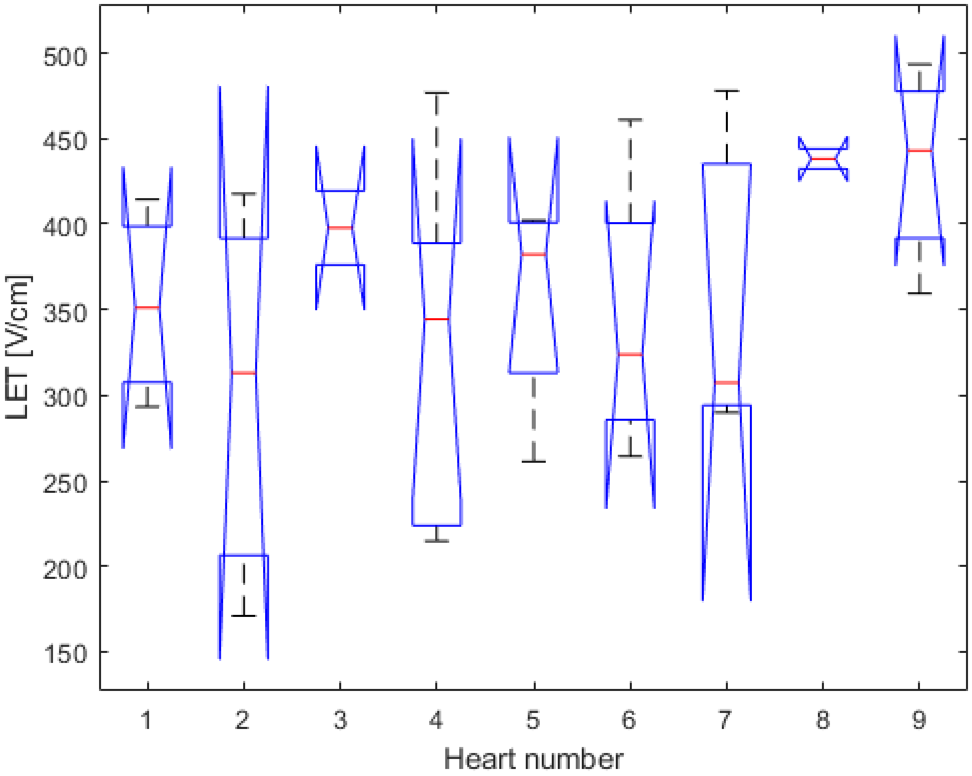

Supplementary Figure S6. ANOVA (Kruskal-Wallis) plot for LET vs heart sample in 100 μs pig

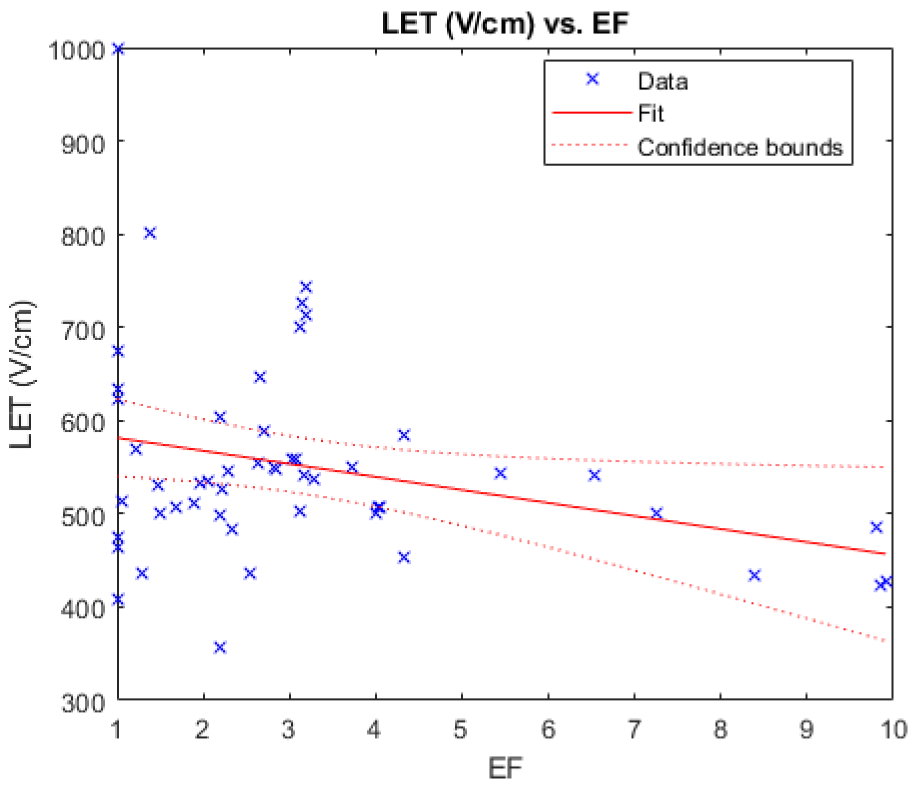

Supplementary Figure S7. linear fit model of LET vs EF in MDT pig

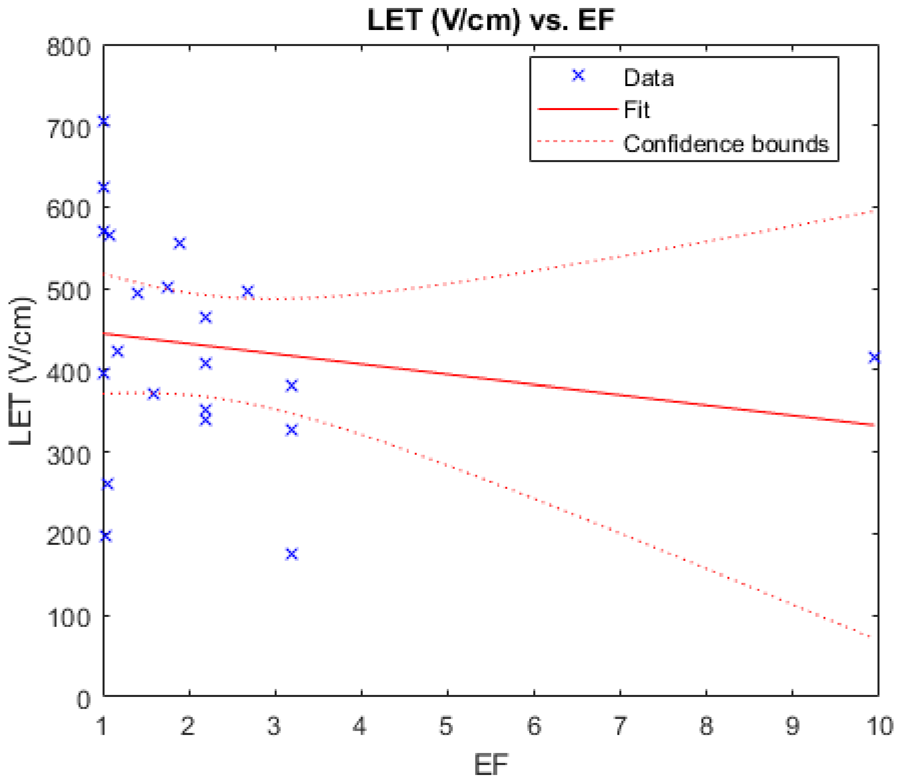

Supplementary Figure S8. linear fit model of LET vs EF in MDT human

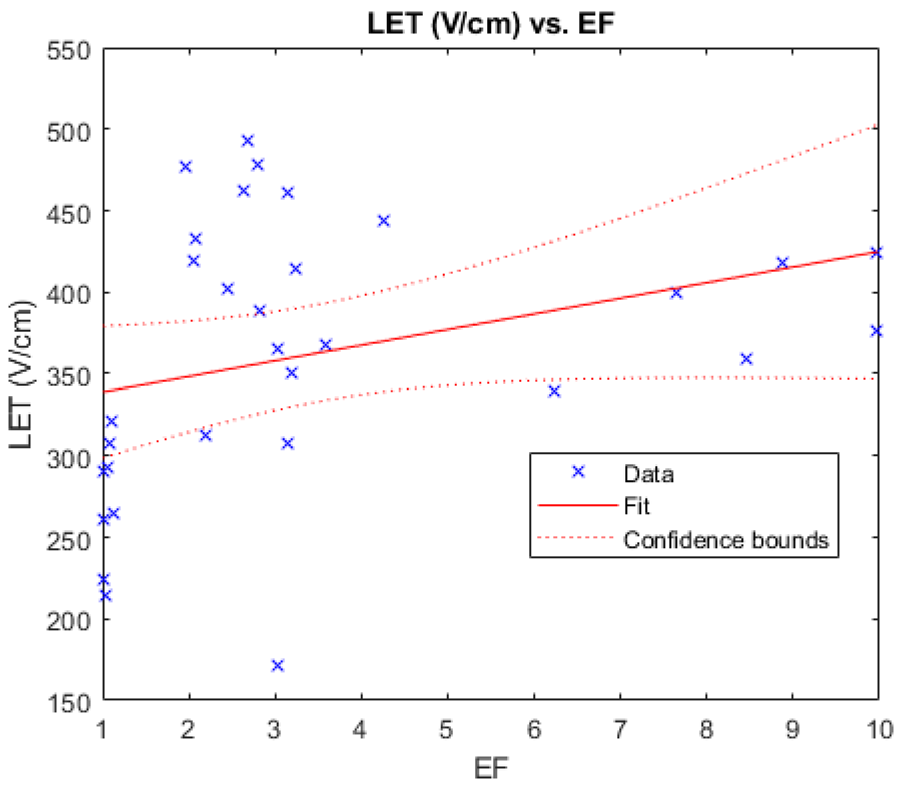

Supplementary Figure S9. linear fit model of LET vs EF in 100 µs pig

3 Lesion images vs model for MDT pig

Lesion 1, Voltage=500, Angle=59.1, LET= 623, AR=3.25, EF=1.01, Dice=0.819

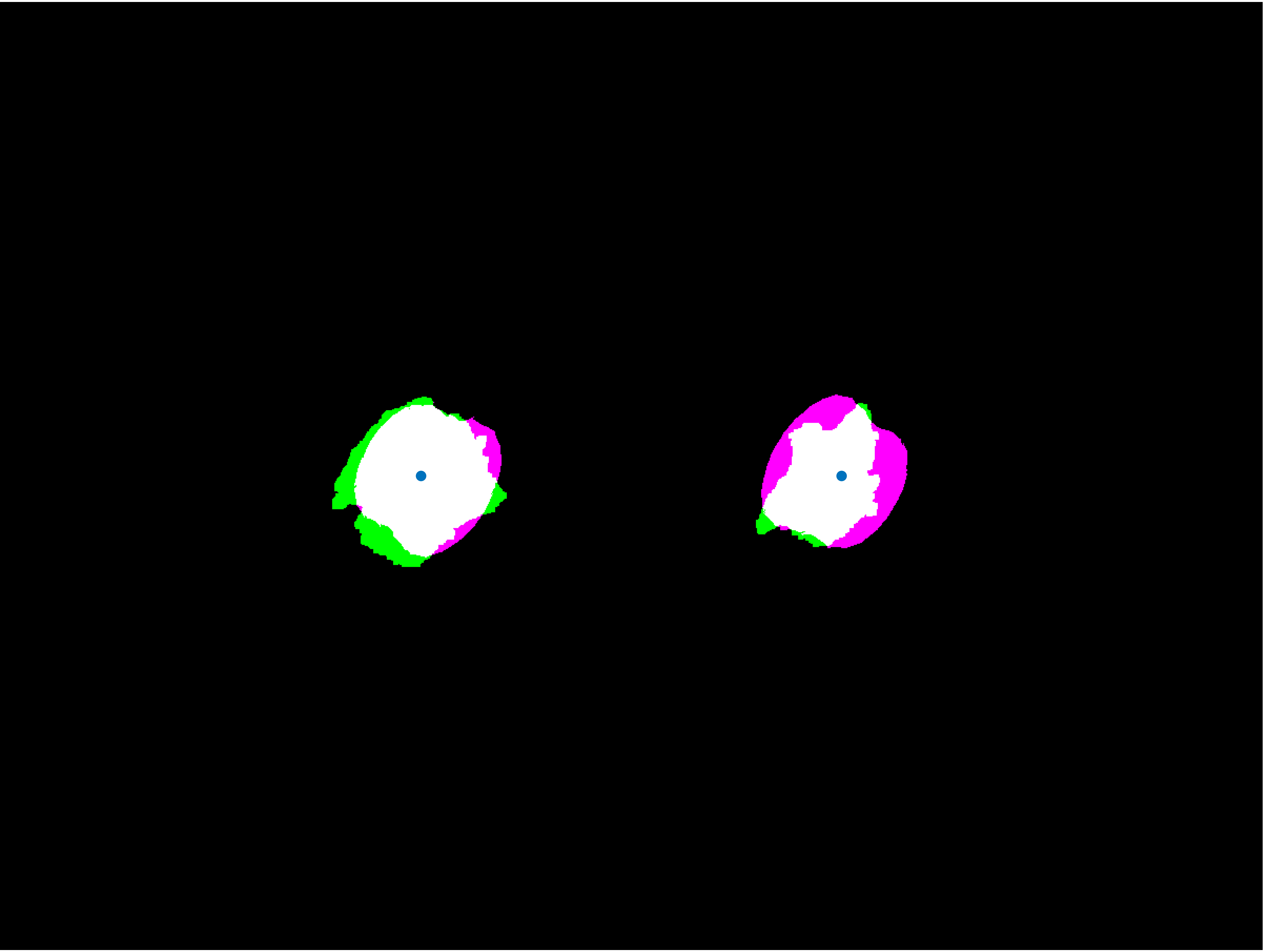

Lesion 2, Voltage=1000, Angle=29.1, LET= 675, AR=4.28, EF=1.01, Dice=0.803

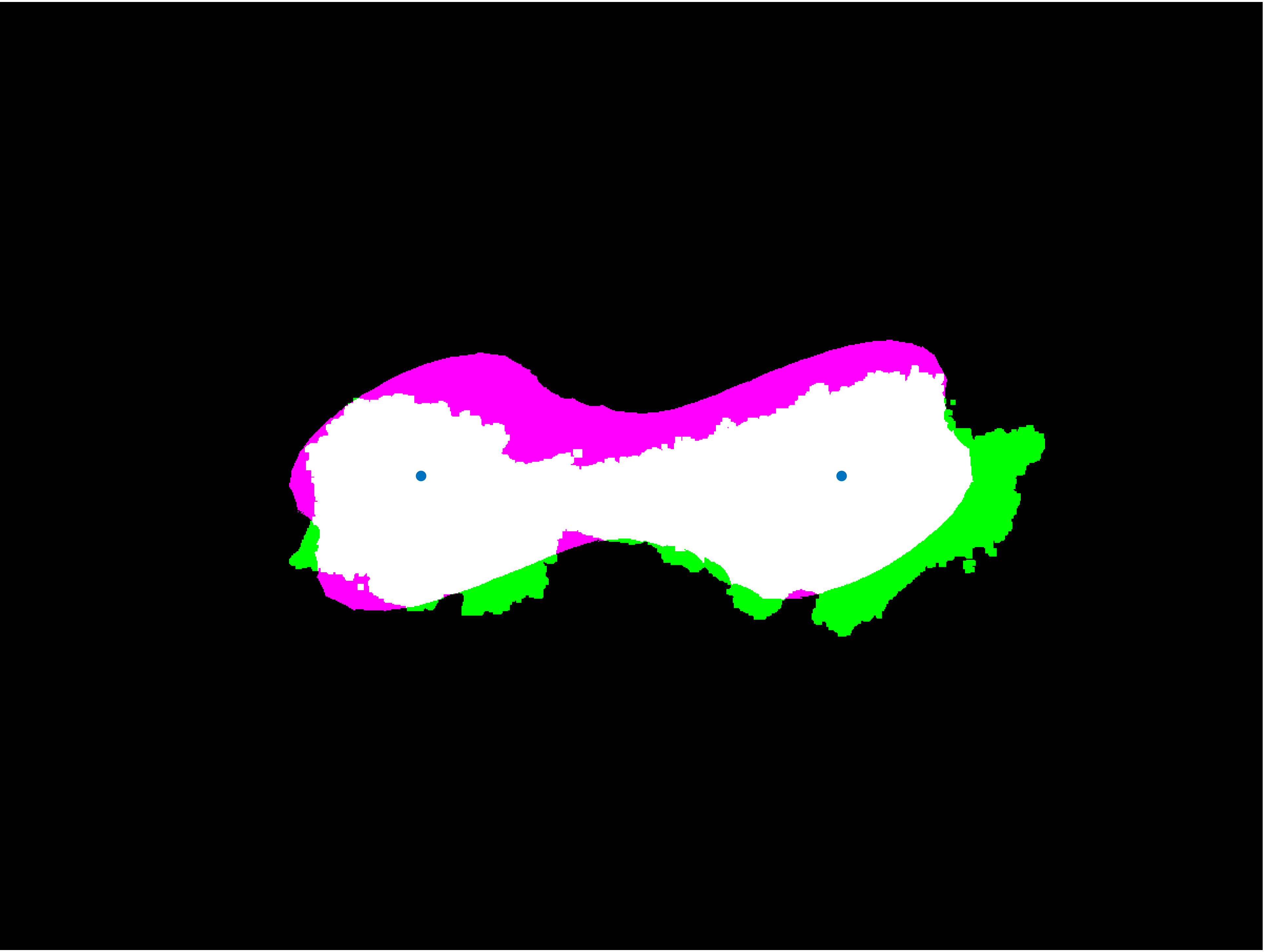

Lesion 3, Voltage=500, Angle=56.1, LET= 508, AR=1.32, EF=1.68, Dice=0.777

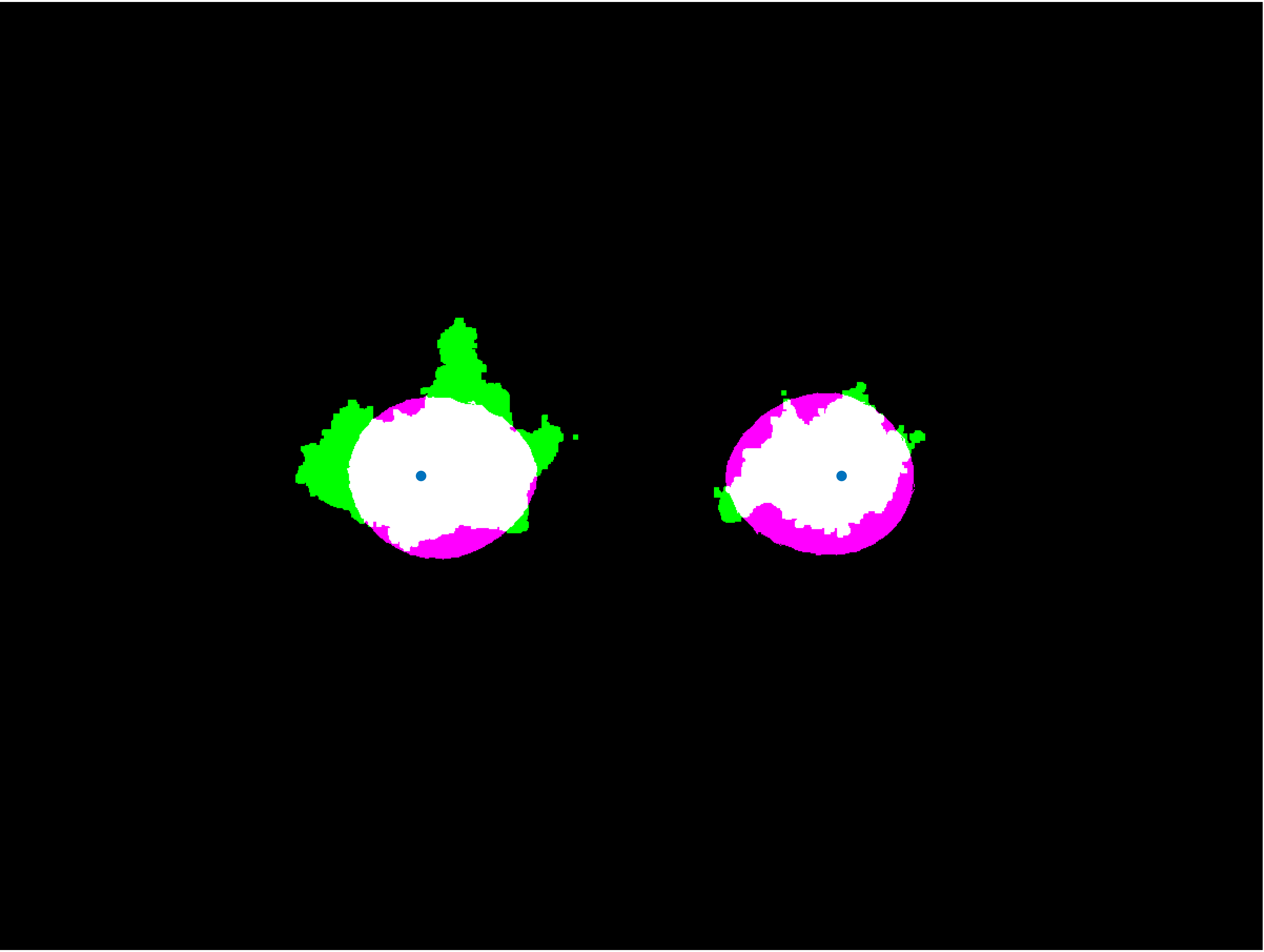

Lesion 4, Voltage=700, Angle=-80.1, LET= 530, AR=1.63, EF=1.48, Dice=0.835

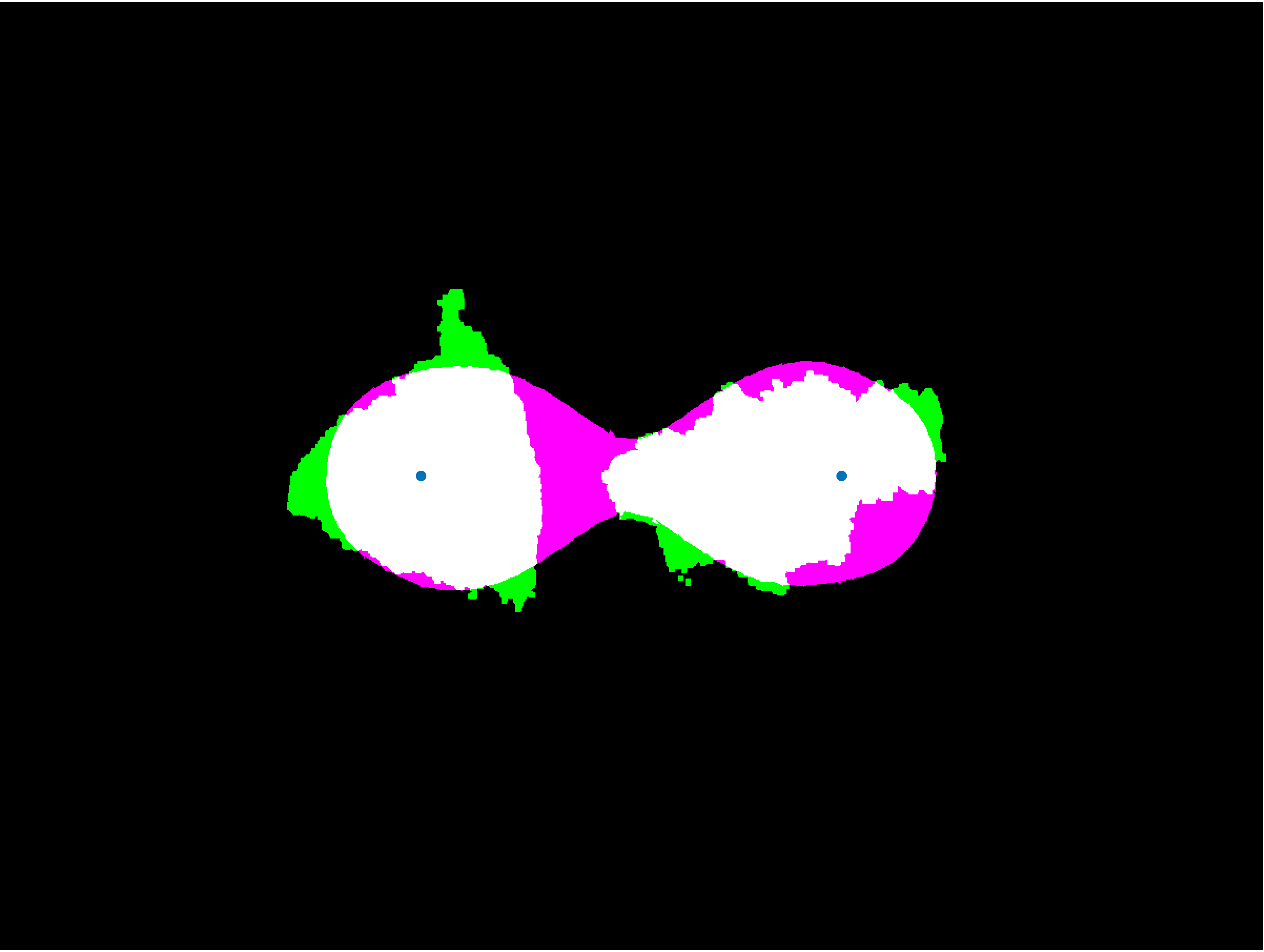

Lesion 5, Voltage=1000, Angle=80.9, LET= 555, AR=1.23, EF=2.63, Dice=0.899

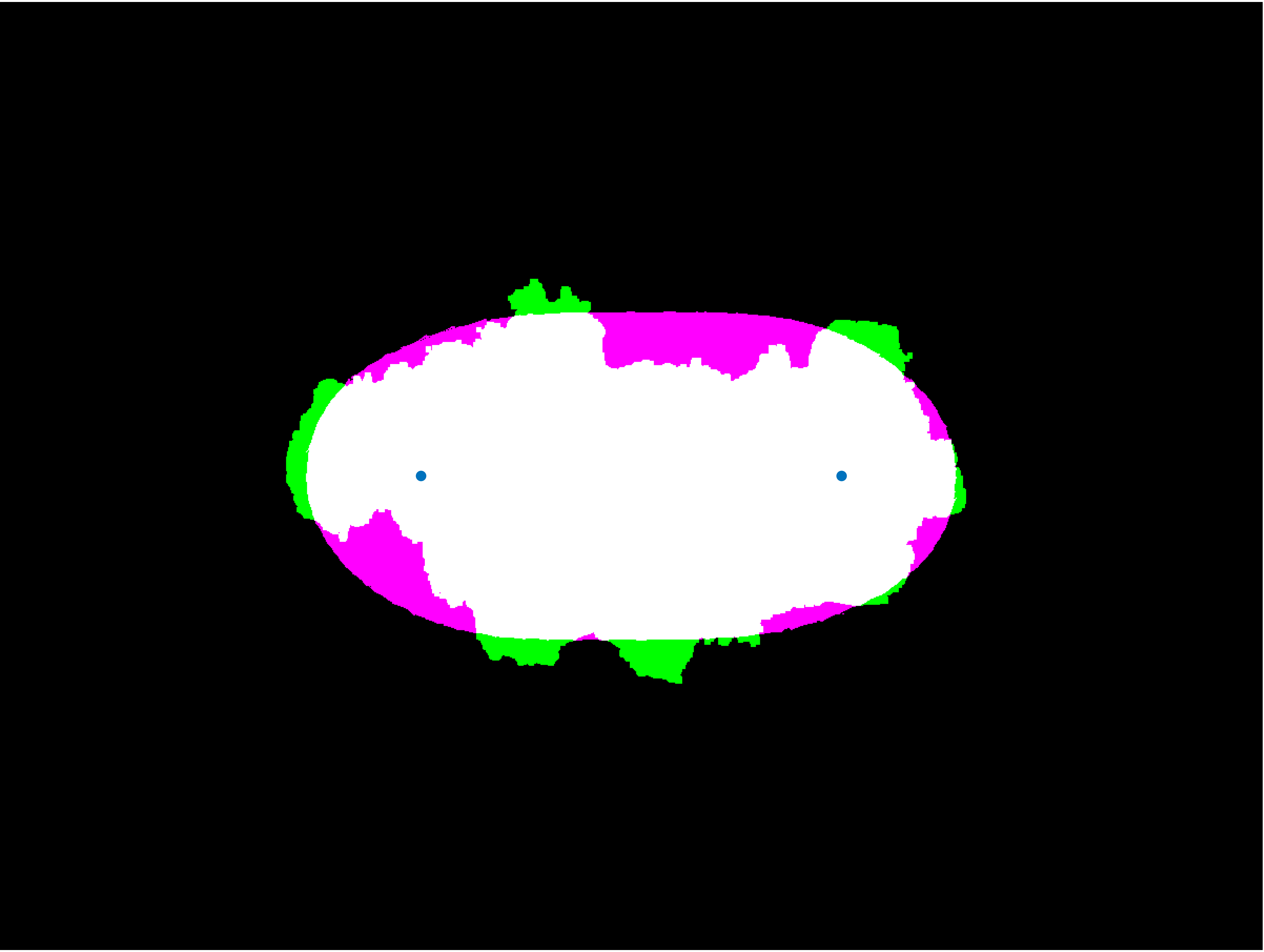

Lesion 6, Voltage=1100, Angle=-86.3, LET= 585, AR=1.92, EF=4.32, Dice=0.925

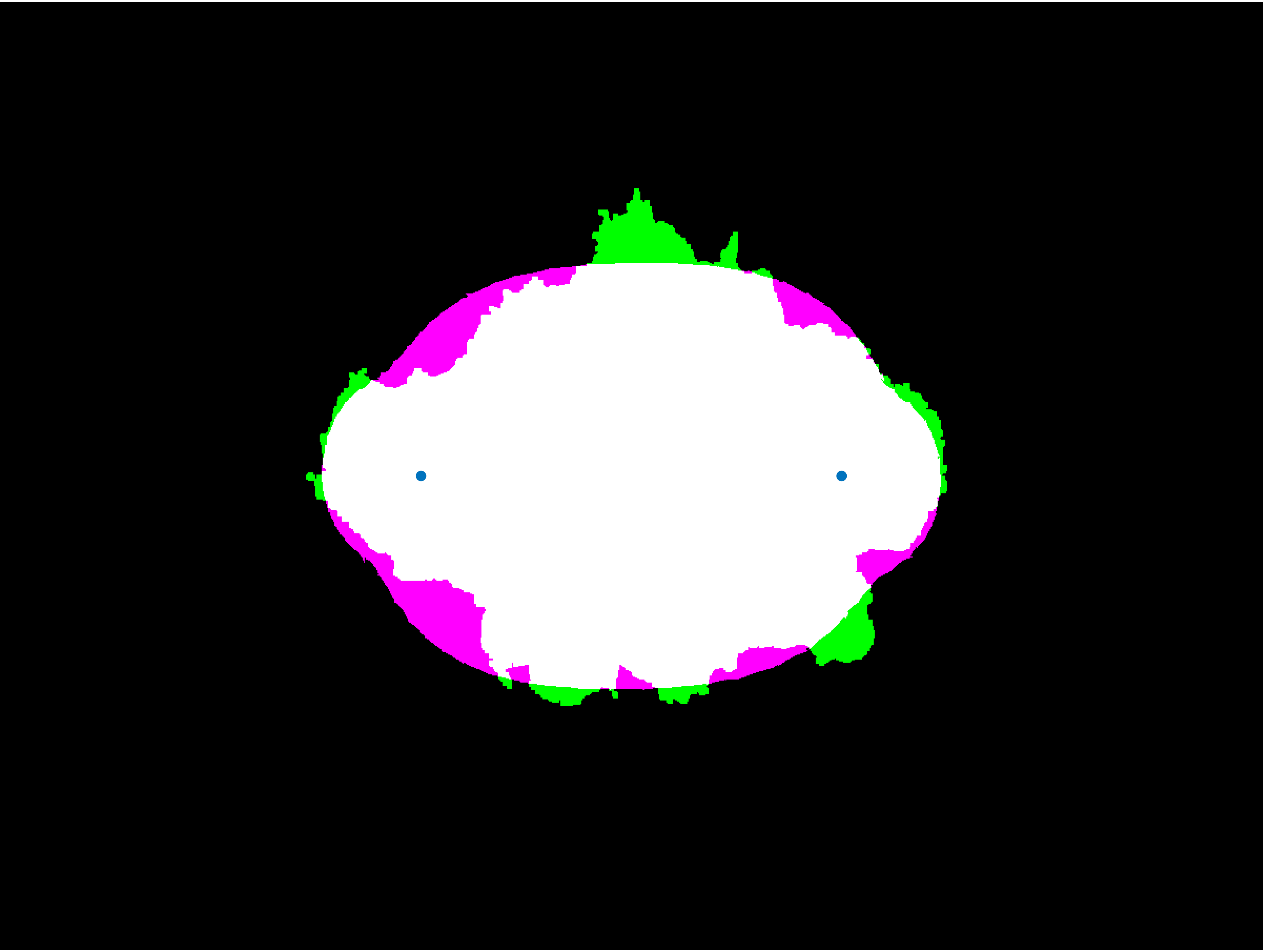

Lesion 7, Voltage=1200, Angle=-67.7, LET= 544, AR=1.02, EF=5.44, Dice=0.844

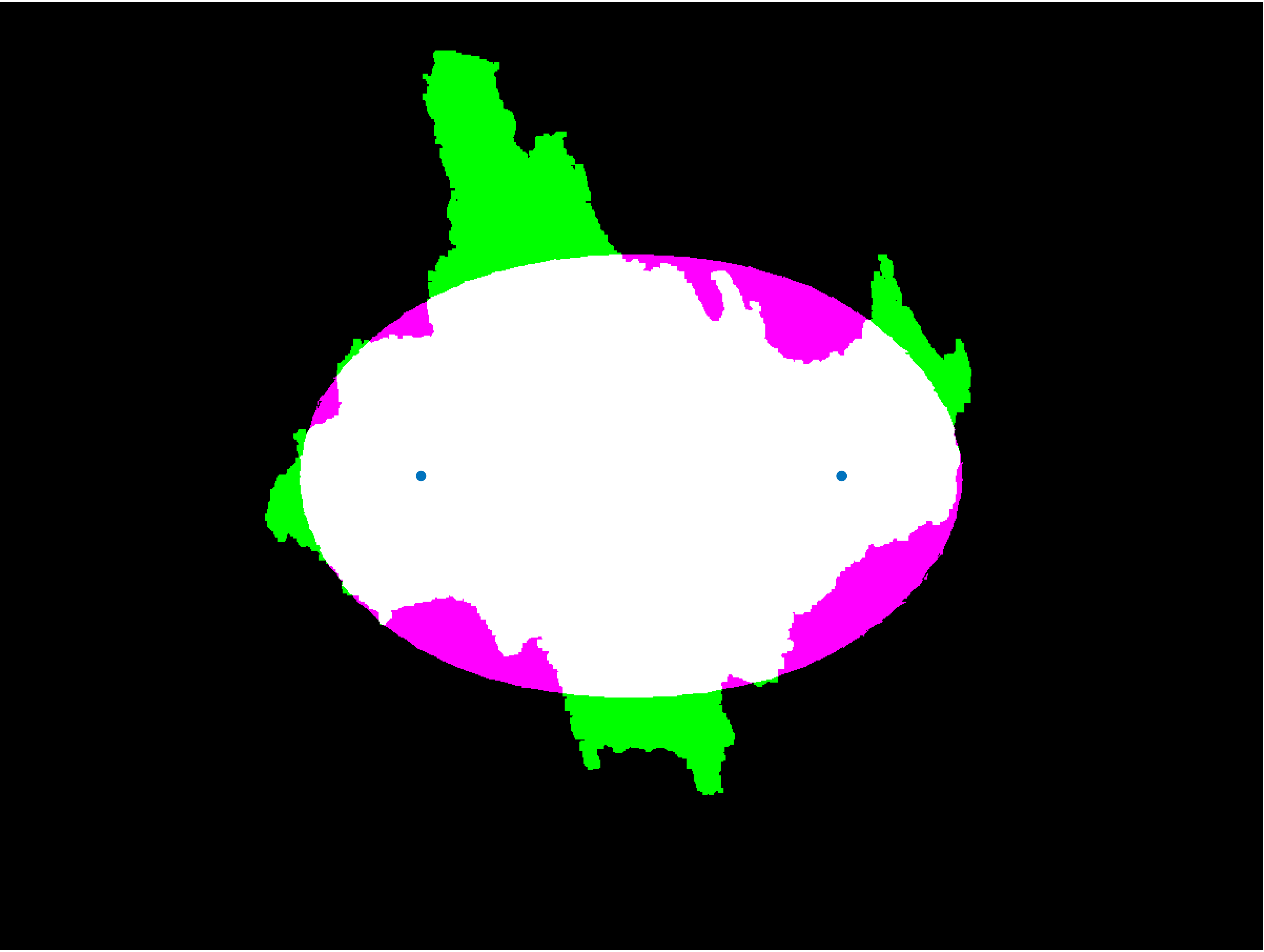

Lesion 8, Voltage=1200, Angle=-54.6, LET= 428, AR=1.07, EF=9.91, Dice=0.881

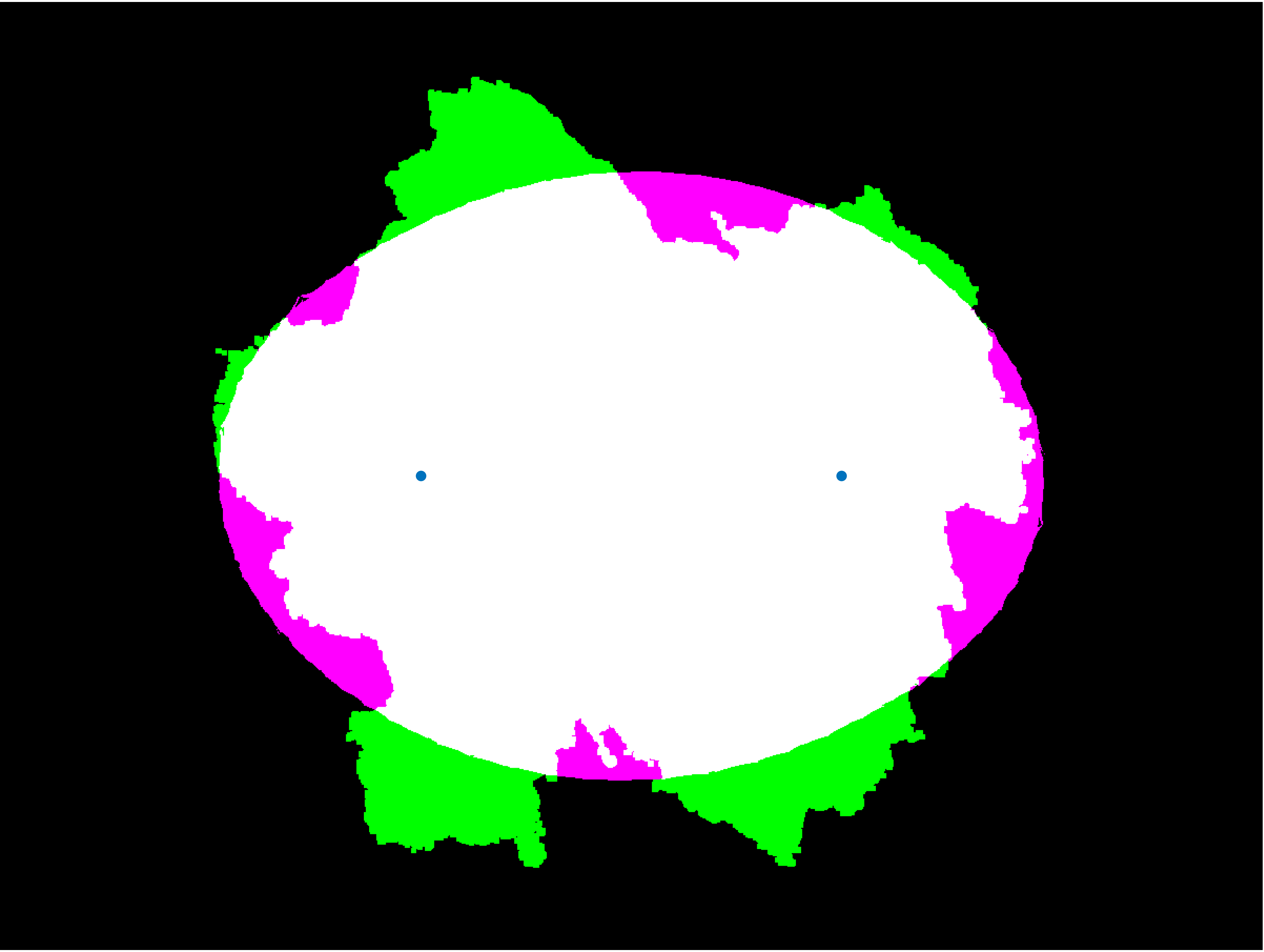

Lesion 9, Voltage=500, Angle=-63.8, LET= 483, AR=1.05, EF=2.34, Dice=0.703

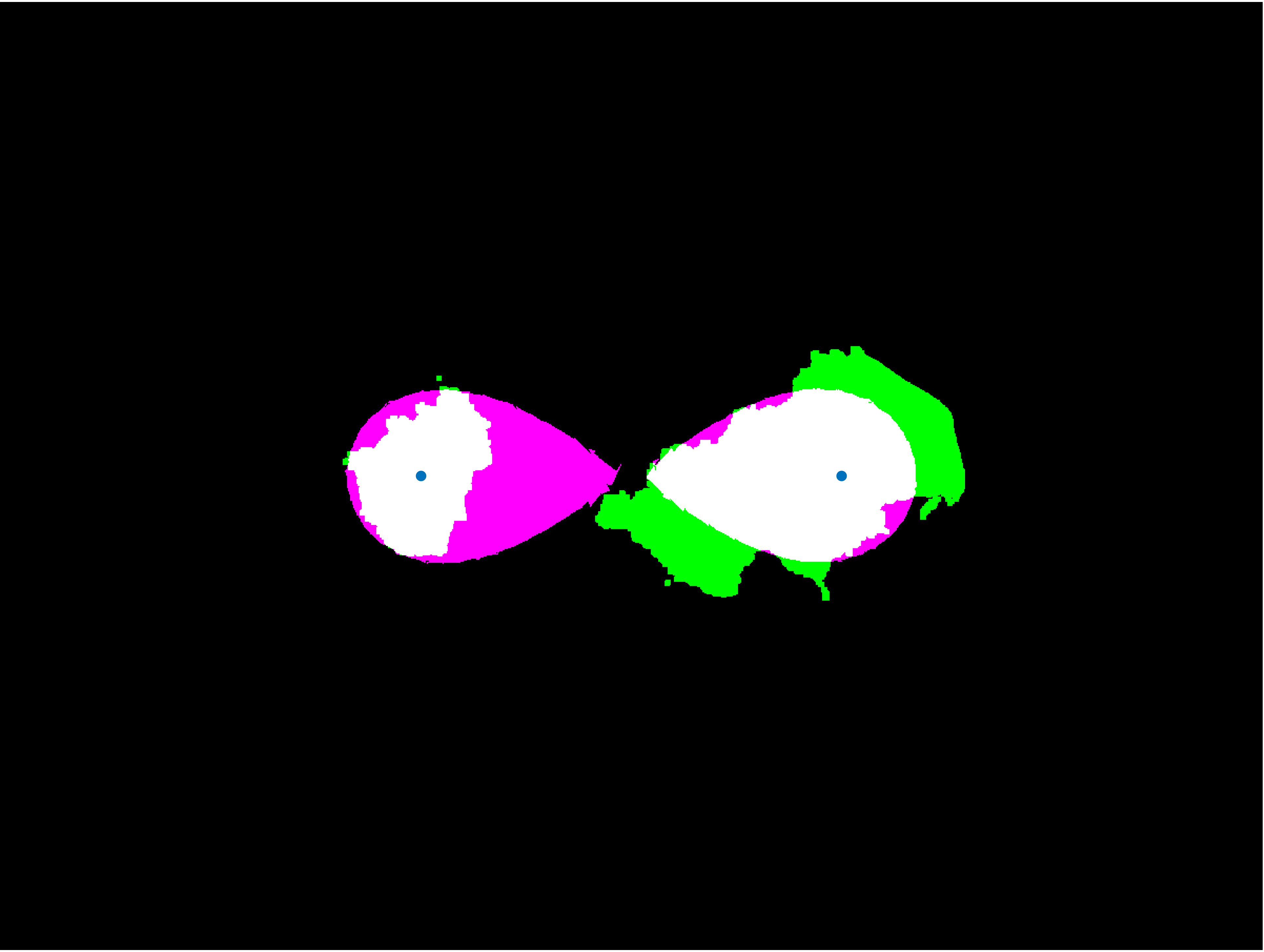

Lesion 10, Voltage=700, Angle=-24.8, LET= 437, AR=5.22, EF=2.54, Dice=0.832

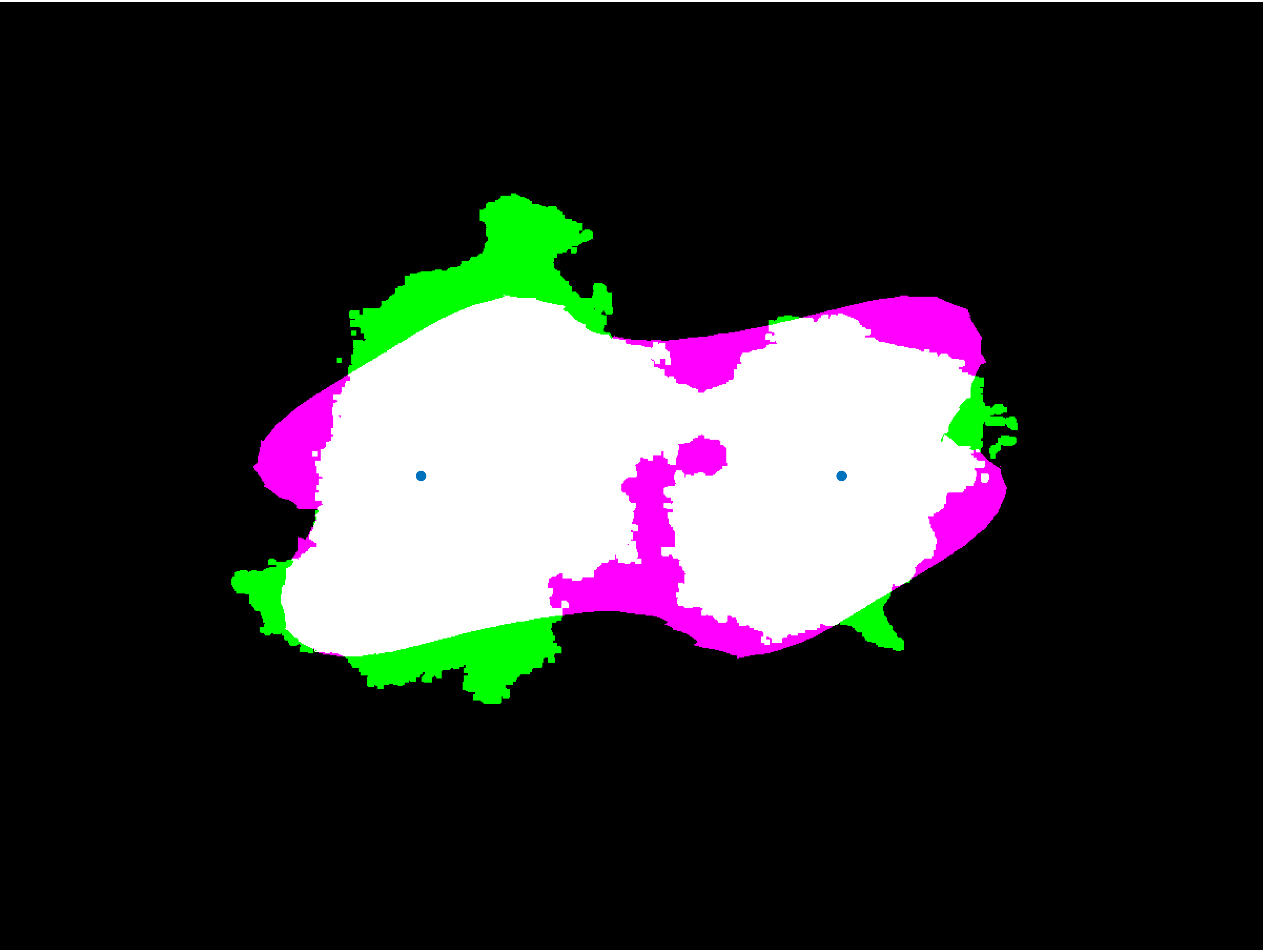

Lesion 11, Voltage=1000, Angle=29.4, LET=1000, AR=3.25, EF=1.01, Dice=0.843

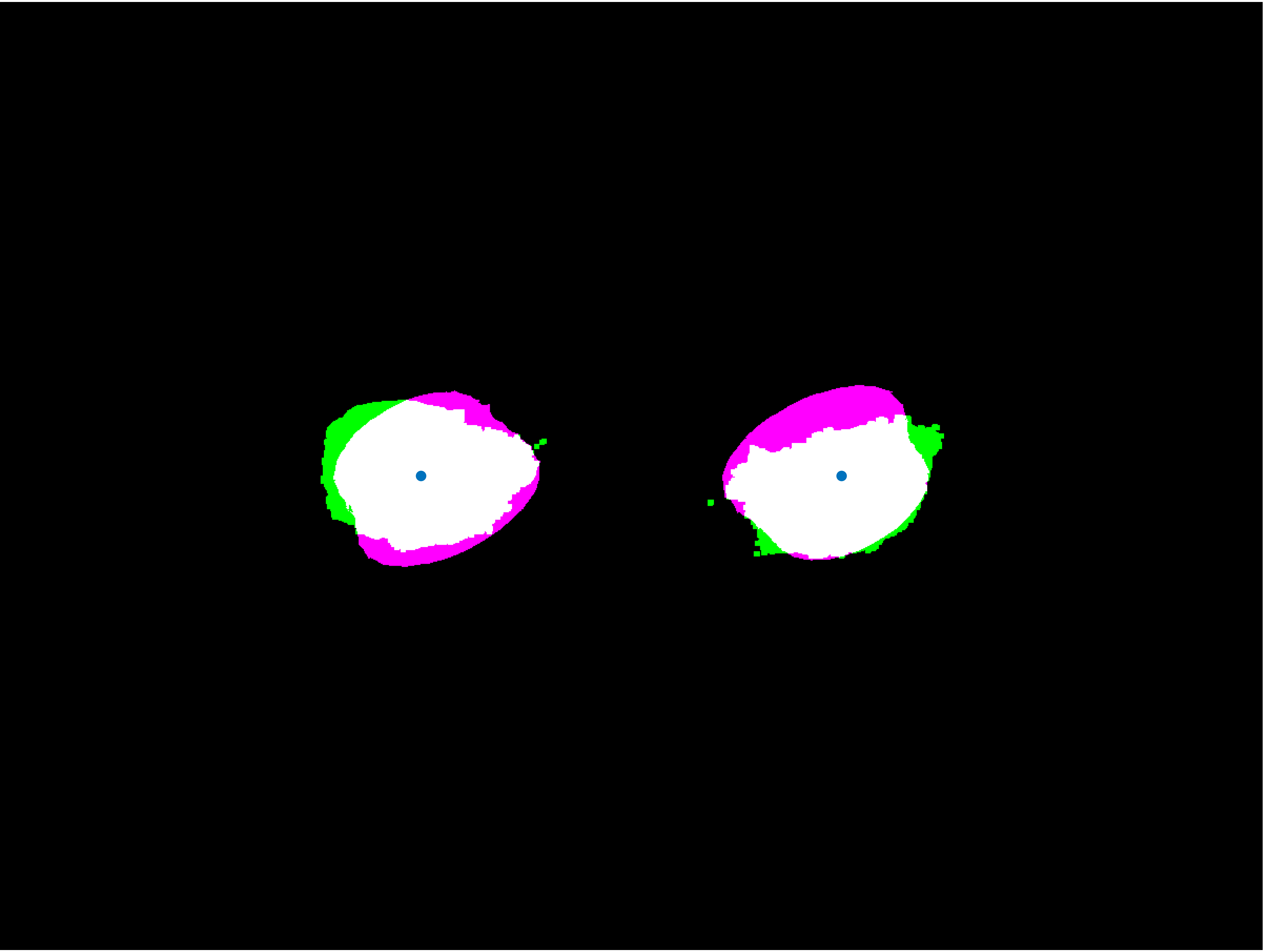

Lesion 12, Voltage=1100, Angle=83.7, LET= 550, AR=1.94, EF=3.71, Dice=0.871

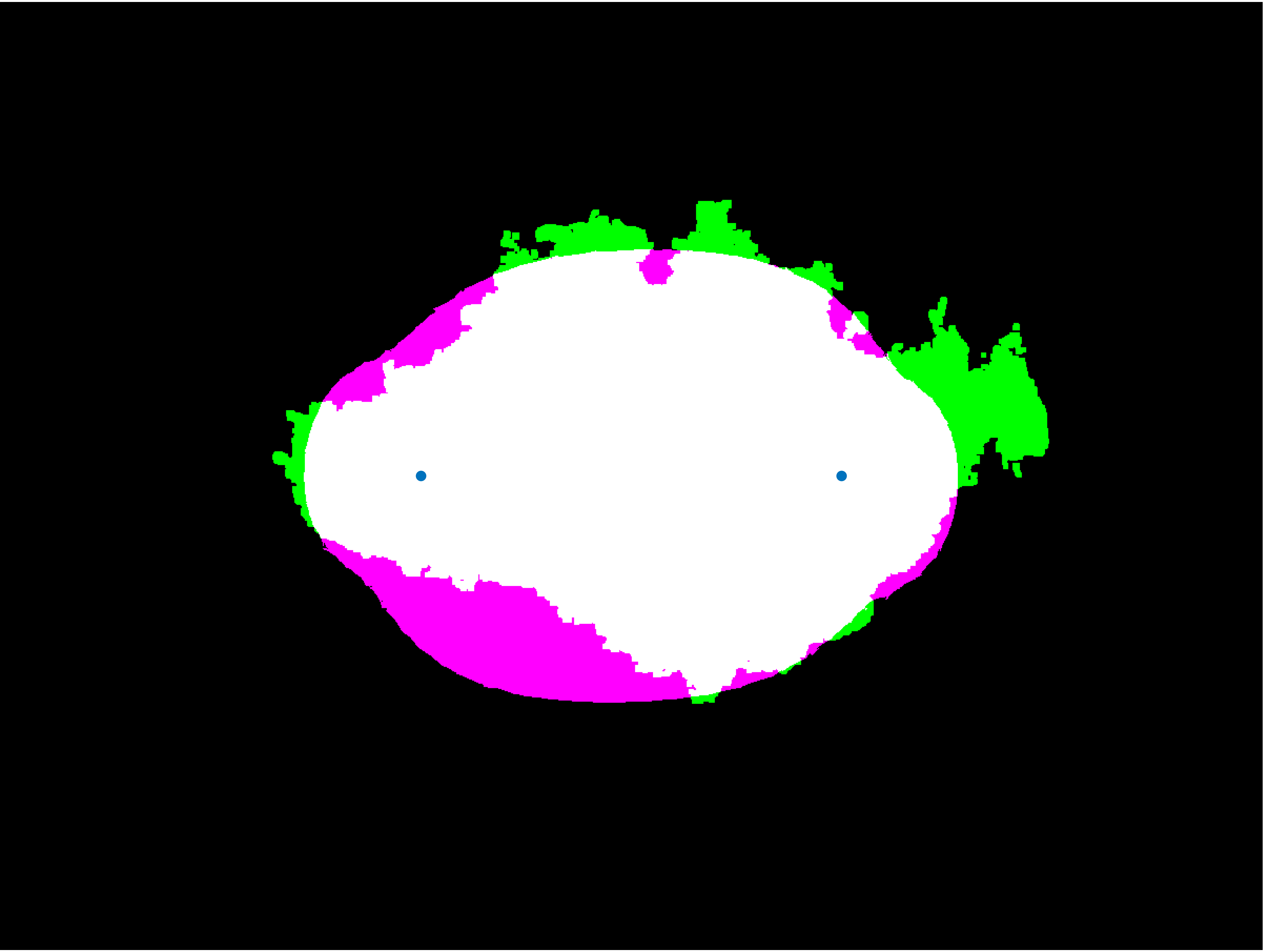

Lesion 13, Voltage=1200, Angle=50.3, LET= 534, AR=1.34, EF=1.97, Dice=0.890

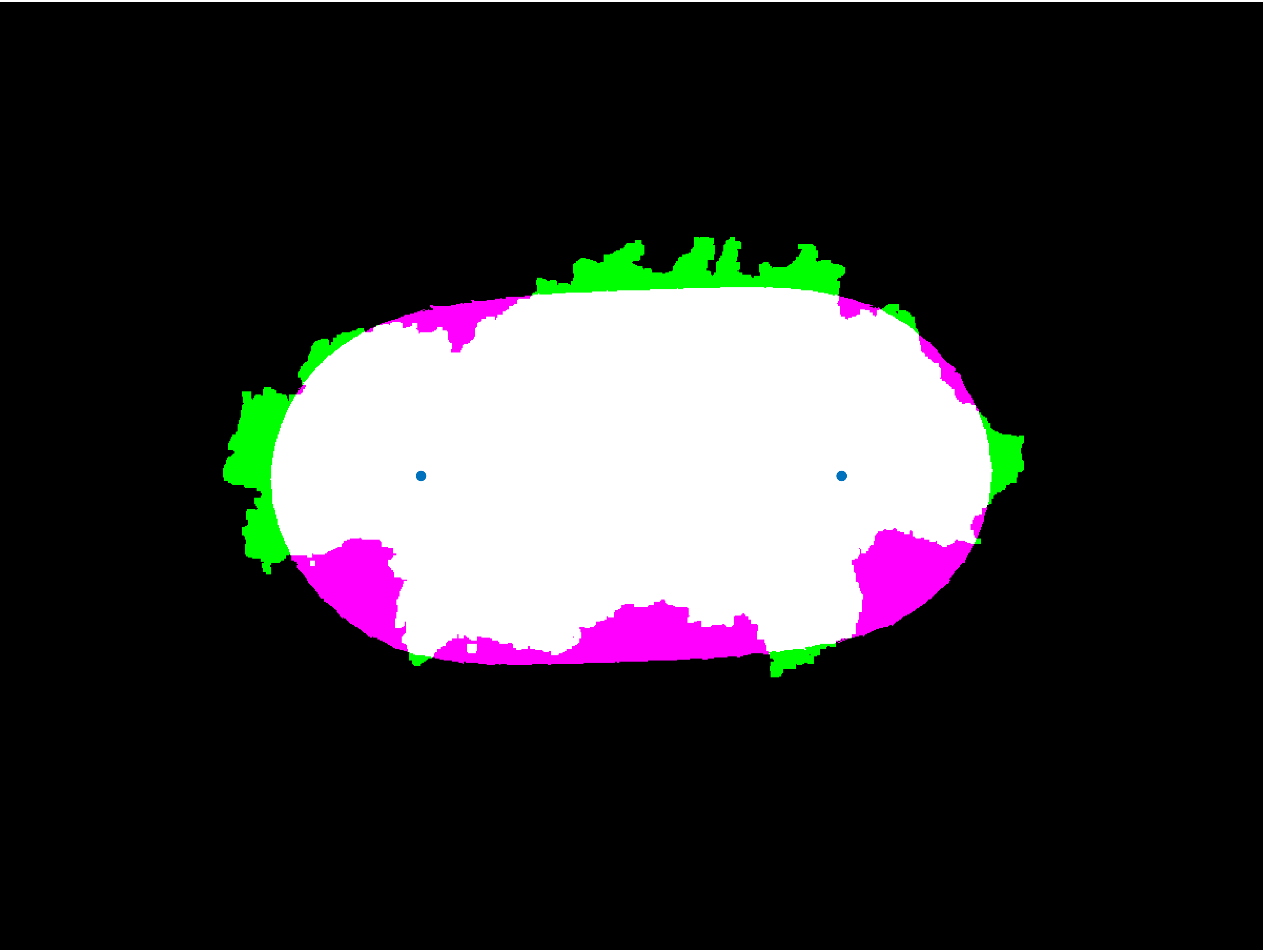

Lesion 14, Voltage=500, Angle=22.7, LET= 549, AR=1.75, EF=2.84, Dice=0.788

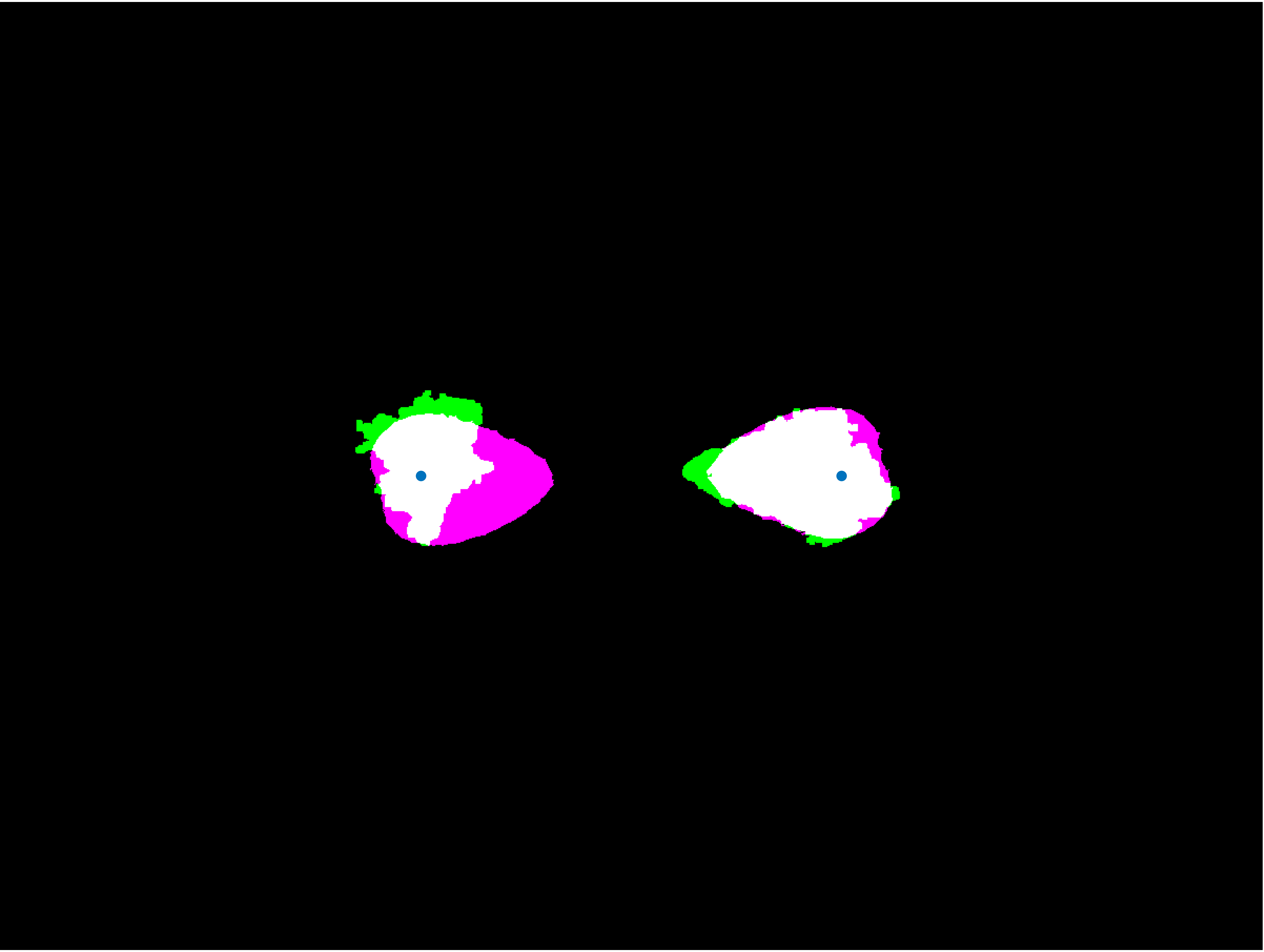

Lesion 15, Voltage=700, Angle=-41.1, LET= 486, AR=3.52, EF=9.81, Dice=0.885

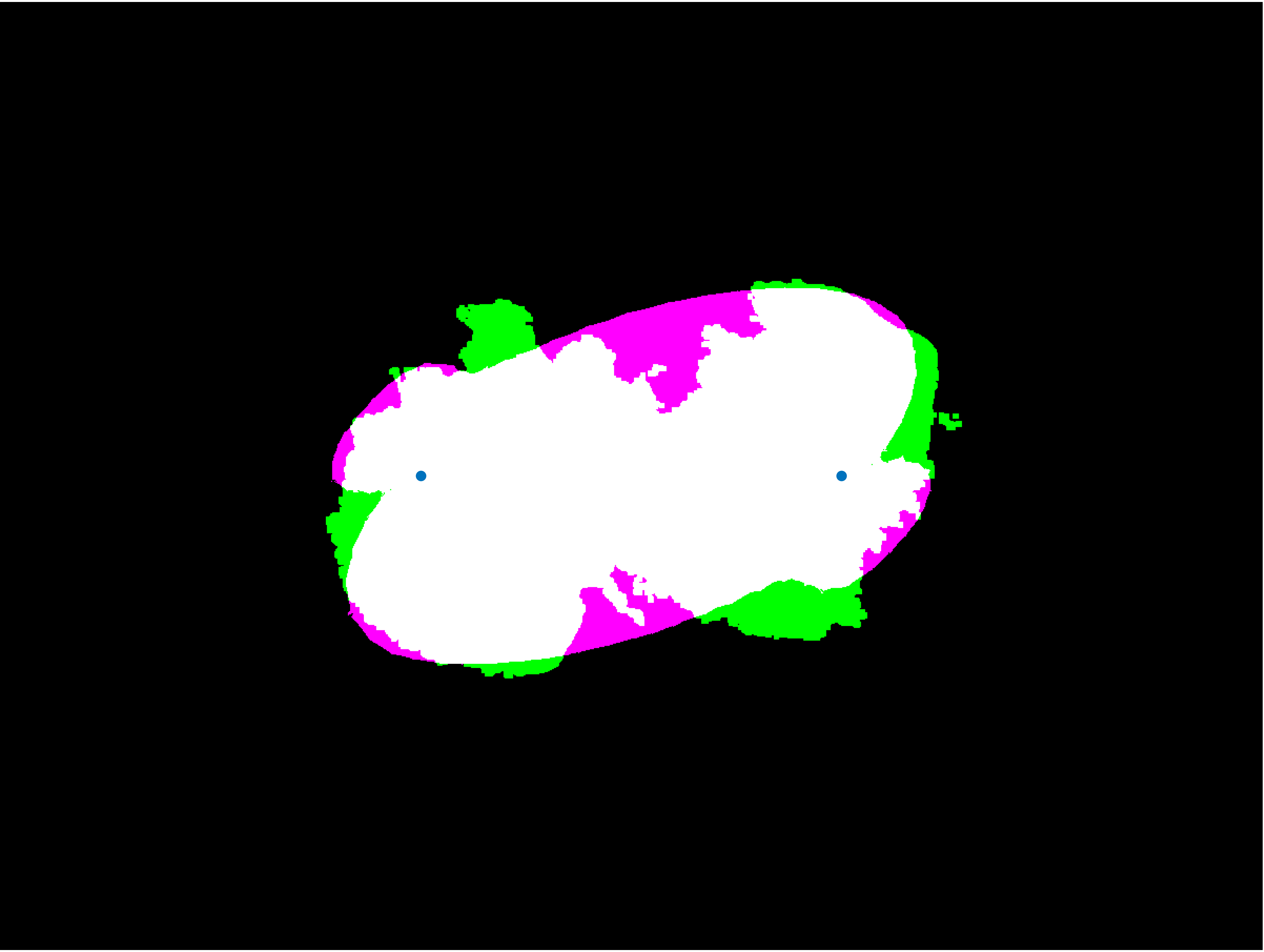

Lesion 16, Voltage=1000, Angle=86.6, LET= 527, AR=1.33, EF=2.22, Dice=0.858

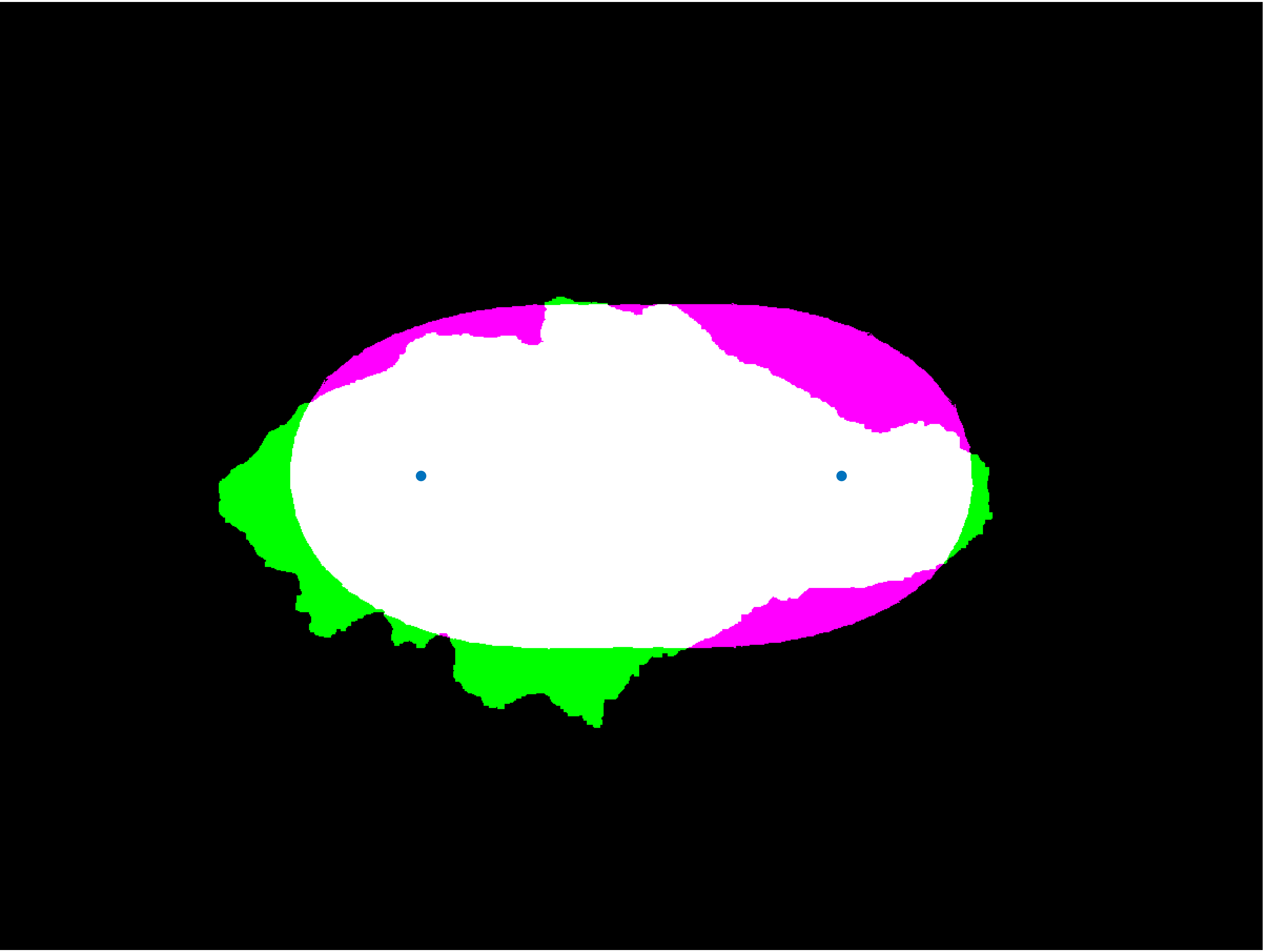

Lesion 17, Voltage=1100, Angle=71.6, LET= 501, AR=1.83, EF=7.26, Dice=0.787

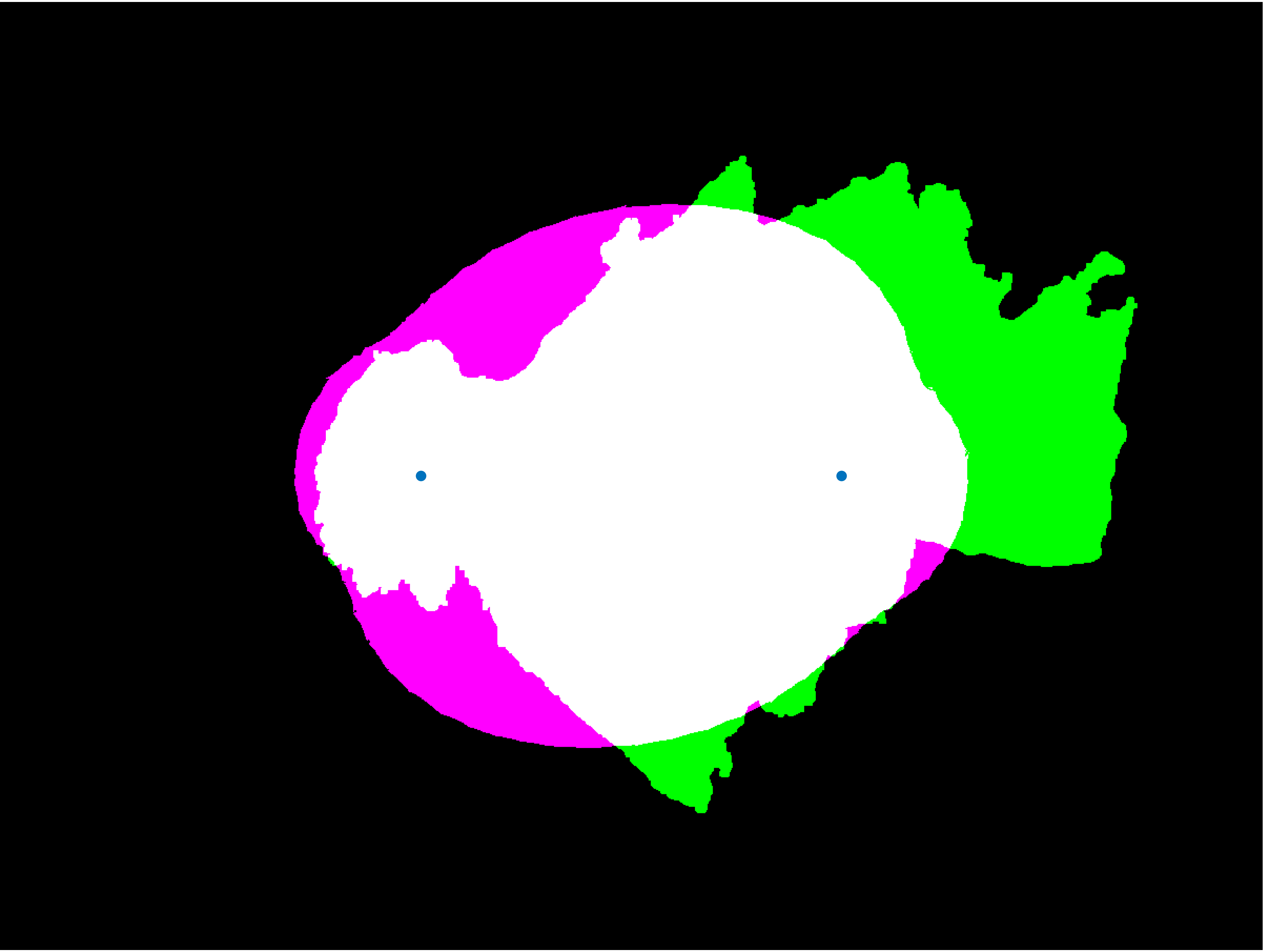

Lesion 18, Voltage=1200, Angle=-74.1, LET= 434, AR=1.00, EF=8.38, Dice=0.887

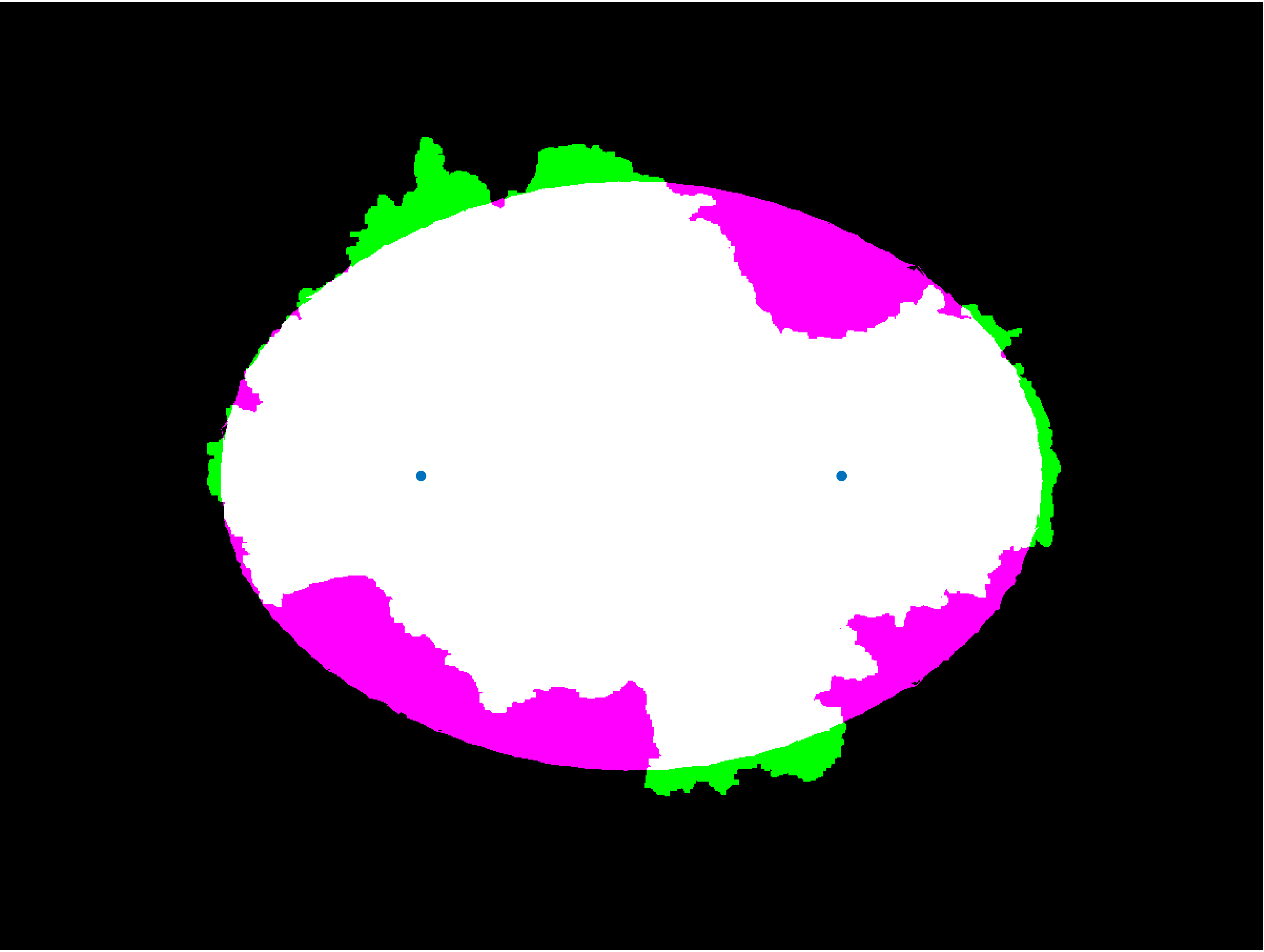

Lesion 19, Voltage=1200, Angle=-35.5, LET= 453, AR=3.23, EF=4.32, Dice=0.881

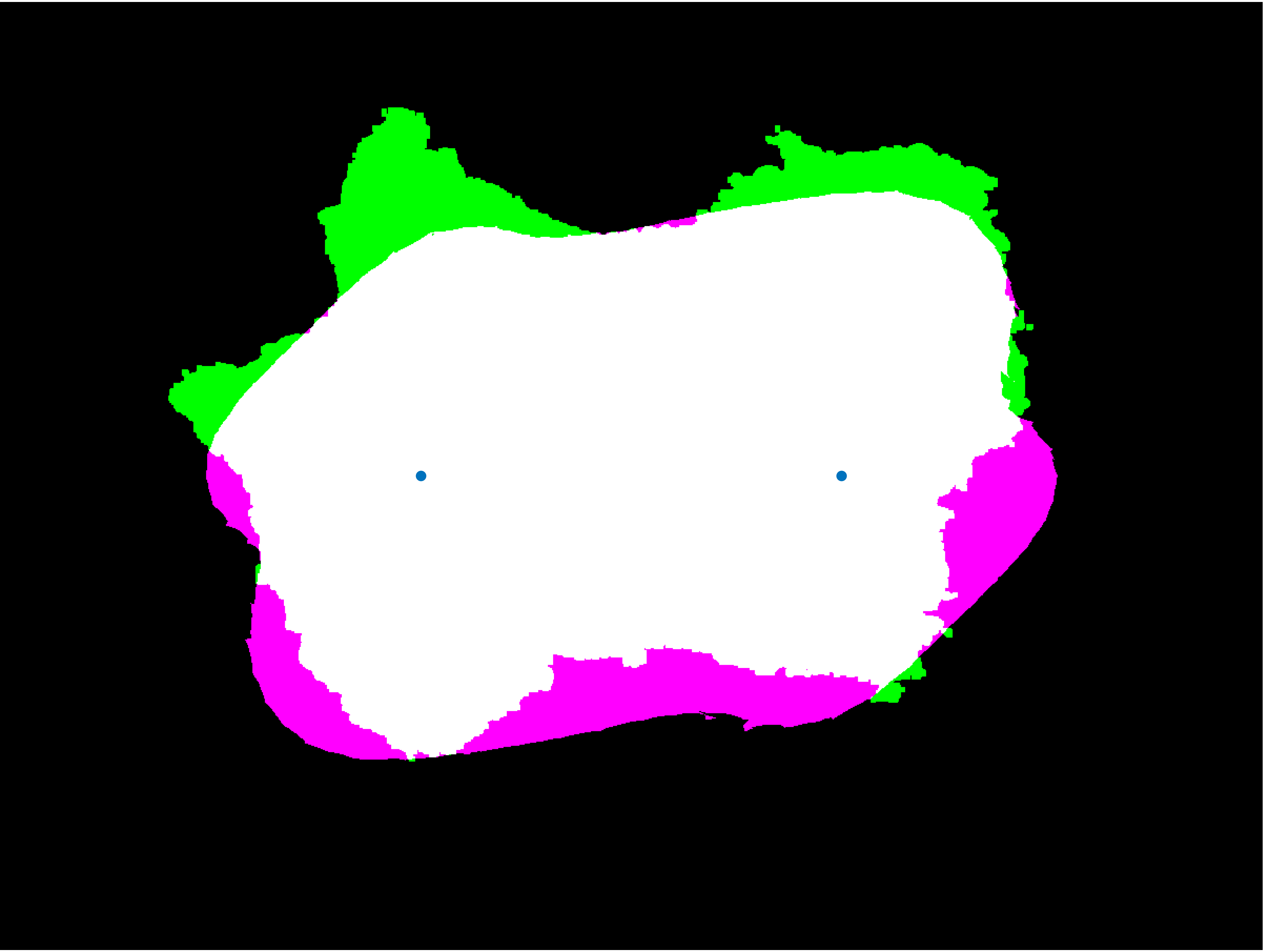

Lesion 20, Voltage=1000, Angle=4.8, LET= 727, AR=1.24, EF=3.14, Dice=0.879

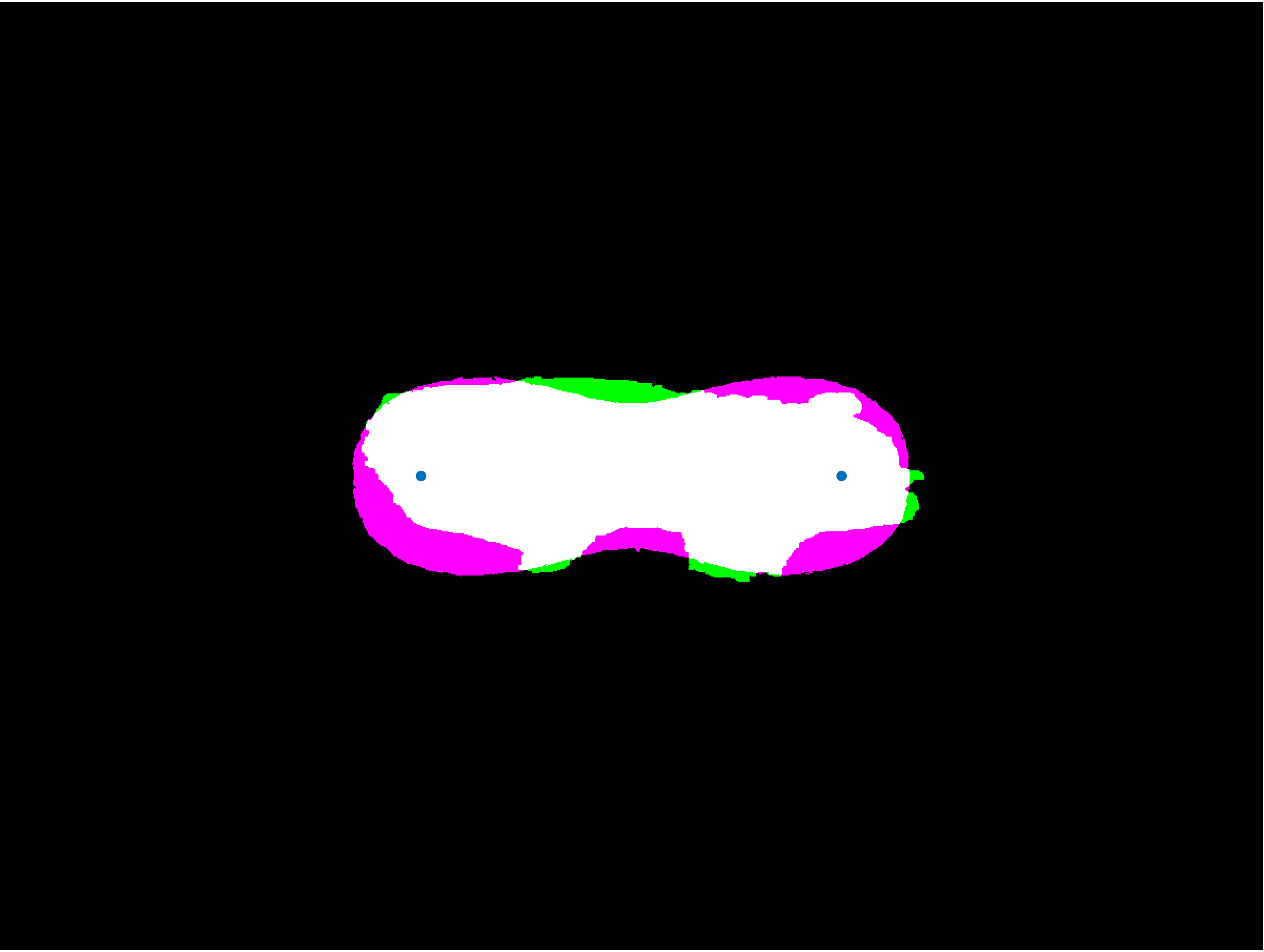

Lesion 21, Voltage=1200, Angle=13.3, LET= 464, AR=3.67, EF=1.00, Dice=0.831

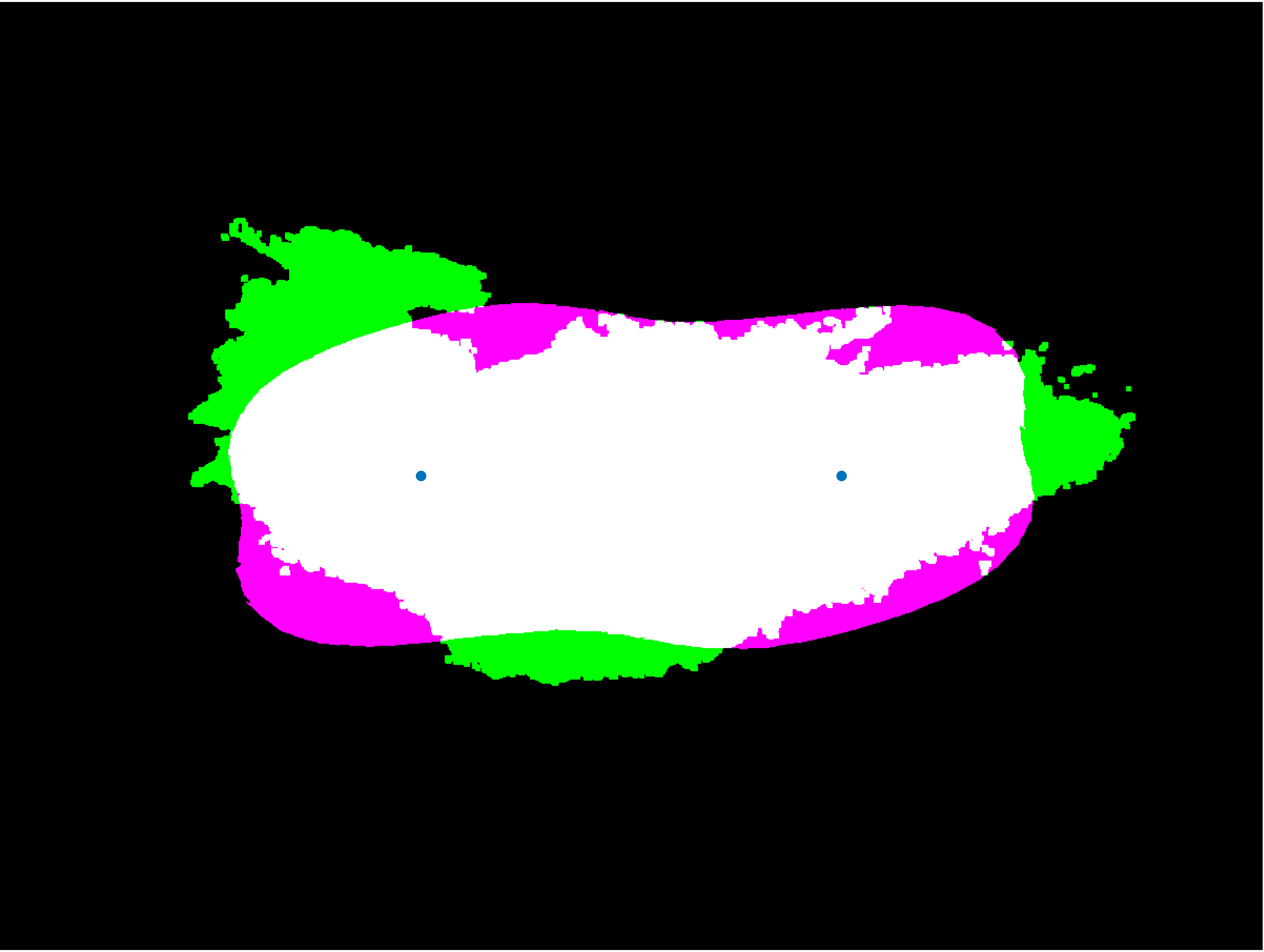

Lesion 22, Voltage=500, Angle=-2.3, LET= 408, AR=1.03, EF=1.00, Dice=0.811

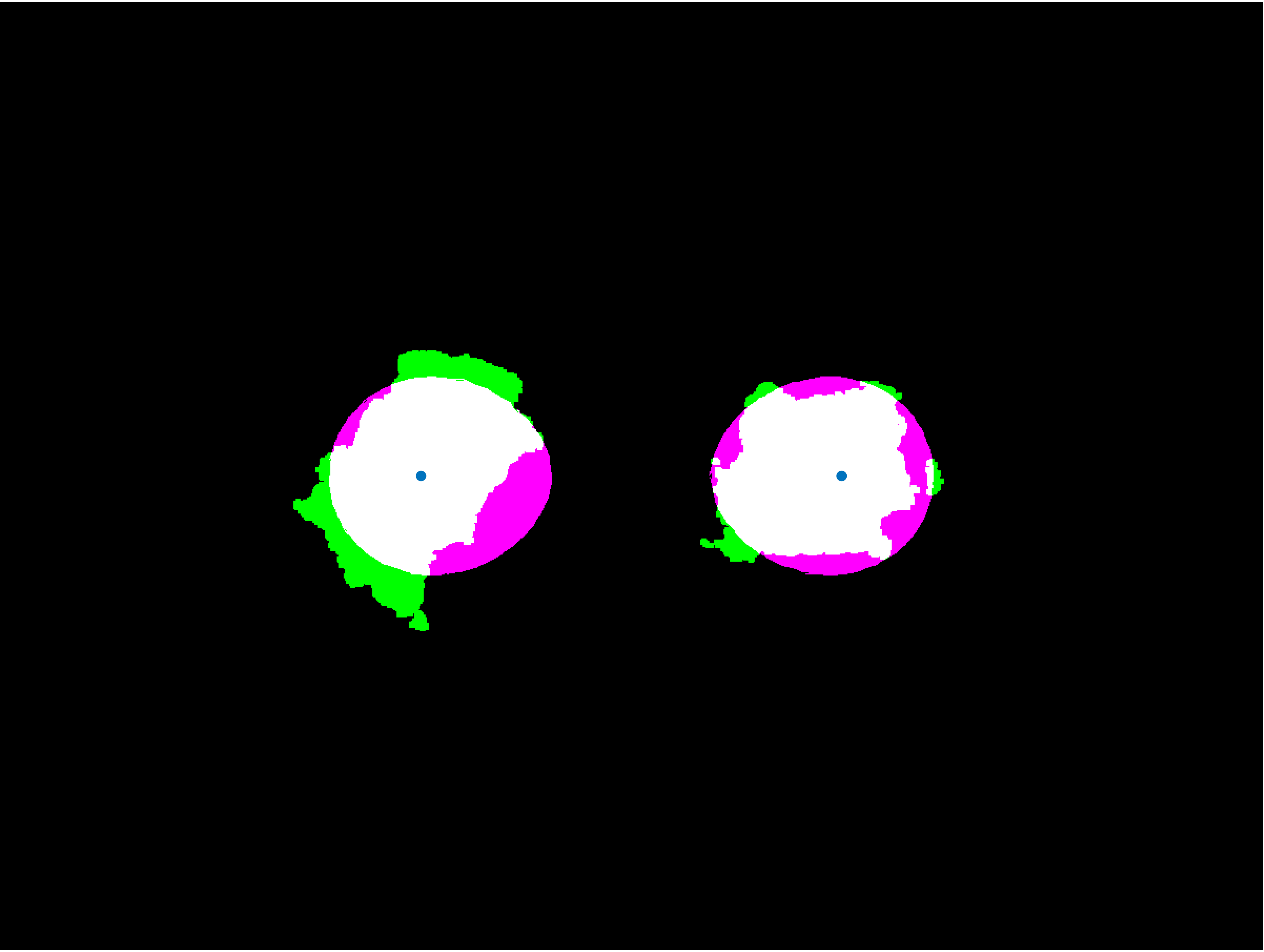

Lesion 23, Voltage=700, Angle=-17.7, LET= 511, AR=1.00, EF=1.88, Dice=0.889

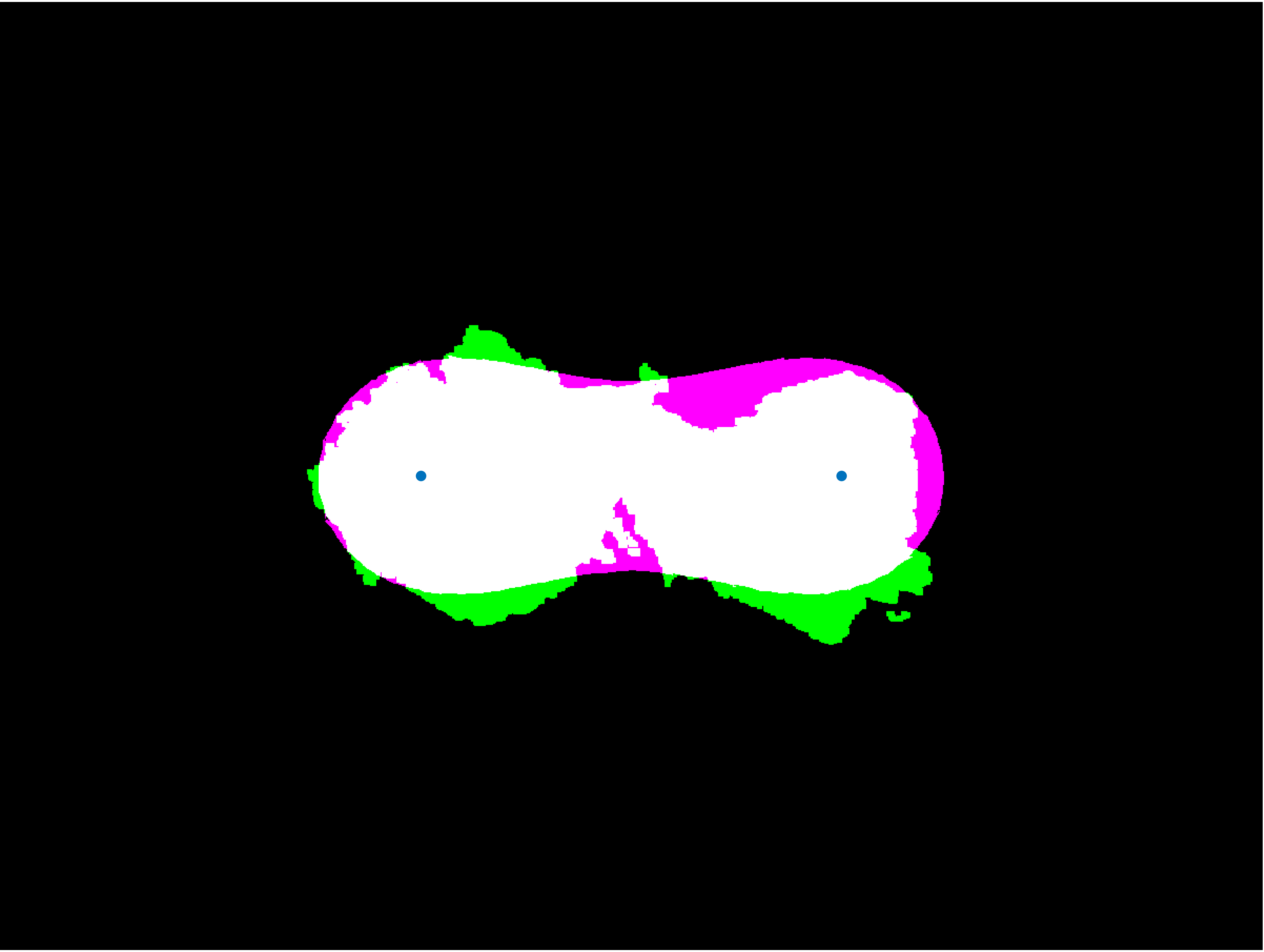

Lesion 24, Voltage=1000, Angle=55.9, LET= 437, AR=3.29, EF=1.29, Dice=0.822

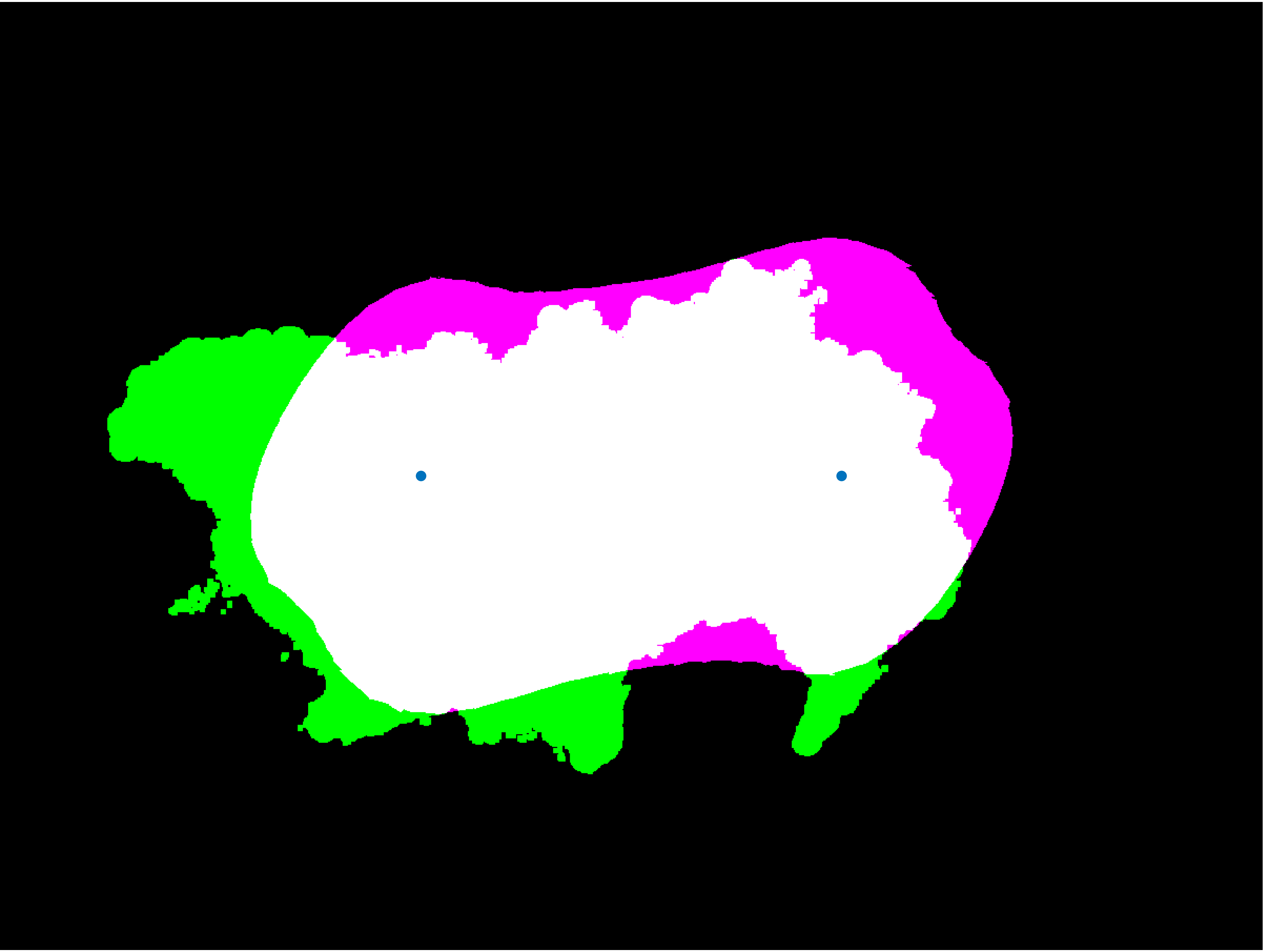

Lesion 25, Voltage=1100, Angle=-73.2, LET= 498, AR=1.06, EF=2.20, Dice=0.869

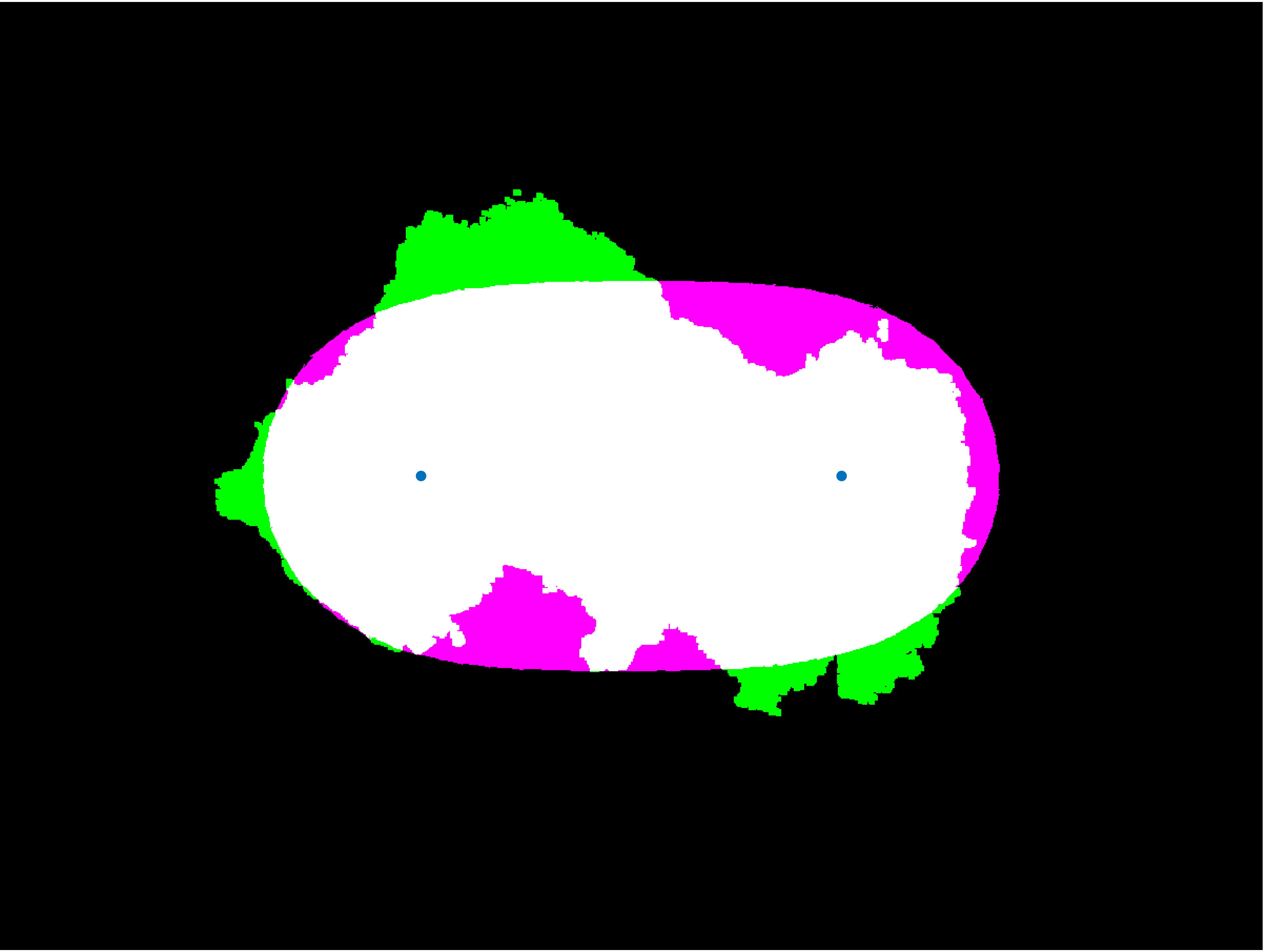

Lesion 26, Voltage=1200, Angle=-56.3, LET= 358, AR=2.17, EF=2.20, Dice=0.870

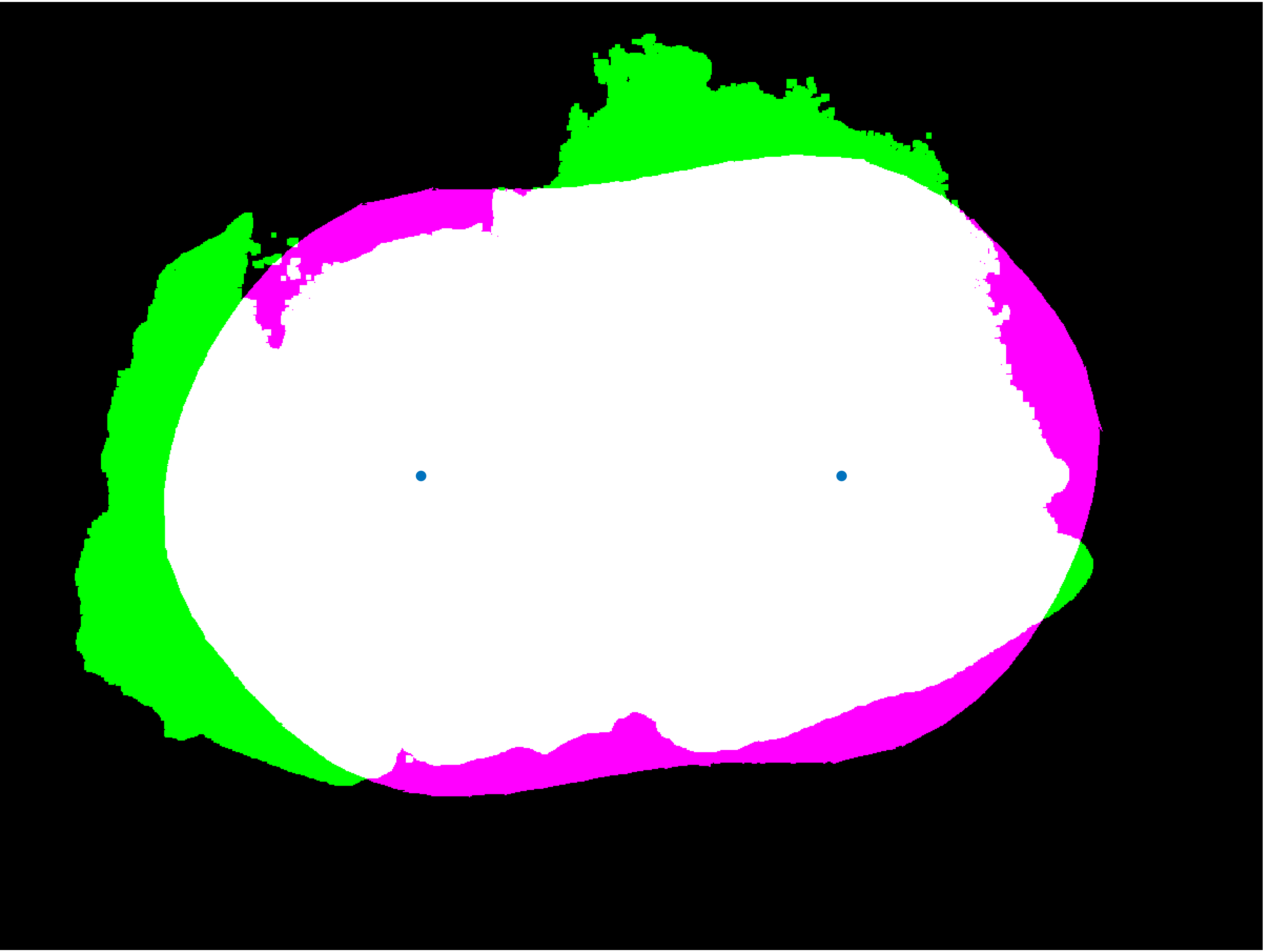

Lesion 27, Voltage=1200, Angle=49.8, LET= 507, AR=1.75, EF=4.06, Dice=0.911

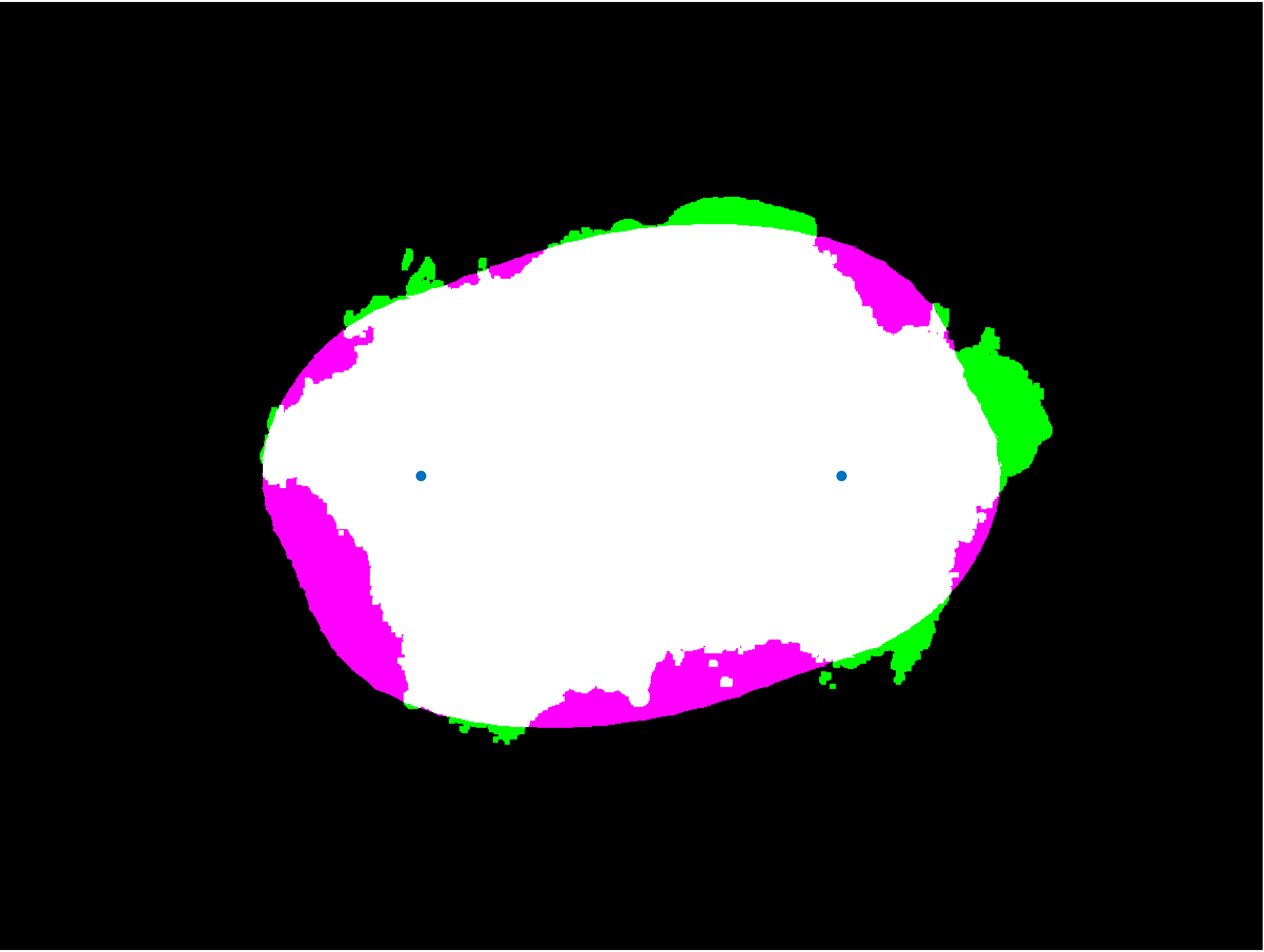

Lesion 28, Voltage=1200, Angle=25.8, LET= 559, AR=1.59, EF=3.03, Dice=0.839

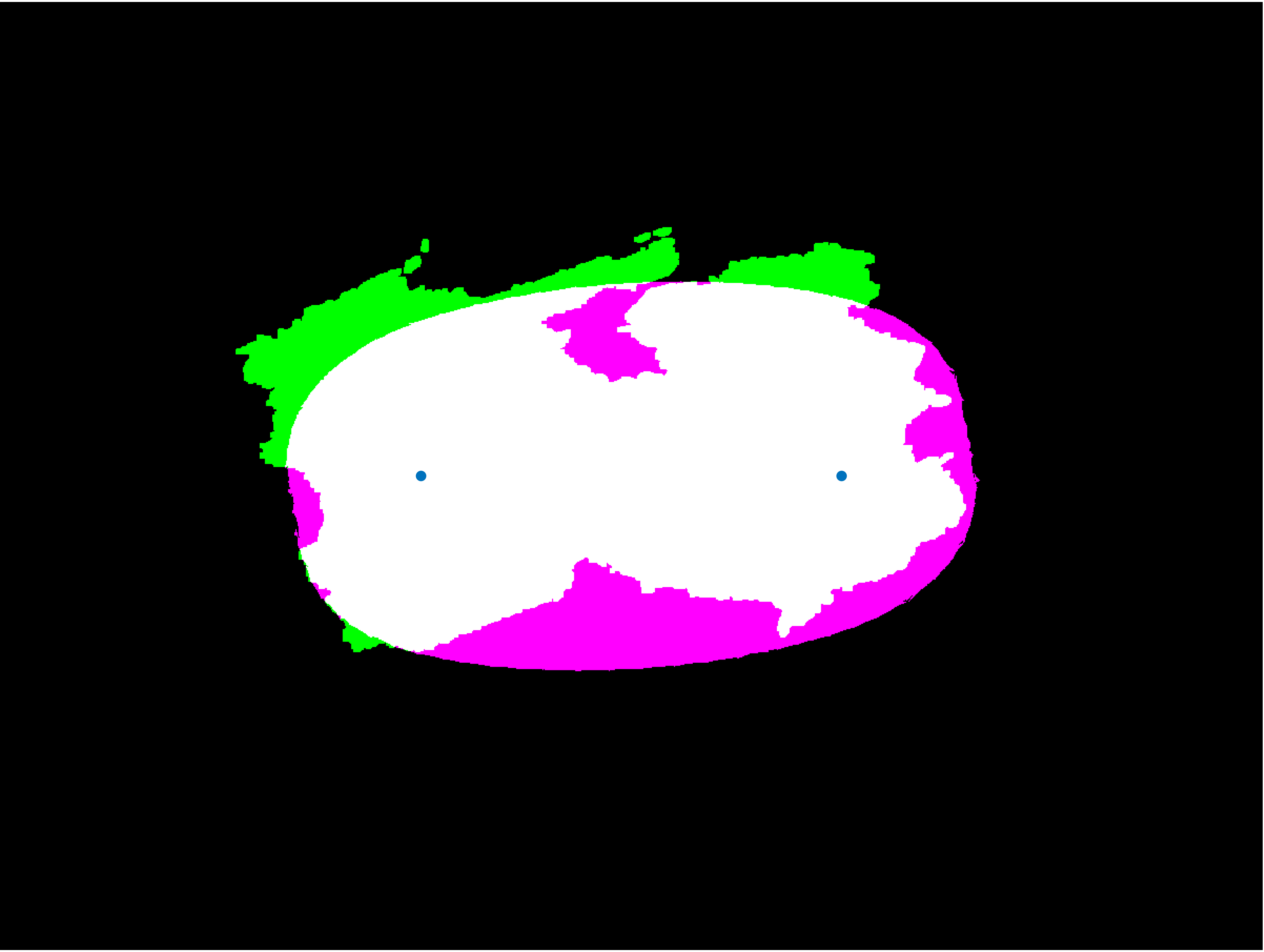

Lesion 29, Voltage=1200, Angle=-77.8, LET= 500, AR=1.06, EF=4.00, Dice=0.896

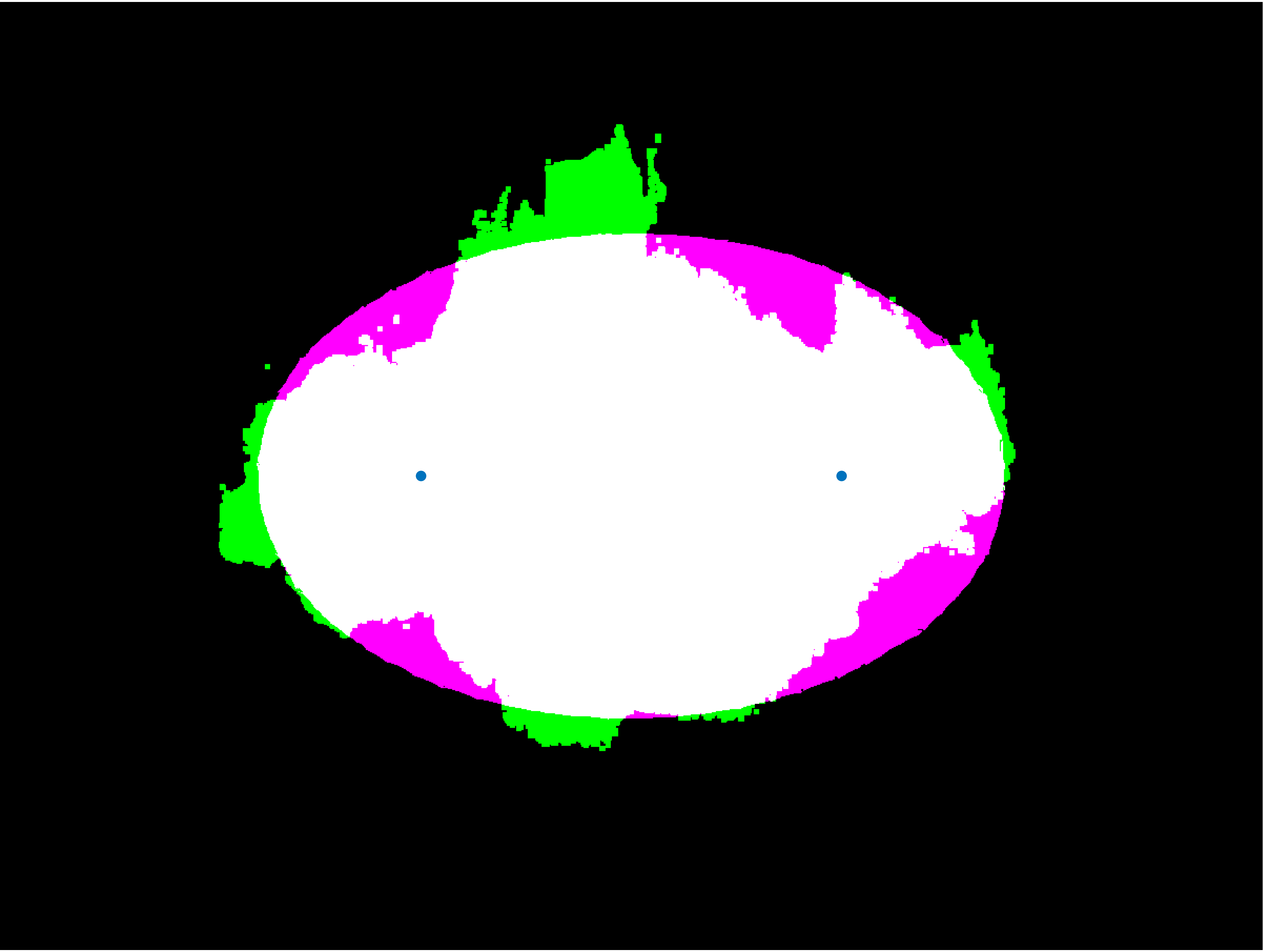

Lesion 30, Voltage=1300, Angle=51.2, LET= 646, AR=2.38, EF=2.65, Dice=0.879

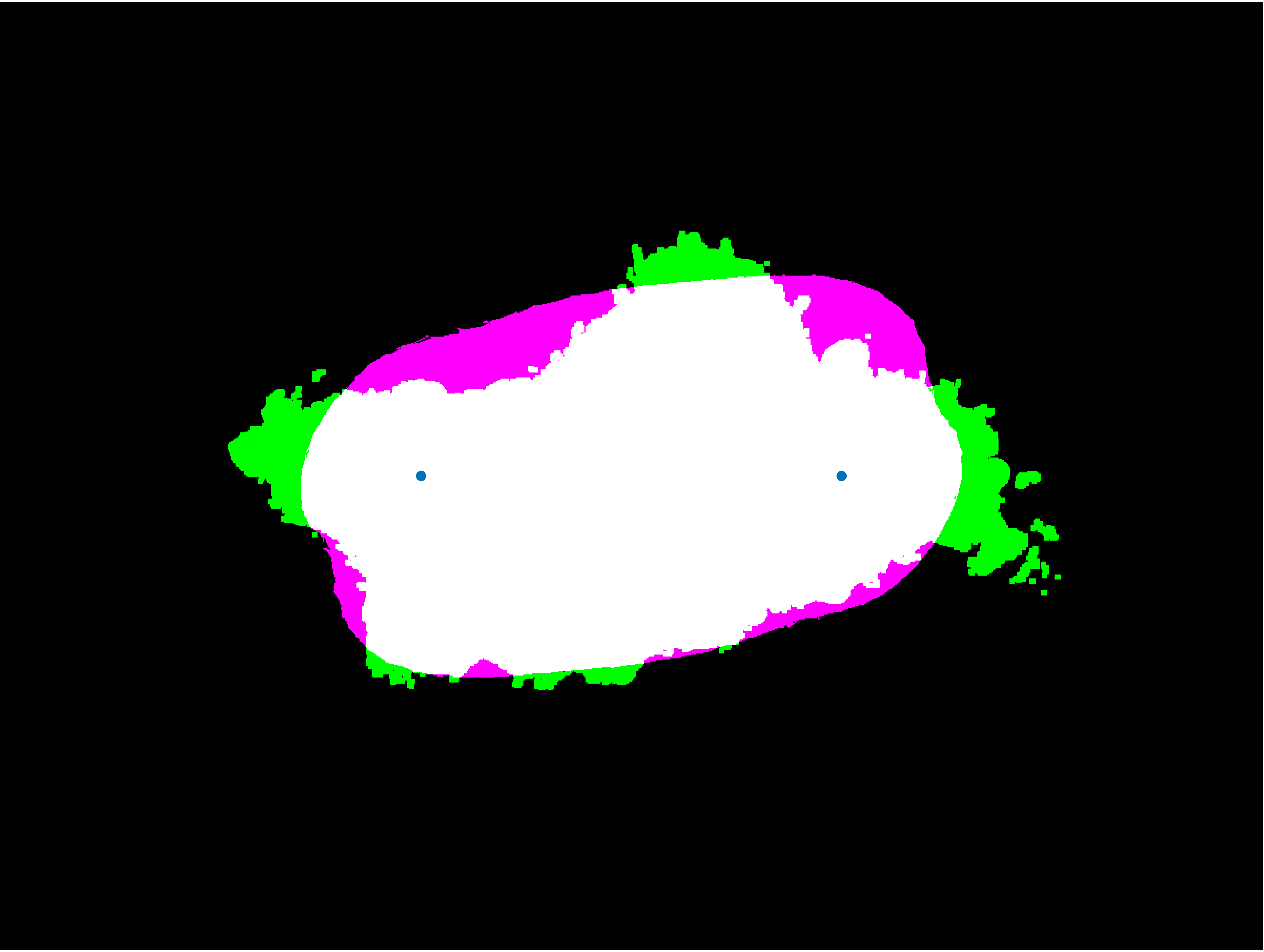

Lesion 31, Voltage=1200, Angle=-89.2, LET= 588, AR=1.33, EF=2.70, Dice=0.914

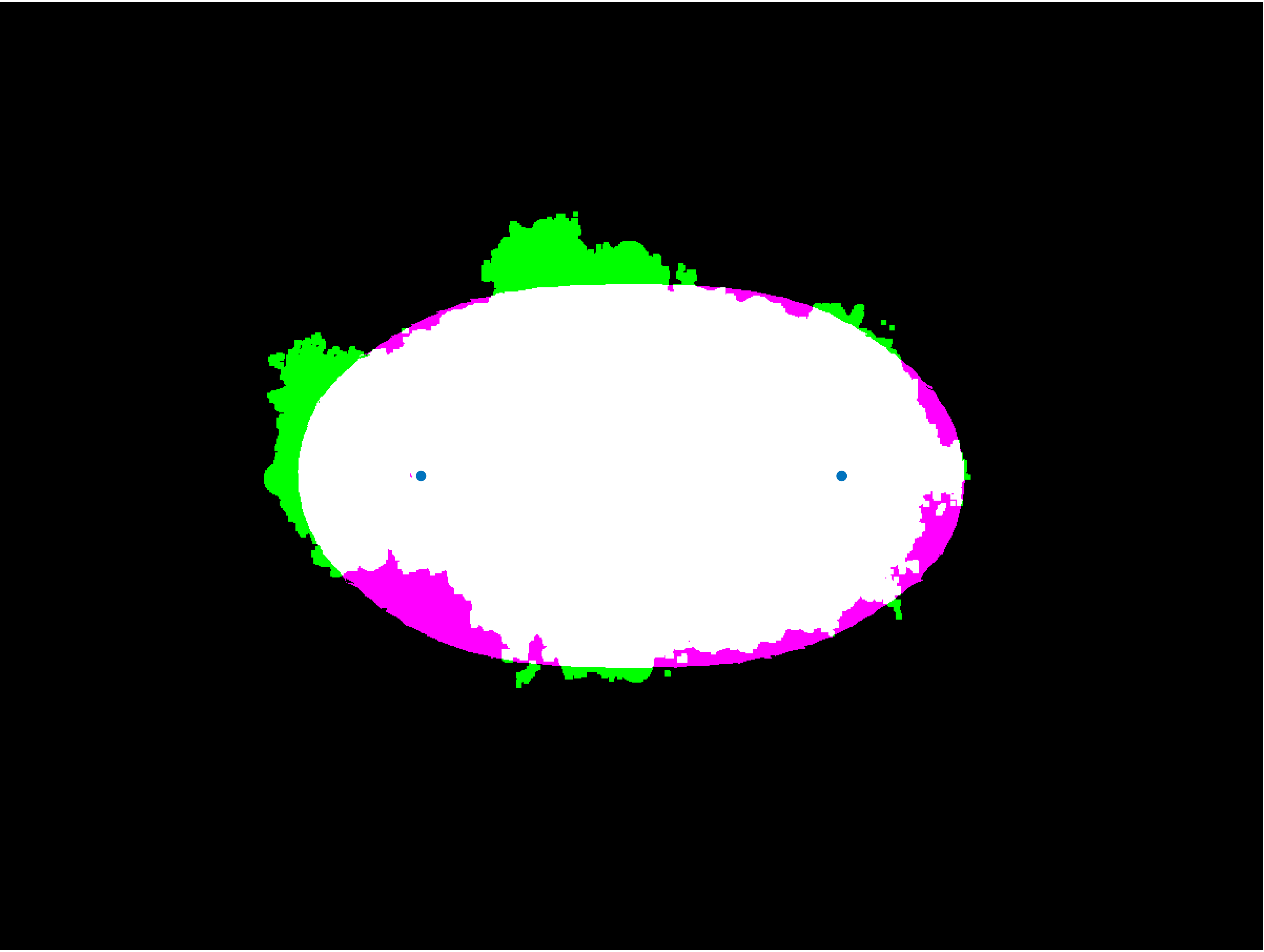

Lesion 32, Voltage=1300, Angle=-83.7, LET= 538, AR=1.43, EF=3.27, Dice=0.905

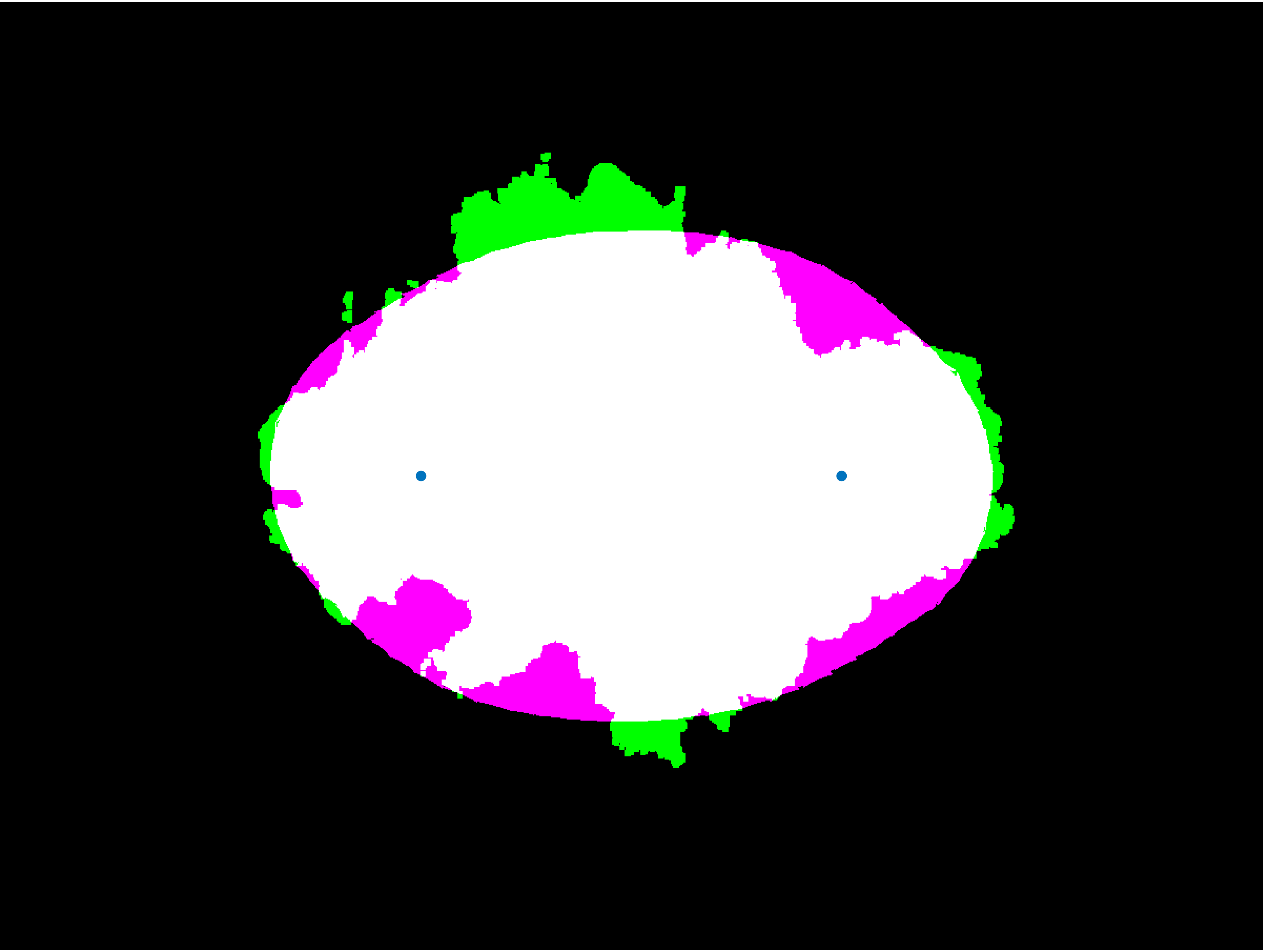

Lesion 33, Voltage=1200, Angle=87.4, LET= 543, AR=2.15, EF=3.18, Dice=0.913

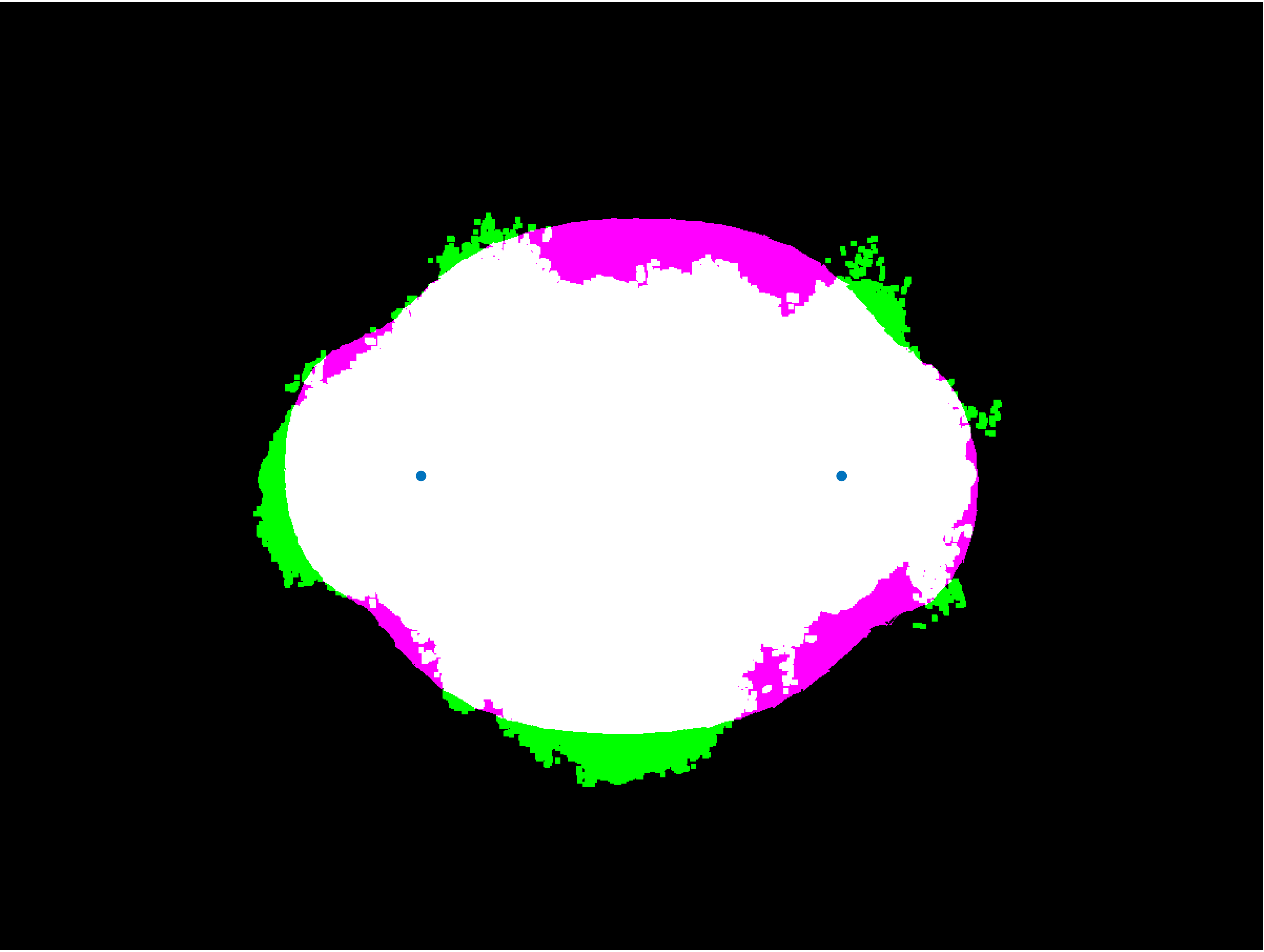

Lesion 34, Voltage=1300, Angle=42.7, LET= 546, AR=1.06, EF=2.28, Dice=0.905

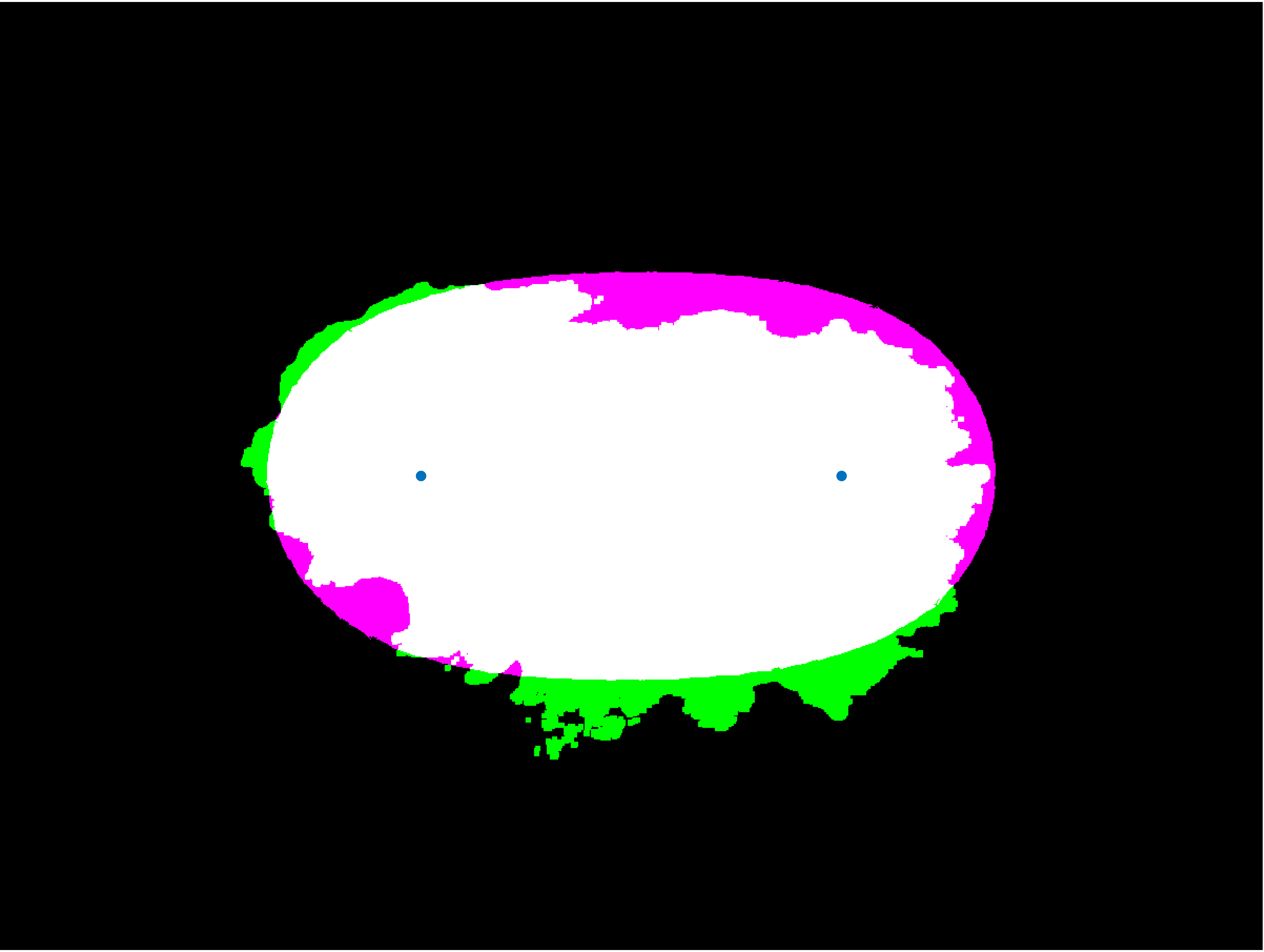

Lesion 35, Voltage=1200, Angle=-4.7, LET= 558, AR=1.39, EF=3.07, Dice=0.878

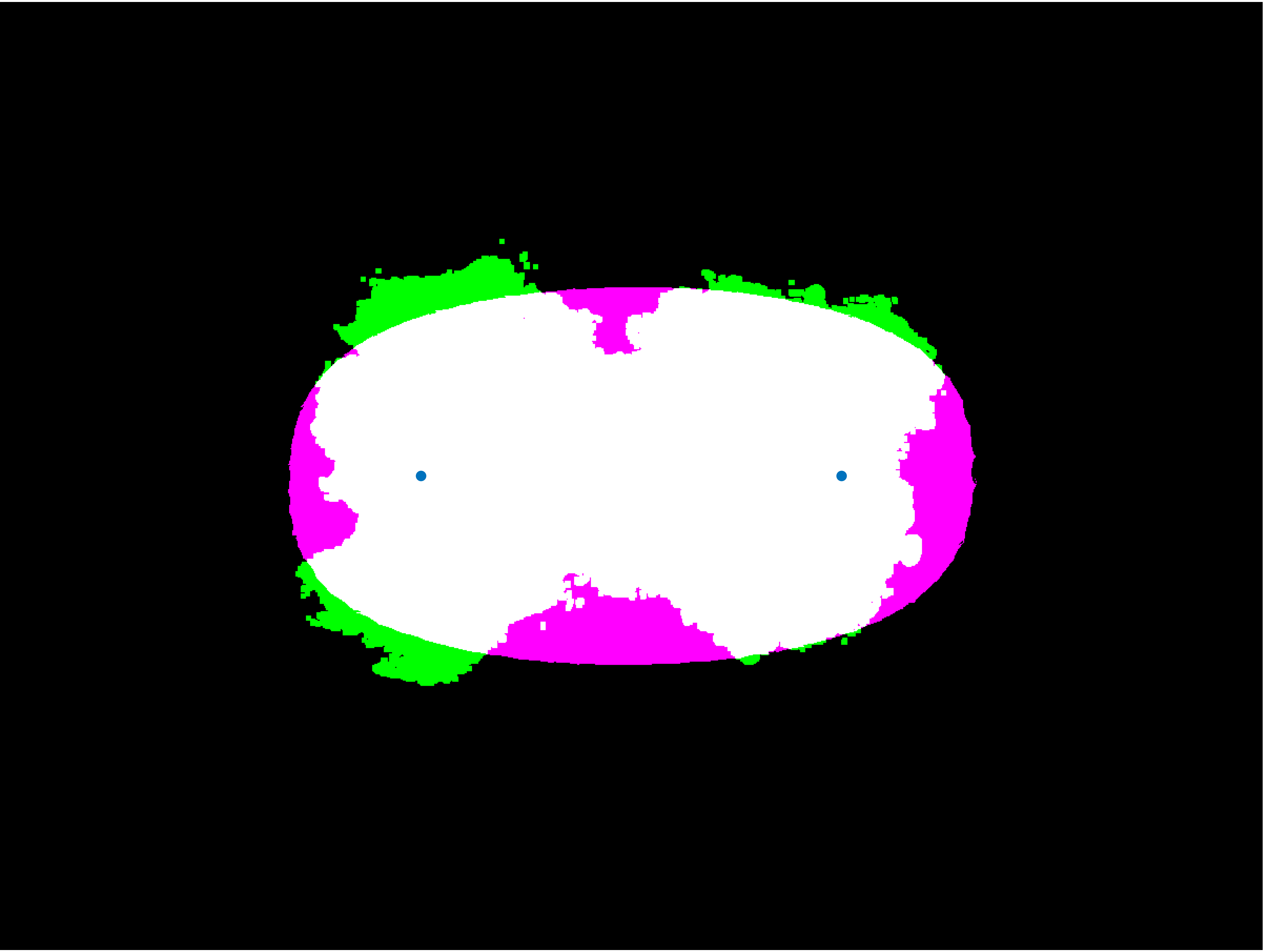

Lesion 36, Voltage=1300, Angle=6.9, LET= 503, AR=1.06, EF=3.11, Dice=0.875

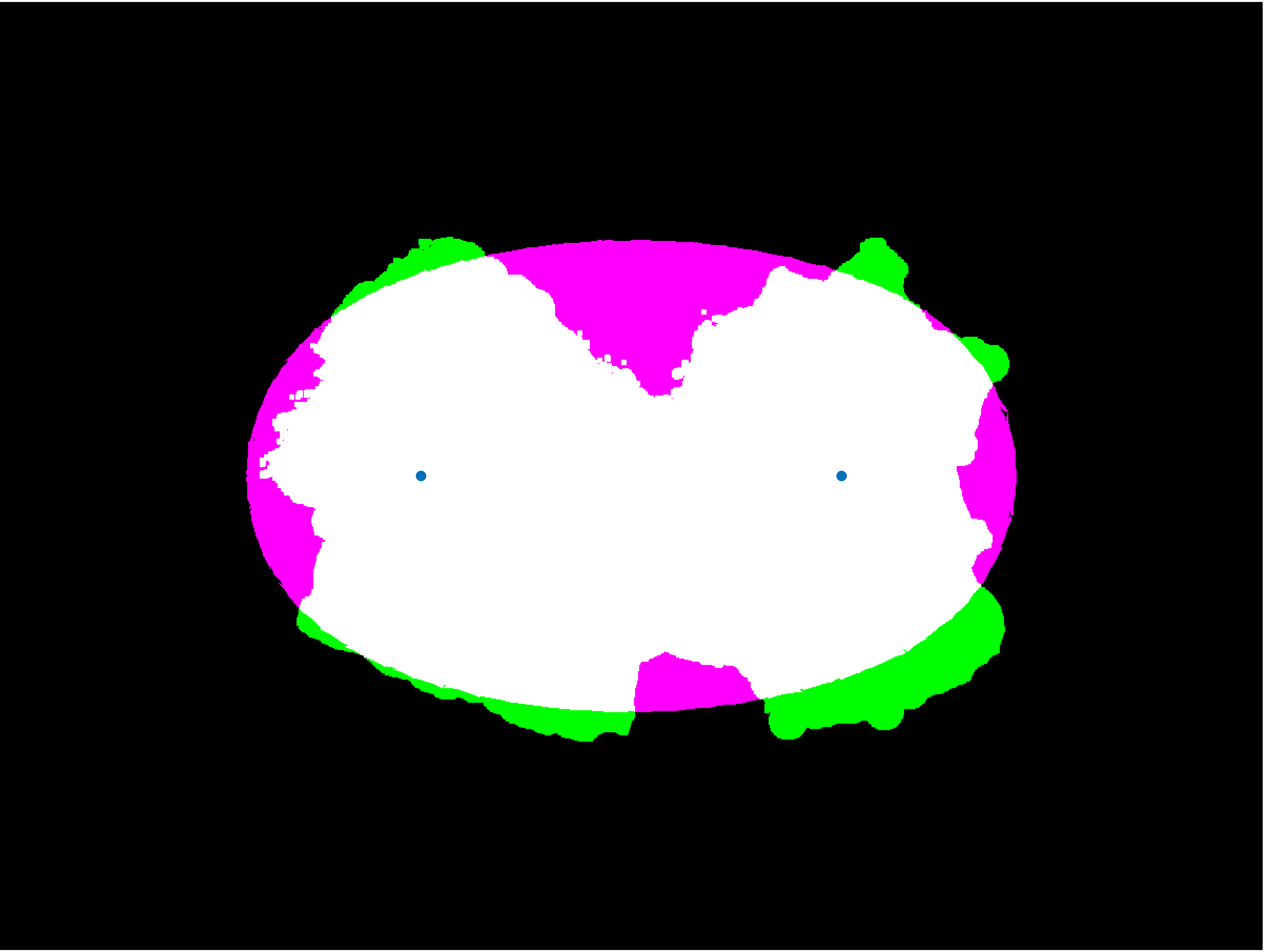

Lesion 37, Voltage=1200, Angle=-79.3, LET= 604, AR=1.06, EF=2.20, Dice=0.821

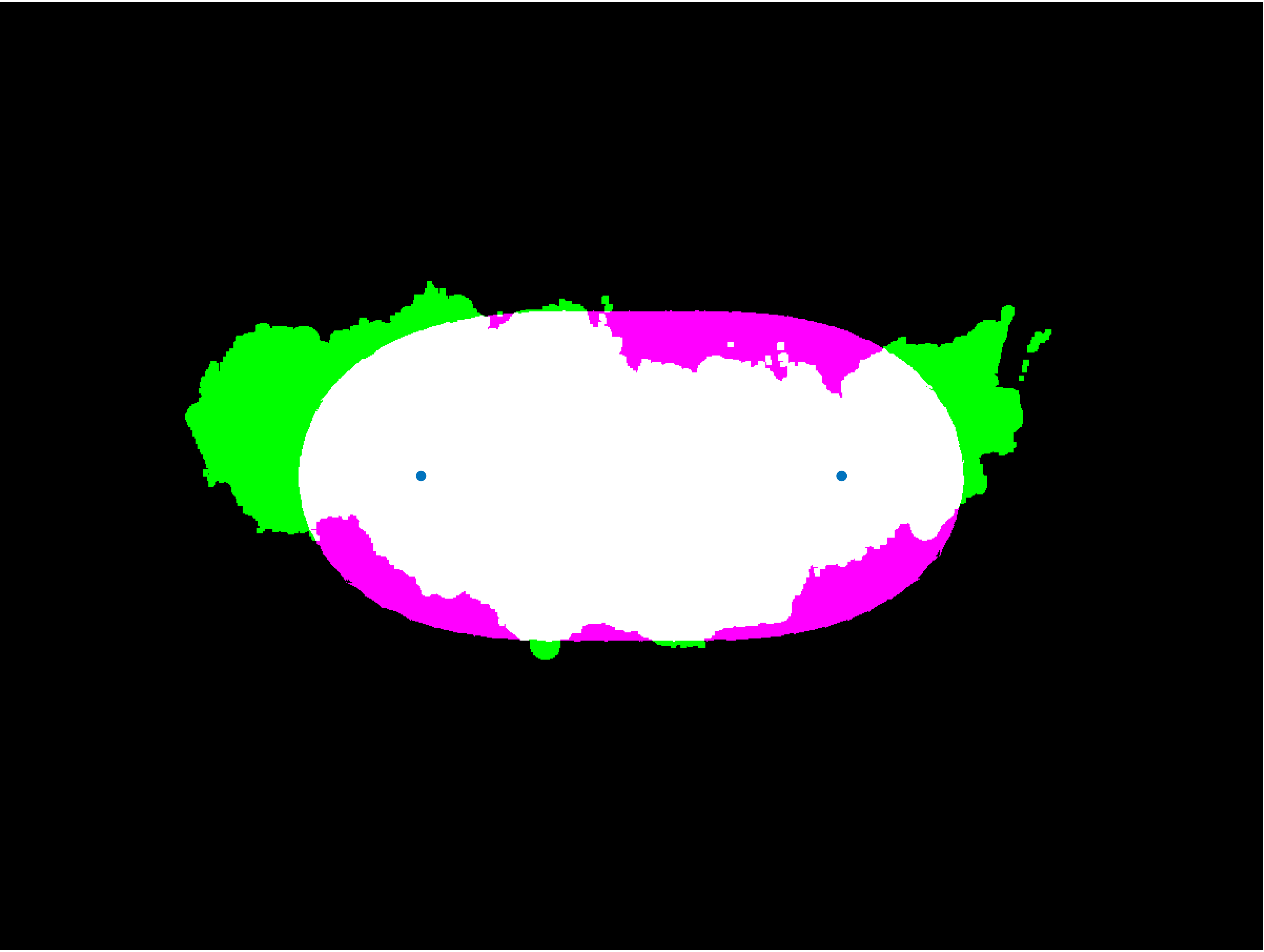

Lesion 38, Voltage=1200, Angle=-84.2, LET= 535, AR=1.04, EF=2.05, Dice=0.881

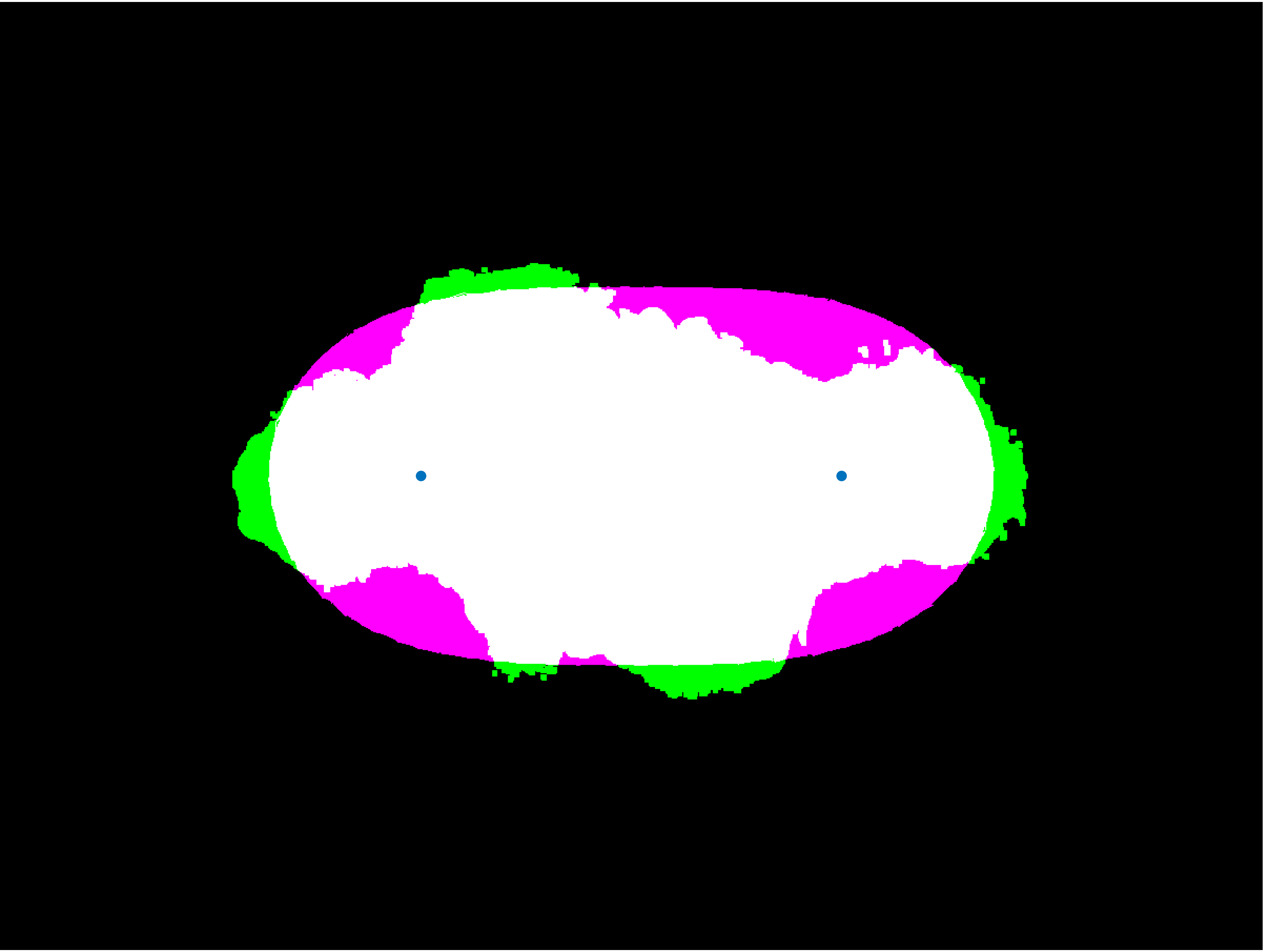

Lesion 39, Voltage=1300, Angle=-77.7, LET= 507, AR=1.01, EF=4.04, Dice=0.869

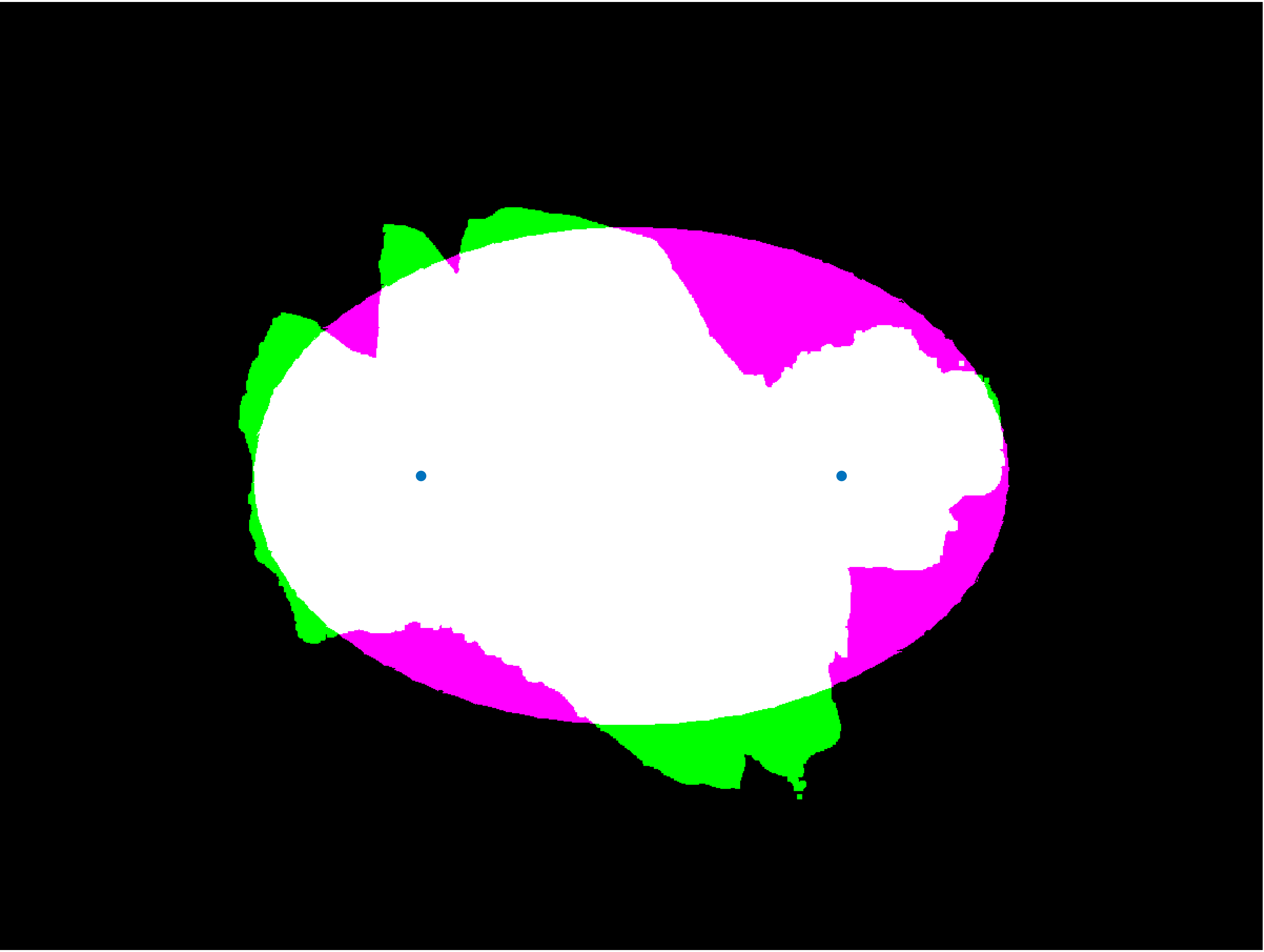

Lesion 40, Voltage=1300, Angle=-84.1, LET= 424, AR=1.43, EF=9.85, Dice=0.830

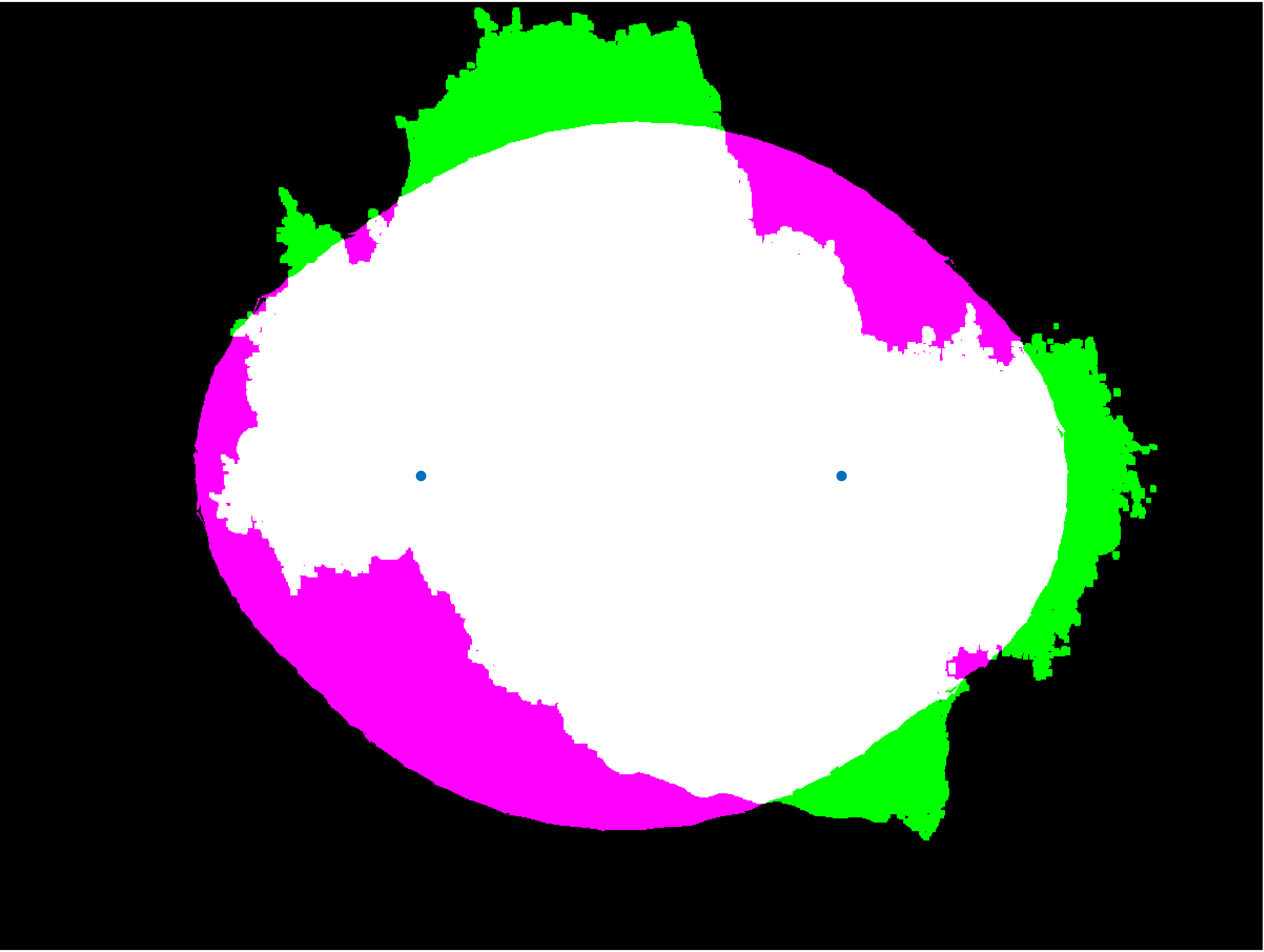

Lesion 41, Voltage=700, Angle=-44.9, LET= 513, AR=3.97, EF=1.07, Dice=0.862

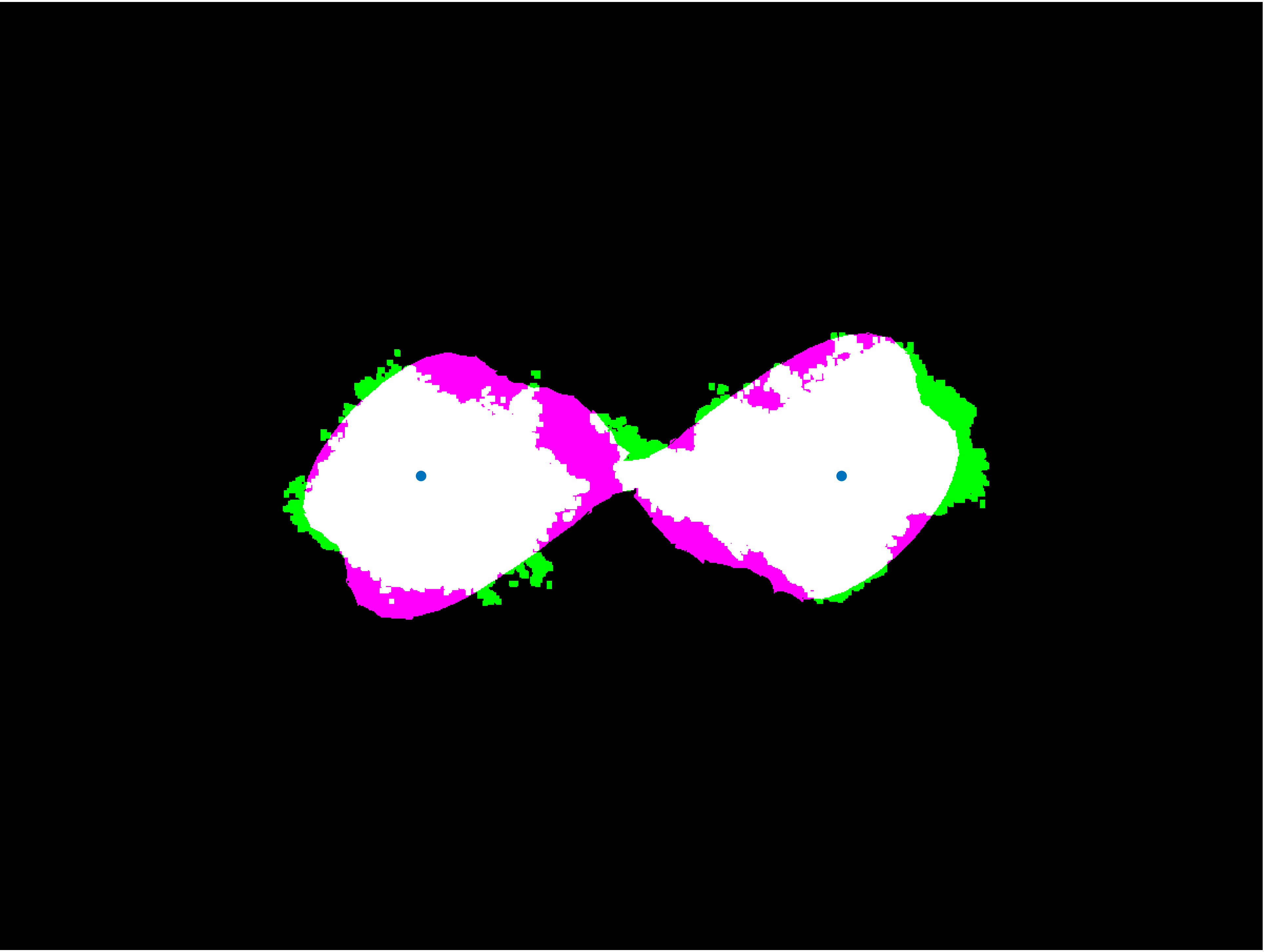

Lesion 42, Voltage=1200, Angle=-81.3, LET= 542, AR=1.09, EF=6.54, Dice=0.875

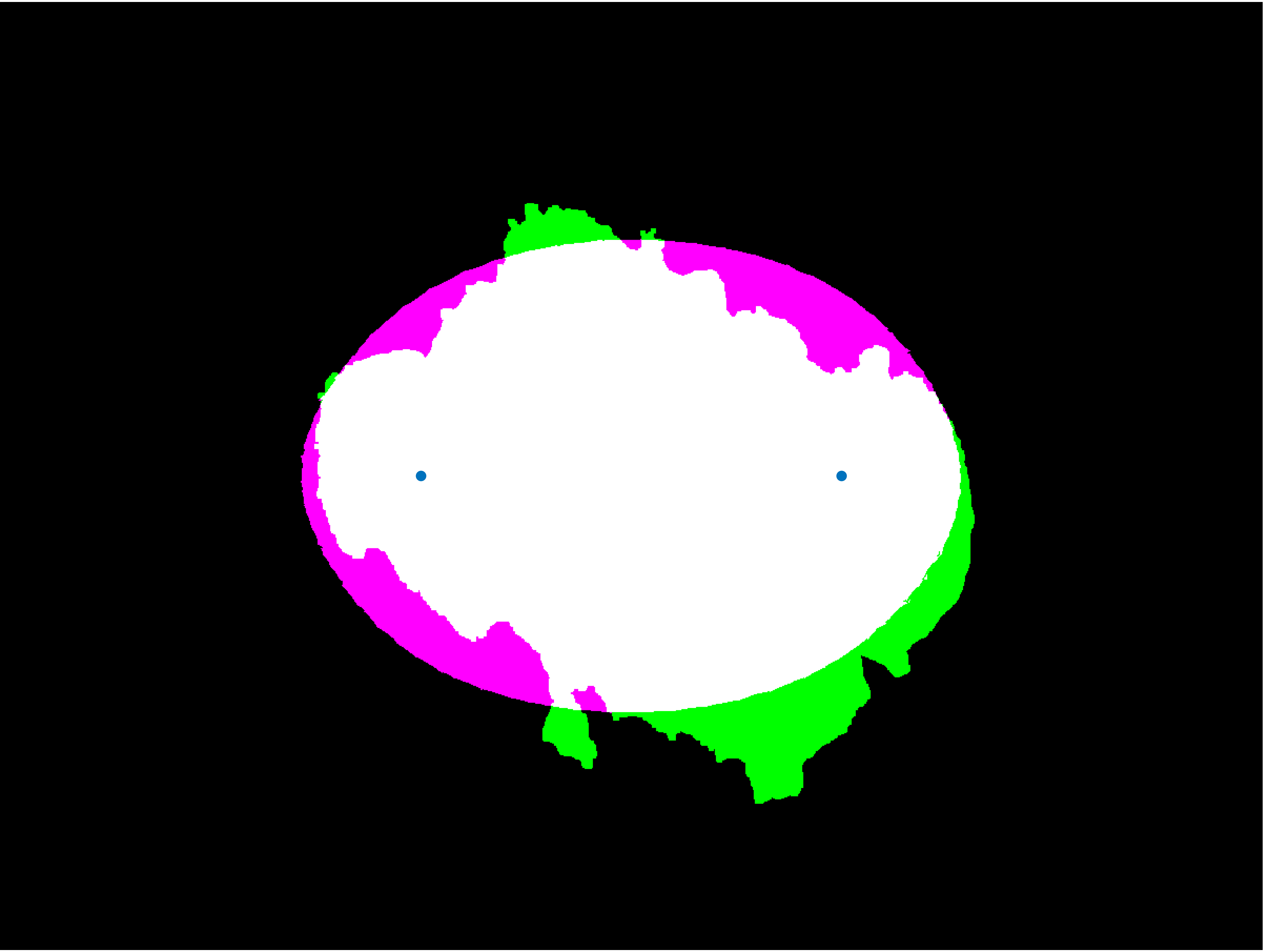

Lesion 43, Voltage=1000, Angle=-84.9, LET= 501, AR=1.02, EF=1.50, Dice=0.816

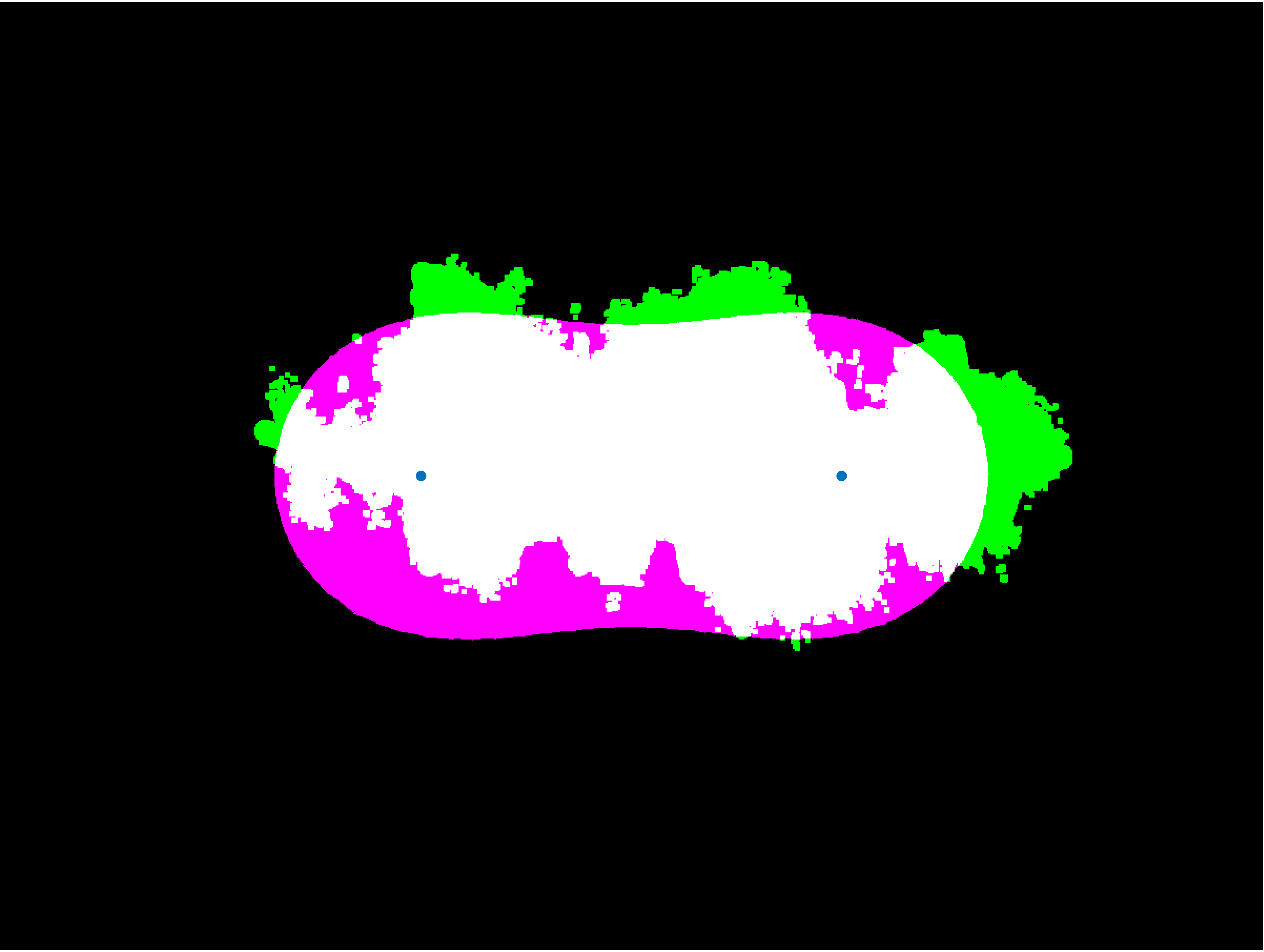

Lesion 44, Voltage=1200, Angle=2.0, LET= 712, AR=1.06, EF=3.19, Dice=0.893

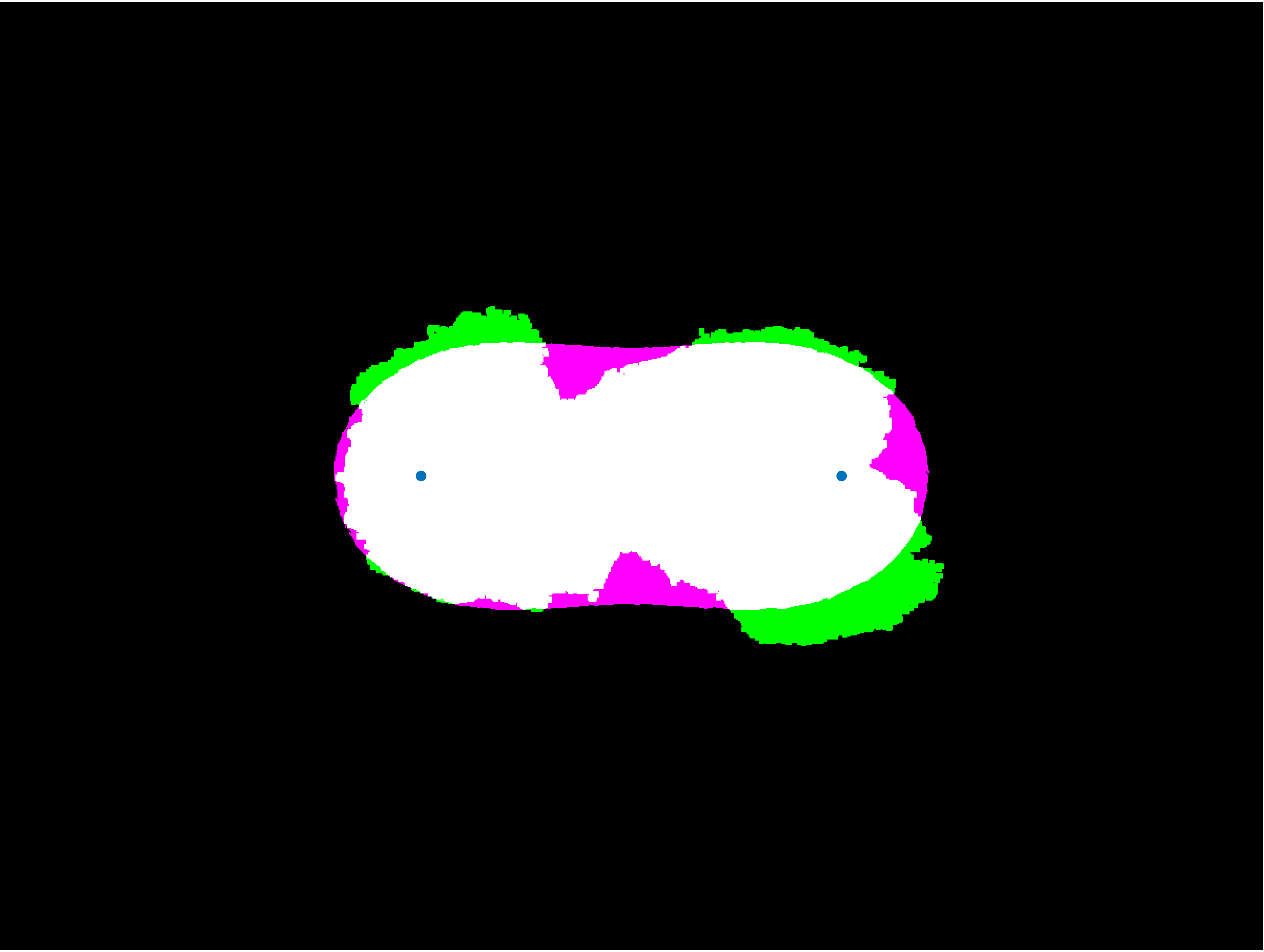

Lesion 45, Voltage=1200, Angle=8.5, LET= 802, AR=1.38, EF=1.38, Dice=0.845

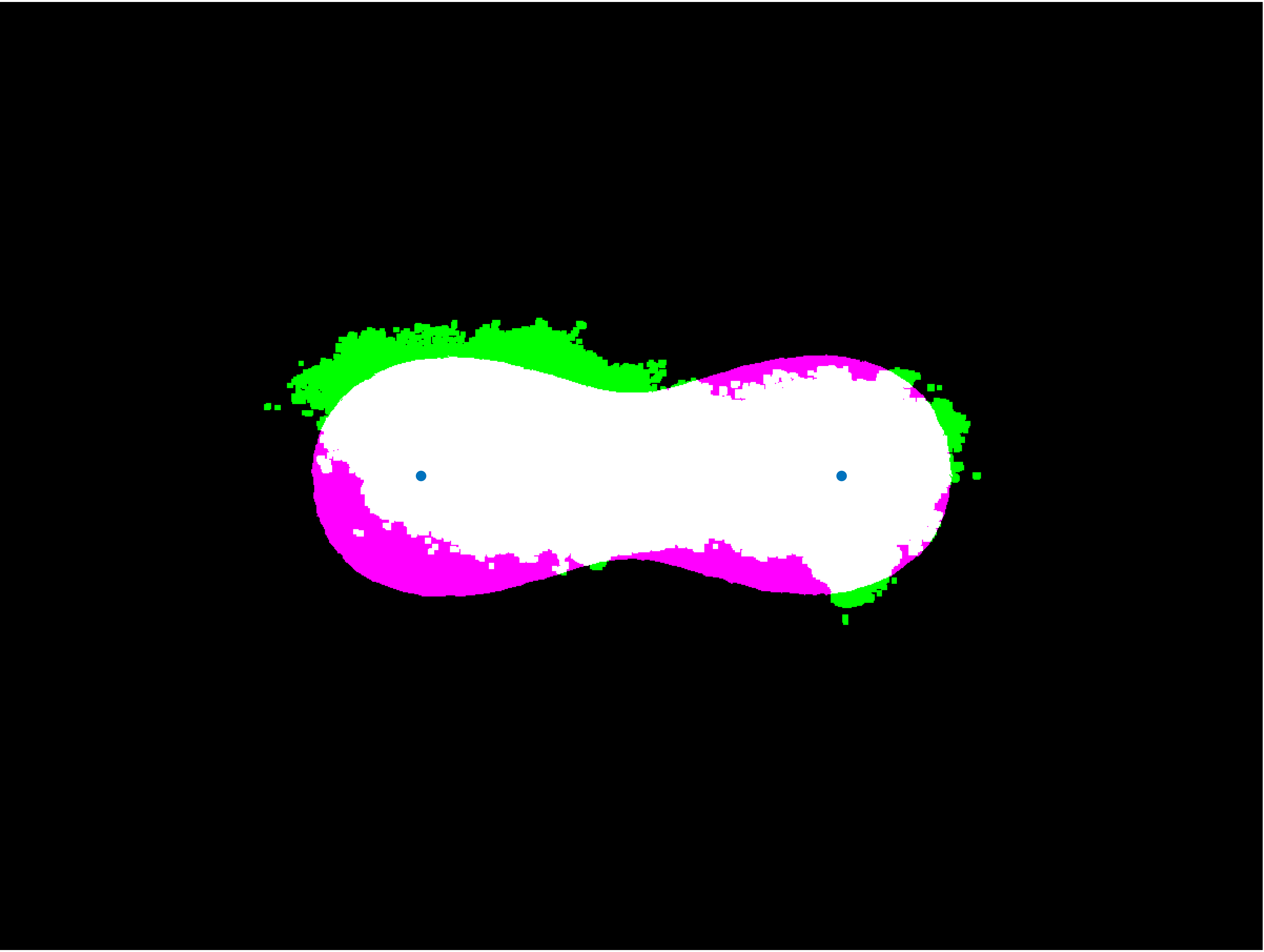

Lesion 46, Voltage=1300, Angle=-0.9, LET= 744, AR=1.06, EF=3.19, Dice=0.854

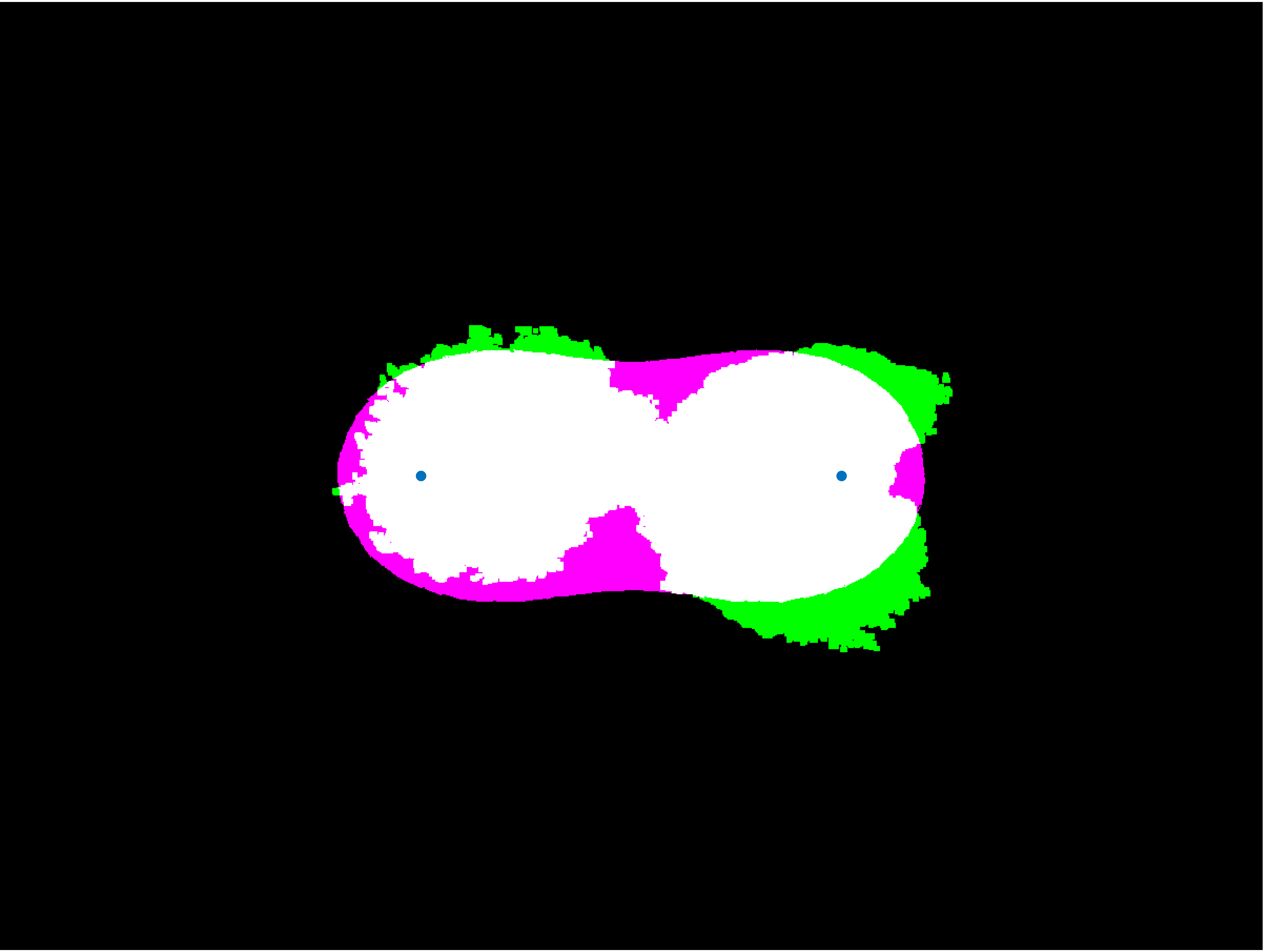

Lesion 47, Voltage=1300, Angle=6.1, LET= 551, AR=1.52, EF=2.81, Dice=0.883

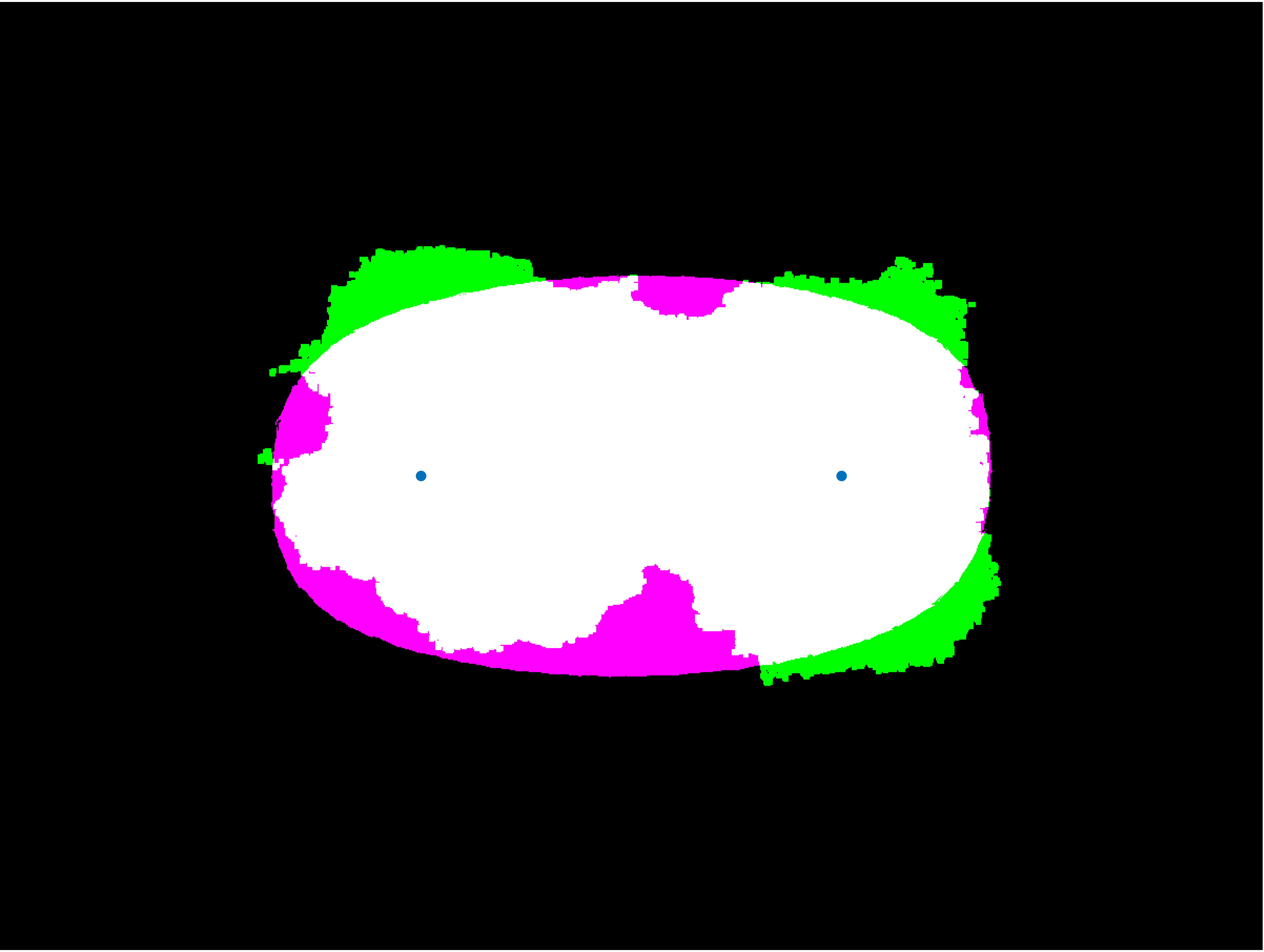

Lesion 48, Voltage=700, Angle=23.7, LET= 635, AR=6.21, EF=1.00, Dice=0.744

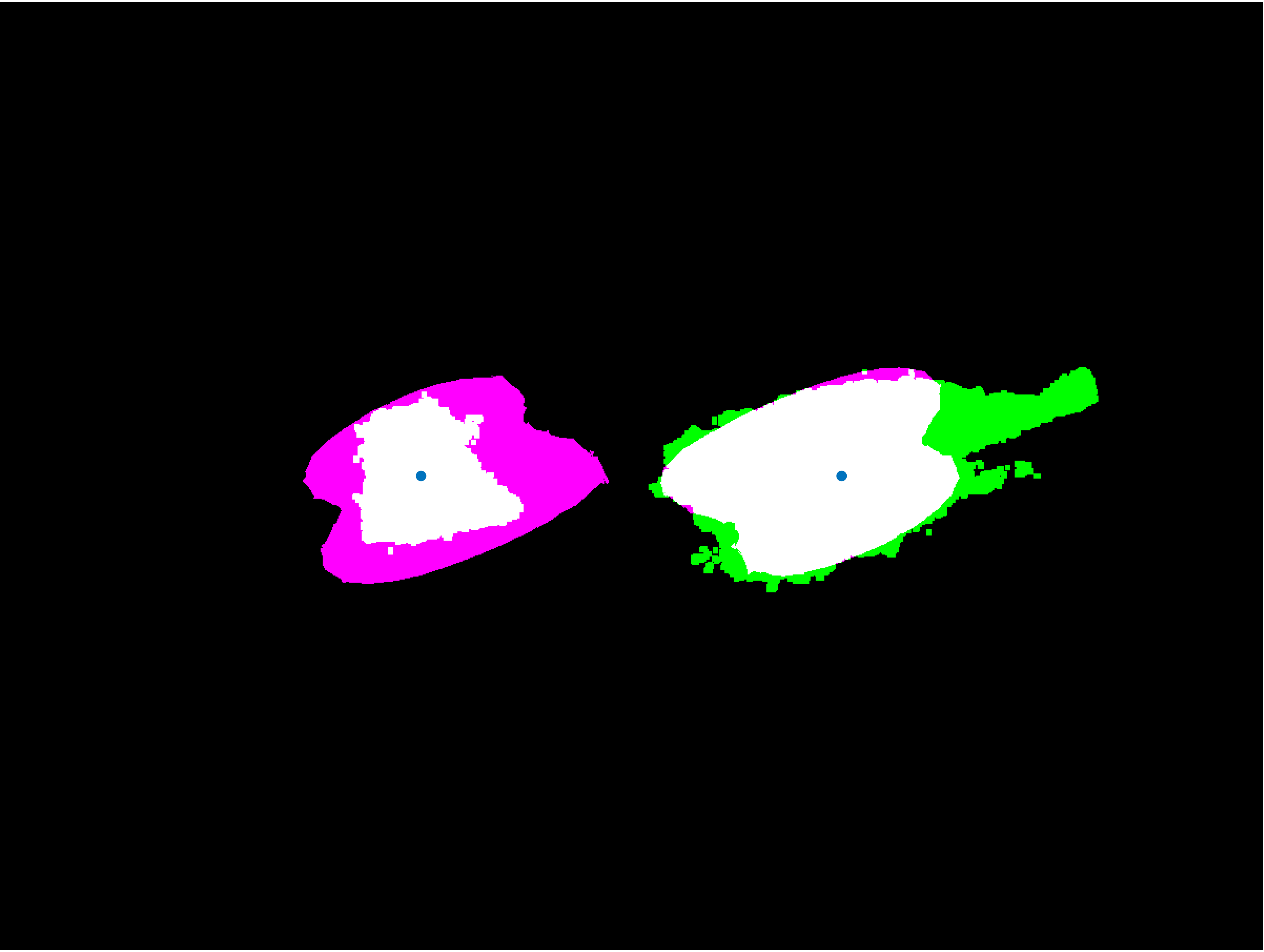

Lesion 49, Voltage=1000, Angle=6.1, LET= 701, AR=1.06, EF=3.11, Dice=0.828

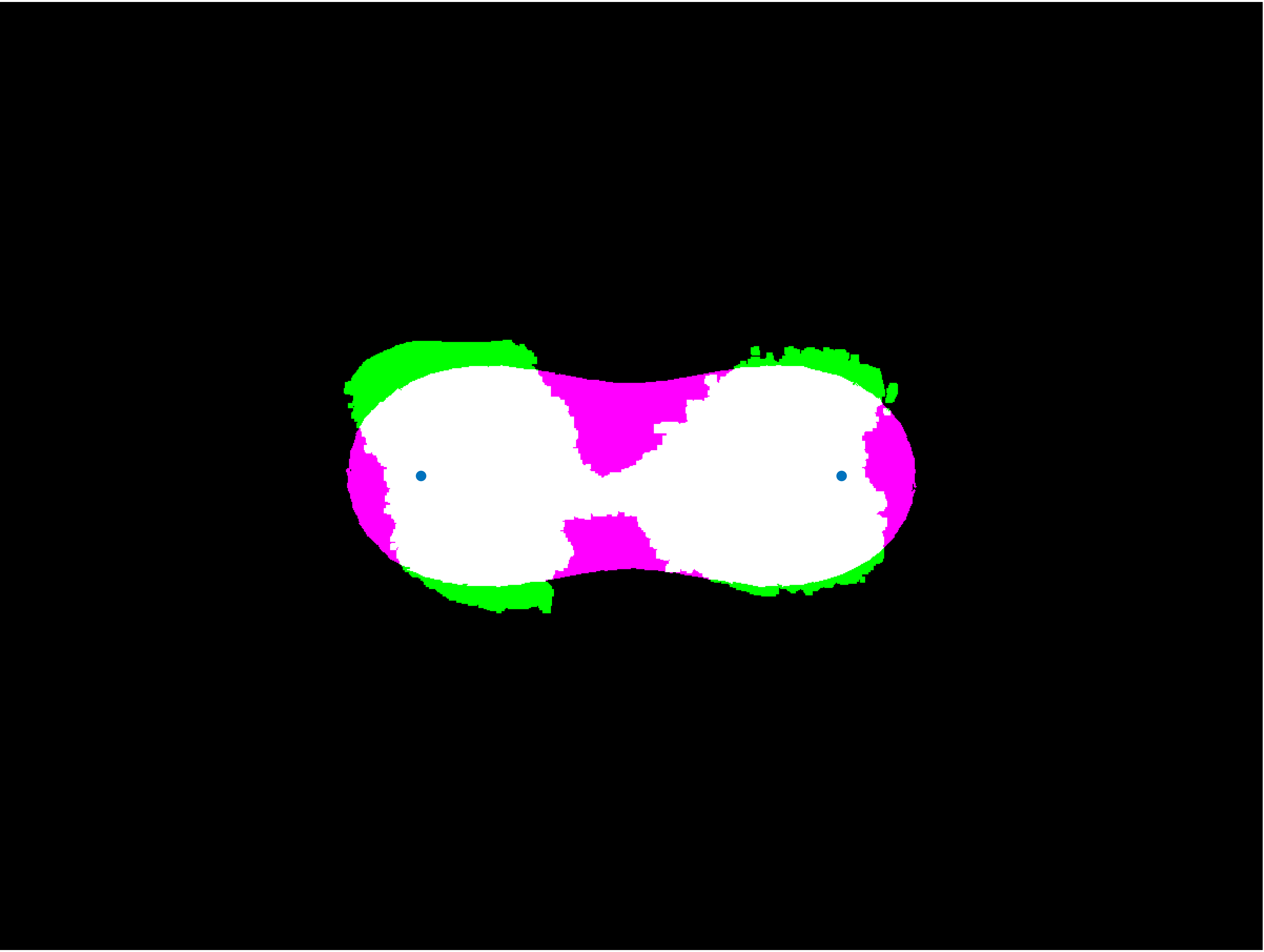

Lesion 50, Voltage=1100, Angle=-2.5, LET= 474, AR=1.04, EF=1.01, Dice=0.854

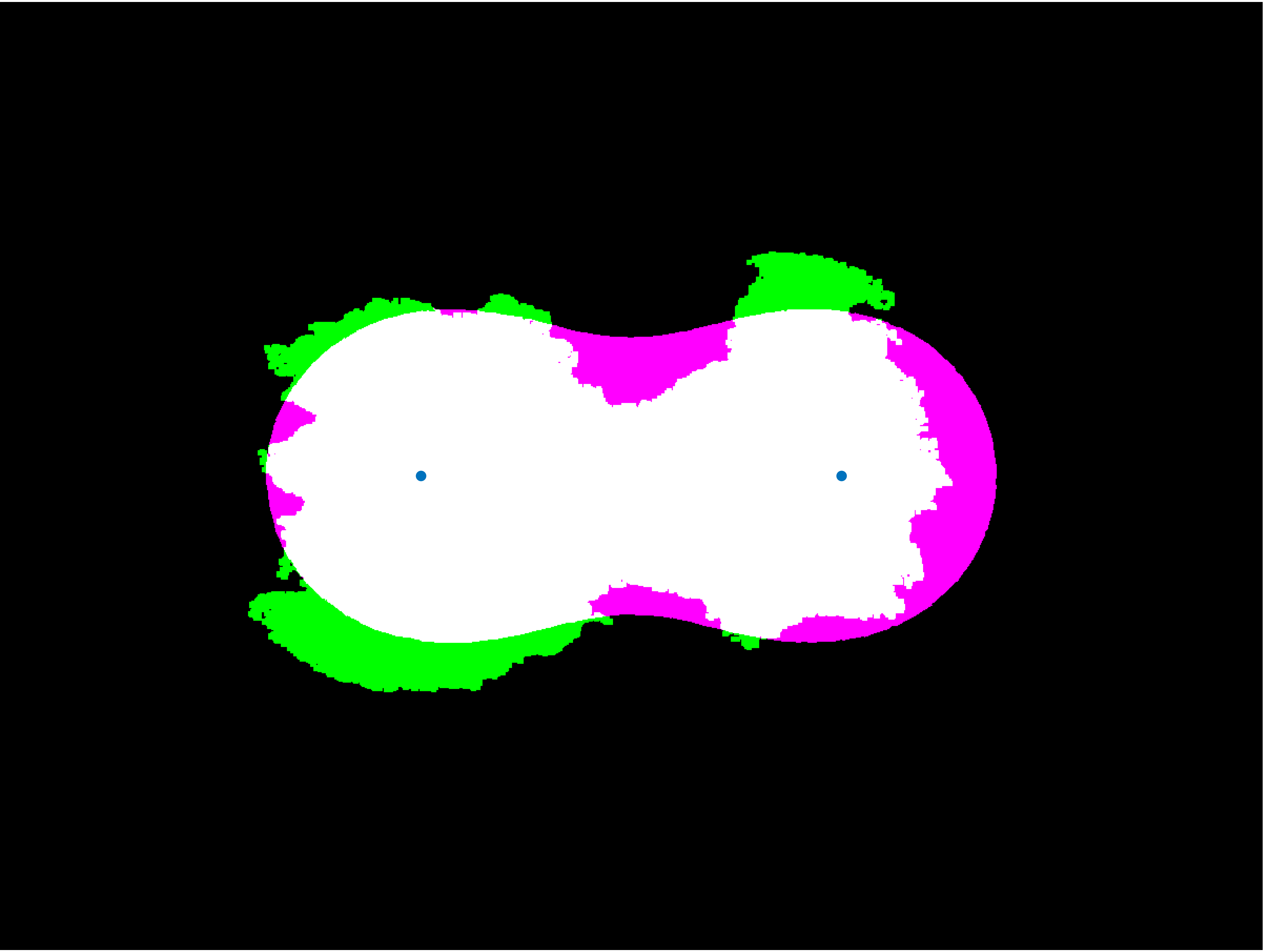

Lesion 51, Voltage=1200, Angle=-30.6, LET= 570, AR=1.03, EF=1.21, Dice=0.886

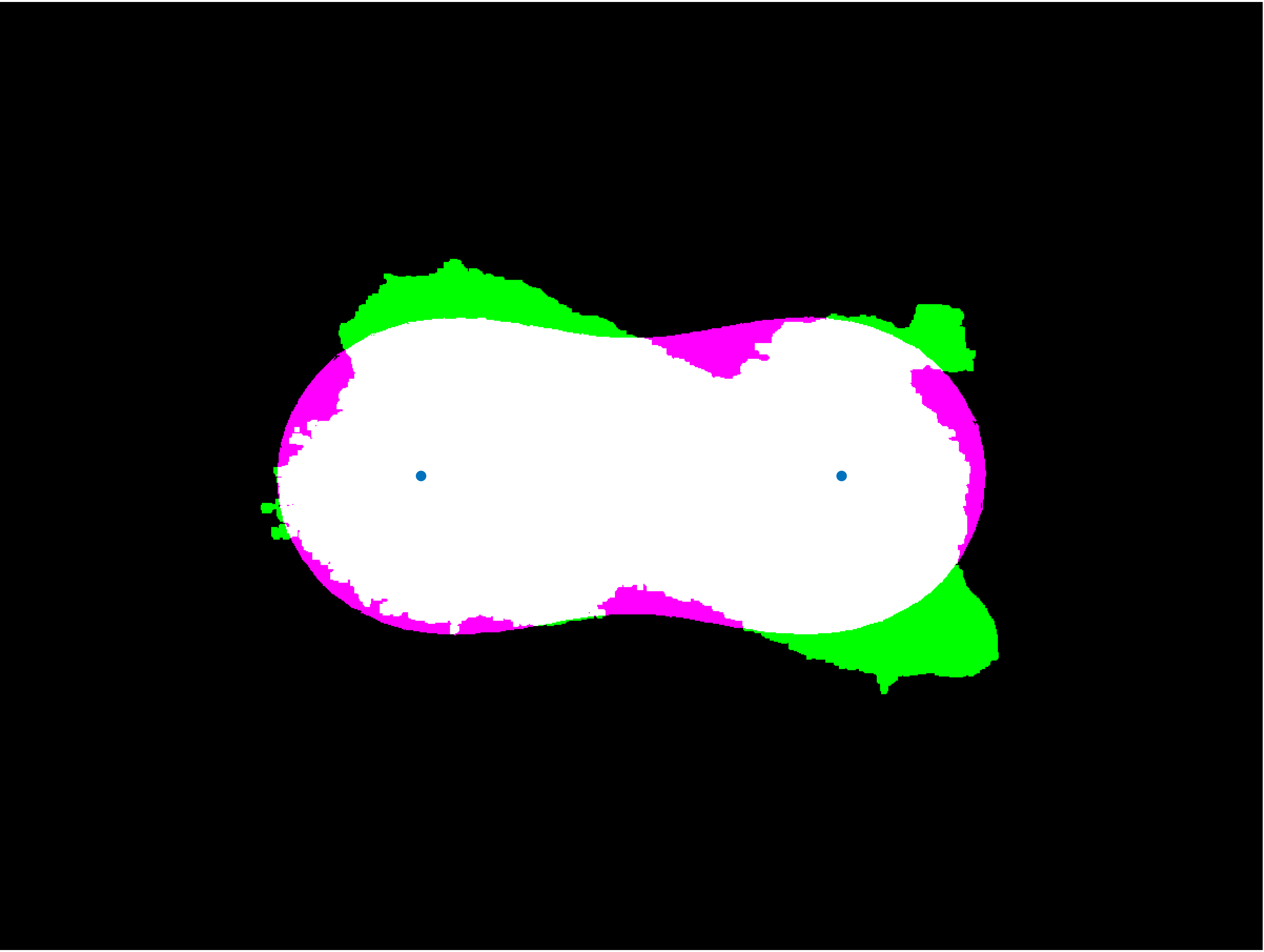

4 Lesion images vs model for MDT human

Lesion 1, Voltage=700, Angle=24.5, LET= 567, AR=1.08, EF=3.24, Dice=0.843

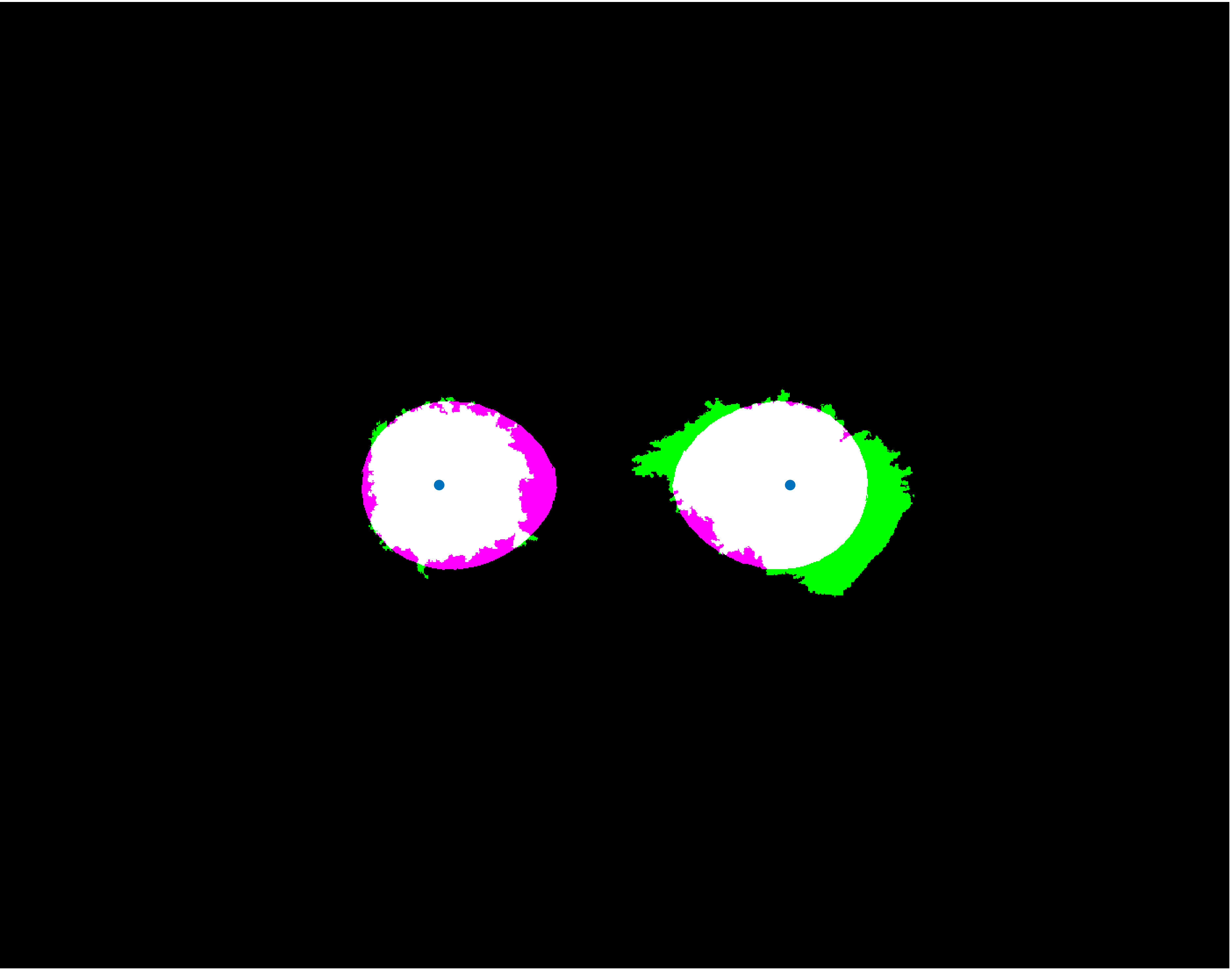

Lesion 2, Voltage=700, Angle=47.0, LET= 572, AR=2.33, EF=1.06, Dice=0.848

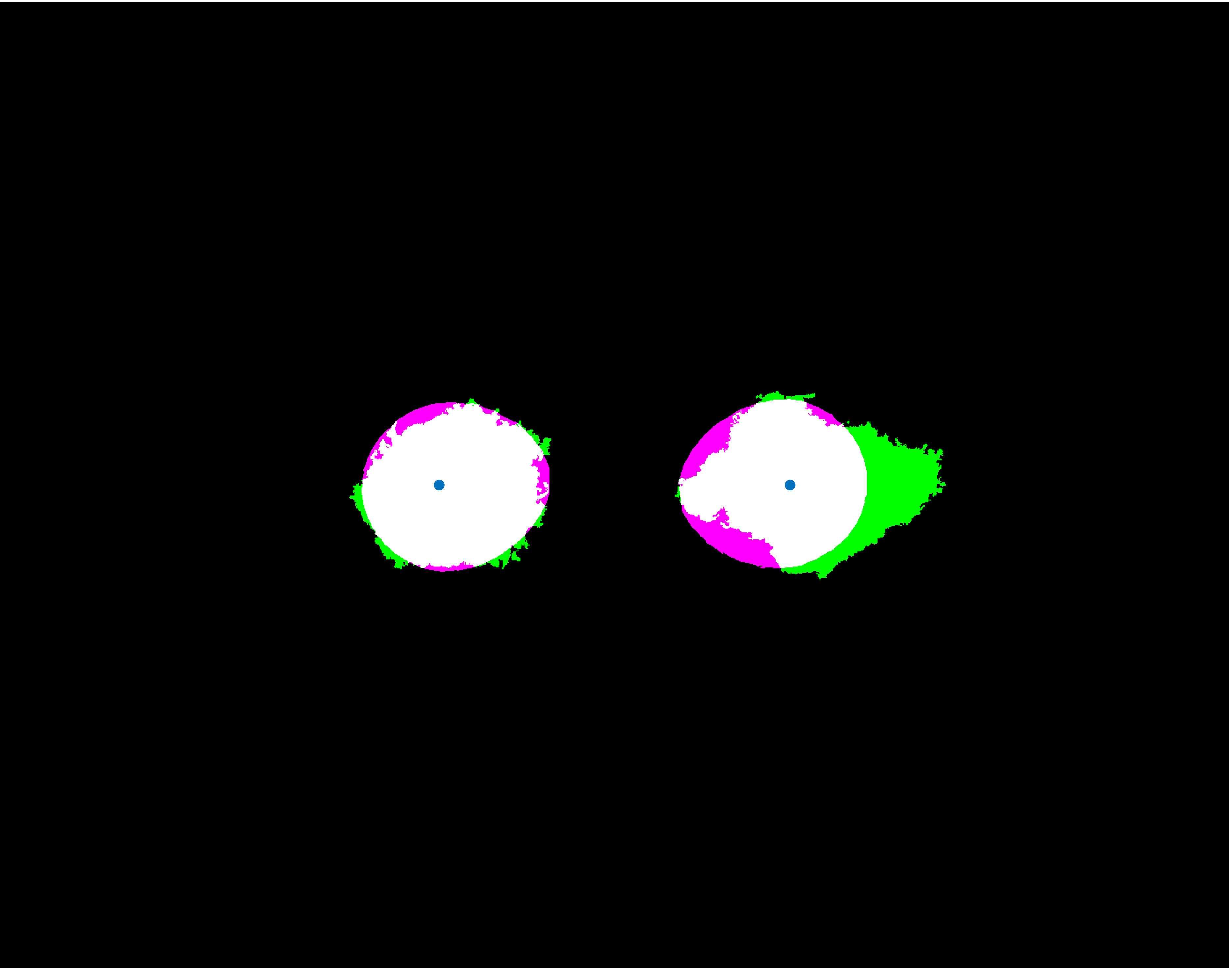

Lesion 3, Voltage=1000, Angle=62.6, LET= 408, AR=1.06, EF=3.19, Dice=0.842

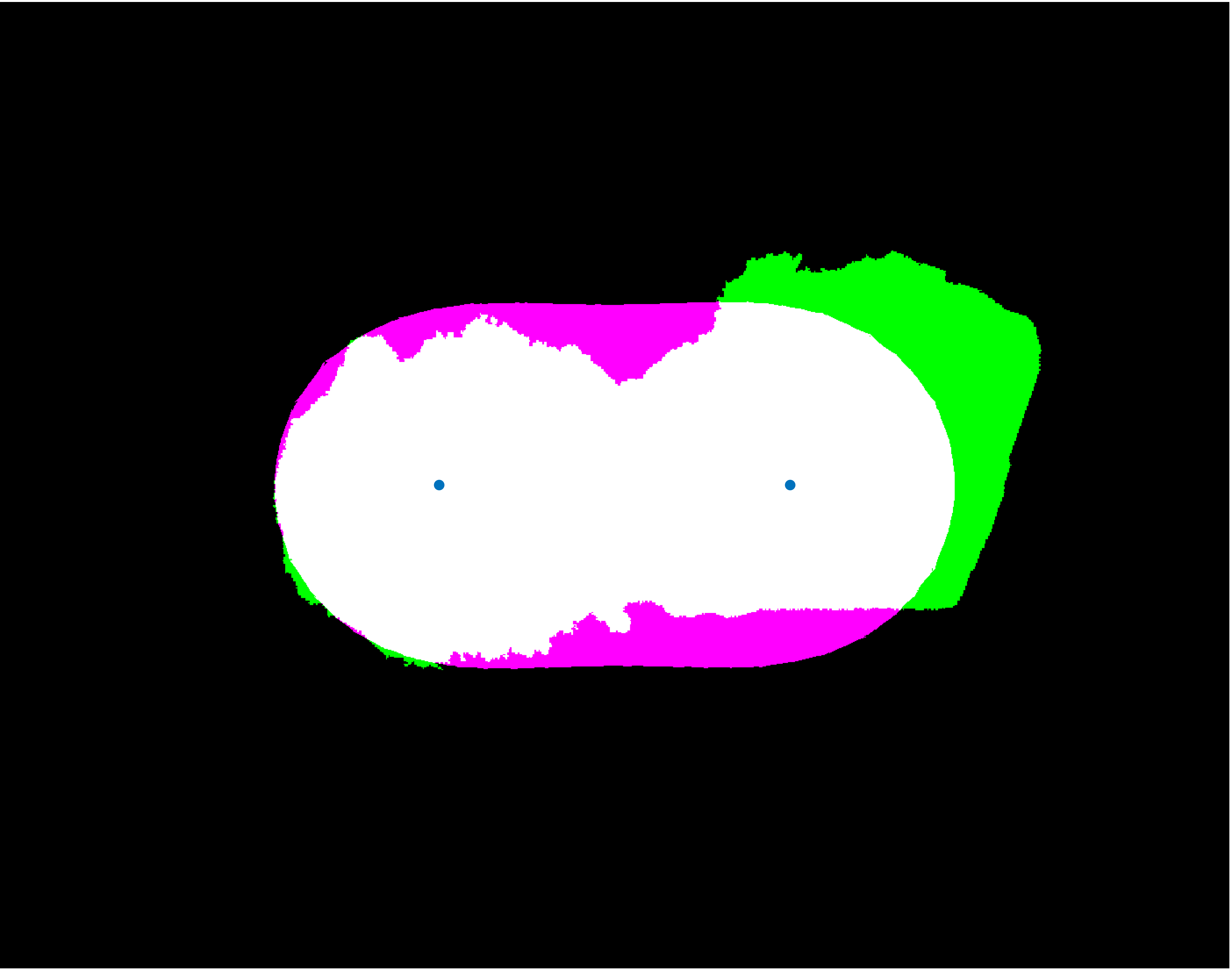

Lesion 4, Voltage=1000, Angle=57.5, LET= 495, AR=3.09, EF=8.88, Dice=0.876

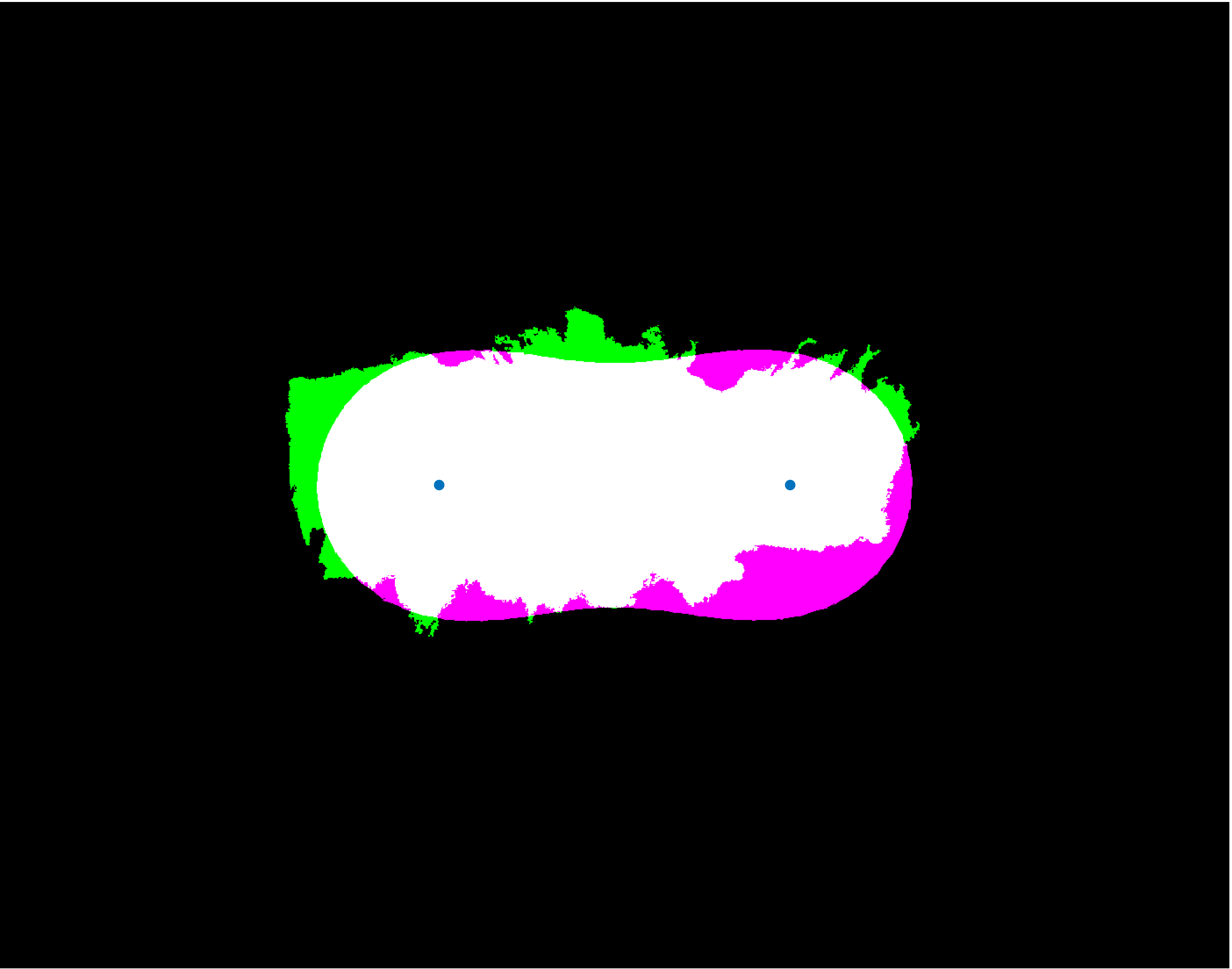

Lesion 5, Voltage=1300, Angle=62.0, LET= 423, AR=1.40, EF=2.18, Dice=0.856

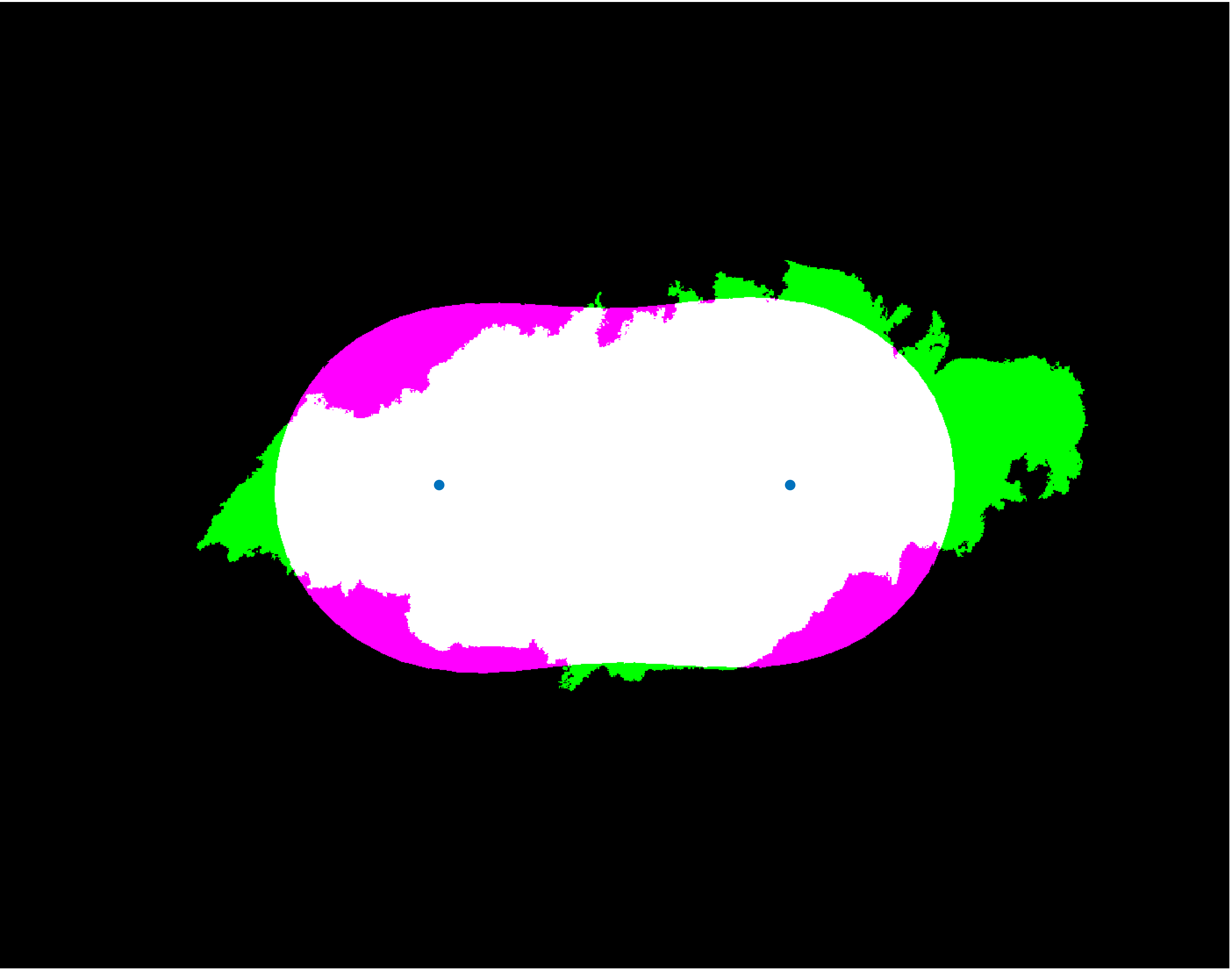

Lesion 6, Voltage=1300, Angle=79.4, LET= 465, AR=1.05, EF=3.02, Dice=0.866

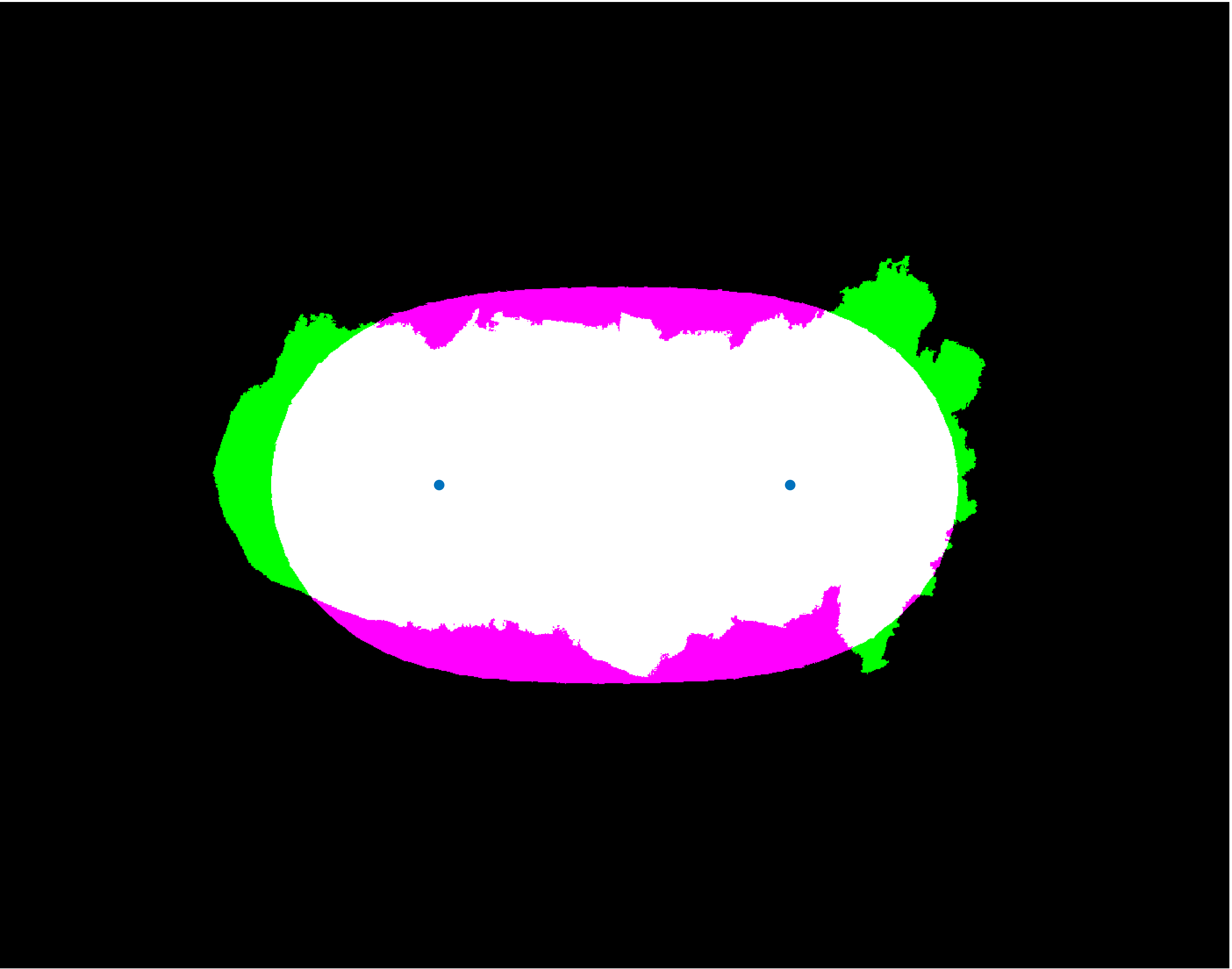

Lesion 7, Voltage=1500, Angle=37.6, LET= 198, AR=3.20, EF=9.98, Dice=0.897

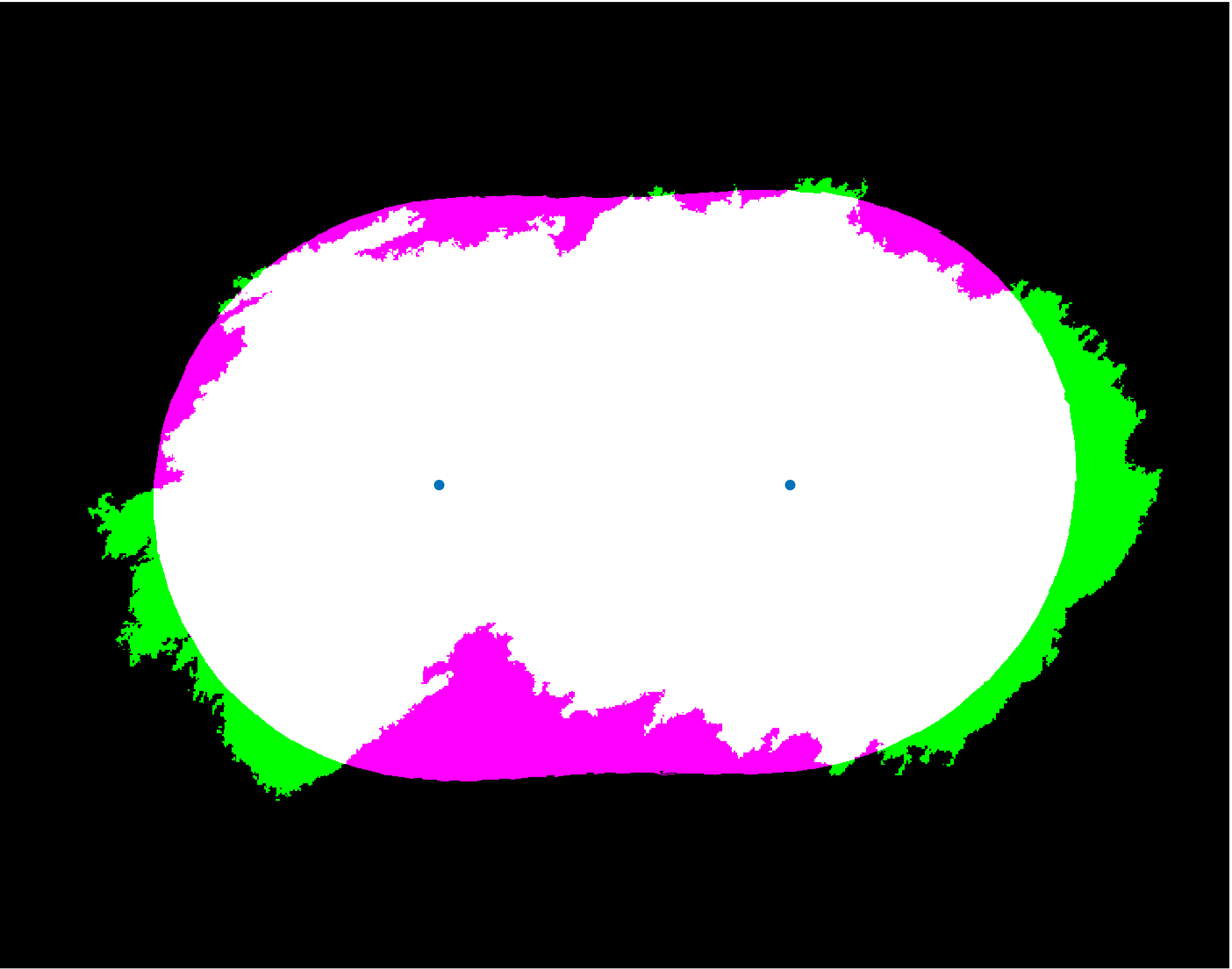

Lesion 8, Voltage=1500, Angle=50.7, LET= 352, AR=3.00, EF=2.05, Dice=0.902

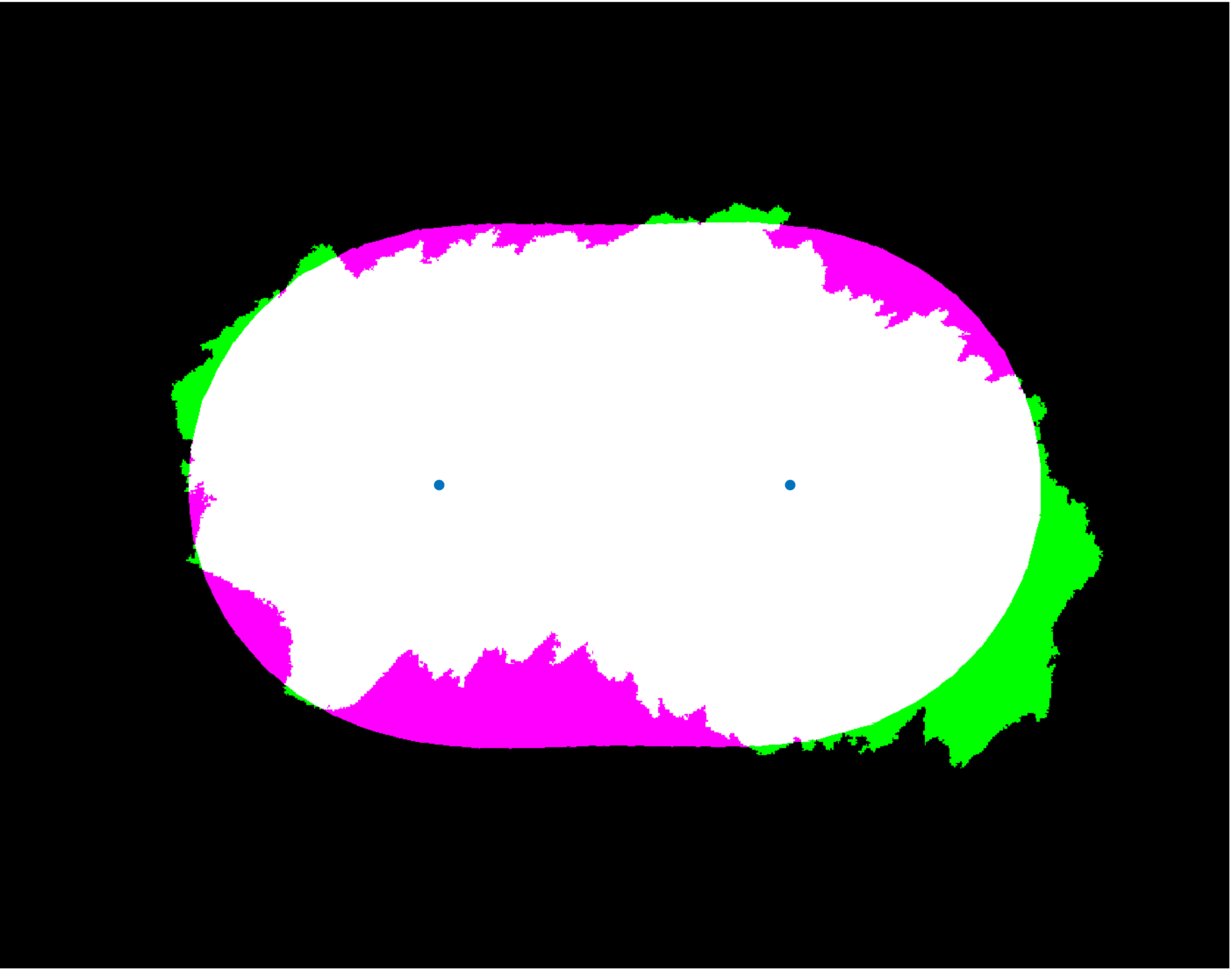

Lesion 9, Voltage=1500, Angle=68.5, LET= 416, AR=6.33, EF=1.95, Dice=0.851

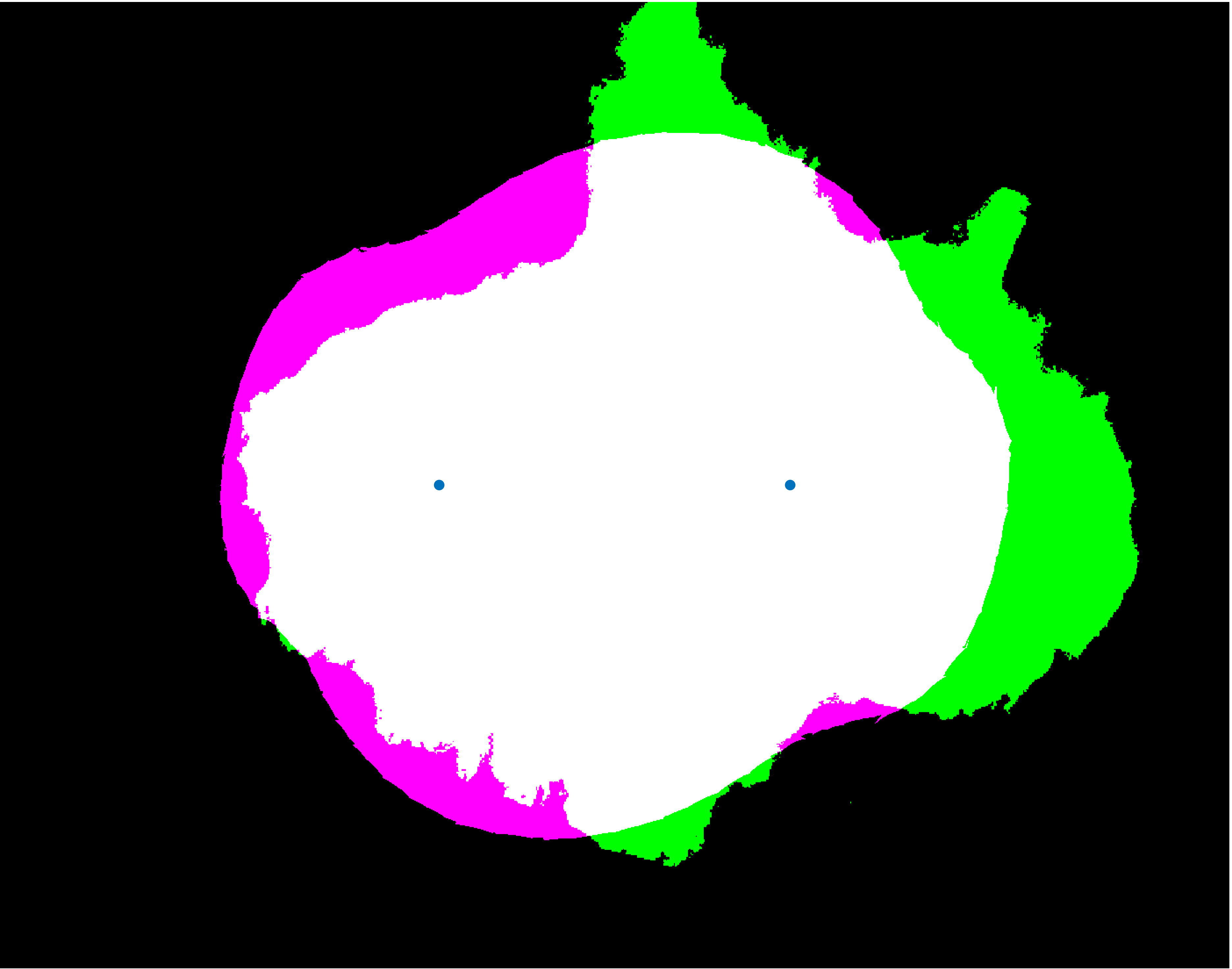

Lesion 10, Voltage=1200, Angle=-27.5, LET= 371, AR=4.85, EF=1.09, Dice=0.874

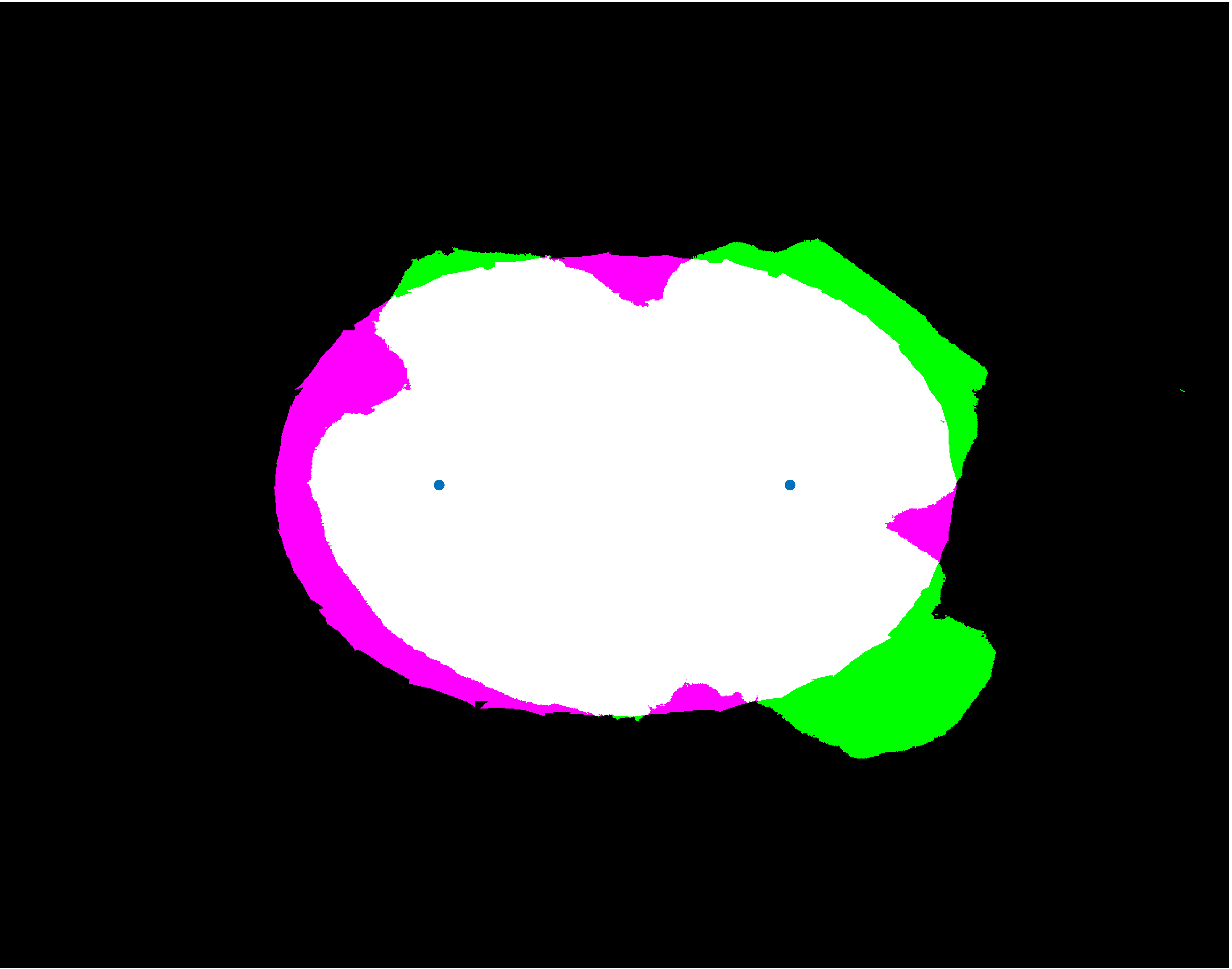

Lesion 11, Voltage=1300, Angle=-23.3, LET= 382, AR=5.14, EF=1.05, Dice=0.806

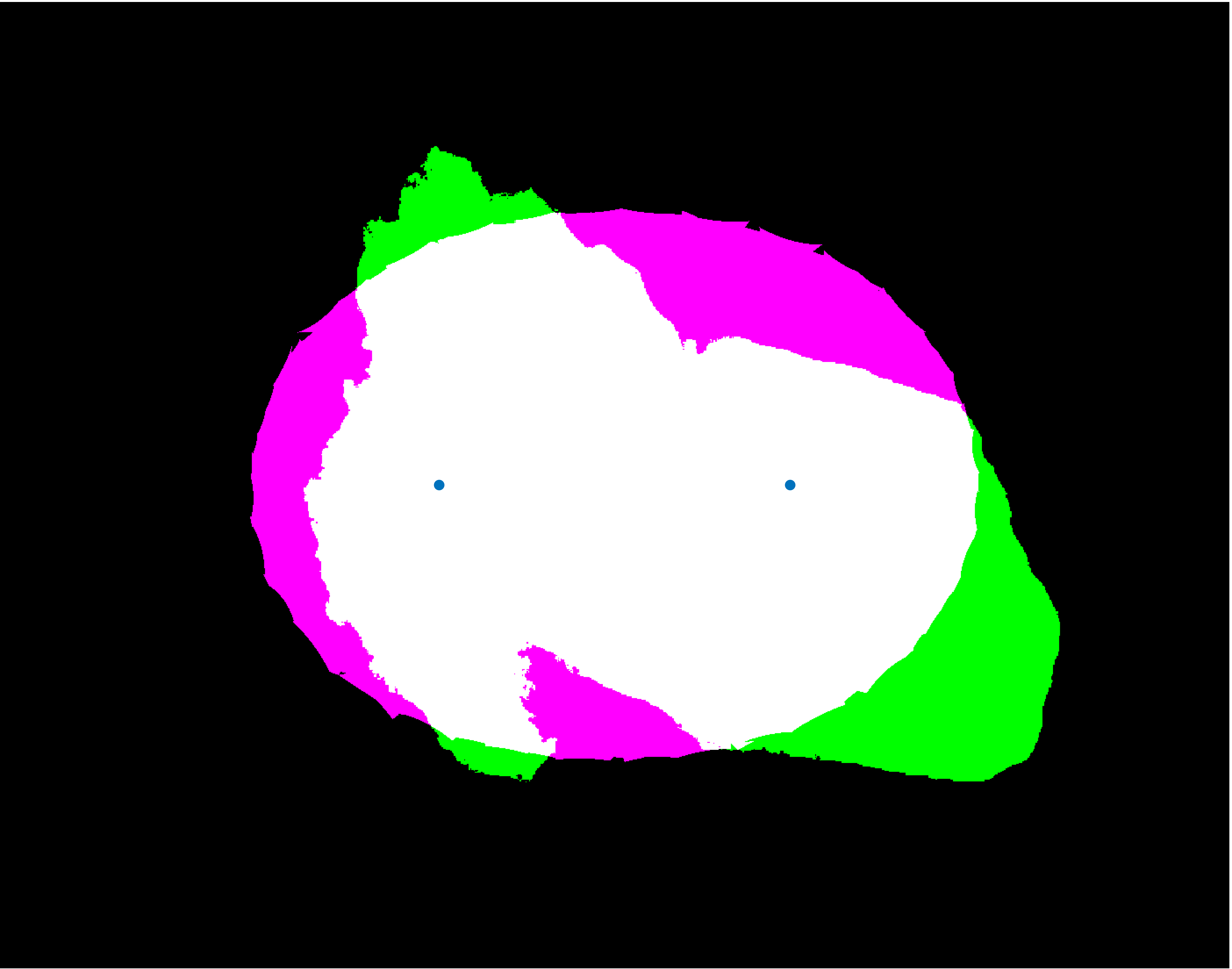

Lesion 12, Voltage=1500, Angle=-2.5, LET= 397, AR=2.90, EF=1.02, Dice=0.887

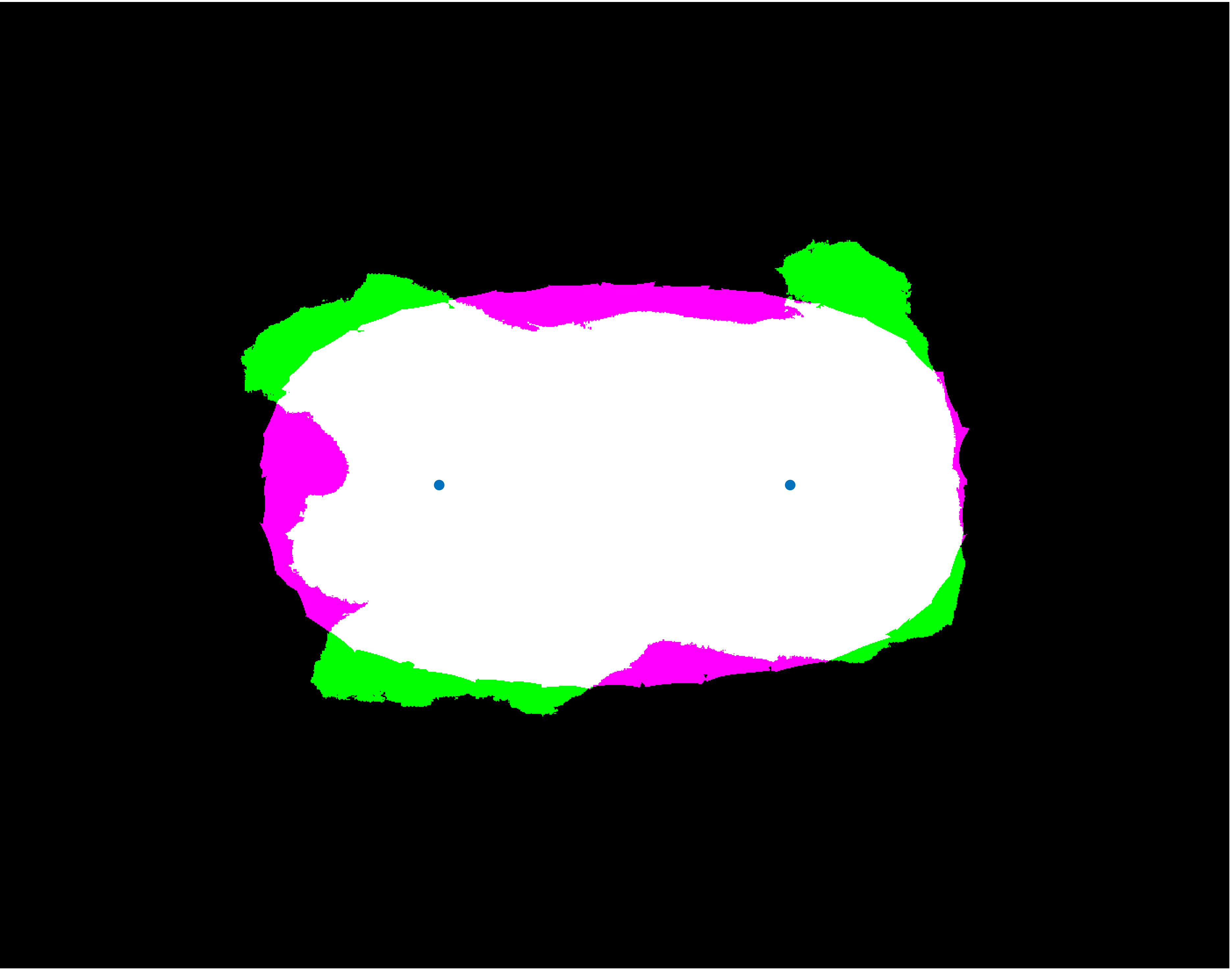

Lesion 13, Voltage=1000, Angle=-18.4, LET= 340, AR=2.66, EF=2.81, Dice=0.873

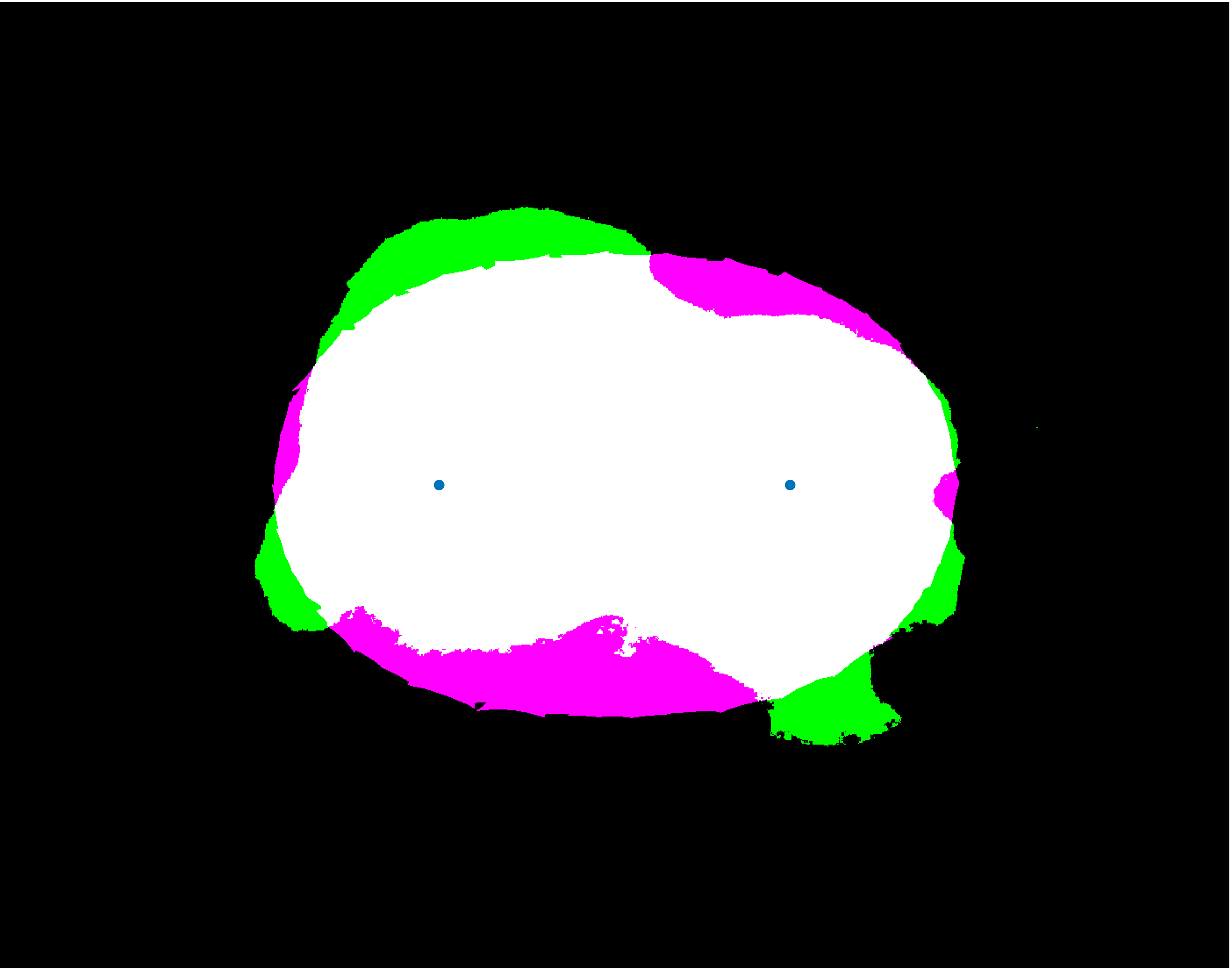

Lesion 14, Voltage=1200, Angle=-25.8, LET= 327, AR=4.30, EF=3.58, Dice=0.901

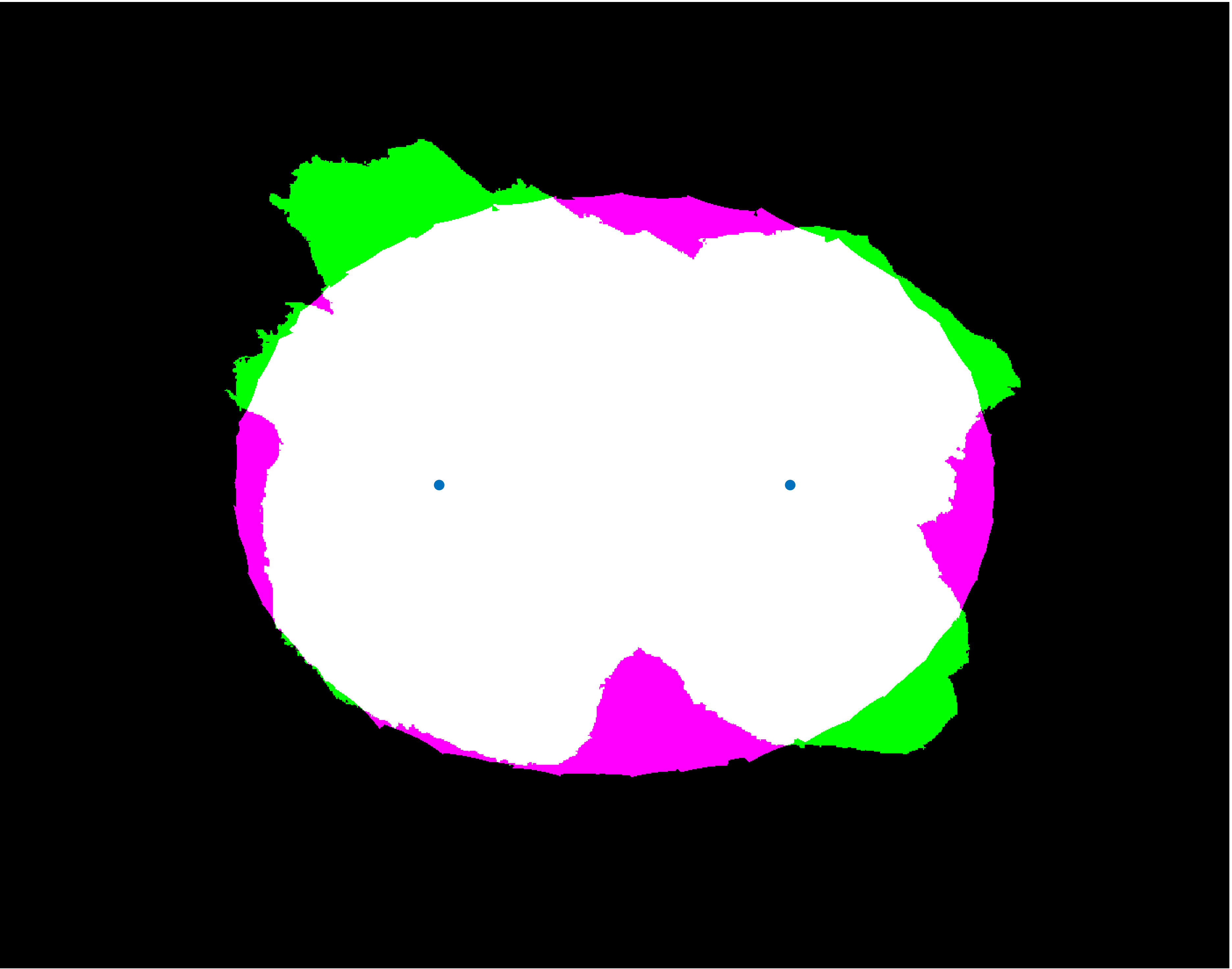

Lesion 15, Voltage=700, Angle=8.7, LET= 624, AR=1.86, EF=1.02, Dice=0.853

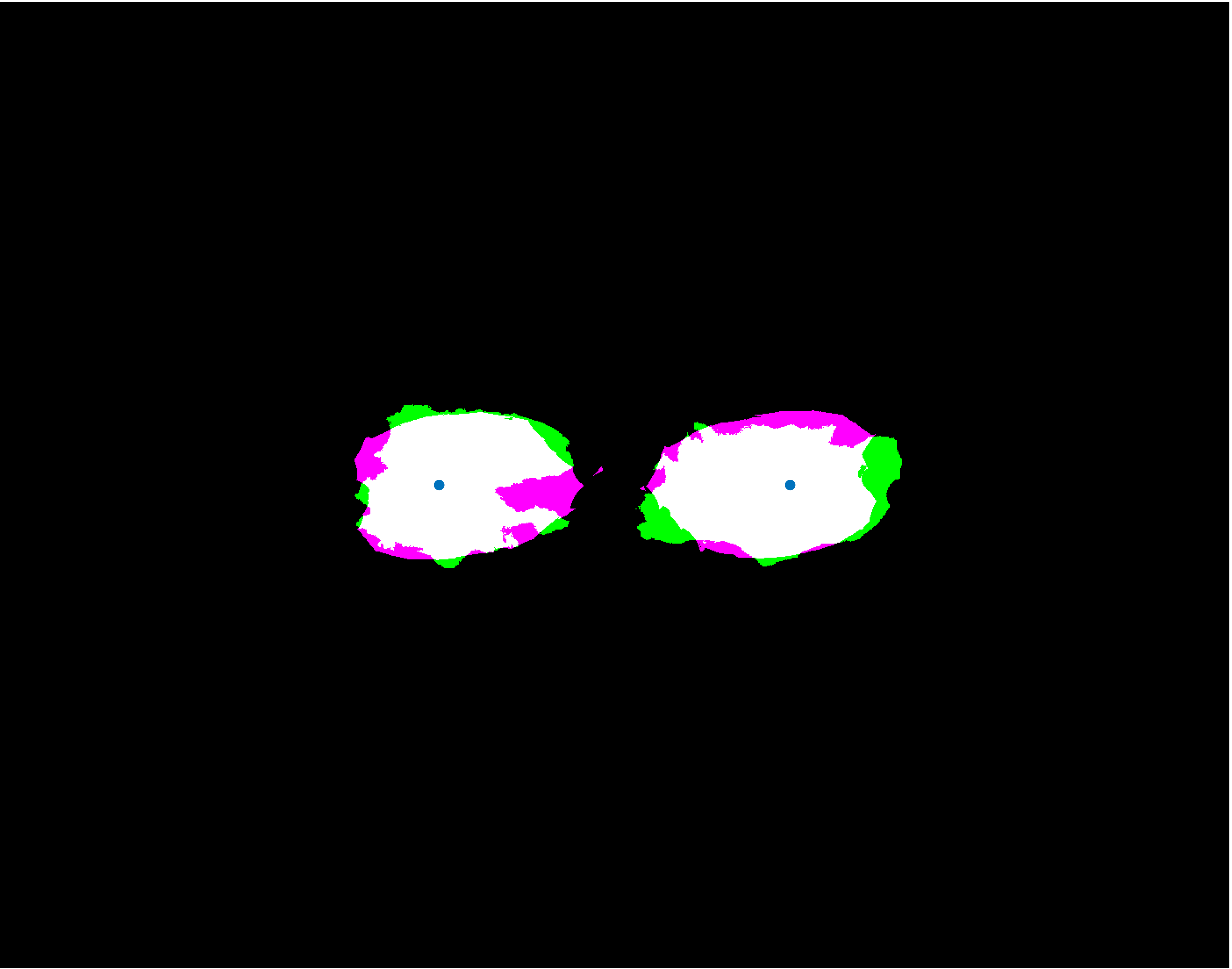

Lesion 16, Voltage=1000, Angle=76.8, LET= 557, AR=1.94, EF=2.46, Dice=0.858

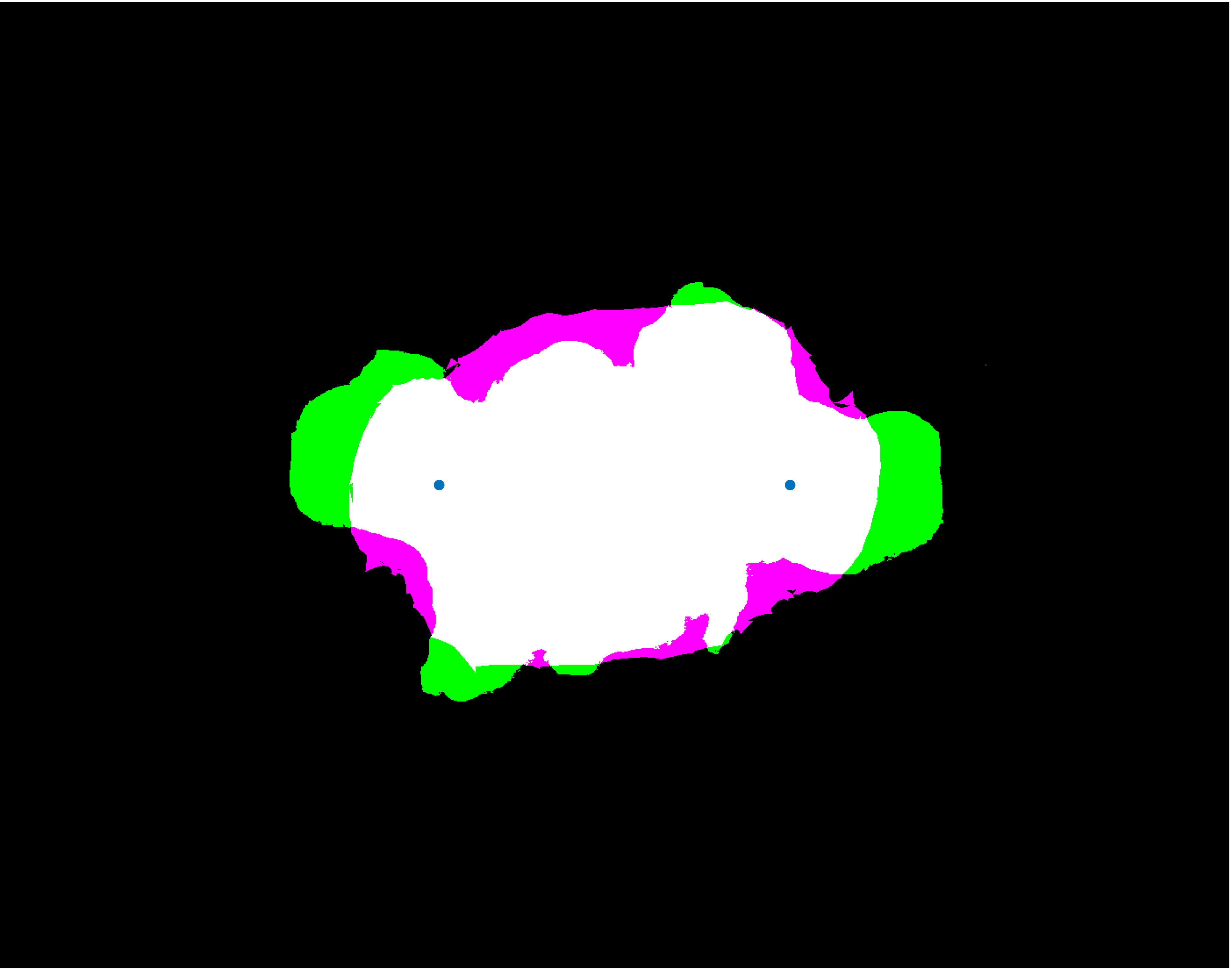

Lesion 17, Voltage=1200, Angle=40.3, LET= 502, AR=1.06, EF=3.03, Dice=0.853

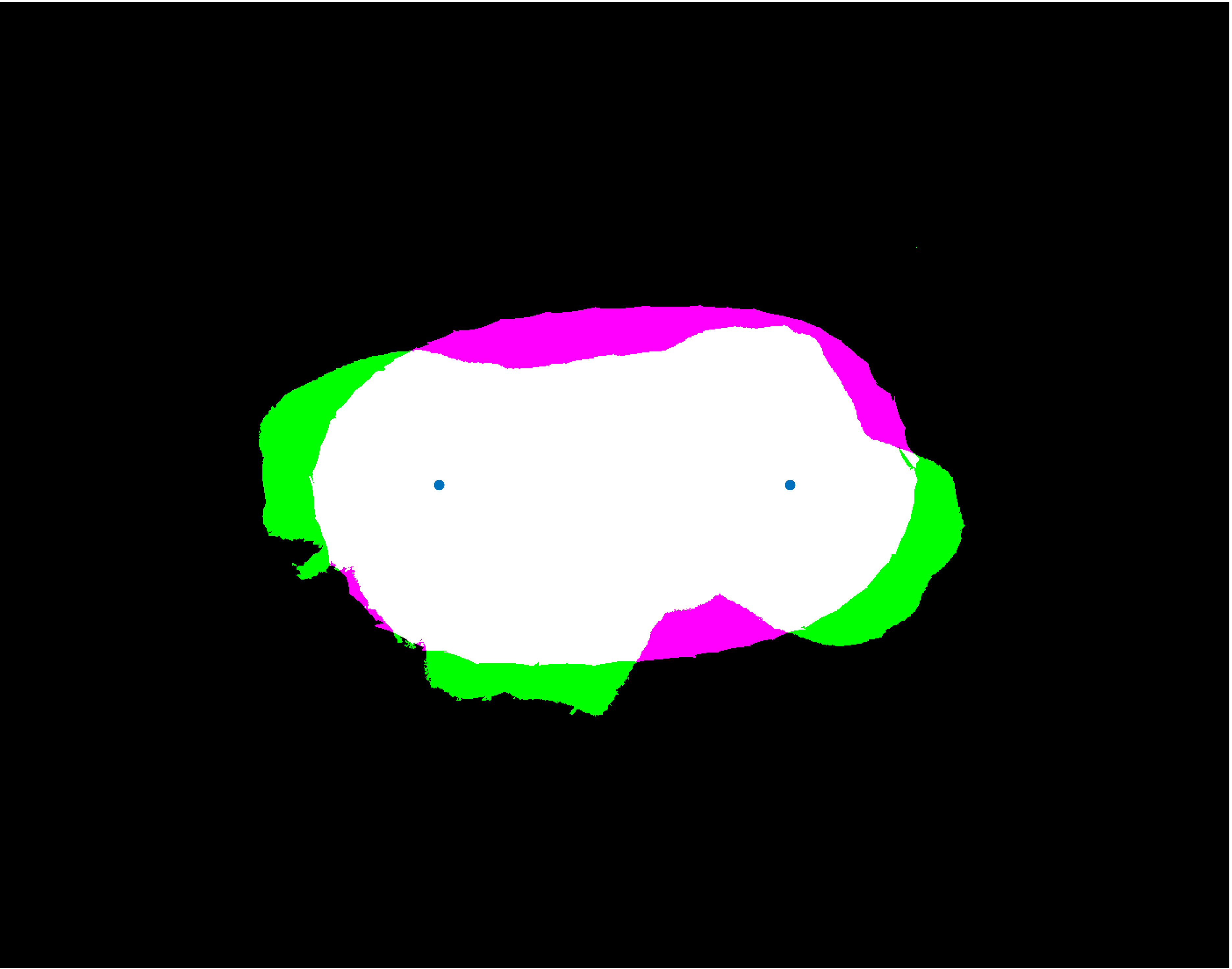

Lesion 18, Voltage=700, Angle=11.0, LET= 262, AR=2.46, EF=7.65, Dice=0.721

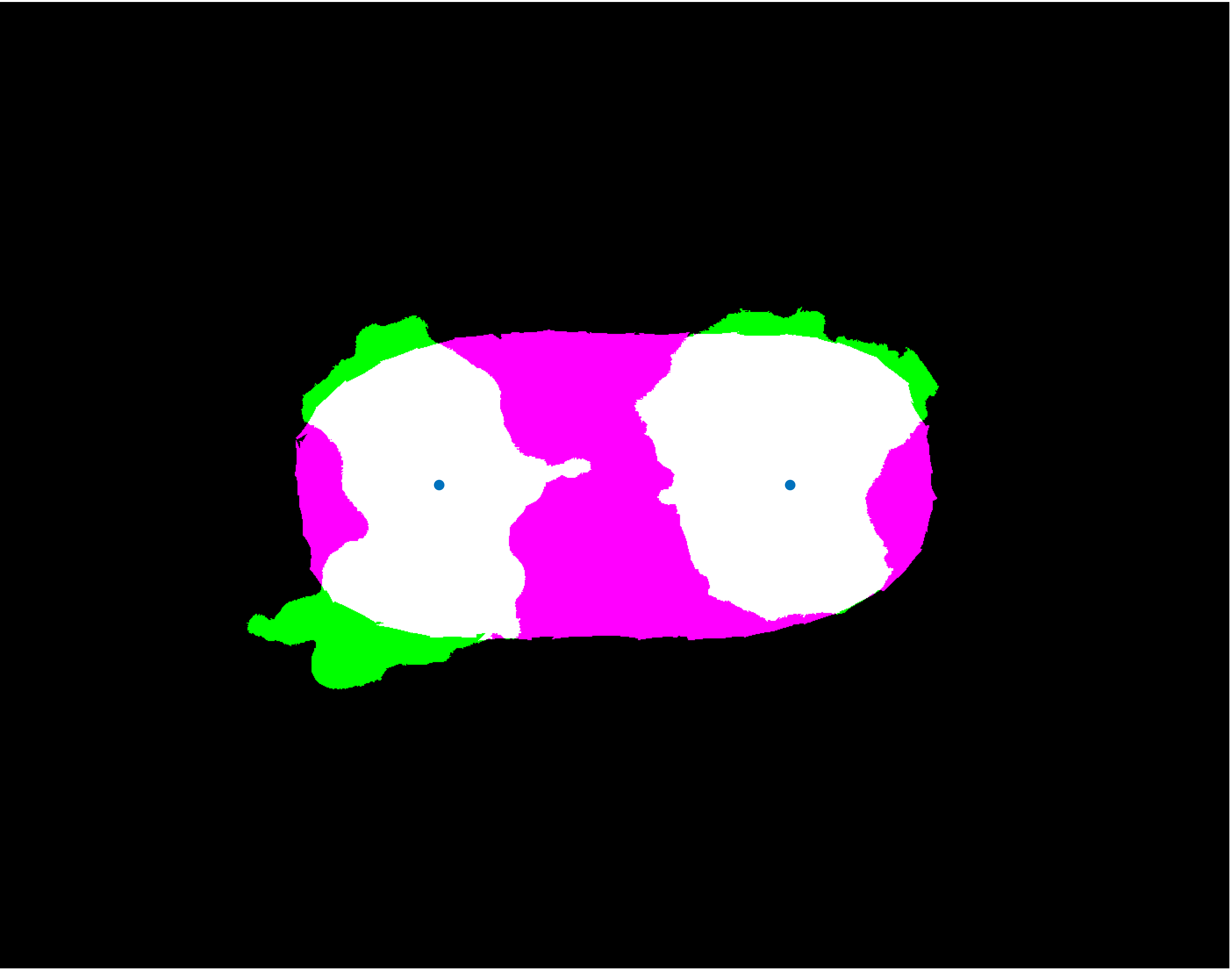

Lesion 19, Voltage=700, Angle=64.0, LET= 704, AR=1.24, EF=3.14, Dice=0.782

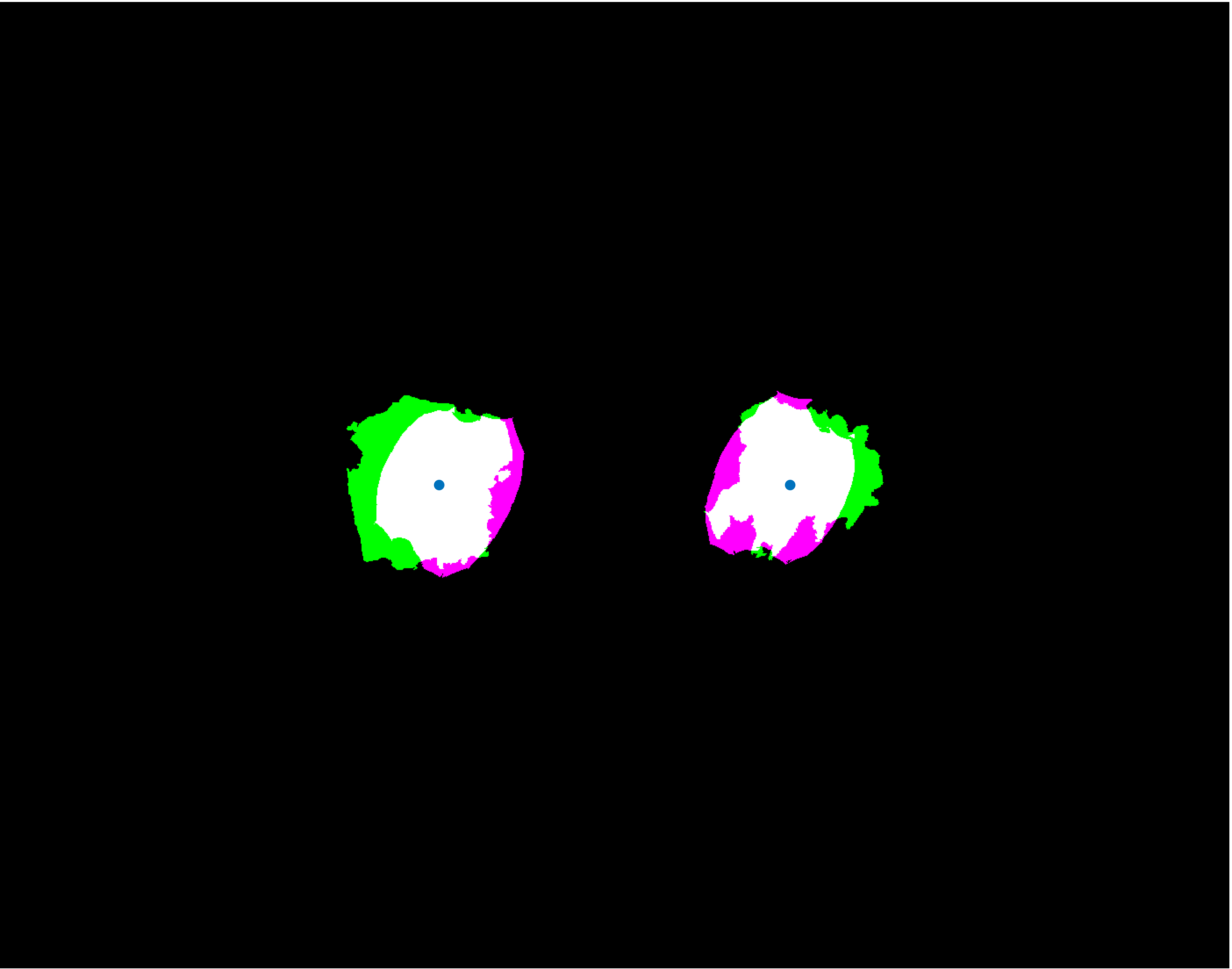

Lesion 20, Voltage=1200, Angle=-66.7, LET= 175, AR=1.95, EF=6.23, Dice=0.777

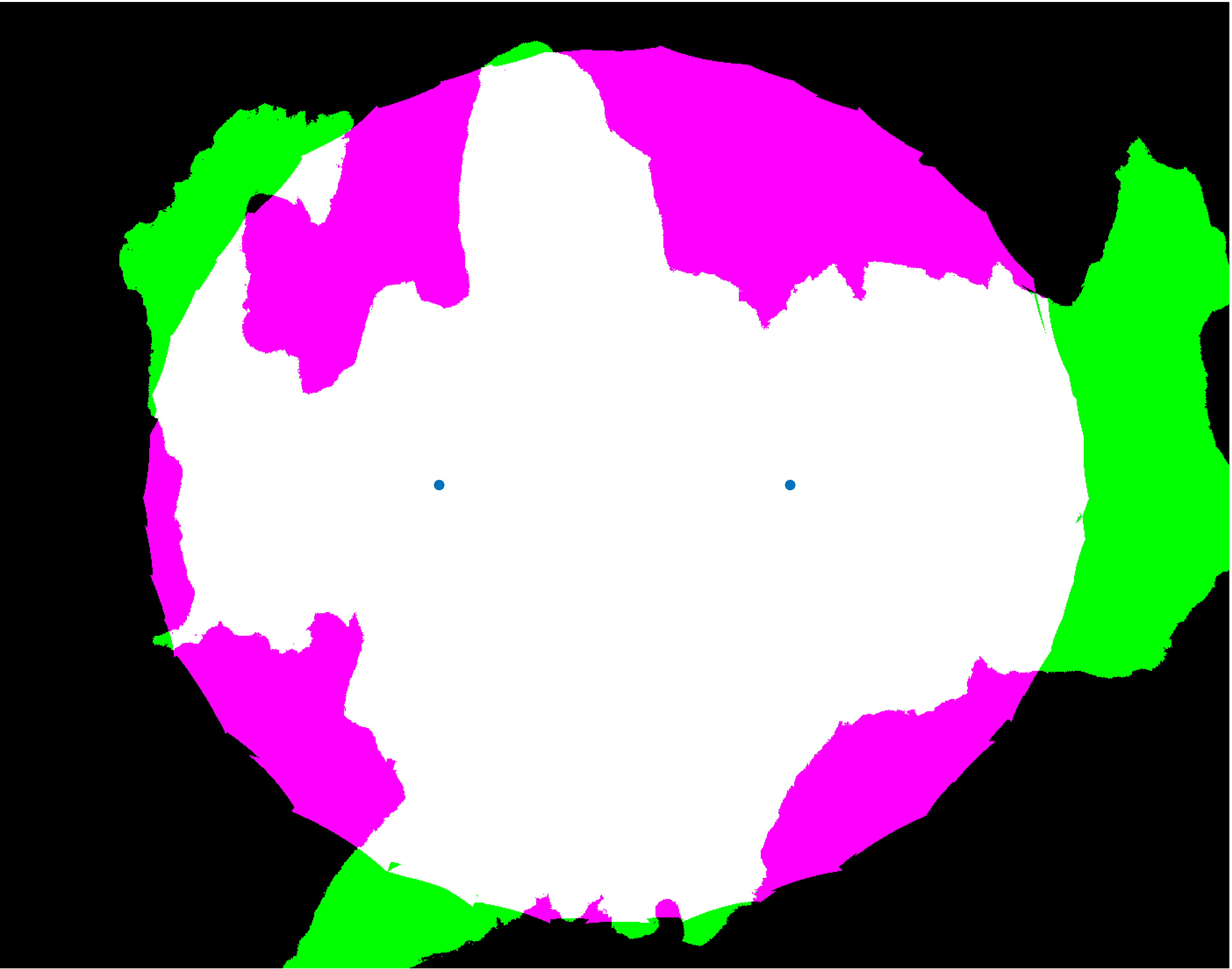

Lesion 21, Voltage=1500, Angle=-14.7, LET= 497, AR=2.03, EF=1.12, Dice=0.900

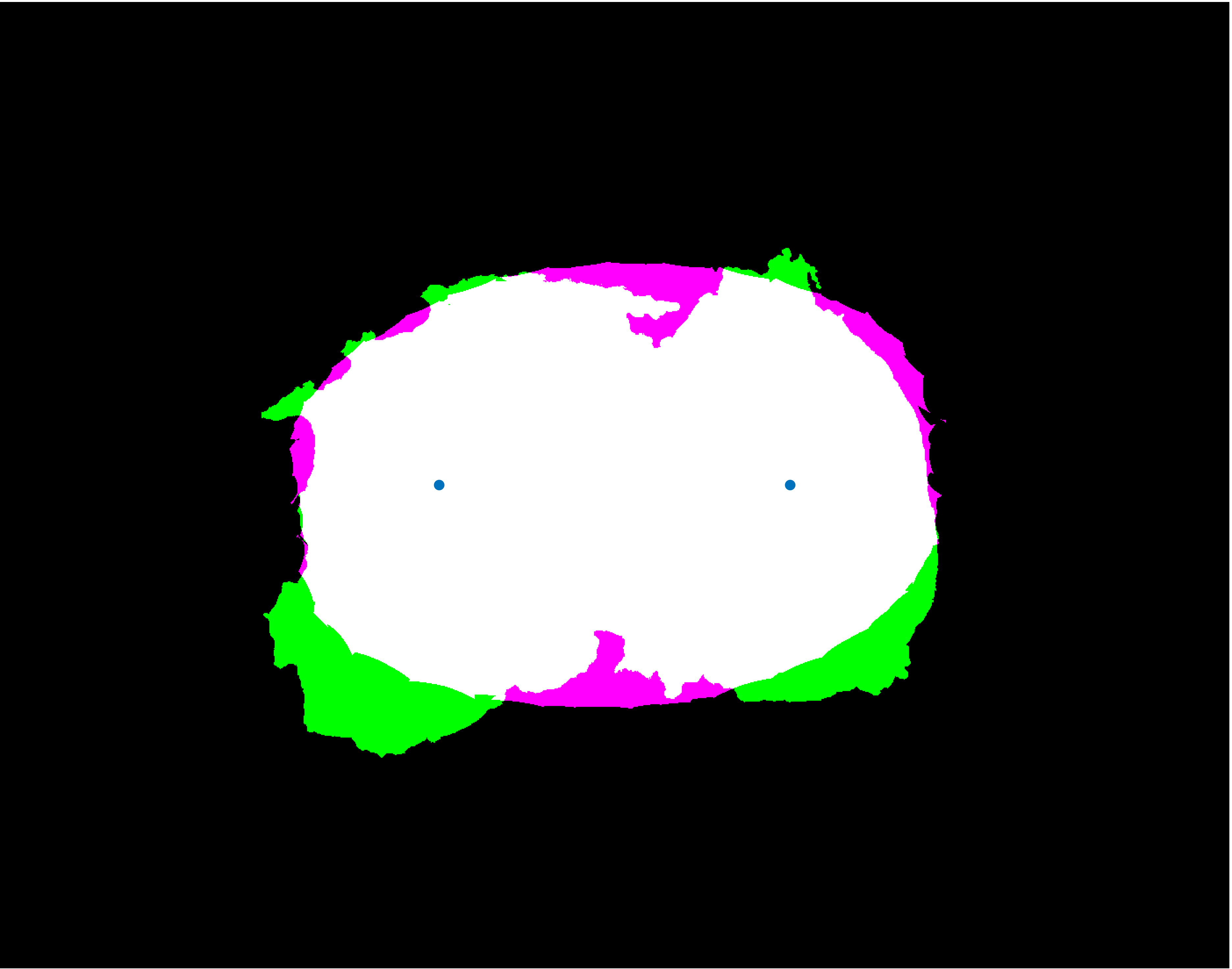

5 Lesion images vs model for 100 μs pig

Lesion 1, Voltage=600, Angle=10.1, LET= 415, AR=1.08, EF=3.24, Dice=0.892

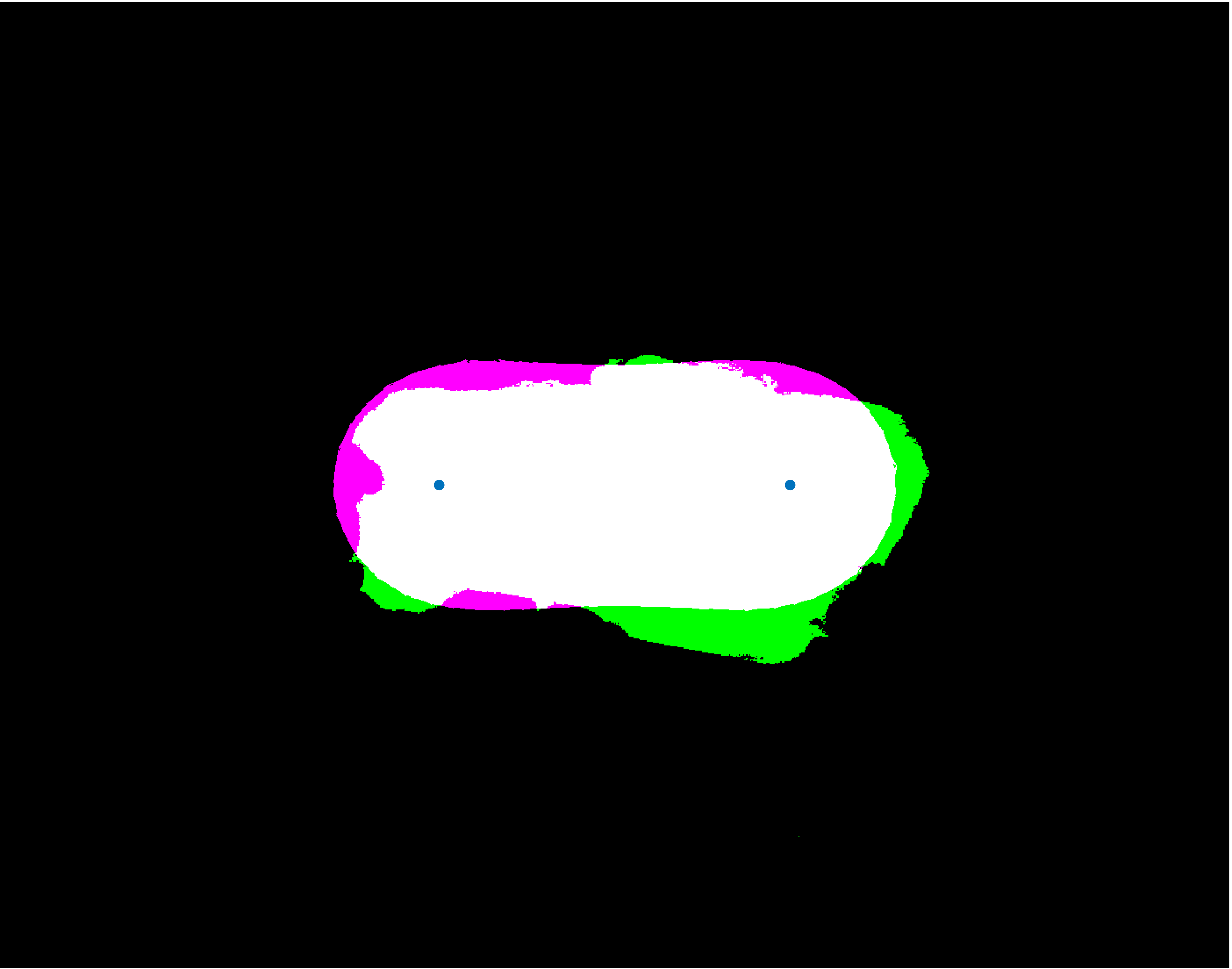

Lesion 2, Voltage=800, Angle=7.7, LET= 293, AR=2.33, EF=1.06, Dice=0.857

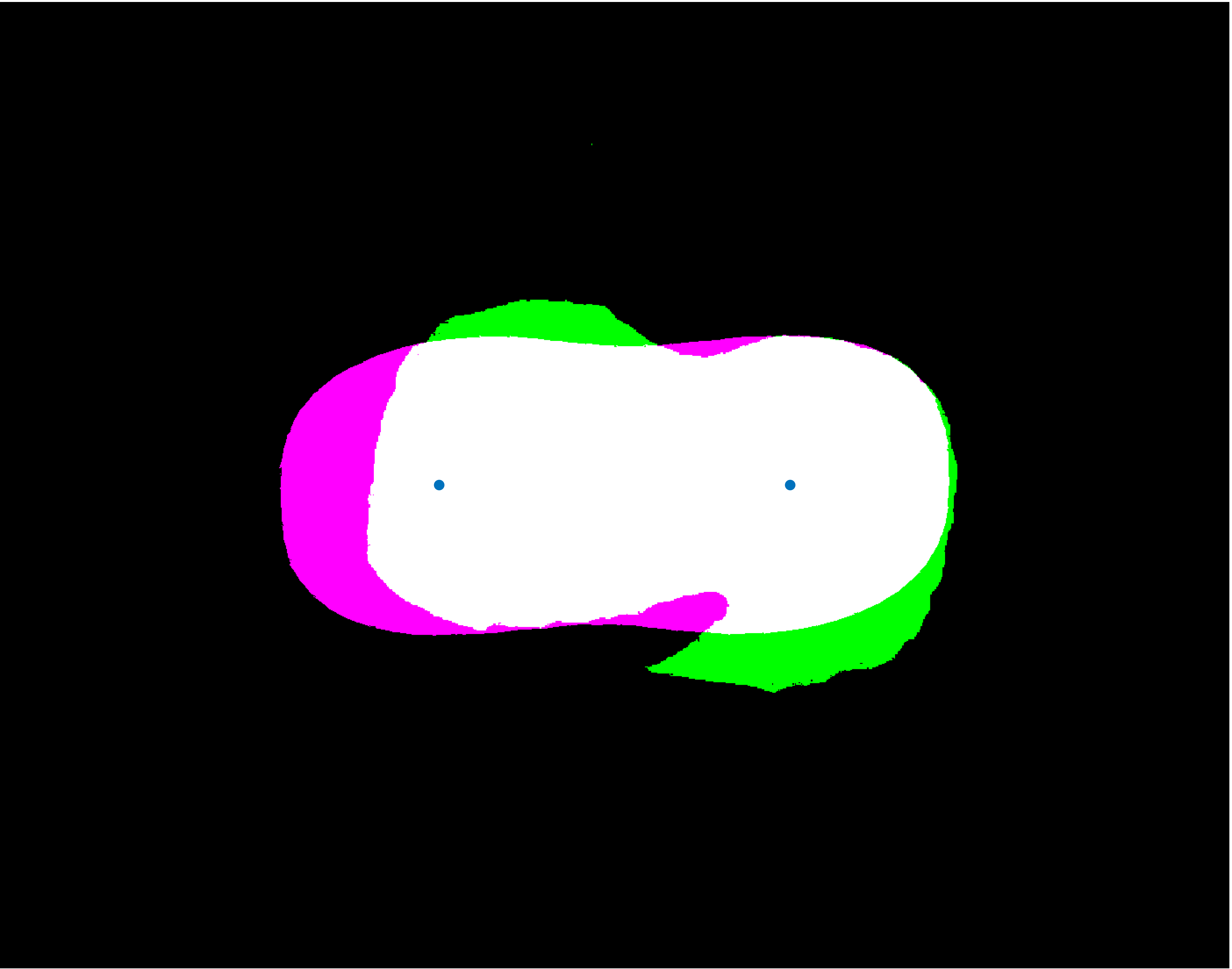

Lesion 3, Voltage=1000, Angle=-1.9, LET= 351, AR=1.06, EF=3.19, Dice=0.825

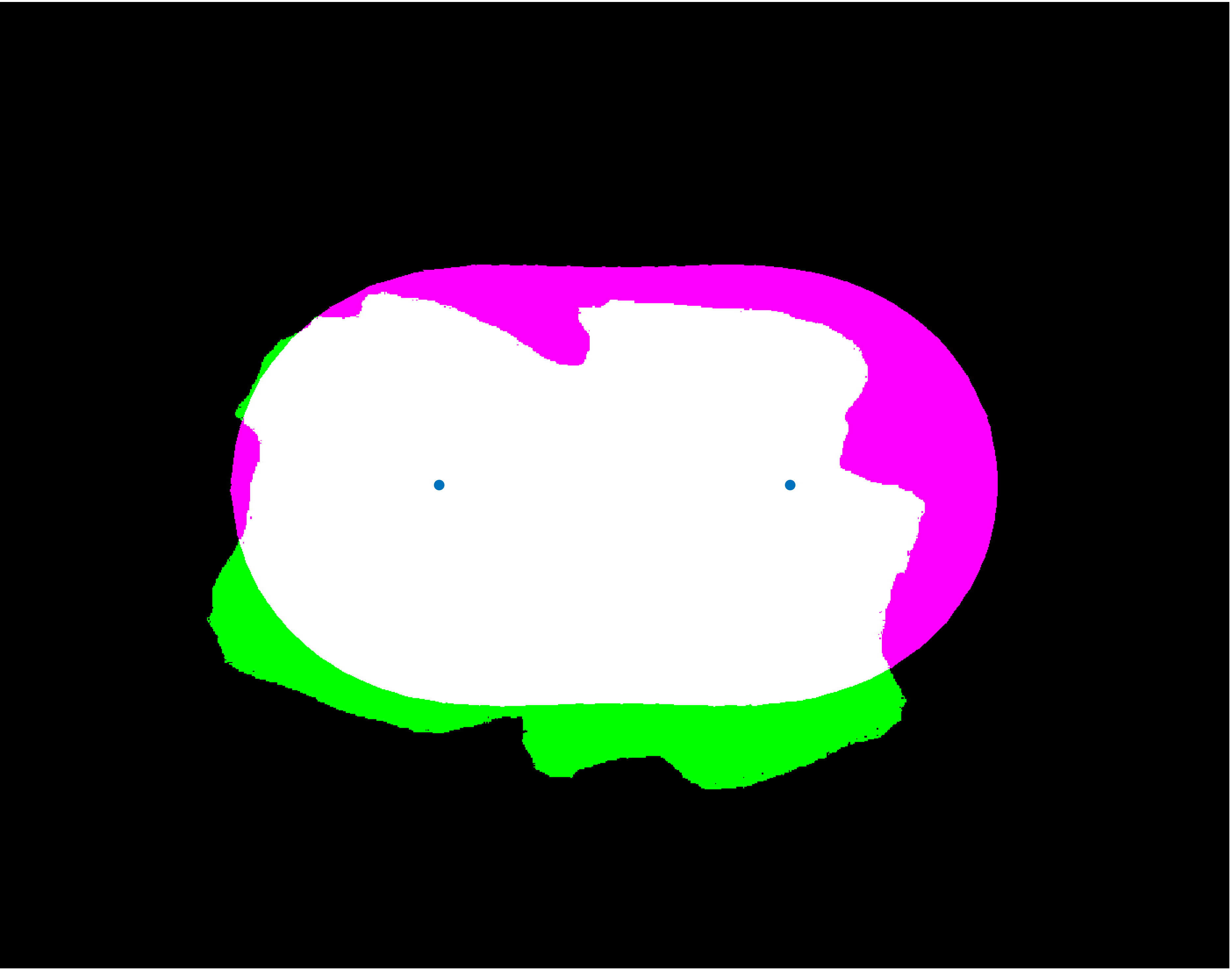

Lesion 4, Voltage=600, Angle=68.4, LET= 418, AR=3.09, EF=8.88, Dice=0.893

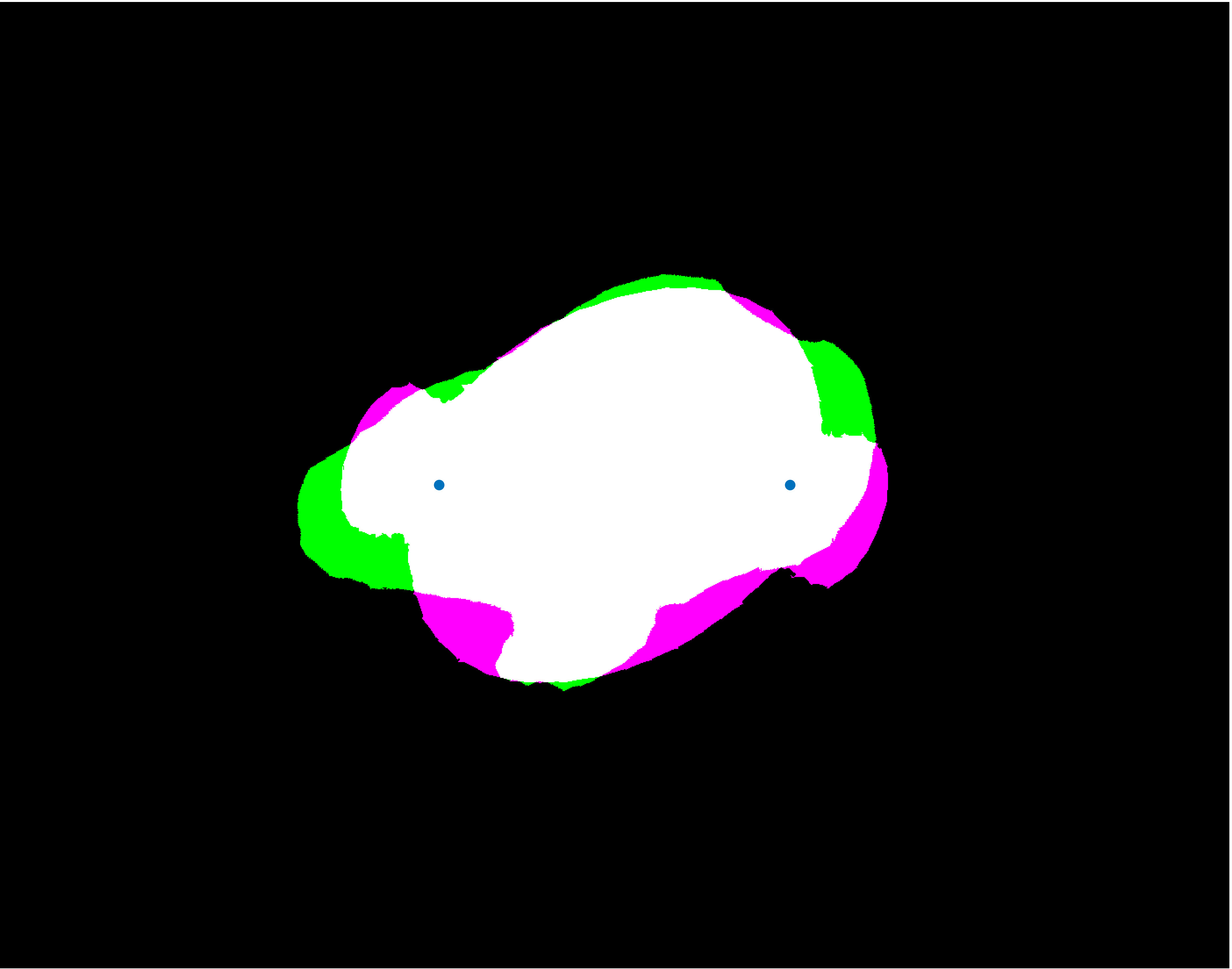

Lesion 5, Voltage=800, Angle=31.2, LET= 313, AR=1.40, EF=2.18, Dice=0.894

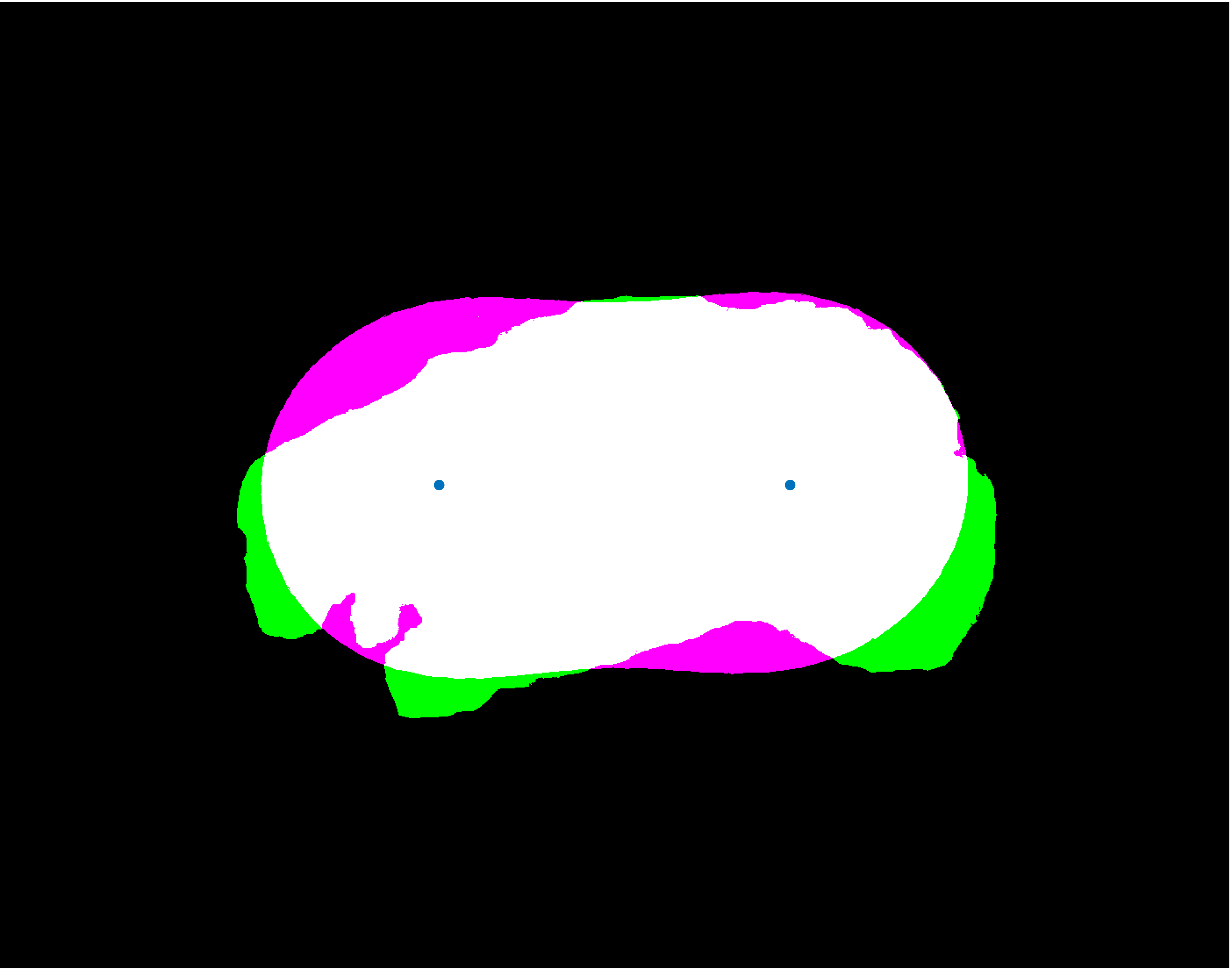

Lesion 6, Voltage=1200, Angle=19.9, LET= 171, AR=1.05, EF=3.02, Dice=0.871

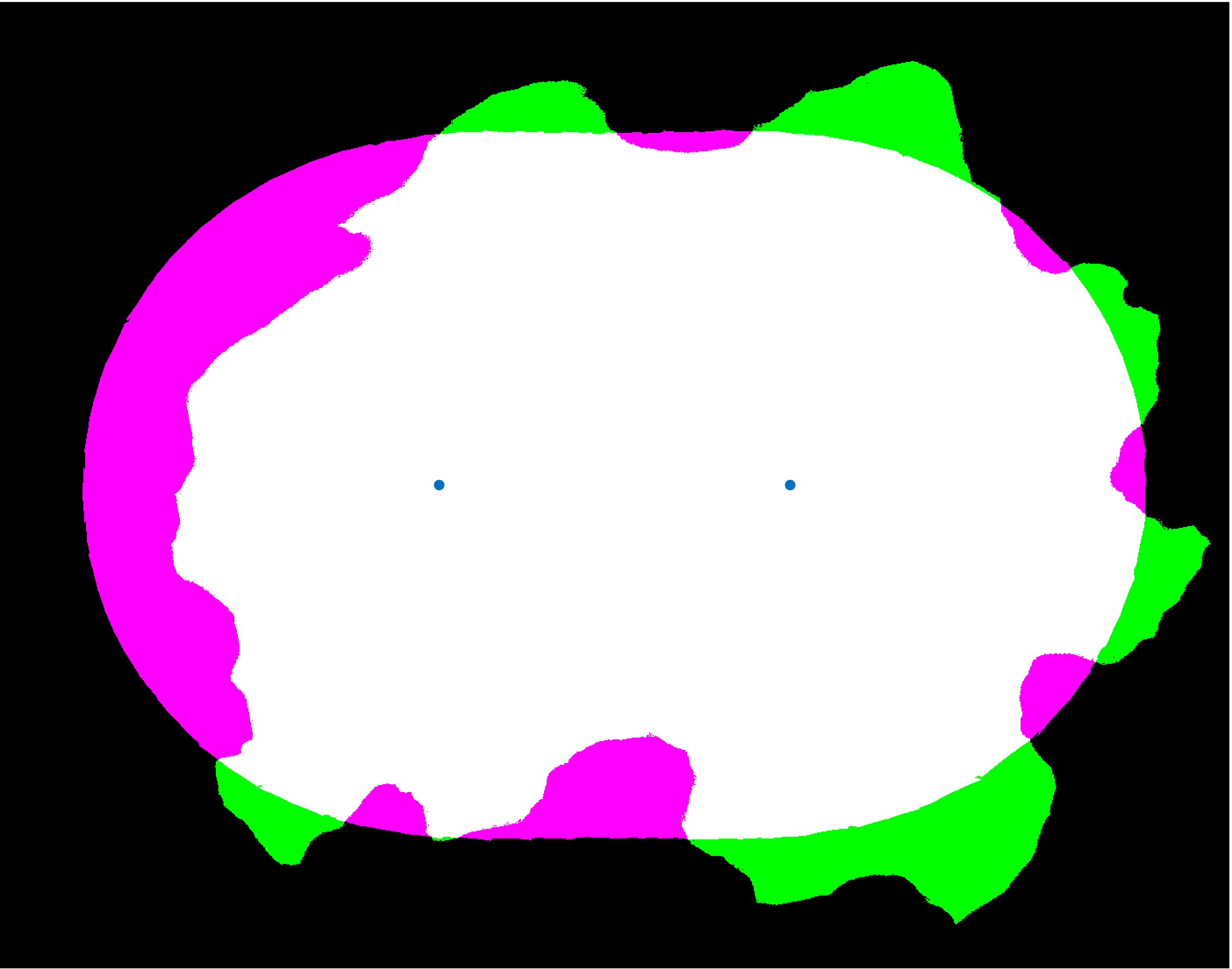

Lesion 7, Voltage=1000, Angle=-79.6, LET= 376, AR=3.20, EF=9.98, Dice=0.872

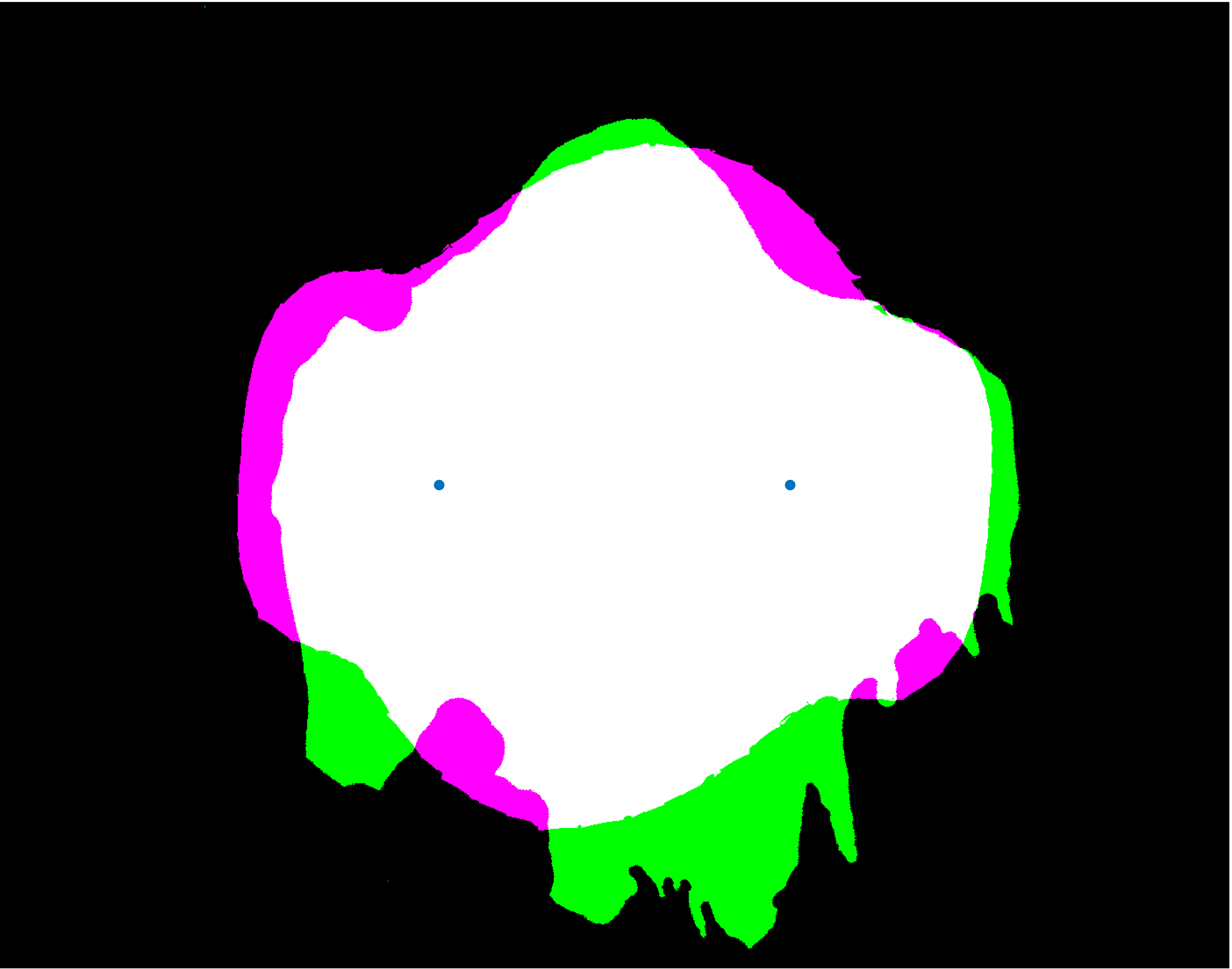

Lesion 8, Voltage=1200, Angle=62.5, LET= 419, AR=3.00, EF=2.05, Dice=0.858

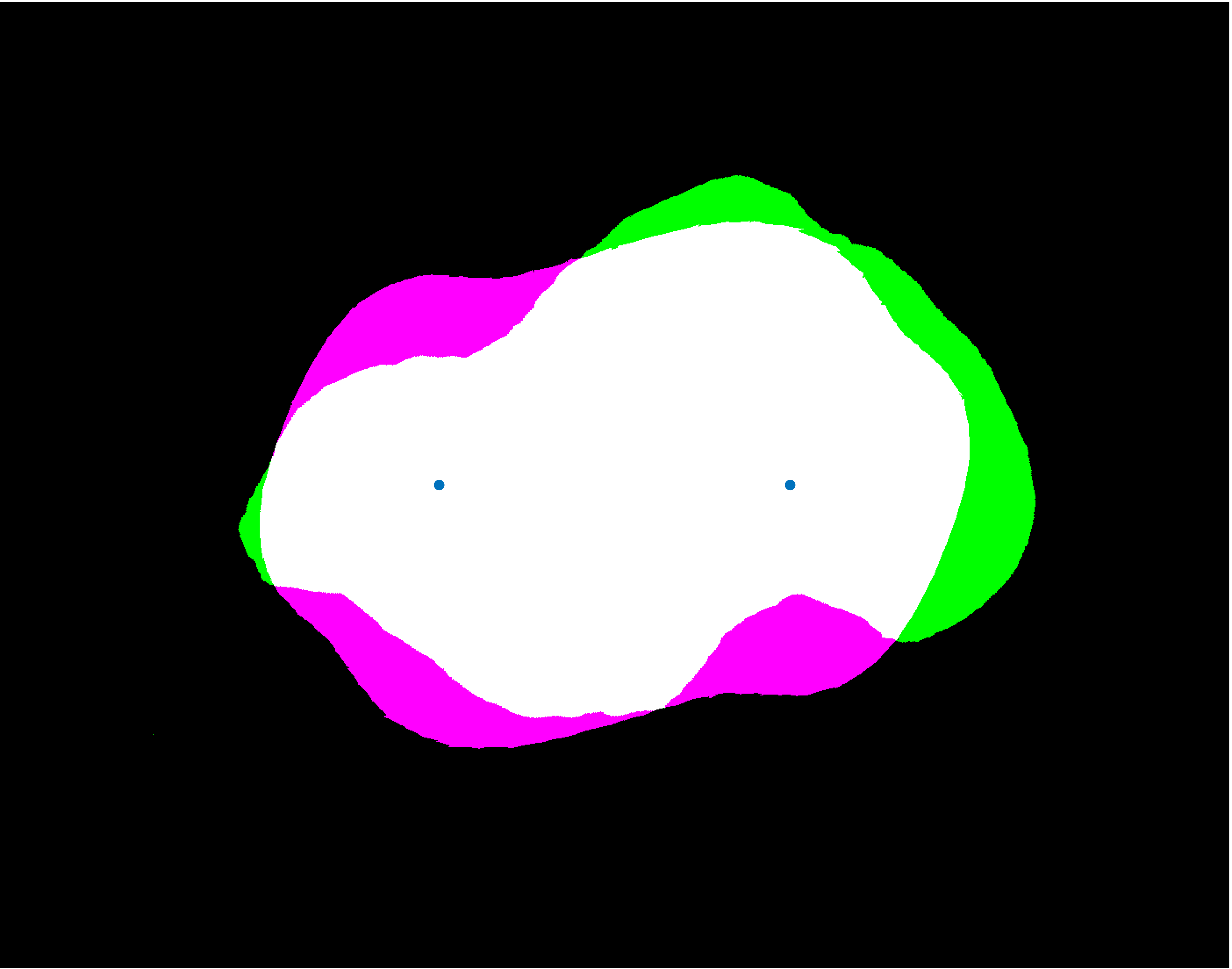

Lesion 9, Voltage=1000, Angle=88.4, LET= 477, AR=6.33, EF=1.95, Dice=0.920

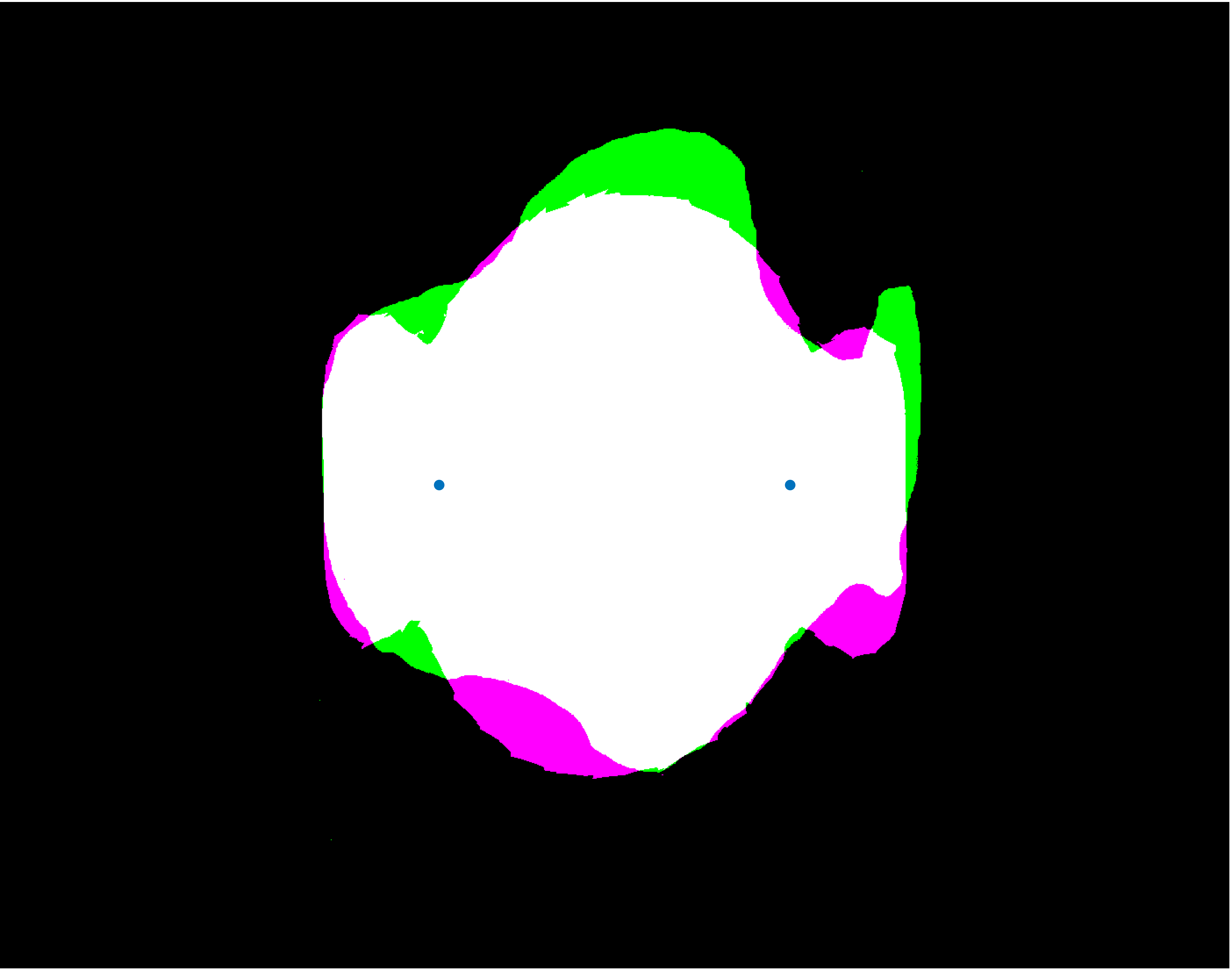

Lesion 10, Voltage=1200, Angle=56.7, LET= 321, AR=4.85, EF=1.09, Dice=0.912

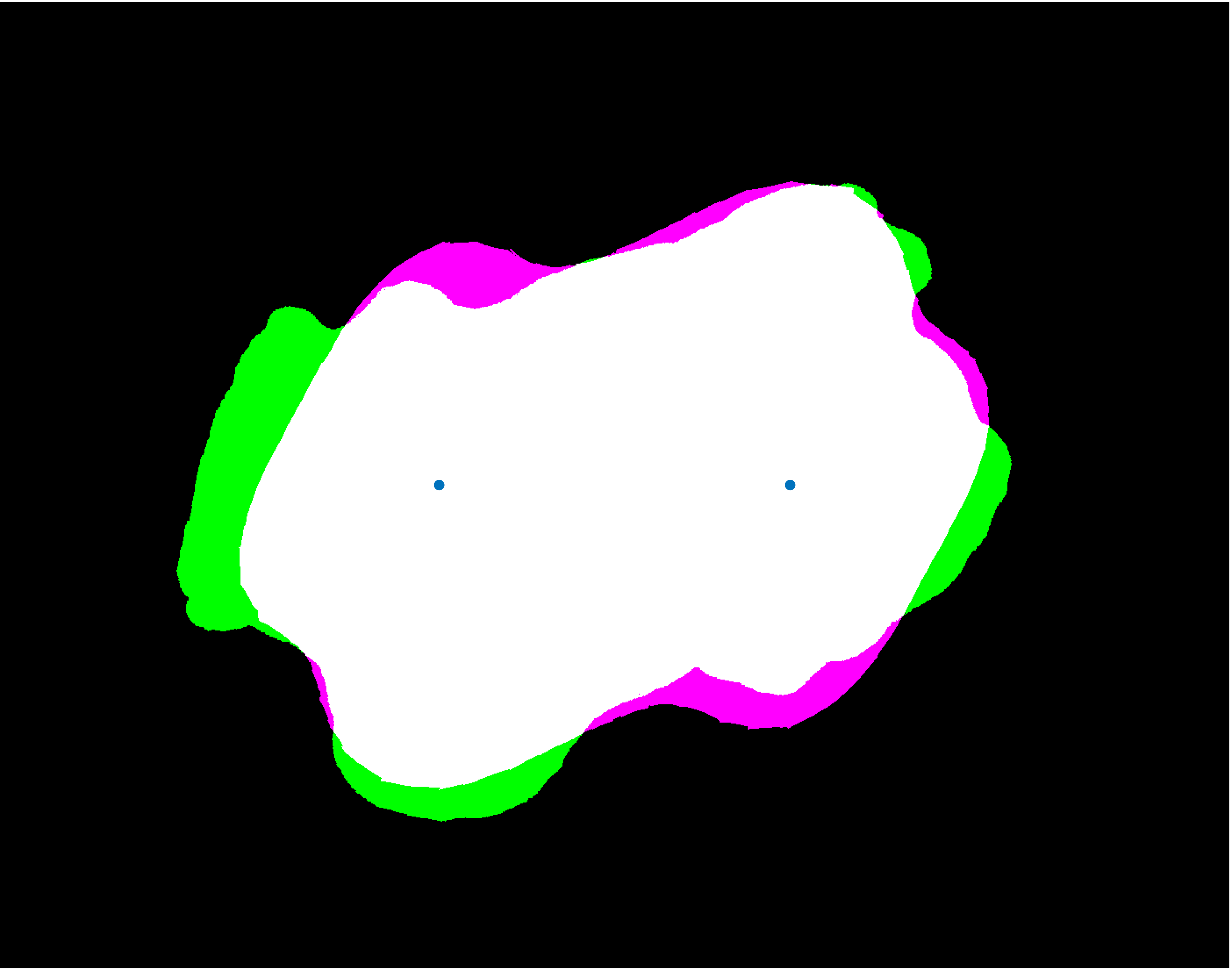

Lesion 11, Voltage=1200, Angle=77.0, LET= 215, AR=5.14, EF=1.05, Dice=0.945

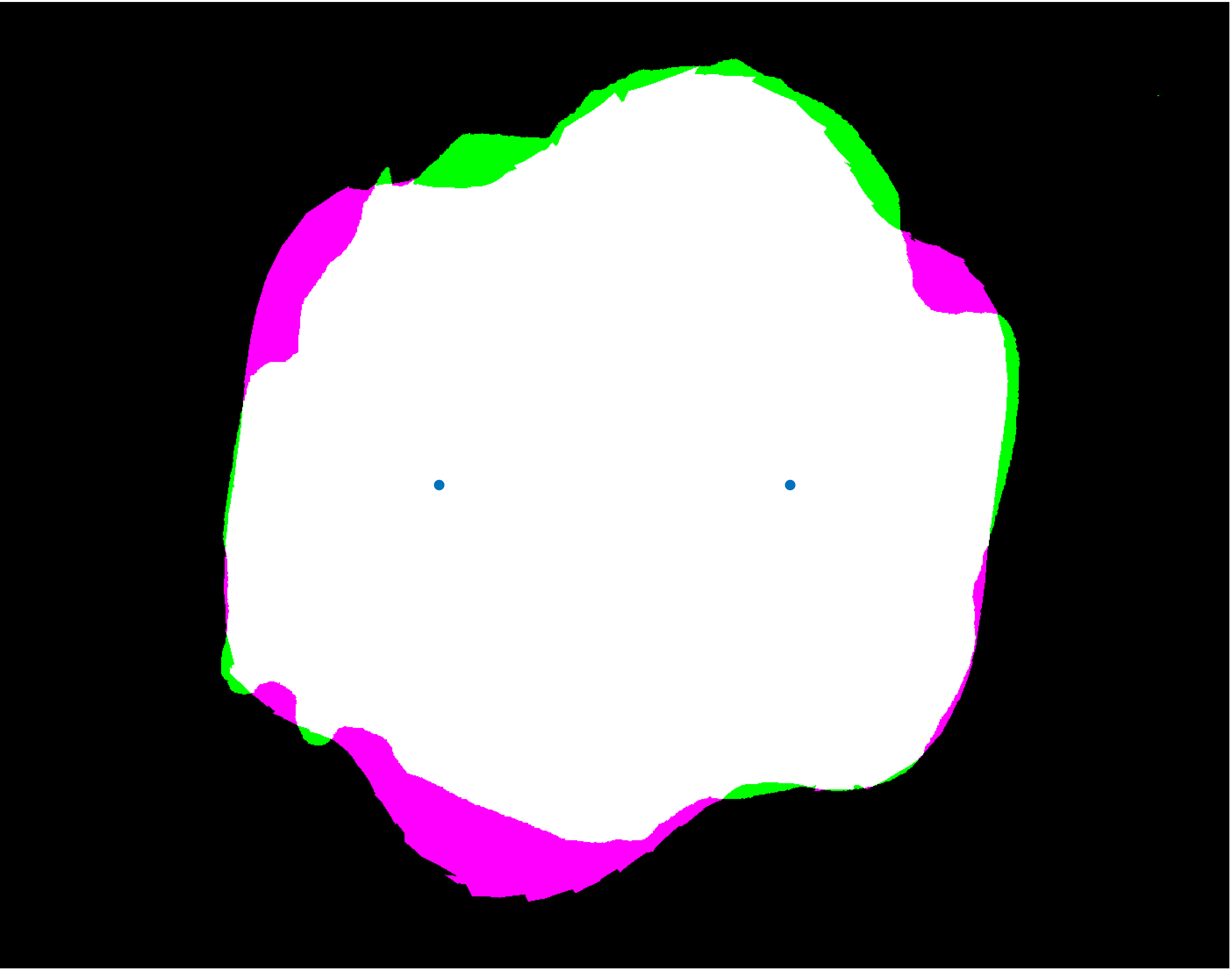

Lesion 12, Voltage=600, Angle=66.6, LET= 224, AR=2.90, EF=1.02, Dice=0.912

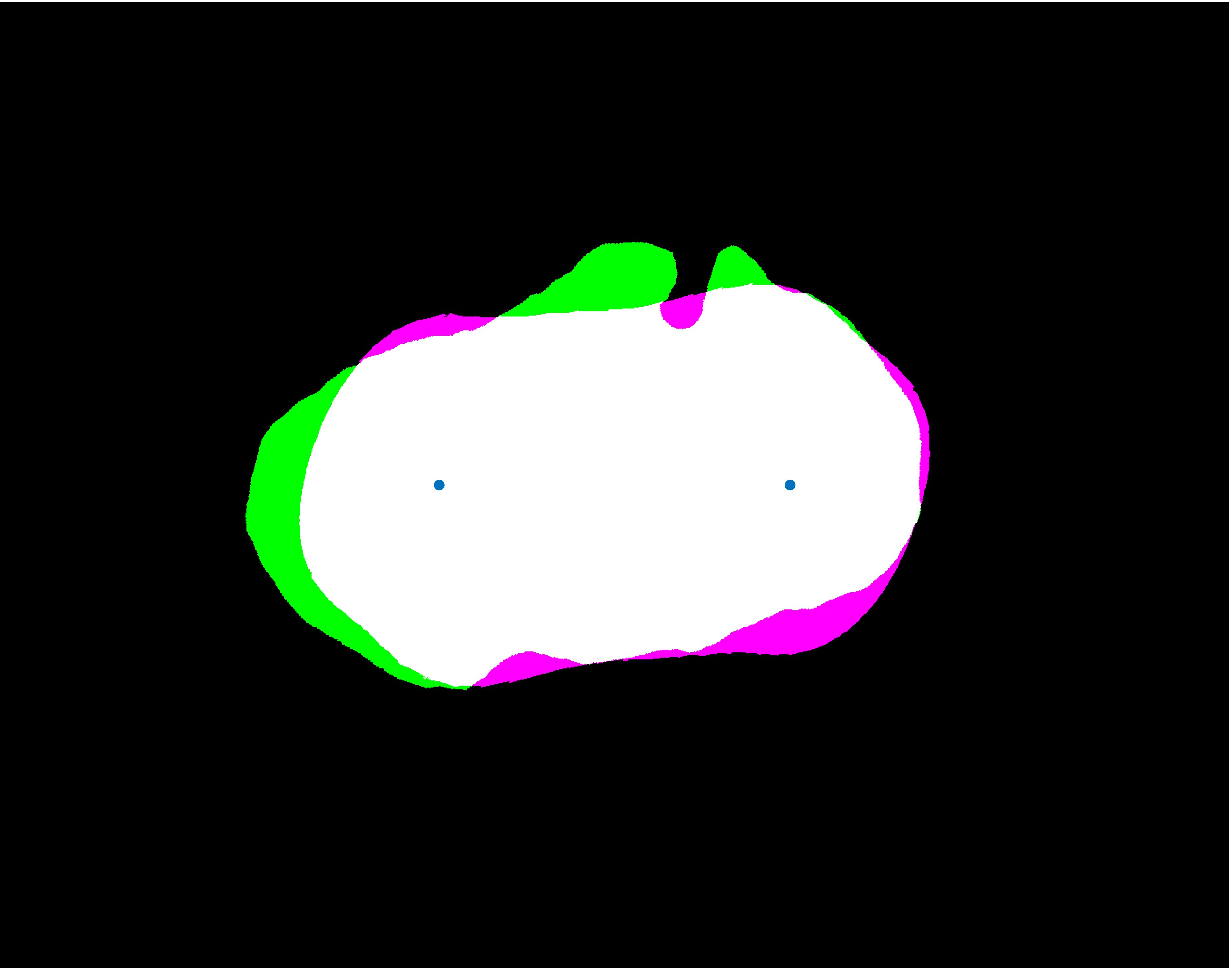

Lesion 13, Voltage=800, Angle=80.1, LET= 389, AR=2.66, EF=2.81, Dice=0.953

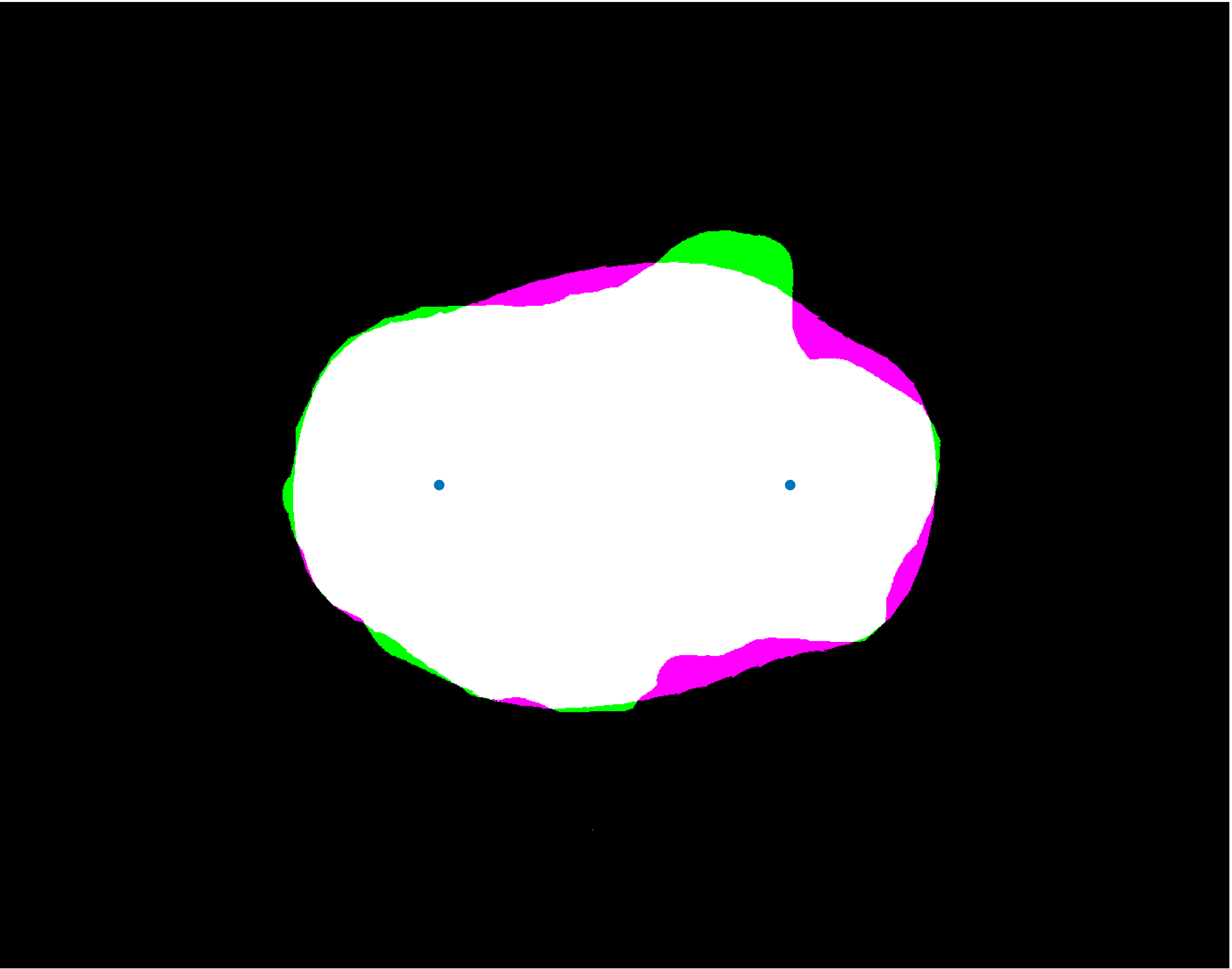

Lesion 14, Voltage=1000, Angle=86.1, LET= 368, AR=4.30, EF=3.58, Dice=0.874

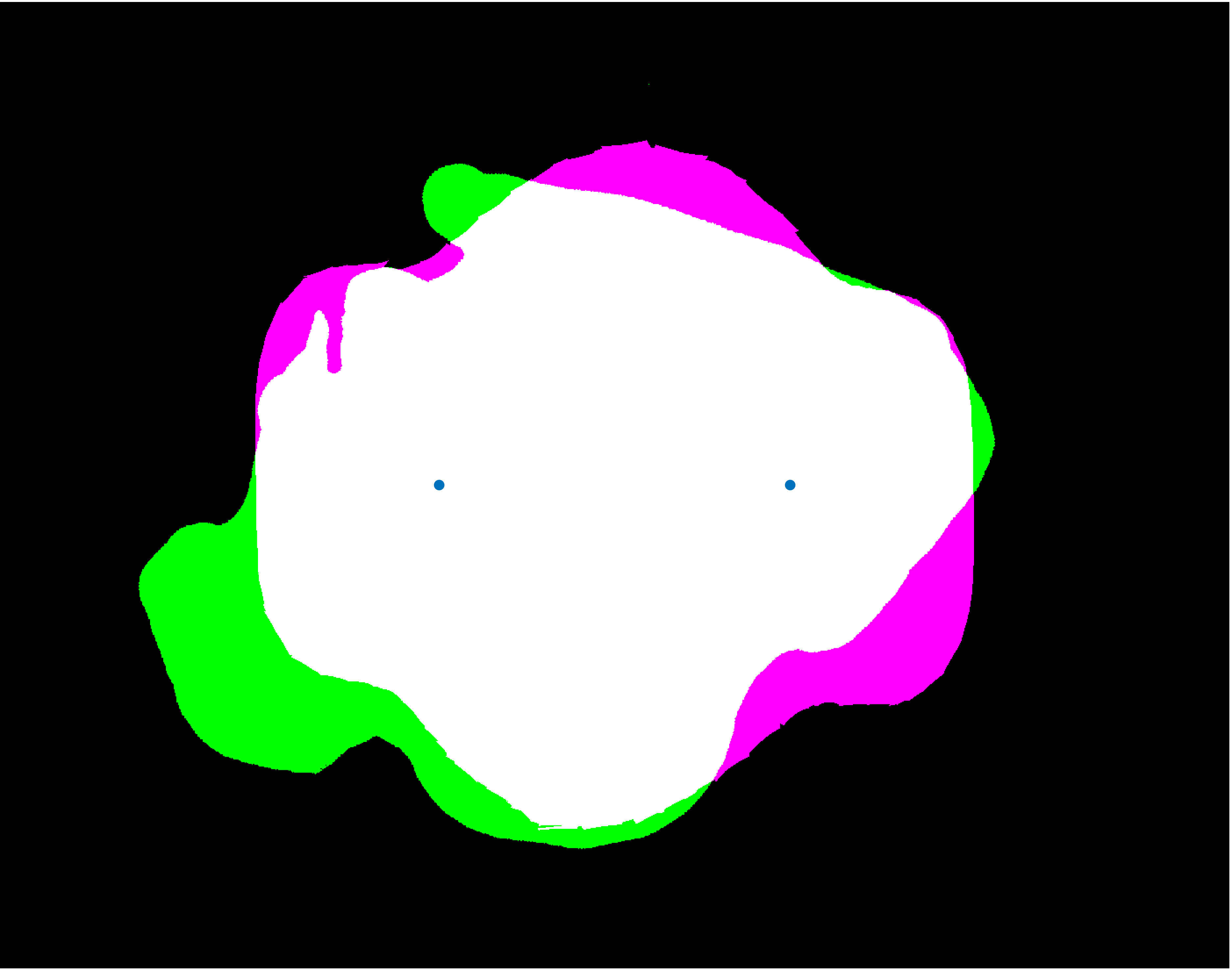

Lesion 15, Voltage=600, Angle=1.8, LET= 261, AR=1.86, EF=1.02, Dice=0.892

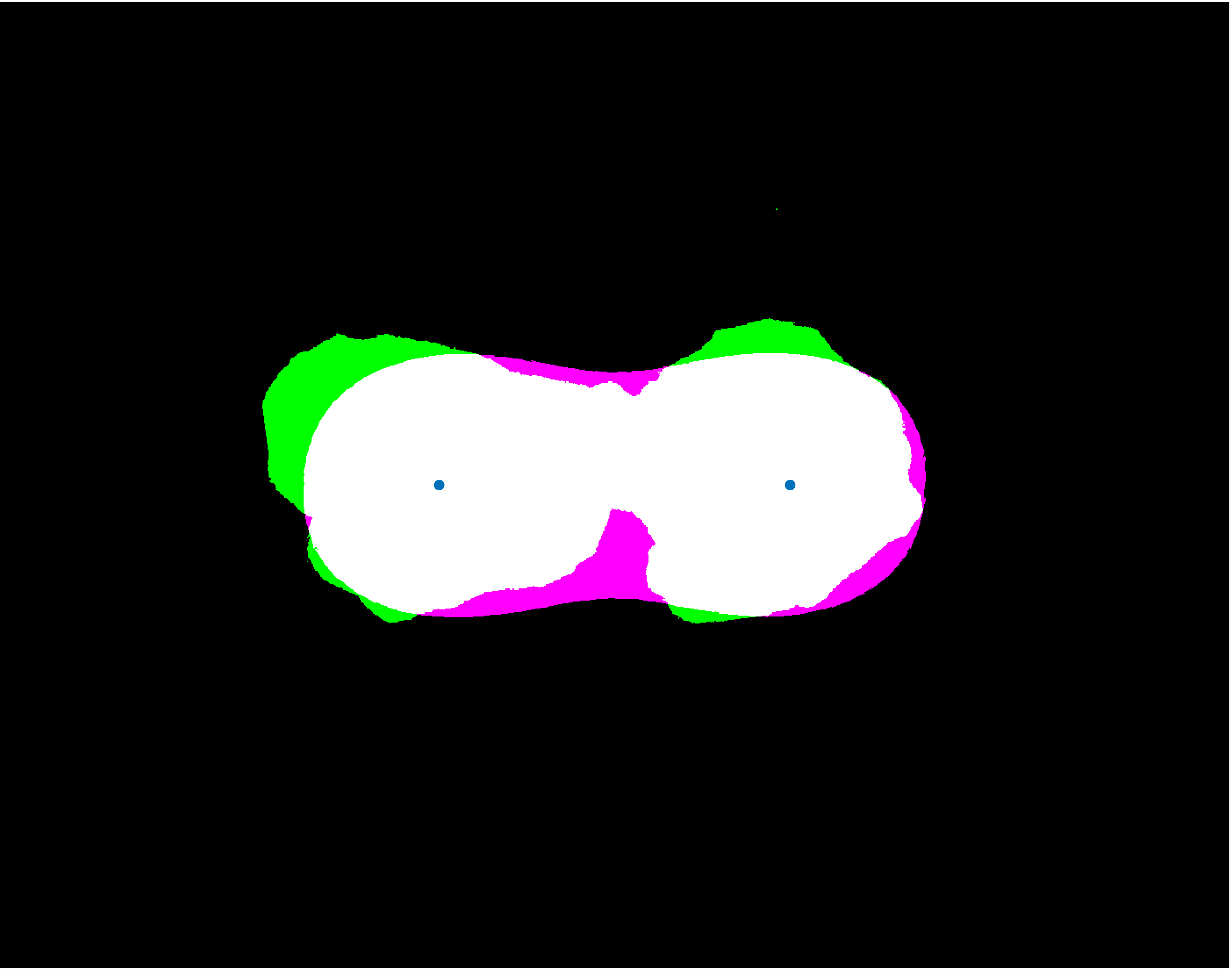

Lesion 16, Voltage=600, Angle=15.7, LET= 402, AR=1.94, EF=2.46, Dice=0.862

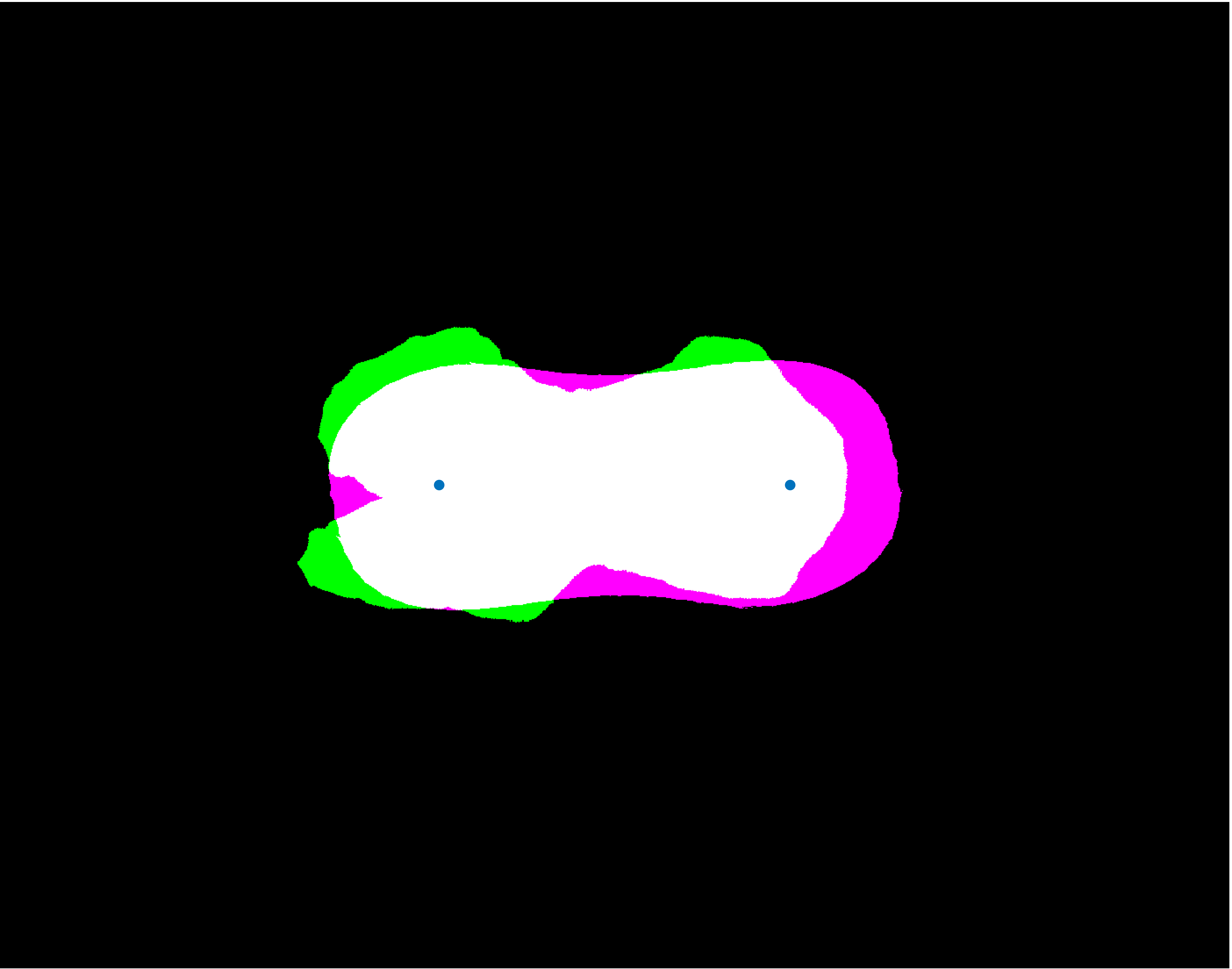

Lesion 17, Voltage=800, Angle=9.6, LET= 365, AR=1.06, EF=3.03, Dice=0.895

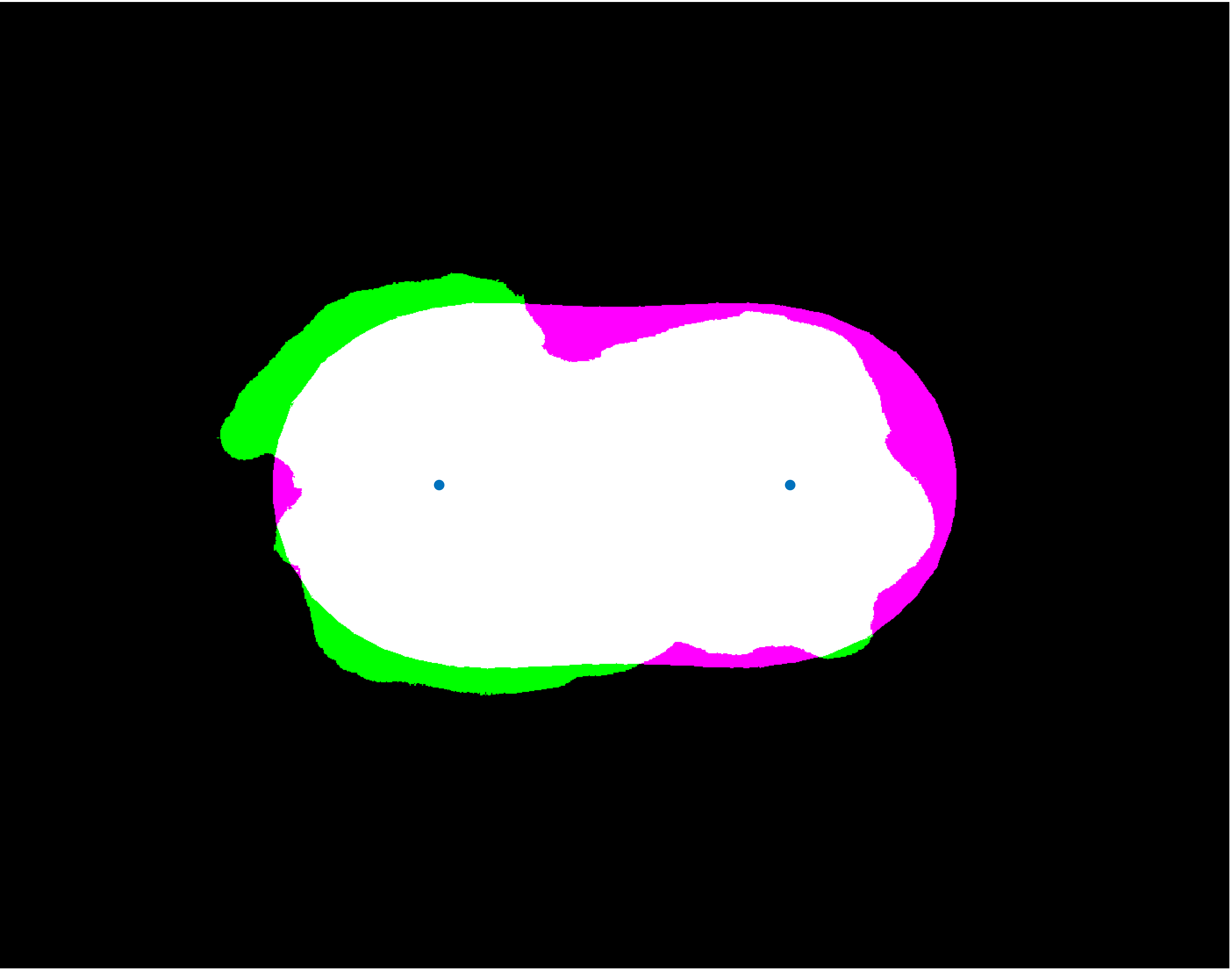

Lesion 18, Voltage=800, Angle=3.8, LET= 400, AR=2.46, EF=7.65, Dice=0.909

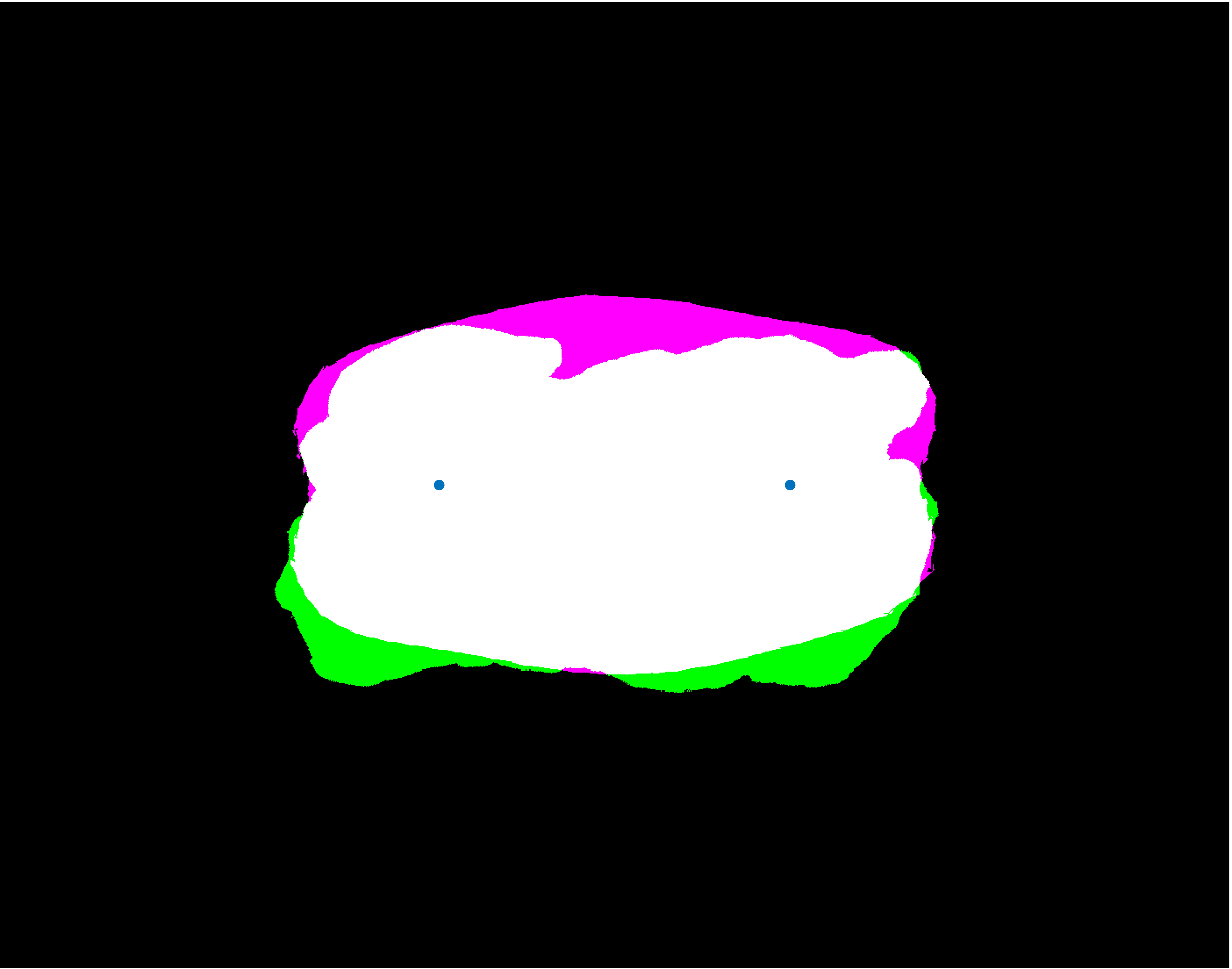

Lesion 19, Voltage=600, Angle=9.6, LET= 307, AR=1.24, EF=3.14, Dice=0.852

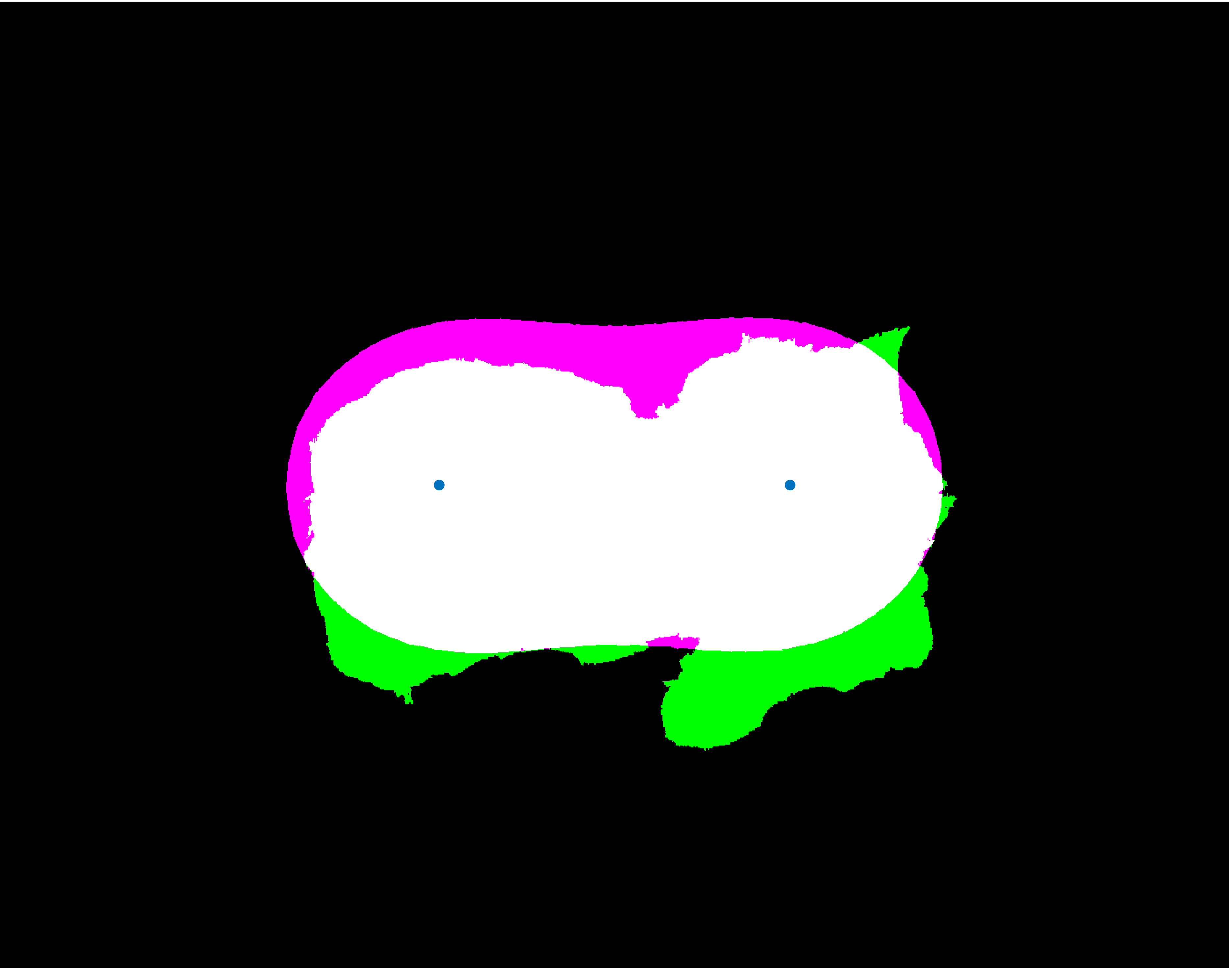

Lesion 20, Voltage=600, Angle=12.4, LET= 340, AR=1.95, EF=6.23, Dice=0.897

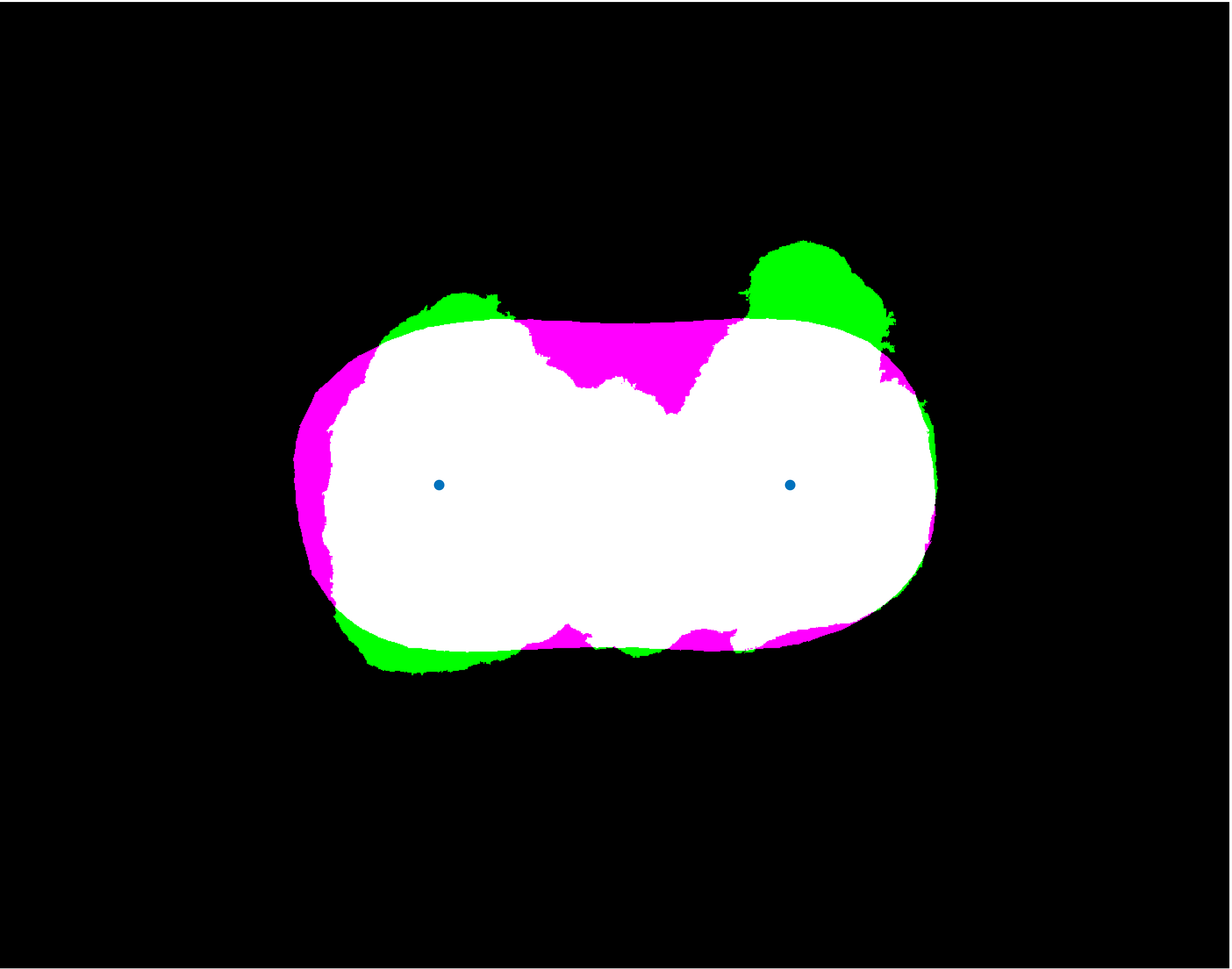

Lesion 21, Voltage=800, Angle=6.1, LET= 264, AR=2.03, EF=1.12, Dice=0.937

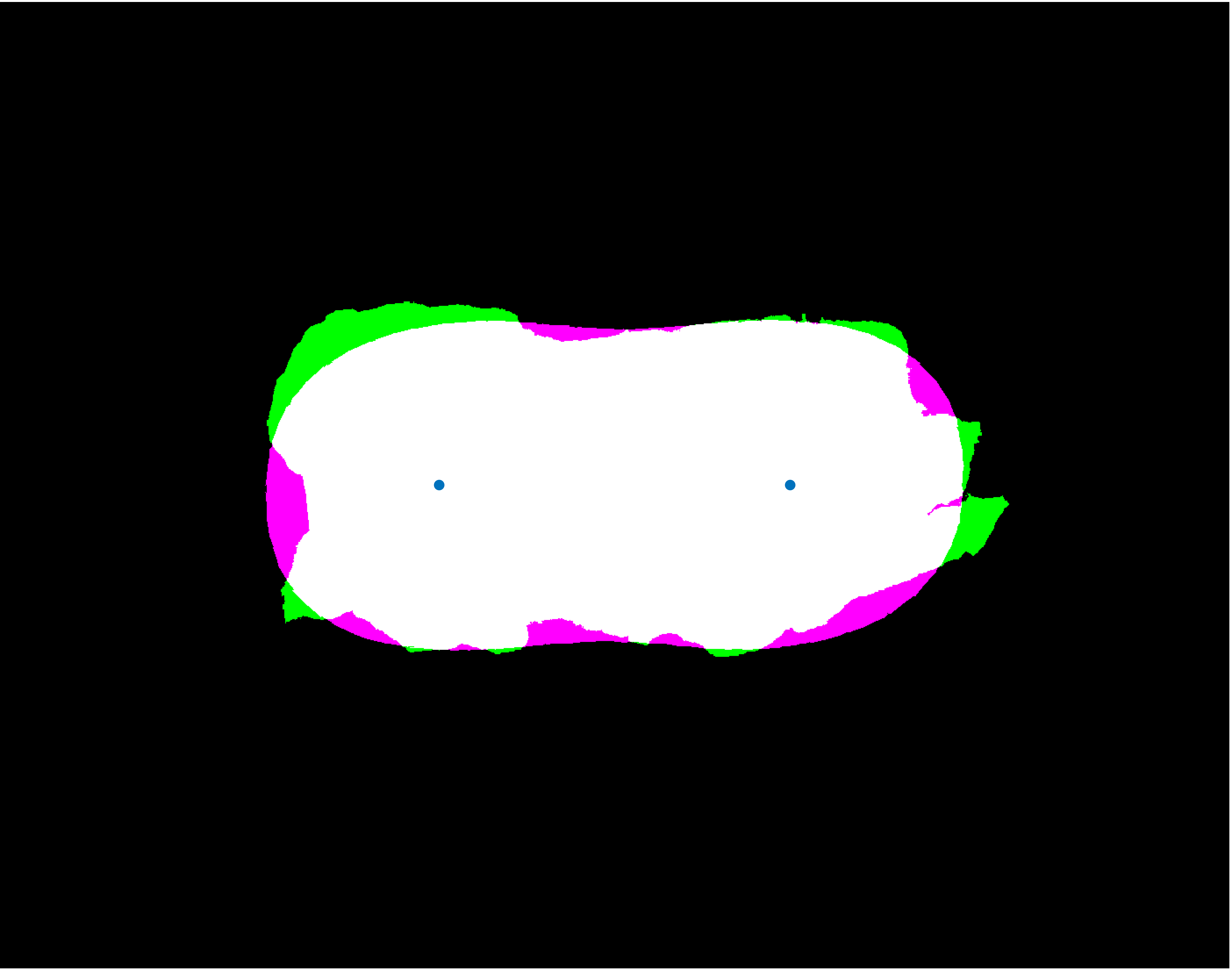

Lesion 22, Voltage=800, Angle=-3.0, LET= 461, AR=1.24, EF=3.14, Dice=0.923

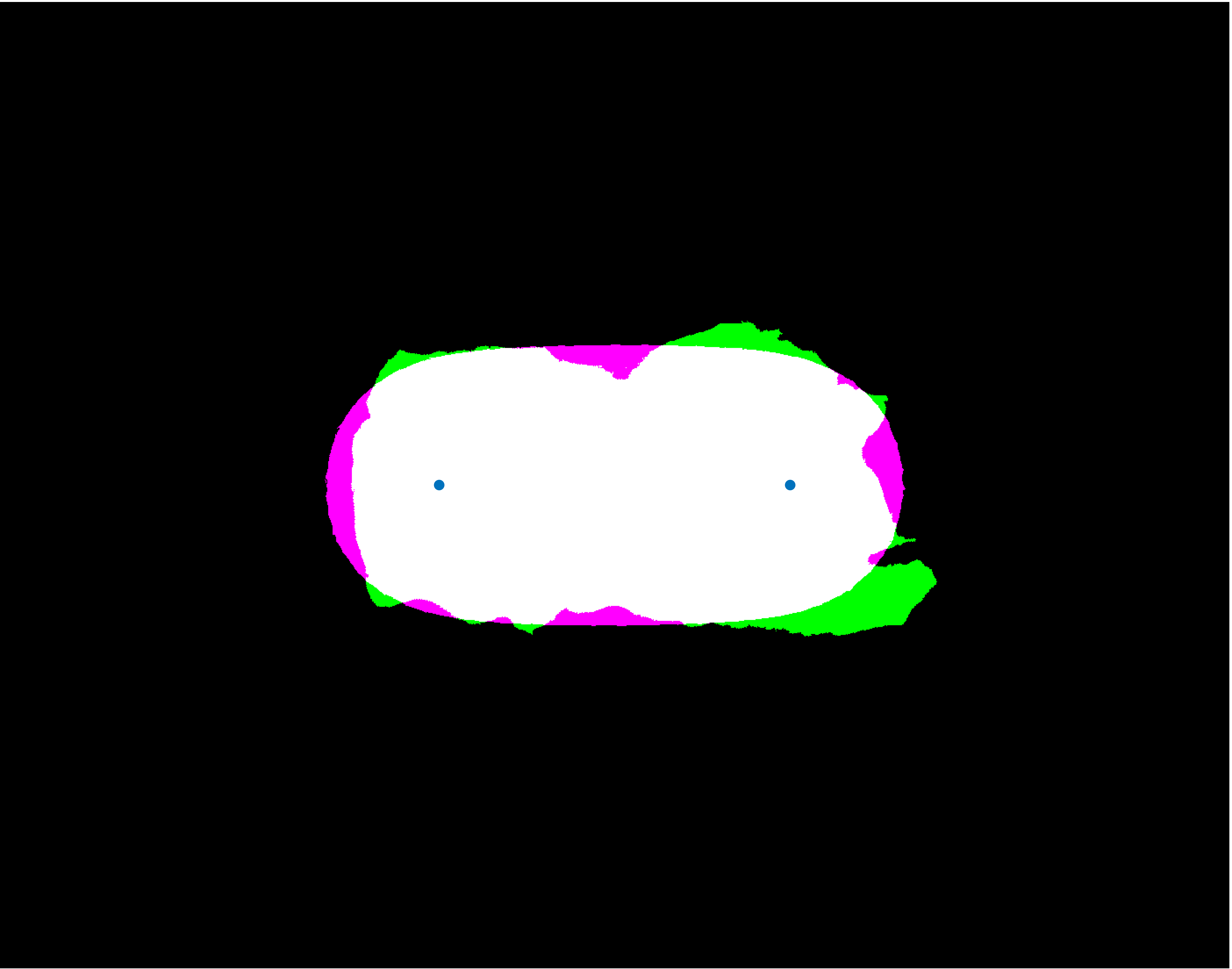

Lesion 23, Voltage=600, Angle=12.8, LET= 290, AR=3.25, EF=1.01, Dice=0.899

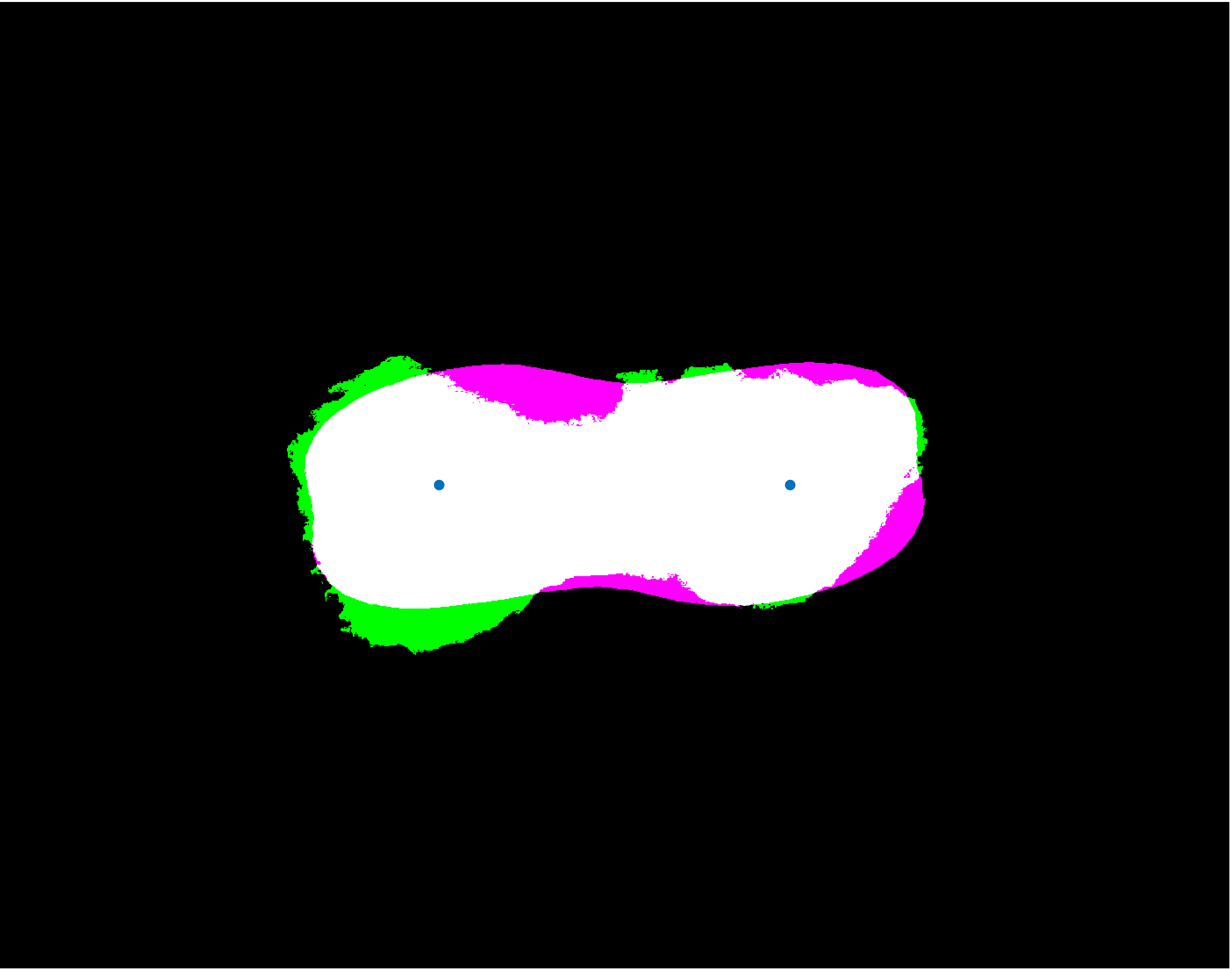

Lesion 24, Voltage=800, Angle=26.1, LET= 307, AR=1.22, EF=1.07, Dice=0.892

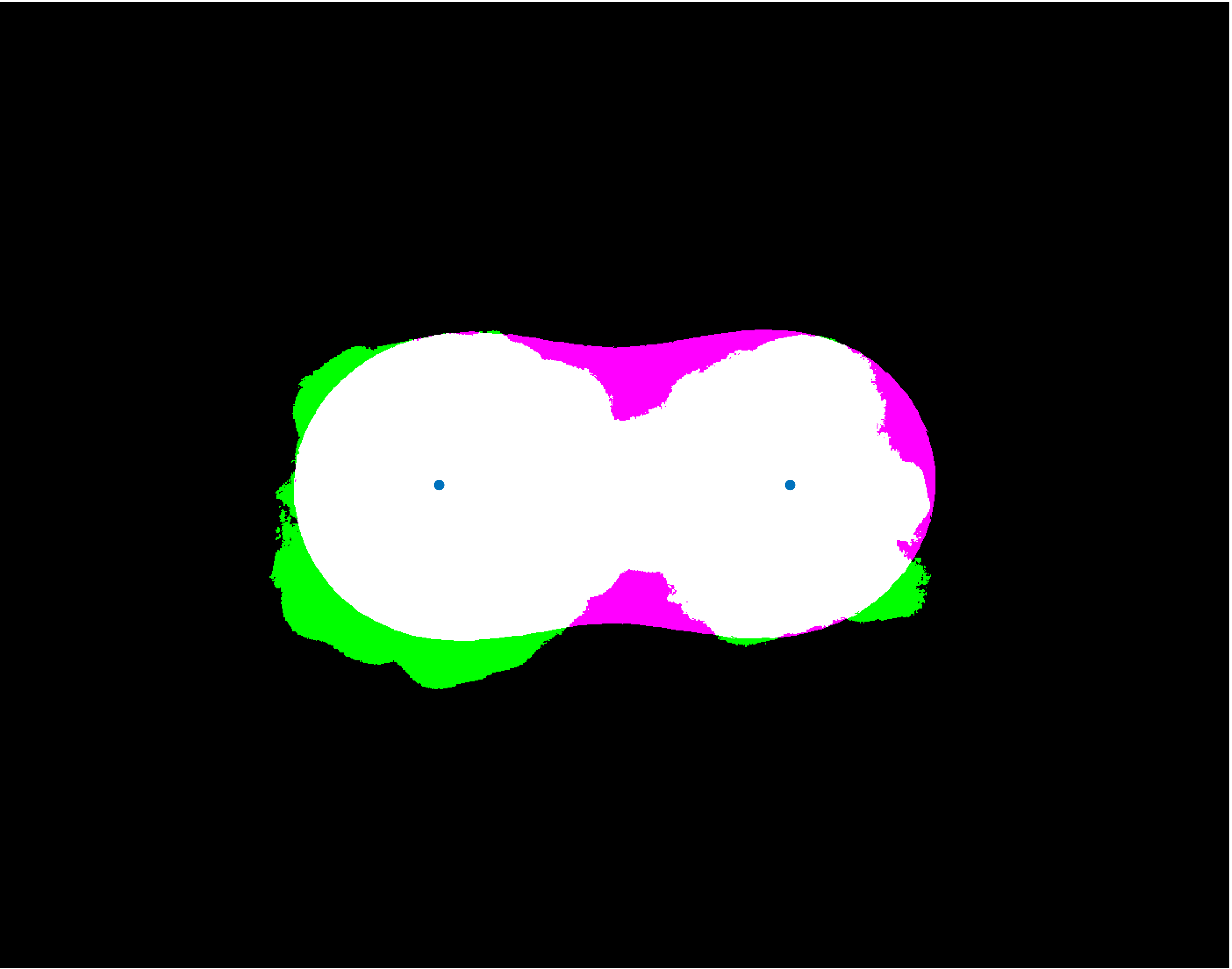

Lesion 25, Voltage=1000, Angle=-86.7, LET= 478, AR=3.01, EF=2.80, Dice=0.849

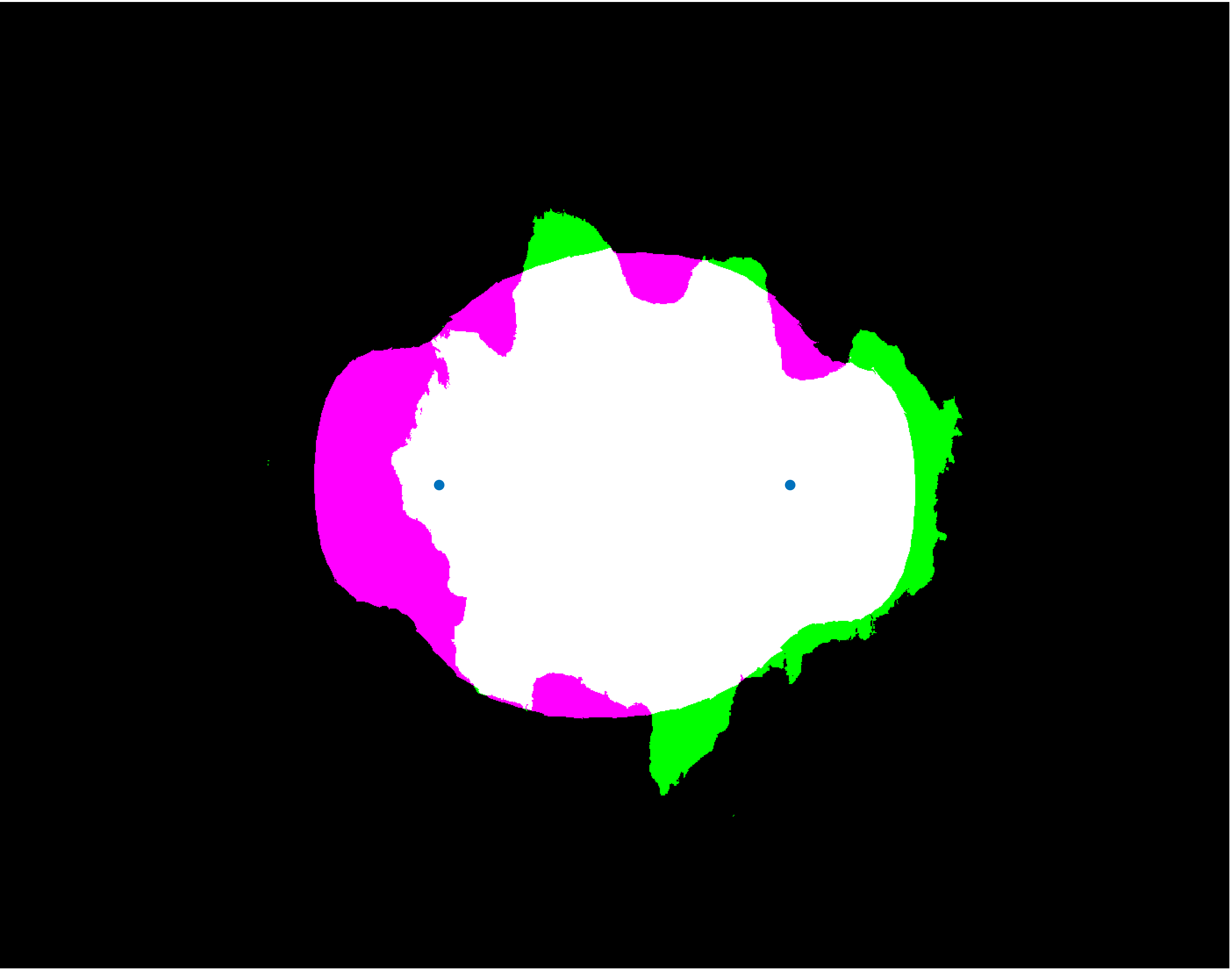

Lesion 26, Voltage=1000, Angle=41.3, LET= 444, AR=3.19, EF=4.25, Dice=0.881

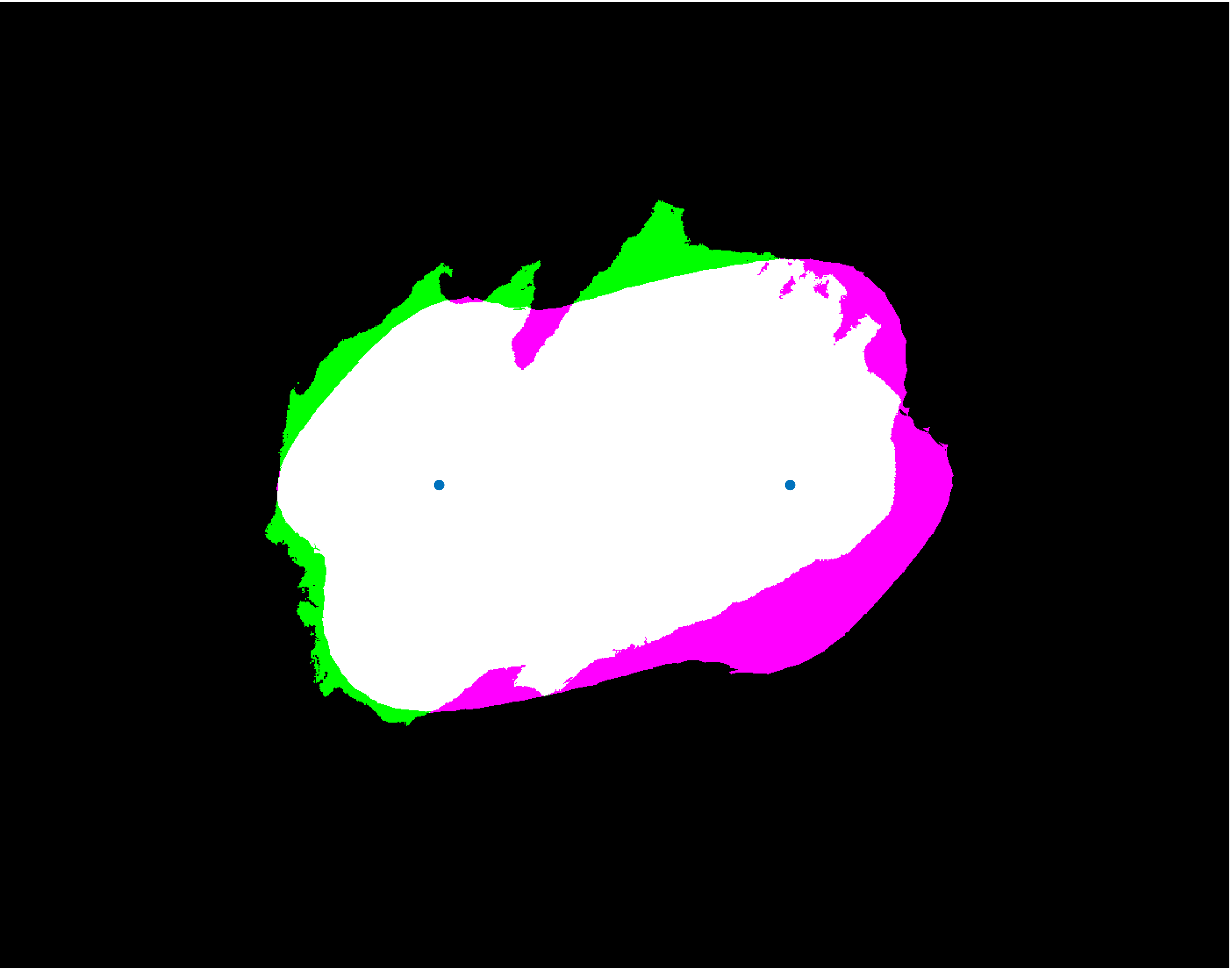

Lesion 27, Voltage=800, Angle=-50.3, LET= 432, AR=2.28, EF=2.08, Dice=0.917

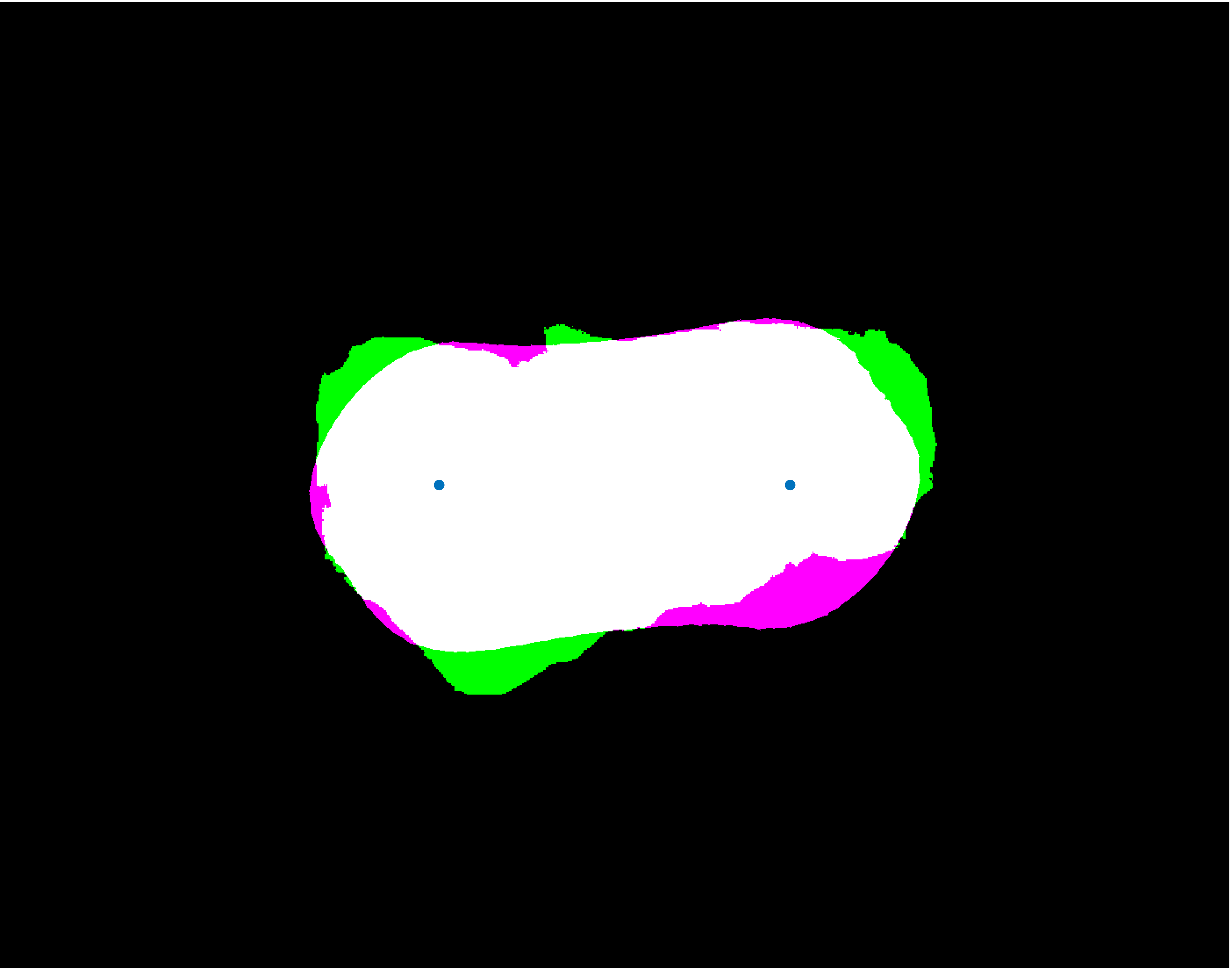

Lesion 28, Voltage=600, Angle=-18.4, LET= 359, AR=4.97, EF=8.47, Dice=0.807

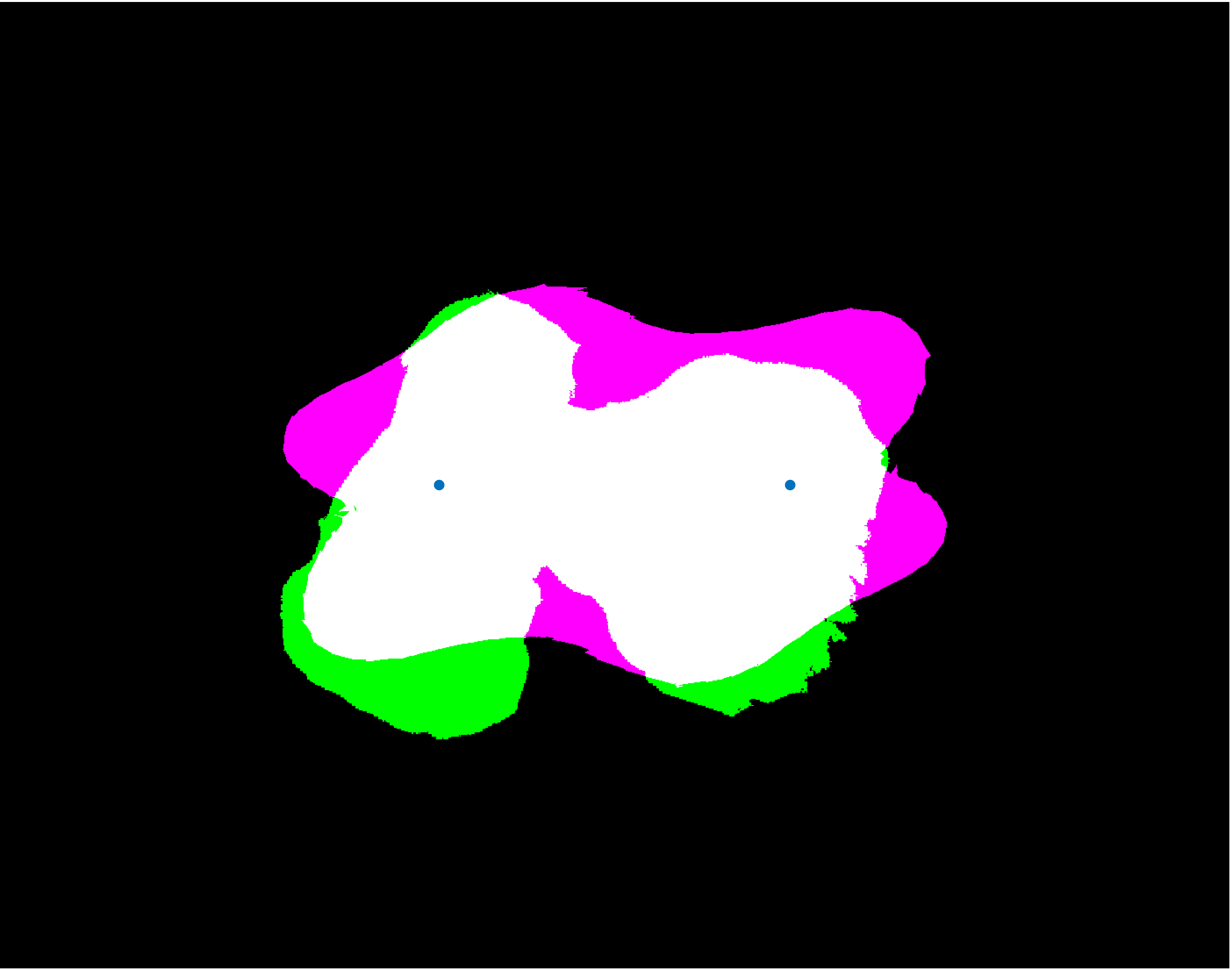

Lesion 29, Voltage=600, Angle=-27.3, LET= 424, AR=5.70, EF=9.95, Dice=0.893

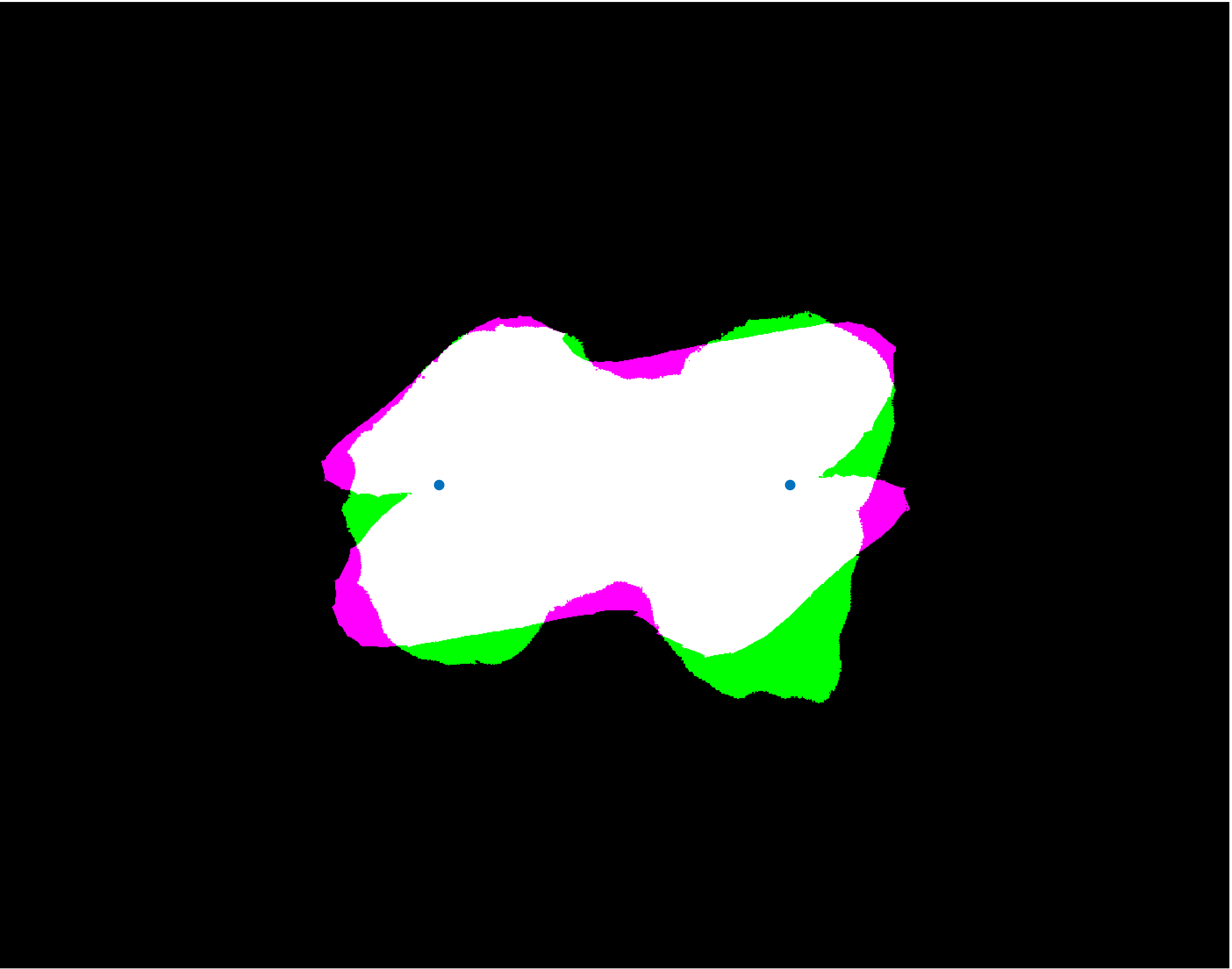

Lesion 30, Voltage=800, Angle=74.2, LET= 462, AR=1.58, EF=2.64, Dice=0.892

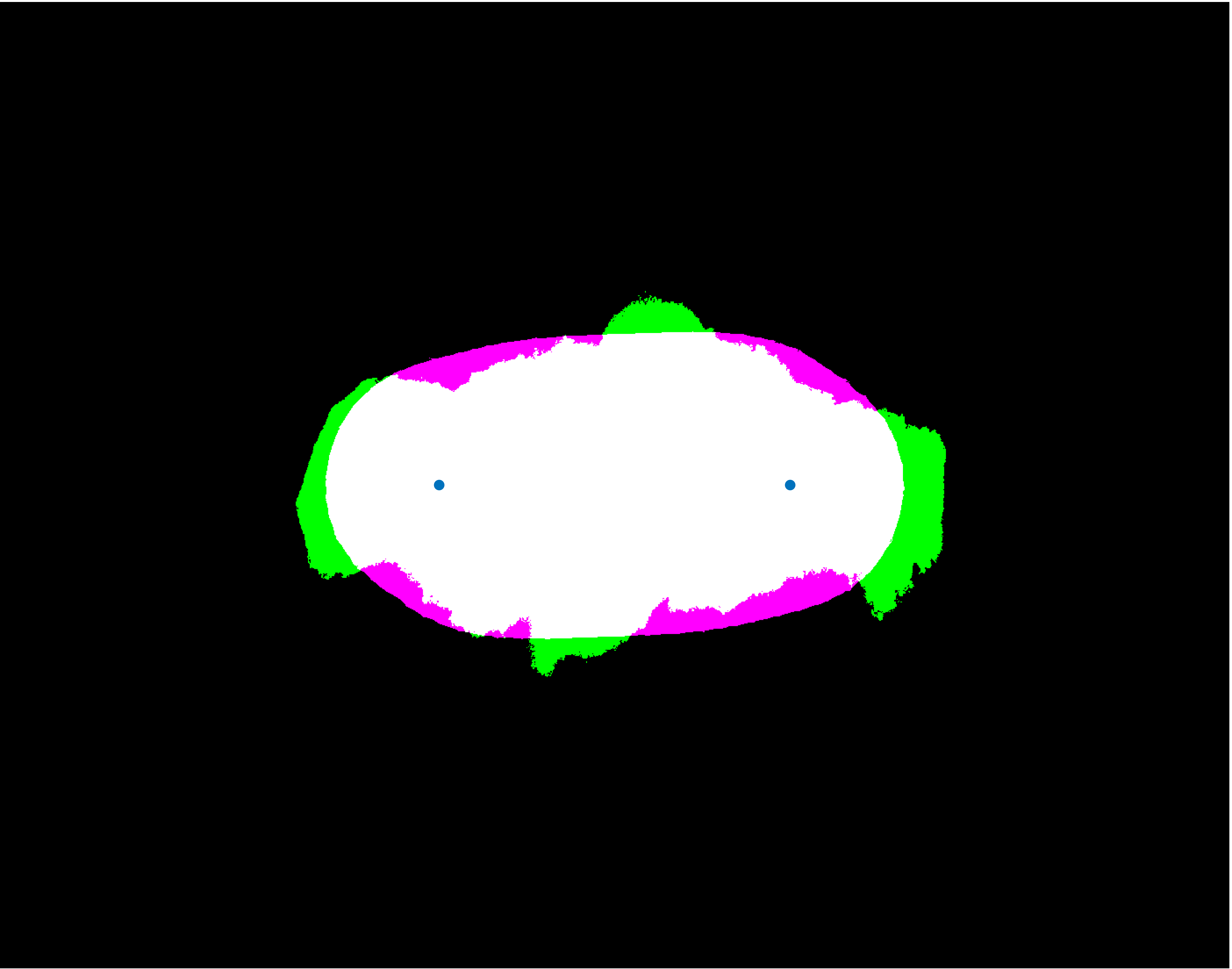

Lesion 31, Voltage=800, Angle=60.5, LET= 493, AR=2.21, EF=2.68, Dice=0.924

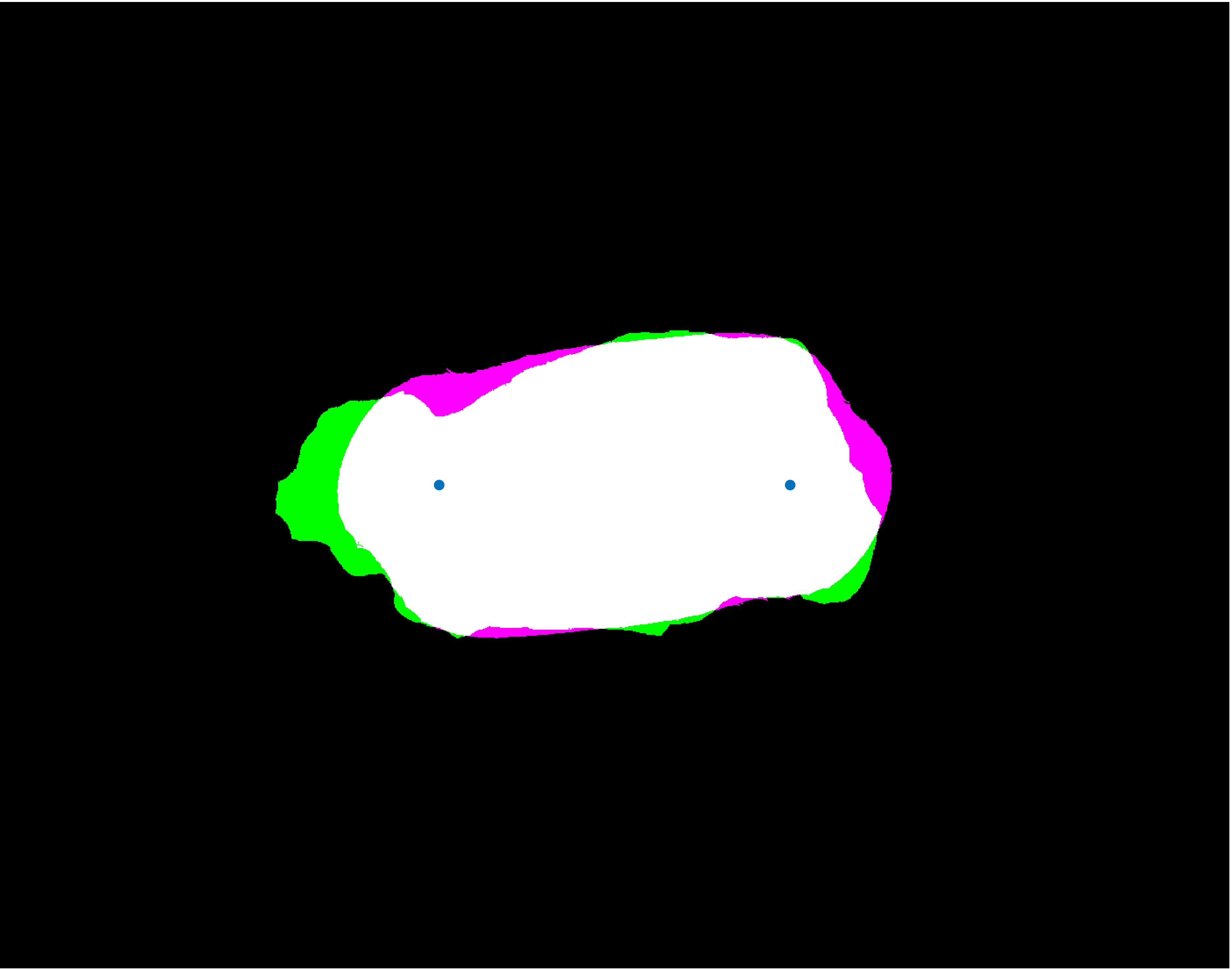

Supplement: Supplementary file 1 [file Datasheet1.pdf]
