# Supplementary figures and images for: Determination of lethal electric field threshold for pulsed field ablation in ex vivo perfused porcine and human hearts (part 2 of 2)
Source: Front Cardiovasc Med. 2023 Jun 23;10:1160231. doi: 10.3389/fcvm.2023.1160231 (PMC10326317; doi:10.3389/fcvm.2023.1160231)

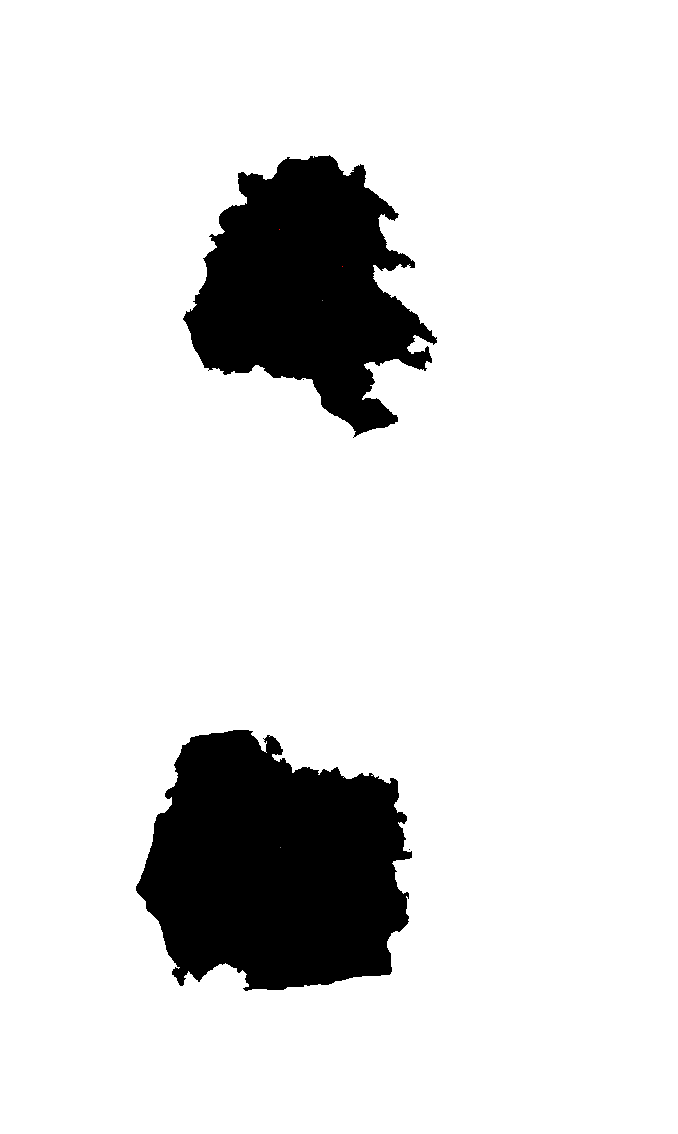

Supplement: Supplementary file 2 [file Datasheet2.zip › figshare/ImageIn/Experiment_101.tif]

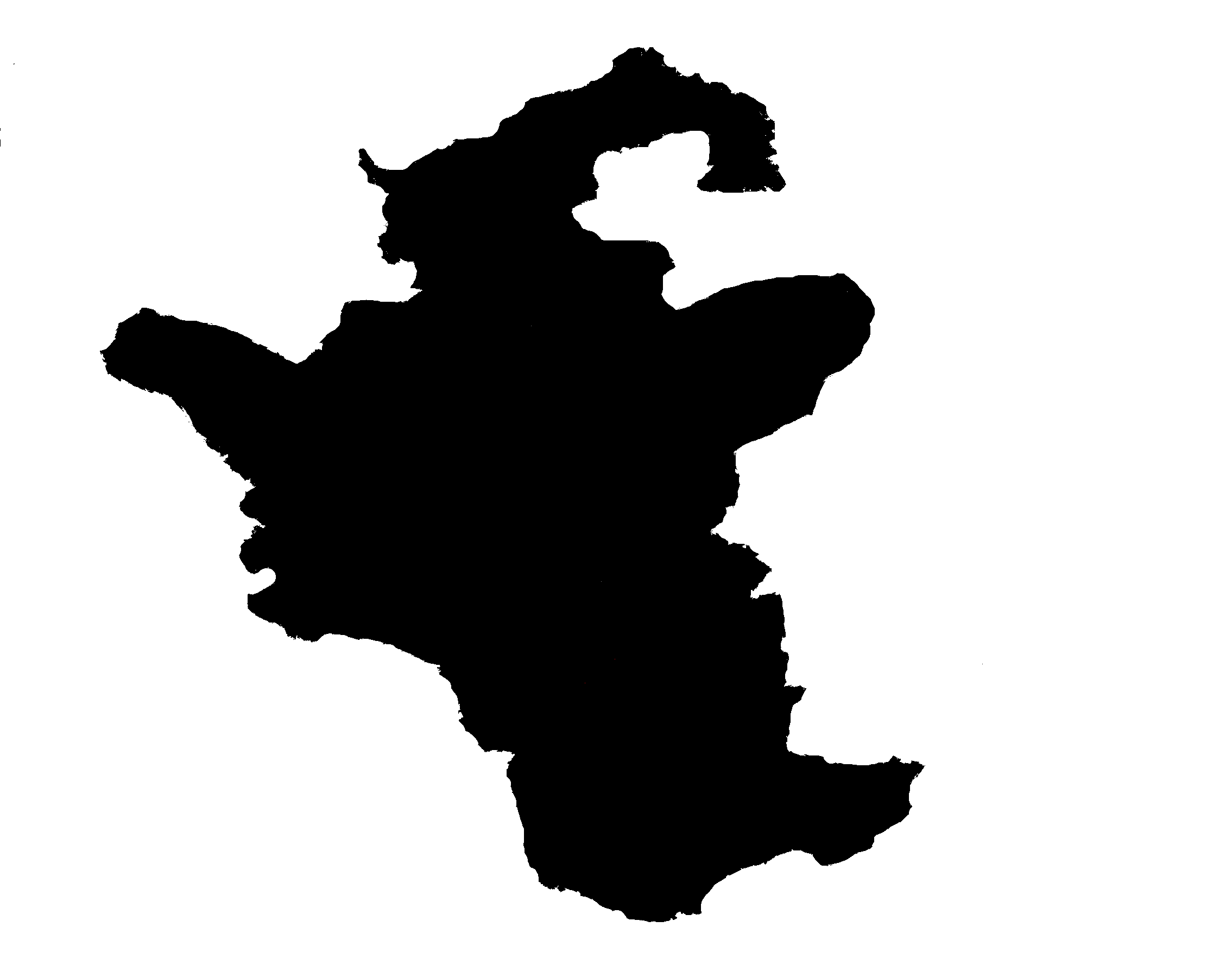

Supplement: Supplementary file 2 [file Datasheet2.zip › figshare/ImageIn/Experiment_102.tif]

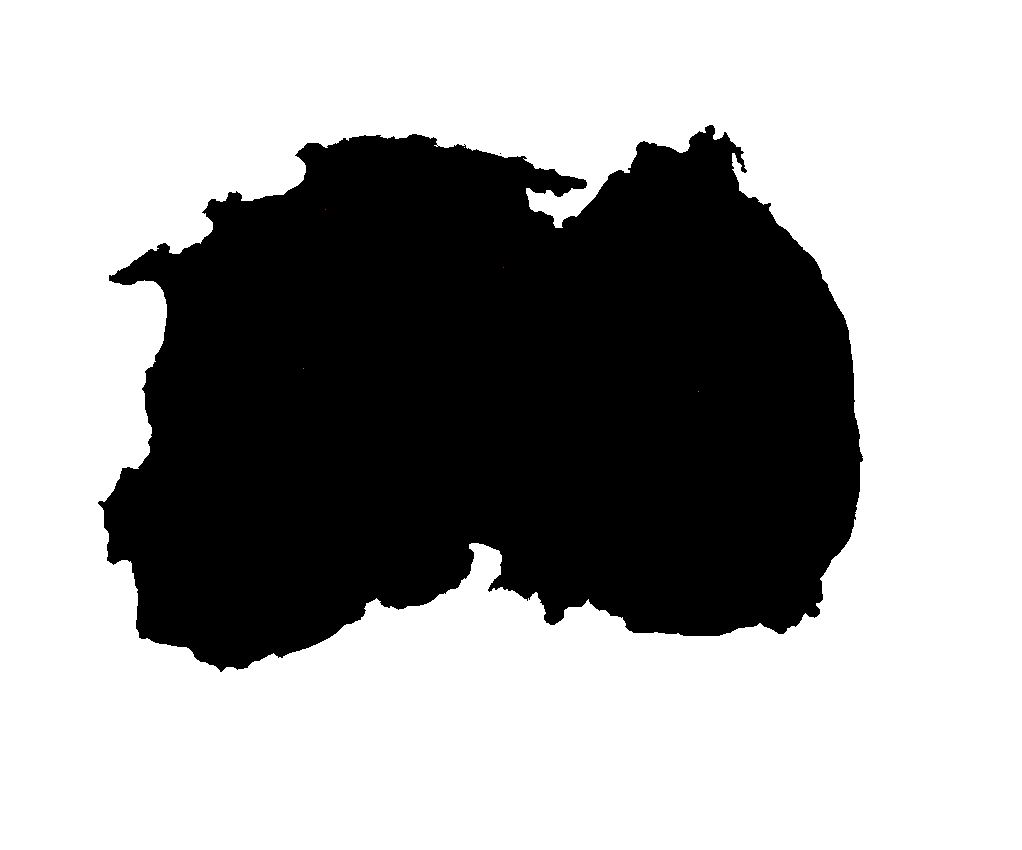

Supplement: Supplementary file 2 [file Datasheet2.zip › figshare/ImageIn/Experiment_103.tif]

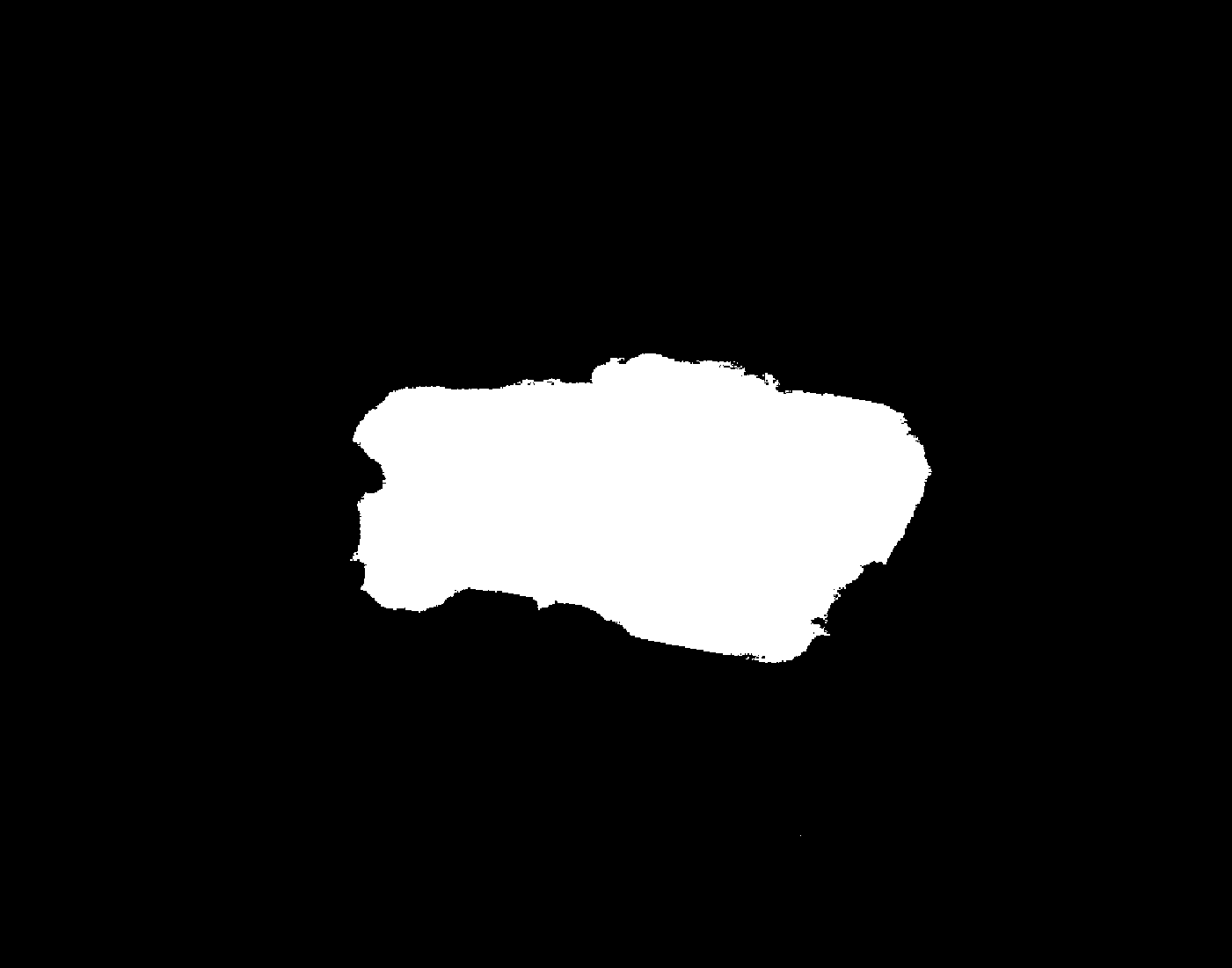

Supplement: Supplementary file 2 [file Datasheet2.zip › figshare/Lesion/Experiment_052.tif]

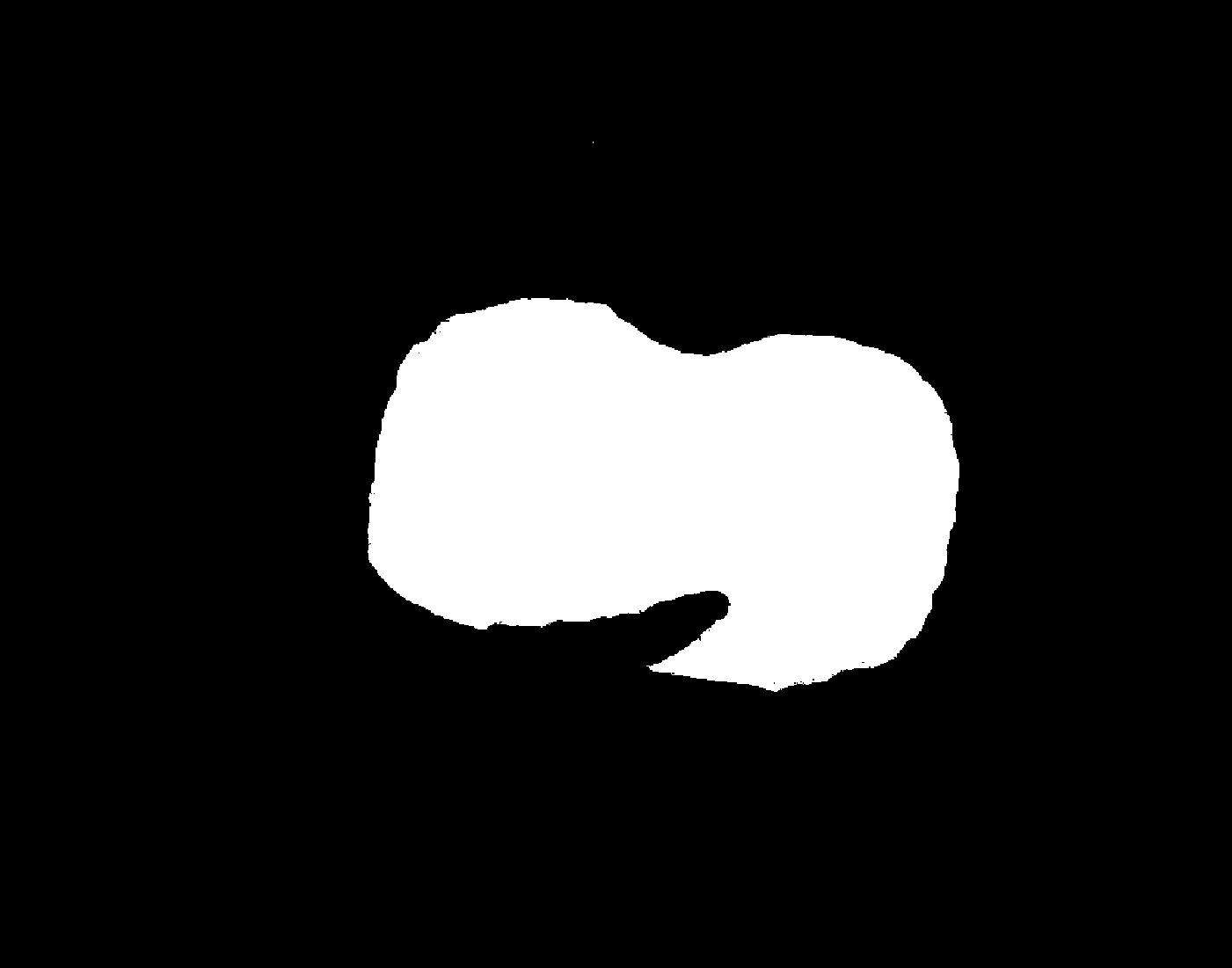

Supplement: Supplementary file 2 [file Datasheet2.zip › figshare/Lesion/Experiment_053.tif]

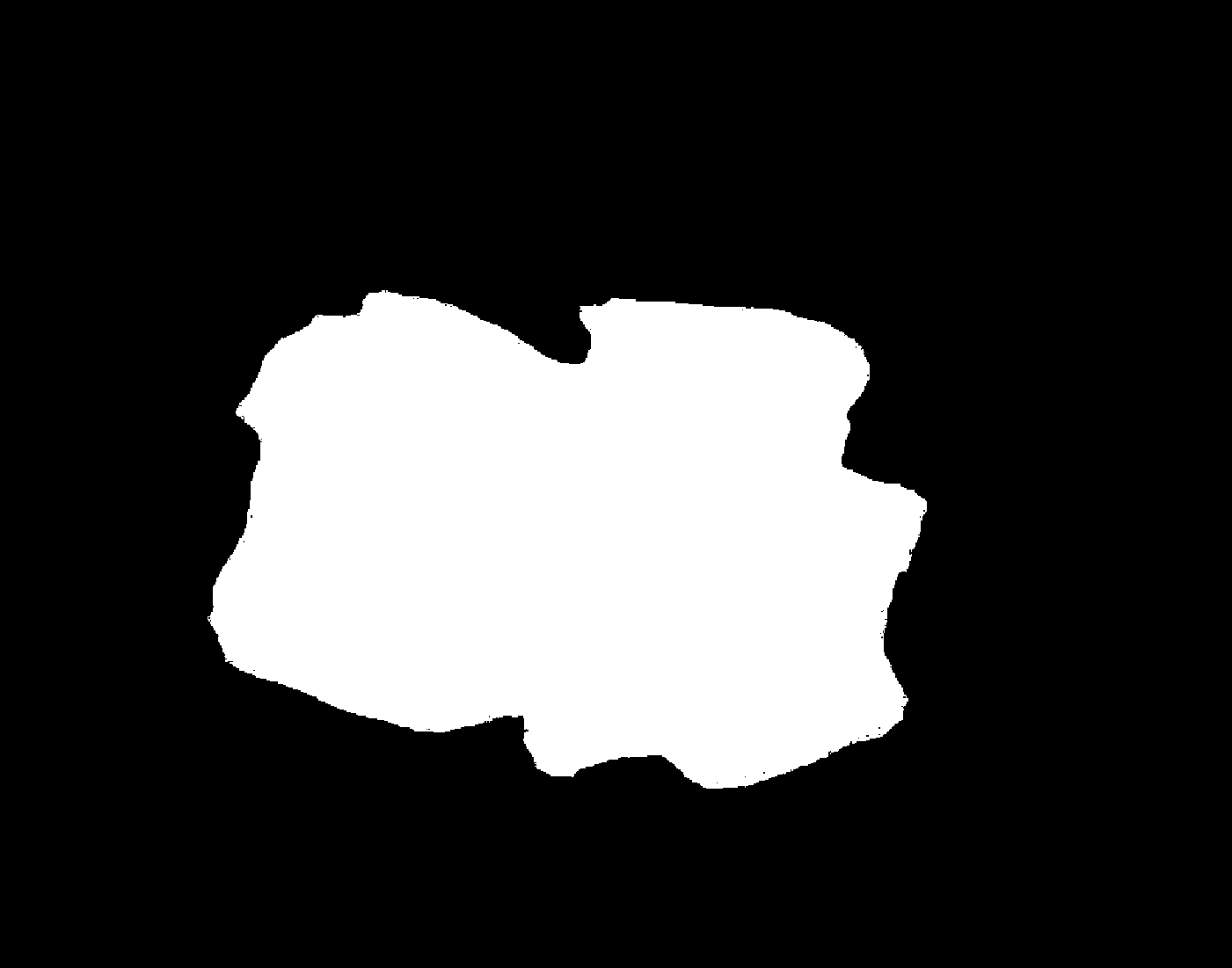

Supplement: Supplementary file 2 [file Datasheet2.zip › figshare/Lesion/Experiment_054.tif]

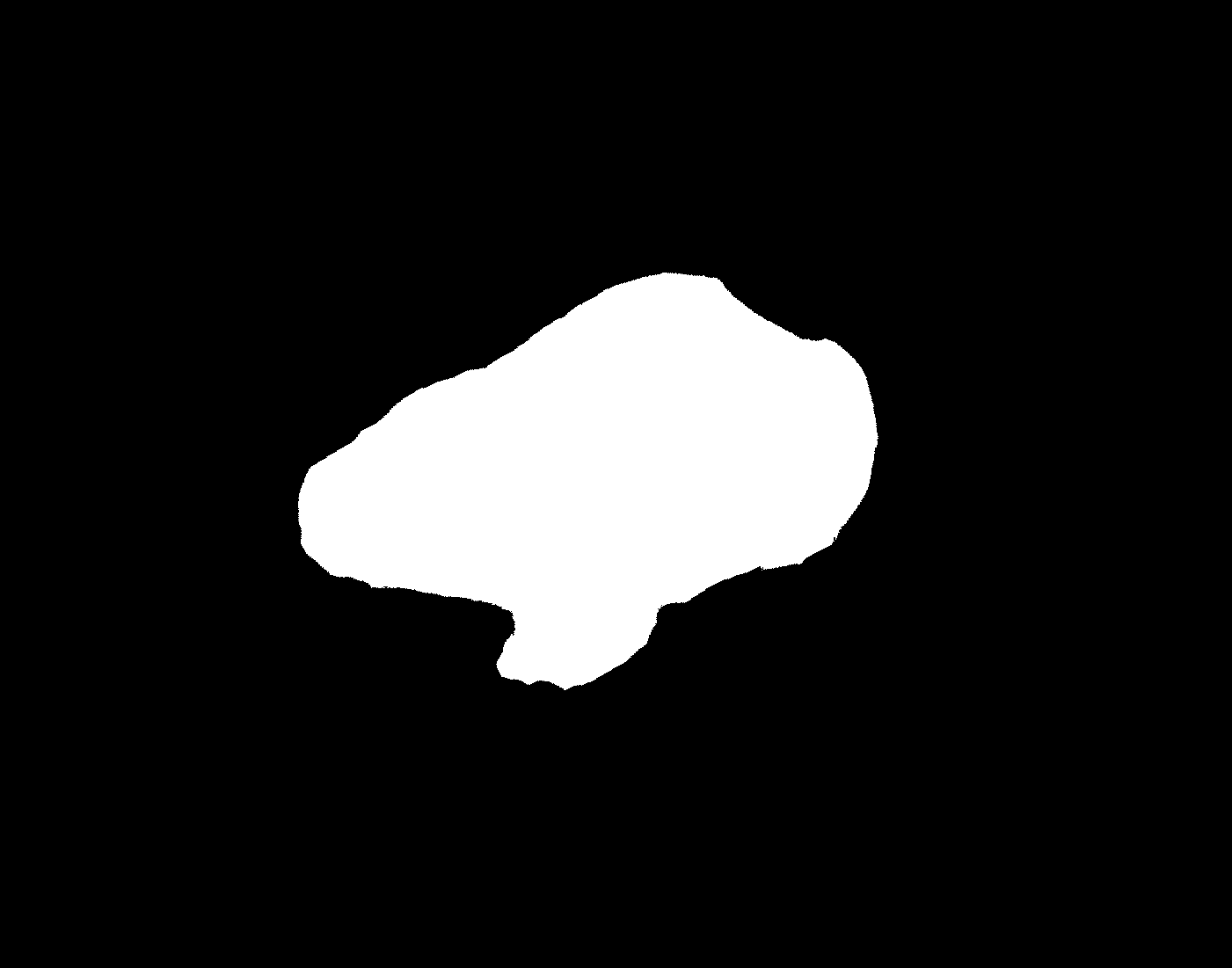

Supplement: Supplementary file 2 [file Datasheet2.zip › figshare/Lesion/Experiment_055.tif]

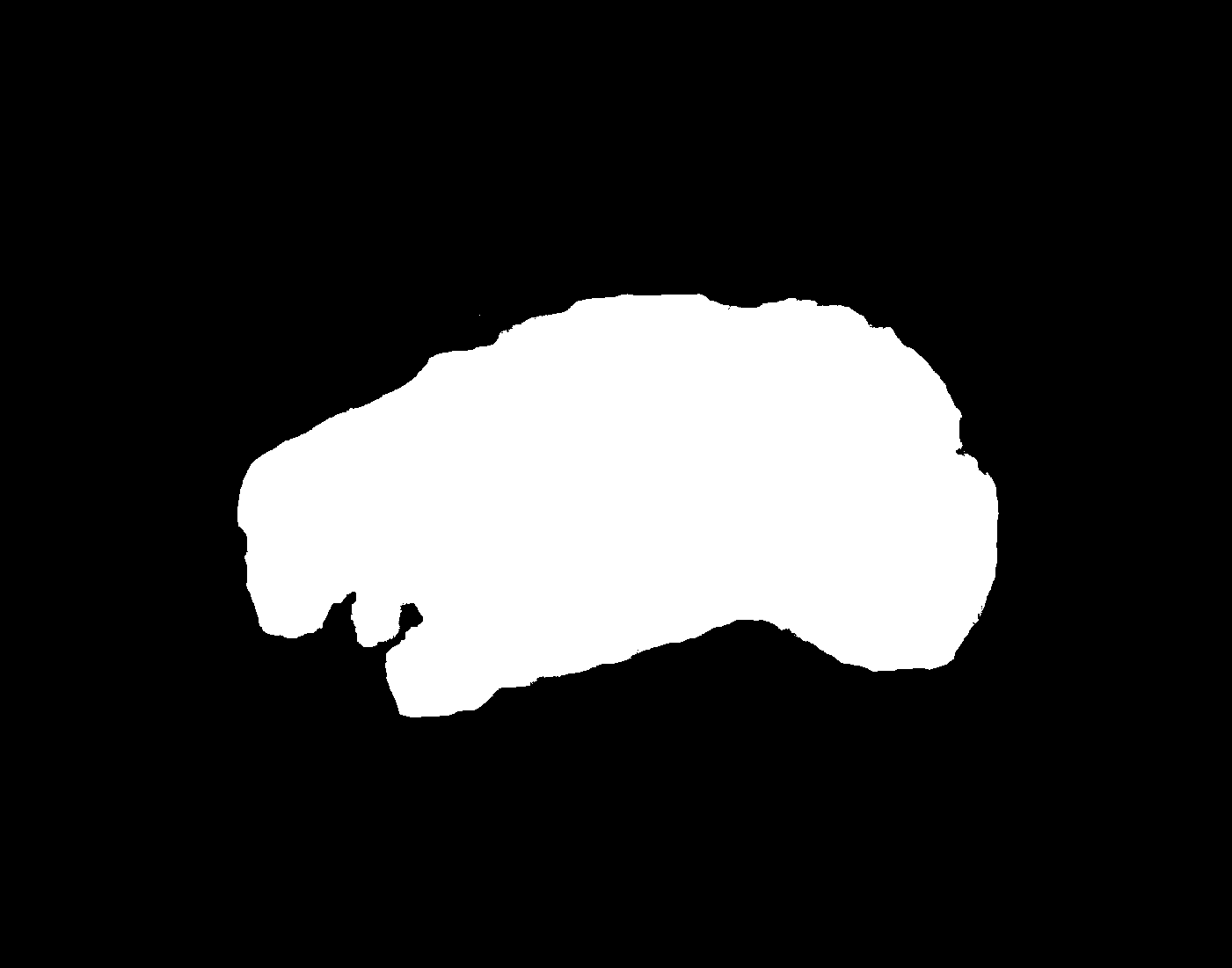

Supplement: Supplementary file 2 [file Datasheet2.zip › figshare/Lesion/Experiment_056.tif]

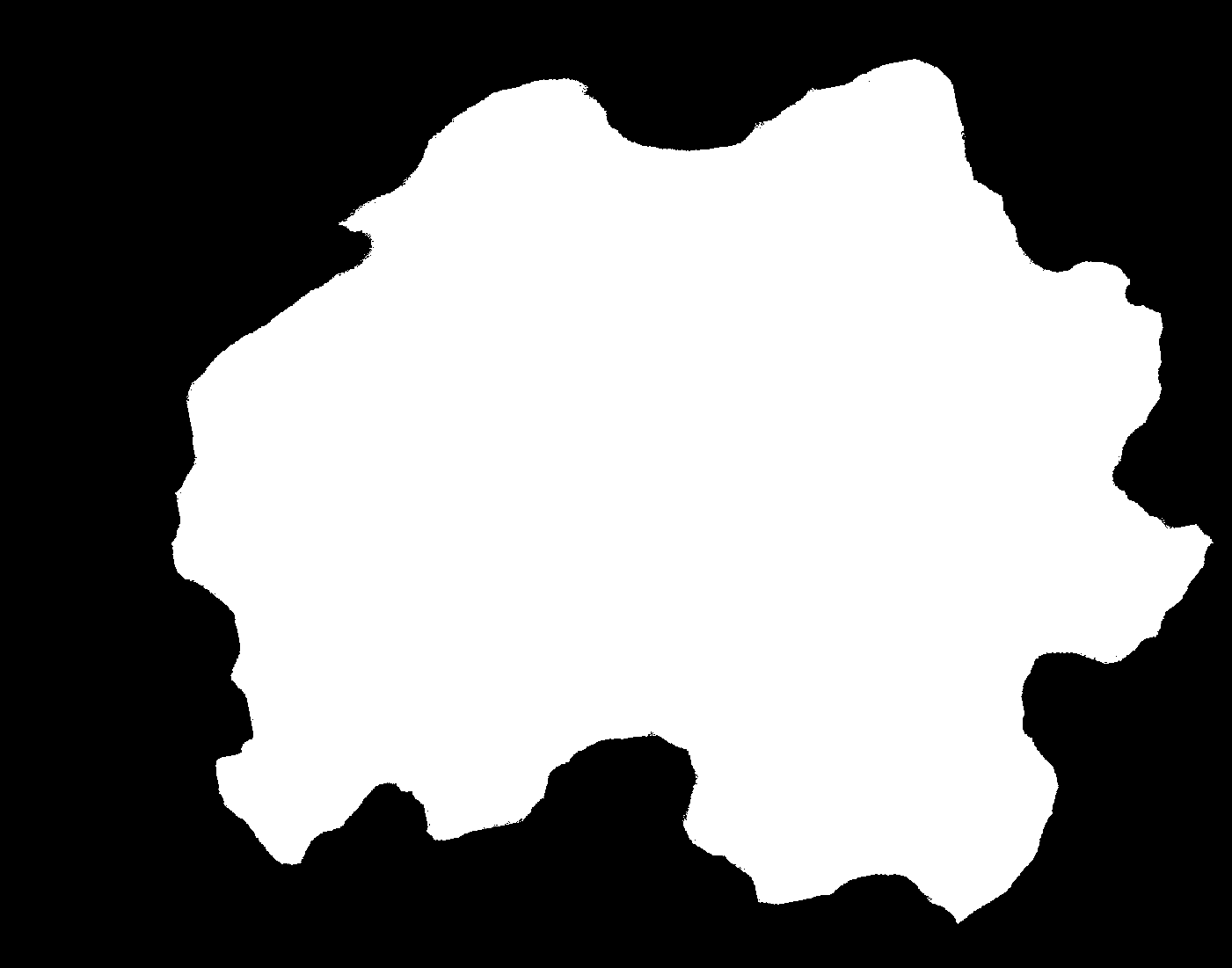

Supplement: Supplementary file 2 [file Datasheet2.zip › figshare/Lesion/Experiment_057.tif]

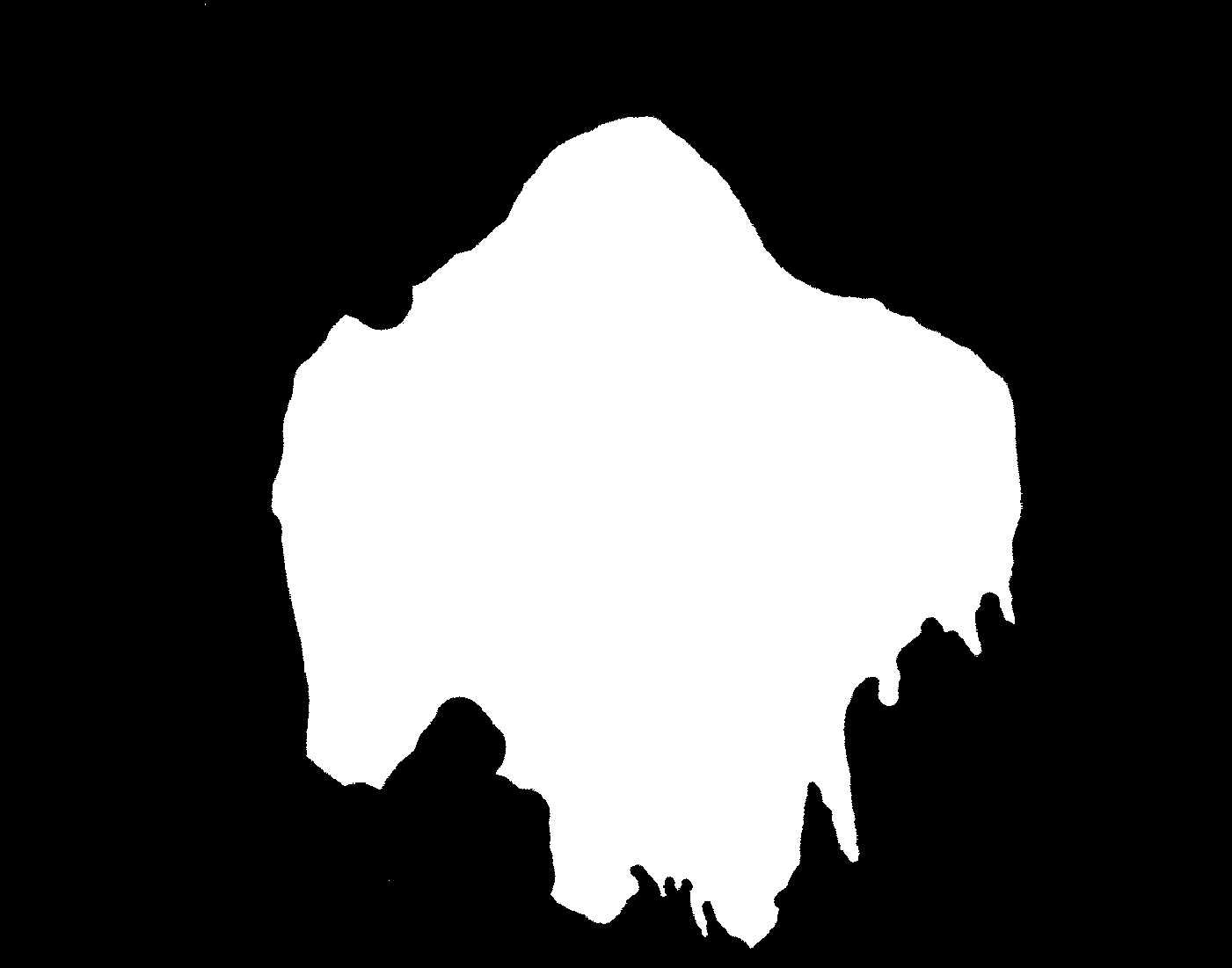

Supplement: Supplementary file 2 [file Datasheet2.zip › figshare/Lesion/Experiment_058.tif]

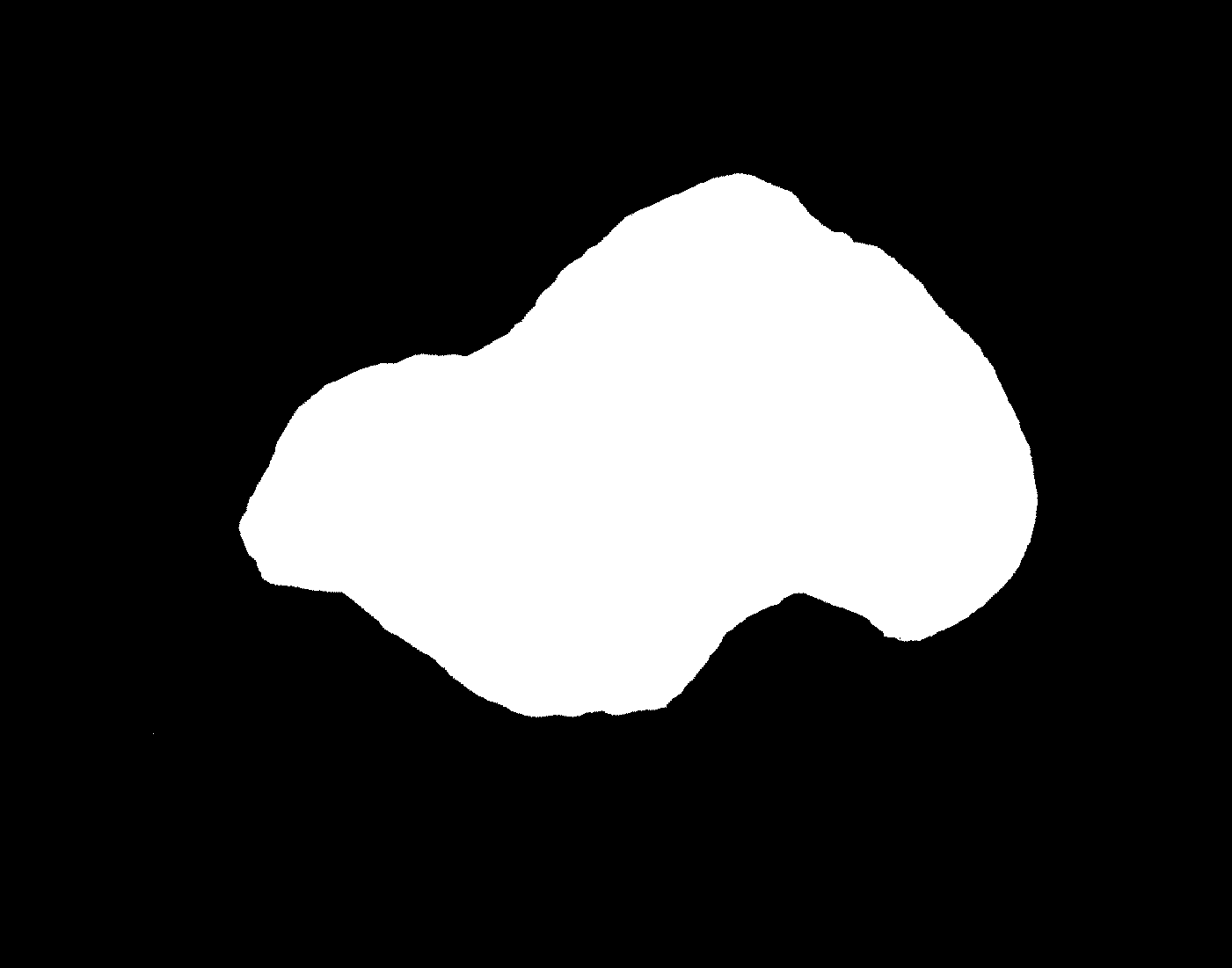

Supplement: Supplementary file 2 [file Datasheet2.zip › figshare/Lesion/Experiment_059.tif]

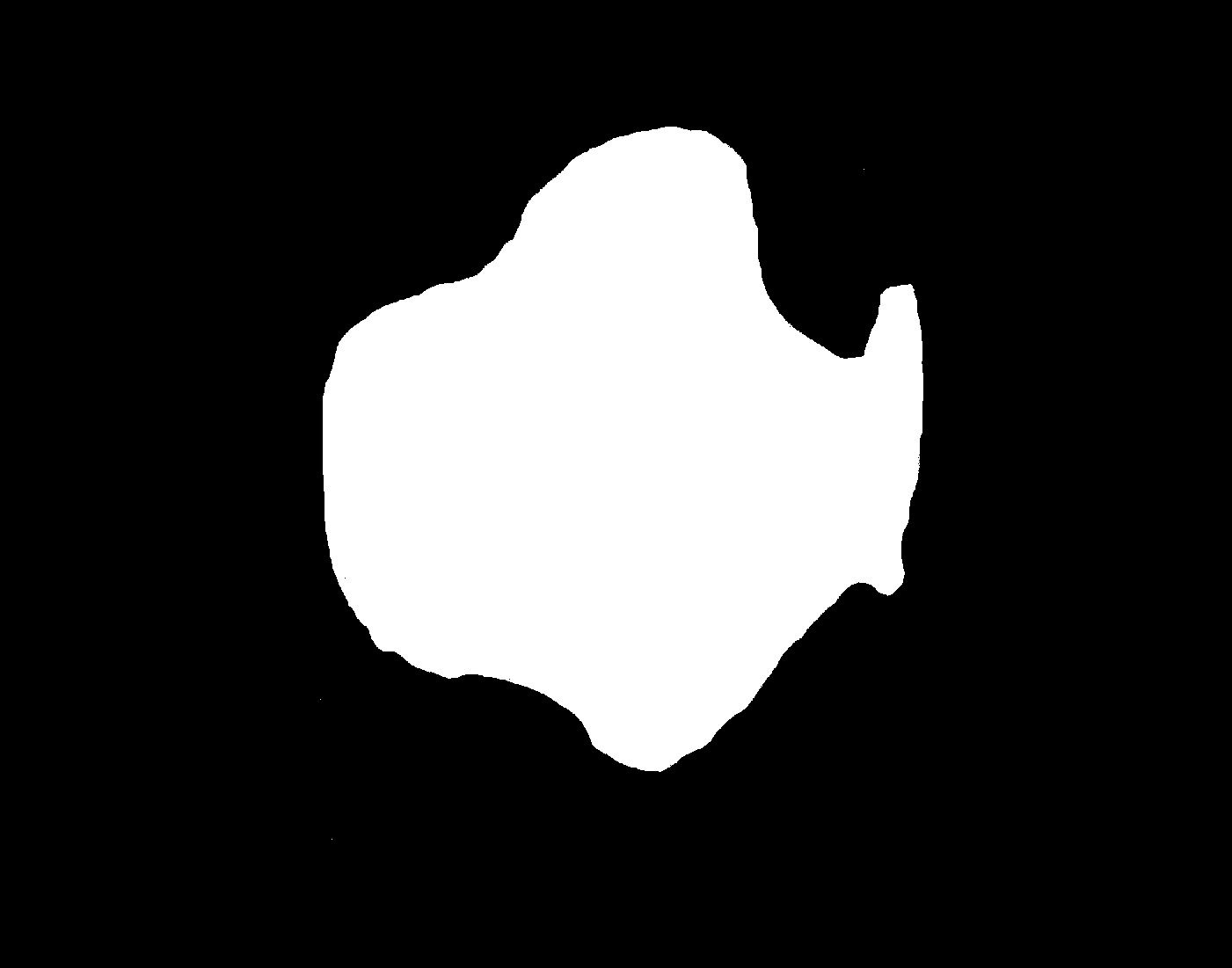

Supplement: Supplementary file 2 [file Datasheet2.zip › figshare/Lesion/Experiment_060.tif]

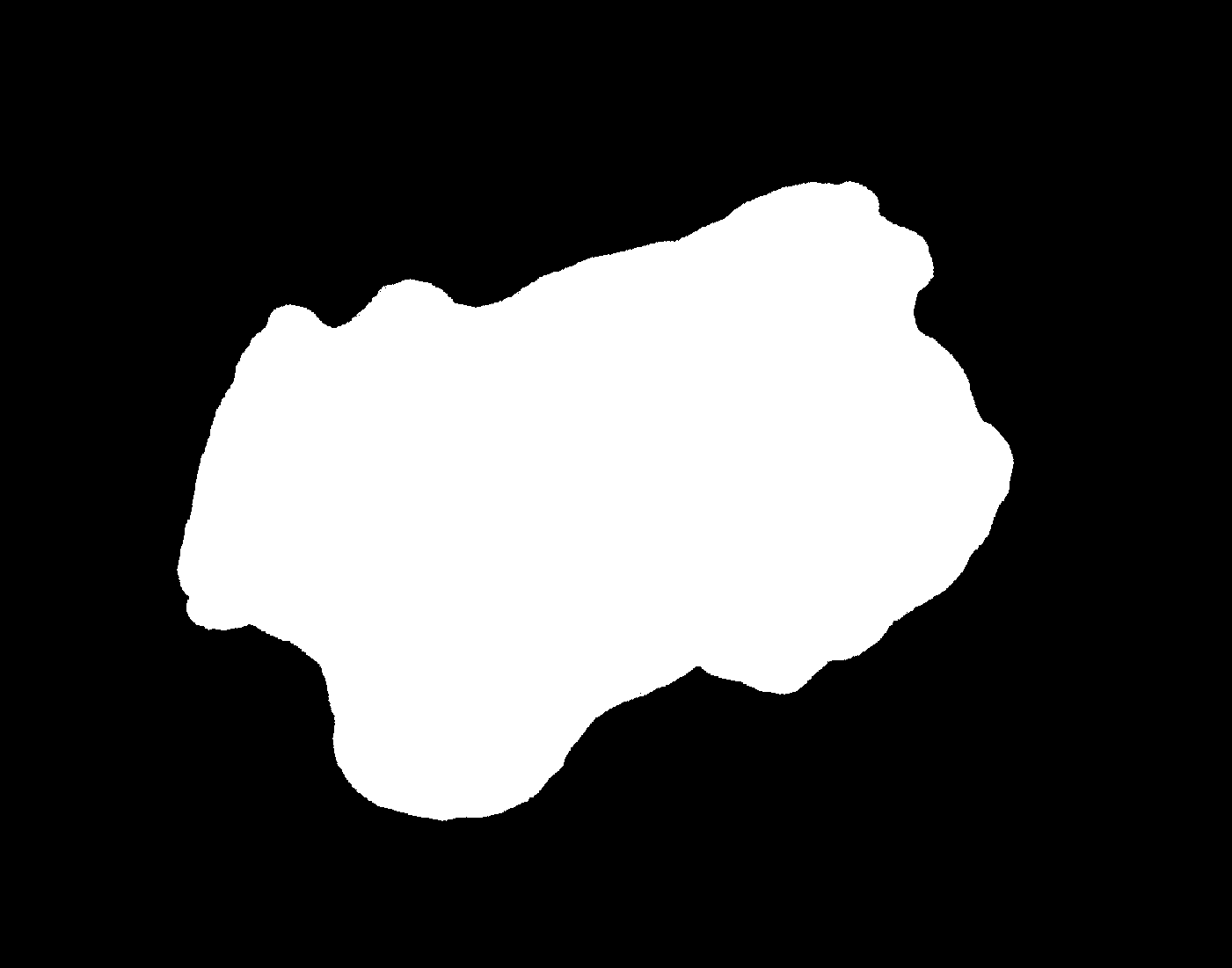

Supplement: Supplementary file 2 [file Datasheet2.zip › figshare/Lesion/Experiment_061.tif]

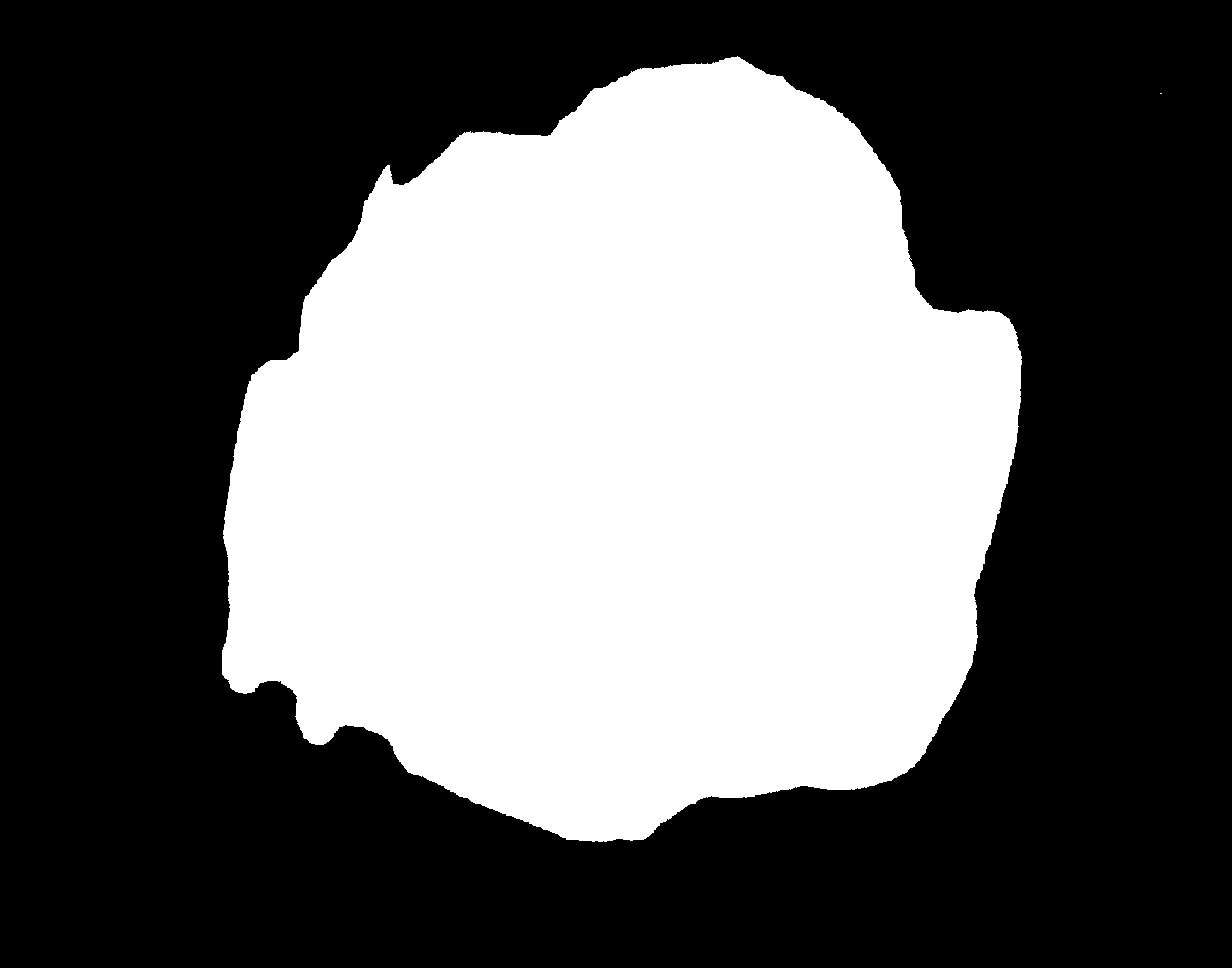

Supplement: Supplementary file 2 [file Datasheet2.zip › figshare/Lesion/Experiment_062.tif]

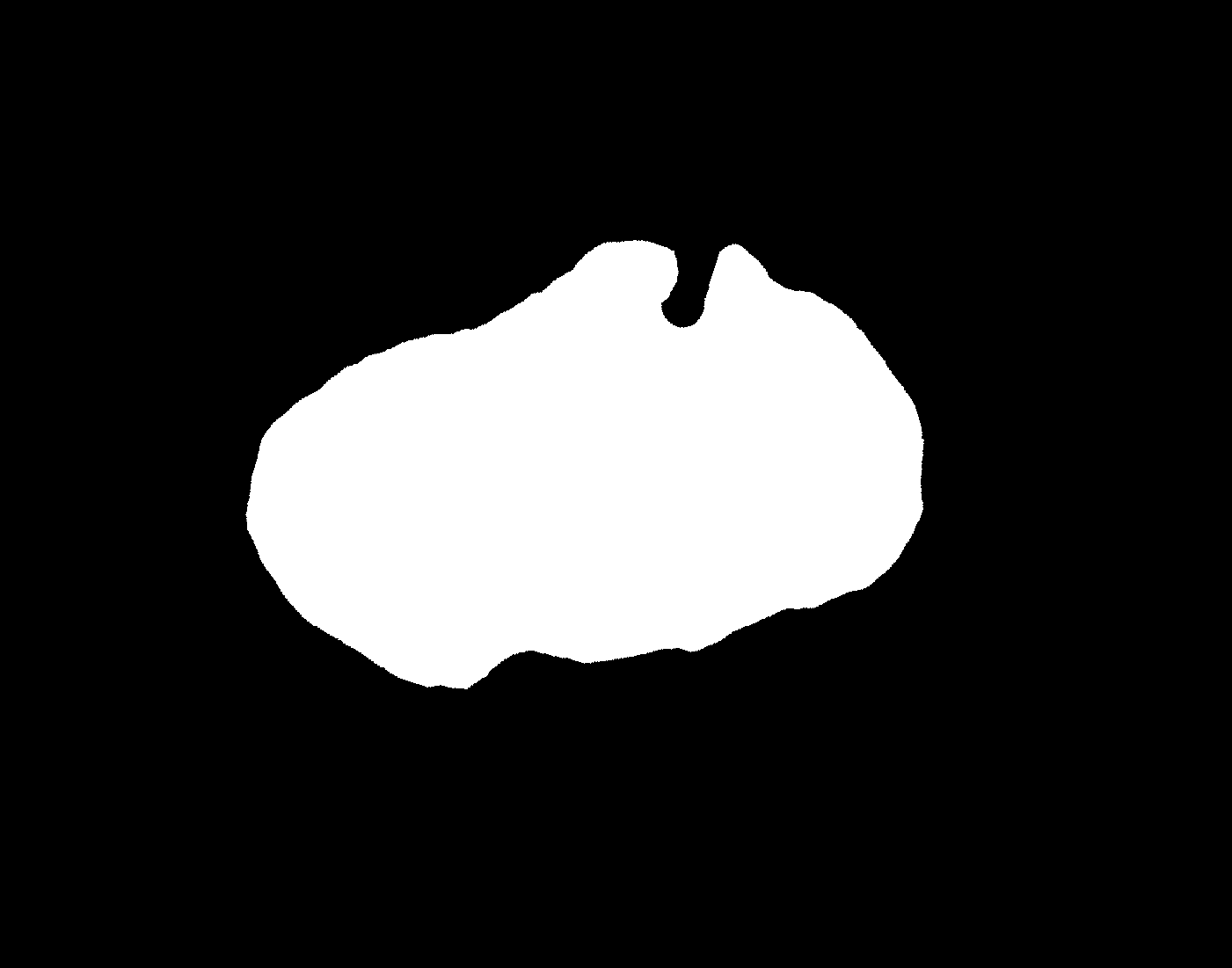

Supplement: Supplementary file 2 [file Datasheet2.zip › figshare/Lesion/Experiment_063.tif]

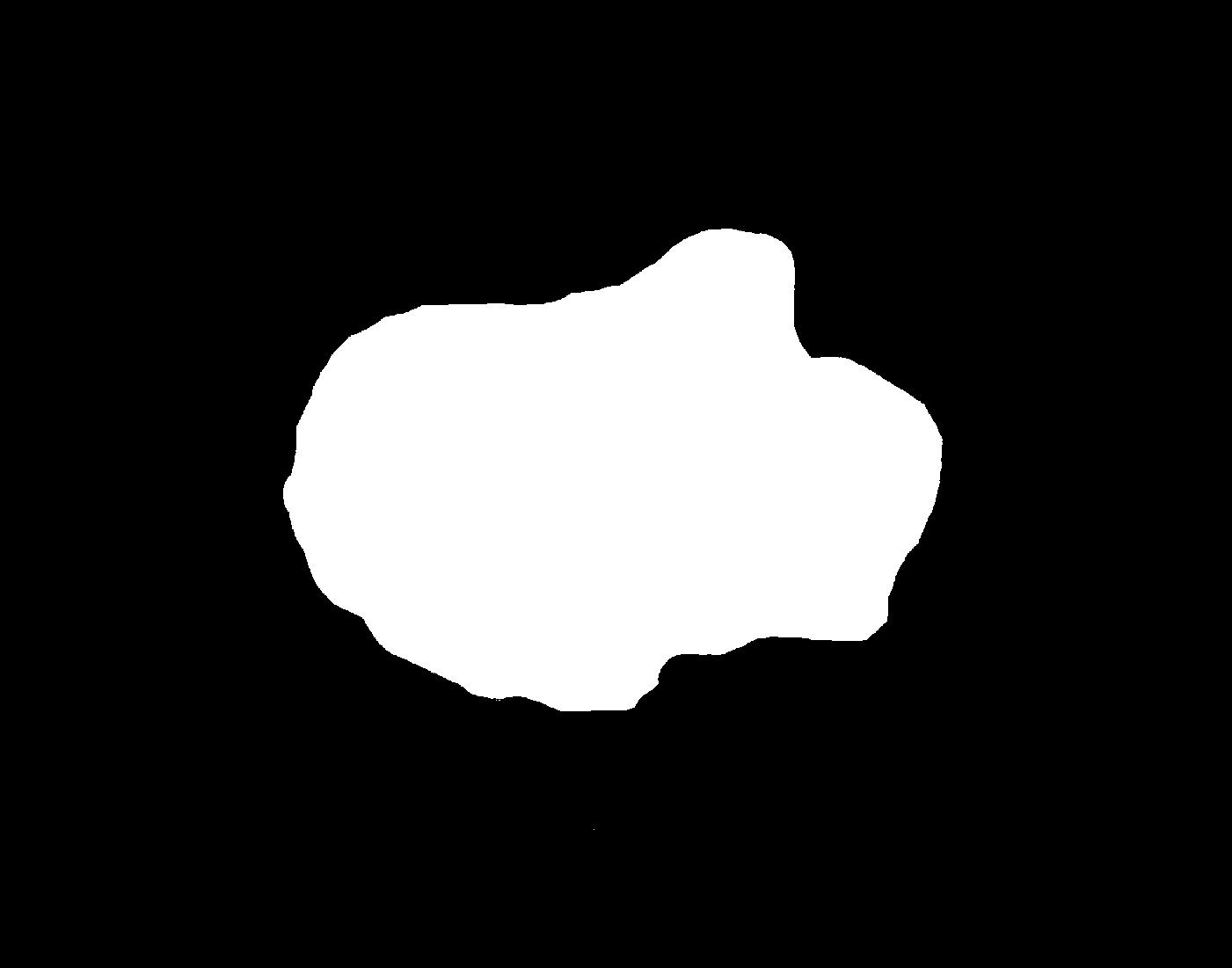

Supplement: Supplementary file 2 [file Datasheet2.zip › figshare/Lesion/Experiment_064.tif]

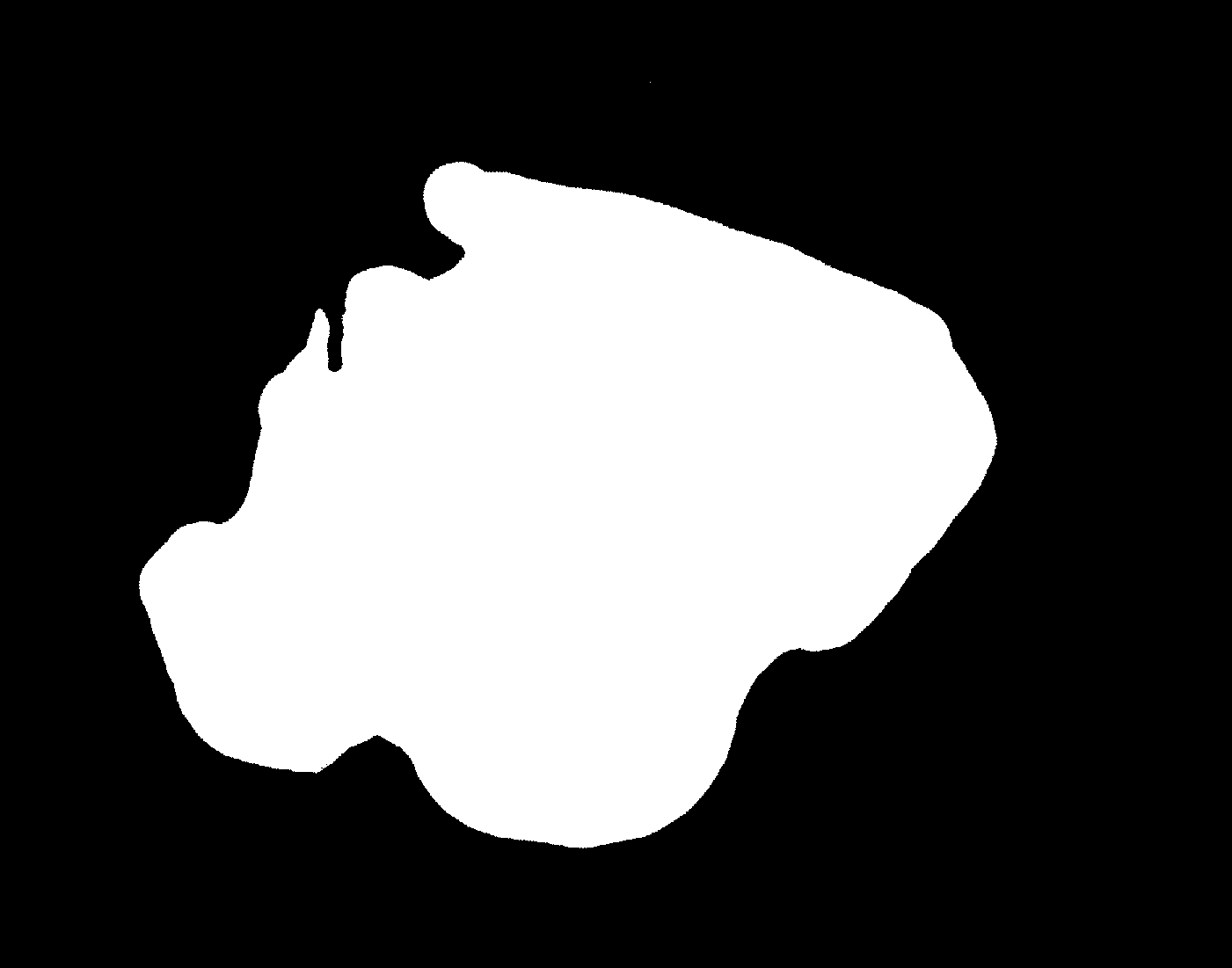

Supplement: Supplementary file 2 [file Datasheet2.zip › figshare/Lesion/Experiment_065.tif]

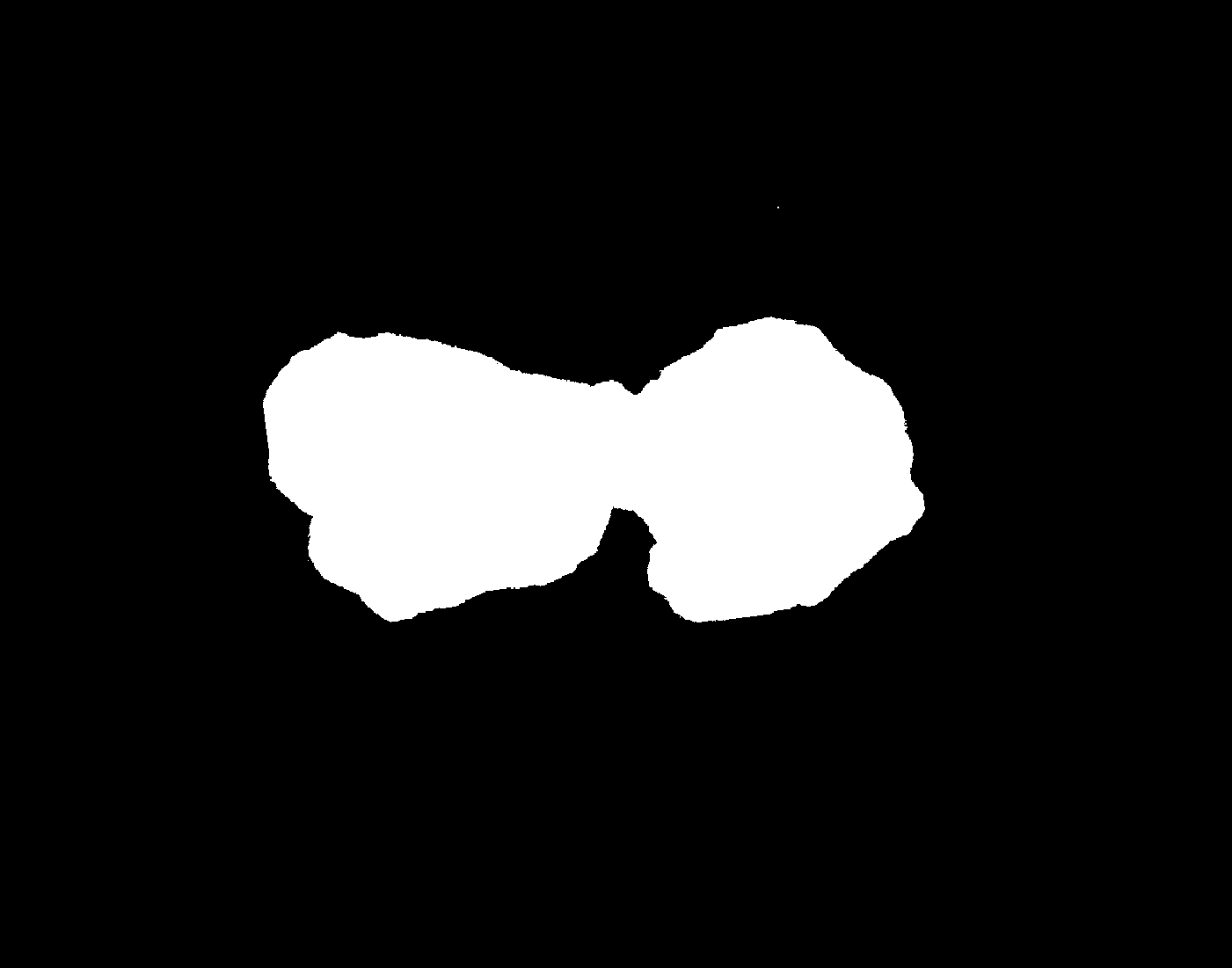

Supplement: Supplementary file 2 [file Datasheet2.zip › figshare/Lesion/Experiment_066.tif]

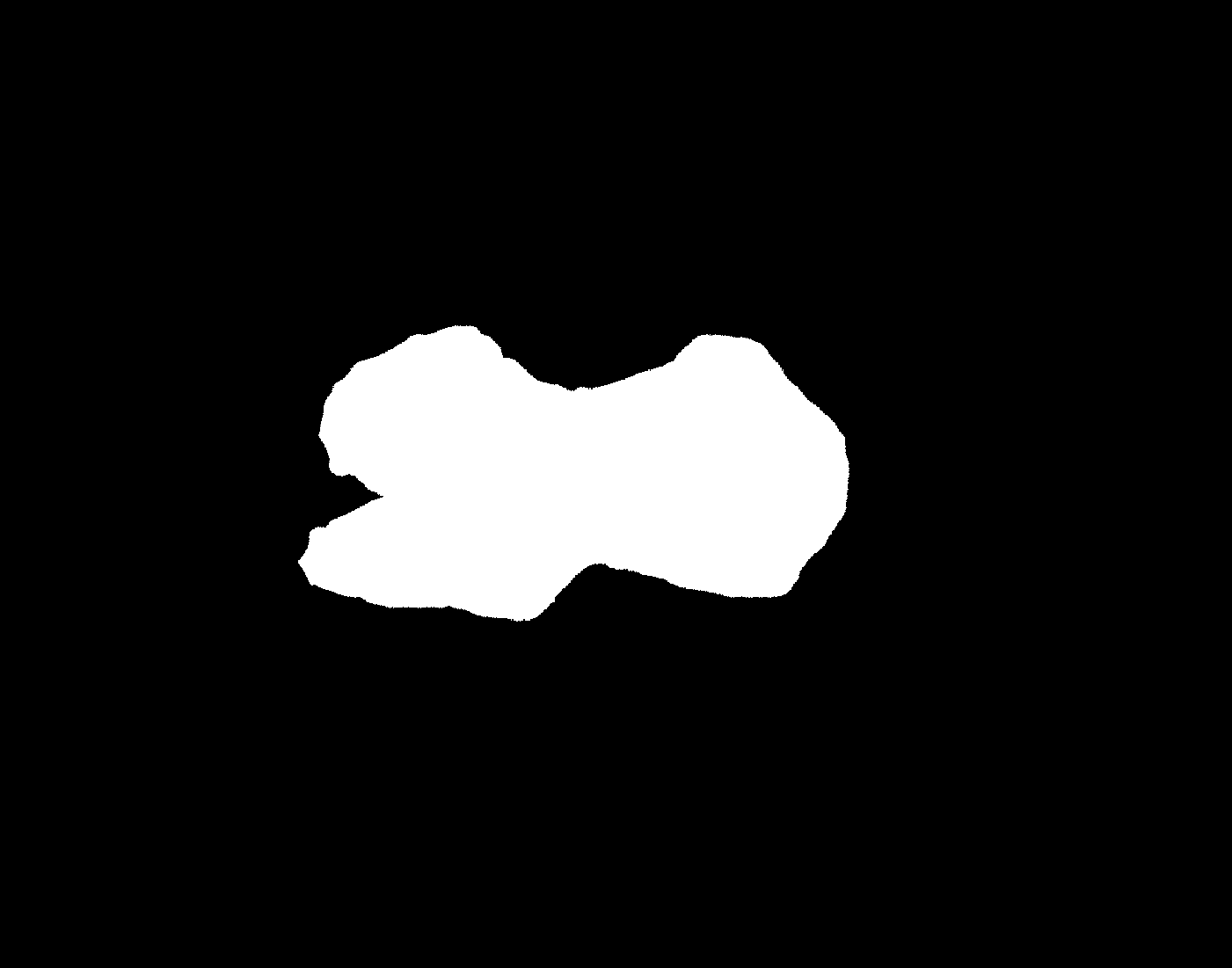

Supplement: Supplementary file 2 [file Datasheet2.zip › figshare/Lesion/Experiment_067.tif]

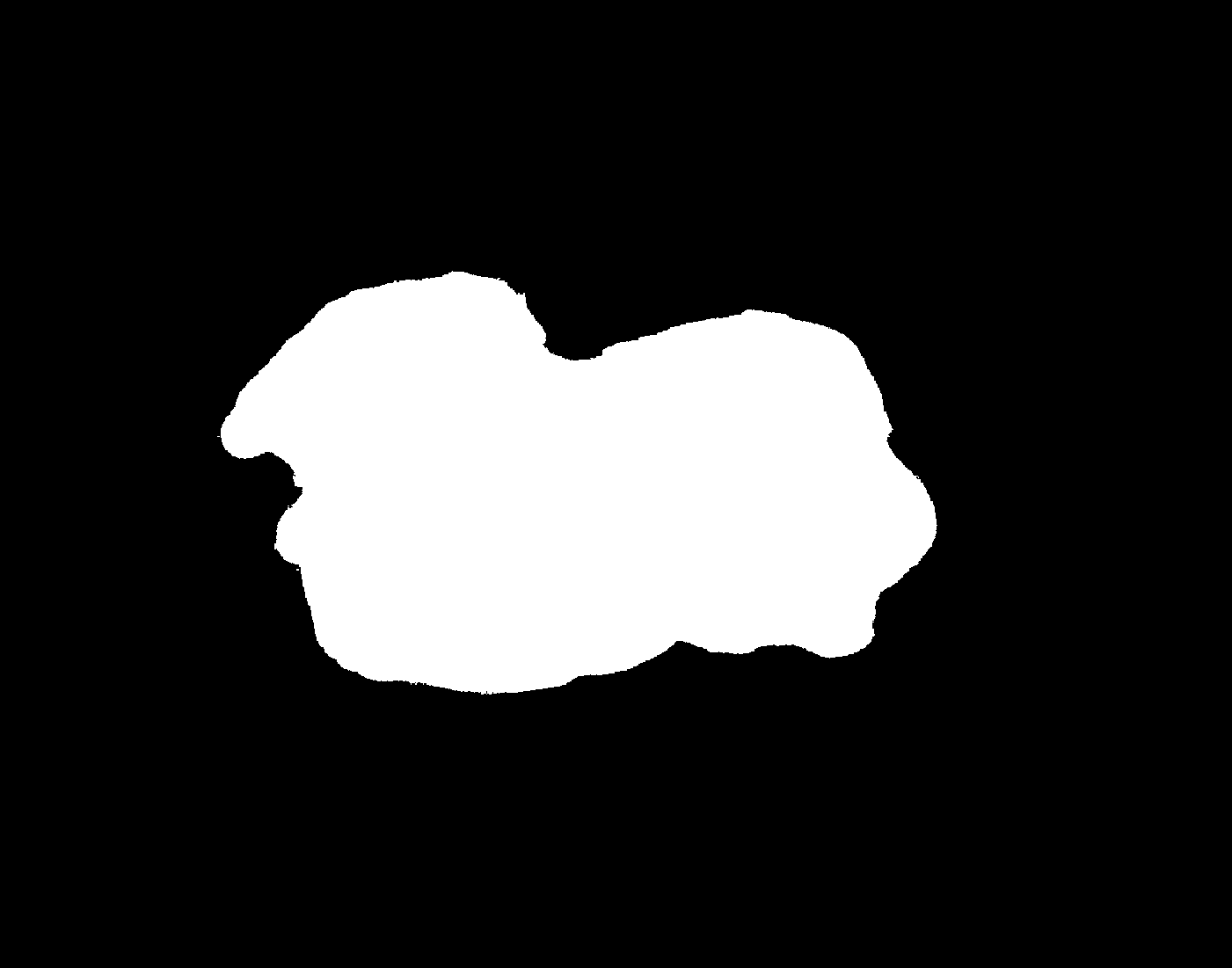

Supplement: Supplementary file 2 [file Datasheet2.zip › figshare/Lesion/Experiment_068.tif]

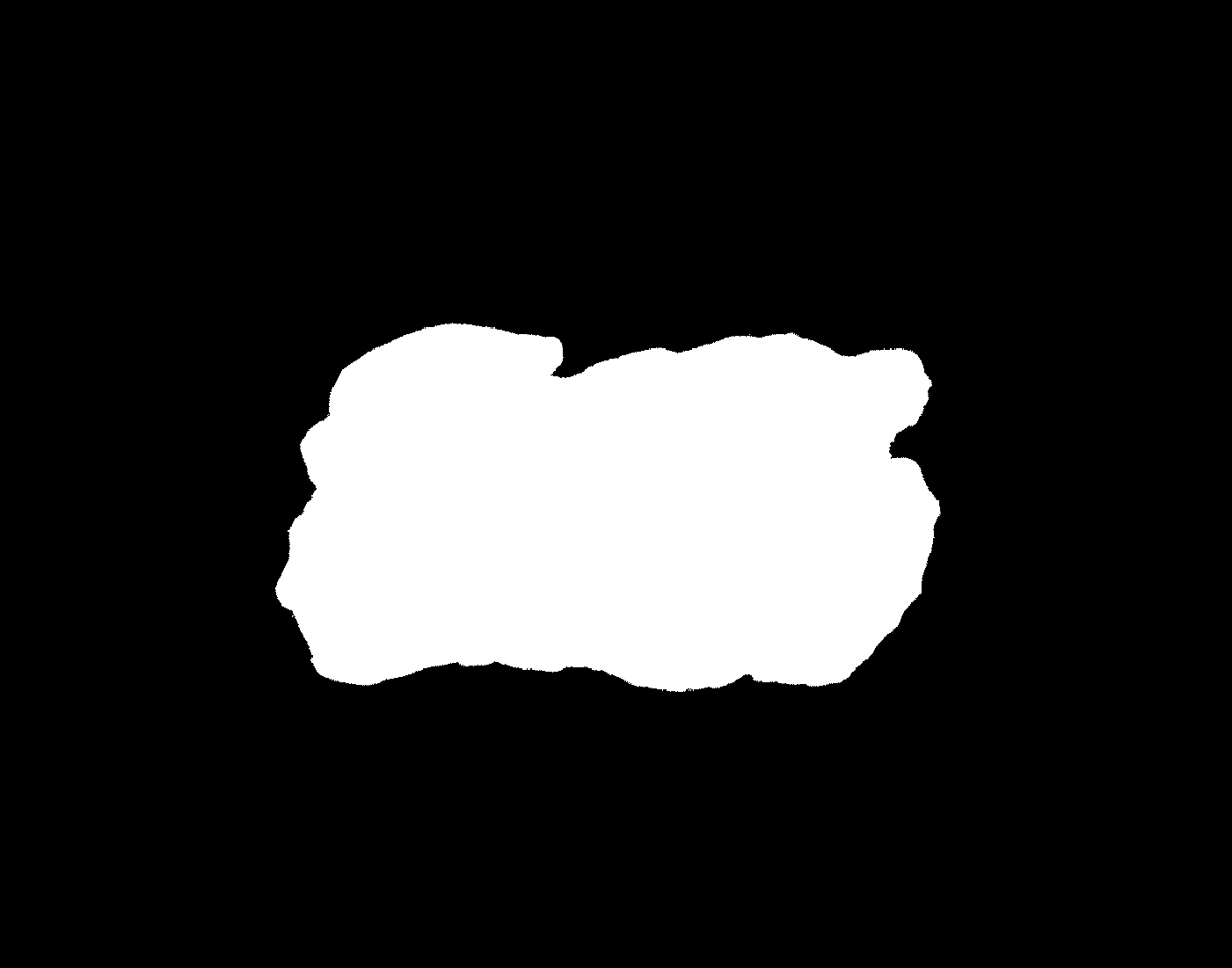

Supplement: Supplementary file 2 [file Datasheet2.zip › figshare/Lesion/Experiment_069.tif]

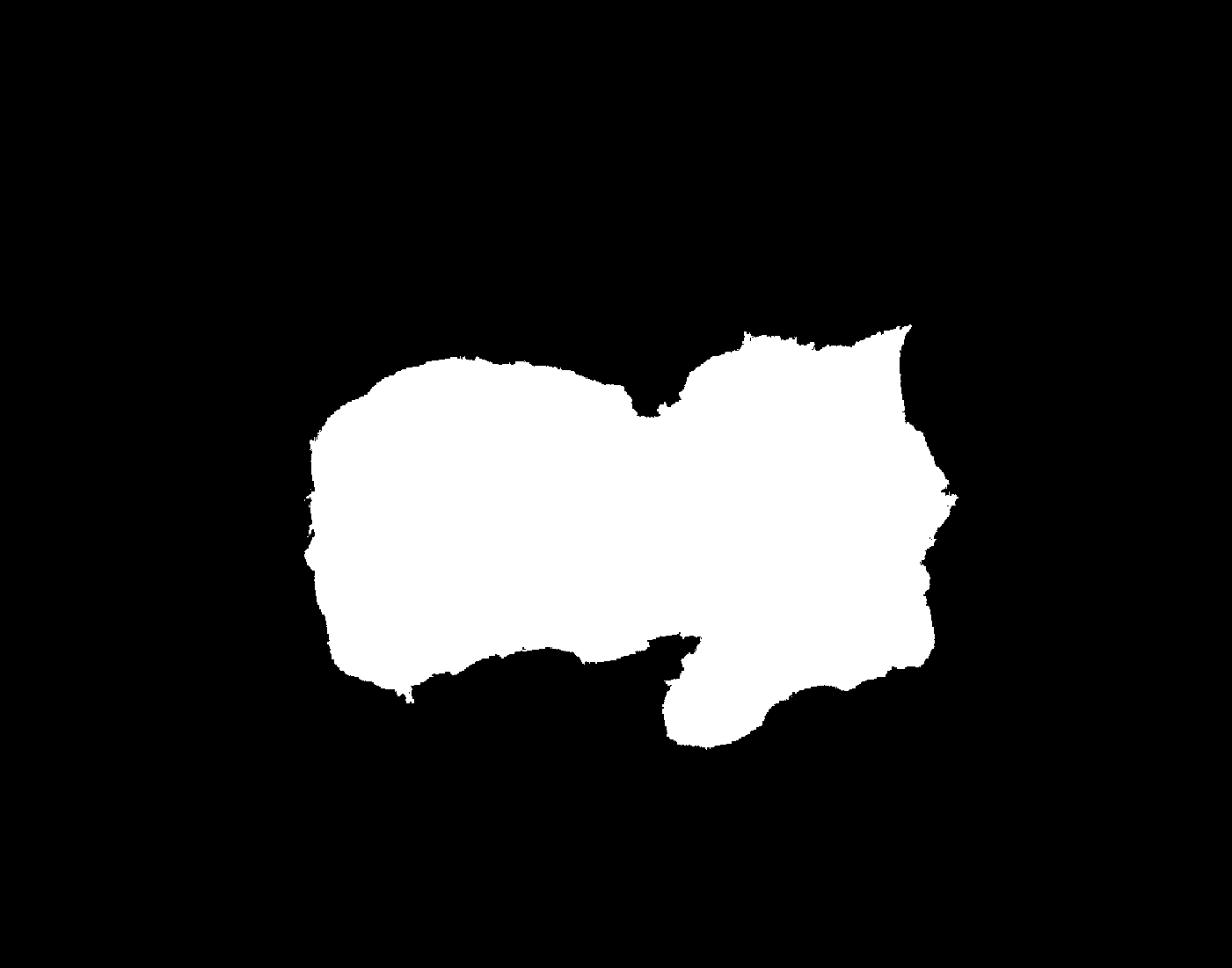

Supplement: Supplementary file 2 [file Datasheet2.zip › figshare/Lesion/Experiment_070.tif]

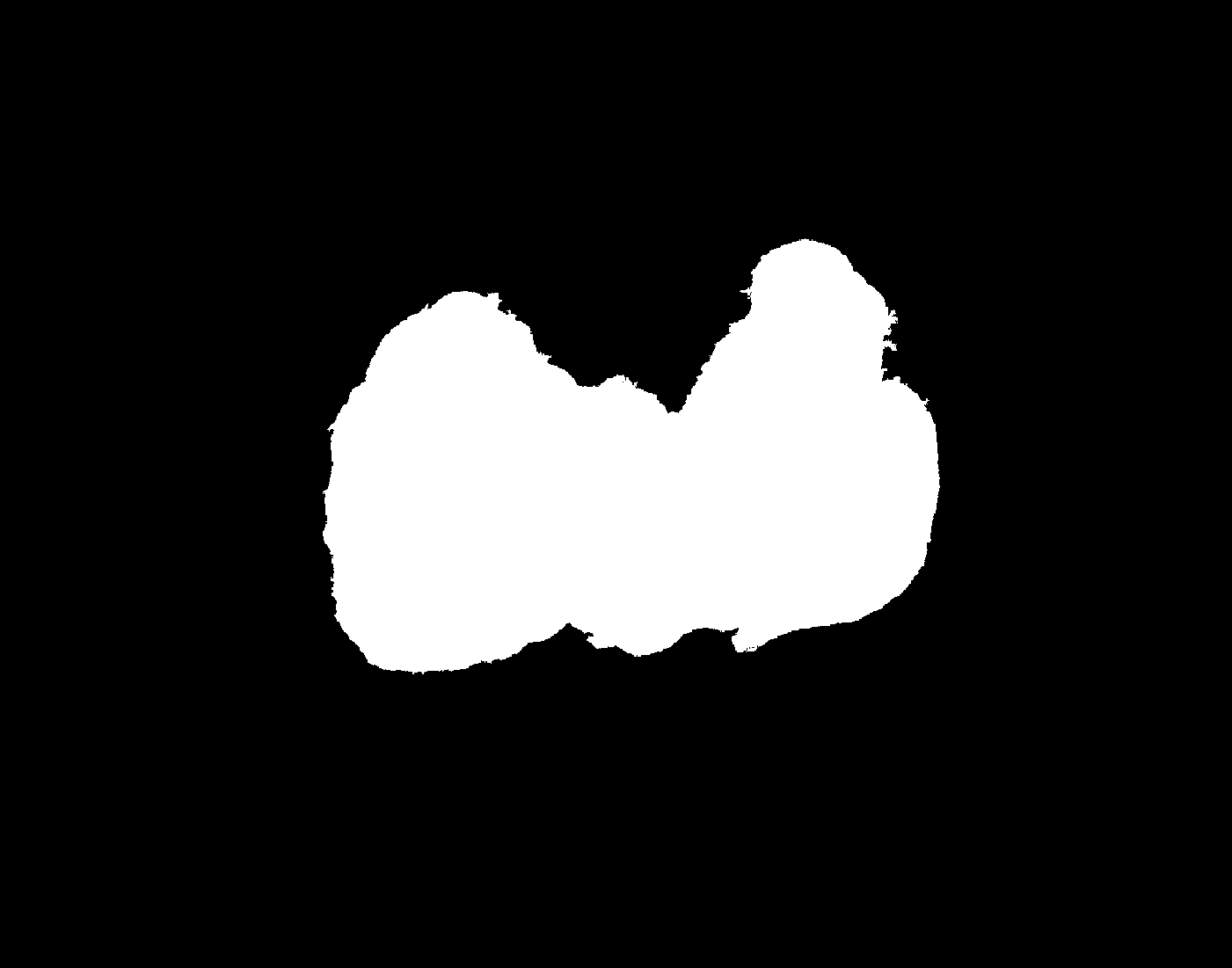

Supplement: Supplementary file 2 [file Datasheet2.zip › figshare/Lesion/Experiment_071.tif]

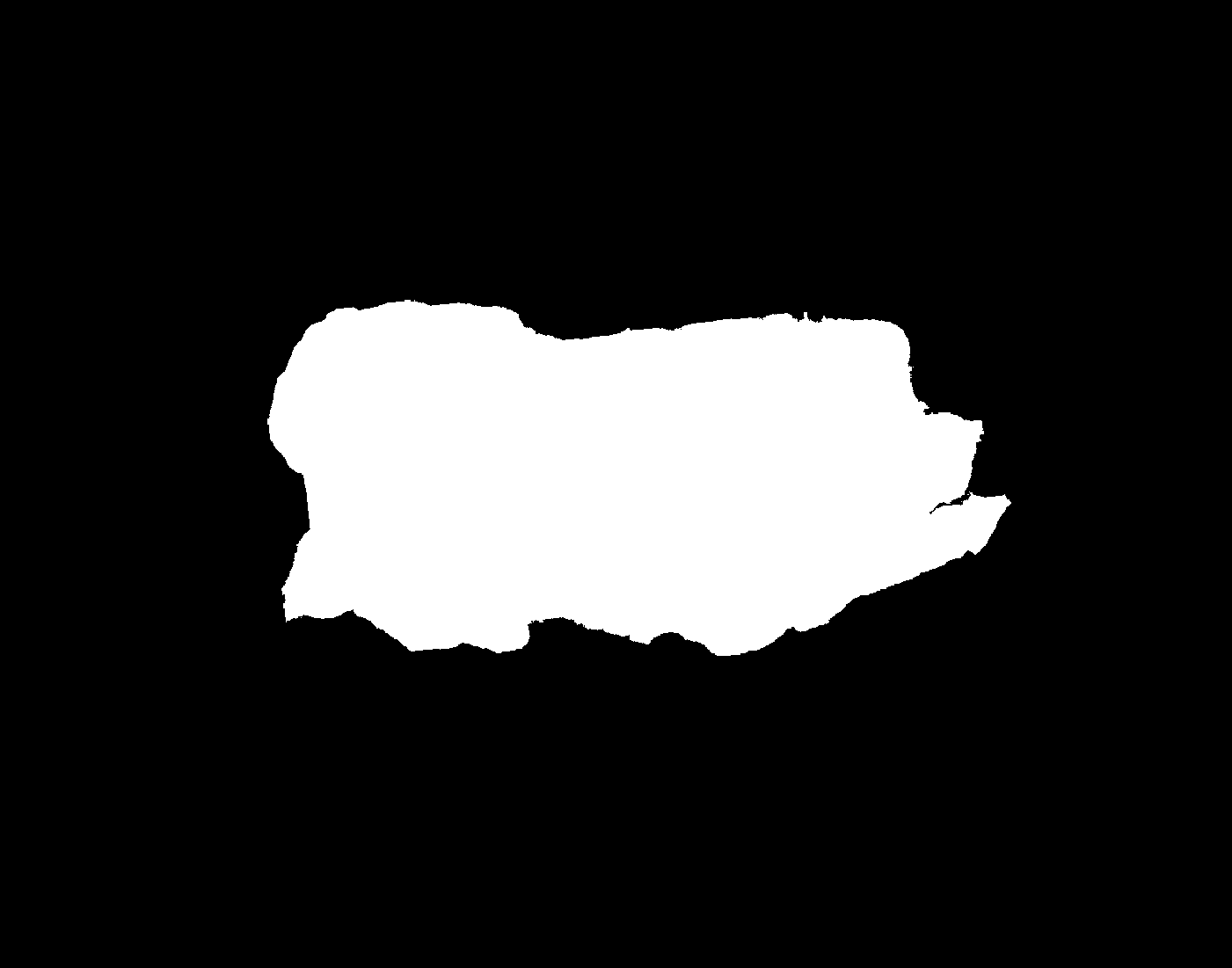

Supplement: Supplementary file 2 [file Datasheet2.zip › figshare/Lesion/Experiment_072.tif]

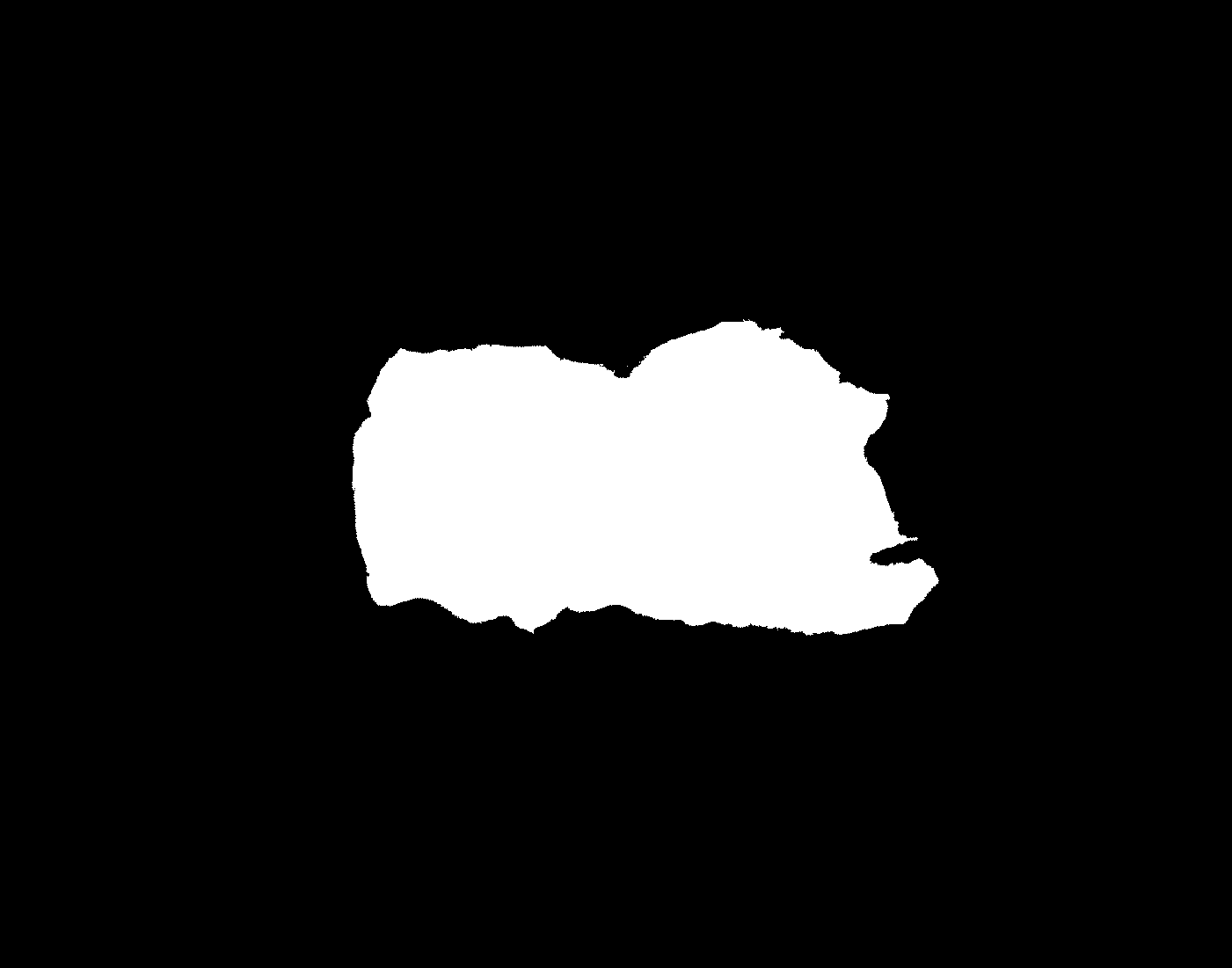

Supplement: Supplementary file 2 [file Datasheet2.zip › figshare/Lesion/Experiment_073.tif]

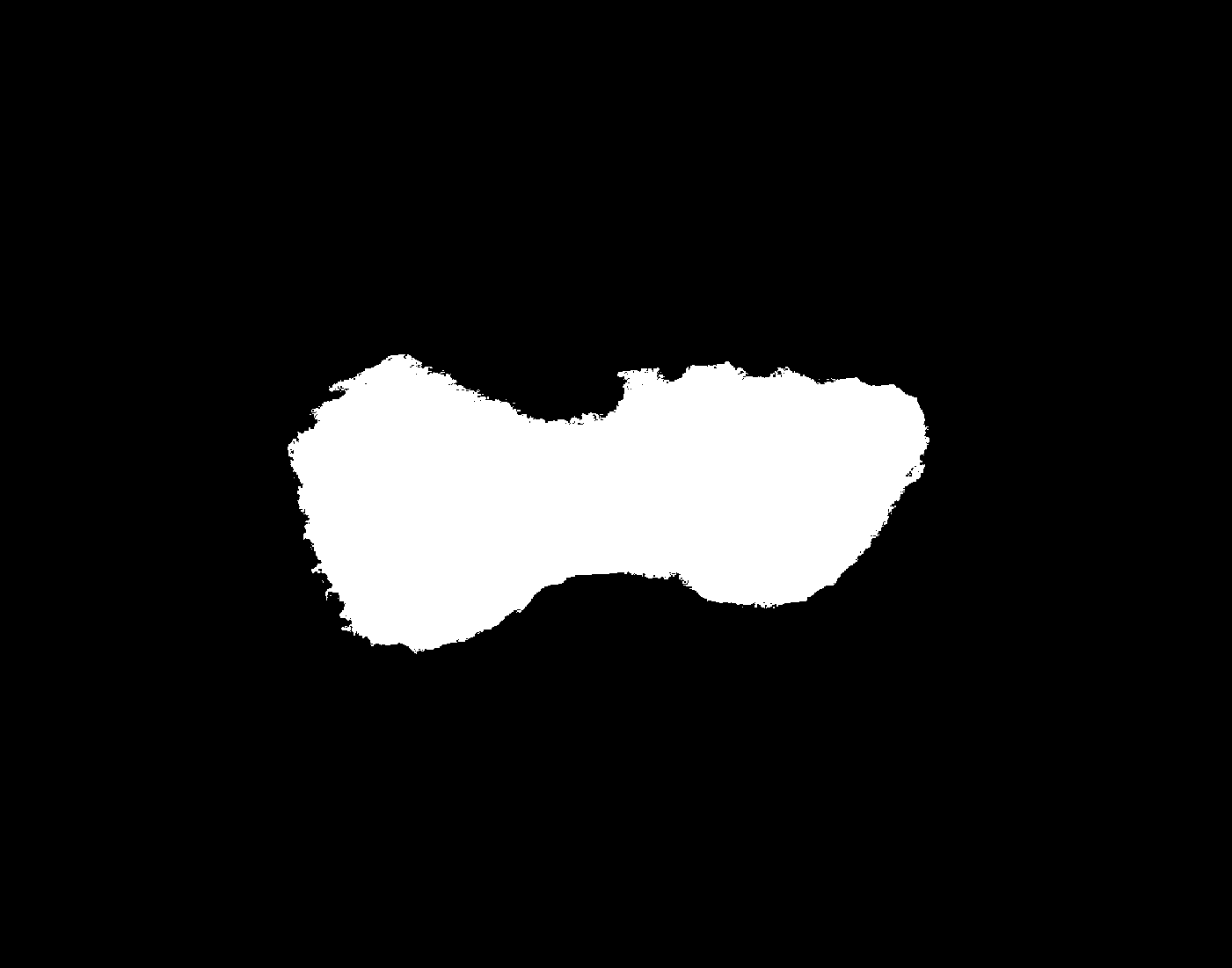

Supplement: Supplementary file 2 [file Datasheet2.zip › figshare/Lesion/Experiment_074.tif]

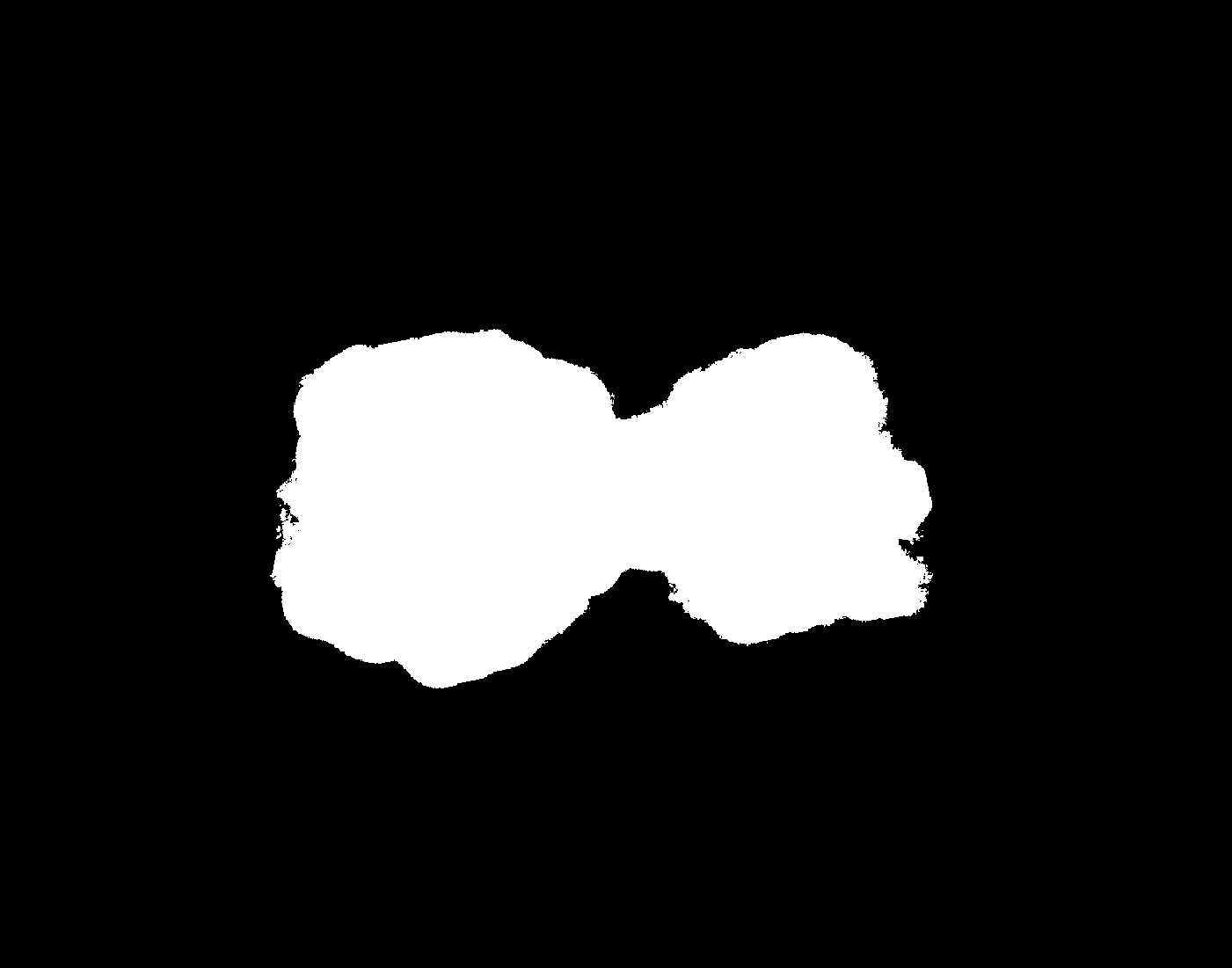

Supplement: Supplementary file 2 [file Datasheet2.zip › figshare/Lesion/Experiment_075.tif]

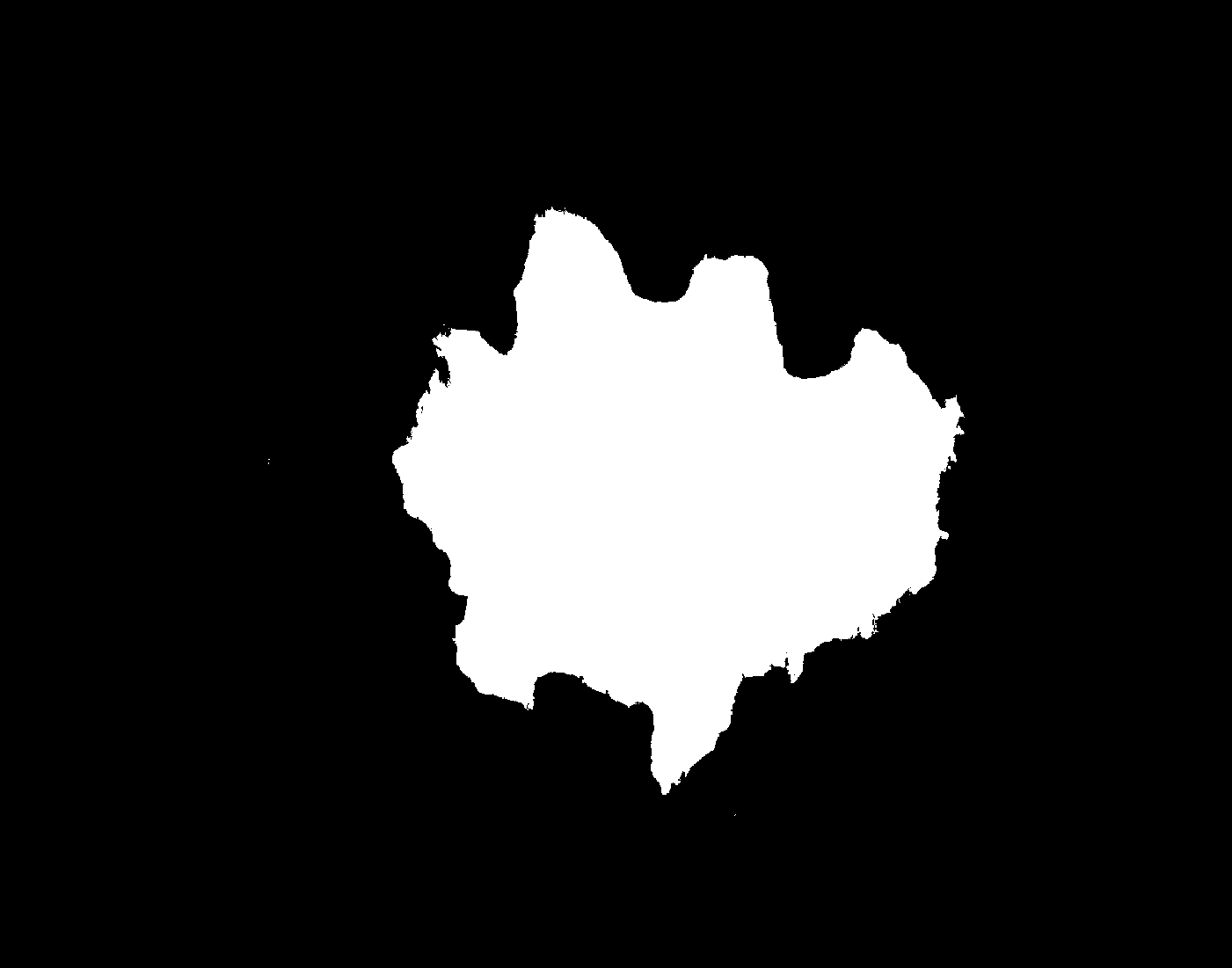

Supplement: Supplementary file 2 [file Datasheet2.zip › figshare/Lesion/Experiment_076.tif]

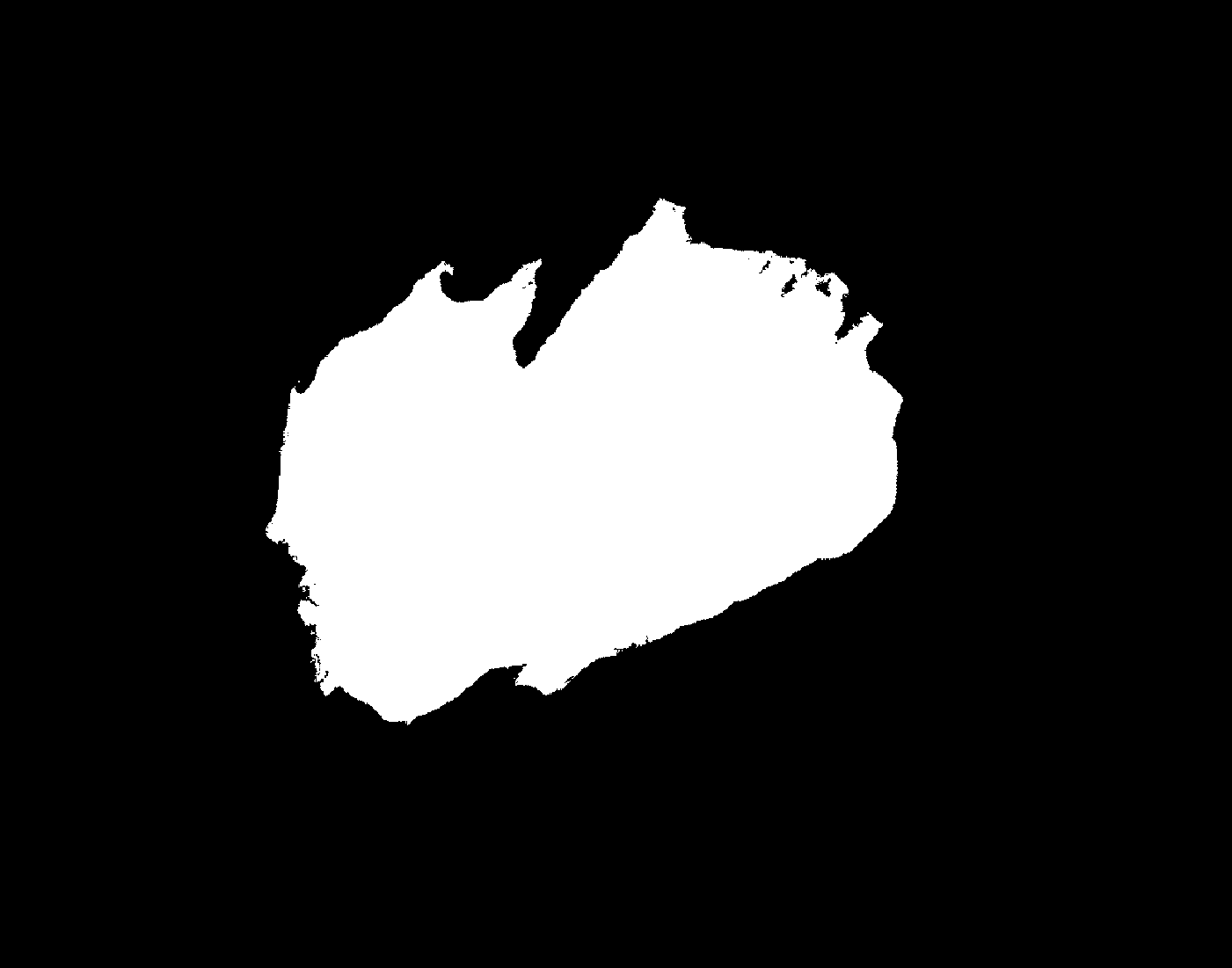

Supplement: Supplementary file 2 [file Datasheet2.zip › figshare/Lesion/Experiment_077.tif]

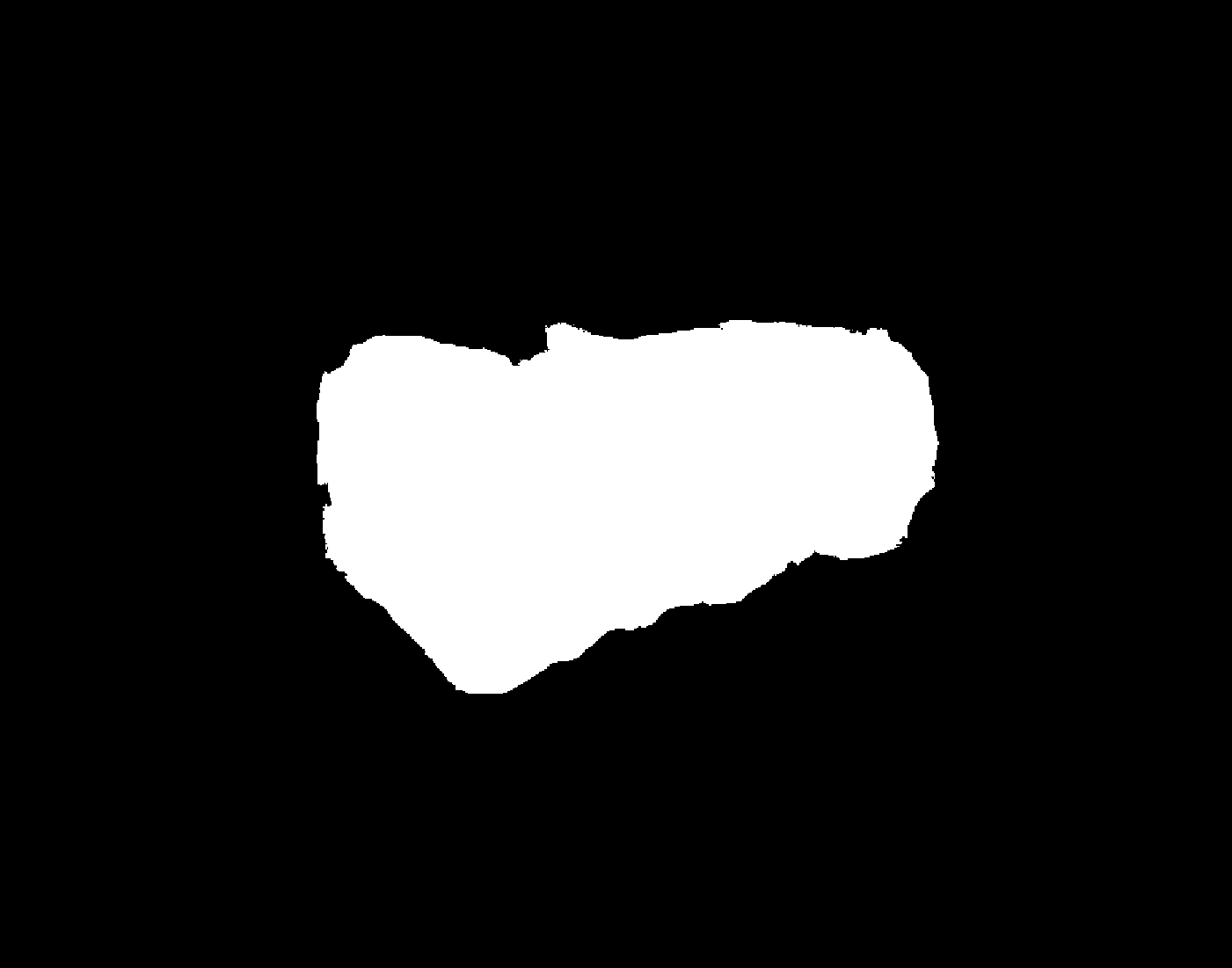

Supplement: Supplementary file 2 [file Datasheet2.zip › figshare/Lesion/Experiment_078.tif]

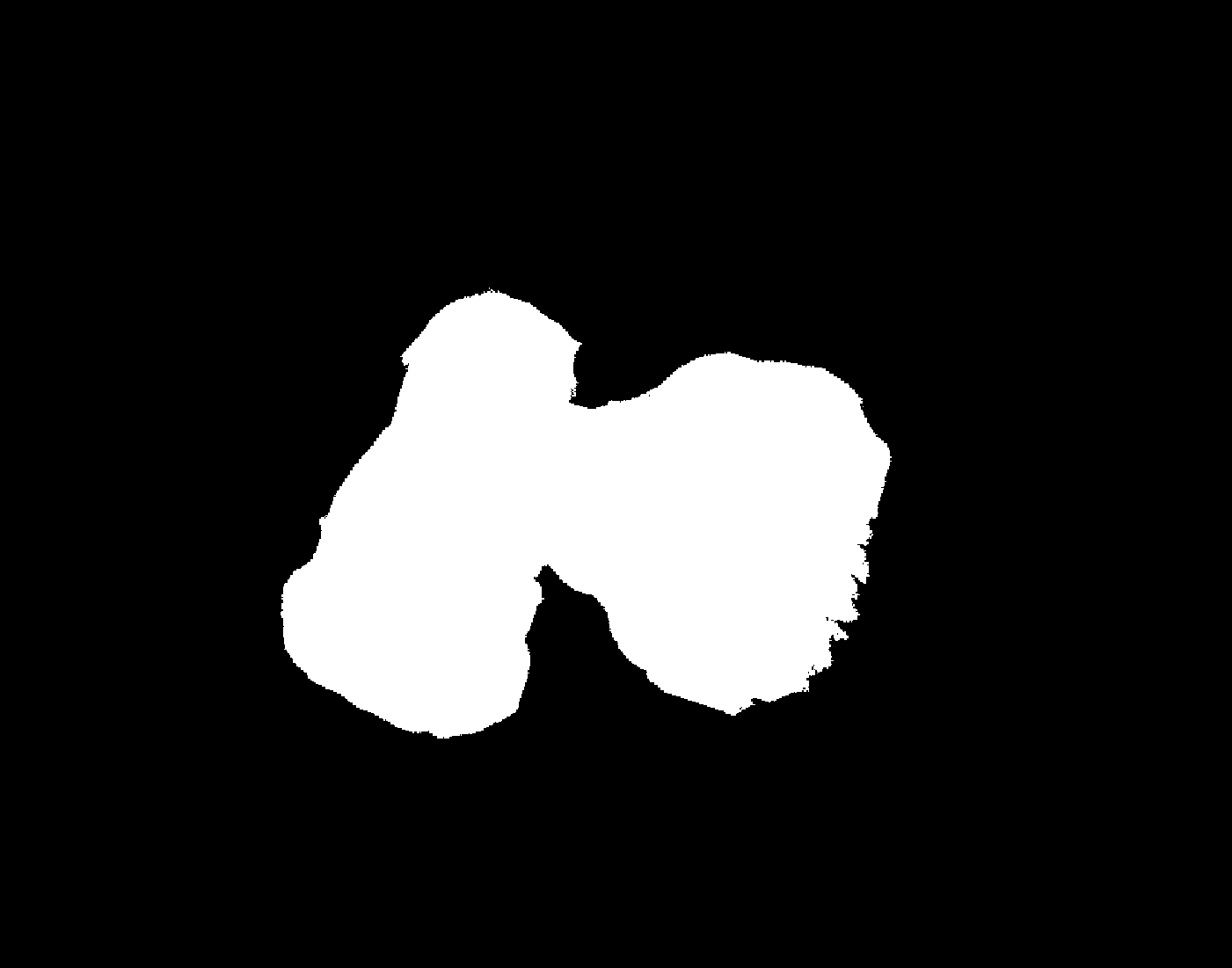

Supplement: Supplementary file 2 [file Datasheet2.zip › figshare/Lesion/Experiment_079.tif]

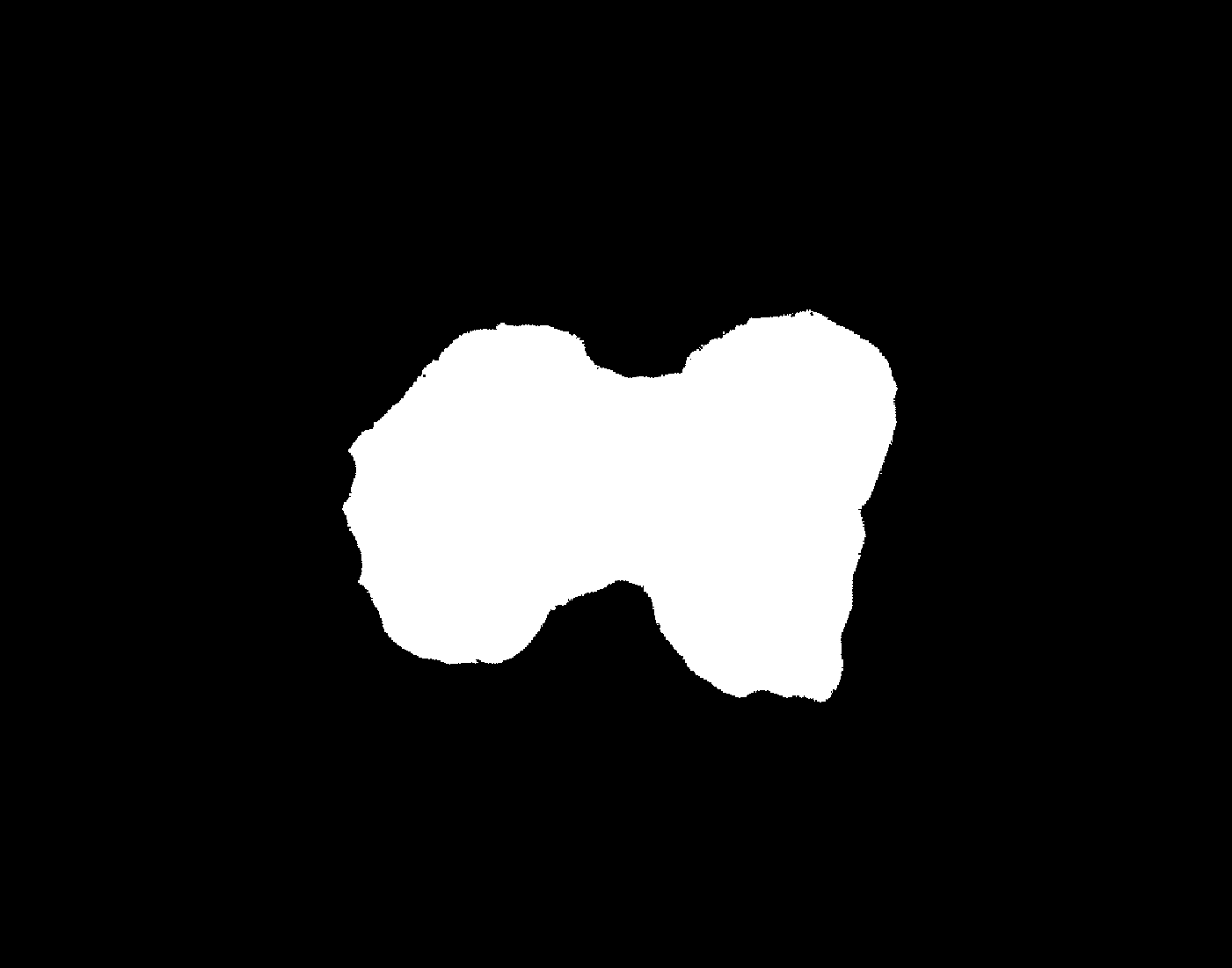

Supplement: Supplementary file 2 [file Datasheet2.zip › figshare/Lesion/Experiment_080.tif]

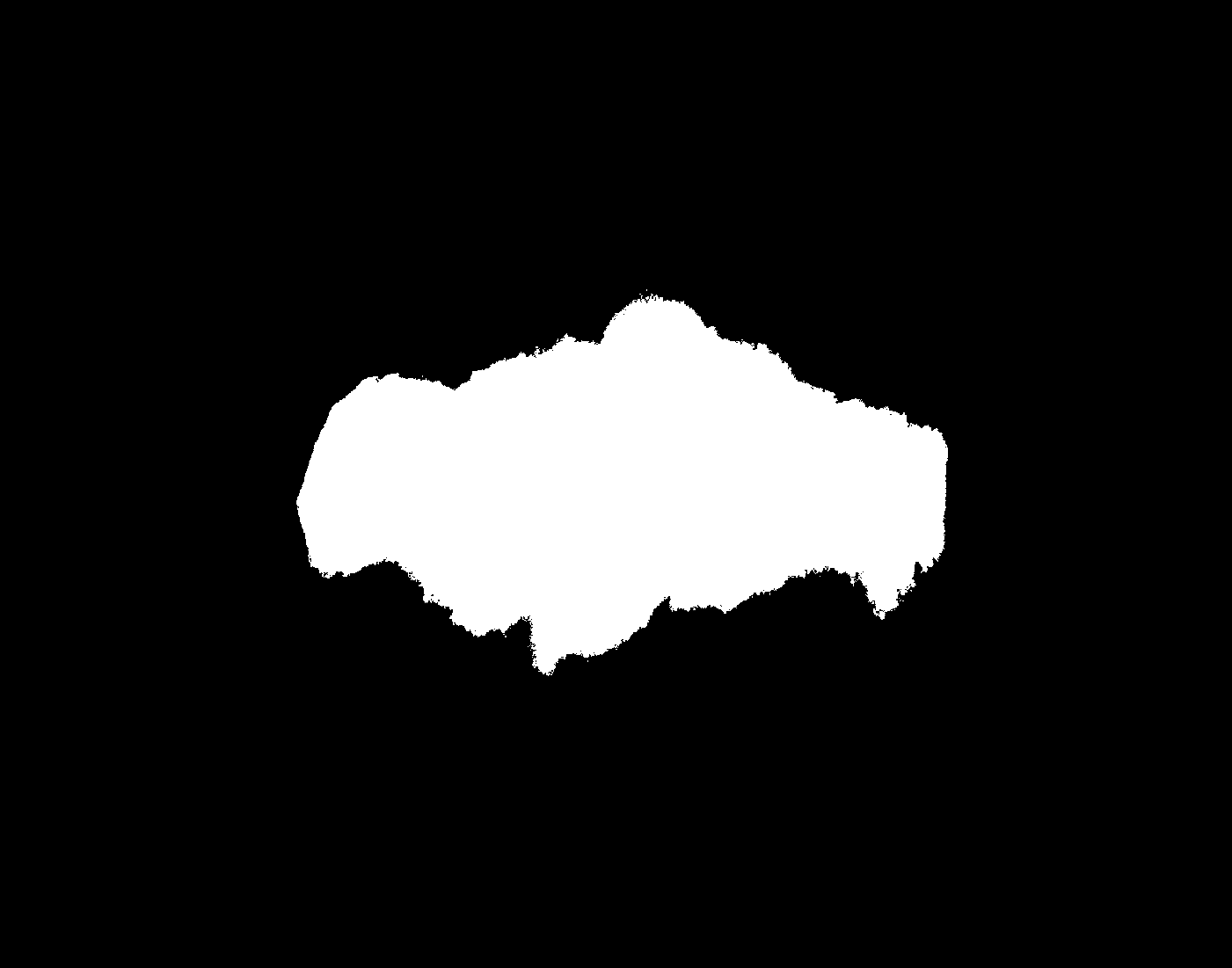

Supplement: Supplementary file 2 [file Datasheet2.zip › figshare/Lesion/Experiment_081.tif]

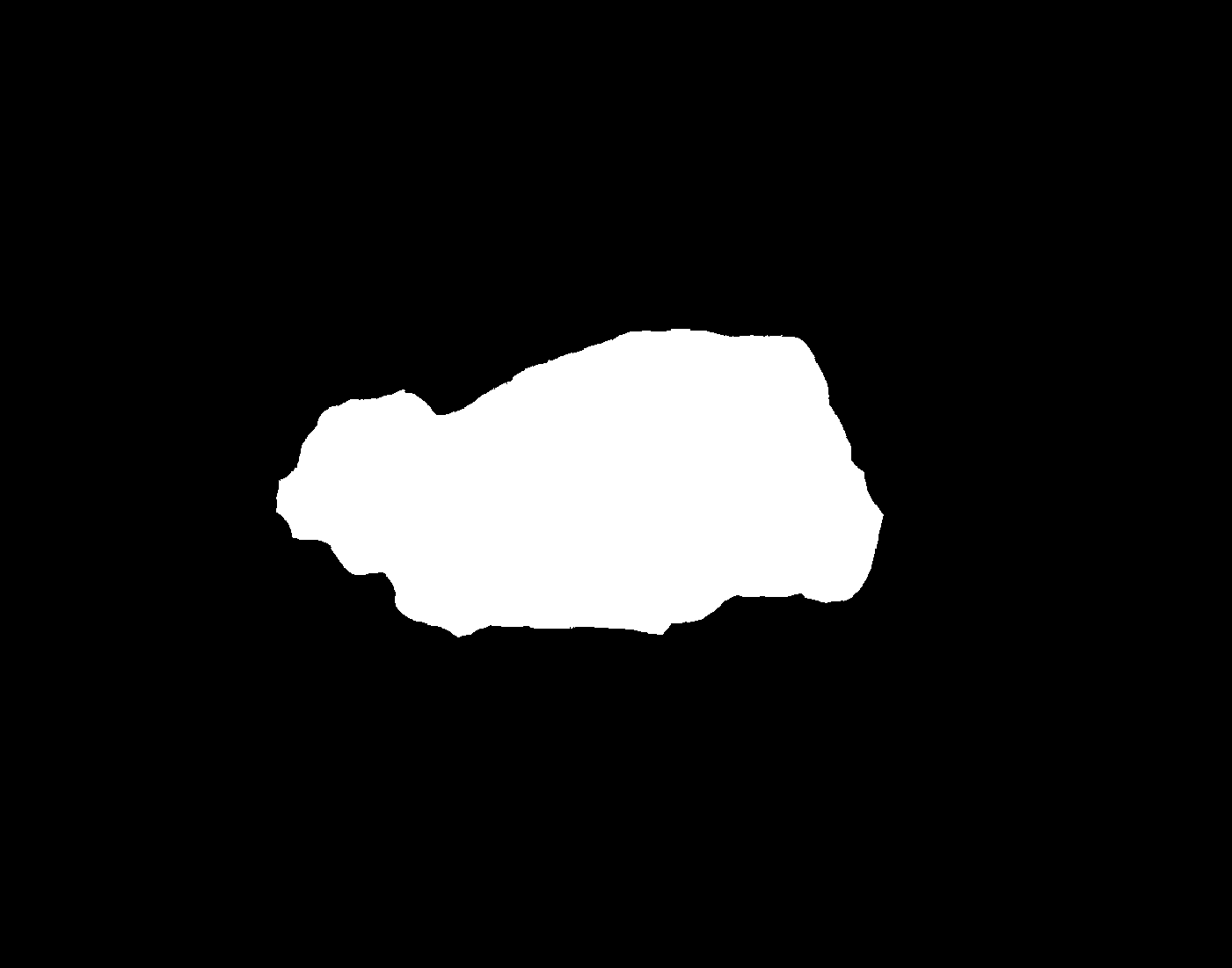

Supplement: Supplementary file 2 [file Datasheet2.zip › figshare/Lesion/Experiment_082.tif]

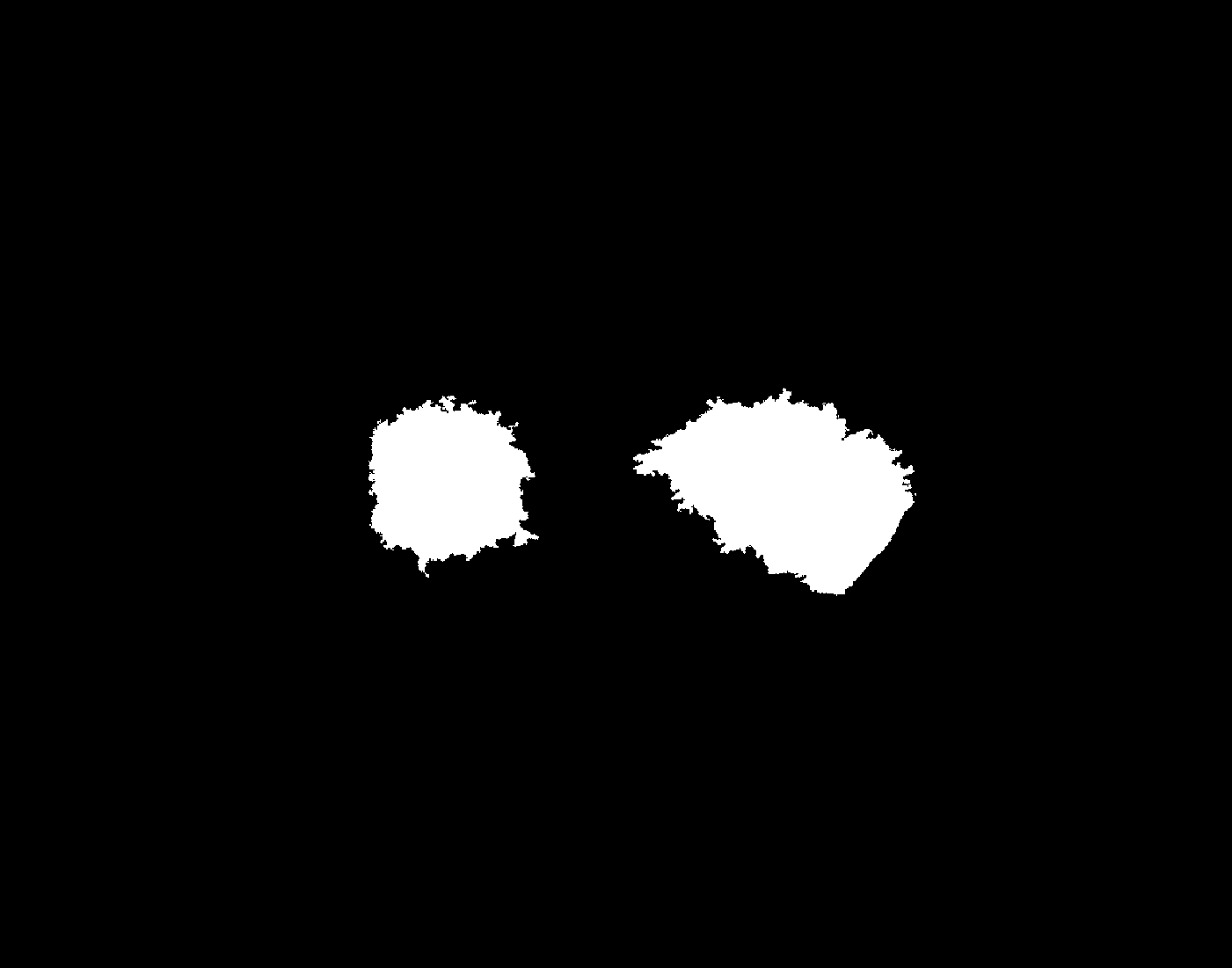

Supplement: Supplementary file 2 [file Datasheet2.zip › figshare/Lesion/Experiment_083.tif]

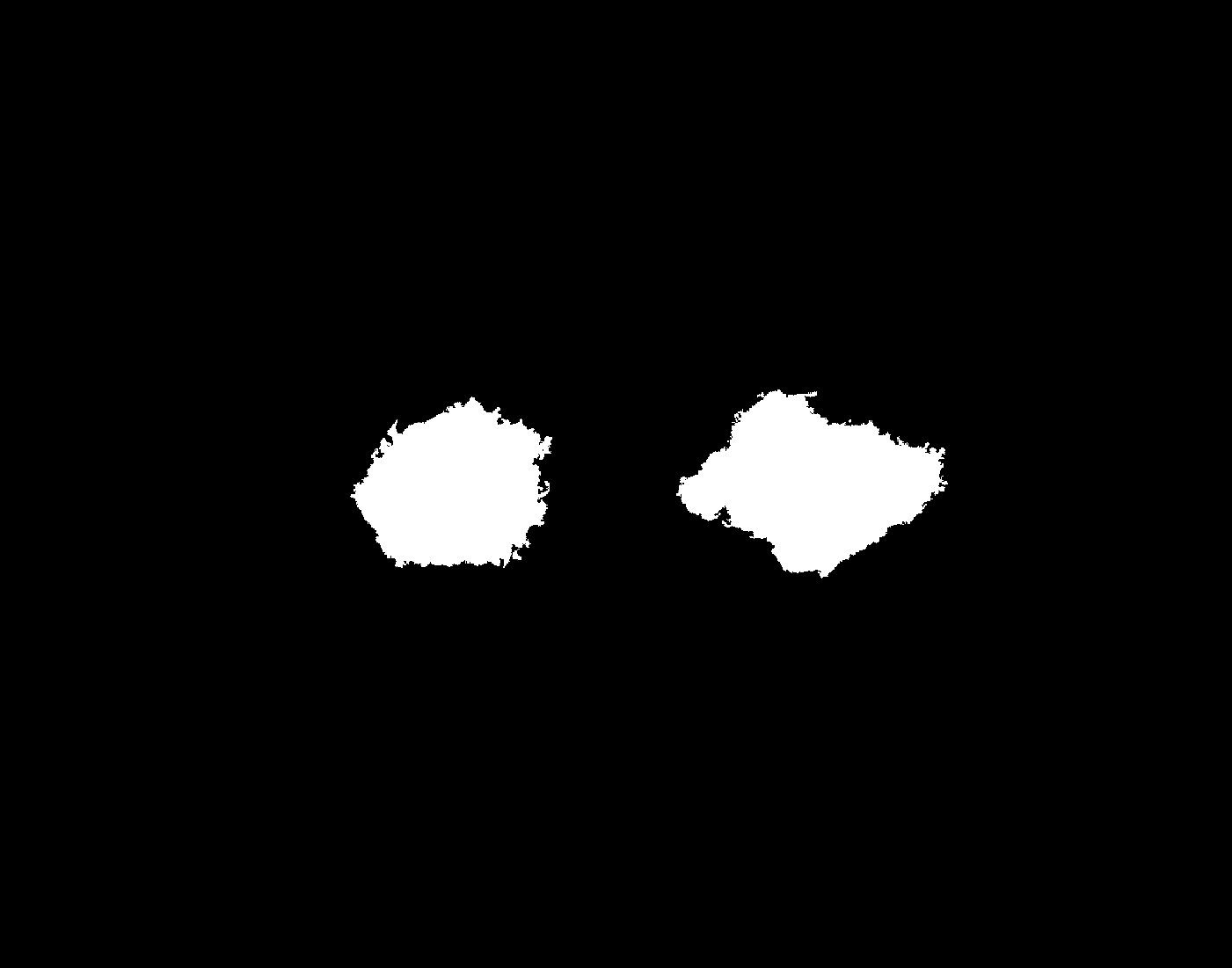

Supplement: Supplementary file 2 [file Datasheet2.zip › figshare/Lesion/Experiment_084.tif]

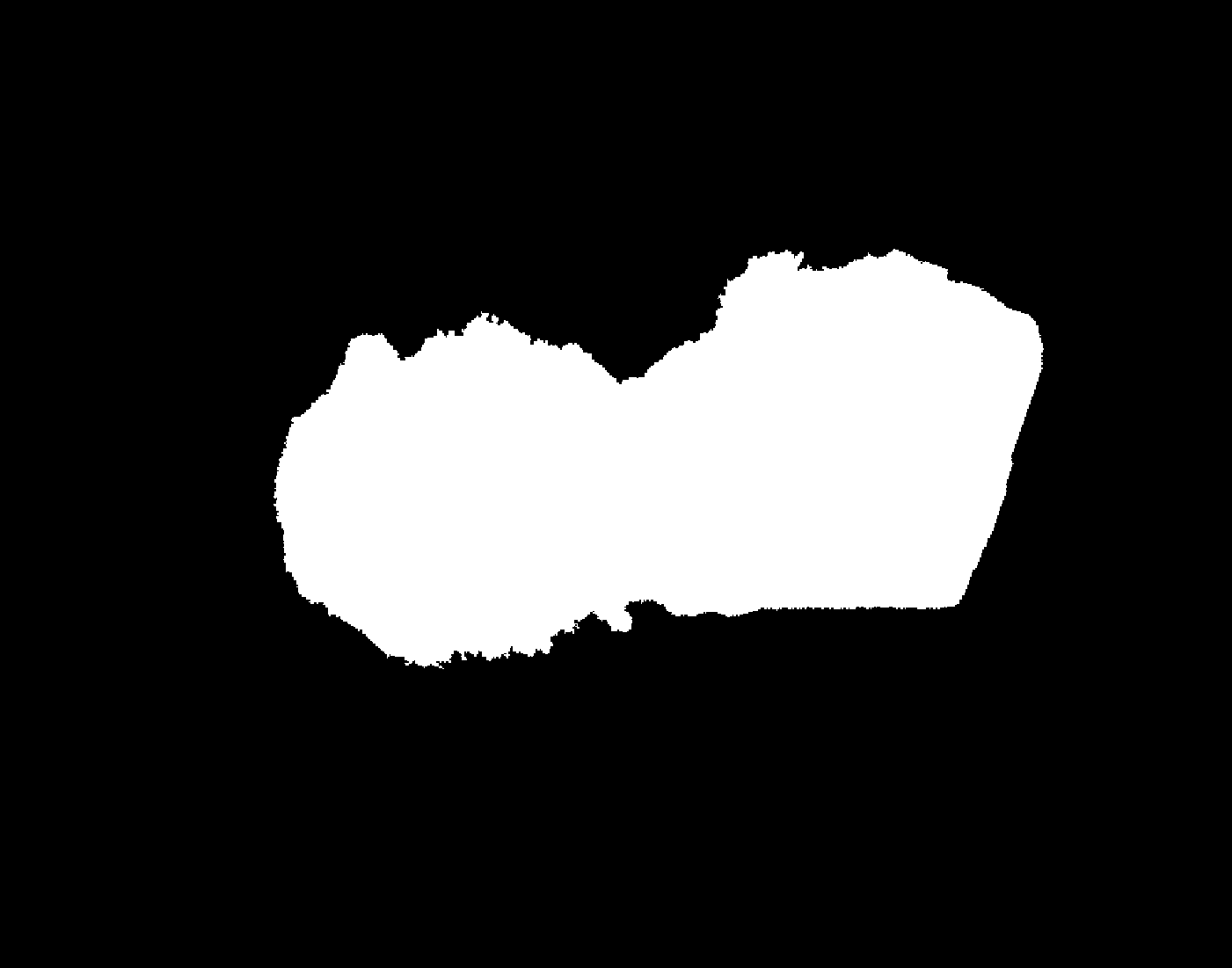

Supplement: Supplementary file 2 [file Datasheet2.zip › figshare/Lesion/Experiment_085.tif]

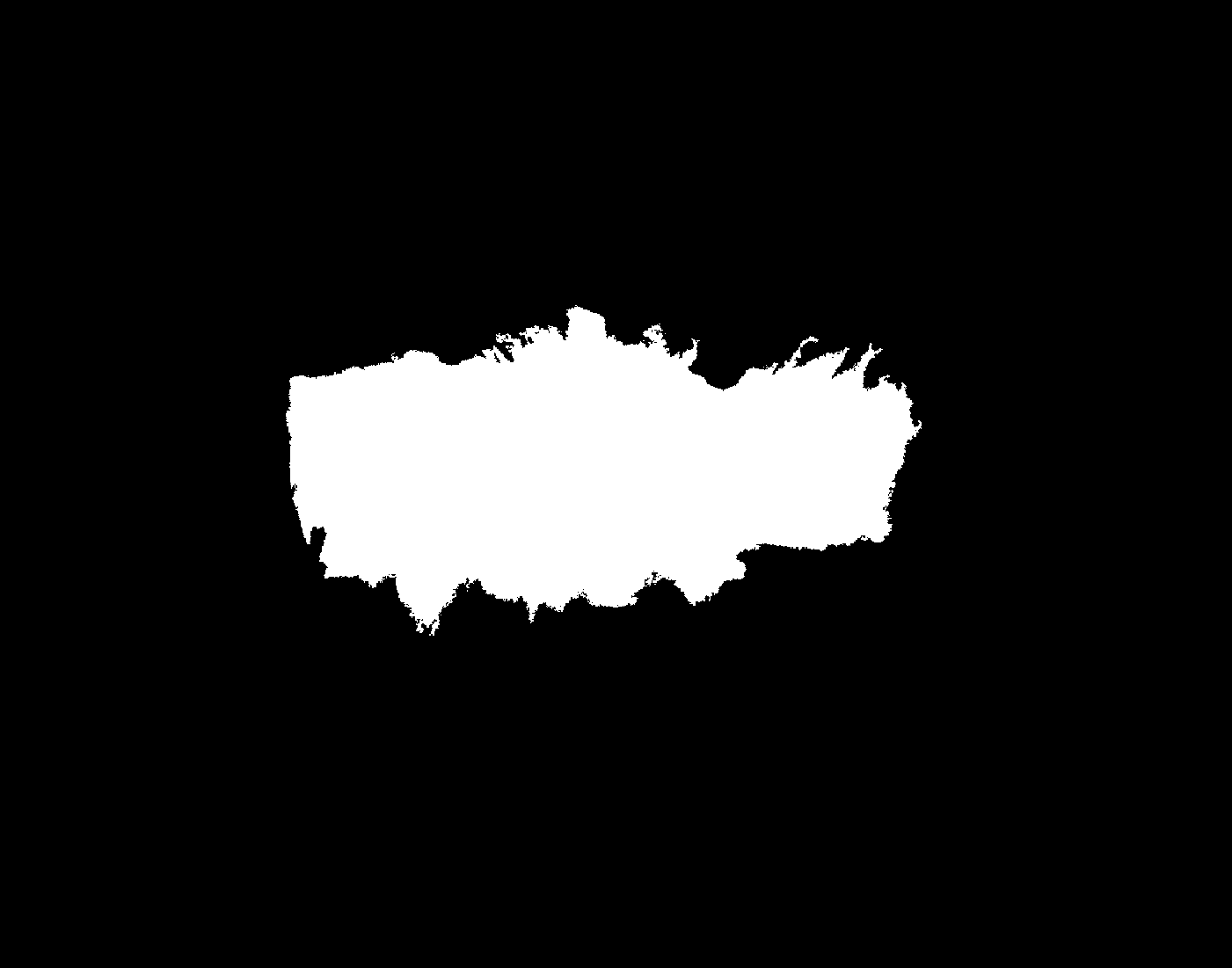

Supplement: Supplementary file 2 [file Datasheet2.zip › figshare/Lesion/Experiment_086.tif]

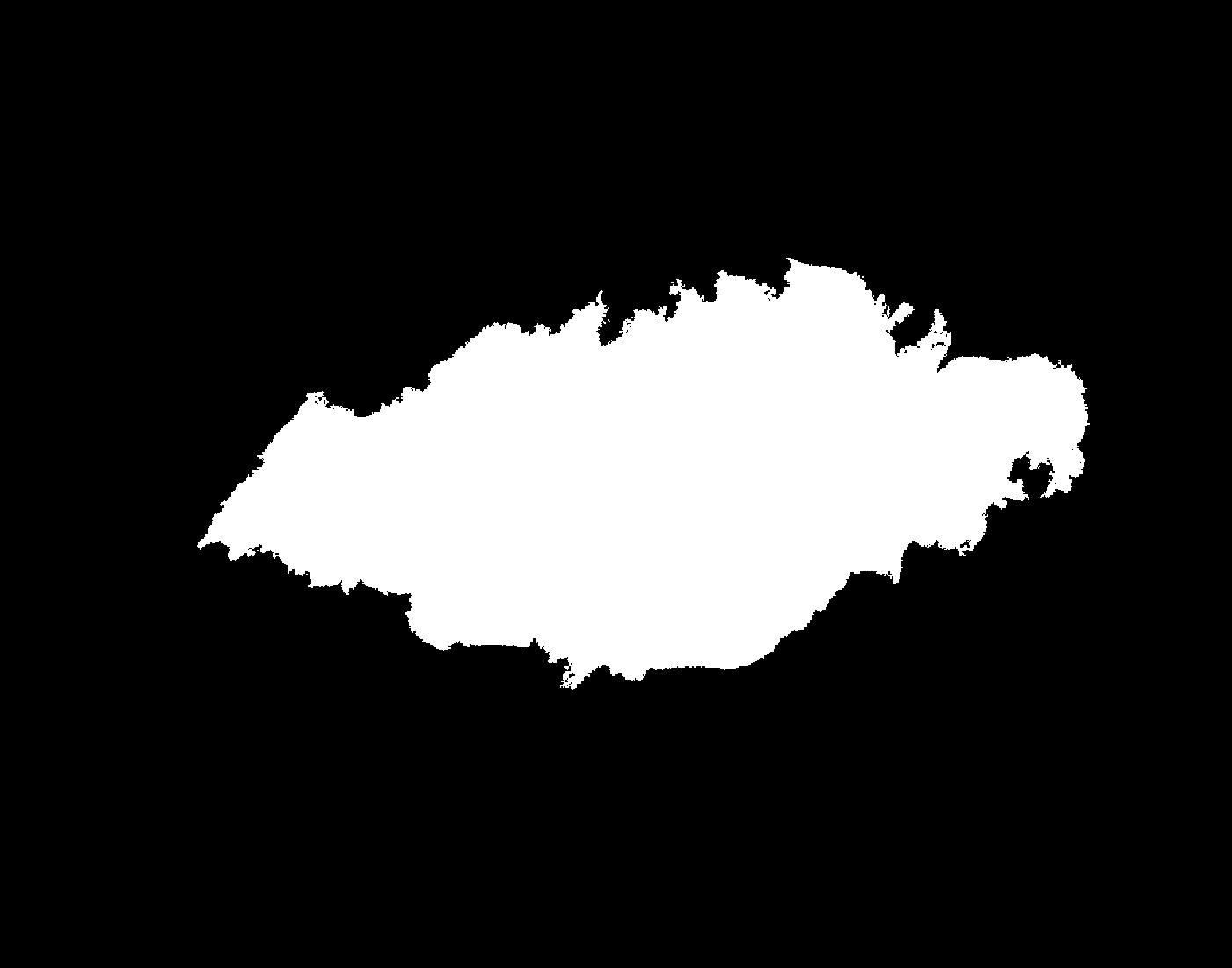

Supplement: Supplementary file 2 [file Datasheet2.zip › figshare/Lesion/Experiment_087.tif]

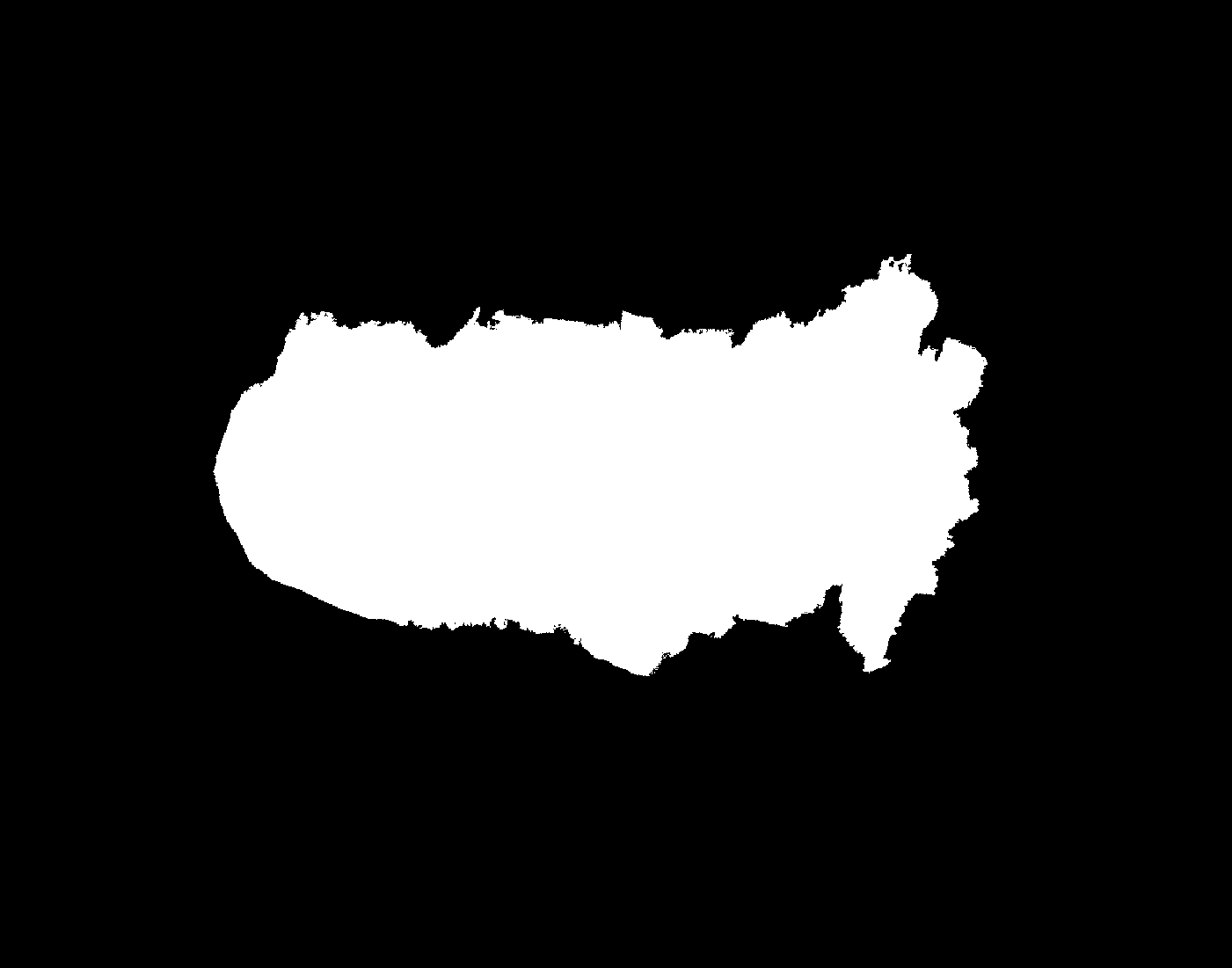

Supplement: Supplementary file 2 [file Datasheet2.zip › figshare/Lesion/Experiment_088.tif]

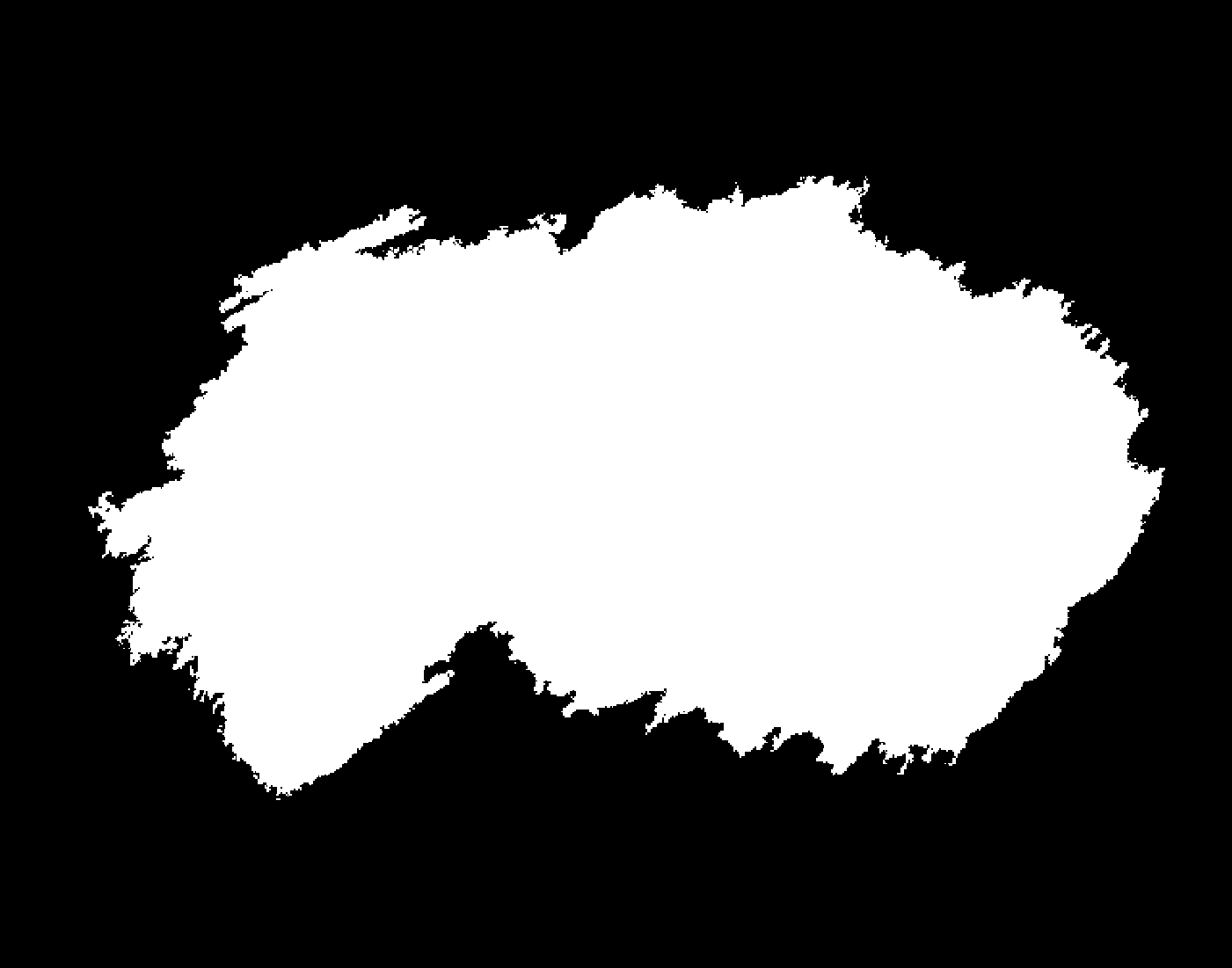

Supplement: Supplementary file 2 [file Datasheet2.zip › figshare/Lesion/Experiment_089.tif]

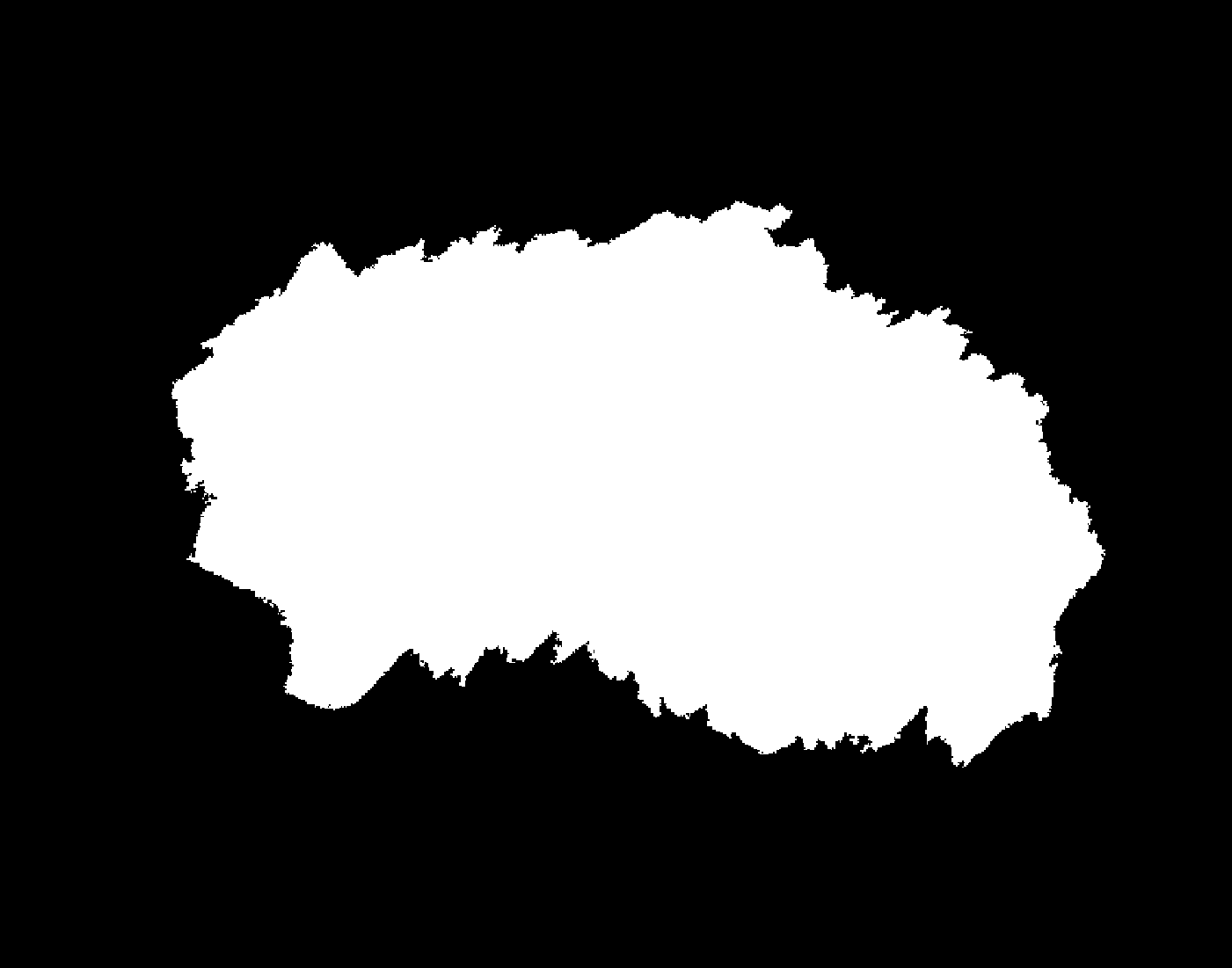

Supplement: Supplementary file 2 [file Datasheet2.zip › figshare/Lesion/Experiment_090.tif]

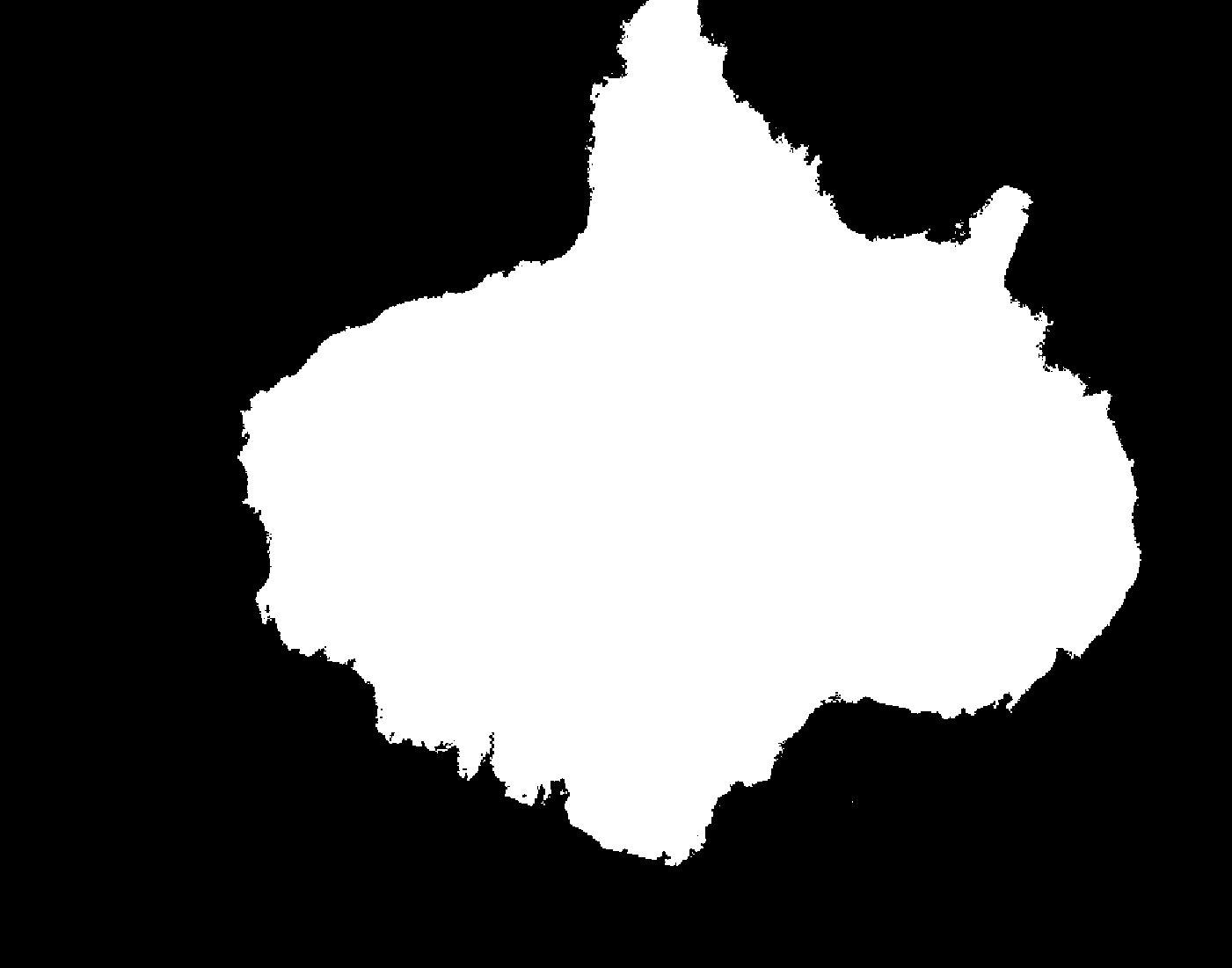

Supplement: Supplementary file 2 [file Datasheet2.zip › figshare/Lesion/Experiment_091.tif]

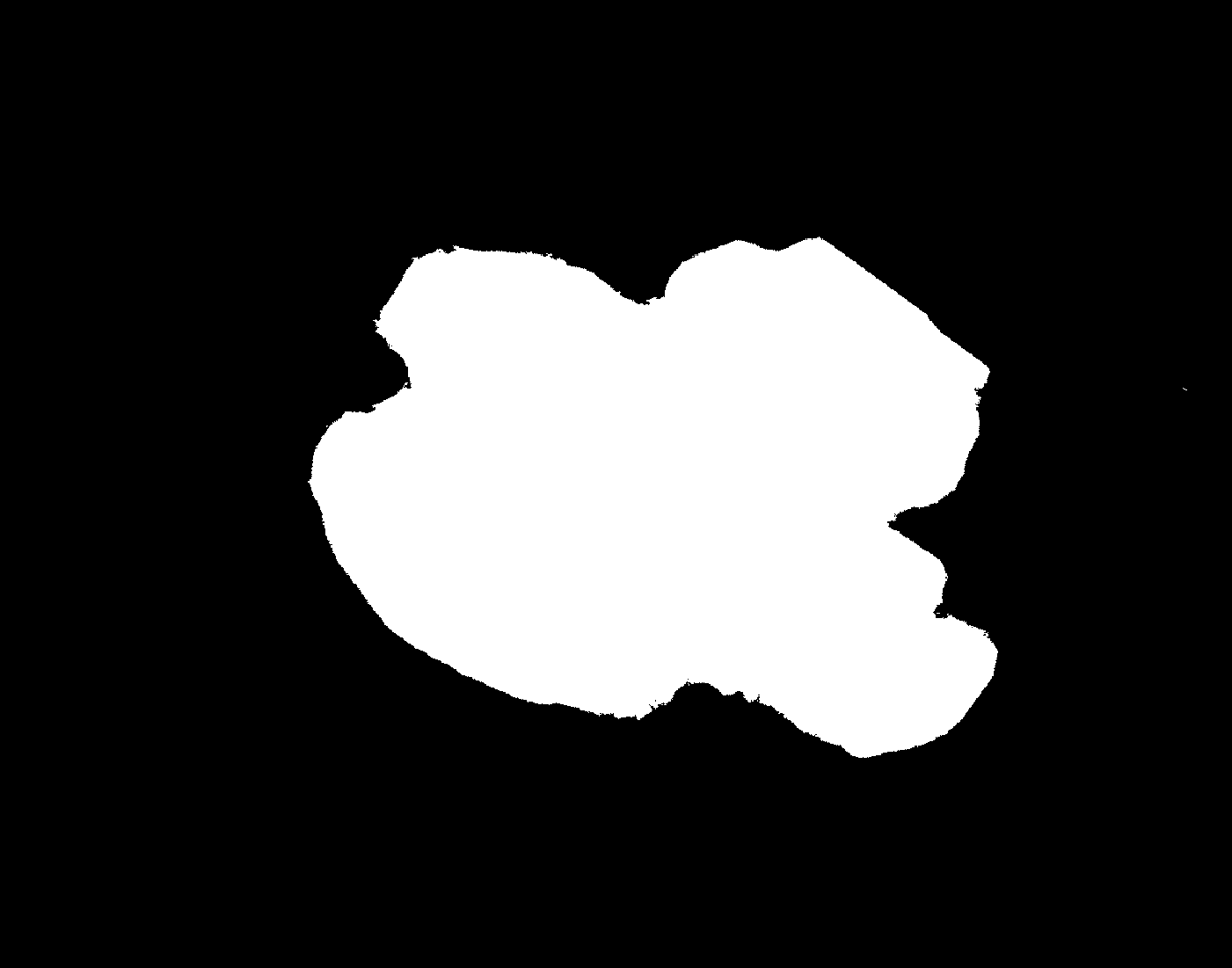

Supplement: Supplementary file 2 [file Datasheet2.zip › figshare/Lesion/Experiment_092.tif]

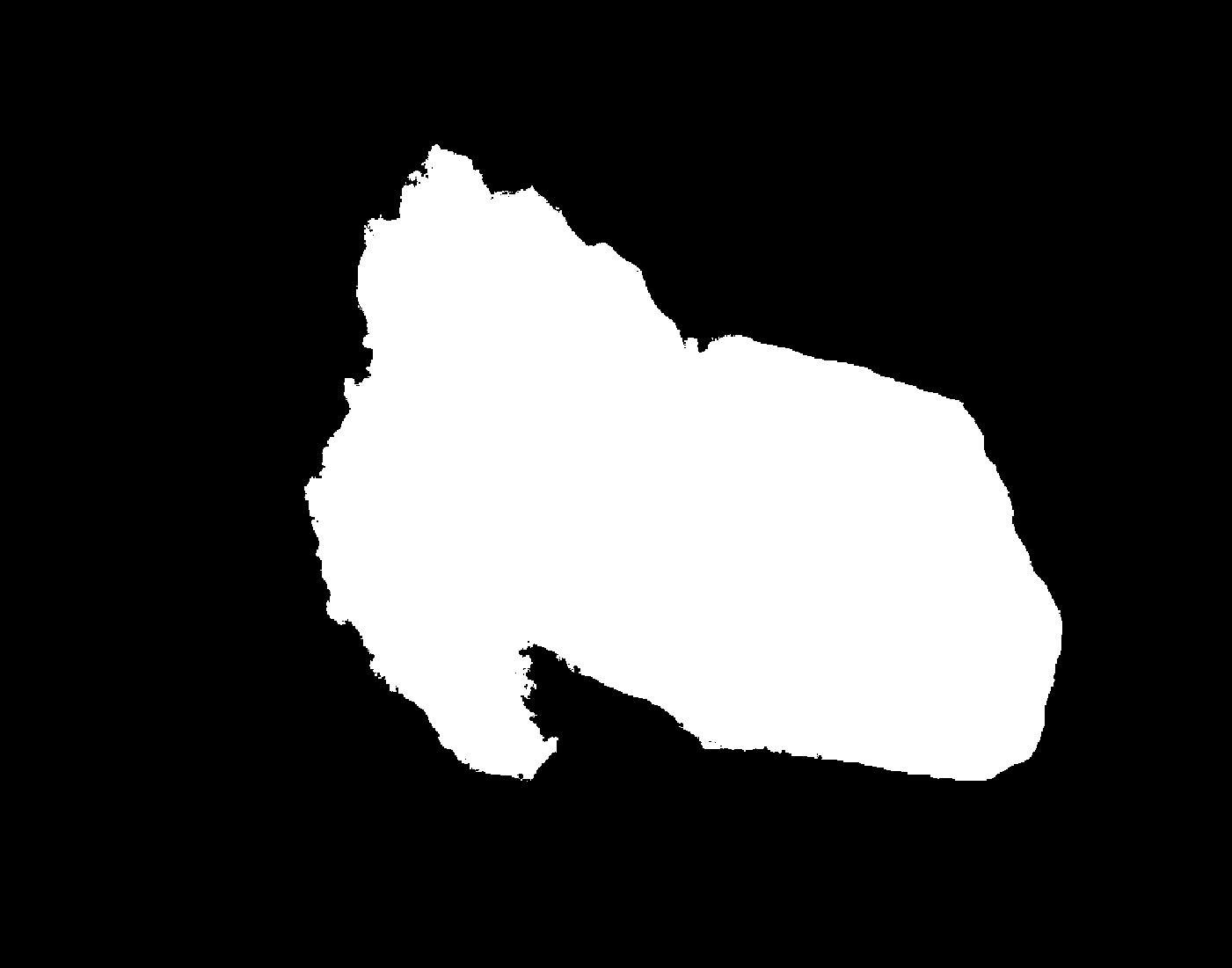

Supplement: Supplementary file 2 [file Datasheet2.zip › figshare/Lesion/Experiment_093.tif]

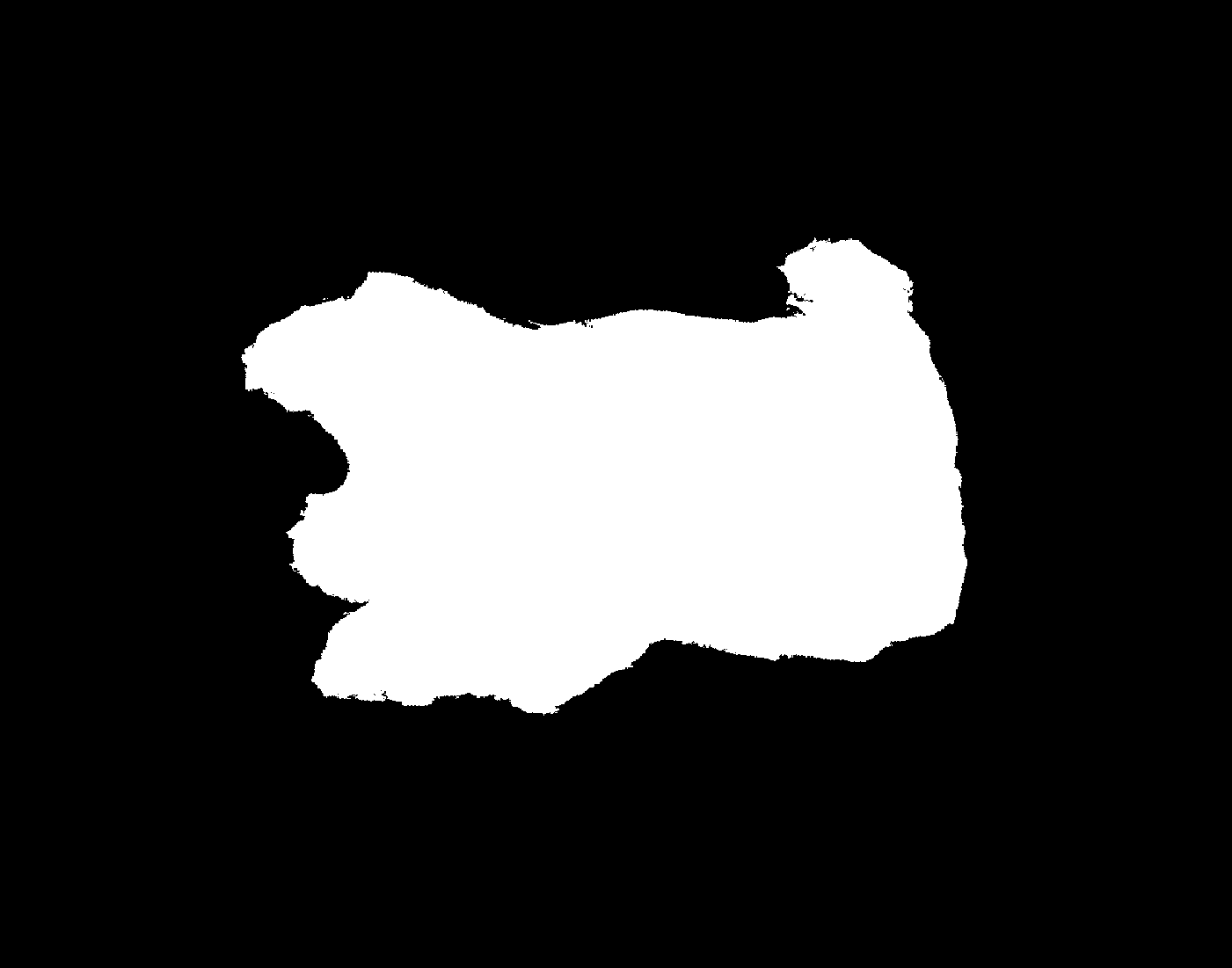

Supplement: Supplementary file 2 [file Datasheet2.zip › figshare/Lesion/Experiment_094.tif]

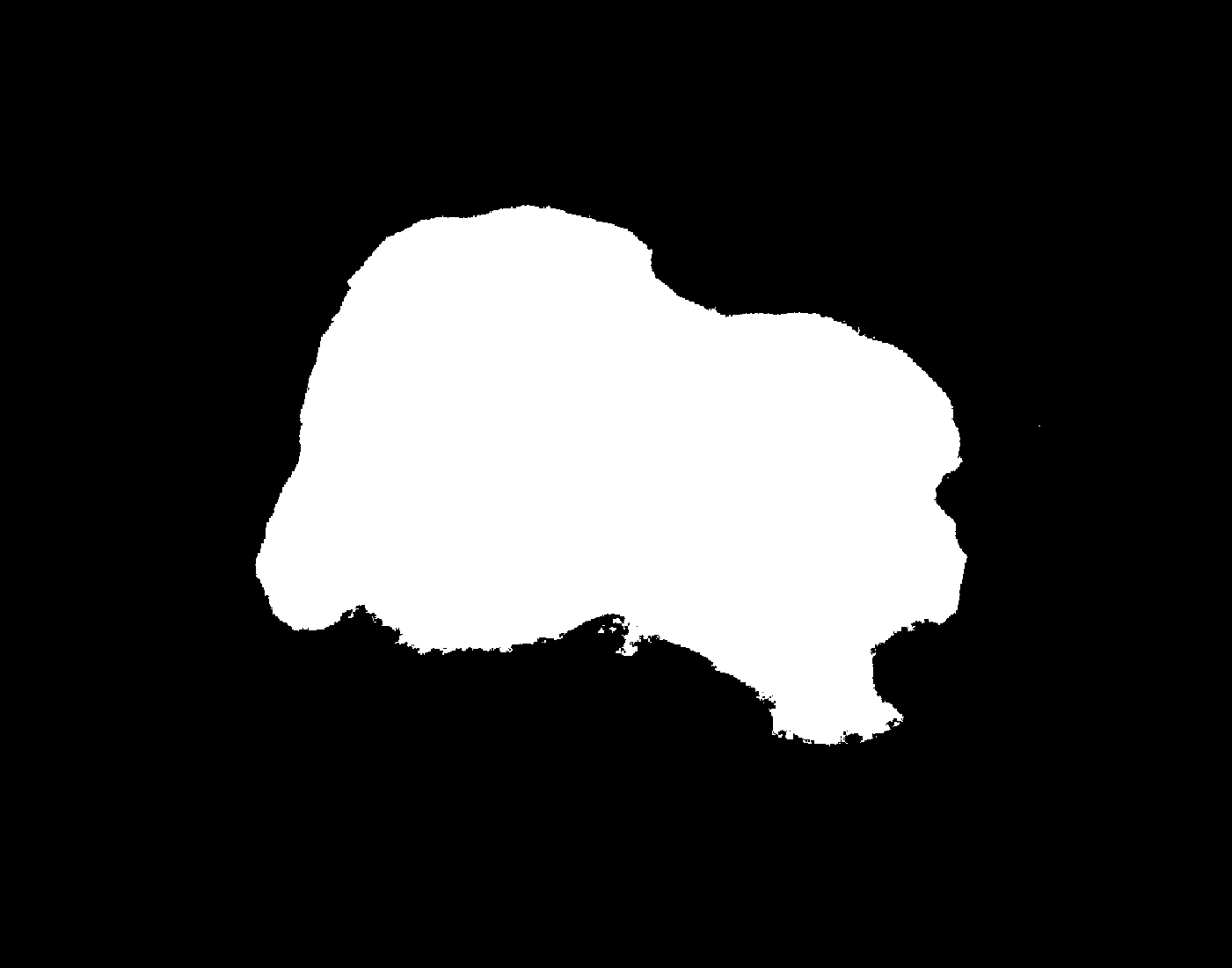

Supplement: Supplementary file 2 [file Datasheet2.zip › figshare/Lesion/Experiment_095.tif]

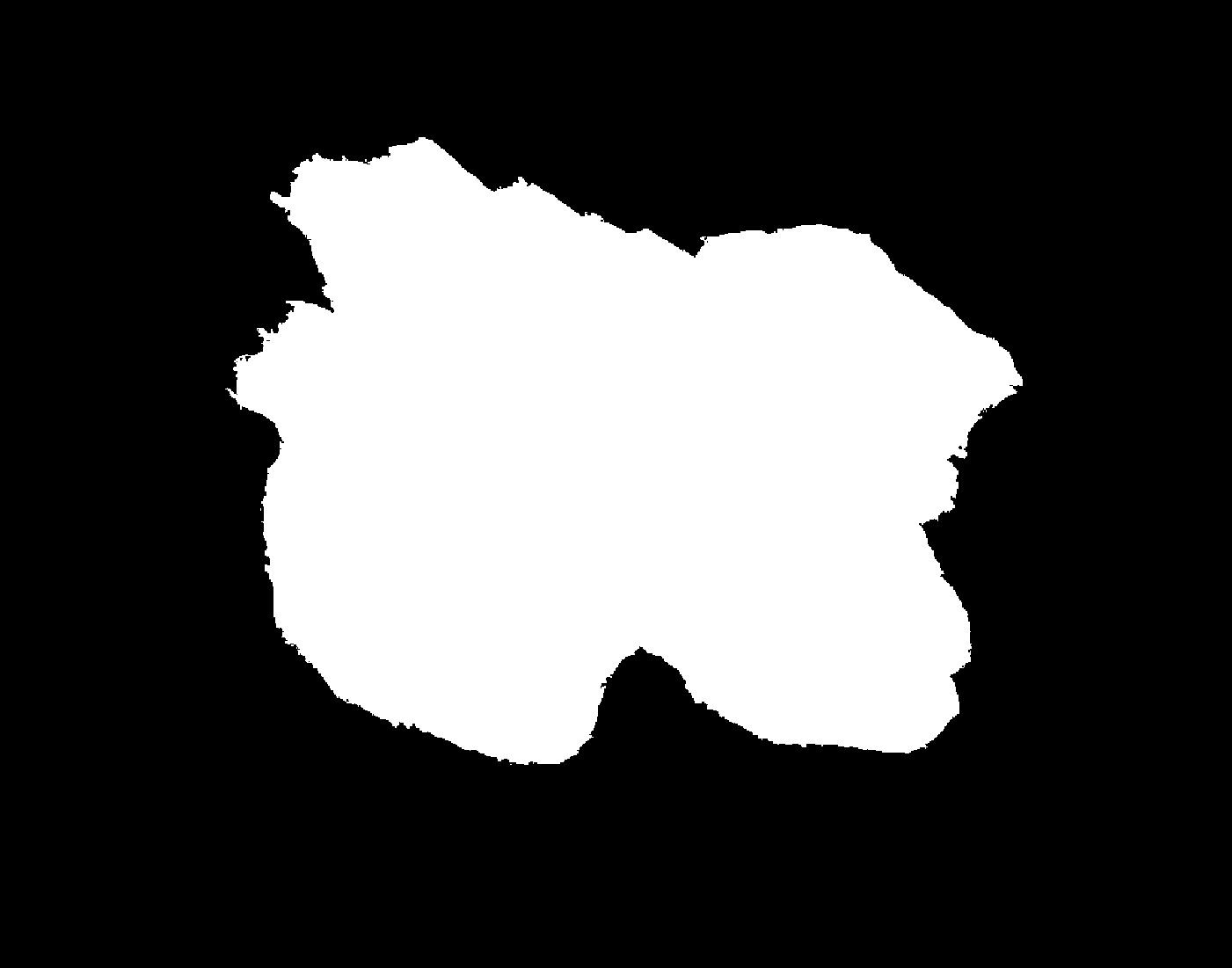

Supplement: Supplementary file 2 [file Datasheet2.zip › figshare/Lesion/Experiment_096.tif]
