# Supplementary figures and images for: Determination of lethal electric field threshold for pulsed field ablation in ex vivo perfused porcine and human hearts (part 1 of 2)
Source: Front Cardiovasc Med. 2023 Jun 23;10:1160231. doi: 10.3389/fcvm.2023.1160231 (PMC10326317; doi:10.3389/fcvm.2023.1160231)

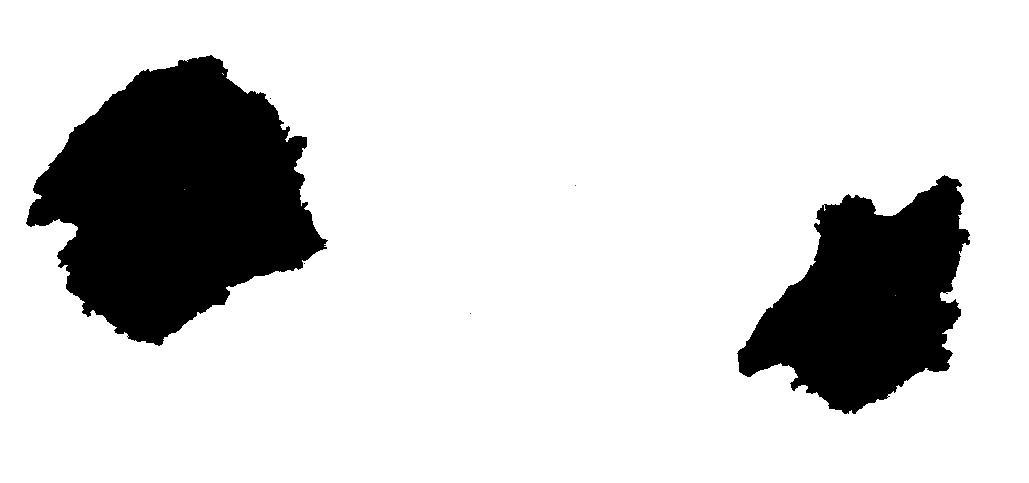

Supplement: Supplementary file 2 [file Datasheet2.zip › figshare/ImageIn/Experiment_001.tif]

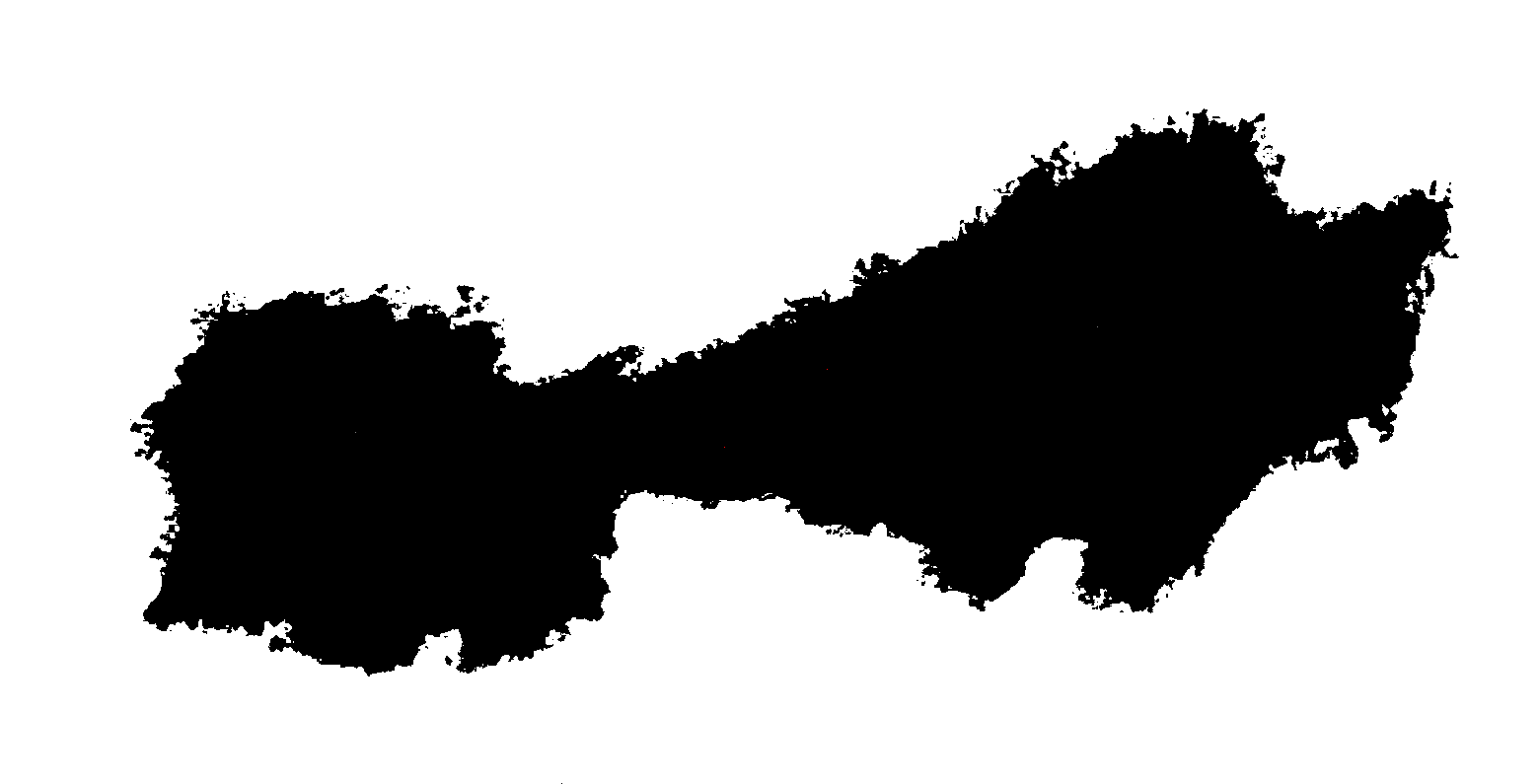

Supplement: Supplementary file 2 [file Datasheet2.zip › figshare/ImageIn/Experiment_002.tif]

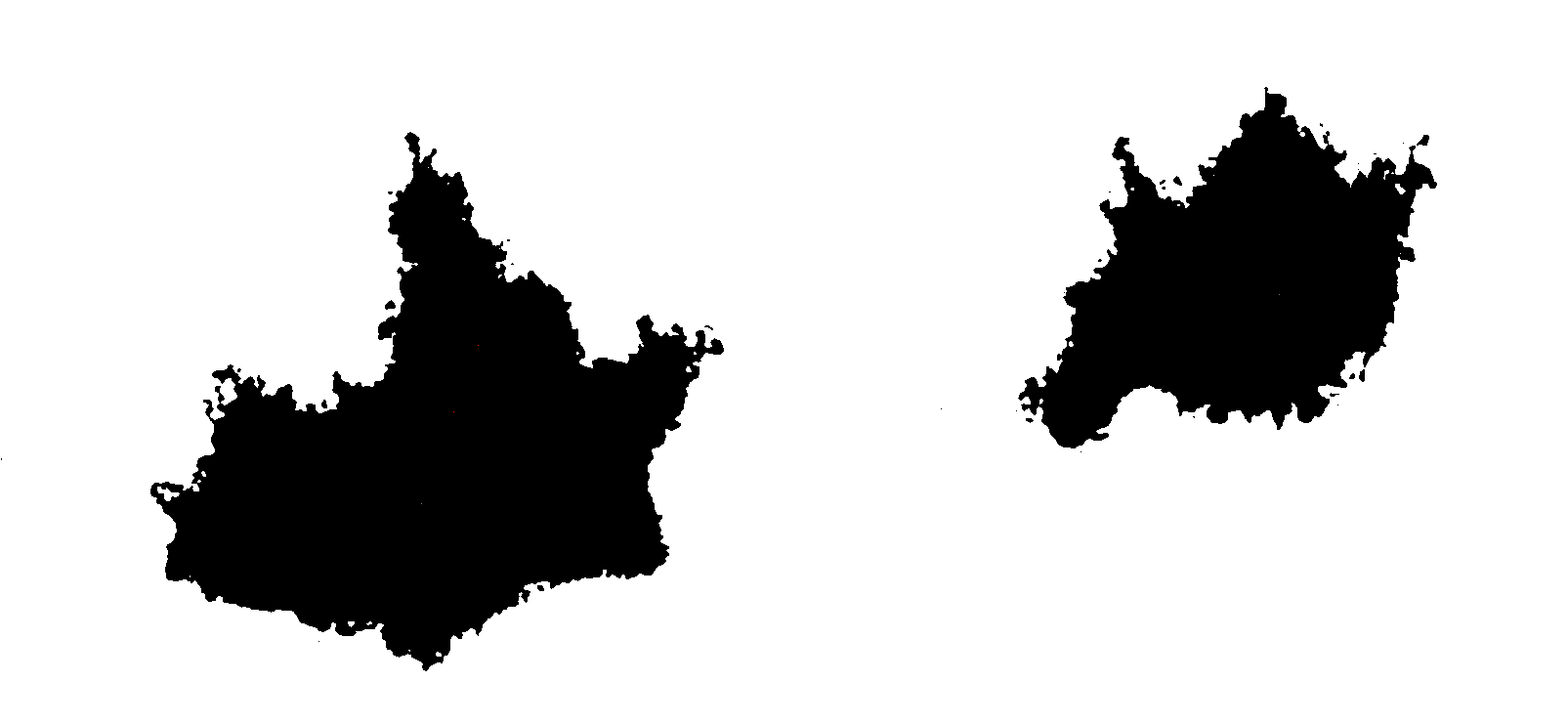

Supplement: Supplementary file 2 [file Datasheet2.zip › figshare/ImageIn/Experiment_003.tif]

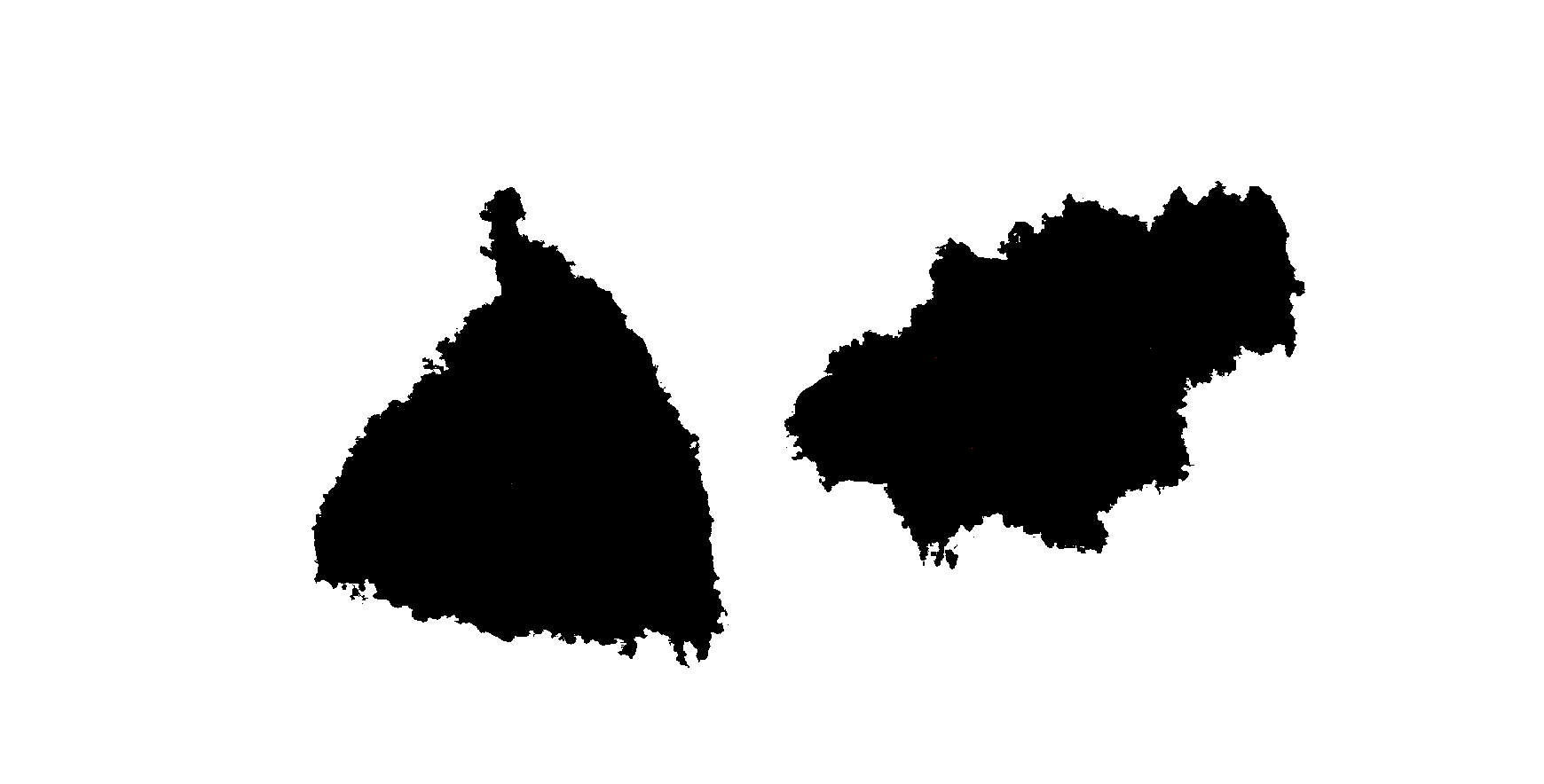

Supplement: Supplementary file 2 [file Datasheet2.zip › figshare/ImageIn/Experiment_004.tif]

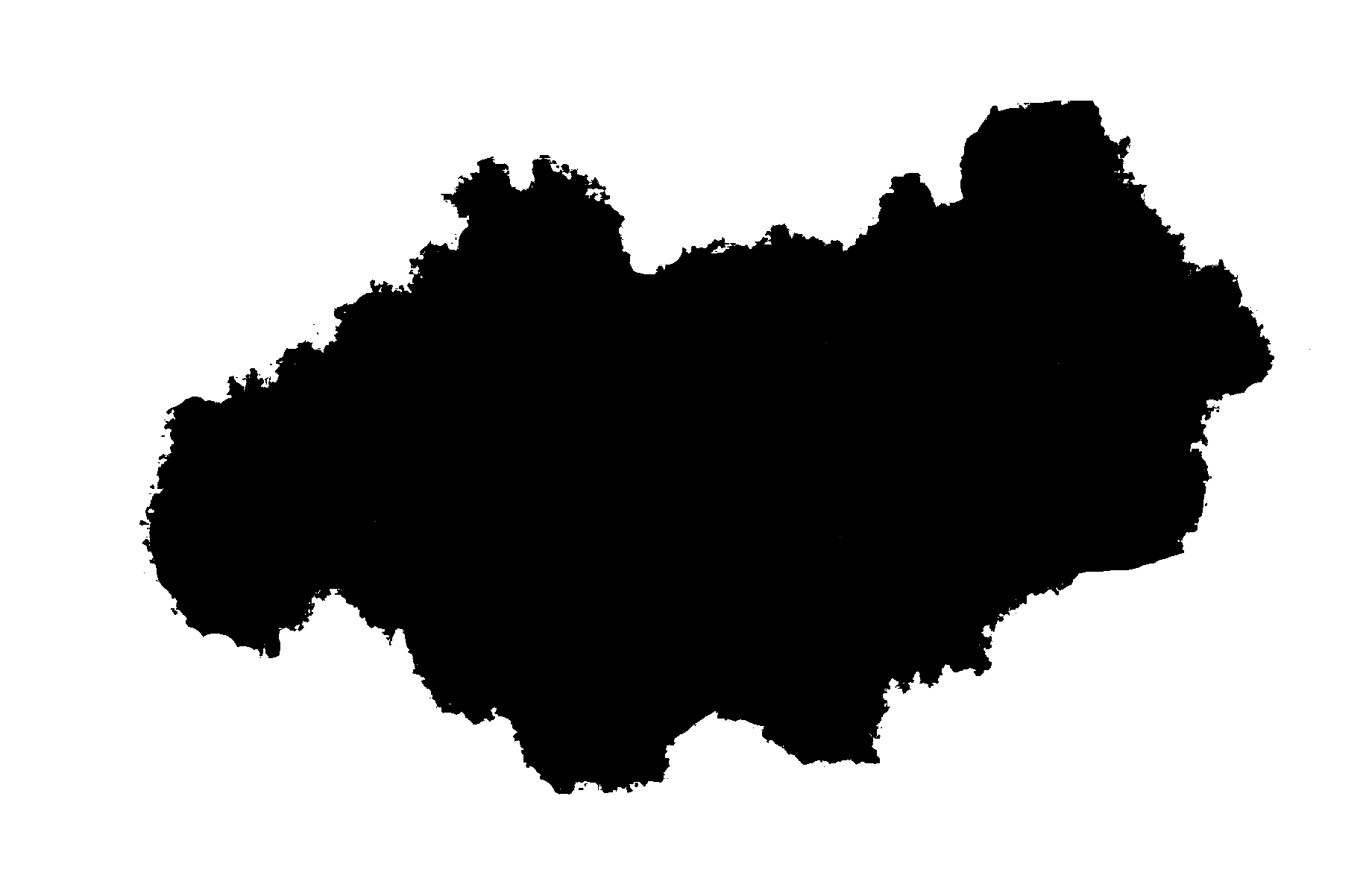

Supplement: Supplementary file 2 [file Datasheet2.zip › figshare/ImageIn/Experiment_005.tif]

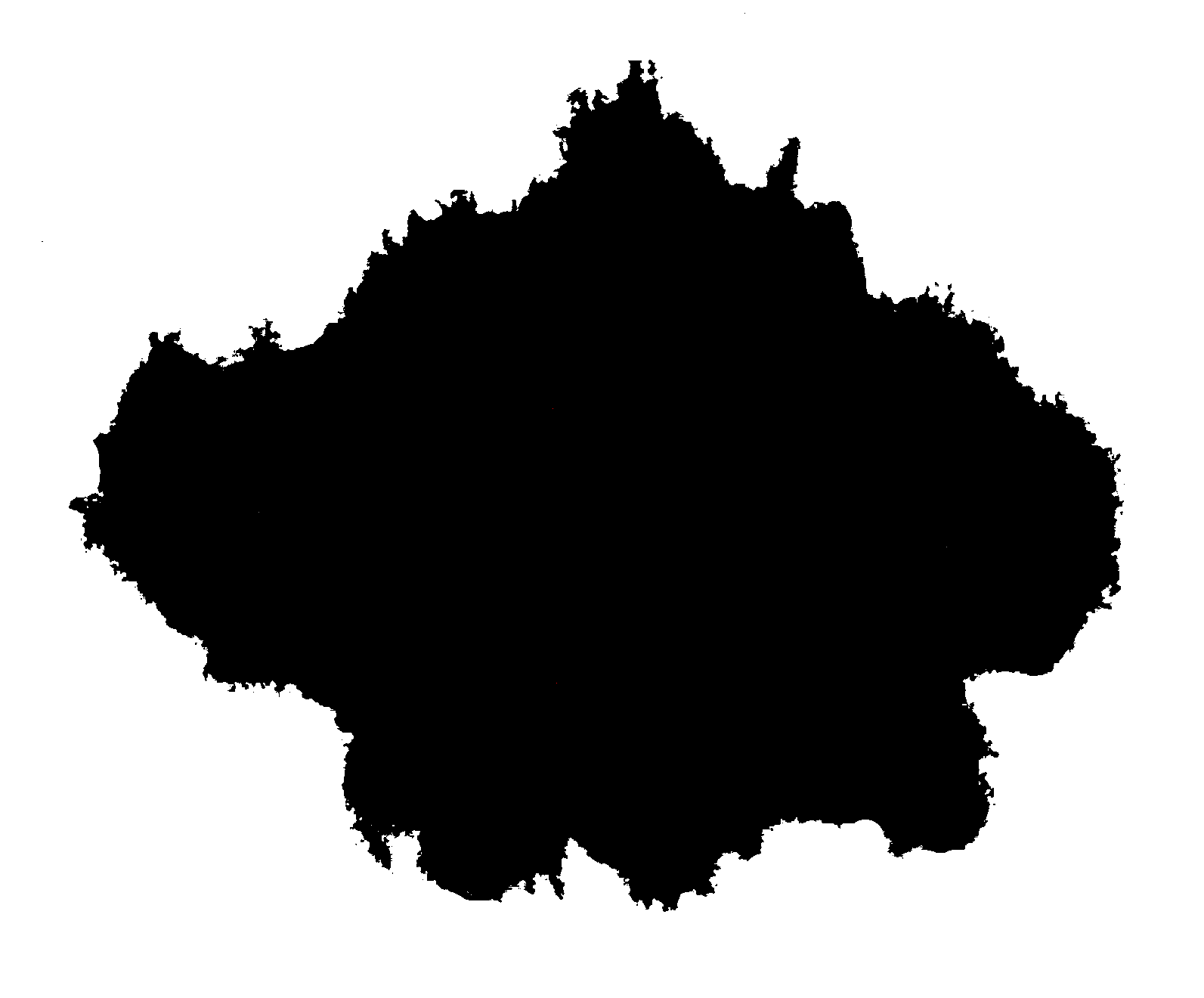

Supplement: Supplementary file 2 [file Datasheet2.zip › figshare/ImageIn/Experiment_006.tif]

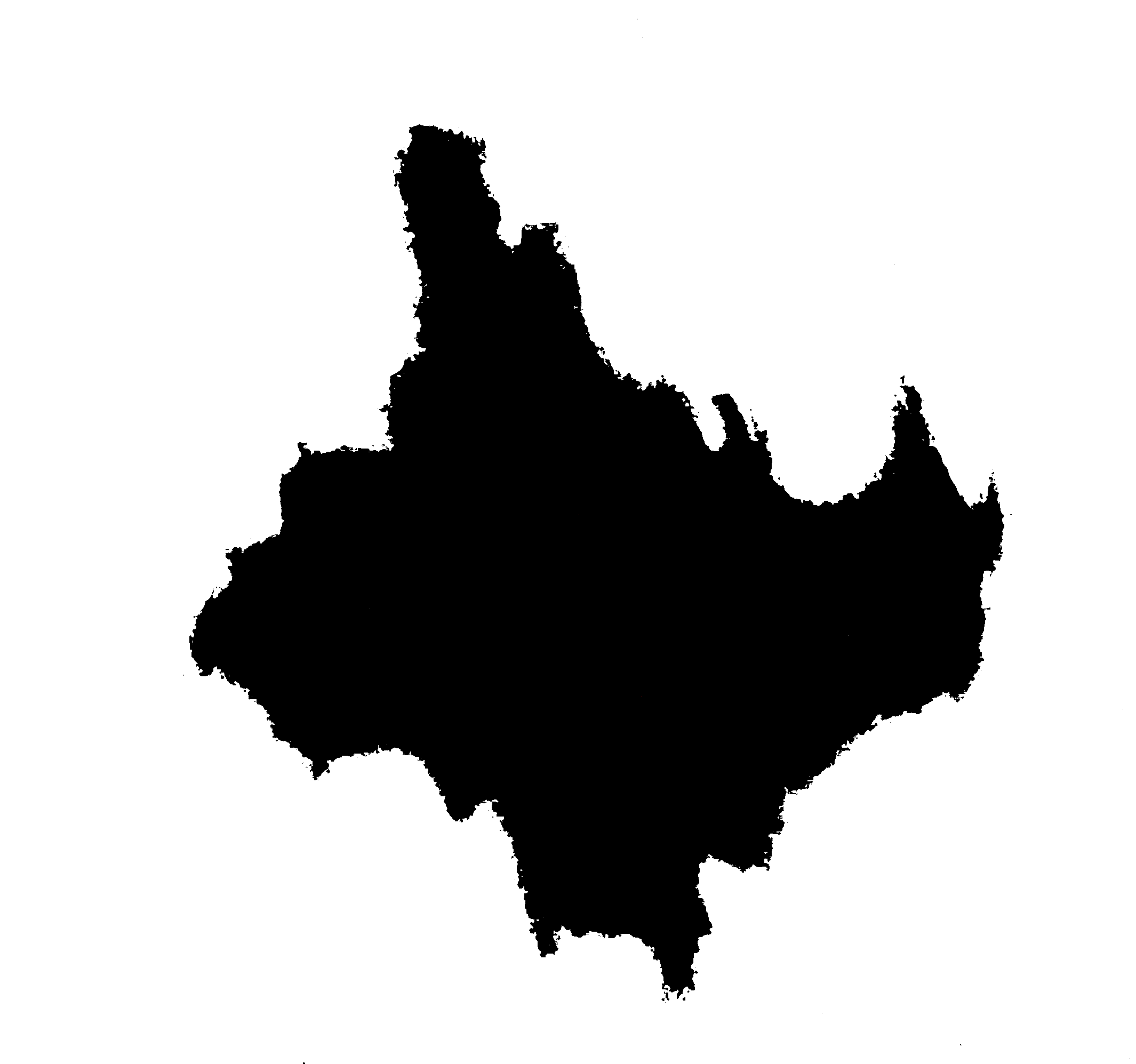

Supplement: Supplementary file 2 [file Datasheet2.zip › figshare/ImageIn/Experiment_007.tif]

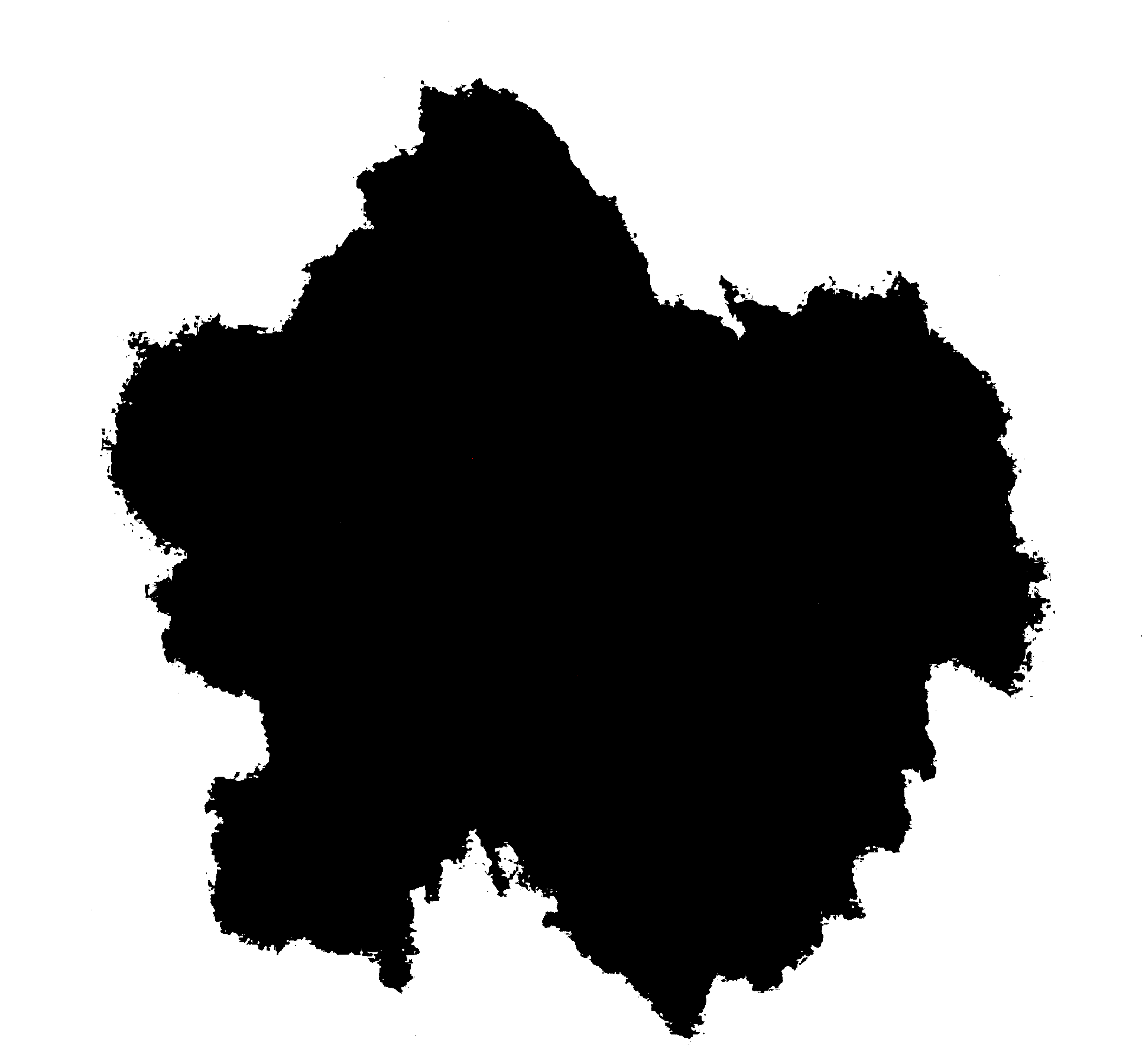

Supplement: Supplementary file 2 [file Datasheet2.zip › figshare/ImageIn/Experiment_008.tif]

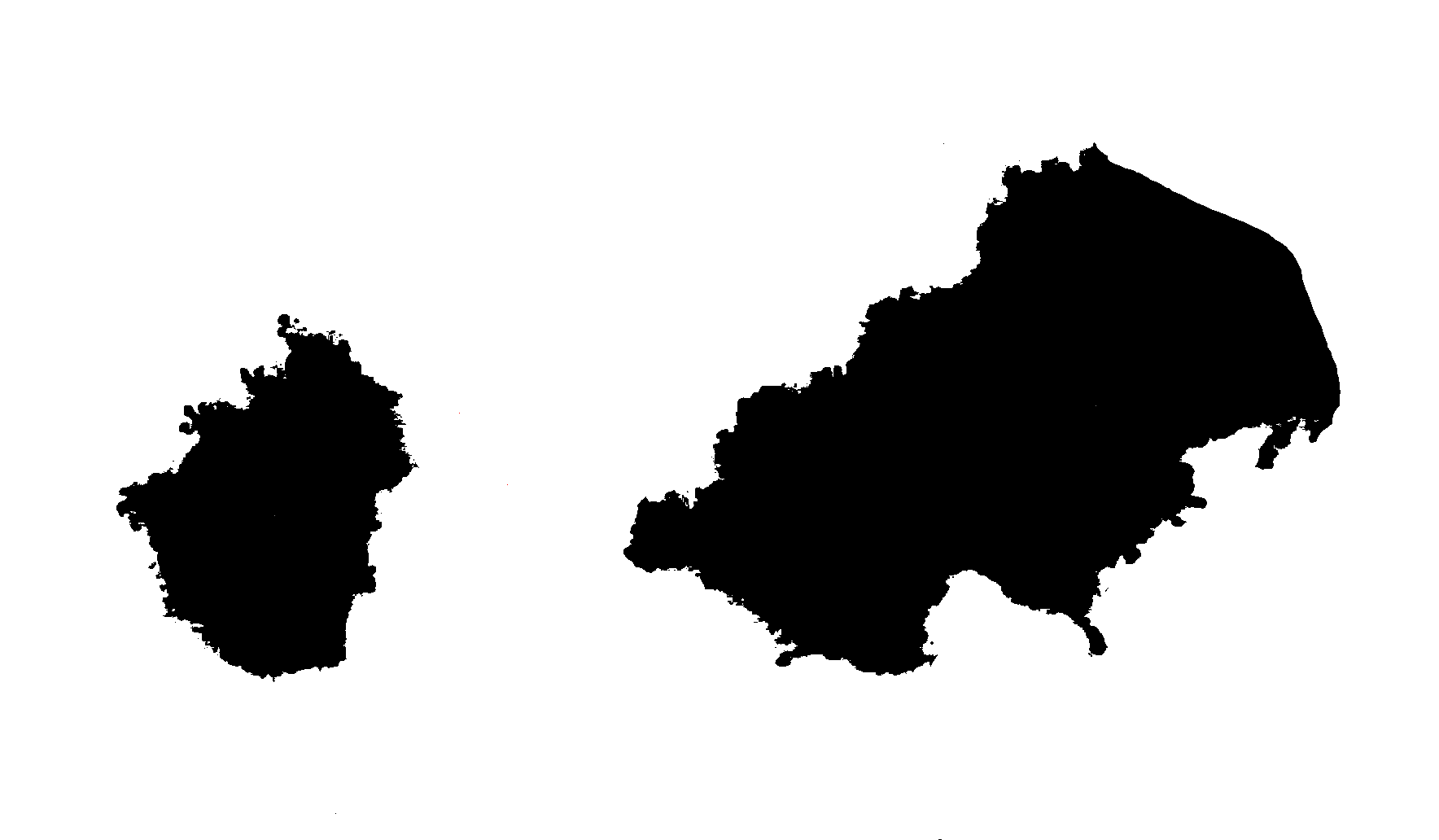

Supplement: Supplementary file 2 [file Datasheet2.zip › figshare/ImageIn/Experiment_009.tif]

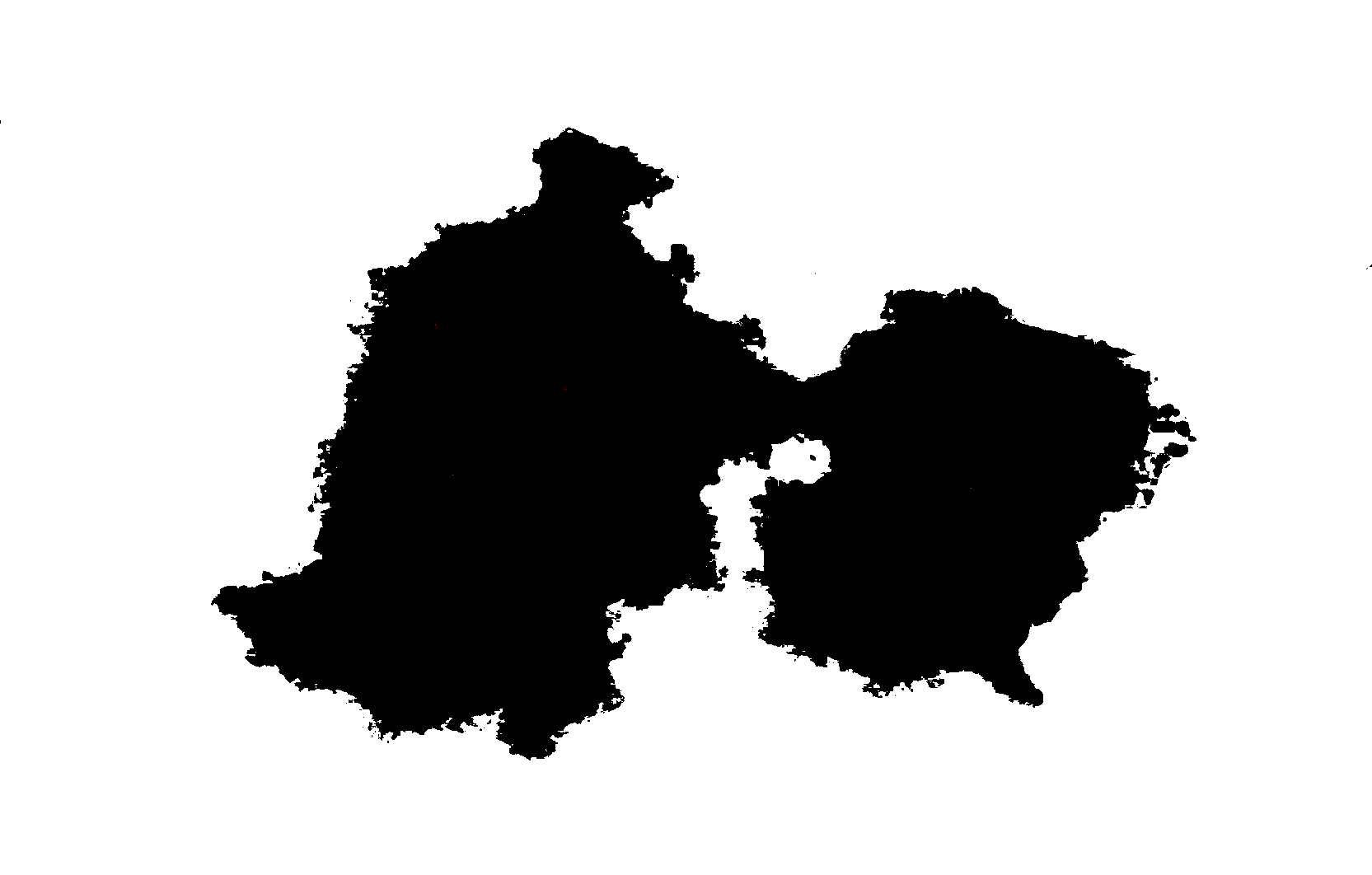

Supplement: Supplementary file 2 [file Datasheet2.zip › figshare/ImageIn/Experiment_010.tif]

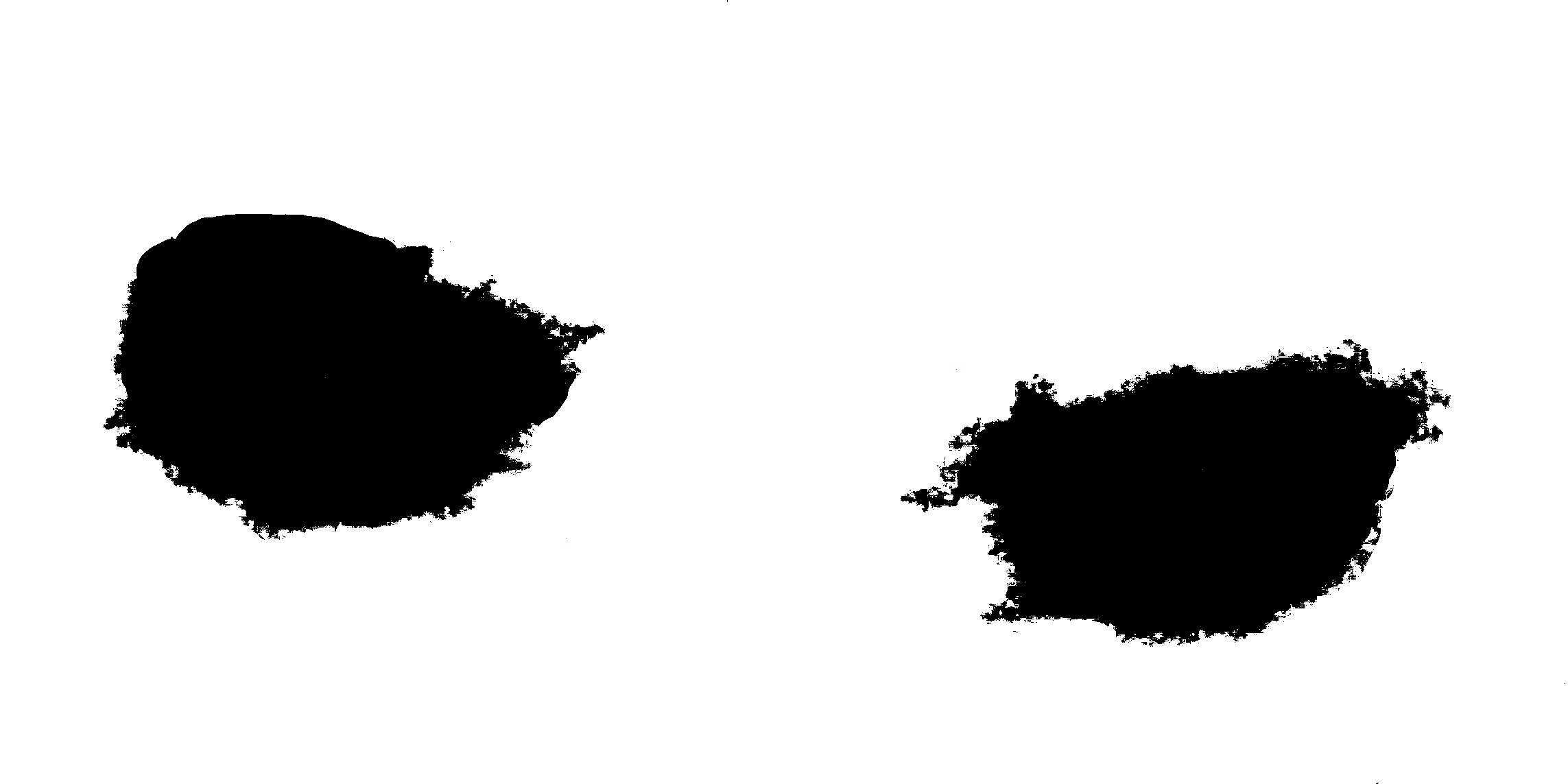

Supplement: Supplementary file 2 [file Datasheet2.zip › figshare/ImageIn/Experiment_011.tif]

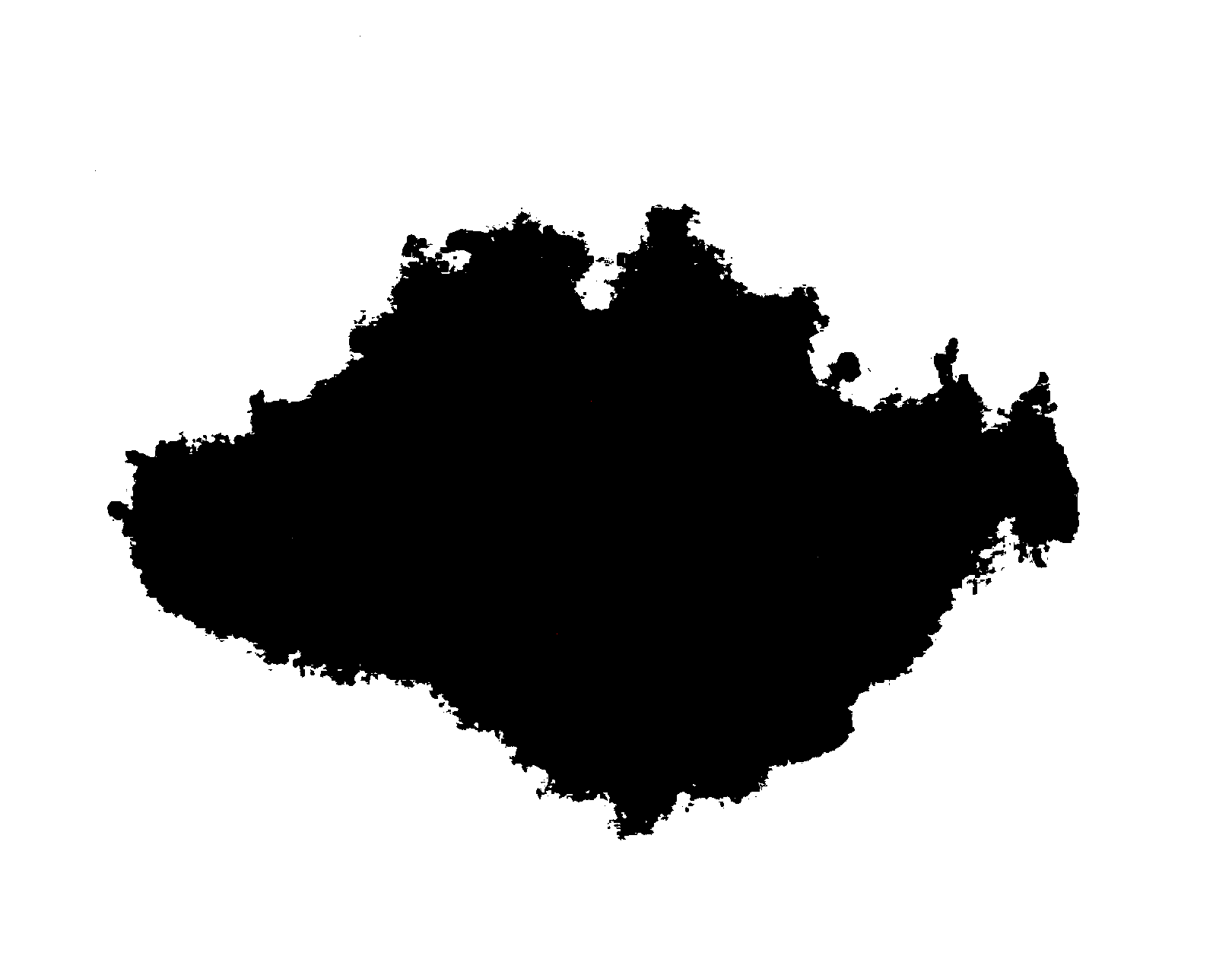

Supplement: Supplementary file 2 [file Datasheet2.zip › figshare/ImageIn/Experiment_012.tif]

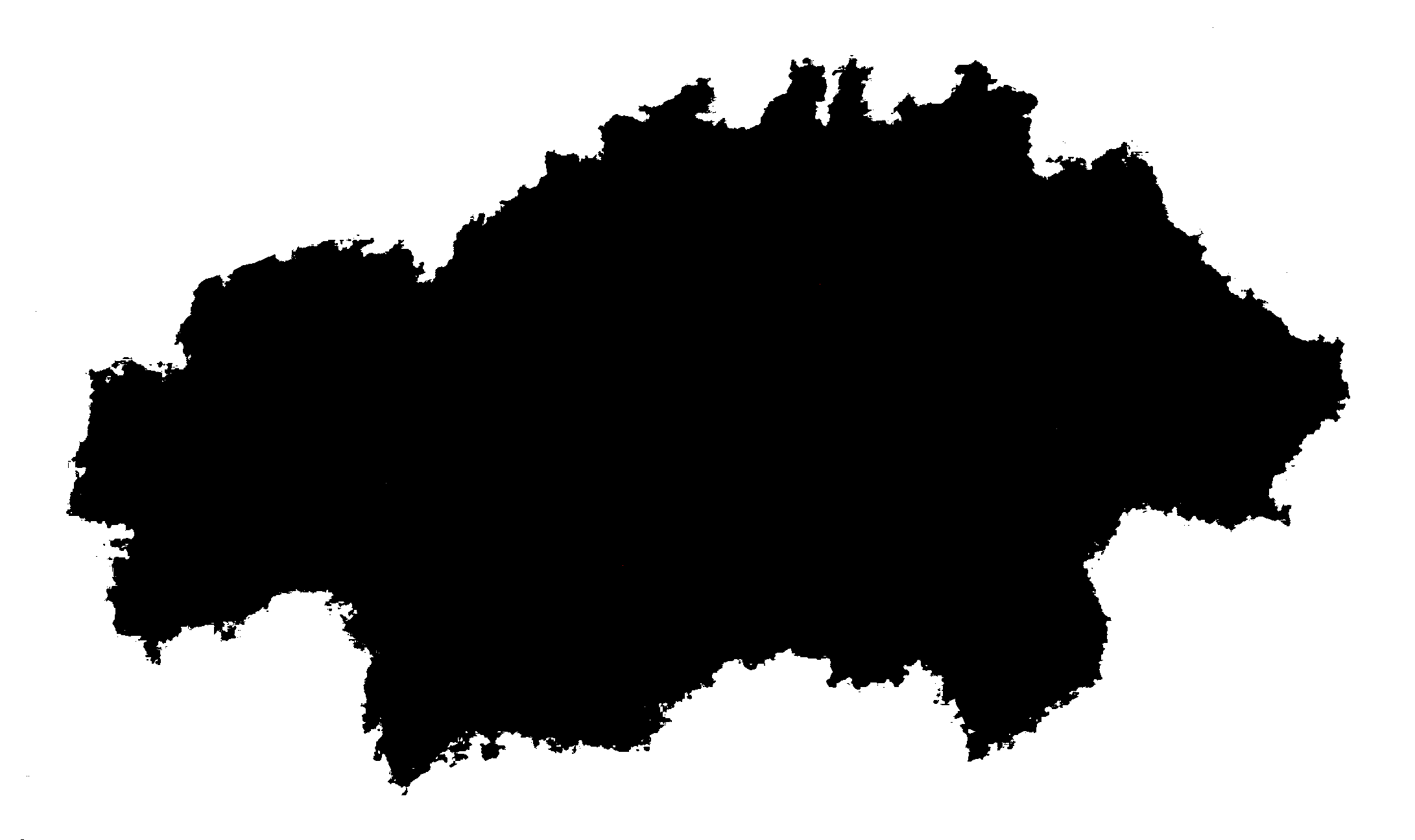

Supplement: Supplementary file 2 [file Datasheet2.zip › figshare/ImageIn/Experiment_013.tif]

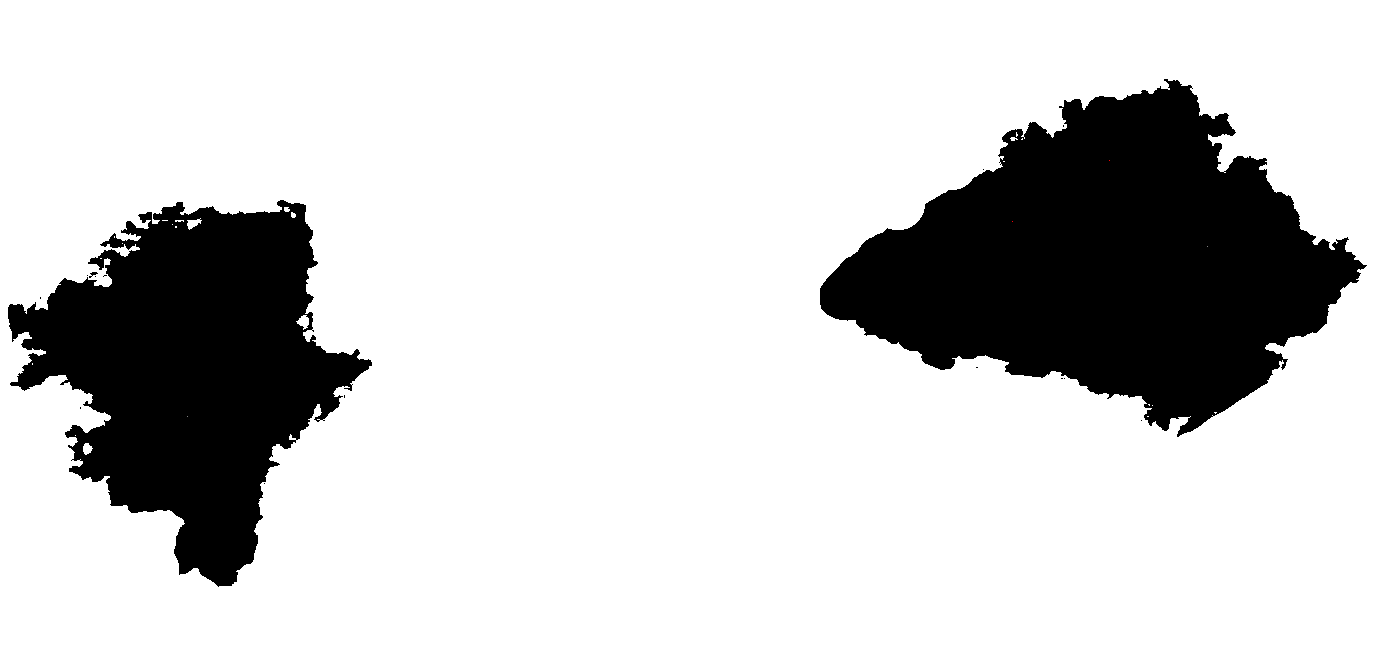

Supplement: Supplementary file 2 [file Datasheet2.zip › figshare/ImageIn/Experiment_014.tif]

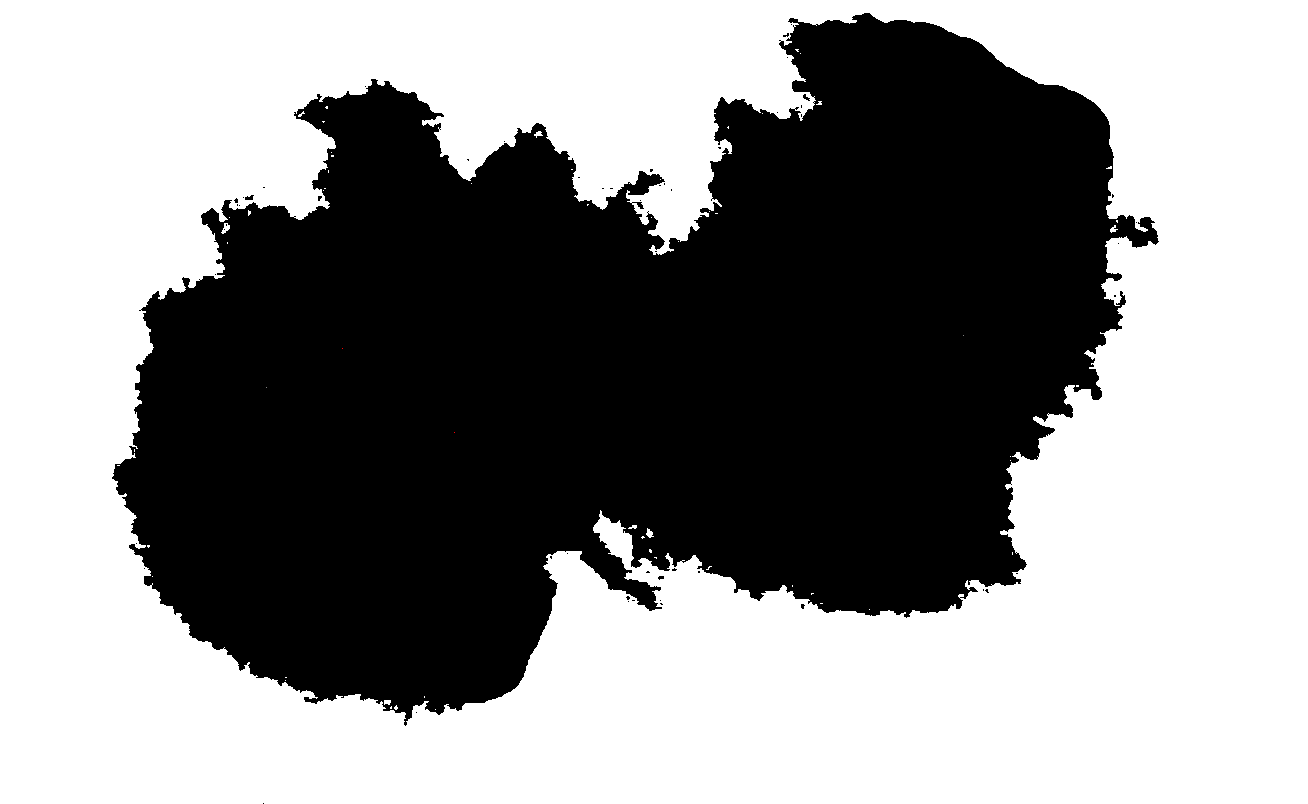

Supplement: Supplementary file 2 [file Datasheet2.zip › figshare/ImageIn/Experiment_015.tif]

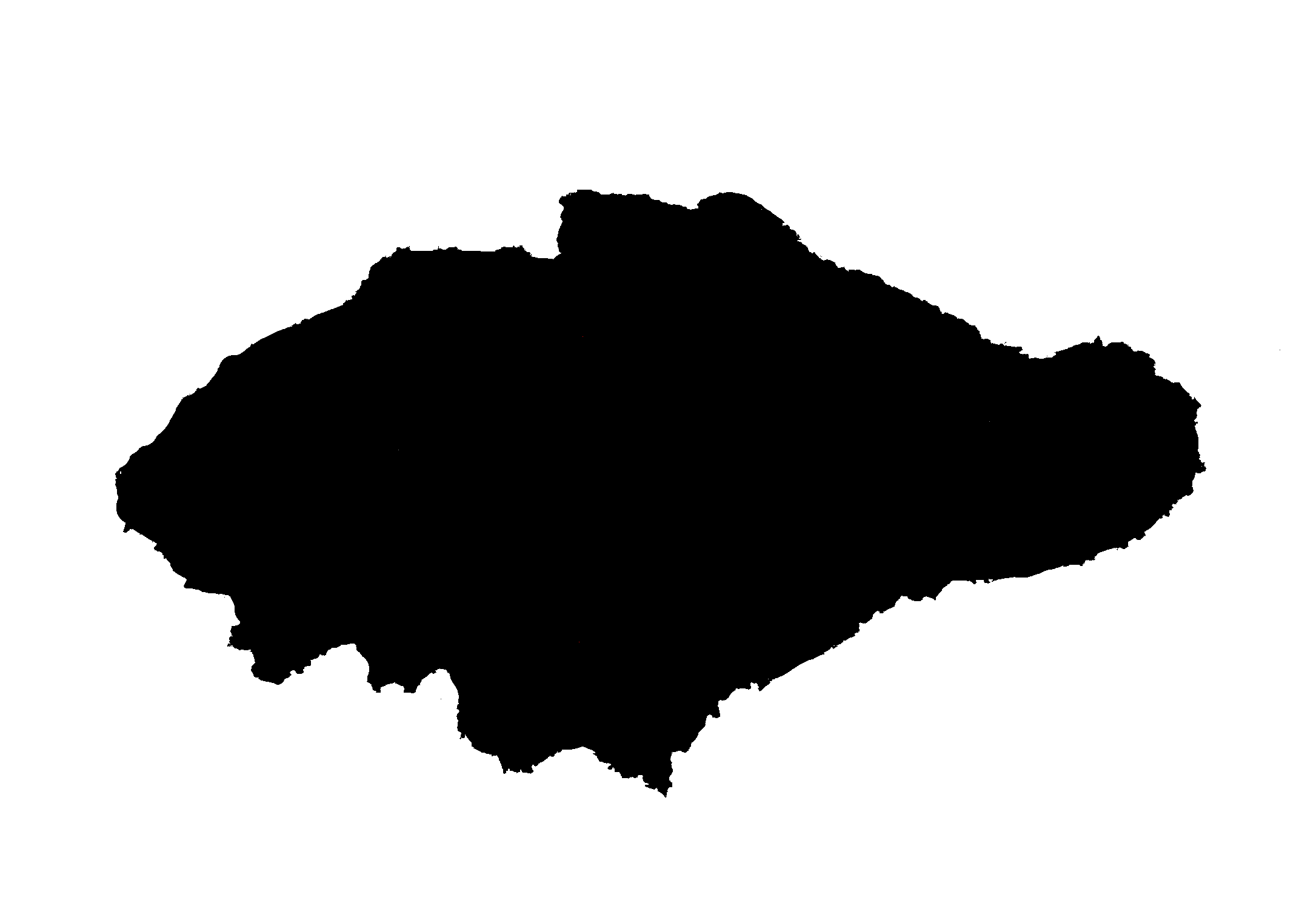

Supplement: Supplementary file 2 [file Datasheet2.zip › figshare/ImageIn/Experiment_016.tif]

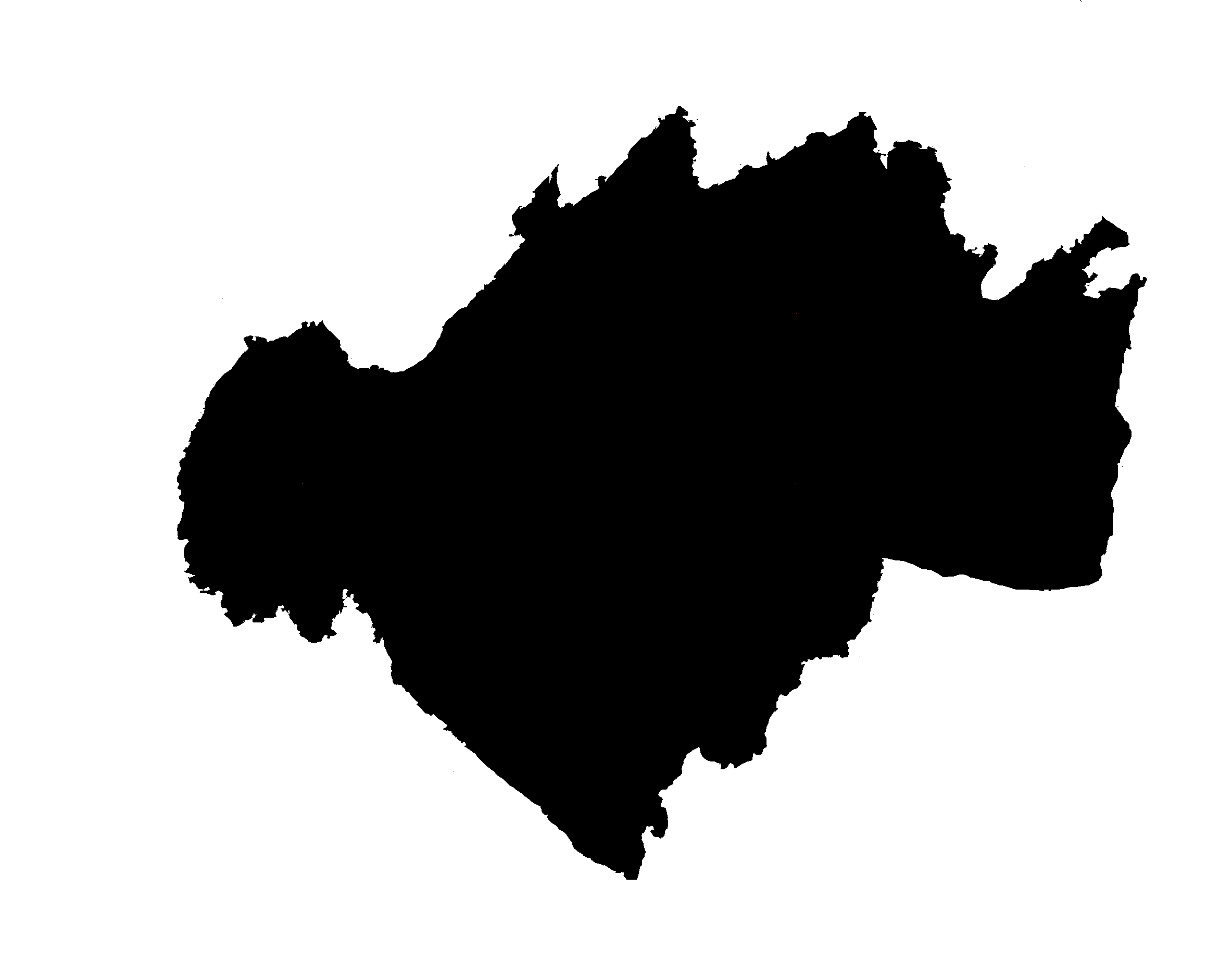

Supplement: Supplementary file 2 [file Datasheet2.zip › figshare/ImageIn/Experiment_017.tif]

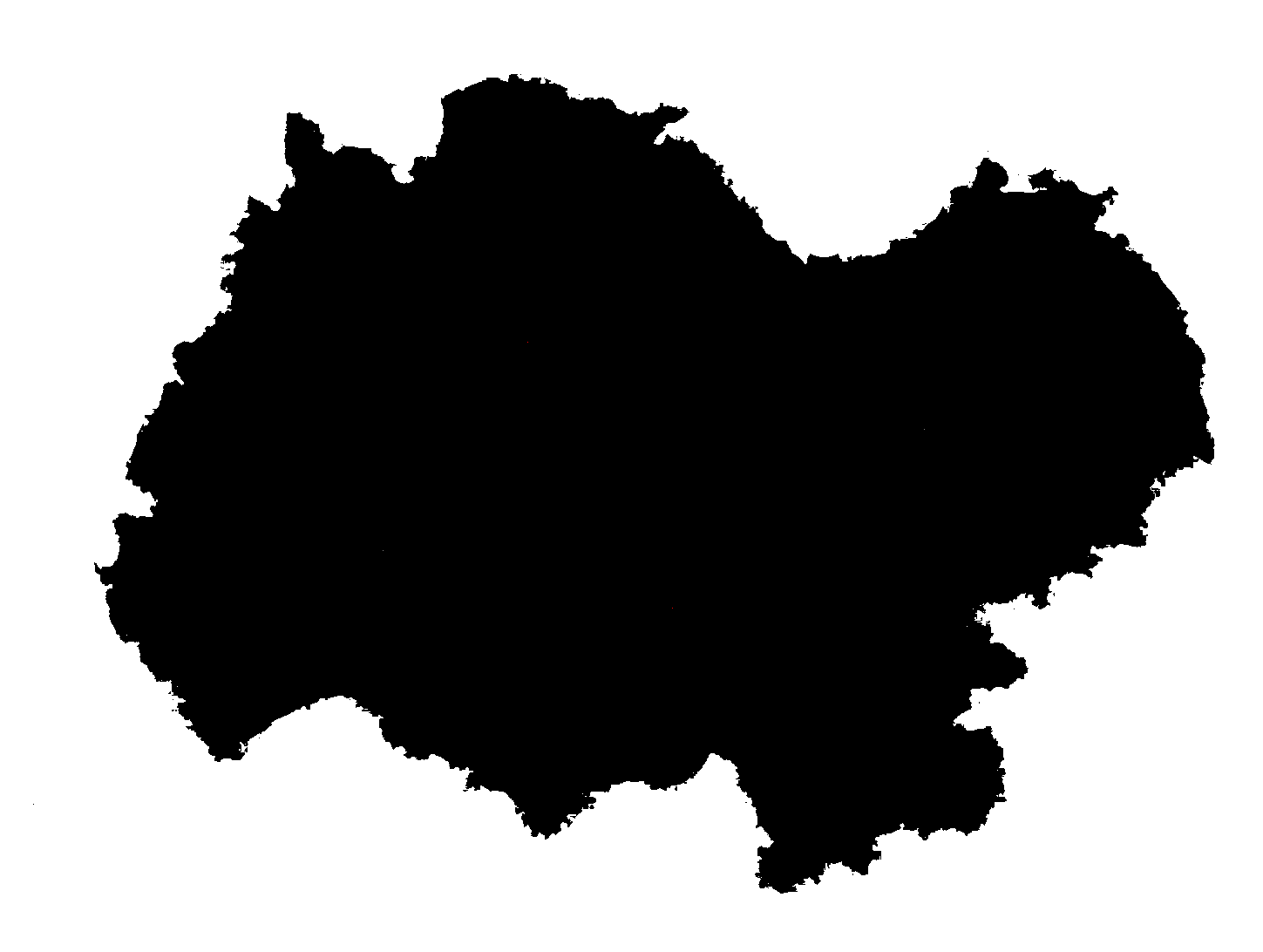

Supplement: Supplementary file 2 [file Datasheet2.zip › figshare/ImageIn/Experiment_018.tif]

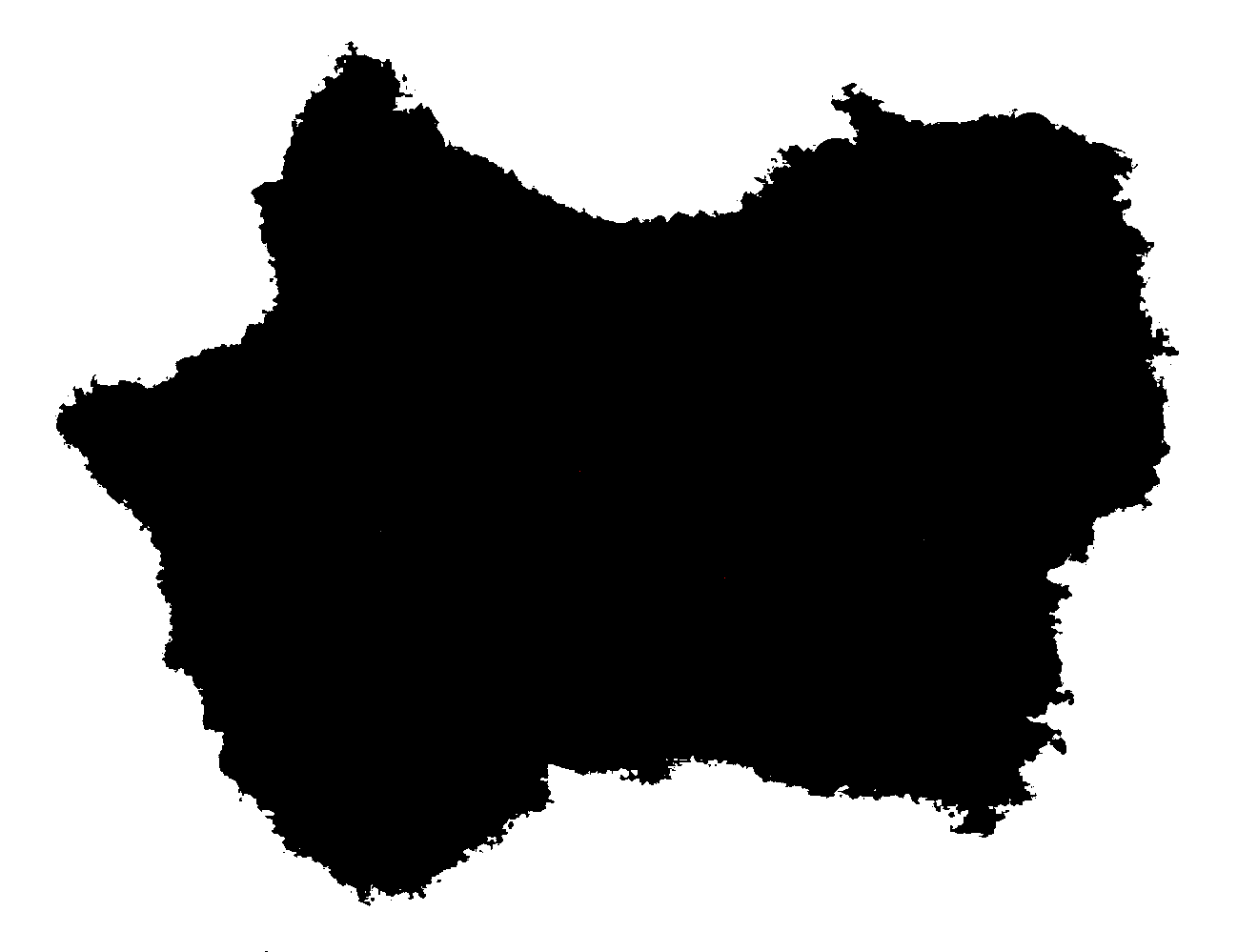

Supplement: Supplementary file 2 [file Datasheet2.zip › figshare/ImageIn/Experiment_019.tif]

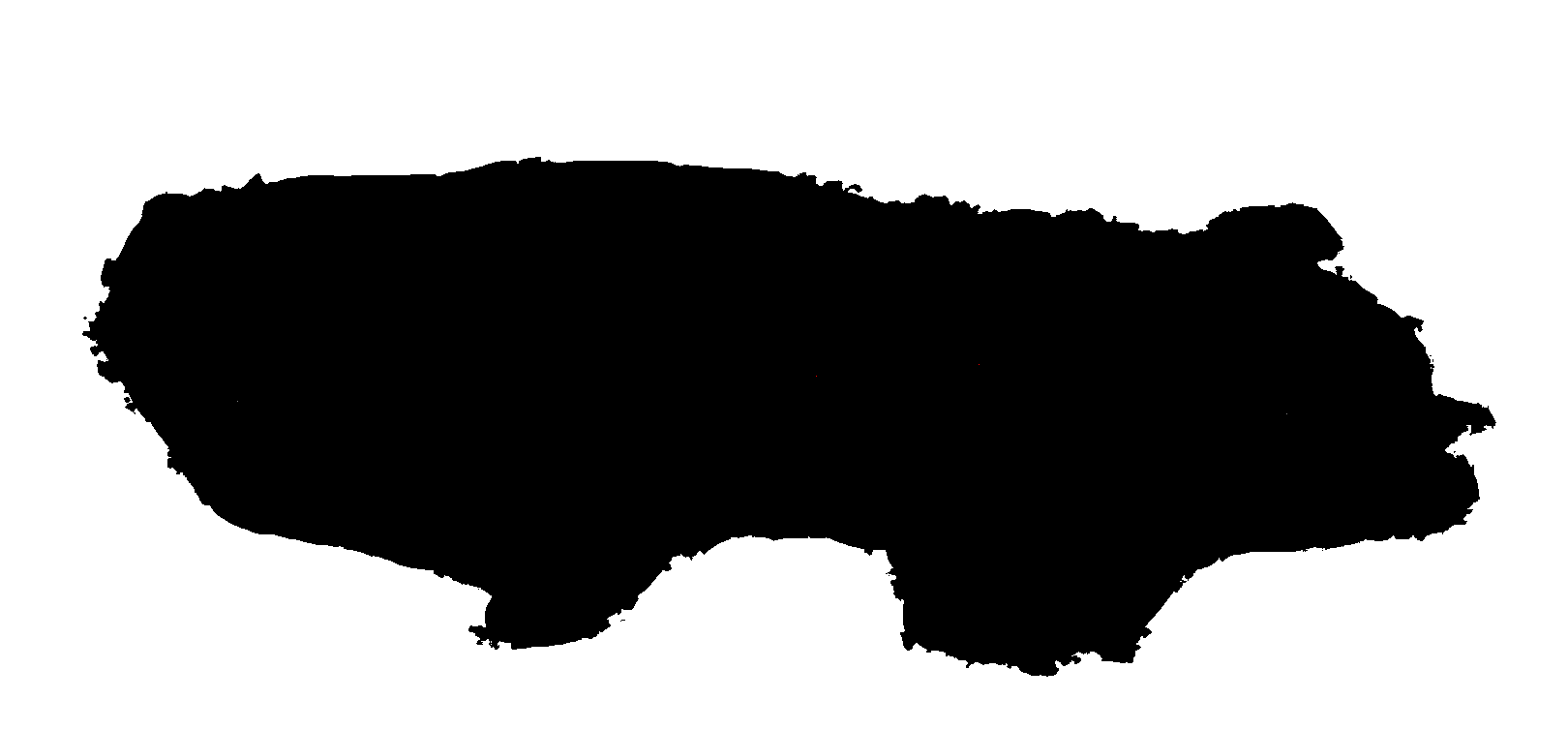

Supplement: Supplementary file 2 [file Datasheet2.zip › figshare/ImageIn/Experiment_020.tif]

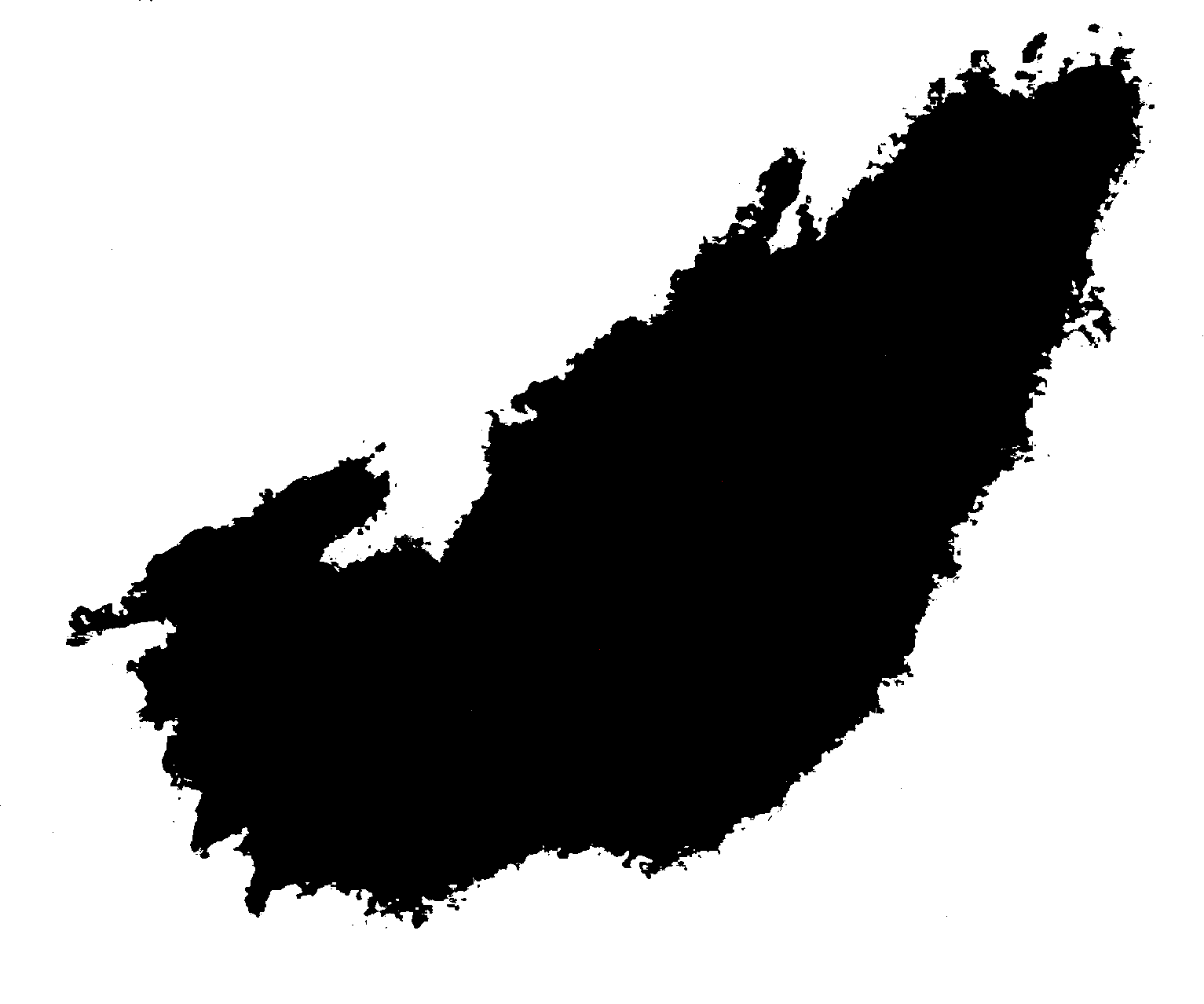

Supplement: Supplementary file 2 [file Datasheet2.zip › figshare/ImageIn/Experiment_021.tif]

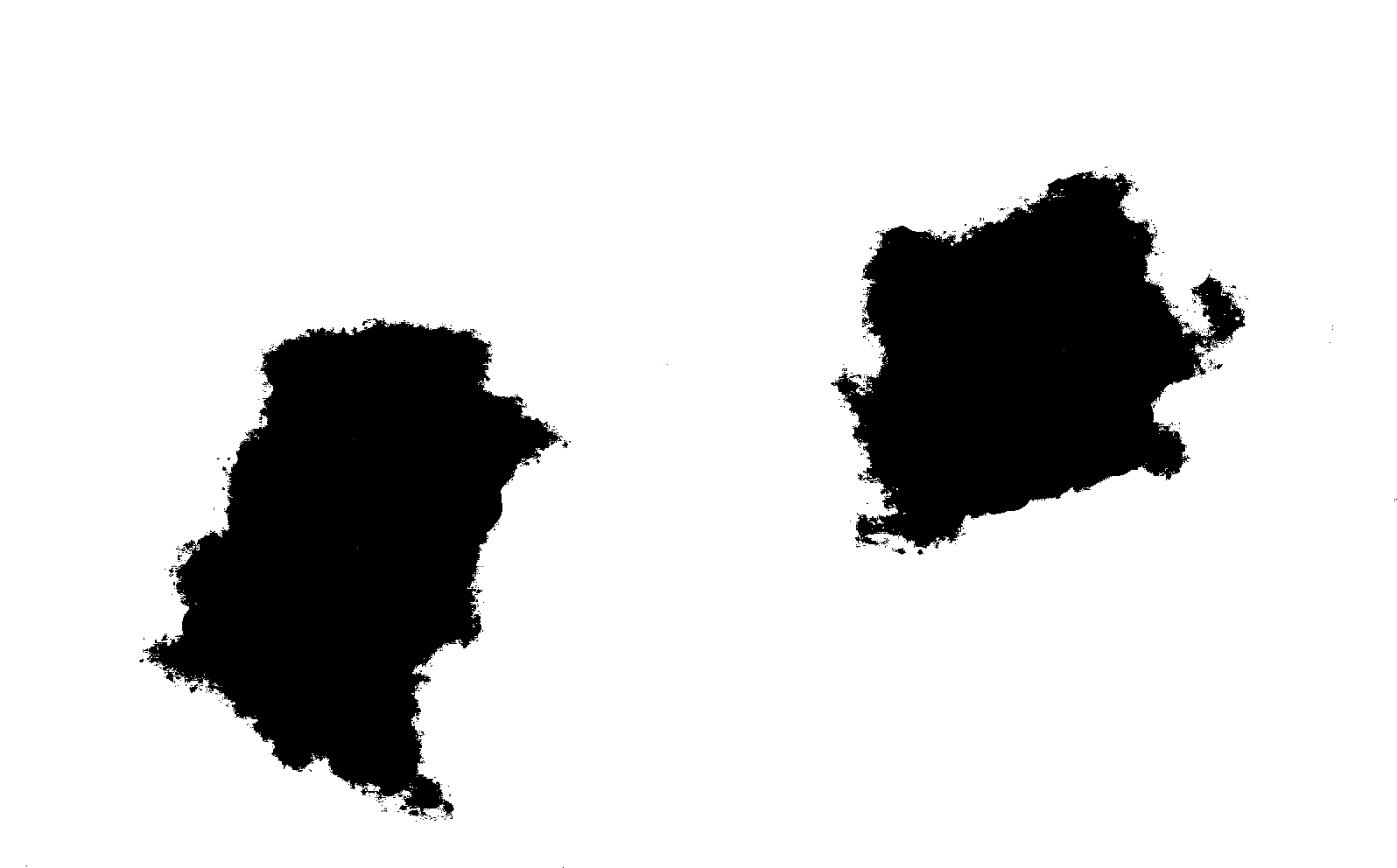

Supplement: Supplementary file 2 [file Datasheet2.zip › figshare/ImageIn/Experiment_022.tif]

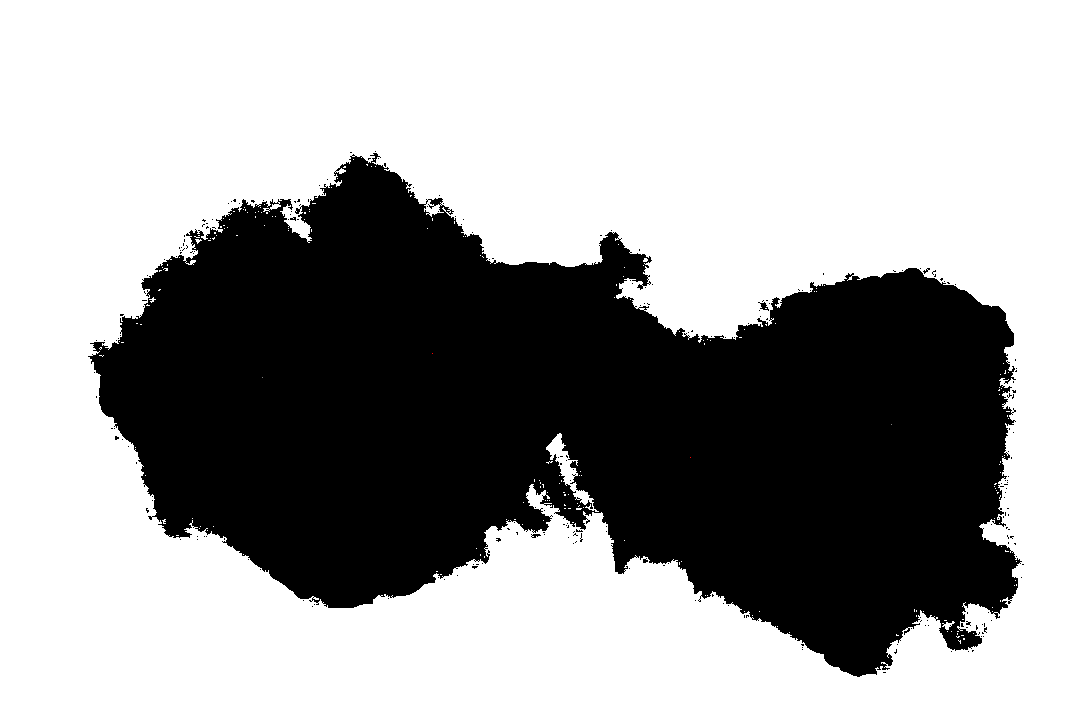

Supplement: Supplementary file 2 [file Datasheet2.zip › figshare/ImageIn/Experiment_023.tif]

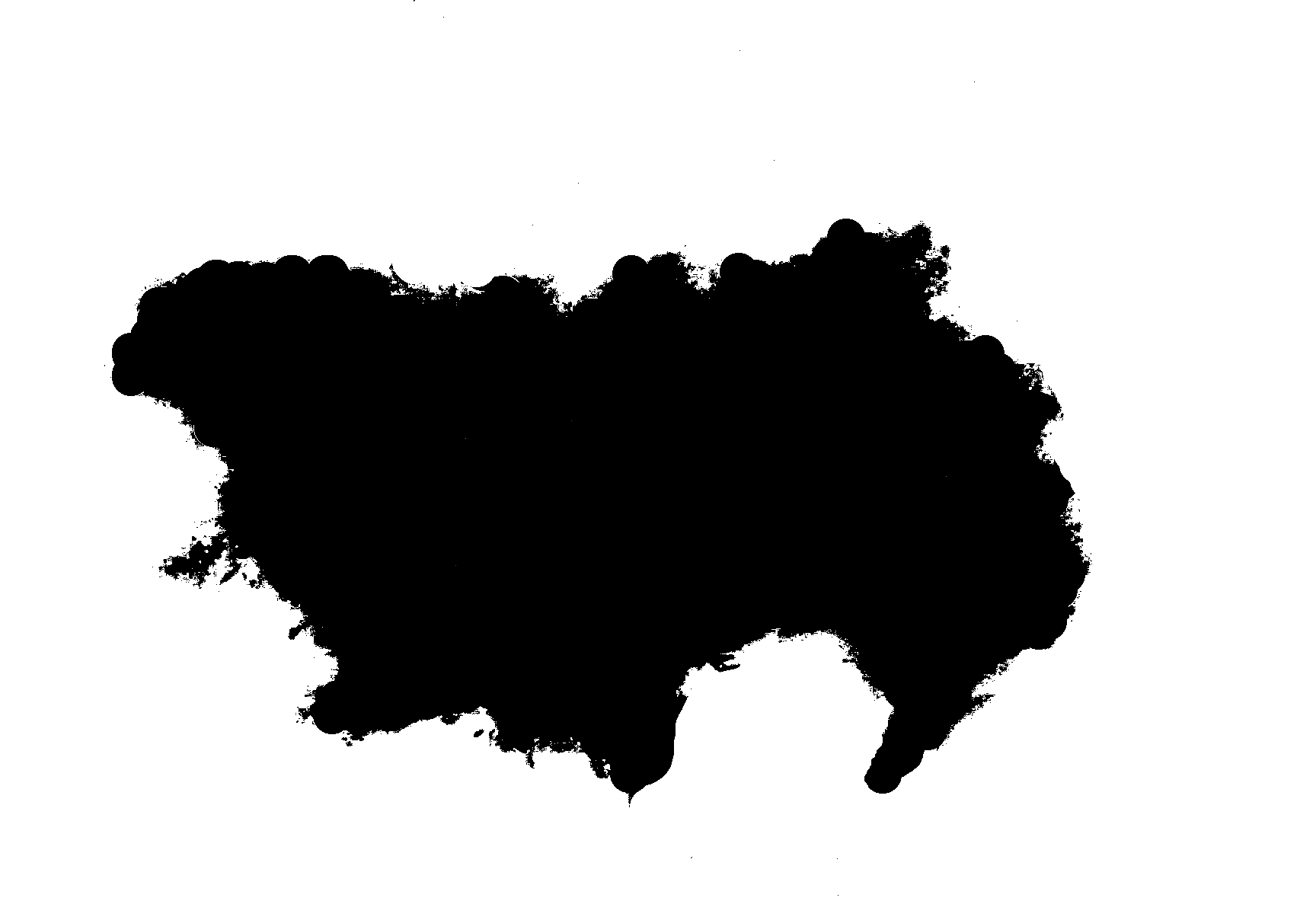

Supplement: Supplementary file 2 [file Datasheet2.zip › figshare/ImageIn/Experiment_024.tif]

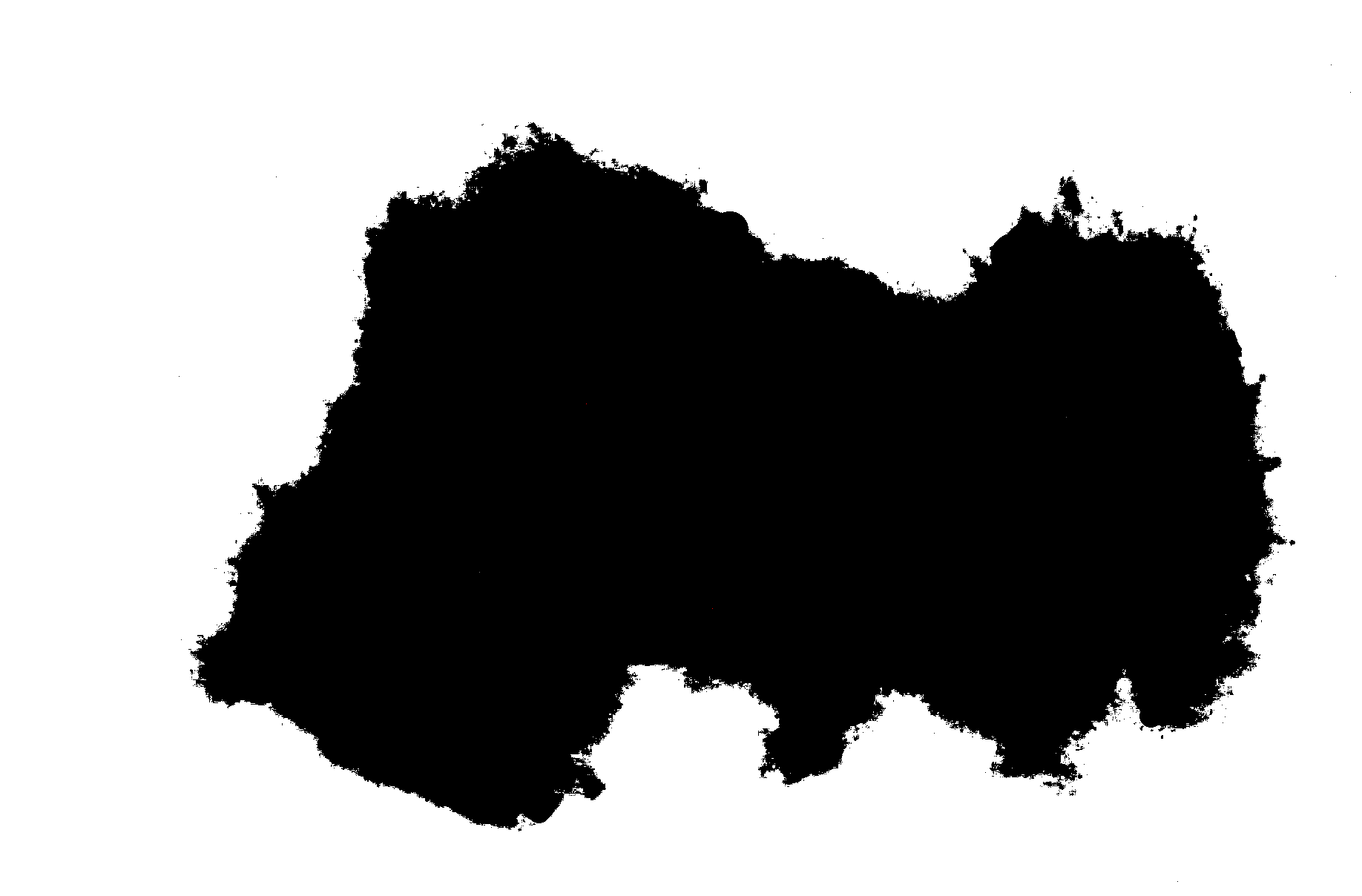

Supplement: Supplementary file 2 [file Datasheet2.zip › figshare/ImageIn/Experiment_025.tif]

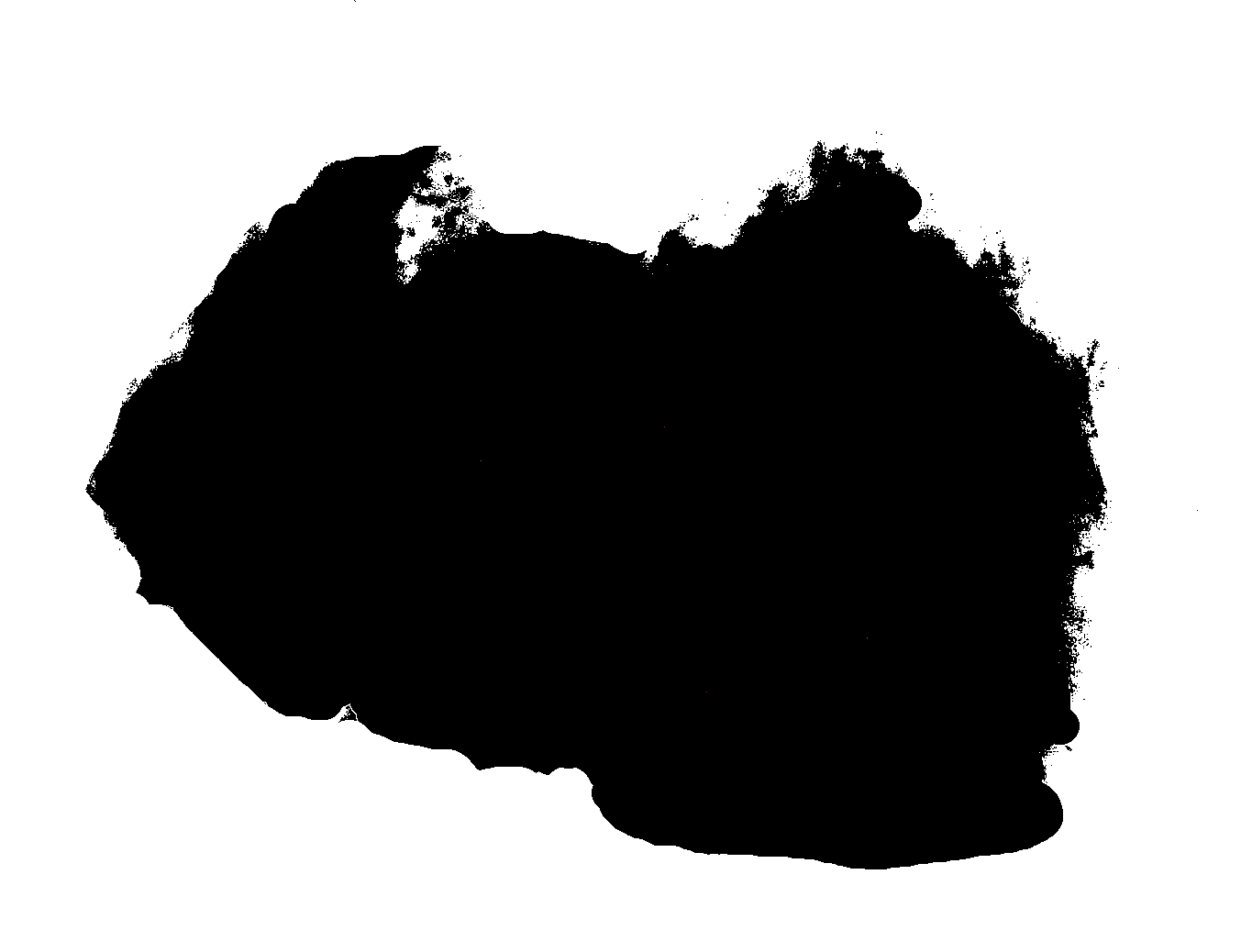

Supplement: Supplementary file 2 [file Datasheet2.zip › figshare/ImageIn/Experiment_026.tif]

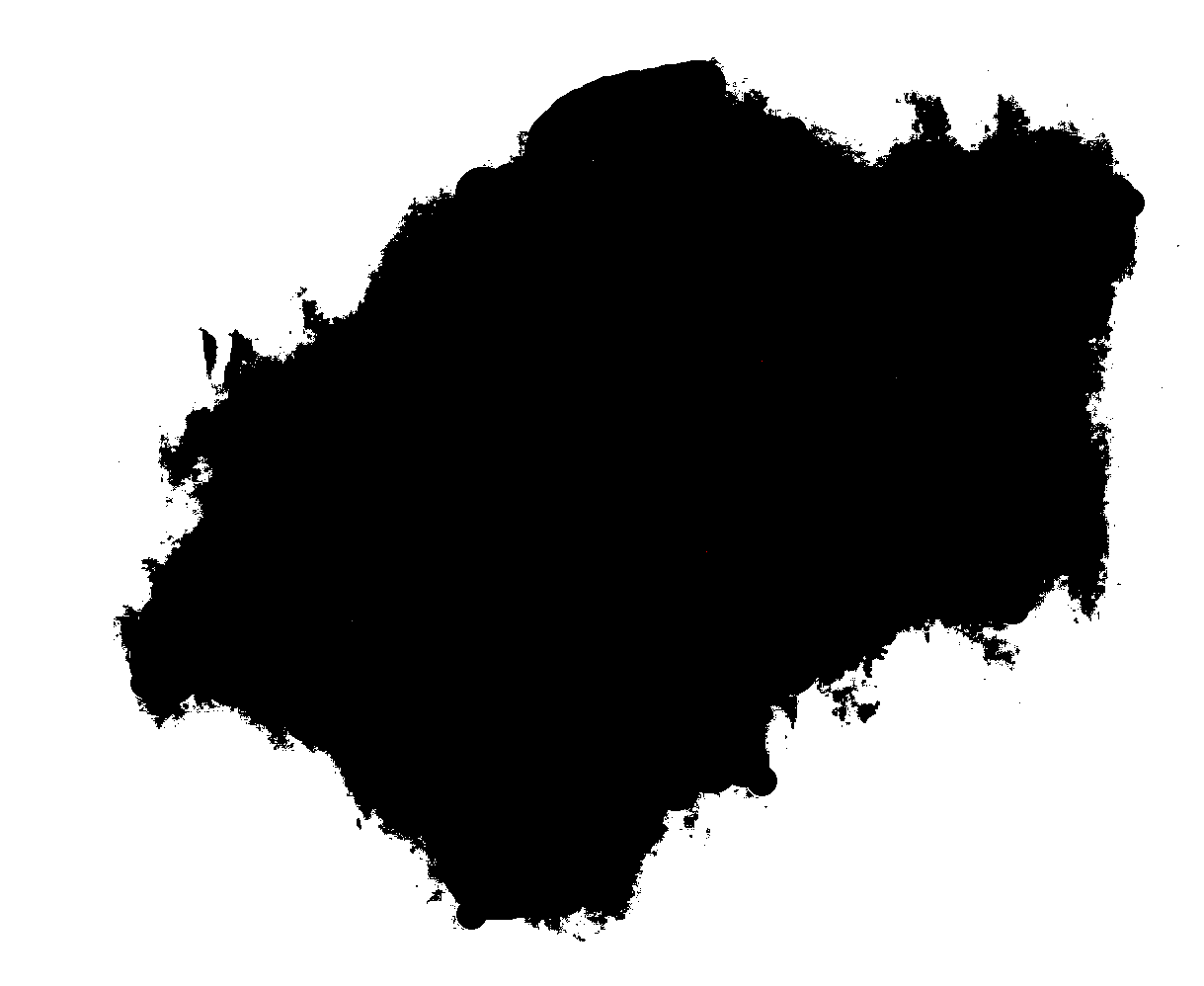

Supplement: Supplementary file 2 [file Datasheet2.zip › figshare/ImageIn/Experiment_027.tif]

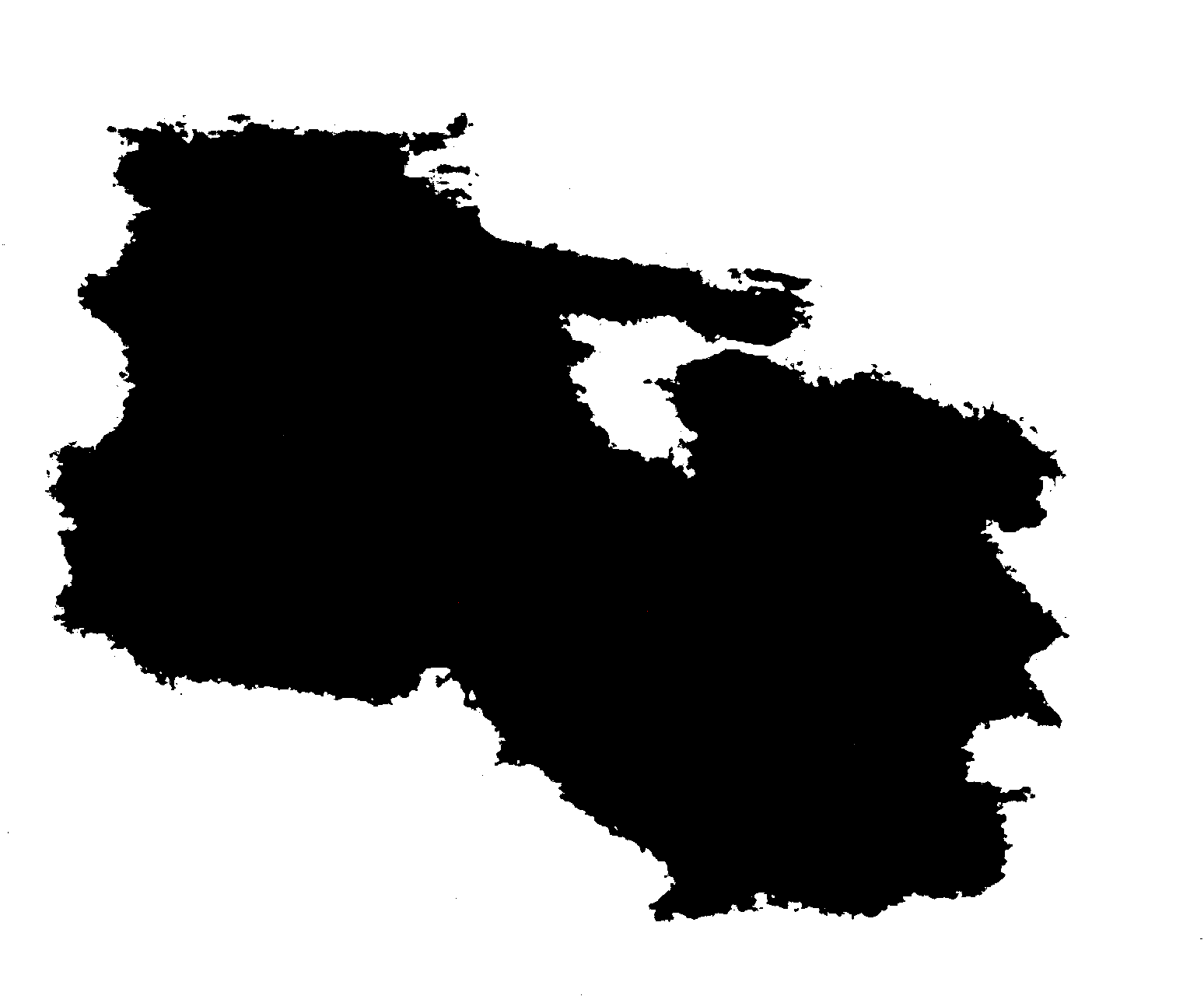

Supplement: Supplementary file 2 [file Datasheet2.zip › figshare/ImageIn/Experiment_028.tif]

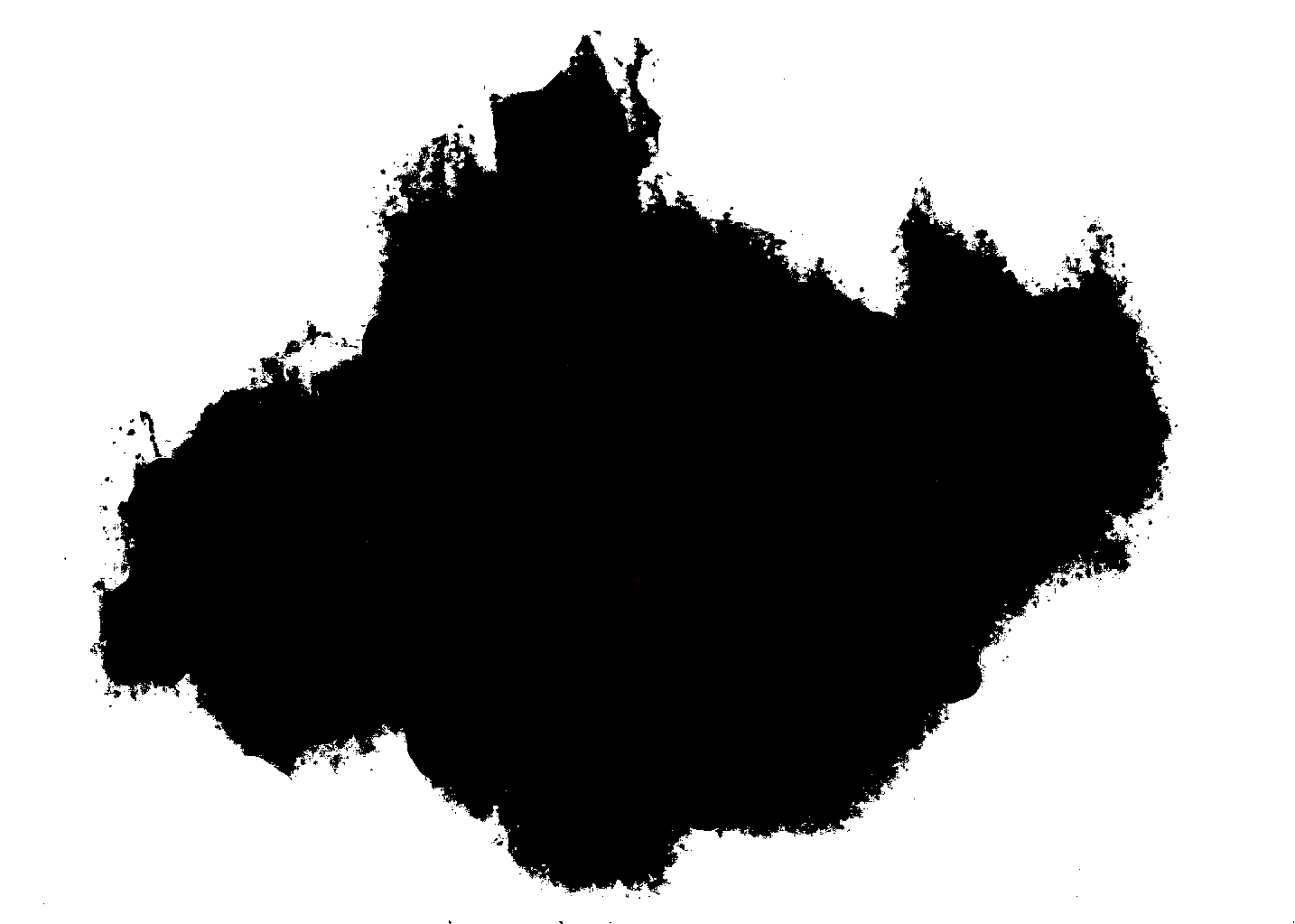

Supplement: Supplementary file 2 [file Datasheet2.zip › figshare/ImageIn/Experiment_029.tif]

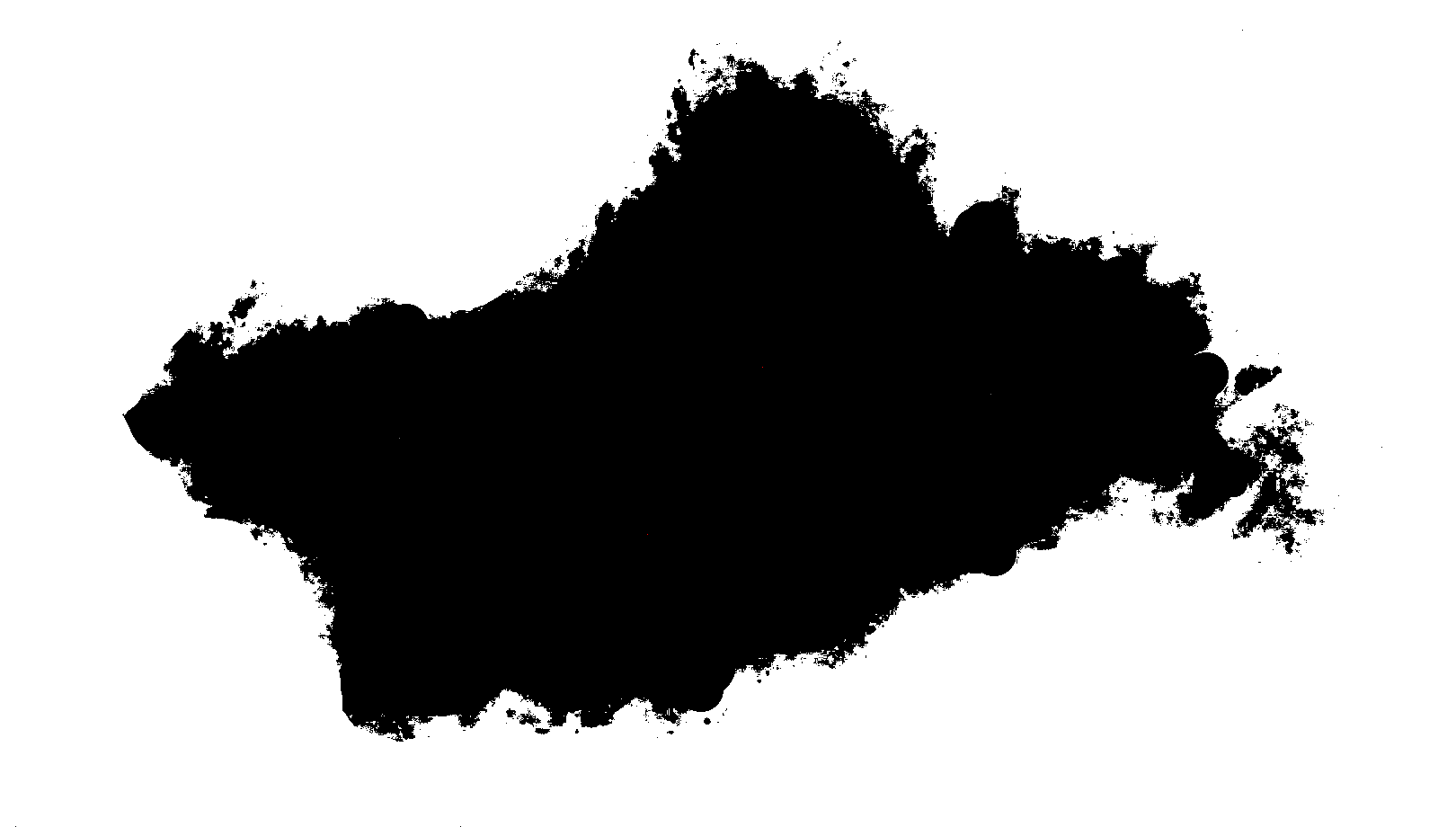

Supplement: Supplementary file 2 [file Datasheet2.zip › figshare/ImageIn/Experiment_030.tif]

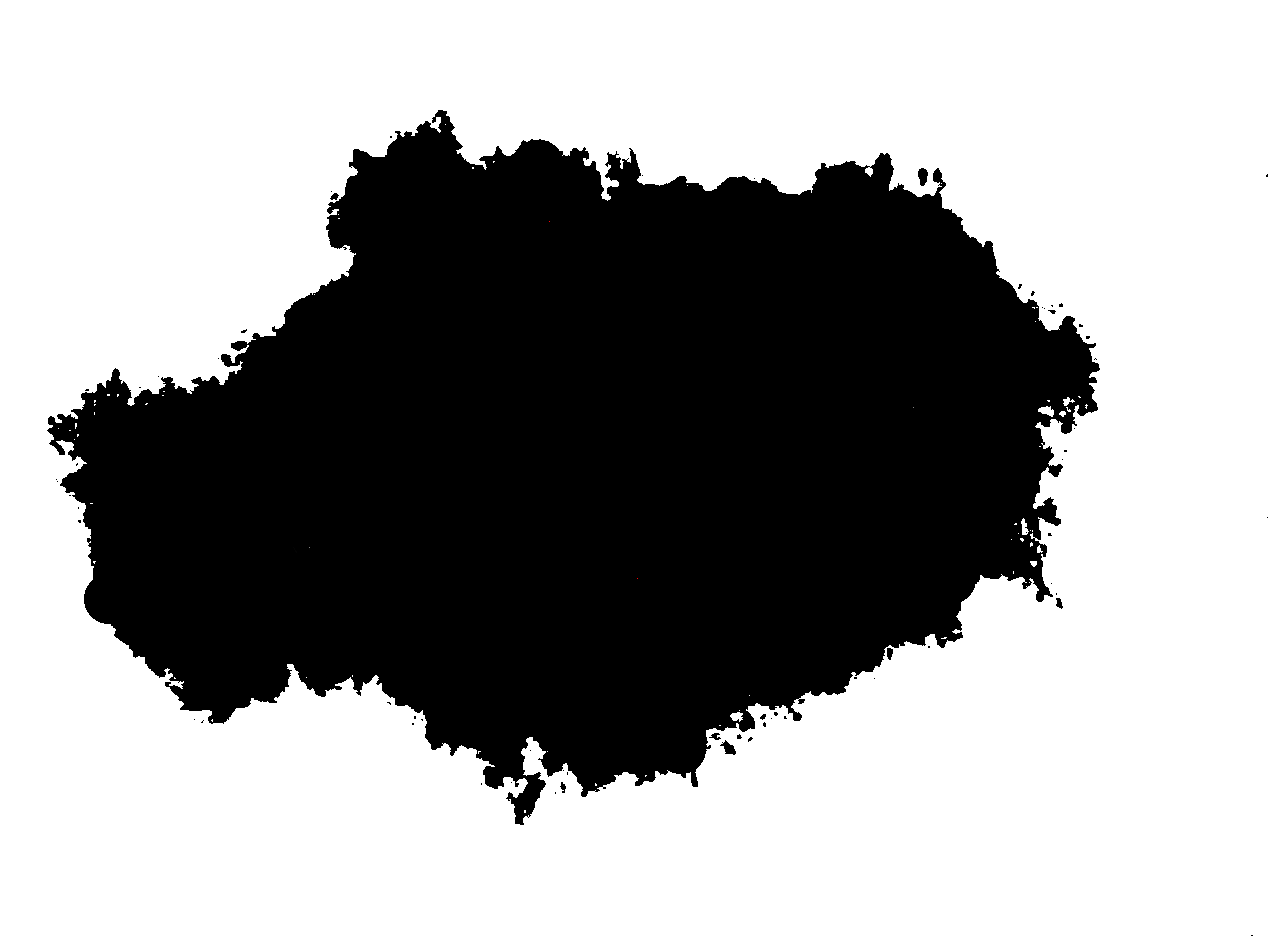

Supplement: Supplementary file 2 [file Datasheet2.zip › figshare/ImageIn/Experiment_031.tif]

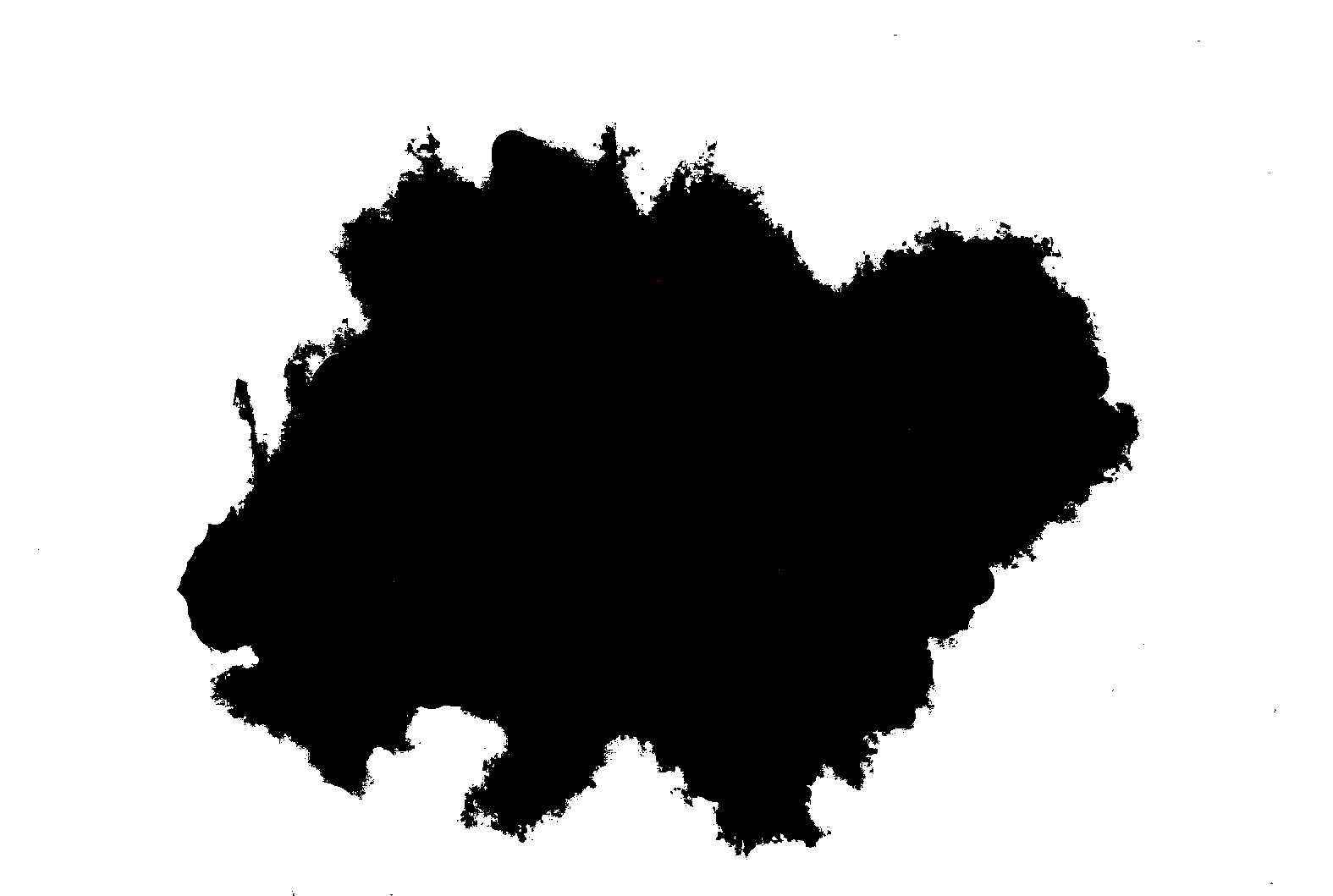

Supplement: Supplementary file 2 [file Datasheet2.zip › figshare/ImageIn/Experiment_032.tif]

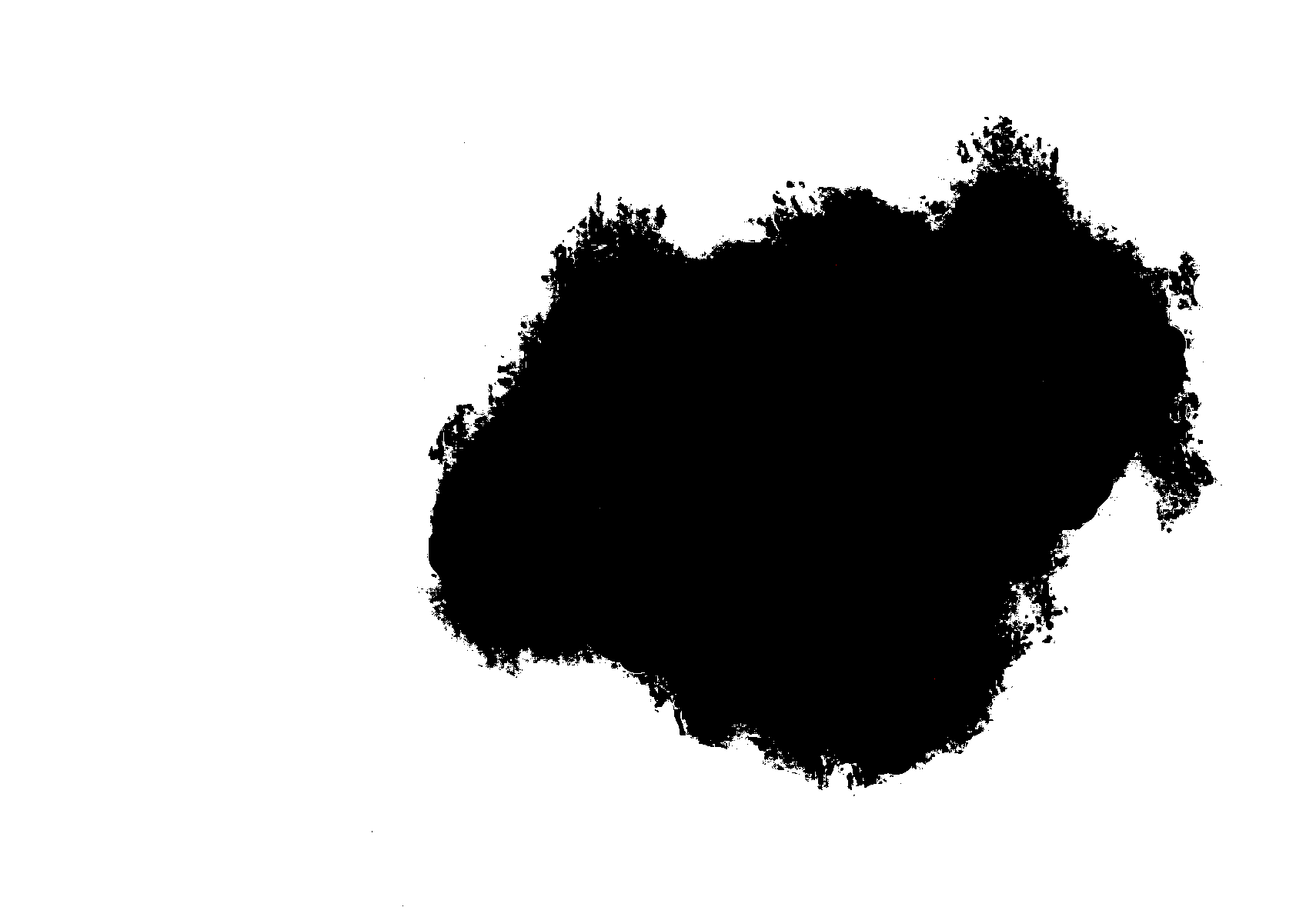

Supplement: Supplementary file 2 [file Datasheet2.zip › figshare/ImageIn/Experiment_033.tif]

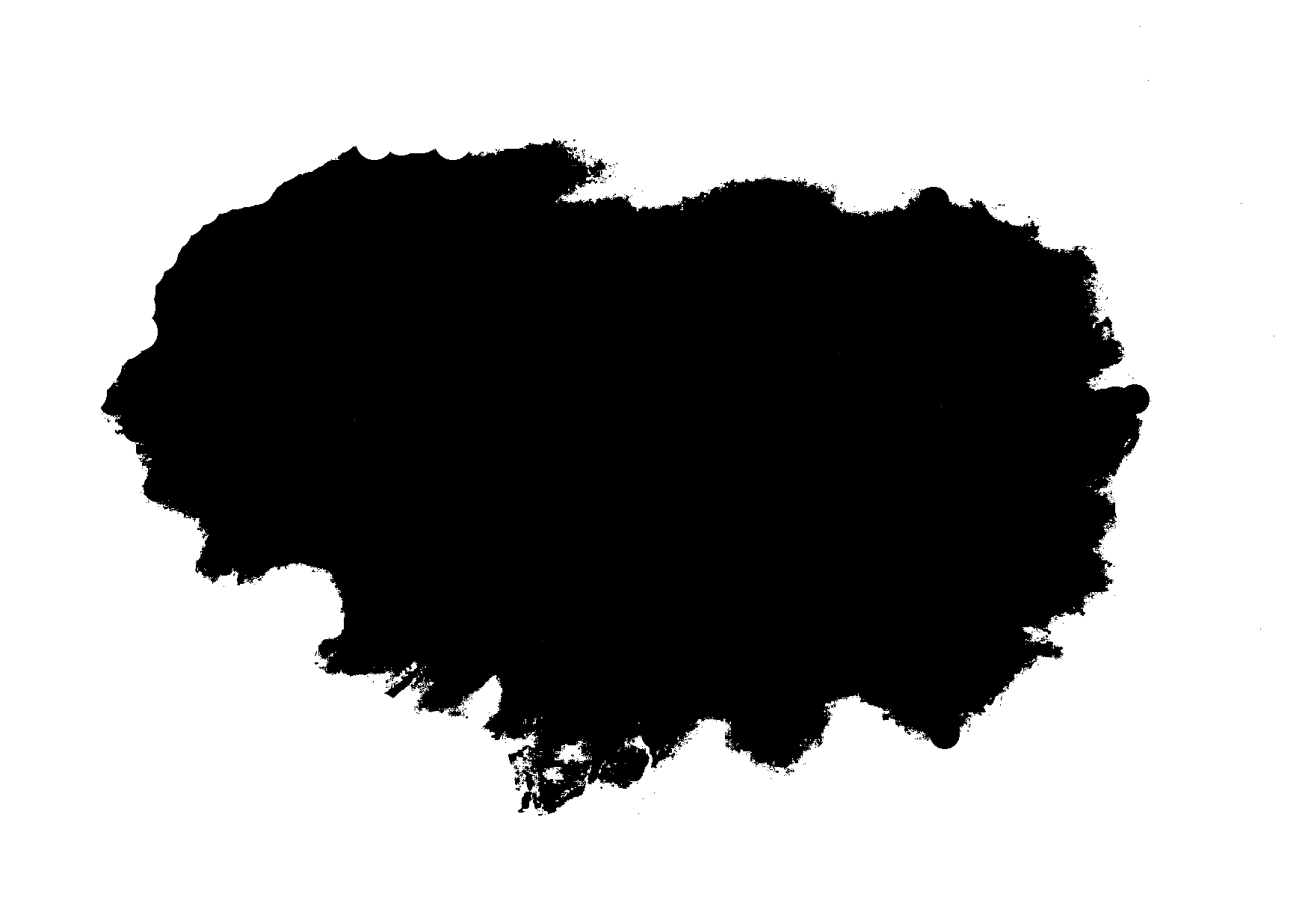

Supplement: Supplementary file 2 [file Datasheet2.zip › figshare/ImageIn/Experiment_034.tif]

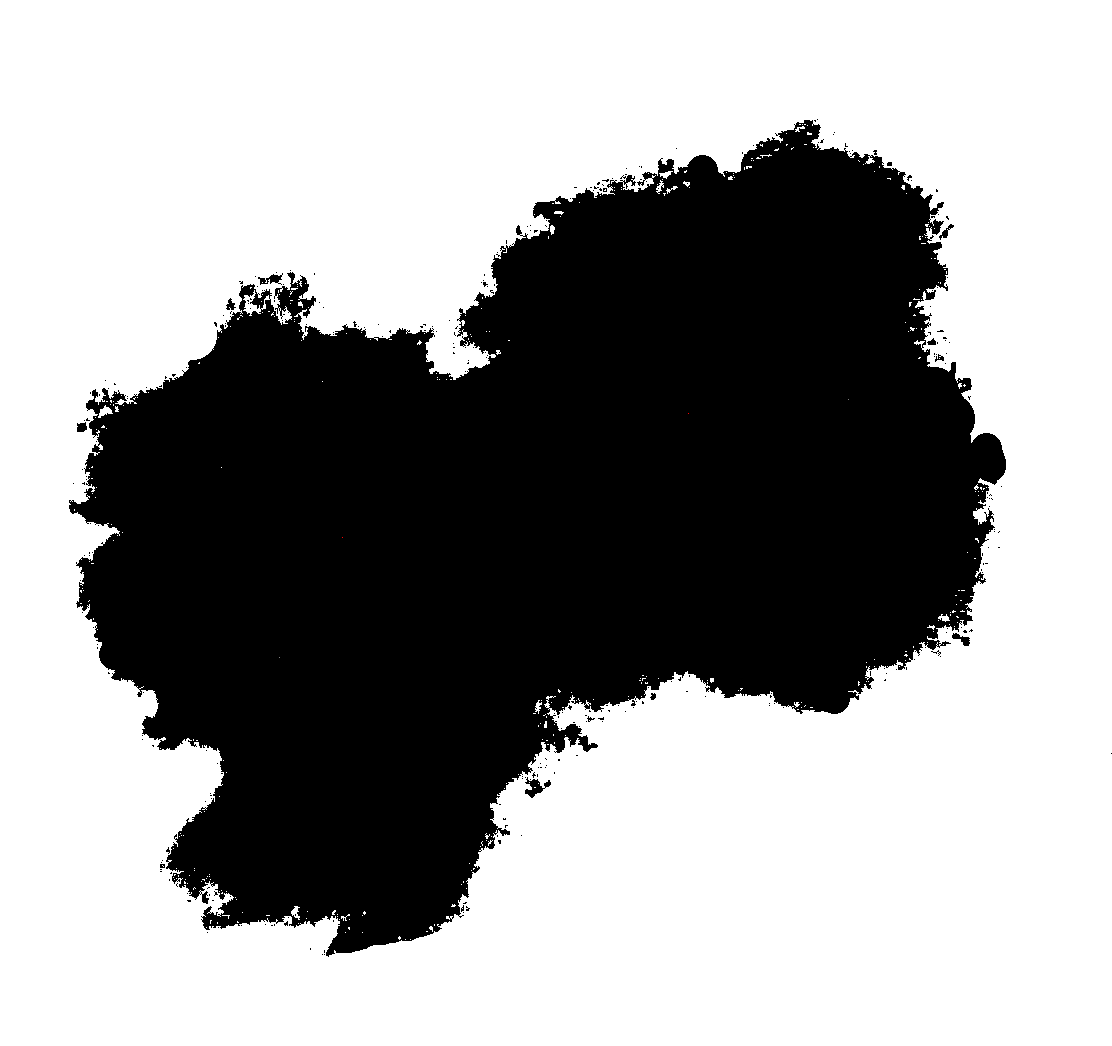

Supplement: Supplementary file 2 [file Datasheet2.zip › figshare/ImageIn/Experiment_035.tif]

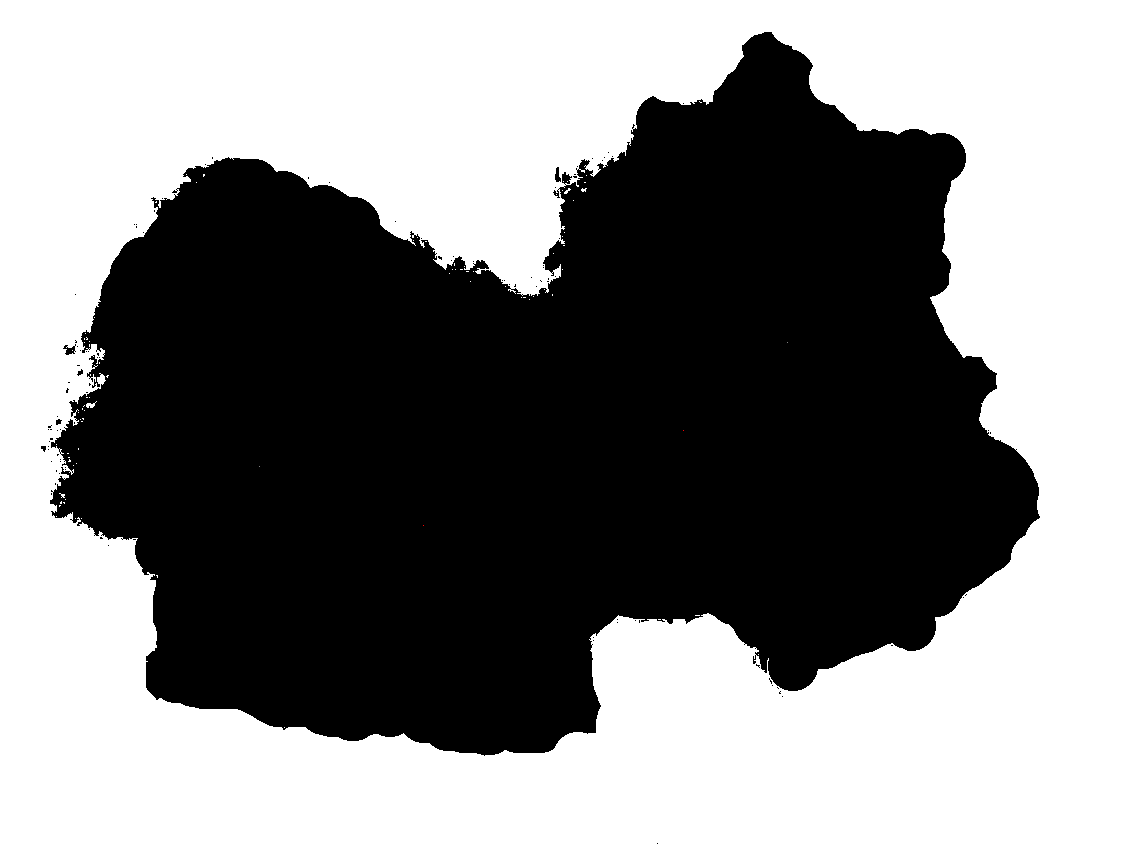

Supplement: Supplementary file 2 [file Datasheet2.zip › figshare/ImageIn/Experiment_036.tif]

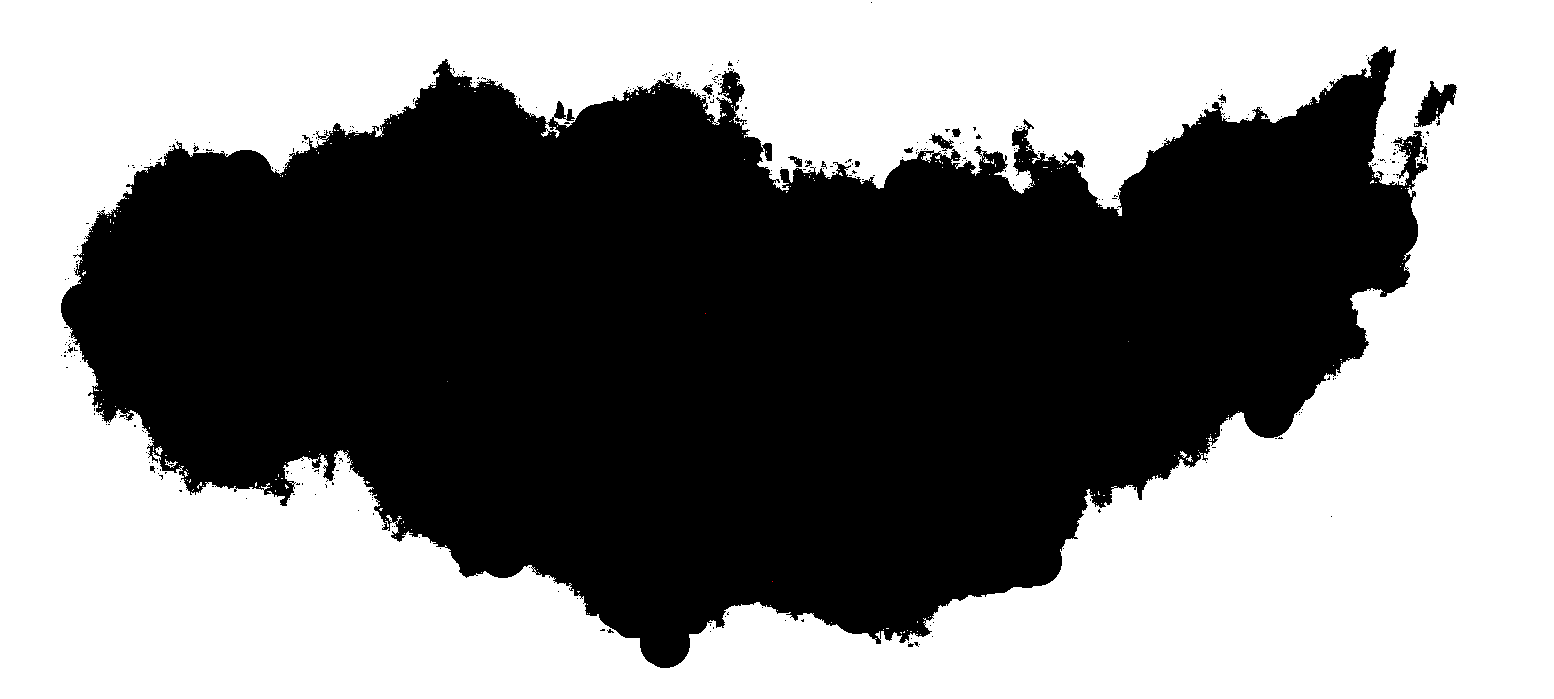

Supplement: Supplementary file 2 [file Datasheet2.zip › figshare/ImageIn/Experiment_037.tif]

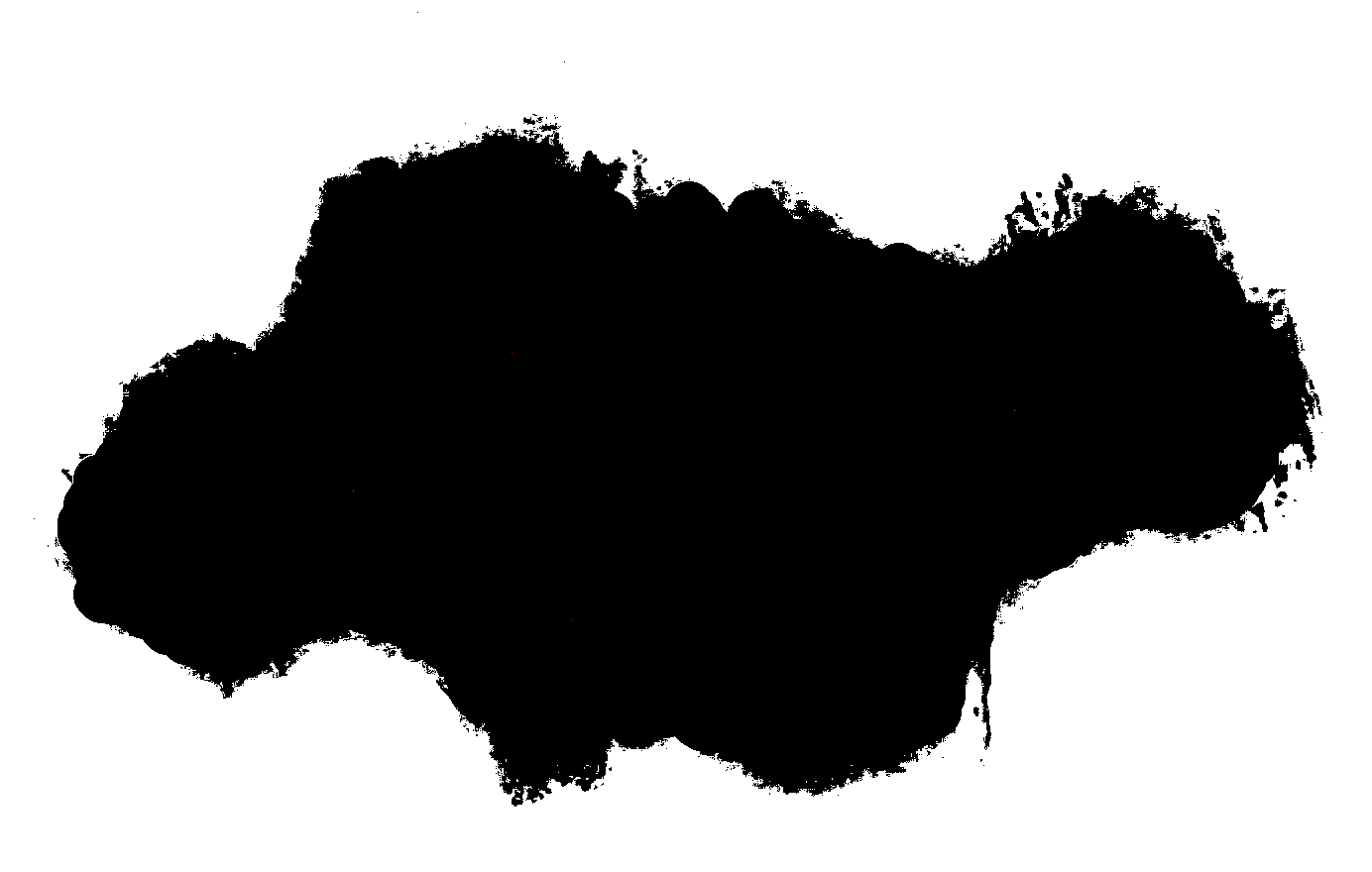

Supplement: Supplementary file 2 [file Datasheet2.zip › figshare/ImageIn/Experiment_038.tif]

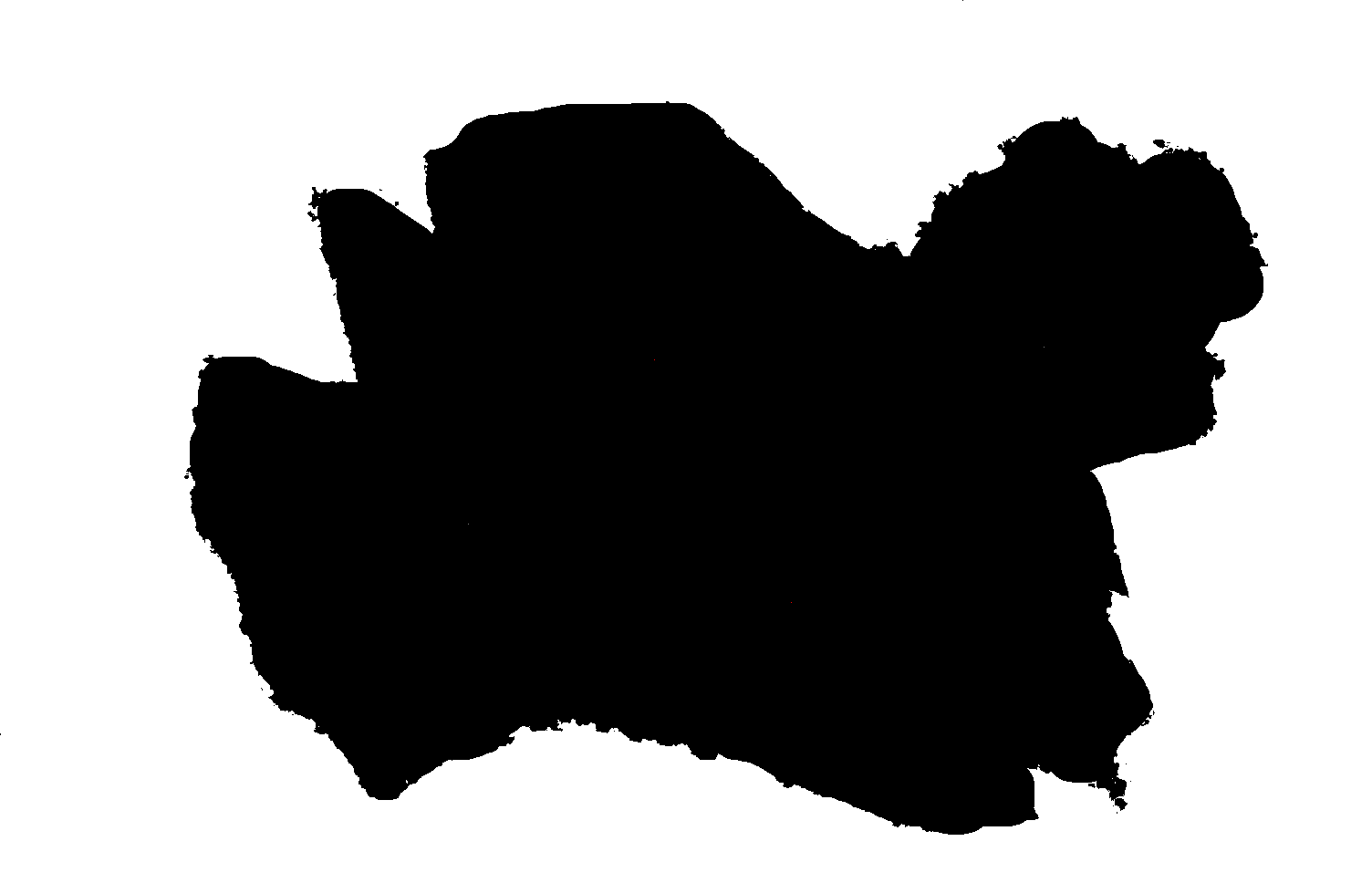

Supplement: Supplementary file 2 [file Datasheet2.zip › figshare/ImageIn/Experiment_039.tif]

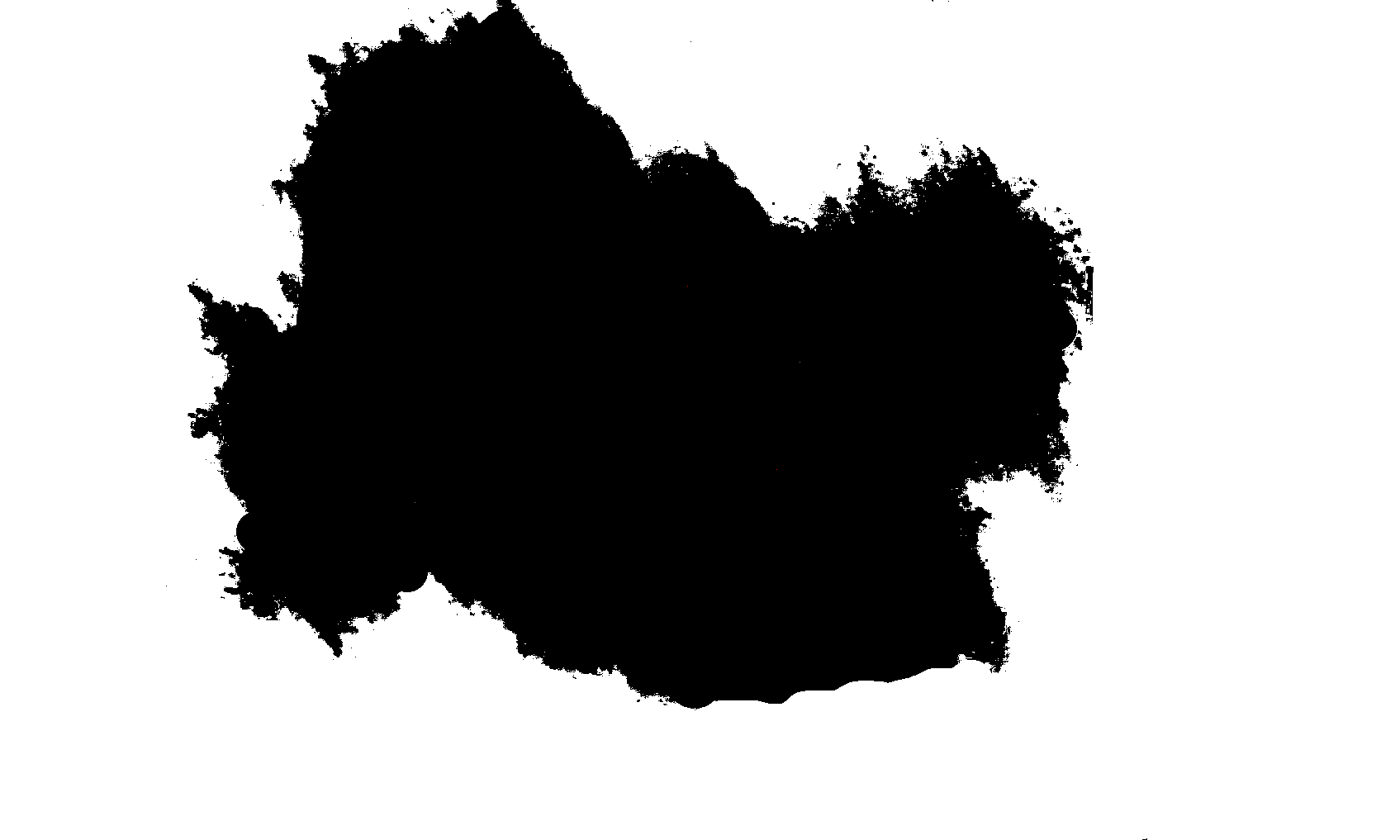

Supplement: Supplementary file 2 [file Datasheet2.zip › figshare/ImageIn/Experiment_040.tif]

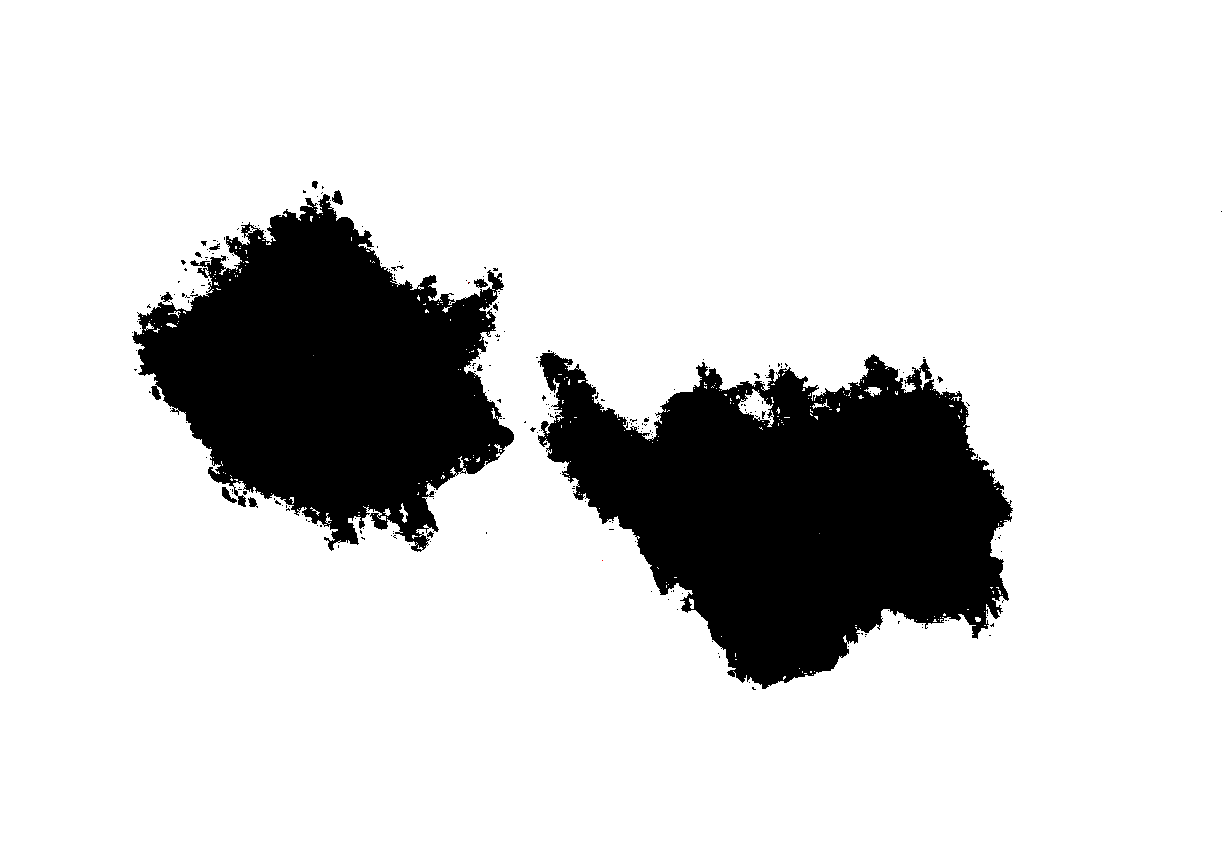

Supplement: Supplementary file 2 [file Datasheet2.zip › figshare/ImageIn/Experiment_041.tif]

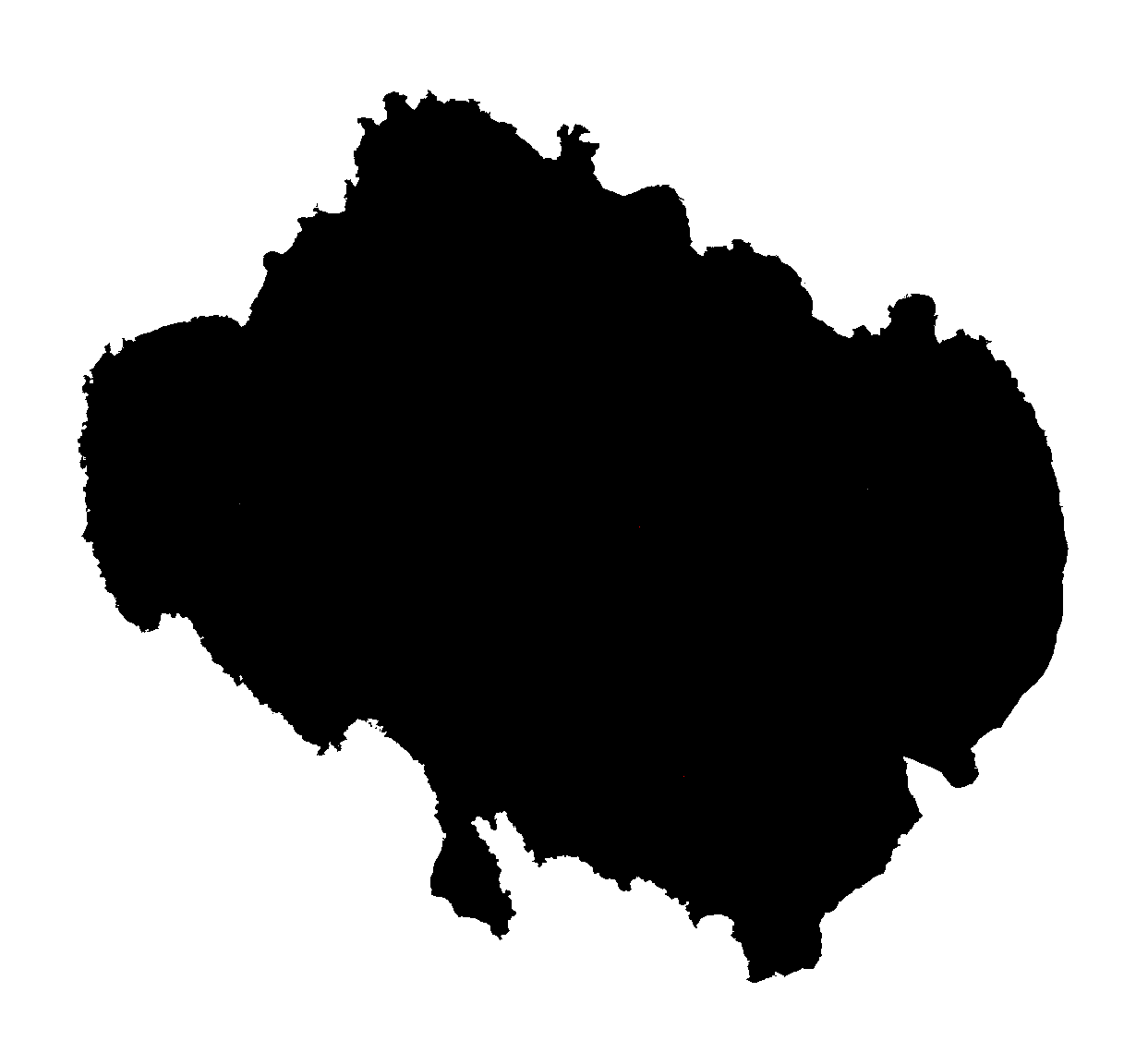

Supplement: Supplementary file 2 [file Datasheet2.zip › figshare/ImageIn/Experiment_042.tif]

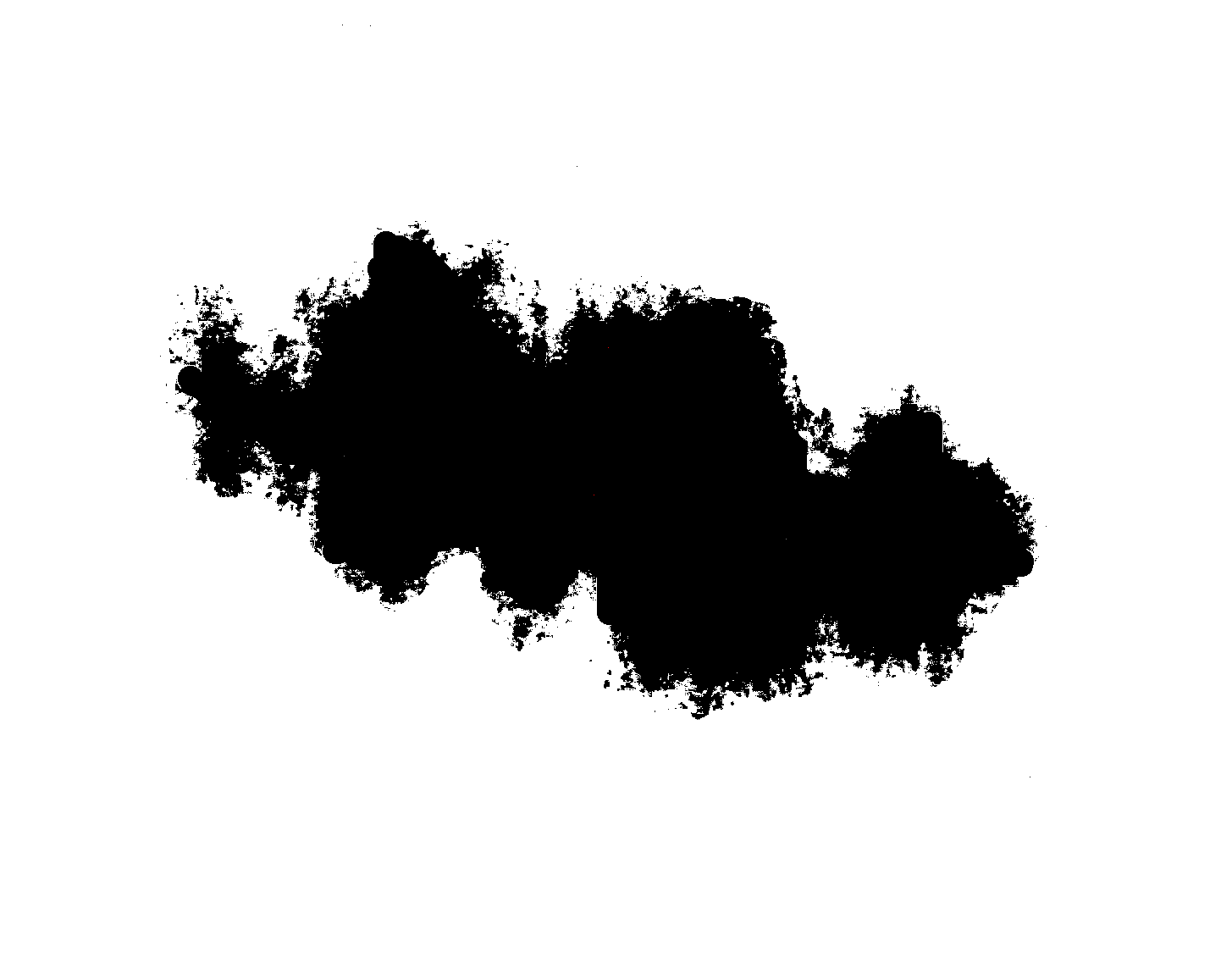

Supplement: Supplementary file 2 [file Datasheet2.zip › figshare/ImageIn/Experiment_043.tif]

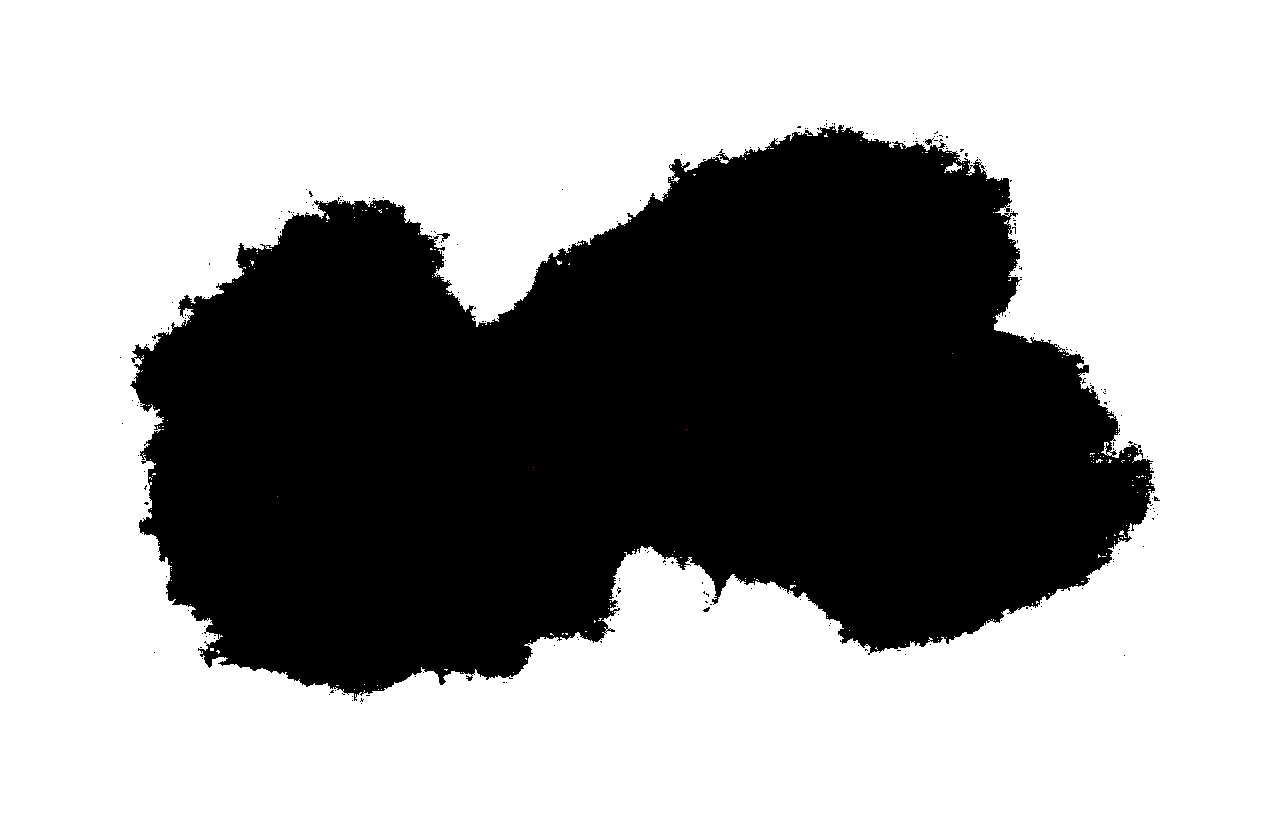

Supplement: Supplementary file 2 [file Datasheet2.zip › figshare/ImageIn/Experiment_044.tif]

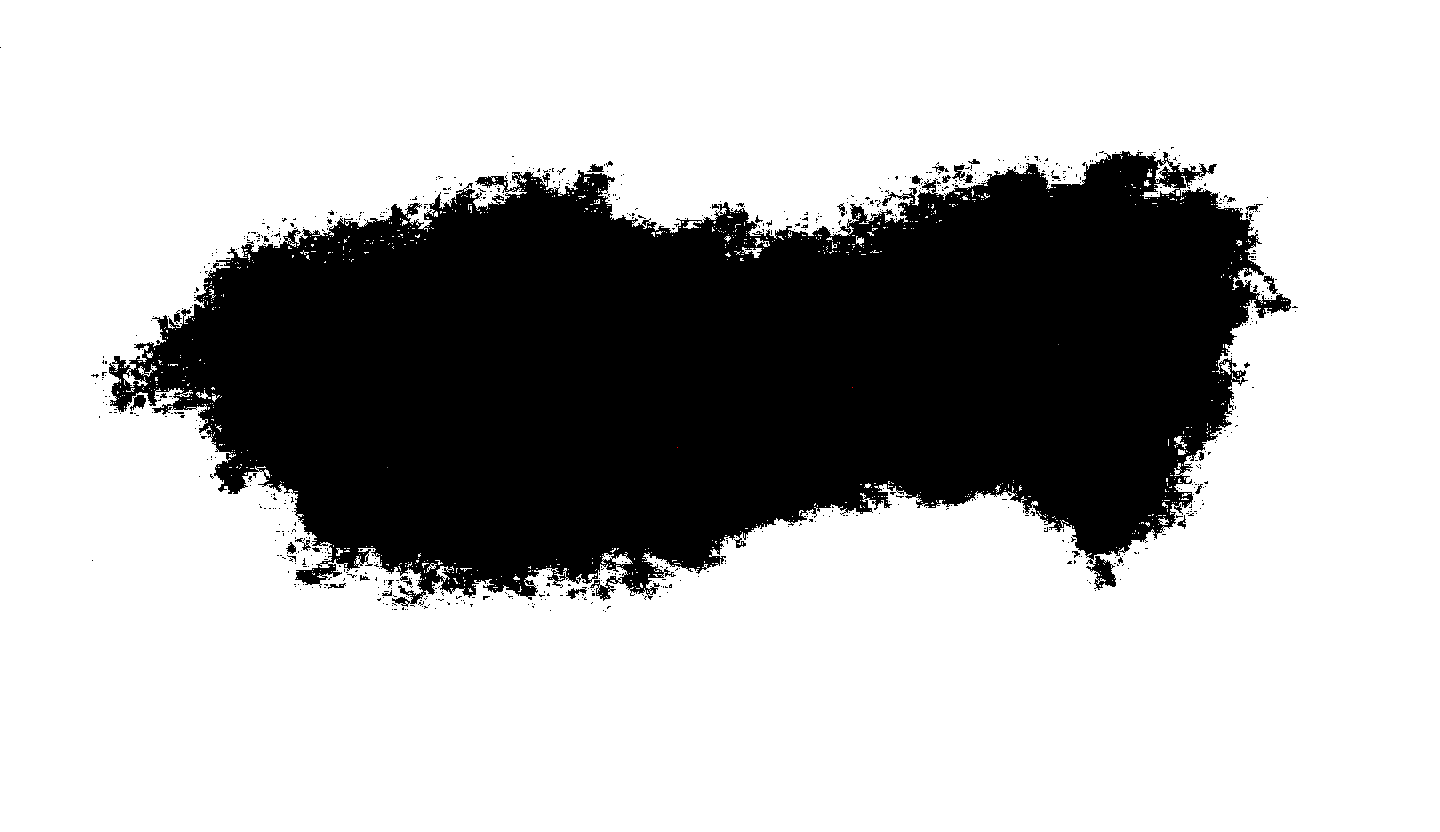

Supplement: Supplementary file 2 [file Datasheet2.zip › figshare/ImageIn/Experiment_045.tif]

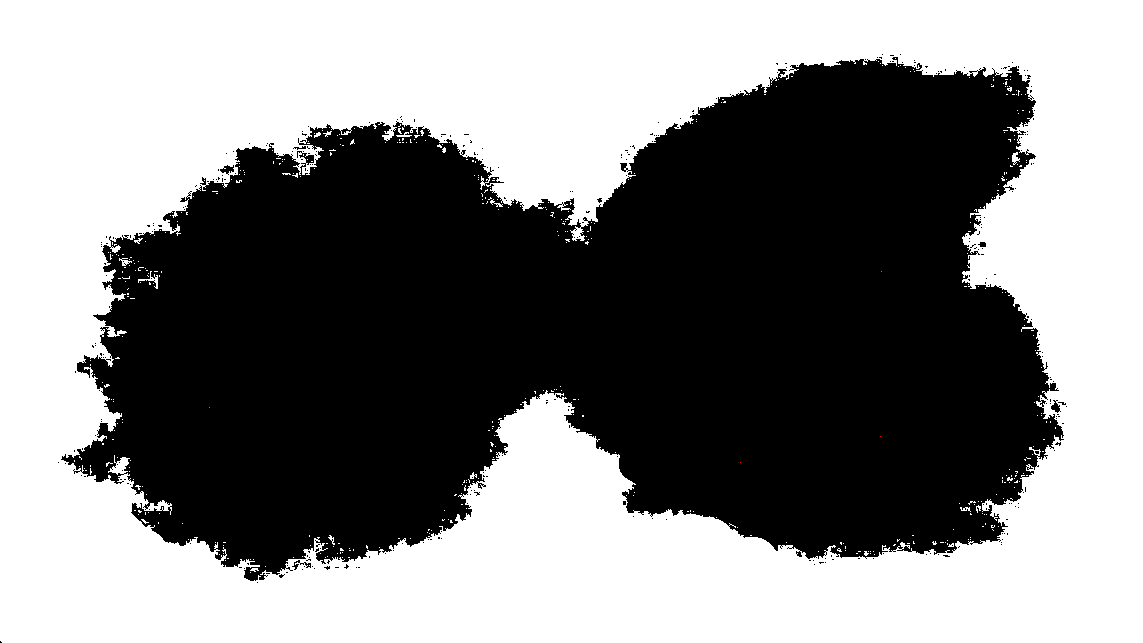

Supplement: Supplementary file 2 [file Datasheet2.zip › figshare/ImageIn/Experiment_046.tif]

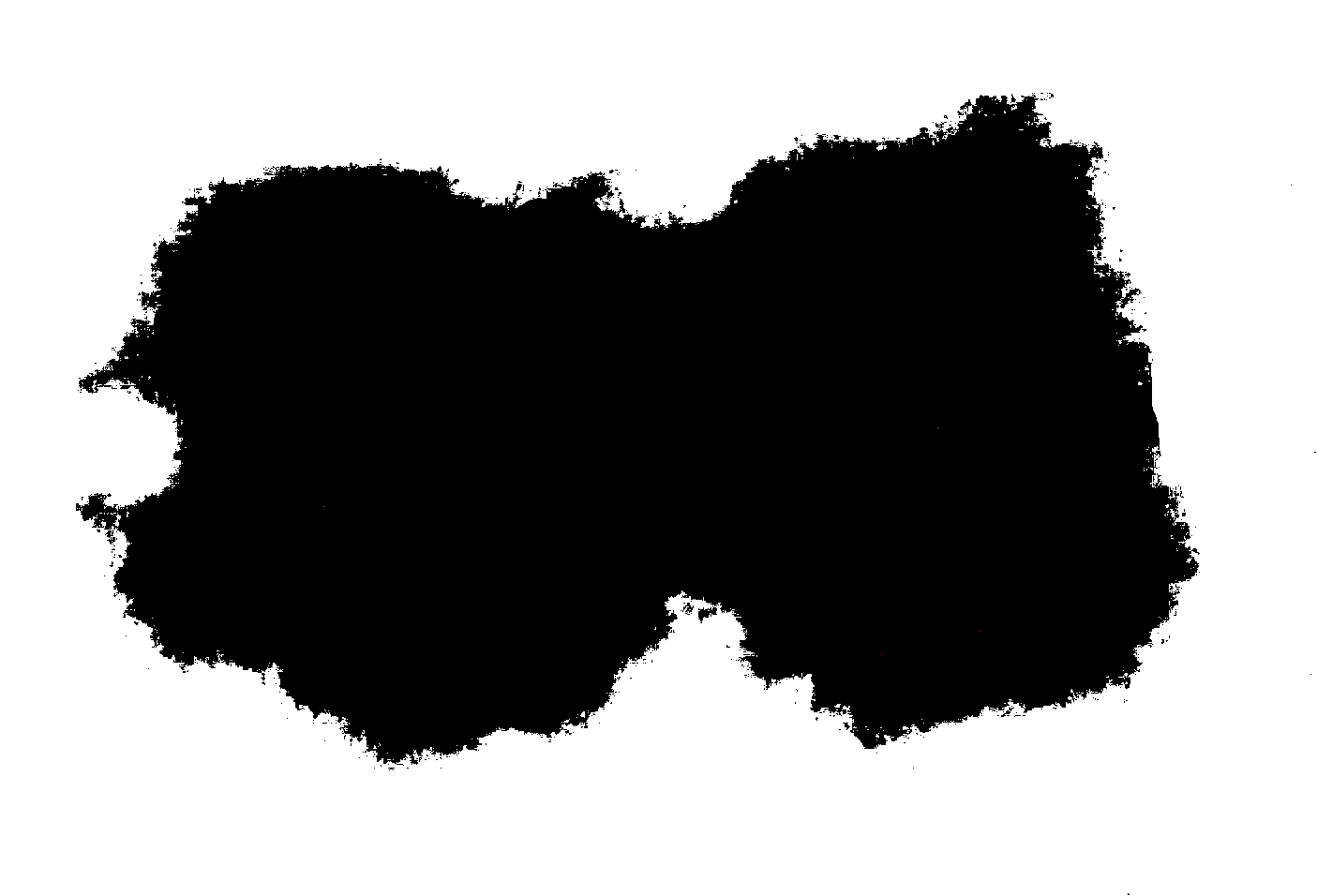

Supplement: Supplementary file 2 [file Datasheet2.zip › figshare/ImageIn/Experiment_047.tif]

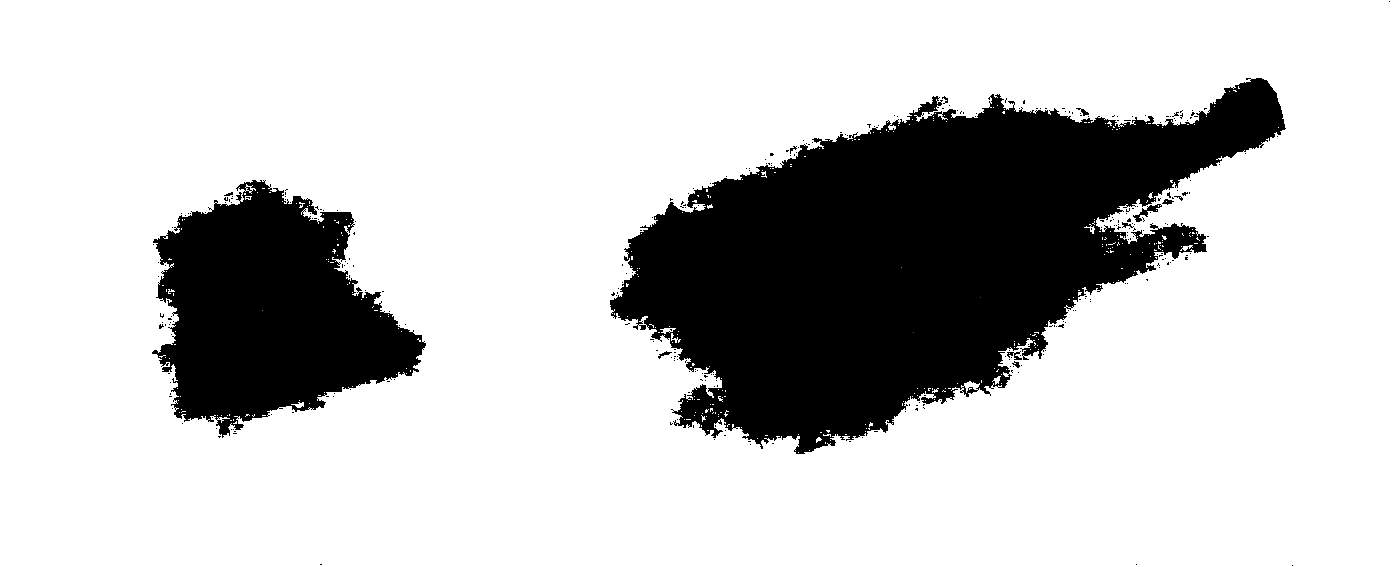

Supplement: Supplementary file 2 [file Datasheet2.zip › figshare/ImageIn/Experiment_048.tif]

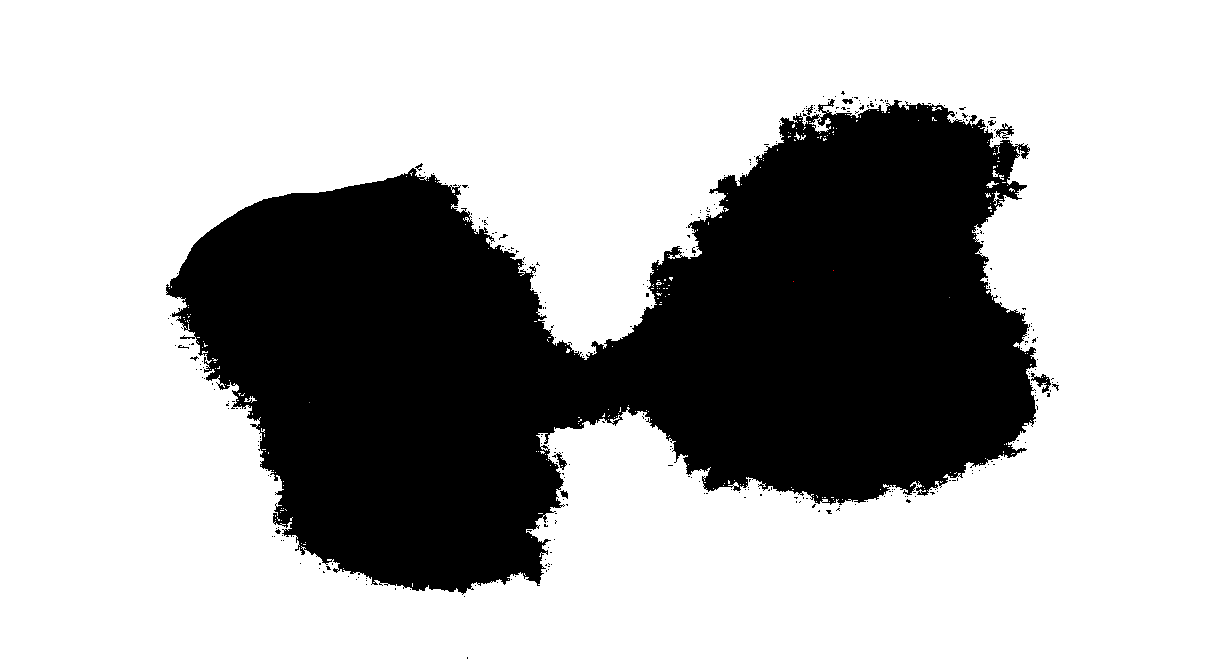

Supplement: Supplementary file 2 [file Datasheet2.zip › figshare/ImageIn/Experiment_049.tif]

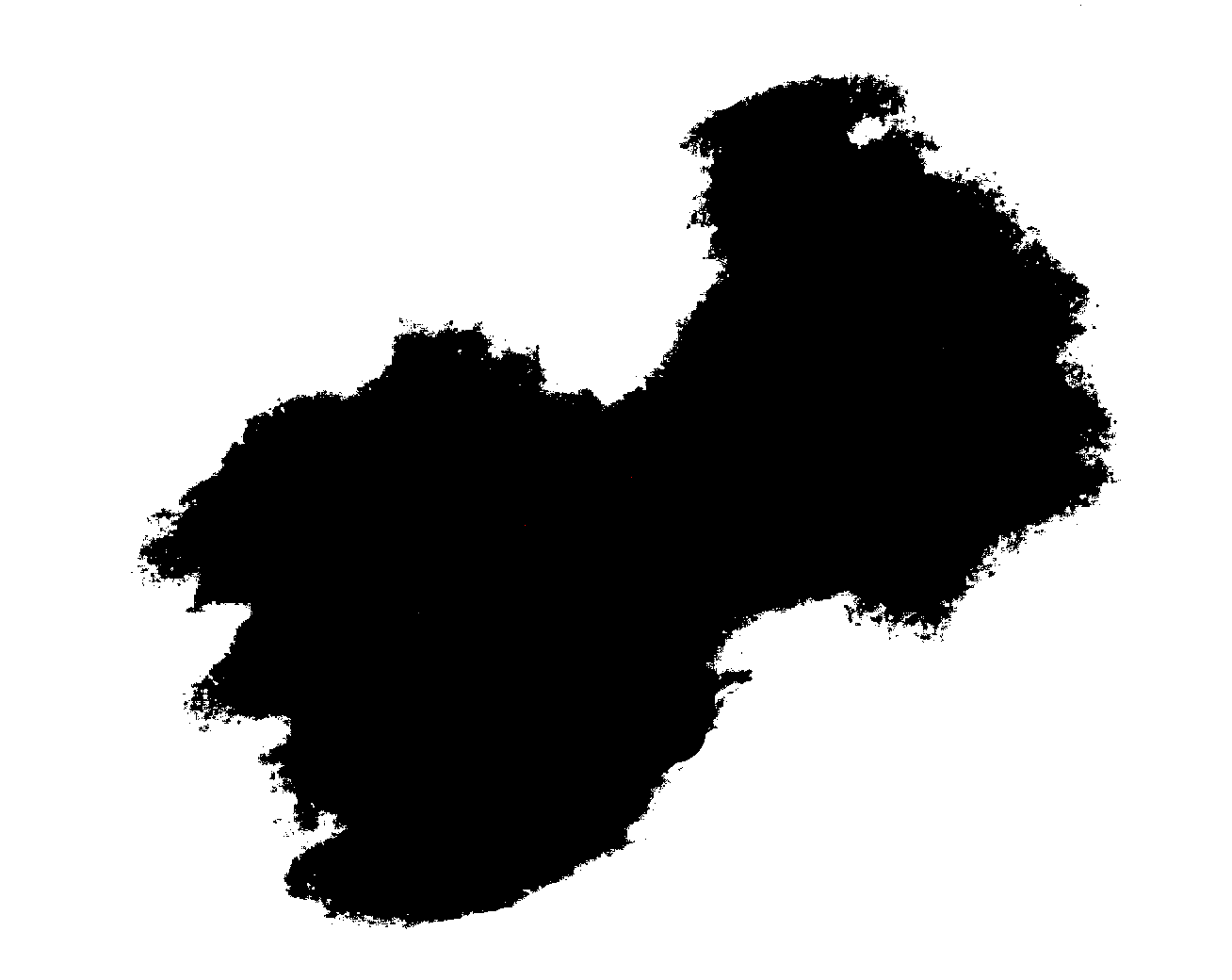

Supplement: Supplementary file 2 [file Datasheet2.zip › figshare/ImageIn/Experiment_050.tif]

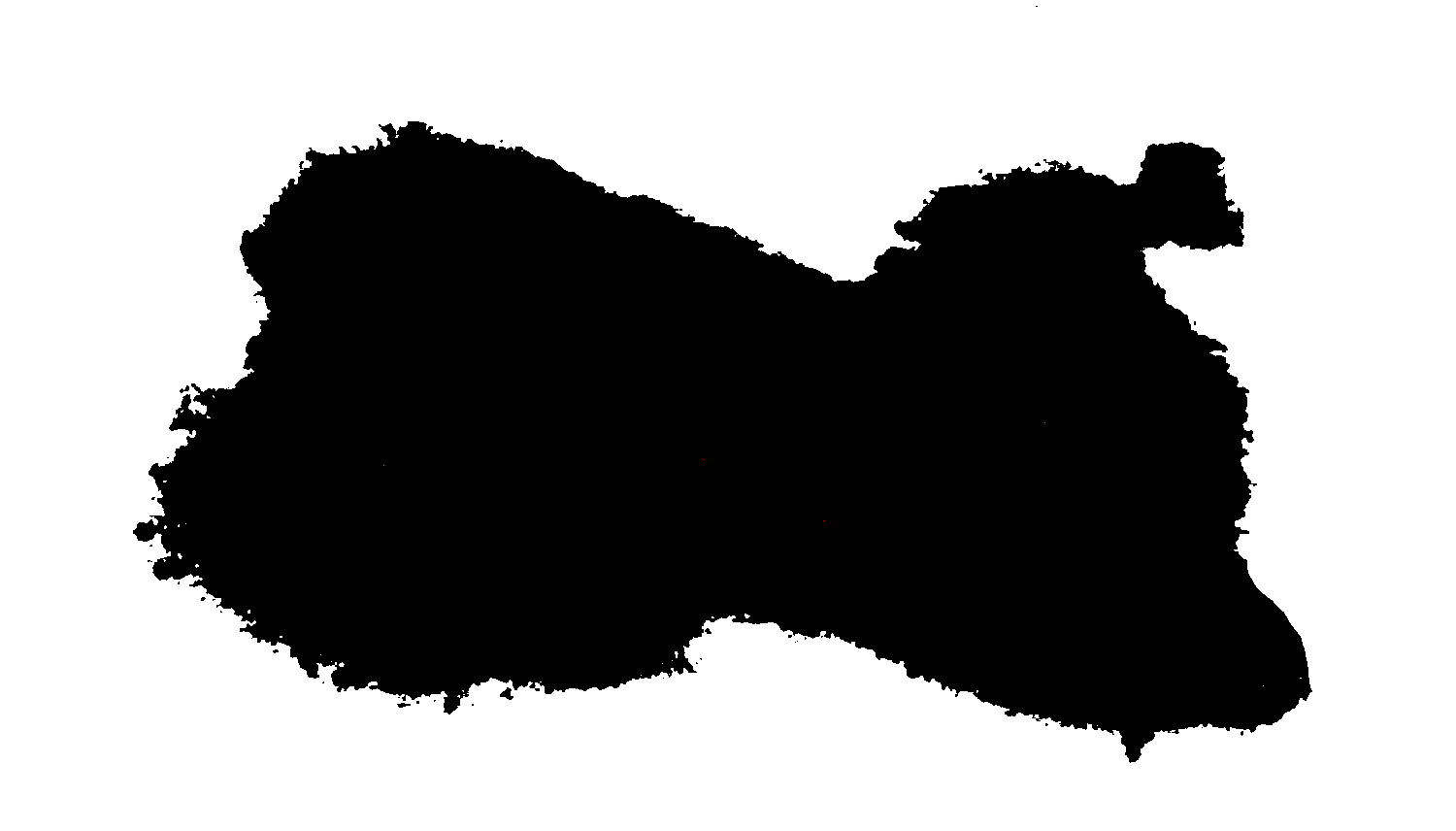

Supplement: Supplementary file 2 [file Datasheet2.zip › figshare/ImageIn/Experiment_051.tif]

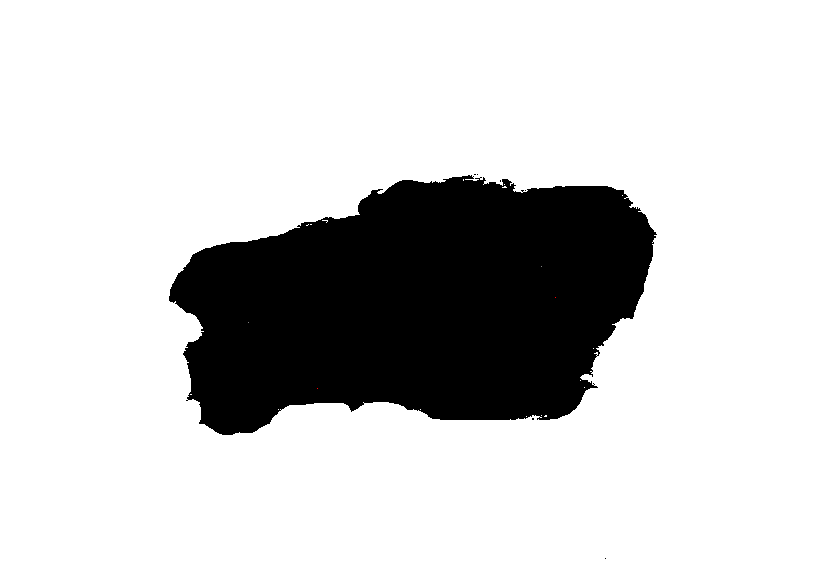

Supplement: Supplementary file 2 [file Datasheet2.zip › figshare/ImageIn/Experiment_052.tif]

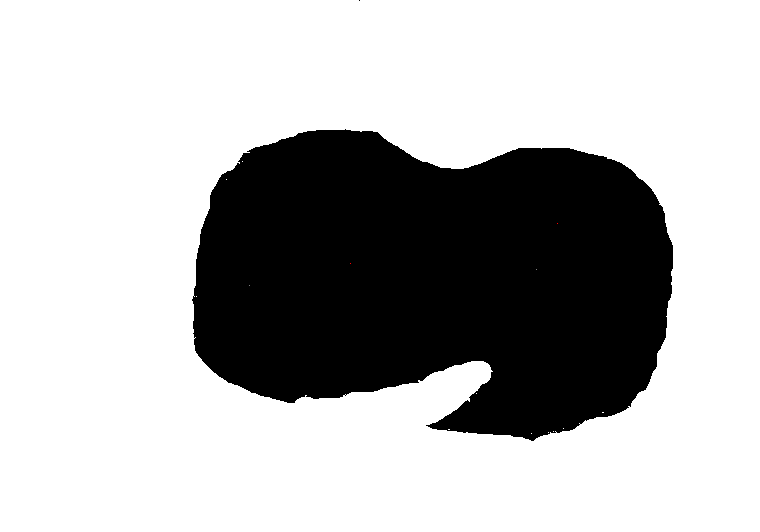

Supplement: Supplementary file 2 [file Datasheet2.zip › figshare/ImageIn/Experiment_053.tif]

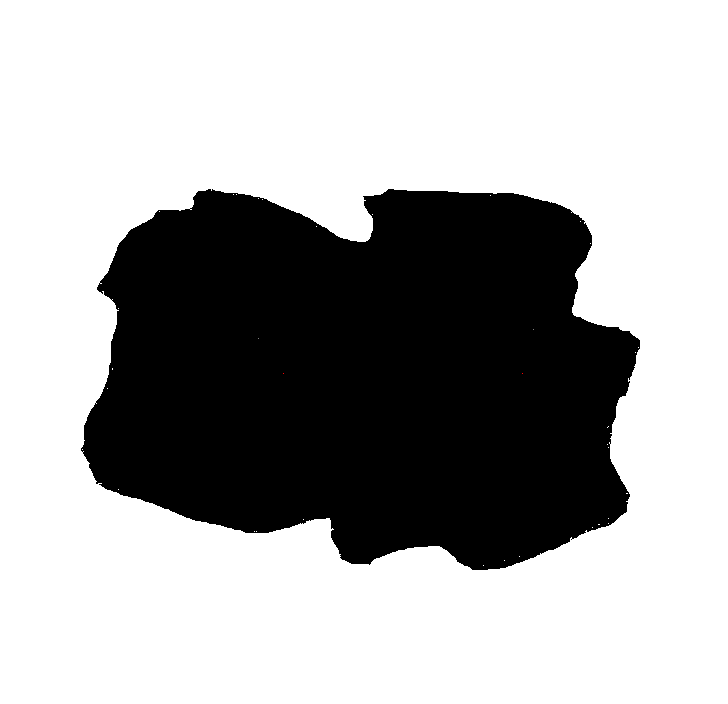

Supplement: Supplementary file 2 [file Datasheet2.zip › figshare/ImageIn/Experiment_054.tif]

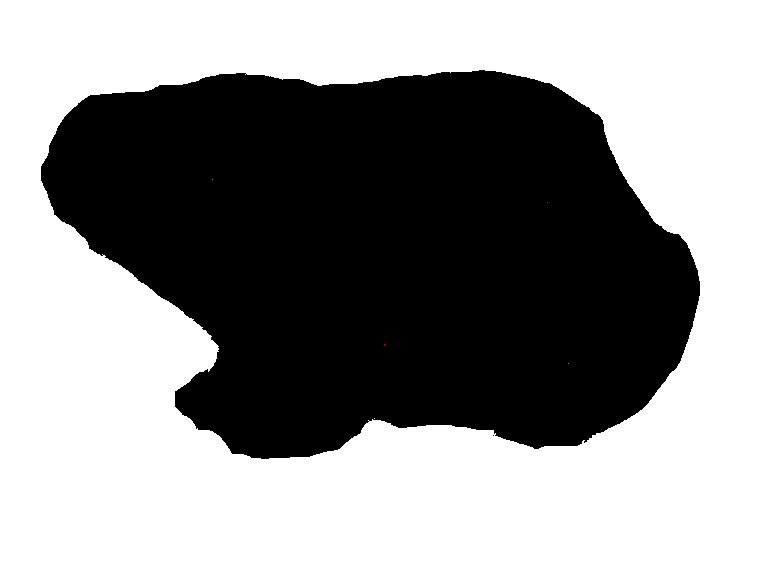

Supplement: Supplementary file 2 [file Datasheet2.zip › figshare/ImageIn/Experiment_055.tif]

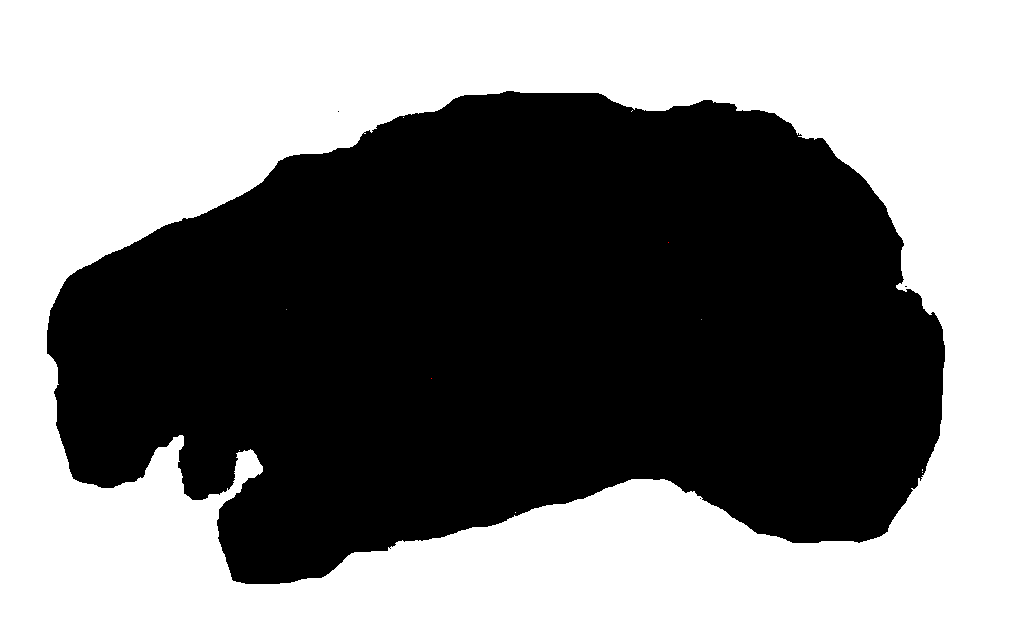

Supplement: Supplementary file 2 [file Datasheet2.zip › figshare/ImageIn/Experiment_056.tif]

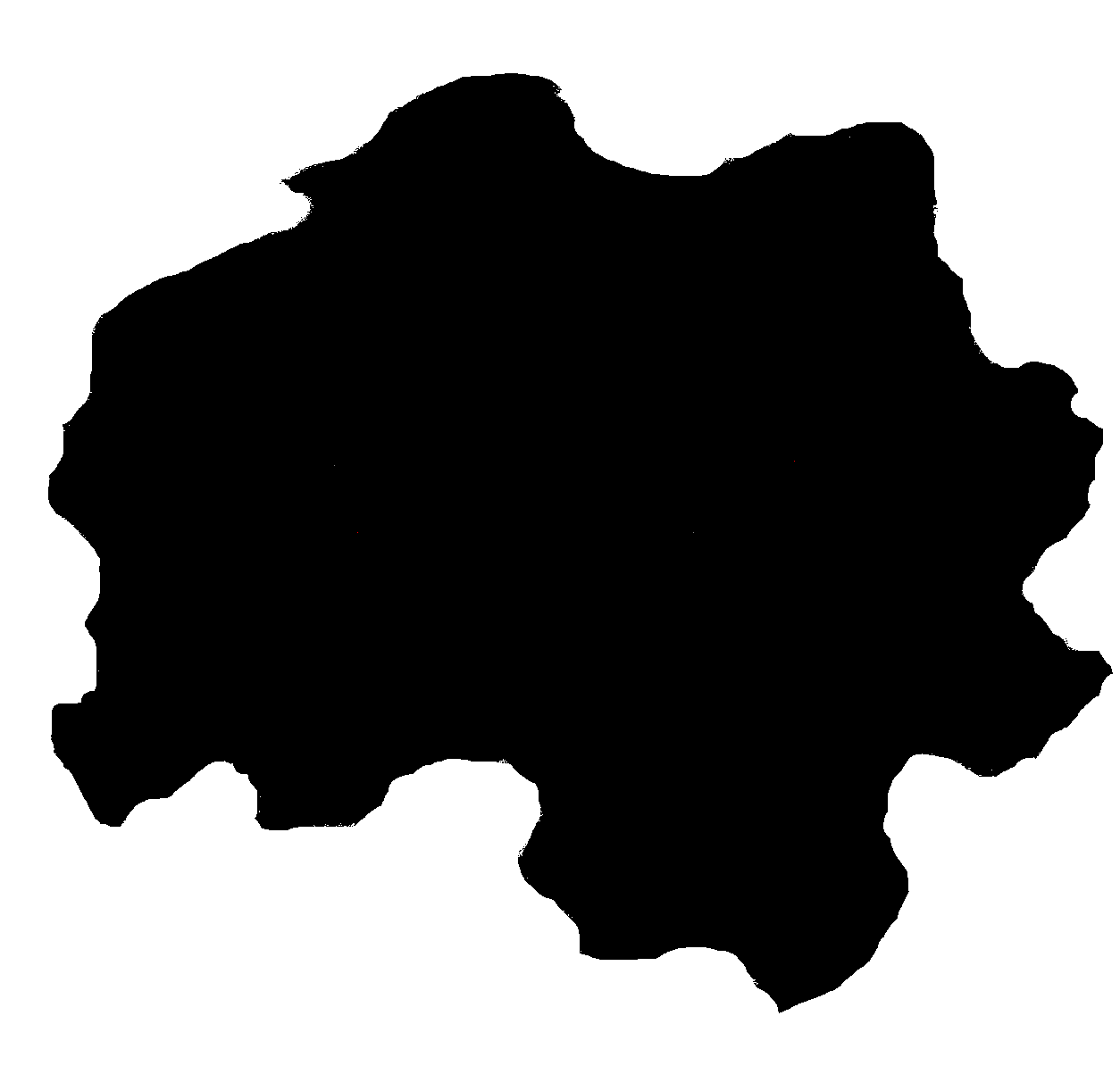

Supplement: Supplementary file 2 [file Datasheet2.zip › figshare/ImageIn/Experiment_057.tif]

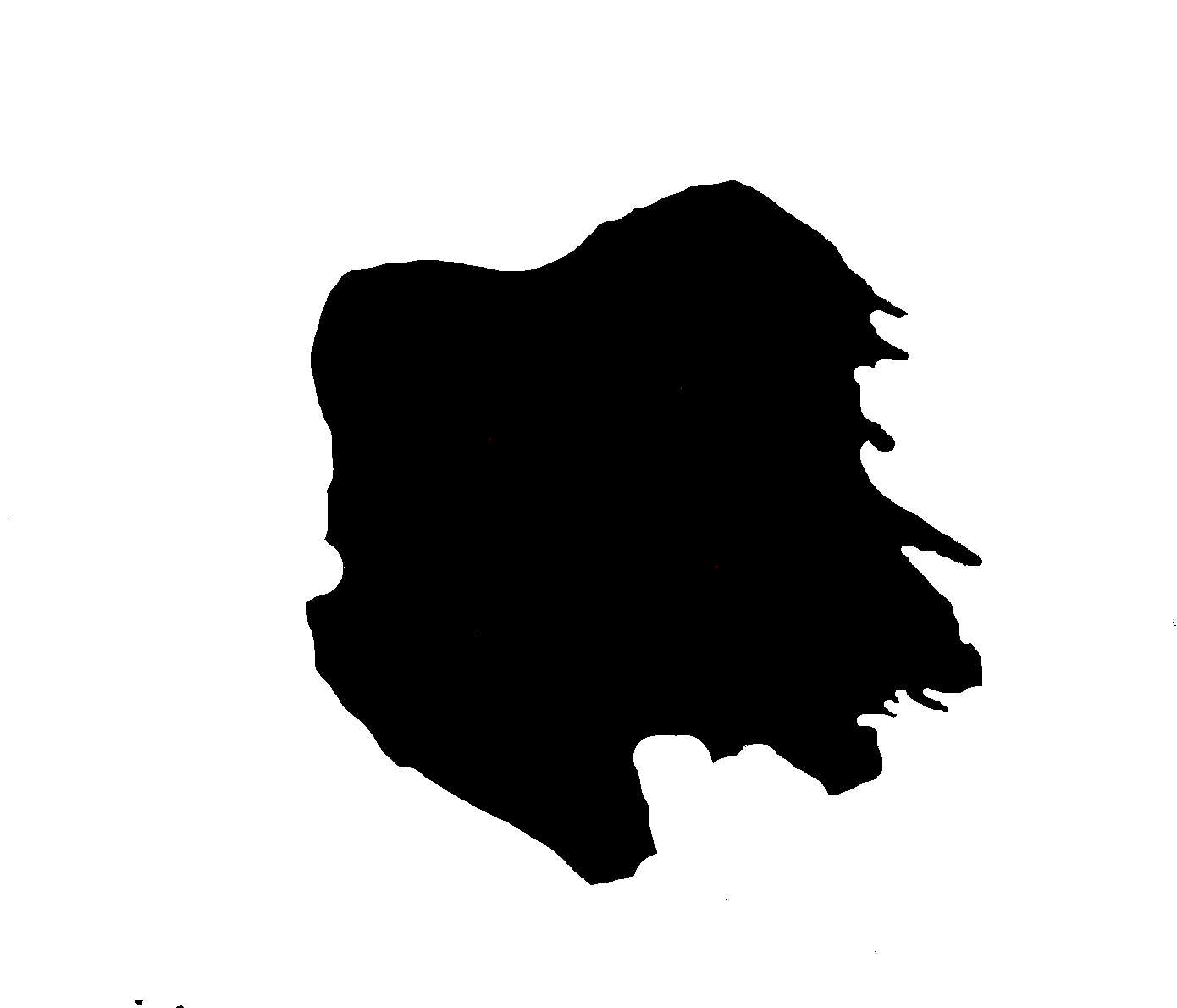

Supplement: Supplementary file 2 [file Datasheet2.zip › figshare/ImageIn/Experiment_058.tif]

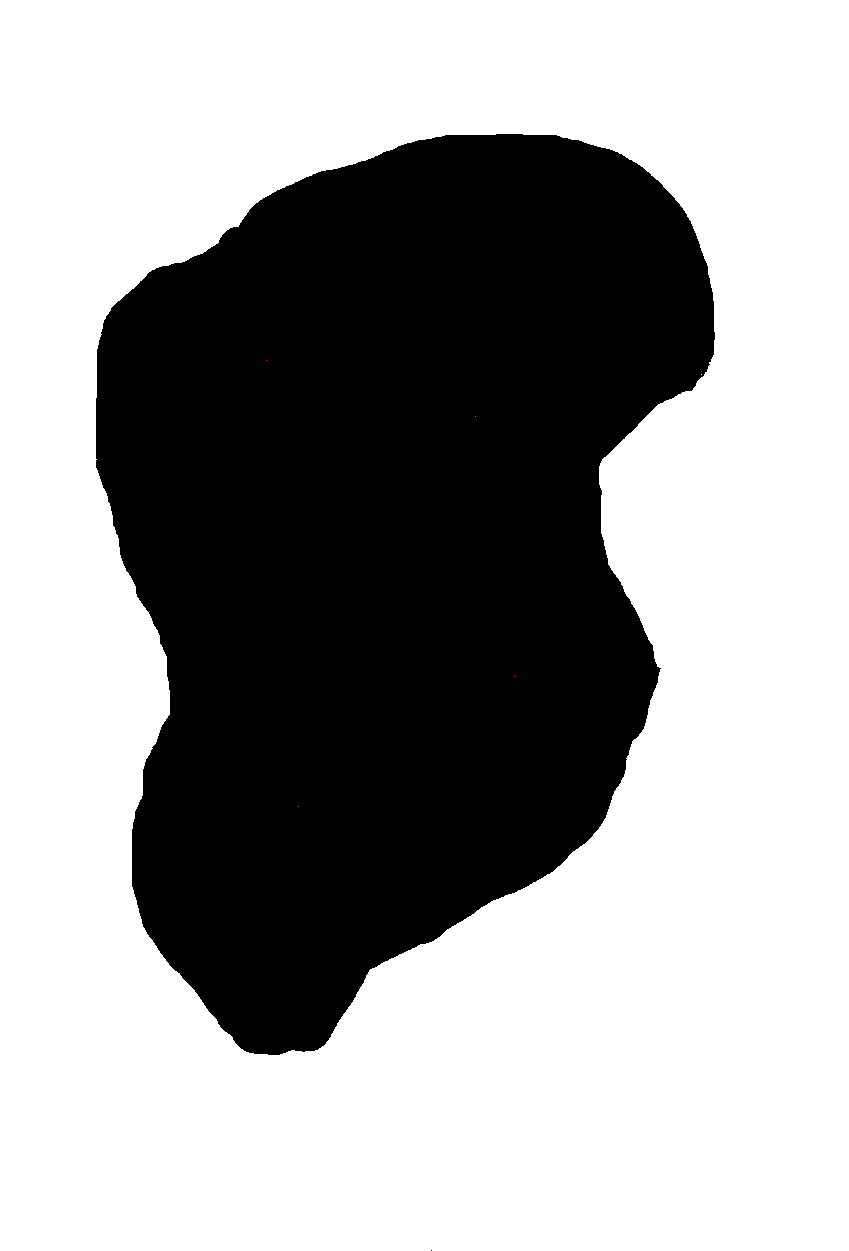

Supplement: Supplementary file 2 [file Datasheet2.zip › figshare/ImageIn/Experiment_059.tif]

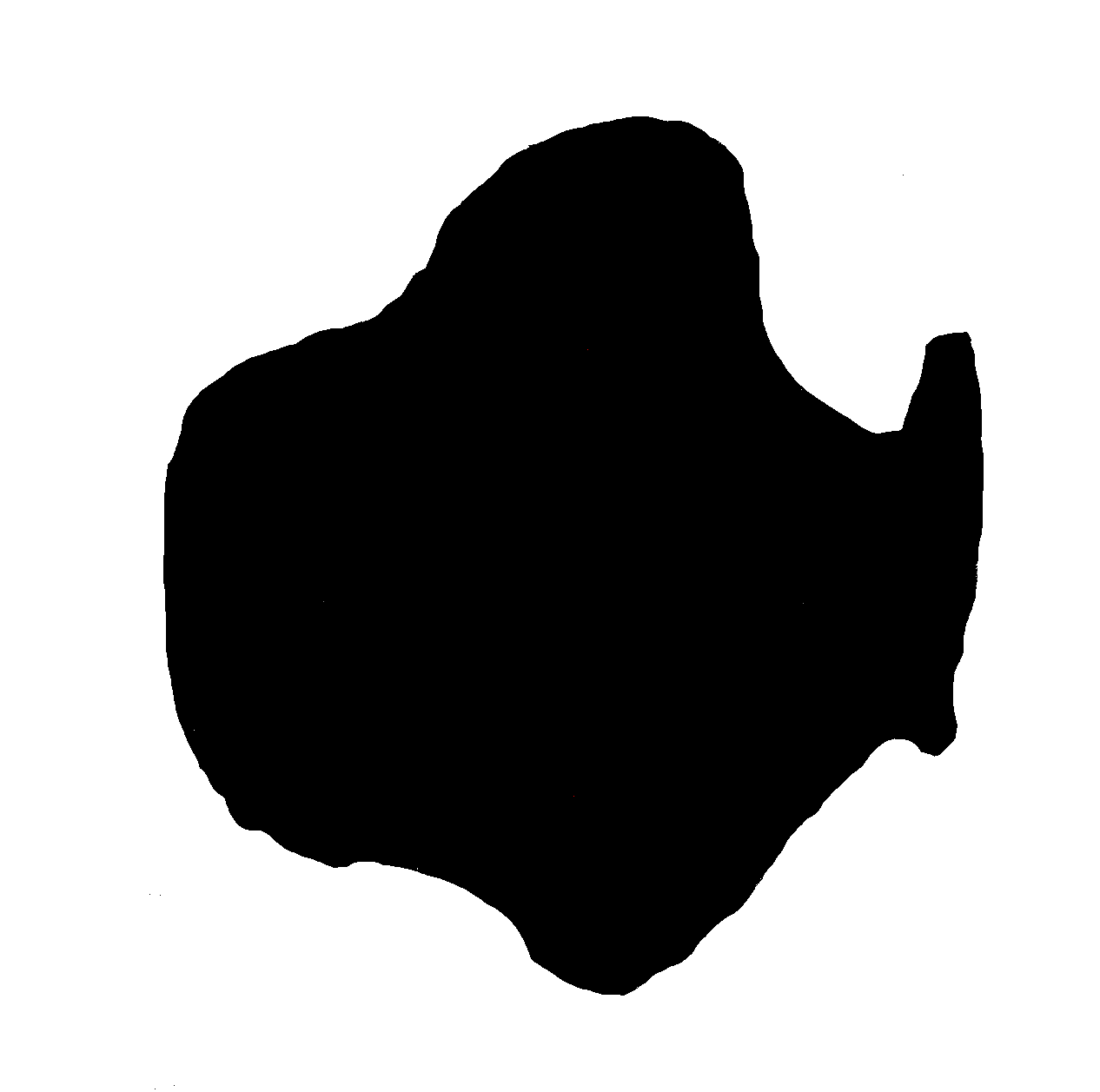

Supplement: Supplementary file 2 [file Datasheet2.zip › figshare/ImageIn/Experiment_060.tif]

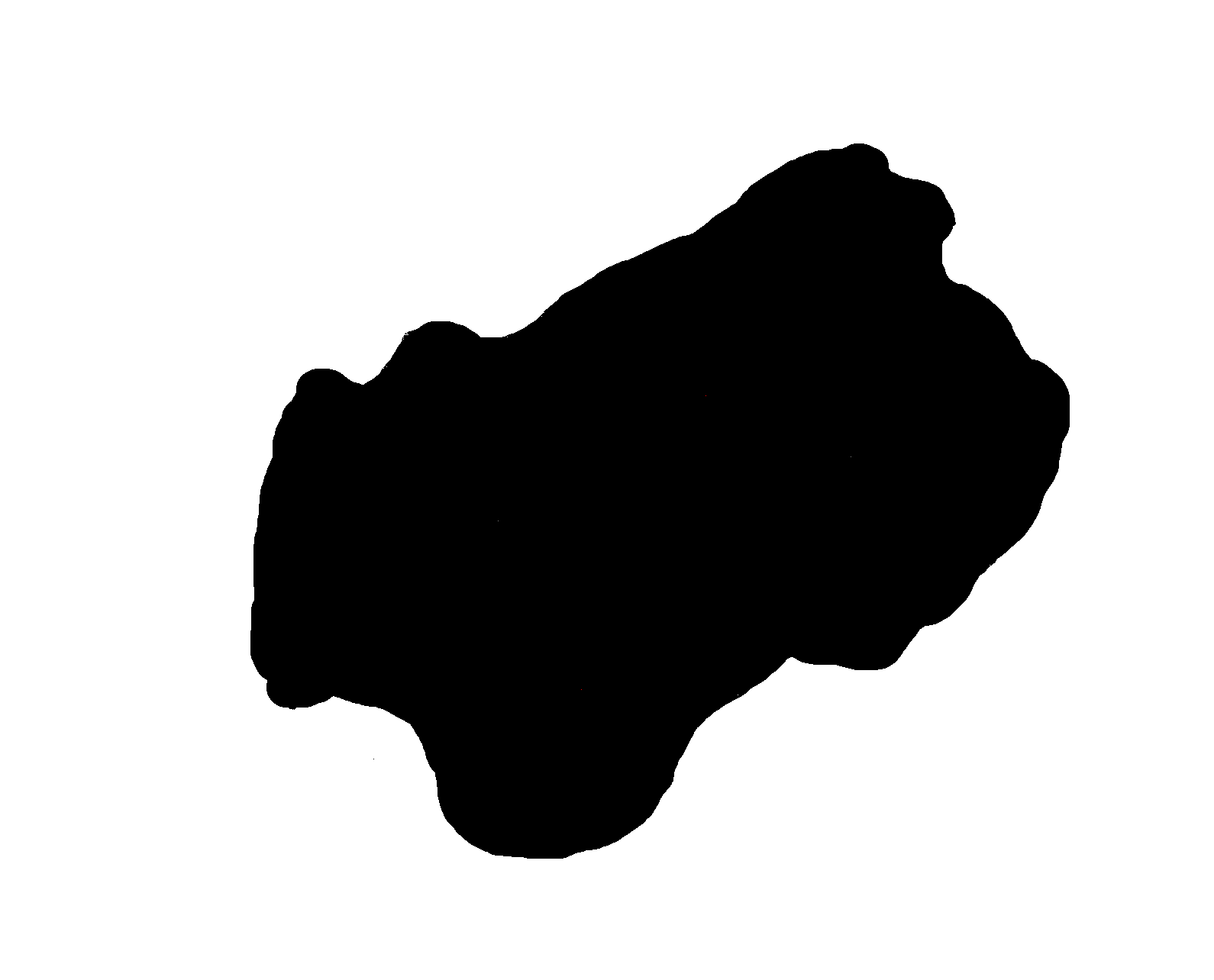

Supplement: Supplementary file 2 [file Datasheet2.zip › figshare/ImageIn/Experiment_061.tif]

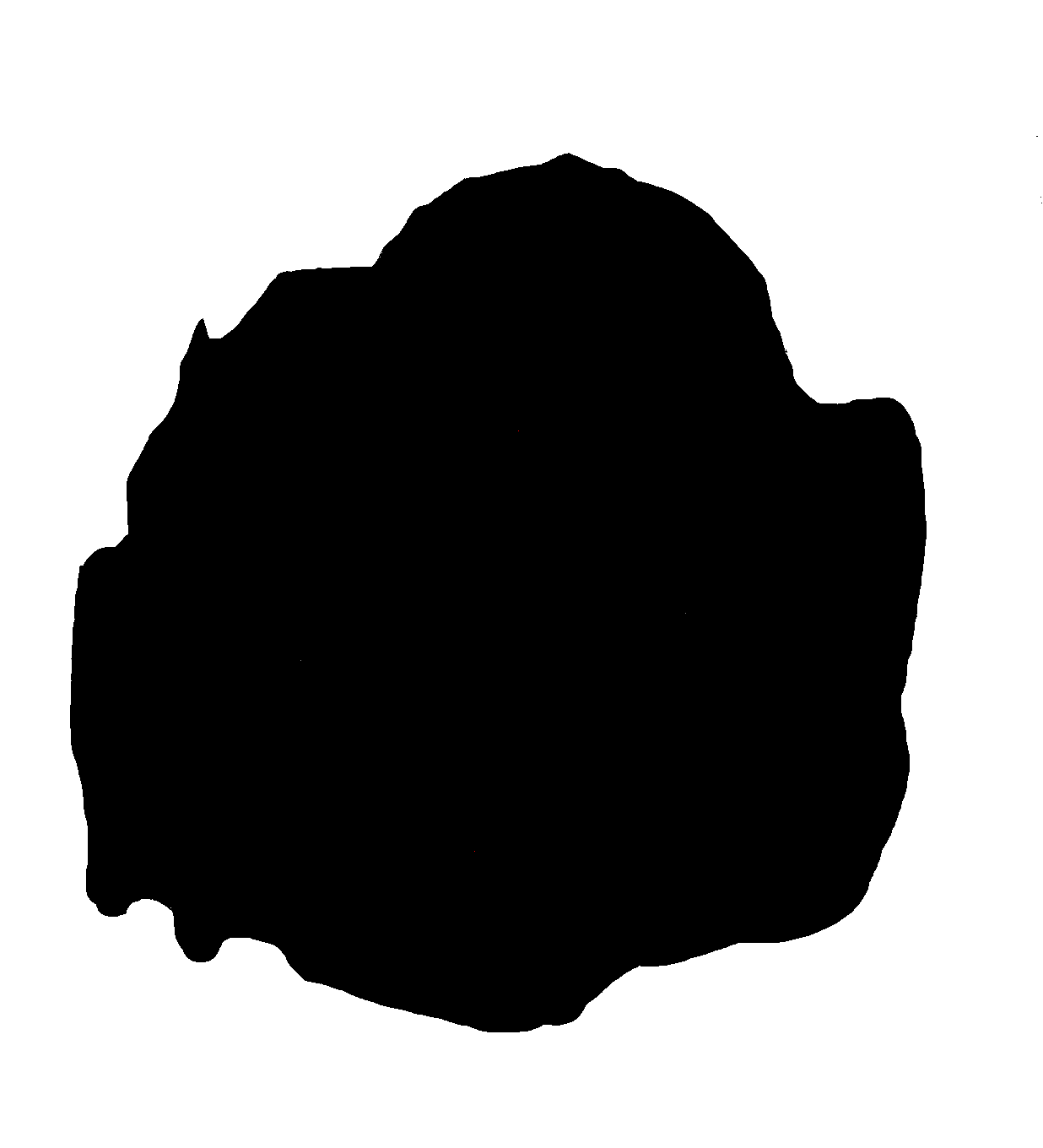

Supplement: Supplementary file 2 [file Datasheet2.zip › figshare/ImageIn/Experiment_062.tif]

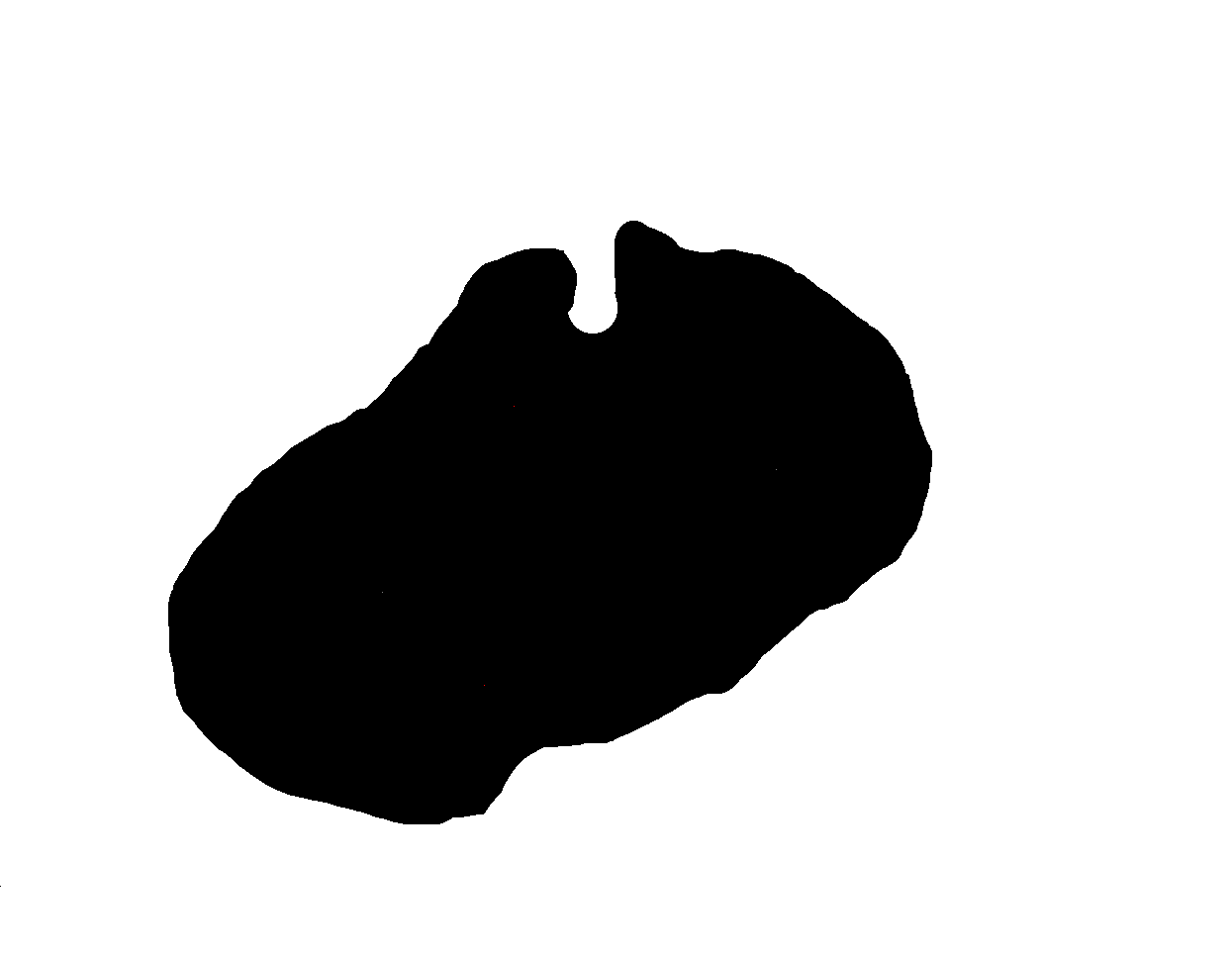

Supplement: Supplementary file 2 [file Datasheet2.zip › figshare/ImageIn/Experiment_063.tif]

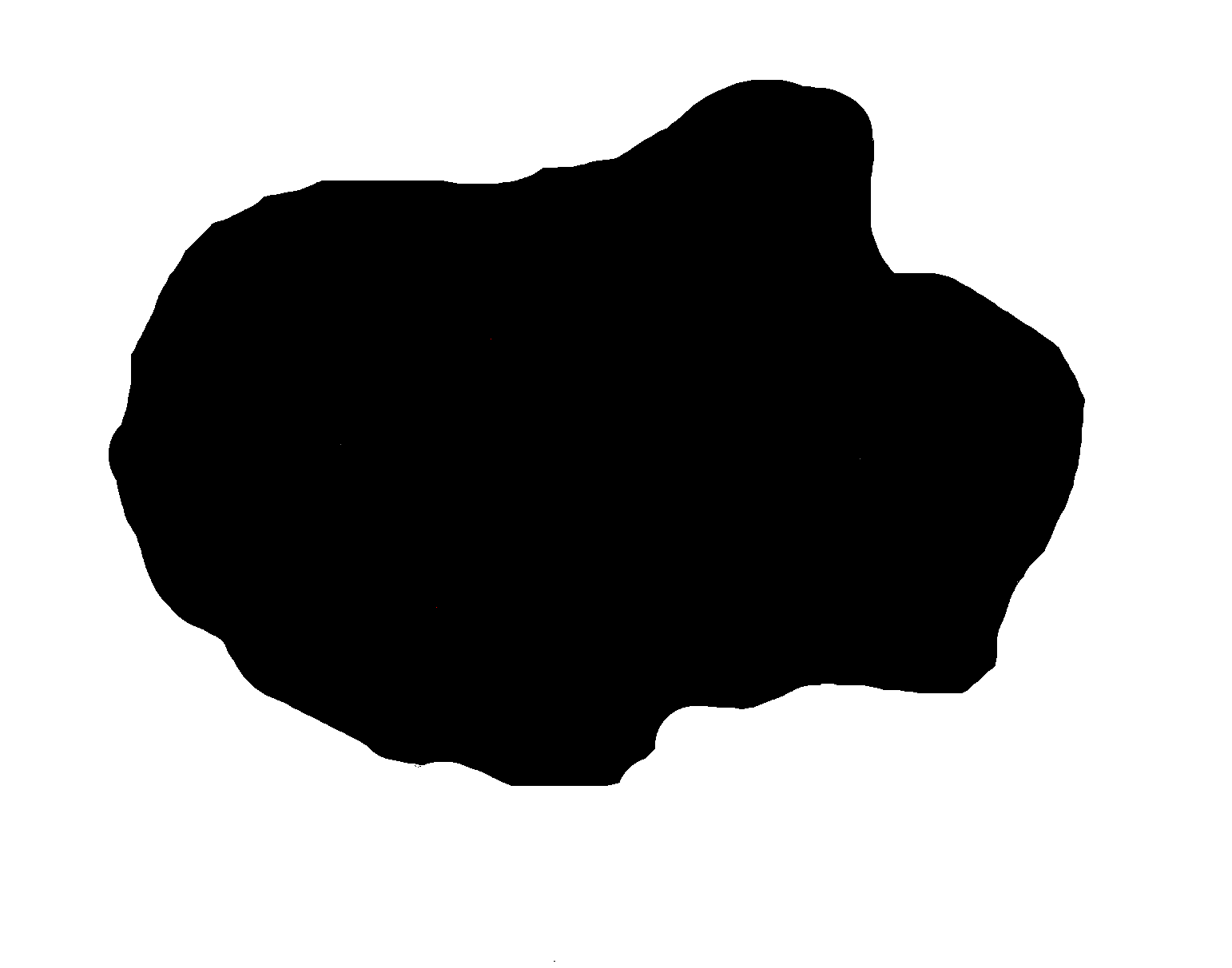

Supplement: Supplementary file 2 [file Datasheet2.zip › figshare/ImageIn/Experiment_064.tif]

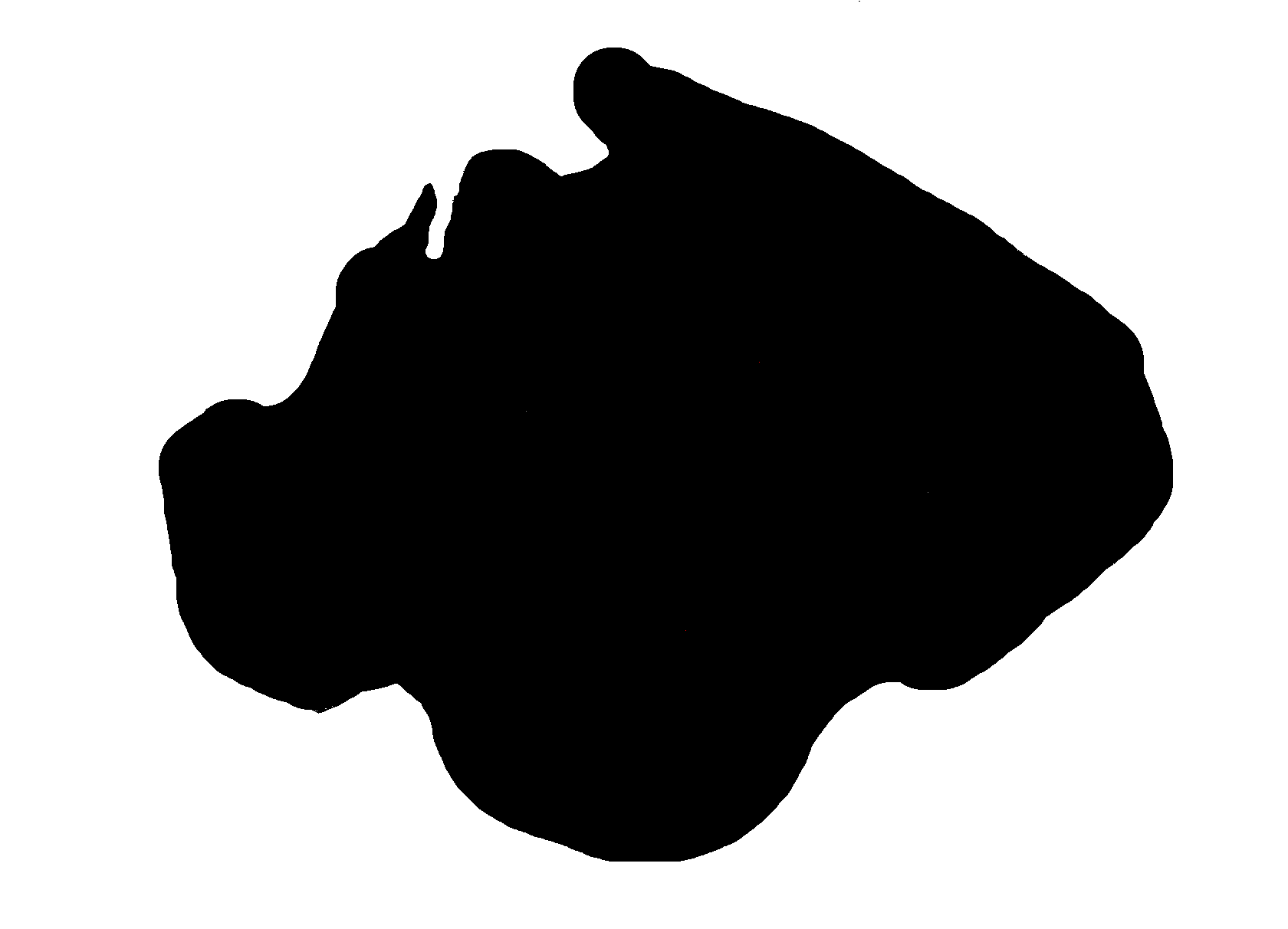

Supplement: Supplementary file 2 [file Datasheet2.zip › figshare/ImageIn/Experiment_065.tif]

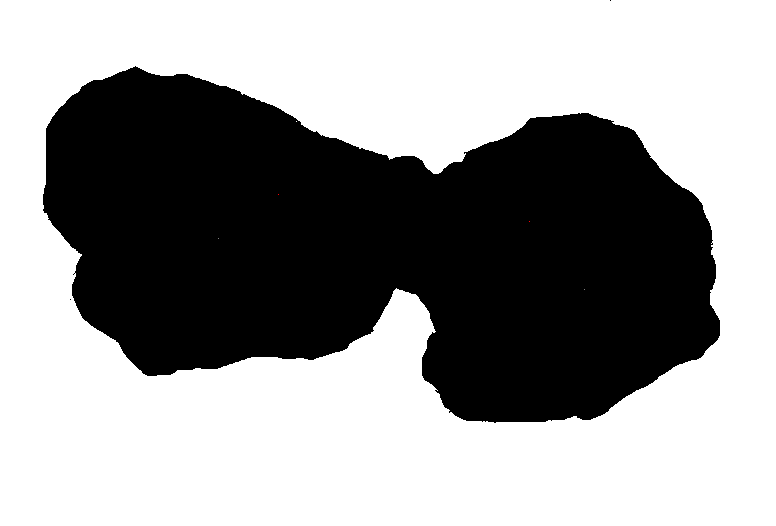

Supplement: Supplementary file 2 [file Datasheet2.zip › figshare/ImageIn/Experiment_066.tif]

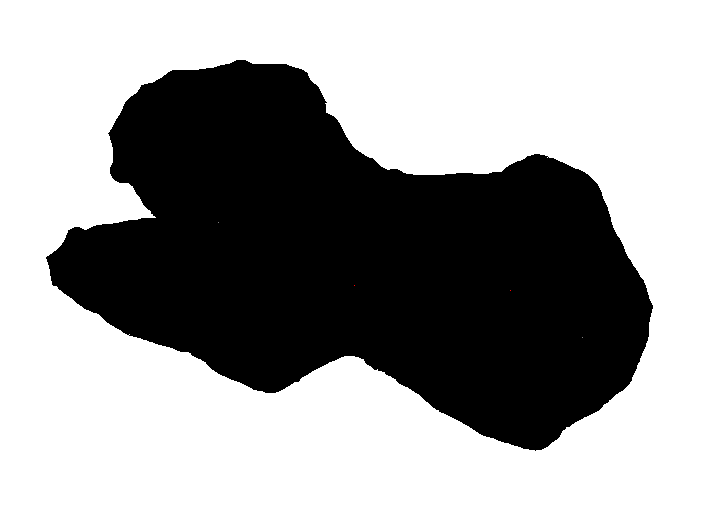

Supplement: Supplementary file 2 [file Datasheet2.zip › figshare/ImageIn/Experiment_067.tif]

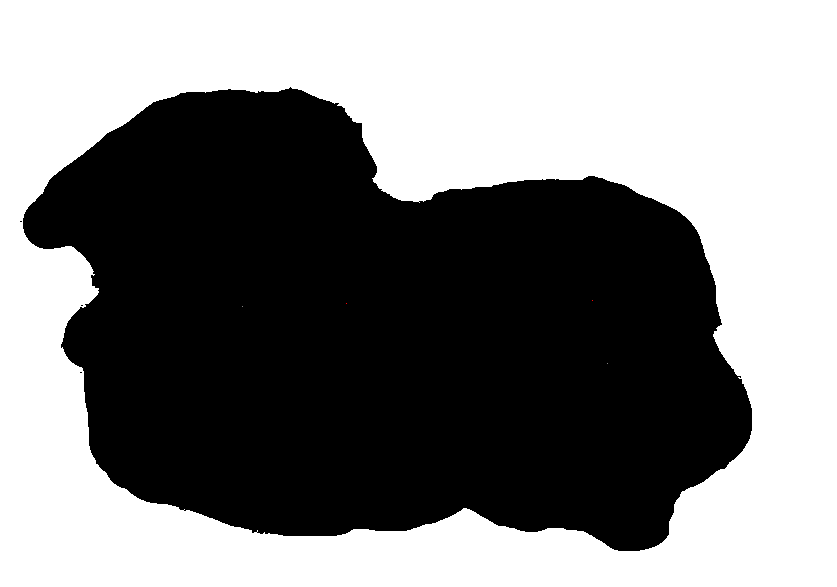

Supplement: Supplementary file 2 [file Datasheet2.zip › figshare/ImageIn/Experiment_068.tif]

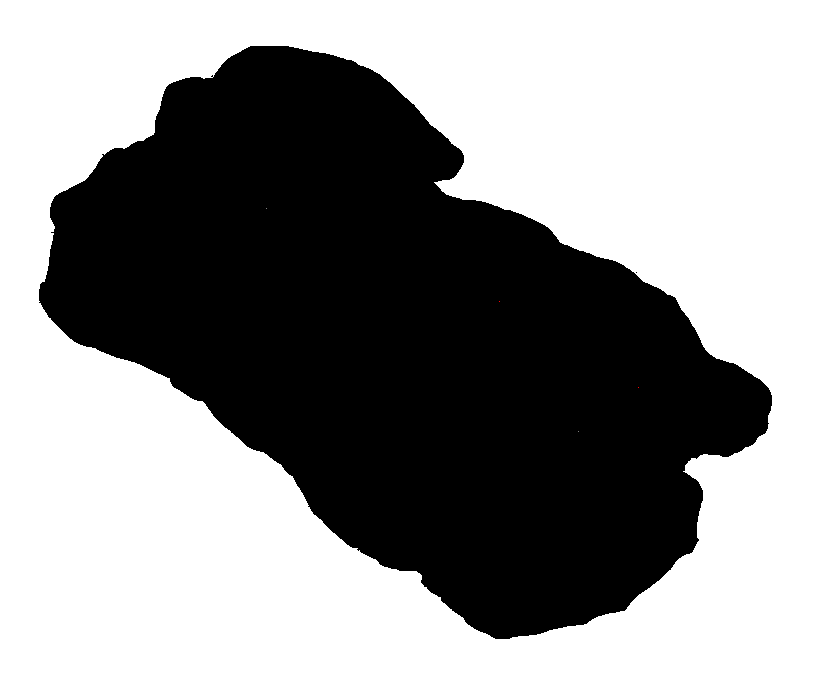

Supplement: Supplementary file 2 [file Datasheet2.zip › figshare/ImageIn/Experiment_069.tif]

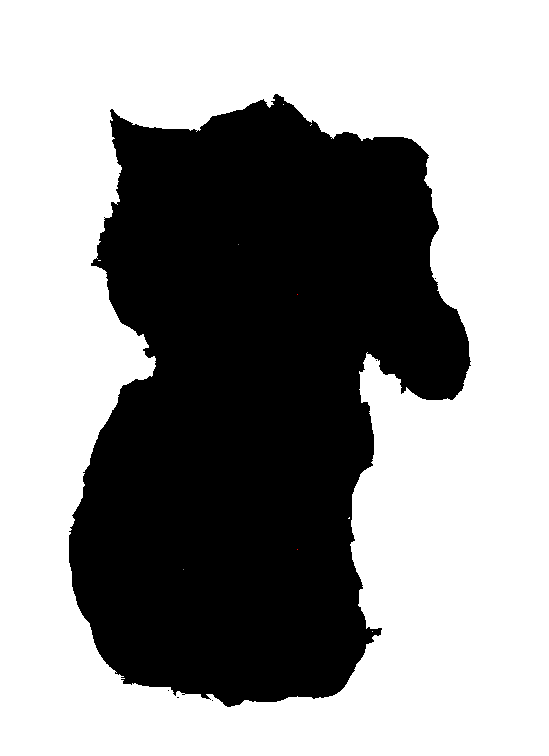

Supplement: Supplementary file 2 [file Datasheet2.zip › figshare/ImageIn/Experiment_070.tif]

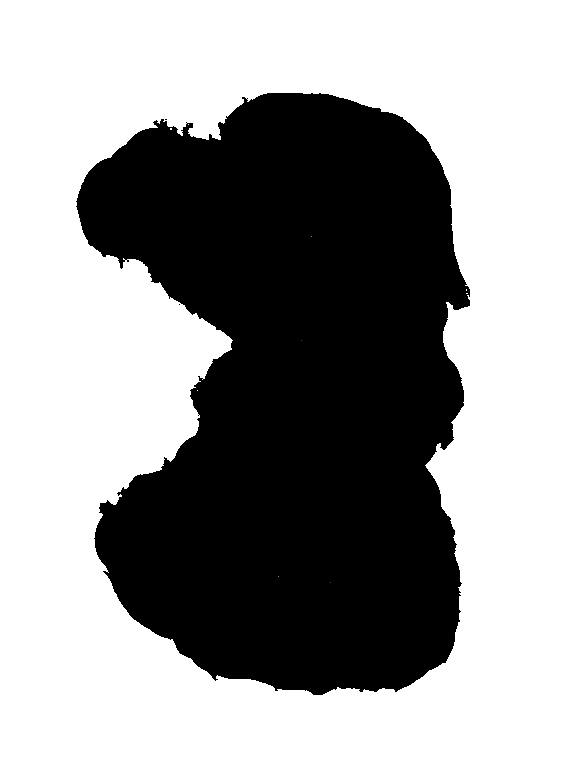

Supplement: Supplementary file 2 [file Datasheet2.zip › figshare/ImageIn/Experiment_071.tif]

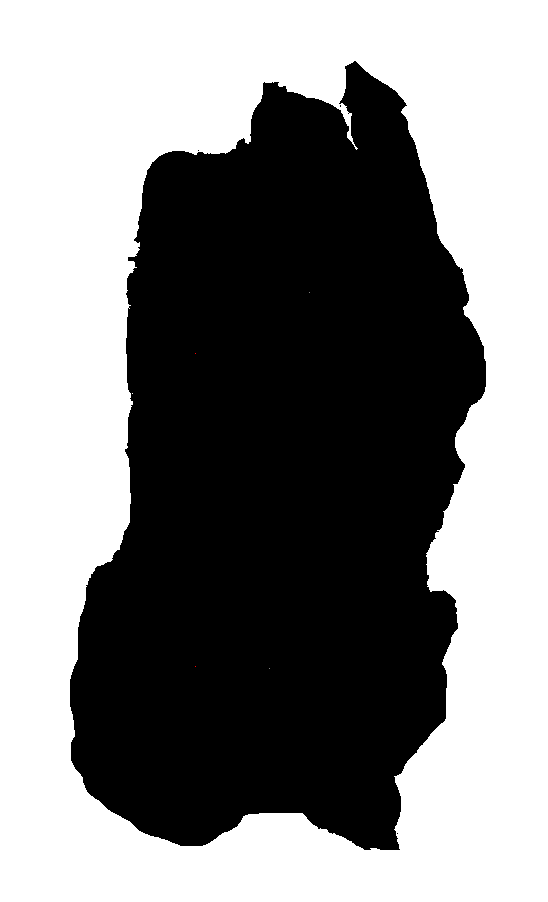

Supplement: Supplementary file 2 [file Datasheet2.zip › figshare/ImageIn/Experiment_072.tif]

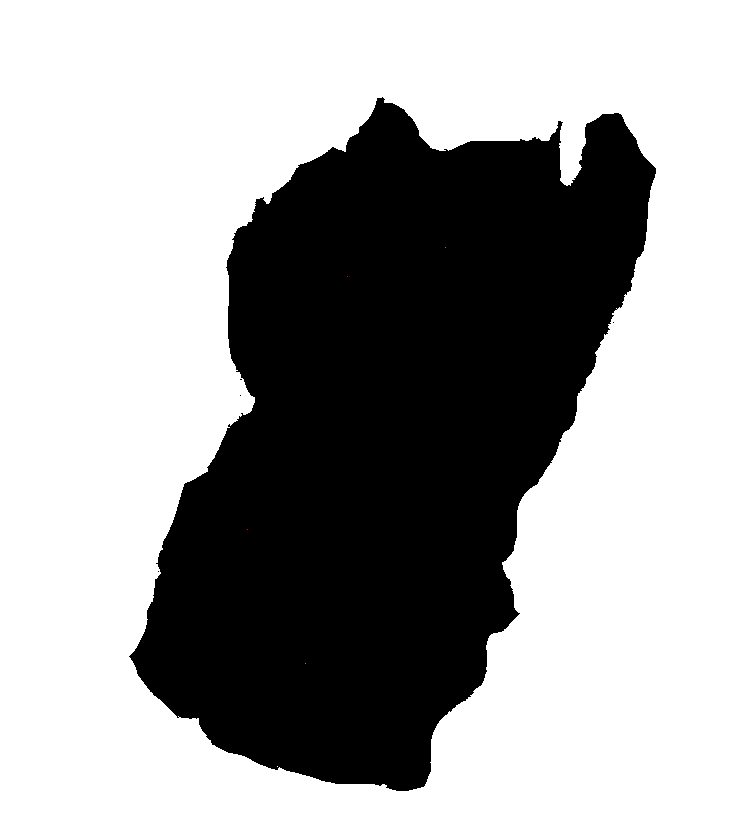

Supplement: Supplementary file 2 [file Datasheet2.zip › figshare/ImageIn/Experiment_073.tif]

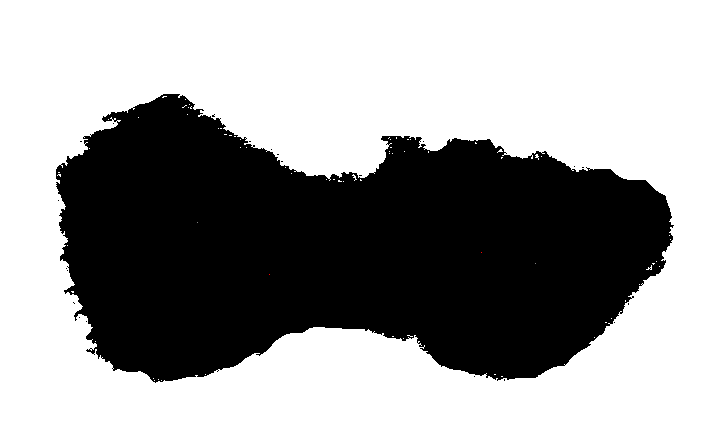

Supplement: Supplementary file 2 [file Datasheet2.zip › figshare/ImageIn/Experiment_074.tif]

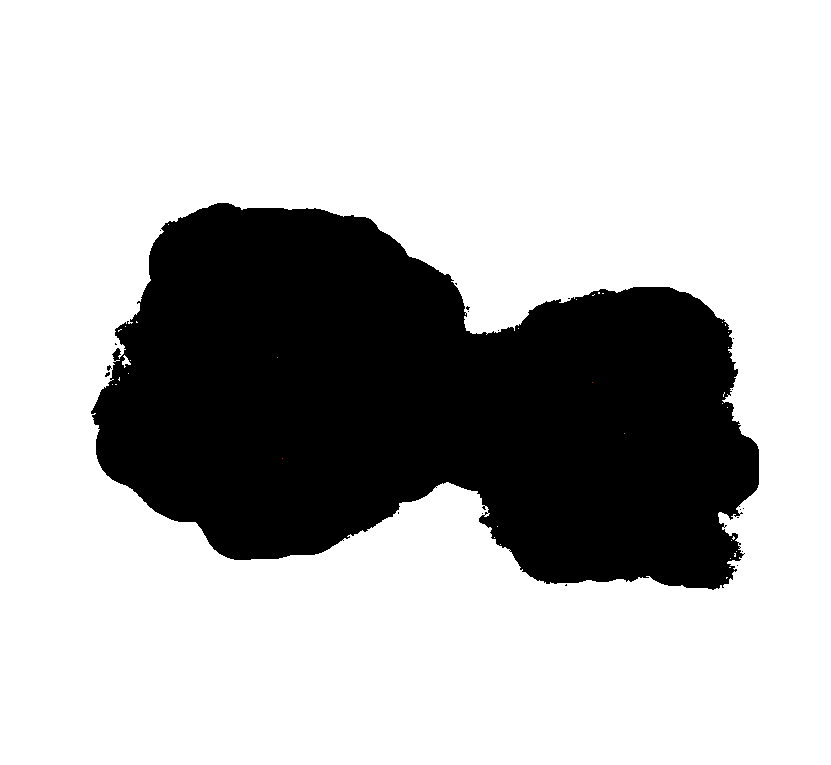

Supplement: Supplementary file 2 [file Datasheet2.zip › figshare/ImageIn/Experiment_075.tif]

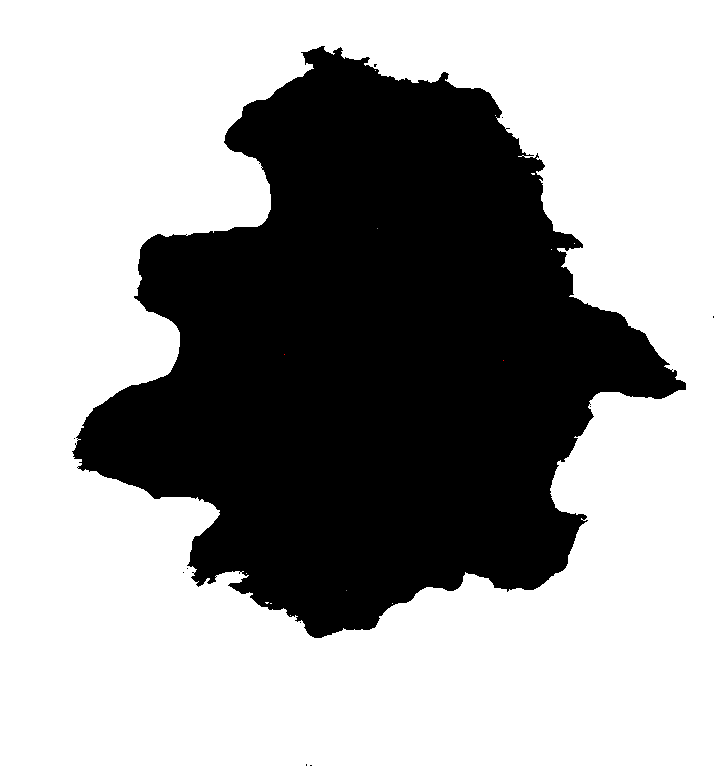

Supplement: Supplementary file 2 [file Datasheet2.zip › figshare/ImageIn/Experiment_076.tif]

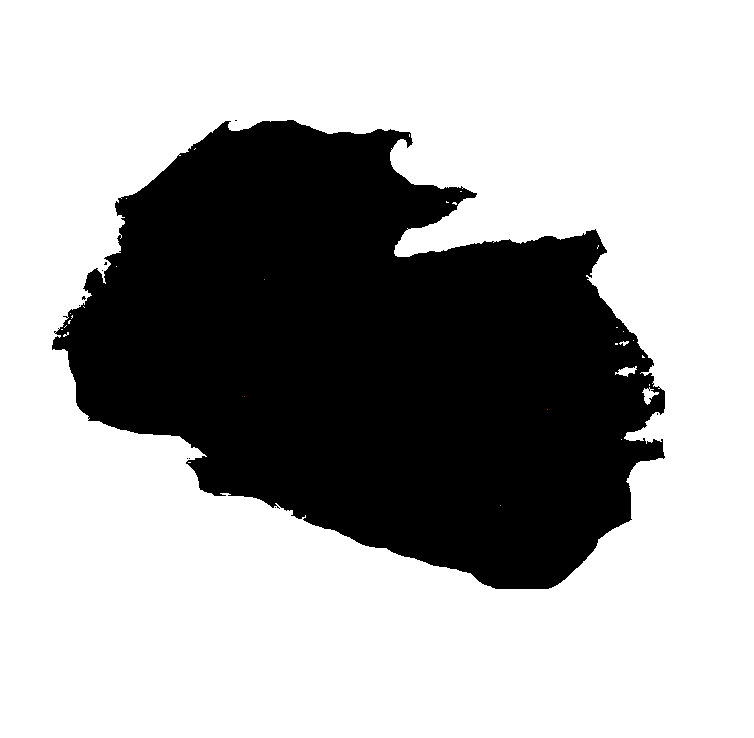

Supplement: Supplementary file 2 [file Datasheet2.zip › figshare/ImageIn/Experiment_077.tif]

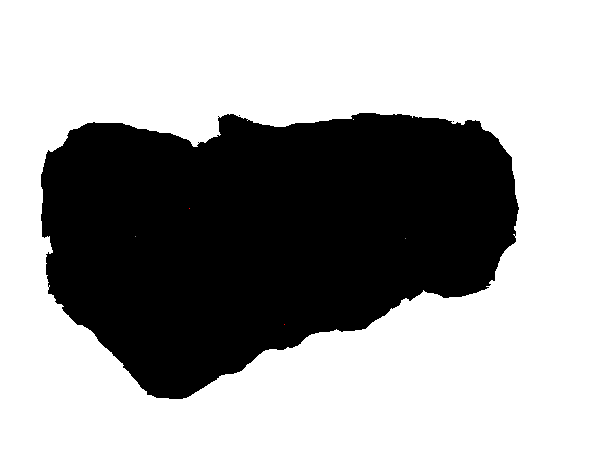

Supplement: Supplementary file 2 [file Datasheet2.zip › figshare/ImageIn/Experiment_078.tif]

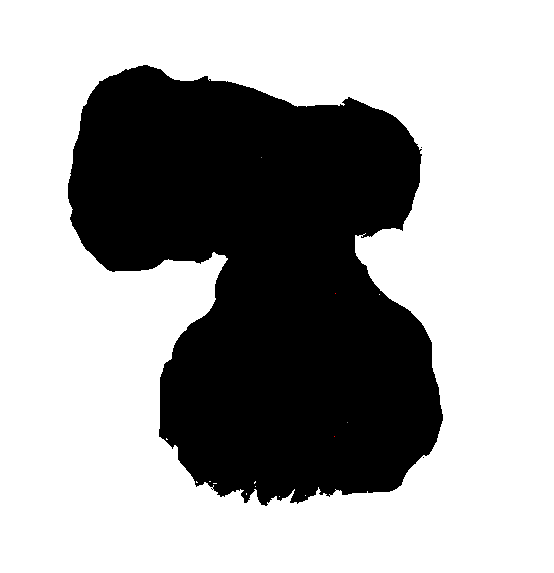

Supplement: Supplementary file 2 [file Datasheet2.zip › figshare/ImageIn/Experiment_079.tif]

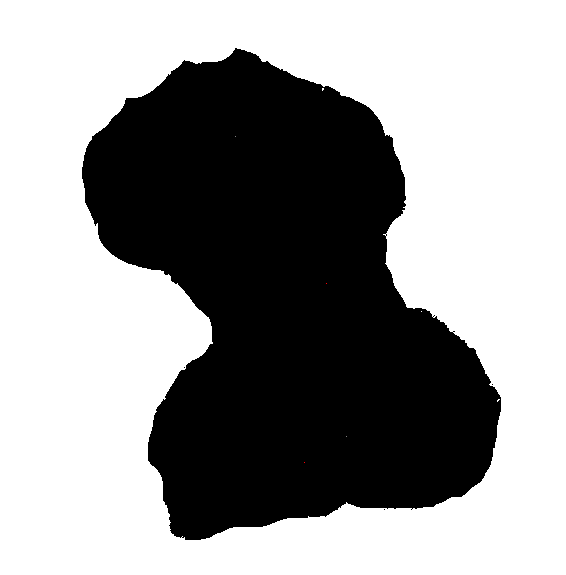

Supplement: Supplementary file 2 [file Datasheet2.zip › figshare/ImageIn/Experiment_080.tif]

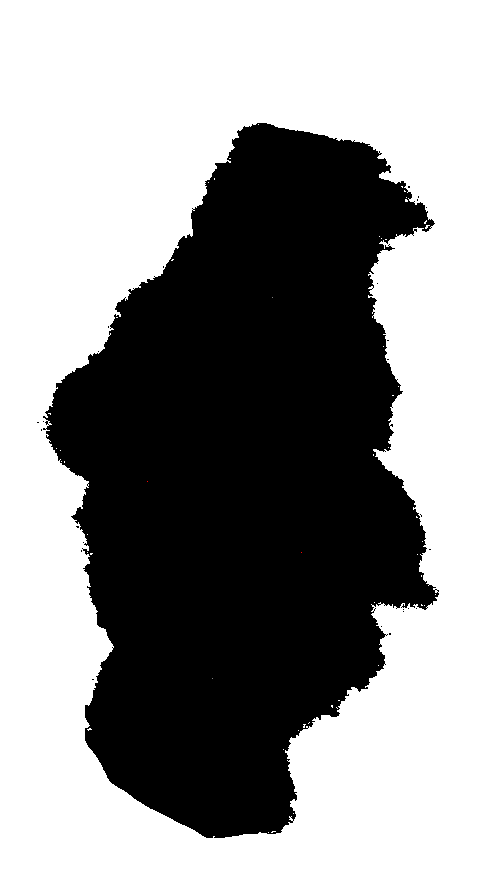

Supplement: Supplementary file 2 [file Datasheet2.zip › figshare/ImageIn/Experiment_081.tif]

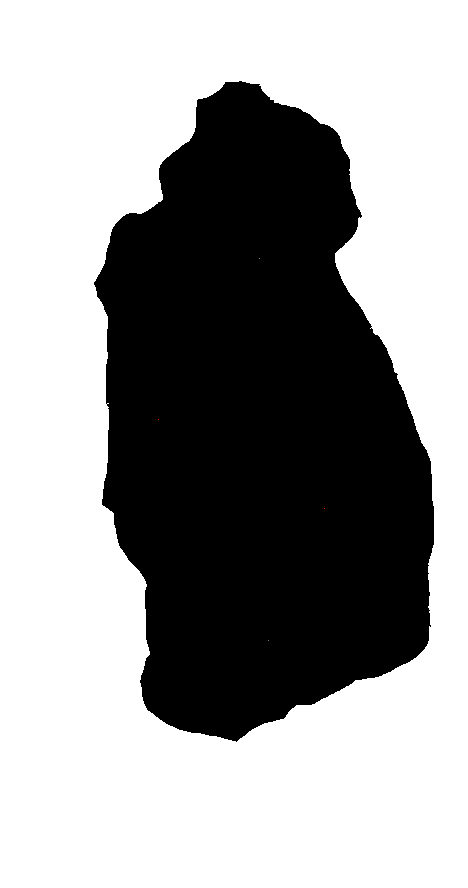

Supplement: Supplementary file 2 [file Datasheet2.zip › figshare/ImageIn/Experiment_082.tif]

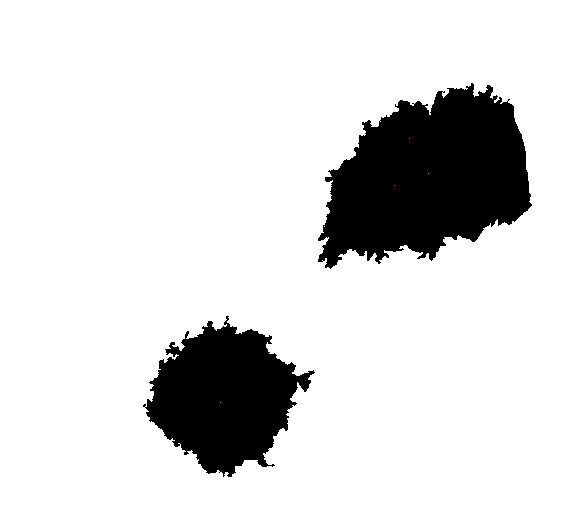

Supplement: Supplementary file 2 [file Datasheet2.zip › figshare/ImageIn/Experiment_083.tif]

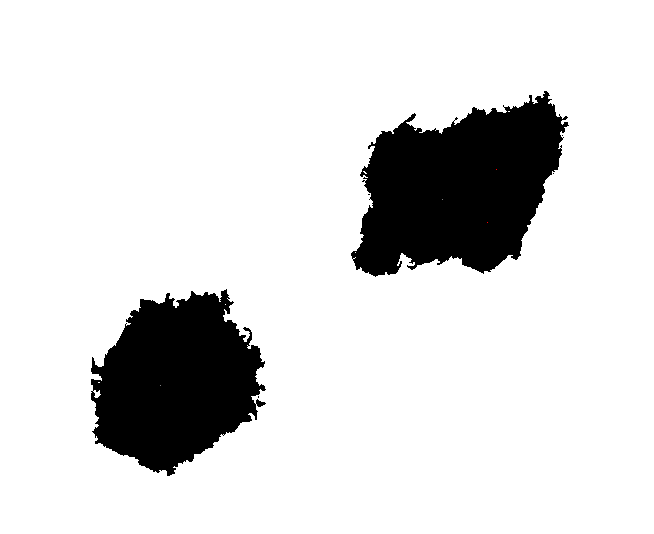

Supplement: Supplementary file 2 [file Datasheet2.zip › figshare/ImageIn/Experiment_084.tif]

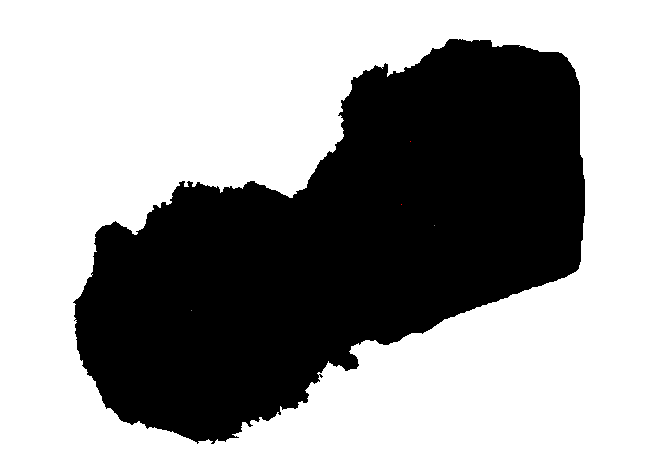

Supplement: Supplementary file 2 [file Datasheet2.zip › figshare/ImageIn/Experiment_085.tif]

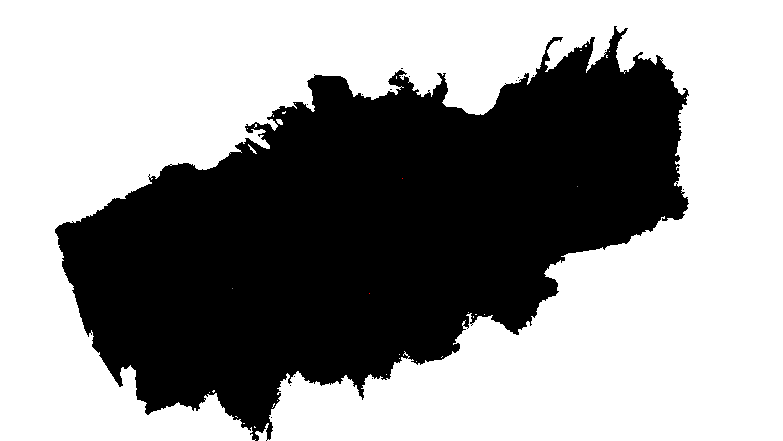

Supplement: Supplementary file 2 [file Datasheet2.zip › figshare/ImageIn/Experiment_086.tif]

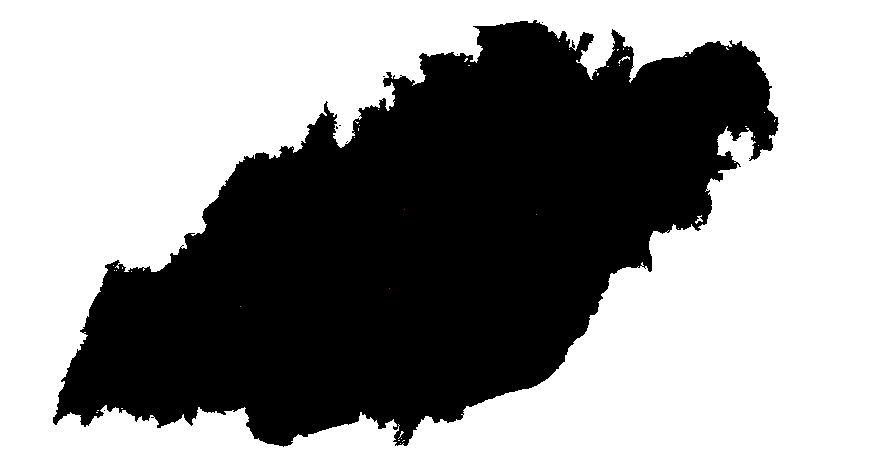

Supplement: Supplementary file 2 [file Datasheet2.zip › figshare/ImageIn/Experiment_087.tif]

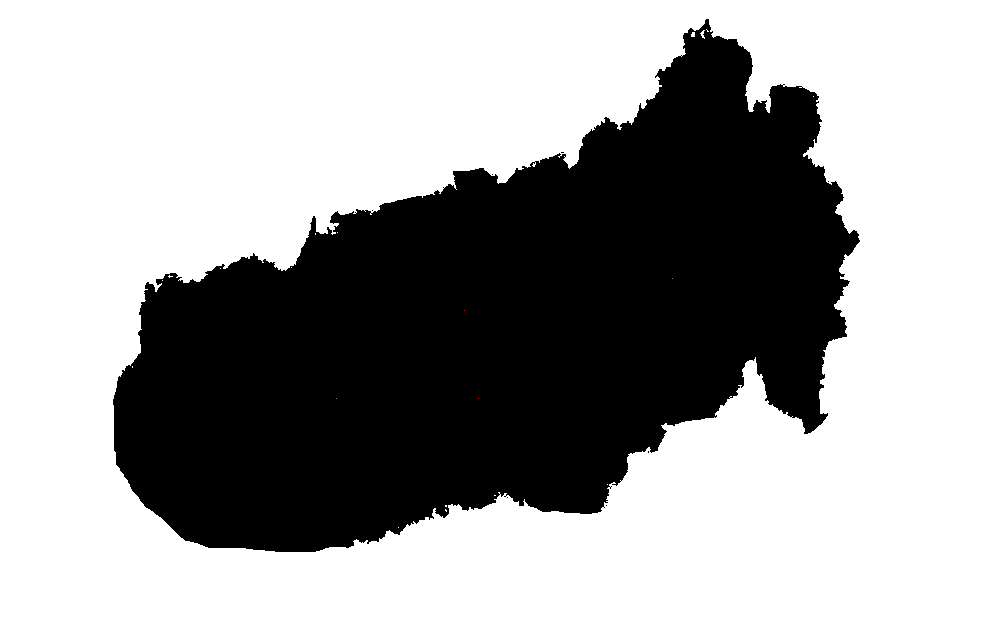

Supplement: Supplementary file 2 [file Datasheet2.zip › figshare/ImageIn/Experiment_088.tif]

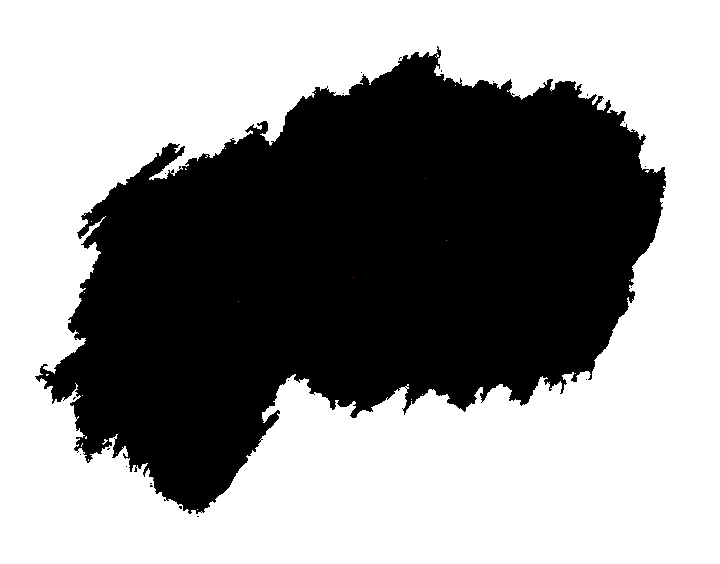

Supplement: Supplementary file 2 [file Datasheet2.zip › figshare/ImageIn/Experiment_089.tif]

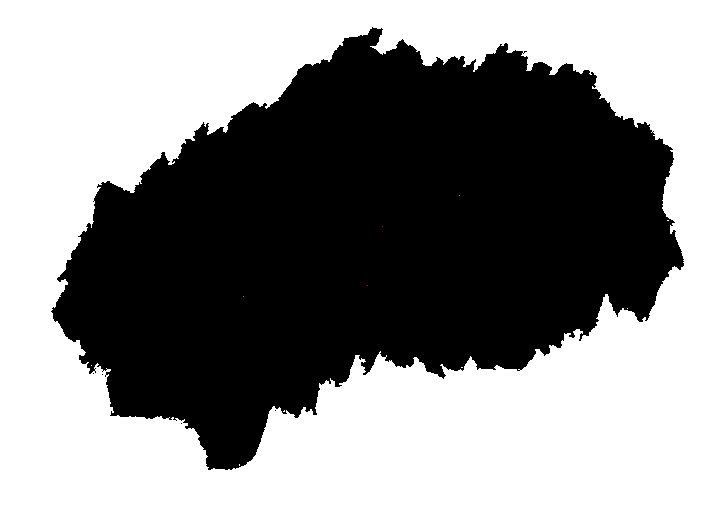

Supplement: Supplementary file 2 [file Datasheet2.zip › figshare/ImageIn/Experiment_090.tif]

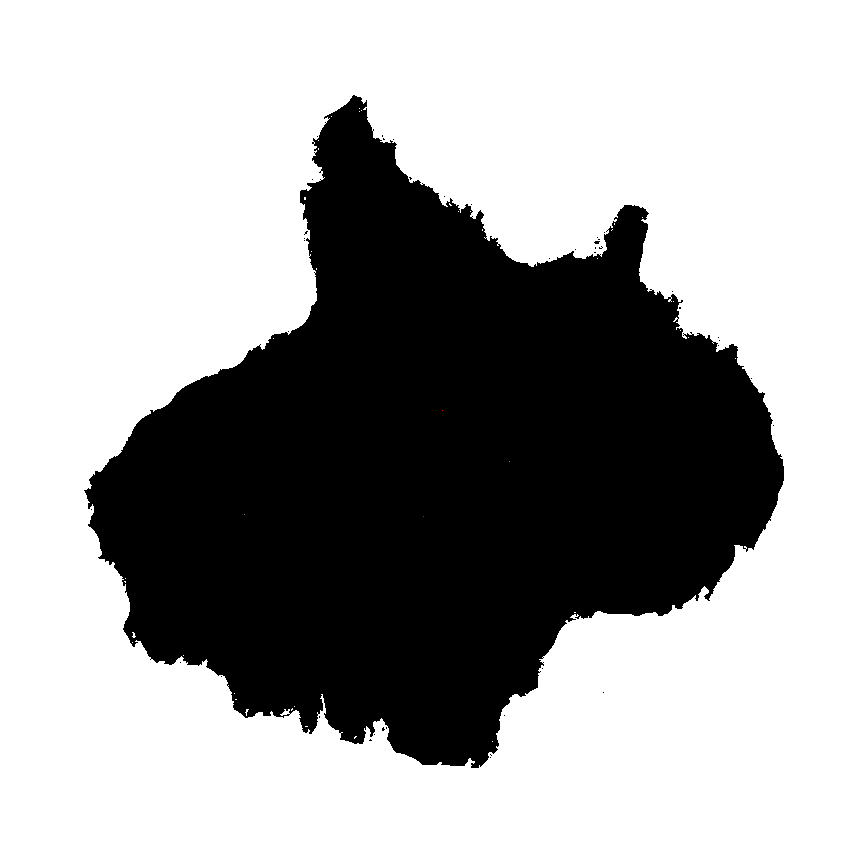

Supplement: Supplementary file 2 [file Datasheet2.zip › figshare/ImageIn/Experiment_091.tif]

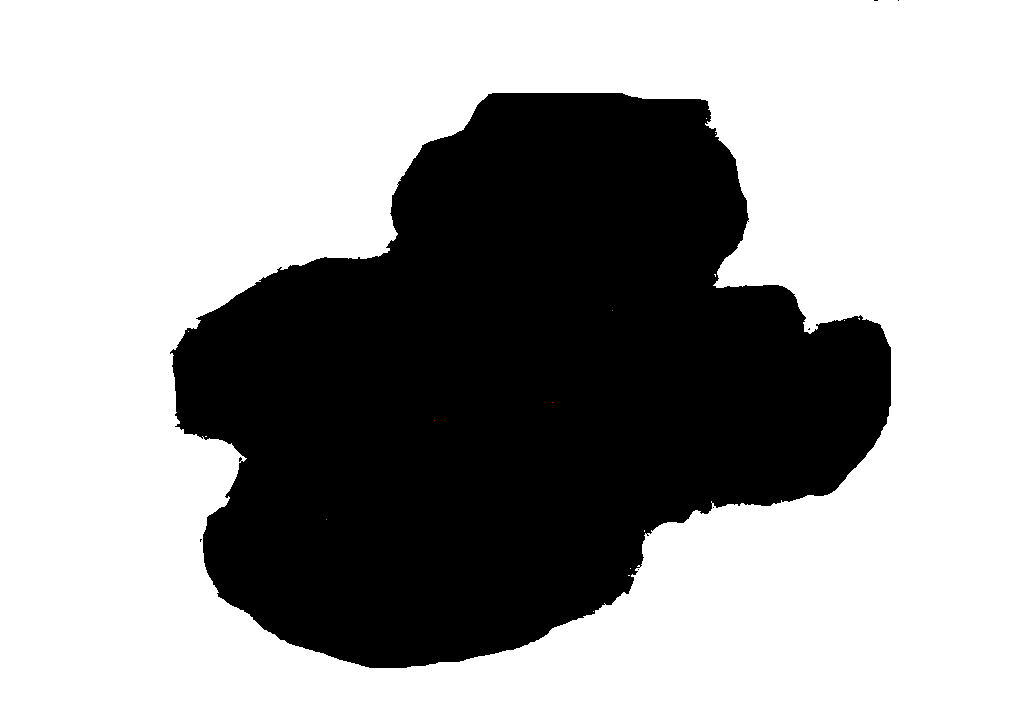

Supplement: Supplementary file 2 [file Datasheet2.zip › figshare/ImageIn/Experiment_092.tif]

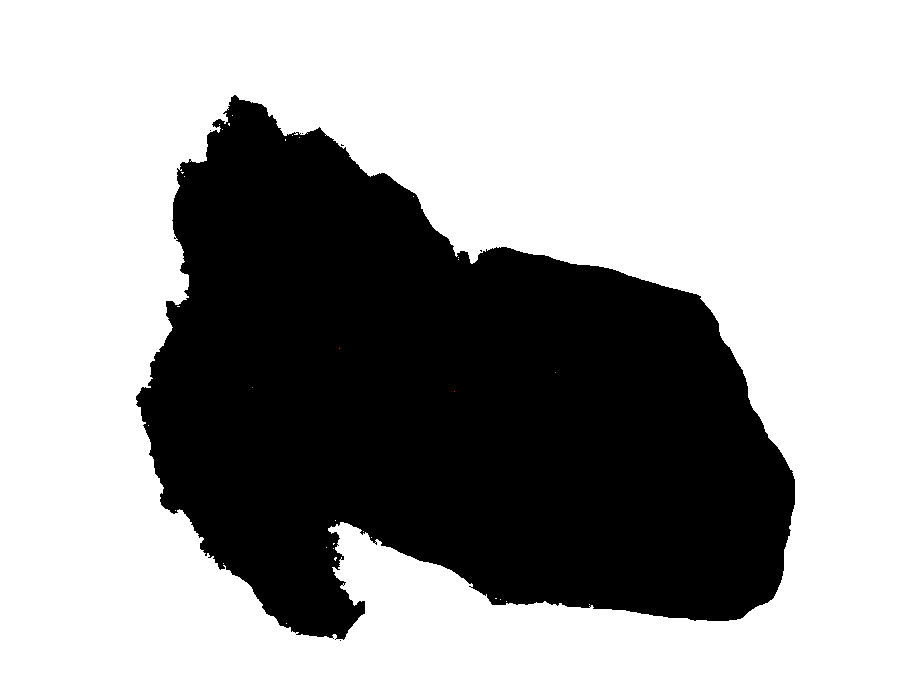

Supplement: Supplementary file 2 [file Datasheet2.zip › figshare/ImageIn/Experiment_093.tif]

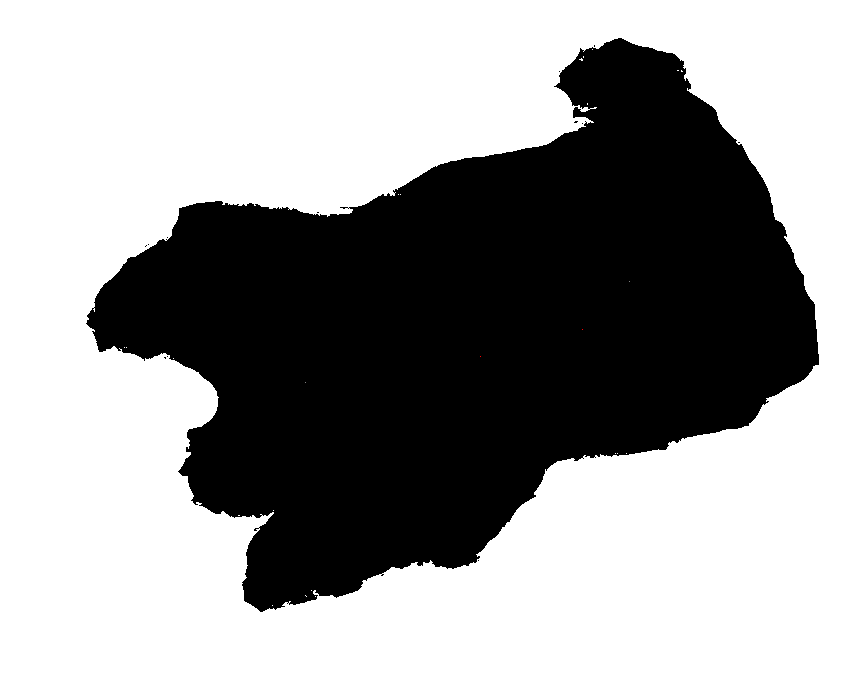

Supplement: Supplementary file 2 [file Datasheet2.zip › figshare/ImageIn/Experiment_094.tif]

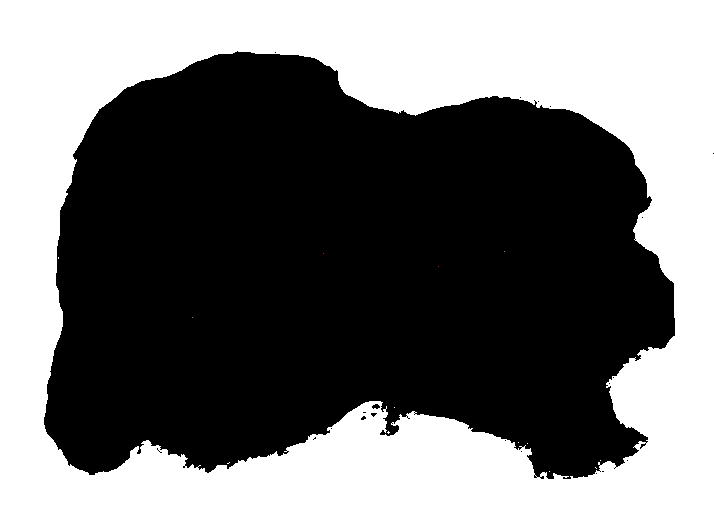

Supplement: Supplementary file 2 [file Datasheet2.zip › figshare/ImageIn/Experiment_095.tif]

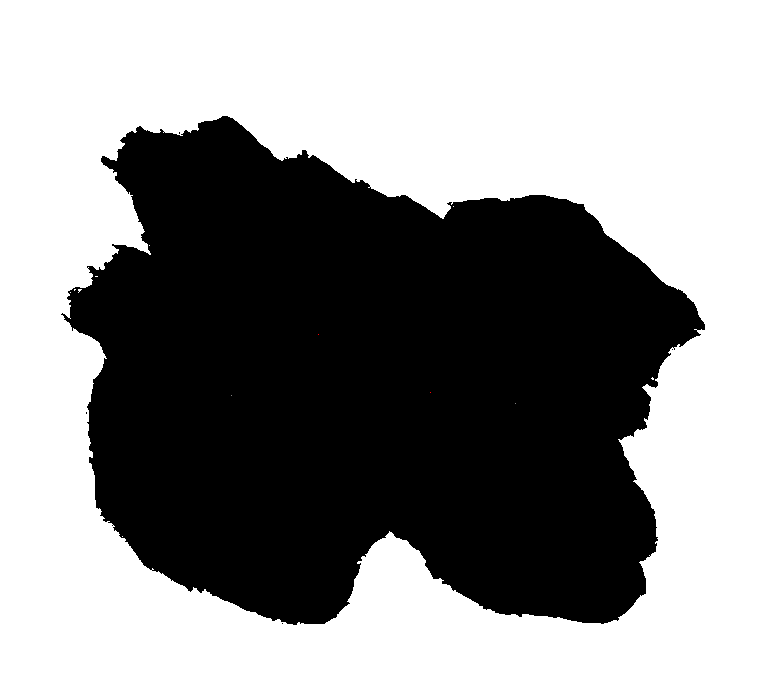

Supplement: Supplementary file 2 [file Datasheet2.zip › figshare/ImageIn/Experiment_096.tif]

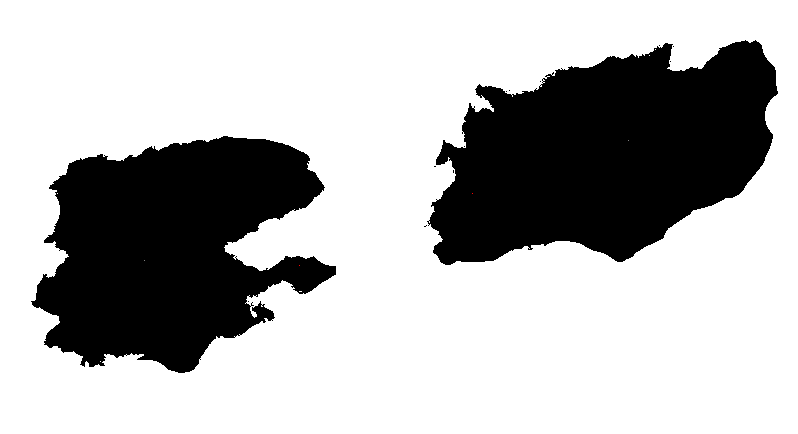

Supplement: Supplementary file 2 [file Datasheet2.zip › figshare/ImageIn/Experiment_097.tif]

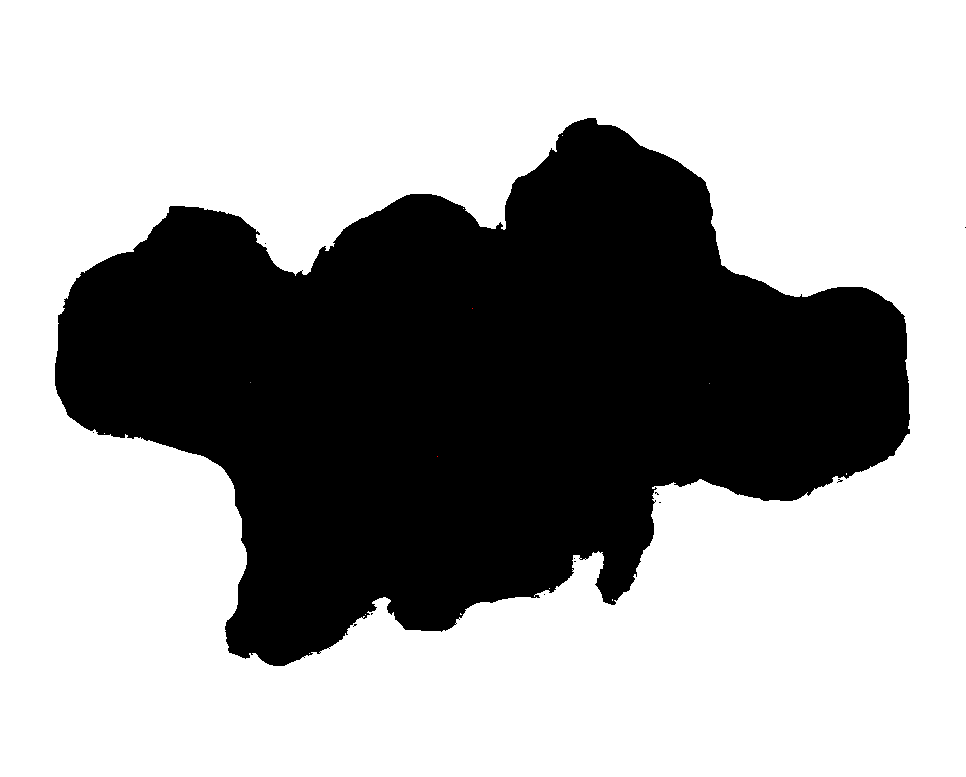

Supplement: Supplementary file 2 [file Datasheet2.zip › figshare/ImageIn/Experiment_098.tif]

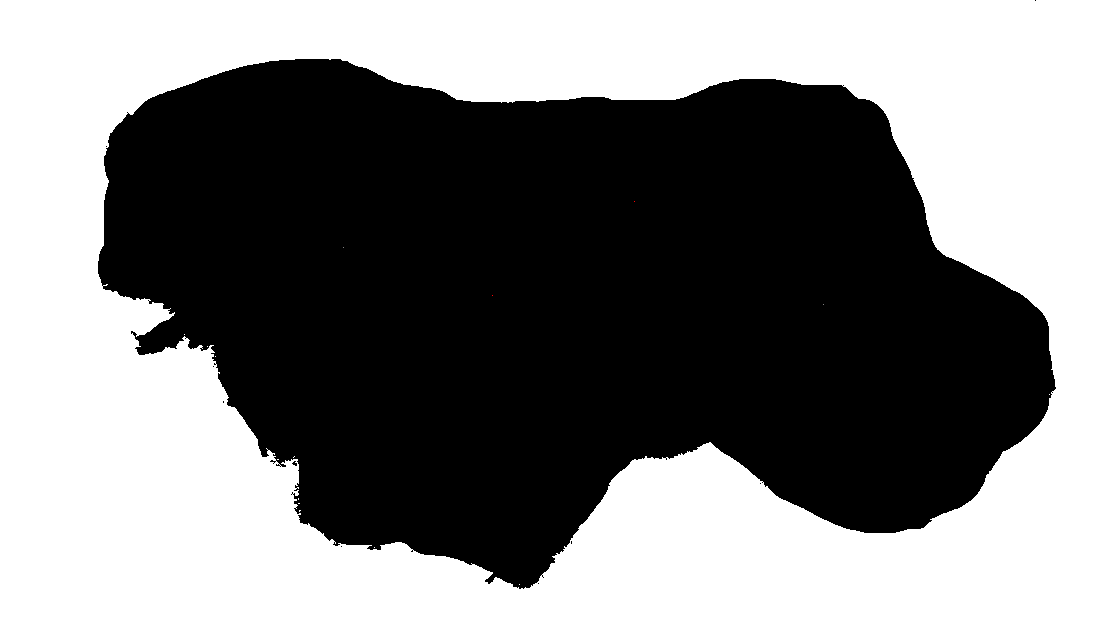

Supplement: Supplementary file 2 [file Datasheet2.zip › figshare/ImageIn/Experiment_099.tif]

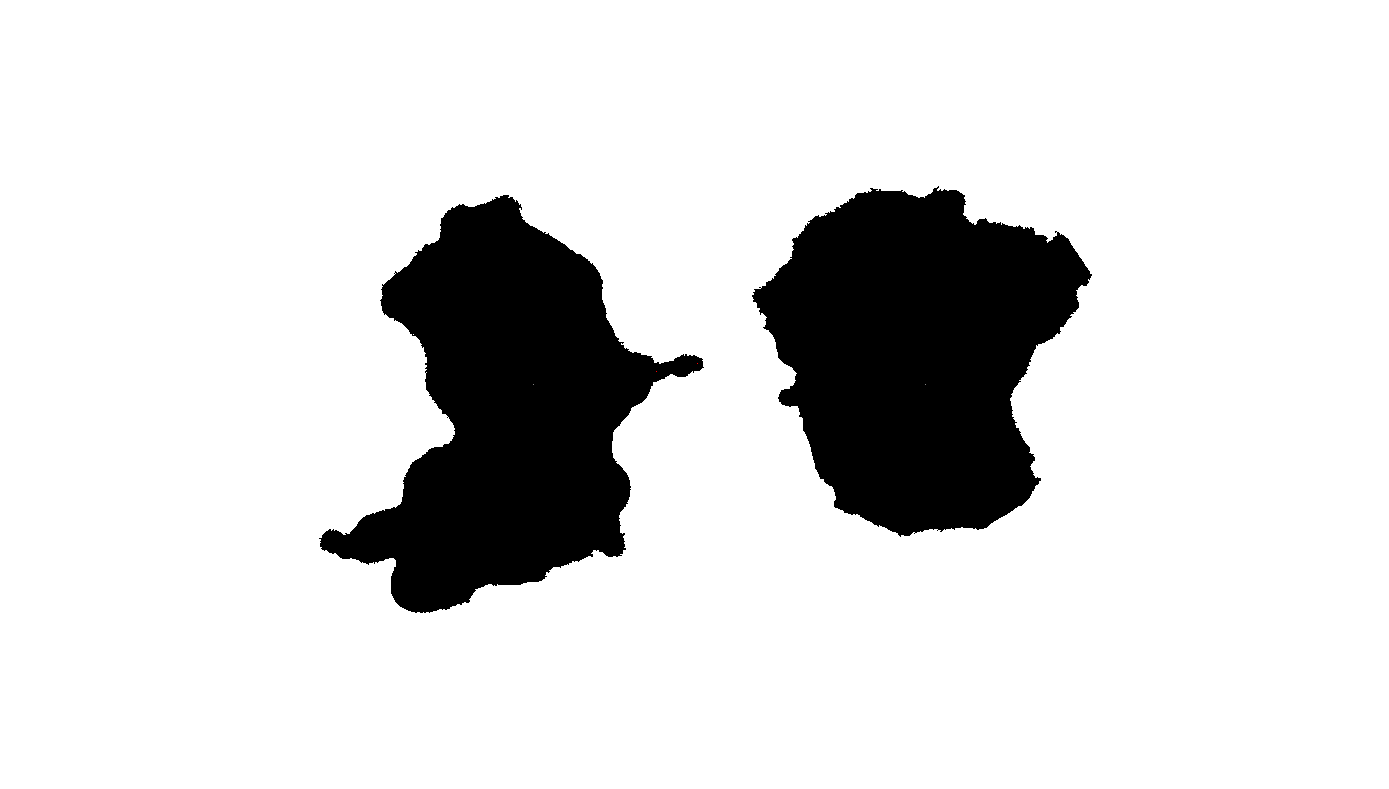

Supplement: Supplementary file 2 [file Datasheet2.zip › figshare/ImageIn/Experiment_100.tif]
